# Identification of the male-specific region on the guppy Y Chromosome from a haplotype-resolved assembly

**DOI:** 10.1101/gr.279582.124

**Published:** 2025-03

**Authors:** Kang Du, Oliver Deusch, Ilja Bezrukov, Christa Lanz, Yann Guiguen, Margarete Hoffmann, Anette Habring, Detlef Weigel, Manfred Schartl, Christine Dreyer

**Affiliations:** 1 Xiphophorus Genetic Stock Center, Institute for Molecular Life Sciences, Texas State University, San Marcos, Texas 78666, USA;; 2 Max Planck Institute for Biology Tübingen, Department of Molecular Biology, 72076 Tübingen, Germany;; 3 INRAE, LPGP, 35073 Rennes, France;; 4 Theodor Boveri Institute, Developmental Biochemistry, Biocenter, University of Würzburg, 97074 Würzburg, Germany;; 5 Research Department for Limnology, University of Innsbruck, 5130 Mondsee, Austria

## Abstract

The guppy Y Chromosome has been a paradigmatic model for studying the genetics of sex-linked traits and Y Chromosome–driven evolution for more than a century. Despite strong efforts, knowledge on genomic organization and molecular differentiation of the sex chromosome pair remains unsatisfactory and partly contradictory with respect to regions of reduced recombination. Especially the border between pseudoautosomal and male-specific regions of the Y has not been defined so far. To circumvent the problems in assigning the repeat-rich differentiated hemizygous or heterozygous sequences of the sex chromosome pair, we sequenced a YY male generated by a cross of a sex-reversed Maculatus strain XY female to a normal XY male from the inbred Guanapo population. High-molecular-weight genomic DNA from the YY male was sequenced on the Pacific Biosciences platform, and both Y haplotypes were reconstructed by Trio binning. By mapping of male specific SNPs and RADseq sequences, we identify a single male specific-region of ∼5 Mb length at the distal end of the Y (MSY). Sequence divergence between X and Y in the segment is on average five times higher than in the proximal part in agreement with reduced recombination. The MSY is enriched for repeats and transposons but does not differ in the content of coding genes from the X, indicating that genic degeneration has not progressed to a measurable degree.

Sex chromosomes evolve differently from the rest of the genome owing to reduced recombination. This process generates on Y Chromosomes a male-specific region (MSY) that is diverging in sequence and structural organization from the homologous region on the X. Because of reduced recombination, genes within the MSY can become restricted to the male sex (Khoo et al. 1999; Lindholm and Breden 2002). The guppy was the first vertebrate in which sex linkage of phenotypic traits was described. In this fish, males develop highly variable, yet heritable nuptial patterns. Genes that control the characteristic color traits could be genetically mapped on the X and Y Chromosomes (Winge 1922; Khoo et al. 1999; Lindholm and Breden 2002; Tripathi et al. 2009a). A genetic map based on EST- and BAC-derived markers was generated, and LG12 was identified as the sex chromosome (Tripathi et al. 2009a,b). Several markers including the most distal genetic marker M_229 (located in the cyclin I gene) (Tripathi et al. 2009a,b) differentiating X and Y were located by molecular cytogenetic analysis on the homologous chromosomes of different guppy populations and the two sister species *Poecilia wingei* and *Poecilia obscura* (Nanda et al. 2014). This analysis revealed polymorphisms in heterochromatin content of the Y as well as differences in distance of the genetic marker M_229 to the physical chromosome end between populations. The sex determination locus (SDL) was mapped to the most distal region of the Y (Winge 1922; Tripathi et al. 2009a,b), but its molecular identity has remained unknown. The ornamental male coloration is a combination of autosomal and many sex chromosome–linked genes (Winge 1927; Kirpichnikov 1981; Lindholm and Breden 2002; Paris et al. 2022; van der Bijl et al. 2024). Similarly, we still lack promising candidates for genes controlling the Y-linked pigmentation traits. In contrast to the MSY, the pseudoautosomal region (PAR) is found on both sex chromosomes and is considered as freely recombining between the X and Y Chromosomes. Yet, the literature contains conflicting results regarding the evolution of the different regions of the sex chromosomes as well as the extent of recombination suppression on the Y (Wright et al. 2017; Bergero and Charlesworth 2019; Darolti et al. 2019; Kirkpatrick et al. 2022).

Two whole-genome assemblies of the common (or Orinoco) guppy (*Poecilia reticulata*) from the Guanapo river in West Trinidad have been published previously: a female genome assembled from Illumina short reads (Künstner et al. 2016) and a male genome assembled from Pacific Biosciences (PacBio) Continuous Long Reads (CLR) (Fraser et al. 2020). A genetic map derived from crosses between individuals from Cumaná (Venezuela) and upper Quare river served to scaffold the assemblies to chromosome level (Tripathi et al. 2009a,b).

Unfortunately, the male guppy genome (Fraser et al. 2020) does not distinguish X and Y Chromosome (Chr) sequences and the maternal and paternal haplotypes of Chr 12 are collapsed into a single scaffold. To obtain a haplotype-resolved male genome, we made use of the fact that male-to-female sex-reversed XY individuals occur spontaneously in the Maculatus strain, likely caused by an as-yet-unidentified autosomal factor. Crosses to regular XY males can produce YY offspring, but these die as embryos, likely owing to a recessive lethal-effect mutation on the Maculatus Y. In XY males, this mutation is complemented by a gene on the X. The lethal-effect mutation on the Maculatus Y can also be complemented by alleles on Y Chromosomes from other populations (Winge 1922, 1938). Thus, to produce an adult YY male for genome sequencing, we crossed an XY female from the Maculatus strain to an XY male of the Guanapo strain.

Trio binning is a powerful method to sort PacBio sequencing long reads according to their maternal and paternal origin (Koren et al. 2018). Thus, we aimed at sequencing and assembling a chromosome-level genome of a YY male from an inter-populational cross using trio binning to resolve both Y Chromosome haplotypes. This genomic resource was expected to inform about the structure and gene content of the guppy Y Chromosome, in particular about the localization and extent of PARs, segments of reduced recombination, and male-specific regions.

## Results

### DNA sequencing and trio binning

**Figure 1.**
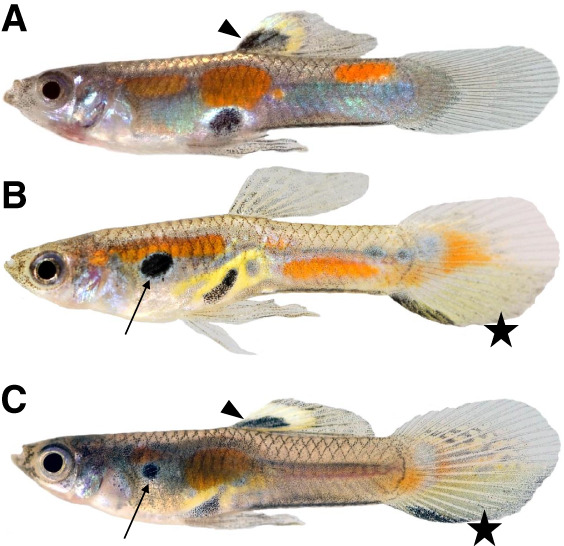
Adult male guppy phenotypes. (*A*) Male of the Maculatus strain, genotype X_mac_Y_mac_. (*B*) Male of the Guanapo strain, genotype X_Gua_Y_Gua_. (*C*) F_1_ male with sex chromosome constitution Y_gua_Y_mac_. Note that both the characteristic Maculatus black spot on the dorsal fin (arrowhead) and Guanapo black spot on the anterior trunk (arrow), as well as the Guanapo tail pattern (star), are expressed in the Y_gua_Y_mac_ male.

To obtain a Y haplotype-resolved assembly, we crossed a Guanapo male with a sex-reversed Maculatus XY female to obtain a YY F_1_ male offspring (Fig. 1A–C). This fish carried one Y Chromosome from the Guanapo population and the other one from the Maculatus population. The reconstructed Y haplotypes are designated Y_mac_ and Y_gua_.

Continuous long-read (CLR) sequencing on the PacBio Sequel II platform of high-molecular-weight DNA from this Y_mac_Y_gua_ male resulted in 170 Gb sequence, corresponding to approximately 226-fold genome coverage, assuming a genome size of ∼750 Mb (Table 1A). Because DNA samples from the actual parents were not available, we generated Illumina short-read data from another Maculatus XY female and from the genomic DNA that was used for the previously published Guanapo XY male genome (Table 1B; Fraser et al. 2020). Trio binning assigned 85.9 Gb to the Guanapo haplotype and 84.0 Gb to the Maculatus haplotype. Only 0.01 Gb could not be assigned to either haplotype, and 0.1 Gb was too short to be binned (Table 1A). BUSCO completeness was 98.0% for Guanapo and 97.9% for Maculatus, with 2.6% apparently duplicated in Guanapo and Maculatus, respectively (Table 1C).

**Table 1.** DNA sequencing overview

A. PacBio Sequel II (CLR)					
	Total bases (Gb)	No. of sequences	Max length (bp)	N50 (bp)	N90 (bp)
All reads	170.06	8,503,777	521,561	29,768	11,540
Guanapo	85.91	4,162,756	397,496	29,755	11,719
Maculatus	84.01	4,094,477	521,561	29,814	11,452
Unclassified	0.01	7328	22,512	1688	1097

B. Illumina (2 × 150 bp, PCR-free)		
Maculatus XY female	146 M cluster	44 Gb
Guanapo XY male	146 M cluster	43 Gb

C. Assembly statistics^a^							
	Total bases (Mb)	No. of sequences	Max length (bp)	N50	L50	BUSCO completeness	BUSCO duplicated
Guanapo	745.3	23	46,441,515	34,623,888	11	98.00%	2.60%
Maculatus	745.2	23	46,441,515	34,623,888	11	97.90%	2.60%

^a^These final genomes were assembled using the same reference genome as guide, resulted in highly similar statistics.

### Reconstruction of all autosomes from phased haplotype contigs

Trio binning allowed reconstructing all 23 chromosomes as phased haplotypes from the Guanapo and Maculatus strains, using the published guppy autosome sequences from the Guanapo XY individual as reference for scaffolding. We then aligned the resulting Guanapo and Maculatus autosomes to their orthologous *Xiphophorus hellerii* and *Xiphophorus maculatus* chromosomes as the most closely related species from which high-quality chromosome-level assemblies are available (Supplemental Fig. S1). These alignments showed, in general, an overall conservation of synteny of guppy autosomes to their *Xiphophorus* homologs (Lu et al. 2023). It also revealed that most of the inversions that distinguish the guppy and *X. maculatus* coincide in length and approximate position with inversions that were also observed between *X. maculatus* and *X. hellerii* (Supplemental Fig. 1 in Lu et al. 2023). As *X. maculatus* is a derived species and *X. hellerii* is more basal in the phylogenomic trees (Lu et al. 2023; Du et al. 2024), these inversions must be caused by events in the *X. maculatus* lineage. Confirming previous assemblies, the guppy female linkage group 2 combines medaka Chr 2 and 21 (Künstner et al. 2016). Chr 2 is a fusion of Chr 7 with Chr 24 (Fraser et al. 2020) of *Xiphophorus*, which represents the basal poeciliid karyotype (Cimino 1974). This fusion was also observed in the genome assembly of *Poecilia picta* (Metzger et al. 2021).

### Assembly, genomic organization, and gene content of the Y Chromosomes

As guidance for assembly of Chr 12, we used a modified version of the published Guanapo male Chr 12 (LR880656.1) (Fraser et al. 2020). Revisiting previously published Hi-C data (Fraser et al. 2020), we changed the order of the XY genome contigs (Supplemental Table S1). We used 11 Guanapo and nine Maculatus contigs >200 kb for a primary assembly of Y_gua_ (28.3 Mb) and Y_mac_ (27.6 Mb) (Supplemental Table S2; for details, see Supplemental Material).

To investigate the gene content for each Y haplotype, we conducted a genome annotation with an in-house pipeline adapted from a previous study (Du et al. 2022), which synthesizes evidence from homology aligning, transcriptome mapping, and ab initio gene prediction. On the Y_gua_ 1159 protein coding genes were annotated, with 932 (80.4%) showing transcriptome support and 1123 (96.9%) with BLAST hits in RefSeq (https://www.ncbi.nlm.nih.gov/refseq/) or Swiss-Prot database (https://www.uniprot.org/). On the Y_mac_, 1122 protein coding genes were annotated, of which 957 (85.3%) were transcriptome supported and 1090 (97.1%) had BLAST hits in RefSeq or Swiss-Prot (Table 2). Although there is congruence in general, discrepancies between both haplotypes are seen in which genes of one haplotype are on short unplaced contigs in the other haplotype. On the other hand, binning errors may have caused apparent gene duplications on Y_gua_.

**Table 2.** Annotation statistics of Y_gua_ and Y_mac_ MSY and pseudoautosomal region (PAR)

	MSY (23.5 Mb-end)		PAR (1–23.5 Mb)	
	Y_gua_	Y_mac_	Y_gua_	Y_mac_
Total genes	210	179	949	943
Single exon	16 (7.6%)	14 (7.8%)	60 (6.3%)	57 (6.0%)
Multiexon	194 (92.4%)	165 (92.2%)	889 (93.7%)	886 (94.0%)
Map to Pfam	161 (76.7%)	140 (78.2%)	770 (81.1%)	784 (83.1%)
Map to RefSeq/Swiss-Prot	199 (94.8%)	171 (95.5%)	924 (97.4%)	919 (97.5%)
With RNA support	57 (27.1%)	24 (13.4%)	108 (11.4%)	84 (8.9%)
Without start codon	35 (16.7%)	15 (8.4%)	50 (5.3%)	50 (5.3%)
Pseudo with RNA	16 (7.6%)	18 (10.1%)	64 (6.7%)	60 (6.4%)
Pseudo without RNA	19 (9.0%)	11 (6.1%)	39 (4.1%)	33 (3.5%)
Repeats	55.85%	54.17%	34.53%	34.48%

### Comparison of Y haplotypes to published female and male guppy Chr 12 sequences

**Figure 2.**
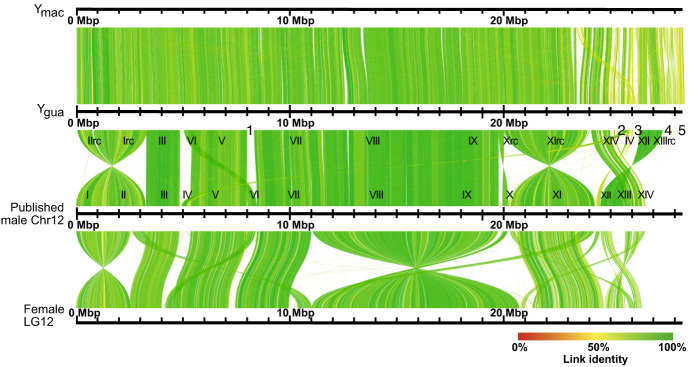
Alignment of Y_gua_ and Y_mac_ to published XY male Chromosome 12 (LR880656) and female LG12 (NC024342). Roman numbers specify positions of XY contigs on LR880656; for details see Supplemental Table S1. Positions of additional unplaced scaffolds of the XY assembly are indicated under Y_gua_: 1, 00013F_0; 2, 000200F_0; 3, 000250F_0; 4, 000181F_0; and 5, 000149F_0.

The reconstructed Y_gua_ and Y_mac_ haplotypes were aligned to each other and to the published chimeric X/Y male Chr 12 (LR880656.1) (Fraser et al. 2020), as well as to LG12 (NC_024342.1) from the XX female assembly (Fig. 2; Supplemental Fig. S2; Künstner et al. 2016). Alignment gaps between Y_gua_ and LR880656.1 are caused by several scaffolds that had remained unplaced in the previous assembly (000013F, 000200F, 000250F, 0000181F, and 000149F) (Supplemental Table S1; Fraser et al. 2020; Whiting et al. 2022).

A genetic linkage map based on SNP markers deduced from EST and BAC end sequences (Tripathi et al. 2009b) had served as guide for the assembly of both the previously published female and male genomes. The most distal sex chromosomal marker with consistently heterozygous SNPs in males but not females is M_229 (cyclin I, g876 on Ygua, g873 on Ymac) (Tripathi et al. 2009a,b; Lisachov et al. 2015; Bergero et al. 2019). Physical location of cyclin I and several other genetic markers has been confirmed cytologically by chromosome FISH with BACs containing these marker genes. This revealed consistent X and Y Chromosome organization in guppies of widely diverging geographic origin (Nanda et al. 2014).

**Figure 3.**
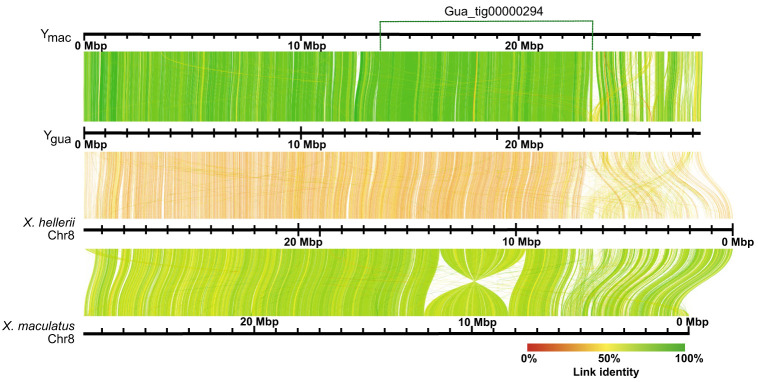
Alignment of Y_gua_ and Y_mac_ haplotypes to *Xiphophorus* Chromosome 8. A 3.5 Mb inversion distinguishes *X. hellerii* and *X. maculatus*. The position of the long contig Gua_tig00000294 that spans the inversion of *X. maculatus* Chr 8 is indicated at the *top*. The position of a short proximal sequence of the guppy Y also conforms to *X. hellerii* Chr 8, whereas it is translocated to the chromosome end in *X. maculatus*. Y_gua_ and Y_mac_ conform to one another and to *X. hellerii* Chr 8, with most divergence seen from 23 Mb to the end of guppy Y haplotypes.

**Figure 4.**
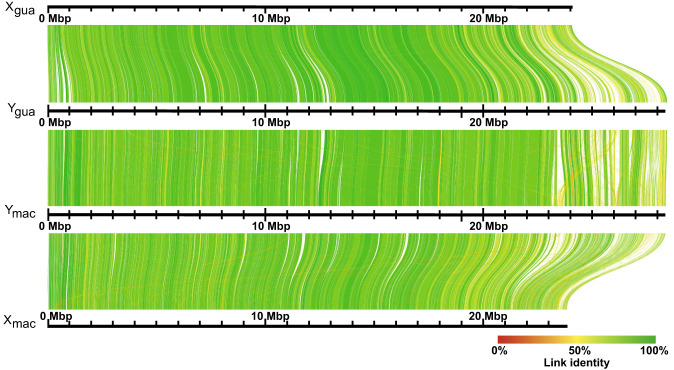
Alignment of reconstructed X Chromosomes to Y_gua_ and Y_mac_ haplotypes. Y_gua_ and Y_mac_ assembled as described in Supplemental Table S2 are aligned to X_gua_ and a virtual X_mac_. Both X Chromosomes were rescaffolded from the female Guanapo contigs using Y_gua_ and Y_mac_ as templates.

Alignments of *X. maculatus* Chr 8 (Lu et al. 2023), which is homologous to guppy Chr 12, to the Y_gua_ and Y_mac_ haplotypes revealed a long inversion of ∼3.8 Mb, as also seen with LR880656.1 (Supplemental Fig. S8 in Fraser et al. 2020). In both haplotypes, this inversion is found in the middle of >9-Mb-long contigs, Gua_tig00000294, and Mac_tig00000028, respectively. An inversion of similar length and position also distinguishes *X. maculatus* and *X. hellerii* Chr 8 (Lu et al. 2023). When Y_Gua_ was aligned to *X. hellerii* Chr 8, it turned out that the inversion is specific to *X. maculatus*, suggesting that guppy Chr 12 is more similar to *X. hellerii* Chr 8, which represents the ground state for *Xiphophorus* (Fig. 3). To validate our inferences, we scaffolded the female Chr 12 from existing female contigs (Künstner et al. 2016) using the Y_gua_ and Y_mac_ haplotypes as guides. This resulted in a reconstructed Guanapo X Chromosome (X_gua_) and a virtual Maculatus X Chromosome (X_mac_). Alignments (Fig. 4) revealed that the large inversion previously detected between the two Y haplotypes and the published LG12 (Fig. 2; Künstner et al. 2016) was almost certainly caused by a previous scaffolding error. This inversion was also noted in the assembly of the male guppy genome (Fraser et al. 2020) and by comparison to the assembly of *P. picta* (Metzger et al. 2021). Total chromosome lengths differ by ∼4.2 Mb between the Guanapo X and Y reconstructions. This difference is mainly caused by Y-sequence regions lacking counterparts on X, especially near the chromosome ends (Supplemental Figs. S3, S4).

### Identification of the male-specific region (MSY) on both Y Chromosomes

**Figure 5.**
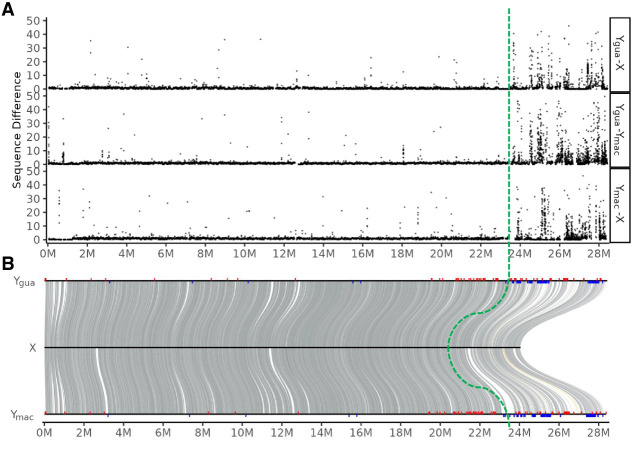
Sequence divergence between Y haplotypes and the reconstructed X_gua_. (*A*) Sequence difference calculated as the number of SNPs and indels in 10 kb sliding windows along the Y Chromosomes. (*B*) Y_gua_ and Y_mac_ are aligned to the X. Male-specific RAD-tags from Caroni swamp guppies (blue bars) and from four different guppy strains (red bars) were mapped on the aligned Y_gua_ (*top*) and Y_mac_ (*bottom*) sequences. The four strains represent a broader range of geographic origins, from East Trinidad to West Venezuela. About 70% of these RAD-tags (red bars) had best hits (*e*-40) within the distal 16% of the Y (>24 Mb). Both Y haplotypes are aligned to the reconstructed X_gua_ (*middle*). The green dotted line indicates the congruent MSY identified by sequence divergence and RAD-tags from the Trinidad populations of guppy.

In another attempt to identify the MSY on Y_gua_ and Y_mac_, we first aligned both to the reassembled version of the female Chr 12, which showed that both Y Chromosomes (Y_gua_ and Y_mac_) diverged from the X Chromosome at the distal end with a sharp transition at ∼ 23.5–24 Mb (Fig. 5A) The average divergence in the distal end is 6% sequence difference (SNPs and indels), around fivefold higher than in the proximal part (average, 1%). Although the higher divergence of the distal part is consistent with lower effective recombination, in agreement with known features of the MSY, our data do not support that there is a second region between 15 and 21 Mb, where X and Y do not recombine (“stratum 2”), as has been postulated previously (Wright et al. 2019), nor that there is an even more proximal nonrecombining region (“MSNR1”), as inferred from meiotic mapping (Tripathi et al. 2009a). The genomic structure of the guppy sex chromosomes with a 4 Mb large region of heterozygosity considerably higher compared with autosomes and the proximal 14 Mb of LG 12 showing autosomal sequence divergence levels is also not in line with previous studies on recombination rates (Bergero et al. 2019) that proposed a single small (1–2 Mb) PAR at the tip of the Y Chromosome. These discrepancies may be explained by the usage of other populations and even different guppy species as parents for mapping crosses (*P. obscura*, *P. wingei*).

To further confirm the location of the MSY, male-specific RAD-tags were generated from four different guppy strains (including three populations from East and West Trinidad and from Venezuela) (Supplemental Data Set 1) and mapped to the assembly (Fig. 5B, red bars). These specific RAD-tags are enriched in the distal region from 20 Mb to the very end at 28.5 Mb. Adding RAD-tags from a separate analysis of guppies from the Caroni swamp in Trinidad reduced the MSY candidate region further to the 23.5 to 28.5 Mb interval (Fig. 5B, blue bars). The different proximal borders of the MSY delineated by the two RAD-tag data sets could indicate that suppression of recombination expanded differently in different populations, a phenomenon noticed frequently in other species (McKinney et al. 2020; Hallast et al. 2023). In a different approach using Pool-seq data for calling male-specific SNPs from a related species, *P. obscura* (Supplemental Data Set 2), also the 24 to 25 Mb segment has a higher SNP density than the rest of the chromosome (Supplemental Fig. S5). The most distal region from 26 to 28.5 Mb exhibits a much lower density of male-specific SNPs than the rest of the chromosome. The absence of SNPs at the very end may be caused by alignment failures between the reference genome and the Pool-seq reads owing to a high divergence of Y Chromosomes between the two species. Length polymorphism between the distal regions of X and Y and between Y Chromosomes of different populations has been documented by fluorescence in situ hybridization and C-banding (Nanda et al. 1990, 2014). However, the region between 24 and 25 Mb appears to be a conserved MSY between *P. reticulata* and *P. obscura*.

Studies of sex chromosomes in many species have revealed that, owing to reduced recombination, transposable elements accumulate in the MSY (Chalopin et al. 2015; Štundlová et al. 2022). Both guppy Y Chromosomes have an increased repeat content in the terminal region (22–28 Mb) (Supplemental Table S3), with these elements being significantly younger than those in the proximal part (Supplemental Fig. S6). Such sharp borders were also seen in sex chromosomes of seahorse (Long et al. 2023) and splitfin (Du et al. 2022) without any inversion identified. Accumulation of helitron-4 elements occurred on different locations in the MSY of Y_gua_ and Y_mac_, indicating independent evolution of the Y Chromosome in different populations of the guppy.

The MSY contains several genes that are known to be related to sperm structure and function and the gene encoding sepiapterin reductase (*spra*), which is a key enzyme for the production of the red and yellow pigment of erythrophores and xanthophores (Table 3; Braasch et al. 2007; Schartl et al. 2016).

**Table 3.** Genes with functions related to male gonadal development and pigmentation in the MSY region

Y_mac_ start	Y_mac_ end	Y_gua_ start	Y_gua_ end	Gene annotation	Comments on function and expression
23.793.174	23.796.136	23.577.725	23.580.685	Sperm-associated antigen 8-like	Cell cycle regulation during spermiogenesis
24.828.972	24.829.886	24.651.601	24.652.515	Growth arrest and DNA damage-inducible protein GADD45 gamma	Regulates SRY; sex reversal candidate
24.833.872	24.834.948	24.656.498	24.657.585	Growth arrest and DNA damage-inducible protein GADD45 gamma	Growth arrest at puberty; male-specific trait
24.940.636	24.943.646	24.771.398	24.774.385	Prostate androgen-regulated mucin-like protein 1 (PARM)	Target of androgen
25.311.394	25.334.880	25.141.772	25.165.256	Cilia- and flagella-associated protein 44	Sperm motility
25.856.670	25.863.321	25.773.319	25.780.388	Cyclin-I isoform X1 [*Poecilia reticulata*]	Last genetic marker 229; male-specific SNPs
25.979.809	25.982.063	25.987.747	25.993.537	Spindlin-1-like [*Xiphophorus maculatus*]	Ssty domain; spermiogenesis
26.568.289	26.572.876	26.574.475	26.579.107	Flagellar WD repeat-containing protein Pf20-like	Sperm motility
27.005.425	27.007.897	27.199.913	27.202.448	Sepiapterin reductase	Red and yellow pigment
27.061.485	27.081.388	27.260.493	27.281.378	Outer dense fiber protein 2	Sperm motility
27.302.394	27.309.962	27.453.812	27.462.891	LMBR1 domain-containing protein 2	Male bias expression in gonad
27.329.422	27.332.468	27.484.292	27.487.357	Cilia- and flagella-associated protein 53 [*P. reticulata*]	Sperm mobility; male bias
28.069.253	28.075.419	28.176.114	28.182.692	Hydroxysteroid dehydrogenase-like protein 2 isoform X1	Steroid metabolism

Positions (in base pairs) are listed for Guanapo and Maculatus haplotypes.

Comments on function and expression are based on an analysis of gene annotations. For expression patterns, see Supplemental Table S4.

To further characterize the guppy MSY, we reanalyzed a transcriptome data set from male and female adult tissues (Sharma et al. 2014) and identified on the entire Y_gua_ Chromosome 45 genes with significant male expression bias in the gonads. Of these, 33 are also male biased in brain and tail tissue. Male biased genes in the distal region of the Y Chromosomes include three genes associated with spermatogenesis, namely, *sperm associated antigen 8*, *flagellar Pf20-like*, and *cilia- and flagella-associated protein 53* (Supplemental Table S4; for a comparison of all genes annotated on the Y_gua_ and Y_mac_ haplotypes, see Supplemental Table S5). Genetic mapping predicted the putative SDL of the guppy to be located distal of the *cyclin I* gene (Tripathi et al. 2009b), which is located at 25.78 Mb of Y_gua_. Therefore, the SDL might be located between *cyclin I* and 27 Mb, provided that the information from the crosses between Cumaná and Upper Quare guppies (probably laboratory-reared *P. obscura*) applies also to Guanapo guppies.

In search of Y-specific genes, we then generated a list of nine genes that occur on both Y Chromosomes between 22.5 and 27 Mb but seem to be missing or are defective on the X Chromosome (Supplemental Table S6). This includes genes in ∼3 Mb of sequence proximal of *cyclin I* that is rich in sex-specific RAD markers and male SNPs (Fig. 5B). When we attempted to validate by PCR and screening female raw reads and female Pool-seq reads the candidate genes differentiating the X and Y Chromosomes, we could generally not confirm that they were missing from the X Chromosome (Supplemental Tables S6, S7). Also, manual curation of the MSY assembly and exon annotation revealed no difference in the content of coding genes compared to the homologous region of the X.

## Discussion

We made use of the long-known observation that sex-reversed XY females occur spontaneously in the Maculatus strain of the guppy to produce viable males with two different Y Chromosomes. This enabled us to assemble the both the Y_gua_ and Y_mac_ from long-range sequence data using the powerful technique of Trio binning. The conserved MSY could be narrowed down to a few megabases of sequence length. The divergence pattern of the sex chromosome pair clearly identified only a single stratum on the guppy Y. Despite a considerable molecular differentiation of the MSY to the corresponding region of the X, our analysis revealed no structural or functional gain of annotated coding genes on the MSY, nor did we find evidence for genes found in the homologous region of the X being lost on Y. Obviously, genic degeneration has not proceeded to a noticeable degree, and no Y-specific coding gene that would act as a master male determining factor is evident from the assembly of the MSY. It cannot be excluded that such a gene failed to be assembled or missed in the annotation. On the other hand, allelic variation on the coding sequence level or in noncoding, regulatory regions of a gene present on the X and the Y could be responsible for a sex determining function (e.g., see Tang et al. 2022). Allelic variation is a common origin of sex determination (SD) genes in fish (Kitano et al. 2024). To detect such minor differences in the X and Y sequences may require the combination of more sequencing at highest possible fidelity of more individuals from different populations and different guppy species to distinguish private SNPs and sequence variation from those that are conserved across all individuals that share the same SD gene and genes encoding sex-linked traits. One possible strategy could be amplicon sequencing targeting X and Y Chromosome ends (Kersten et al. 2023). Crossing males from other populations to Maculatus X Y_mac_ females to produce a range of Y_mac_Y_x_ males would allow comparison of different Y Chromosomes to the Y_gua_ described here. Sequence comparisons between the MSY of the Y_gua_ and the corresponding regions of other Y Chromosomes could also reveal the most likely candidate genes for Y-linked pigmentation patterns and other traits shared by different populations.

An important role in sex chromosome evolution is assigned to genes that are beneficial for one and/or detrimental to the other sex (Kirkpatrick and Guerrero 2014). We identified several spermatogenesis and pigmentation genes on the Y Chromosome. Some are located in the MSY (Table 3), whereas others reside in the PAR (e.g., spermatogenesis genes *morn3*, *strbp*, pigmentation genes *slc45a2* [*aim1*], *skiv2l2*, *slc31a1*). The genes in the MSY are also present in the corresponding region of the X. For the pigmentation genes (e.g., *spra*), we cannot say how they are related to male ornaments, because many male colors are encoded on the X and the PAR of the Y (Kirpichnikov 1981). Their expression, however, is dependent on a high testosterone level (Nanda et al. 2022). Future studies are necessary to reveal whether the X and Y alleles of these pigmentation candidate genes are divergent and under androgen control.

In the common guppy *P. reticulata*, the sex chromosome pair has been assigned to linkage group 12 (Künstner et al. 2016; Dor et al. 2019), and its homology with the sex chromosomes of its sister species within the subgenus *Acantophacelus*, namely, *P. obscura* and *P. wingei*, has been shown (Nanda et al. 2014; Morris et al. 2018). Also in the distantly related species *P. picta* and *Poecilia parae* (subgenus *Micropoecilia*), the Y Chromosome is derived from linkage group 12 (Bergero et al. 2019; Charlesworth et al. 2020; Metzger et al. 2021). However, in these two taxa, in strong contrast to *P. reticula*, the Y Chromosome is highly degenerated at the molecular and morphological level (Darolti et al. 2019; Charlesworth et al. 2021a,b; Nanda et al. 2022). Two opposing explanations have been put forward: either a common origin but different degrees of recombination suppression and, consequently, speed of degeneration (Darolti et al. 2019, Fong et al. 2023) or convergent evolution of the same ancestral linkage group to independently become the sex chromosome (Charlesworth et al. 2021a; Kirkpatrick et al. 2022). In this scenario, Y's have independently evolved from the same linkage group and at different evolutionary times in the lineages of *P. reticulata*/*P. obscura/P. wingei* on the one hand and *P. picta*/*P. parae* on the other hand. The availability of a high-quality reference genome of the common guppy with a fully assembled Y can foster population genomic studies for investigating the evolution of Y Chromosomes in this unique model species and should help to resolve the discrepancies from previous studies on sex chromosome structure and their evolutionary origin (Bergero et al. 2019; Darolti et al. 2019; Kirkpatrick et al. 2022).

## Methods

### Fish sampling and aquaculture

We made use of the fact that in the *P. reticulata* Maculatus laboratory strain (WLC 1250), spontaneous XY females arise spontaneously, which can be diagnosed by the Y-encoded black spot in the dorsal fin. Such a sex-reversed XY female was crossed to a XY male of the *P. reticulata* Guanapo strain (WLC 5856). In the resulting F_1_ generation, the YY males were identified by their coexpression of the distinct Maculatus and Guanapo pigmentation patterns. One of these males (ID 5944-1) was used for sequencing. These fish were raised at the fish facilities of the Biocenter of the University of Würzburg following approved experimental protocols through an authorization (568/300-1870/13) of the Veterinary Office of the District Government of Lower Franconia, Germany, in accordance with the German Animal Protection Law (TierSchG).

### DNA sequencing

For long-read sequencing, high-molecular-weight genomic DNA prepared from a Y_gua_ Y_mac_ male was analyzed by pulse field gel electrophoresis, which revealed a main peak ∼165 kb. After gentle shearing using an Eppendorf blue tip, the DNA was purified and concentrated using Ampure beads. After size-fractionation on a BluePippin Pulsed-Field gel, a fraction of 30 to 80 kb (average, 43.5 kb) was selected for construction of a PacBio CLR library following the PacBio manual. The library was sequenced on a PacBio Sequel II instrument resulting in 170 Gb sequence. This corresponds to 226-fold coverage if a genome size of 750 Mb is assumed (Table 1).

PCR-free libraries prepared from a Maculatus XY female and a Guanapo XY male with an ∼300 bp insert length were sequenced with 150 bp paired end reads on an Illumina HiSeq 2000 instrument.

### Read filtering and quality trimming

Illumina short reads were trimmed using Trimmomatic (version 0.36) (Bolger et al. 2014) with the following parameters: ILLUMINACLIP:NexteraPE-PE.fa:2:30:10 SLIDINGWINDOW:6:30 MINLEN:60. Compared with less stringent trimming parameters, these settings reduced the amount of apparent copy number variation between the Maculatus and Guanapo haplotypes significantly.

### Trio binning and genome assembly

Trio binning (Koren et al. 2018) and haplotype assembly were performed on 170.1 Gb of PacBio long-read data using Canu (version 2.2, genomeSize=750 m). The contigs of both haplotypes were compared by dotplots (Supplemental Fig. S2) using D-Genies (Cabanettes and Klopp 2018).

BUSCO (version 4.12, reference “actinopterygii”, revision odb10) and QUAST (Gurevich et al. 2013) were used for quality assessment of the assemblies.

Scaffolding of the assembled contigs to the published chromosome-level assembly (Fraser et al. 2020) was performed by RagTag (version 2.20-r1061) (Alonge et al. 2022). We used 11 Guanapo and nine Maculatus contigs >200 kb, respectively, for a primary assembly of Y_gua_ (28.3 Mb) and Y_mac_ (27.6 Mb) (see Supplemental Table S2; for details, see Supplemental Material). Telomere repeats are found at the start of the first contig Gua_tig00000738 and of the last contig Gua_tig000001309, but not on the corresponding Maculatus contigs or on the published X/Y Chr LR880656.1. Telomere repeats are also present on the unplaced XY scaffold_149F_0, which aligns to Gua_tig000001309 (Supplemental Fig. S3). Alignments for synteny assessment were visualized with AliTV (Ankenbrand et al. 2017).

**Figure 6.**
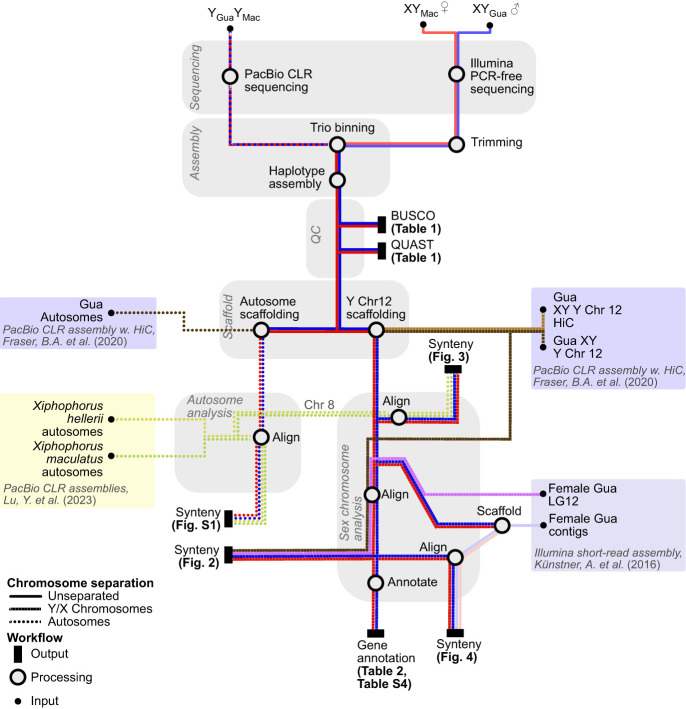
Overview of the data flow and methods for genome assembly and comparisons with published results.

An overview of the data flow and methods for genome assembly and data analysis is shown in Figure 6.

### Genome annotation

Protein coding genes on Y Chromosomes were annotated by synthesizing gene evidence from homologous alignments, transcriptome mapping, and ab initio prediction.

For homologous alignment, we collected 424,637 protein sequences from vertebrate database of Swiss-Prot (https://www.uniprot.org/statistics/Swiss-Prot), RefSeq database (proteins with ID starting with “NP” from “vertebrate_other”), and the NCBI genome annotation of human (GCF_000001405.39_GRCh38), zebrafish (GCF_000002035.6), platyfish (GCF_002775205.1), medaka (GCF_002234675.1), mummichog (GCF_011125445.2), turquoise killifish (GCF_001465895.1), and guppy (GCF_000633615.1). We aligned the protein sequences to the genome assembly using Exonerate (https://github.com/nathanweeks/exonerate) and GeneWise (Birney et al. 2004), respectively, to predict gene location and intron/exon structures. To speed up GeneWise, we used genBlastA to roughly locate the alignment first (She et al. 2009).

To collect transcriptome evidence, we downloaded the RNA-seq data from NCBI's BioProject database (https://www.ncbi.nlm.nih.gov/bioproject/) under accession number PRJNA230881 (tissues include whole embryo, adult gonad, brain, tail, and pooled organs) and mapped them to the assembly using HISAT (Kim et al. 2015). StringTie was then used to interpret the mapping result for gene location and structure. In parallel processing, we assembled transcripts from the mapping result using Trinity (Haas et al. 2013) and aligned them to the genome using Splign (Kapustin et al. 2008).

We used AUGUSTUS for ab initio gene prediction (Stanke et al. 2006). AUGUSTUS was first trained by BUSCO with the parameter “-long” (Simão et al. 2015). We then trained it again using high-quality gene models that are commonly agreed upon by Exonerate, GeneWise, StringTie, and Splign. The retrained AUGUSTUS took the homologous and transcriptome gene models collected above as hints and screened the genome for ab initio gene prediction.

To synthesize all gene evidence into a final consistent set of annotation, we clustered overlapping homology gene models and kept the one best supported by transcriptome evidence (Supplemental Code 1), ƒ. When the terminal exon of the kept gene model was poorly supported by transcriptome evidence, we screened the eliminated gene models for better supported terminal exons and replaced them. For genome regions where no homologous gene was predicted, ab initio gene models were recruited if they were fully supported by transcriptome evidence.

### Sequence alignment and divergence calculation

The sequences of the Y and X (NC_024342.1) Chromosomes were aligned using minimap2 (Li 2016, 2021), and the alignment was improved using Genome Alignment Tools from the Hiller laboratory as indicated below (Supplemental Code 2). Default parameters were used during minimap2 aligning. The alignment blocks were chained up using axtChain. The unaligned regions neighboring those blocks were realigned using patchChain.perl. We used RepeatFiller to incorporate the newly captured alignments into the alignment chain, whereas chainCleaner was used to remove obscure local alignments (Suarez et al. 2017; Osipova et al. 2019). At last, chainNet was used to collect alignment chains hierarchically to capture the orthologous alignments only (Kent et al. 2003). Based on the alignment, the sequence differences were then calculated as the percentage of SNPs and indels in 10 kb sliding windows along the alignment (Supplemental Code 3).

### RAD-tag sequencing and analysis of sex-specific markers

Of four different guppy strains kept in community tanks, 48 males and 48 females per strain were analyzed using the double digest RADSeq method (Poland et al. 2012), essentially as previously described (Kottler et al. 2015). Only 91 individuals of each strain could be successfully genotyped because five of the barcodes failed. The populations originated from the rivers Tranquille (West Trinidad), upper Quare (Quare_II 215-3), lower Oropouche (Oro209), East Trinidad, and Poza Azufre (PV6) Venezuela. These were all descendants of populations previously used for genotyping (Table 1 in Willing et al. 2010). The libraries were sequenced on an Illumina HiSeq 2000 instrument with 100 bp single-end reads. Reads were clustered with *Stacks* (Catchen et al. 2011), first by strain and then by sex. Alignment to male and female Guanapo WGA resulted in 3218 male-only tags (data set 1) (Fig. 5B, red bars).

In a second experiment, RAD-tag libraries were built from genomic DNA of 25 females and 25 males of the Caroni Swamp strain of *P. reticulata* (CS; WLC 3501) and sequenced on the HiSeq 2500 platform. The reads were demultiplexed and then determined present or not in each female and male individual in RADSex (R Core Team 2013; Feron et al. 2021). A tile plot describing the number of reads in the number of female/male individuals was then generated and used to reveal the sex-determination system of the species. Reads present only in males were then aligned to the genome to locate the sex-determination region (data set 2) (Fig. 5B, blue bars).

### PoolSex analysis

Male and female tissue samples pooled from 30 males and 30 females of *P. obscura* were sequenced with Illumina short reads, yielding the PoolSex reads for male and female. The reads were analyzed using the PoolSex pipeline (https://github.com/tankbuild/PoolSex). Specifically, male and female PoolSex reads were first mapped to the reference genome using BWA v0.7.17 with default parameters (Li 2014). Then the mapping coverage and content of sex-specific SNPs were determined in 50 kb sliding windows using PSASS (https://github.com/SexGenomicsToolkit/PSASS).

### TE identification

Transposable elements were identified using RepeatModeler2 (Flynn et al. 2020) and RepeatMasker (https://www.repeatmasker.org/). DNA sequences were first scanned by RepeatModeler for de novo TE family identification. The results, together with models from FishTEDB (Shao et al. 2018; https://www.fishtedb.com), were transferred into RepeatMasker as a query library to further identify and mask TEs from the DNA sequences. The Kimura value of each TE was retrieved from the result file of RepeatMasker. TE density and Kimura distribution were plotted using ggplot2 in R (https://www.R-project.org/).

## Data access

The PacBio CLR sequencing data of the Y_gua_ Y_mac_ male, haplotype assemblies, and Pool-seq data have been submitted to the NCBI BioProject database (https://www.ncbi.nlm.nih.gov/bioproject/) under accession numbers PRJEB75520, PRJNA1111885, PRJNA1111886, and PRJNA1108343. The Illumina short-read data for PCR-free libraries of the Guanapo XY male and the Maculatus XY female have been submitted to the European Nucleotide Archive (ENA; https://www.ebi.ac.uk/ena/browser/home) under accession number PRJEB75519. The gene annotations on each haplotype are available at figshare (https://figshare.com/articles/dataset/Poecilia_reticulata_YY_chromosome_Guanapo_Maculatus_/21637295?file=38357789) and as Supplemental Data.

## Supplemental Material

Supplement 1

Supplement 2

Supplement 3

Supplement 4

Supplement 5

Supplement 6

Supplement 7

Supplement 8

Supplement 9

Supplement 10

Supplement 11

Supplement 12

Supplement 13

Supplement 14
