# Supplementary material for: Identification of the male-specific region on the guppy Y Chromosome from a haplotype-resolved assembly
Source: Genome Res. 2025 Mar;35(3):489–98. doi: 10.1101/gr.279582.124 (PMC11960691; doi:10.1101/gr.279582.124)
Supplement: Supplement 8 [file Supplemental_Code_1.docx]

**Supplemental code 1. Perl script synthesizing gene evidence during genome annotation.**

#!/usr/bin/perl -w

my $usage=<<EOF;

--------------------------------

"ab homo", refers to "ab from homo", or "ab and homo", aim to let ab_initio and homology prediction make up for each other

Usage: $0 clusterred.gff(or stdin) (-ab AUGUSTUS) >ab_homo.gff

-ab assigns the marker to recognize ab initio prediction, could also be "AUGUSTUS,snap,..."

* input gff should be scored by splice_score.pl first

* output about "pre_stop;frameshift" will not always be right anymore, replace them afterword

Du Kang 2020-10-20

--------------------------------

EOF

use List::Util qw(max min);

$ab="AUGUSTUS";

while ($_=shift @ARGV) {

if (/^-ab$/) {

$ab=shift @ARGV;

}else{

push @file, $_;

}

}

die $usage if (!@file and -t STDIN);

$ab=~s/,/|/g;

open IN, "cat @file |cut -f9-|" or die $!;

while (<IN>) {

my @ab;

my @homo;

my @iso=split /_flag_/;

foreach $iso (@iso){

$iso=~s/# alignment.*//;

$iso= &gff_order($iso);

$iso=~/\t($ab)\t/i? push @ab, $iso : push @homo, $iso;

}

if (@homo==0 or @ab==0) {

s/_break_/\n/g and print for @iso;

}else{

# to judge whether to merge the multiple homo together or not

# I only set an easy threshold here: merge them if the sum of the coverage <120 and none of them is perfectly supproted by RNA

my $cover_sum=0;

my $flag=0;

foreach $homo (@homo){

my ($score,$cover,$start,$stop)=$homo=~/\tgene\t\d+\t\d+\t(\S+)\t\S+\t\S+\t\S+cover=(.*?);.*;start_c=(\d);stop_c=(\d);/;

$cover_sum+=$cover;

$flag=1 if $score==100 and $start==1 and $stop==1;

}

if ($cover_sum<=120 and $flag==0) { # merge, @homo==1 is included in this case

foreach $homo (@homo){ # I know this section is hard to understand, but correct

my @tmp;

foreach $ab (@ab){

my $cut= &cut($homo, $ab);

if($cut eq ";"){push @tmp, $ab}elsif($cut eq "-"){$homo=$ab}else{$homo= &paste($homo, $ab, $cut)}

}

@ab=($homo,@tmp);

$result=$homo;

}

$result=~s/_break_/\n/g;

print $result;

}else{ # do not merge, left gets left, right gets right, middle gets nothing

my $cut= &cut($homo[0], $ab[0]);

$cut=~s/;\d+/;/;

$homo[0]= $cut eq ";"? $homo[0] : $cut eq "-"? $ab[0] : &paste($homo[0], $ab[0], $cut);

$cut= &cut($homo[-1], $ab[-1]);

$cut=~s/\d+;/;/;

$homo[-1]= $cut eq ";"? $homo[-1] : $cut eq "-"? $ab[-1] : &paste($homo[-1], $ab[-1], $cut);

s/_break_/\n/g and print for do { my %seen; grep { !$seen{$_}++ } @homo};

}

}

}

############################################### subs ######################################################

sub gff_order {

# I eat in a flatted gff, order then output

my $in=shift @_;

my ($gff,$suffix)=$in=~/(.*?)(#.*)$/;

$gff=~s/_break_/\n/g;

$gff=~s/\tintron\t/\tantron\t/g;

my $o=`echo "$gff" |sort -k4,4n -k3,3Vr`;

$o=~s/\n/_break_/gs;

$o=~s/\tantron\t/\tintron\t/g;

return $o.$suffix;

}

sub exon {

# I eat in a flatted gff line and output exon structure (no sort version, already sorted by &gff_order)

my $i=shift @_;

my @exons=();

for $catch ($i=~/\t(CDS|exon)\t(\d+\t\d+)\t/ig){

next if $catch=~/CDS|exon/i;

$catch=~/(\d+)\t(\d+)/;

my ($s,$e)= $1<$2? ($1,$2) : ($2,$1);

my $exon="$s..$e";

push @exons, $exon;

}

my $exons=join(";", @exons);

return $exons;

}

sub cut {

# I eat in two flatted gff(homo, ab) and determine the cut site for paste

my $homo=shift @_;

my $ab=shift @_;

my $cut;

my ($score_homo,$start_homo,$stop_homo,$S_homo,$E_homo)=$homo=~/\tgene\t\d+\t\d+\t(\S+)\t\S\t\S+\t.*;start_c=(\d);stop_c=(\d);\S+;S=(\d);E=(\d)/;

my ($score_ab,$strand,$start_ab,$stop_ab,$S_ab,$E_ab)=$ab=~/\tgene\t\d+\t\d+\t(\S+)\t(\+|\-)\t\S+\t.*;start_c=(\d+);stop_c=(\d);\S+;S=(\d);E=(\d)/;

my $exons_homo= &exon($homo);

my ($l_homo,$r_homo)=$exons_homo=~/(\d+)\..*\.(\d+)/;

my $exons_ab= &exon($ab);

my ($l_ab,$r_ab)=$exons_ab=~/(\d+)\..*\.(\d+)/;

if (($score_homo==100 and $start_homo==1 and $stop_homo==1) or $exons_homo!~/;/){

$cut=";"; # take the homo

}elsif($score_ab==100 and $start_ab==1 and $stop_ab==1 and $l_ab<=$l_homo and $r_ab>=$r_homo){

$cut="-"; # take the ab

# }elsif($l_ab<=$l_homo and $r_ab>=$r_homo and $S_ab>=$S_homo and $E_ab>=$E_homo and $score_ab>$score_homo){

# $cut="-"; # take the ab

}else{

my $left="";

my $right="";

my $cut_left;

my $cut_right;

$cut_left=1 if $strand eq "+" and ($start_homo<$start_ab or $S_homo<$S_ab or ($start_homo==$start_ab and $S_homo==$S_ab and $l_homo>$l_ab));

$cut_left=1 if $strand eq "-" and ($stop_homo<$stop_ab or $E_homo<$E_ab or ($stop_homo==$stop_ab and $E_homo==$E_ab and $l_homo>$l_ab));

$cut_right=1 if $strand eq "+" and ($stop_homo<$stop_ab or $E_homo<$E_ab or ($stop_homo==$stop_ab and $E_homo=$E_ab and $r_homo<$r_ab));

$cut_right=1 if $strand eq "-" and ($start_homo<$start_ab or $S_homo<$S_ab or ($start_homo==$start_ab and $S_homo==$S_ab and $r_homo<$r_ab));

#check the inner slice sides for the first and the last exon of the homo

if ($cut_left) {

my ($site)=$exons_homo=~/(;\d+\.\.)/;

my $reg=quotemeta($site);

($left)=$site=~/(\d+)/ if $exons_ab=~/$reg/;

}

if ($cut_right) {

my ($site)=$exons_homo=~/.*(\.\.\d+;)/;

my $reg=quotemeta($site);

($right)=$site=~/(\d+)/ if $exons_ab=~/$reg/;

}

$cut="$left;$right";

}

return $cut;

}

sub paste {

# I eat in two flatted gff and the cut site, then paste them and inherit the information

my $homo=shift @_;

my $ab=shift @_;

my $cut=shift @_;

my ($homo_head, $homo_gff, $homo_cds, $homo_pep)=$homo=~/^(.*\tgene\t.*?_break_)(.*)# coding sequence = \[(.*?)\]_break_# protein sequence = \[(.*?)\]/;

my ($ab_head, $ab_gff, $ab_cds, $ab_pep)=$ab=~/^(.*\tgene\t.*?_break_)(.*)# coding sequence = \[(.*?)\]_break_# protein sequence = \[(.*?)\]/;

$homo_pep=~s/\#|\!|\*|X//ig; # remove the symbol of prestop and frameshift if there is any

my ($a, $b)=$cut=~/(.*);(.*)/;

# get the cds length on the left and right for ab and homo prediction

my $homo_left=0;

my $homo_right=0;

my $ab_left=0;

my $ab_right=0;

if ($a) {

my ($catch)=$homo_gff=~/(.*)\tcds\t$a\t/is;

foreach $cds_range ($catch=~/\tcds\t(\d+\t\d+)\t/isg){

$cds_range=~/(\d+)\t(\d+)/;

$homo_left+=$2-$1+1;

}

($catch)=$ab_gff=~/(.*)\tcds\t$a\t/is;

foreach $cds_range ($catch=~/\tcds\t(\d+\t\d+)\t/isg){

$cds_range=~/(\d+)\t(\d+)/;

$ab_left+=$2-$1+1;

}

}

if ($b) {

my ($catch)=$homo_gff=~/\t$b\t(.*)/is;

foreach my $cds_range ($catch=~/\tcds\t(\d+\t\d+)\t/isg){

$cds_range=~/(\d+)\t(\d+)/;

$homo_right+=$2-$1+1;

}

($catch)=$ab_gff=~/\t$b\t(.*)/is;

foreach my $cds_range ($catch=~/\tcds\t(\d+\t\d+)\t/isg){

$cds_range=~/(\d+)\t(\d+)/;

$ab_right+=$2-$1+1;

}

}

my $strand= $homo_head=~/\t\+\t/? "+" : "-";

# nail the cds

my $left= $strand eq "+"? substr($ab_cds, 0, $ab_left) : substr($ab_cds, 0, $ab_right);

my $right= $strand eq "+"? substr($ab_cds, -$ab_right, $ab_right) : substr($ab_cds, -$ab_left, $ab_left);

if ($strand eq "+") {substr($homo_cds, 0, $homo_left)=""} else {substr($homo_cds, 0, $homo_right)=""}

if ($strand eq "+") {substr($homo_cds, -$homo_right, $homo_right)=""} else {substr($homo_cds, -$homo_left, $homo_left)=""}

my $middle=$homo_cds;

my $cds=$left.$middle.$right;

# nail the pep

$left= $strand eq "+"? substr($ab_pep, 0, int($ab_left/3)) : substr($ab_pep, 0, int($ab_right/3));

$right= $strand eq "+"? substr($ab_pep, -int($ab_right/3), int($ab_right/3)) : substr($ab_pep, -int($ab_left/3), int($ab_left/3));

if ($strand eq "+") {substr($homo_pep, 0, int($homo_left/3))=""} else {substr($homo_pep, 0, int($homo_right/3))=""}

if ($strand eq "+") {substr($homo_pep, -int($homo_right/3), int($homo_right/3))=""} else {substr($homo_pep, -int($homo_left/3), int($homo_left/3))=""}

$middle=$homo_pep;

my $pep=$left.$middle.$right;

# nail the gff

$ab_gff=~/(\t\S+\tcds\t$a\b.*\b$b\t.*?[+-])/i;

my $ori= $1? quotemeta $1 : "";

$homo_gff=~/(\t\S+\tcds\t$a\b.*\b$b\t.*?[+-])/i;

my $rep= $1? $1 : "";

$ab_gff=~s/$ori/$rep/ if $ori;

# nail the head

$ab_gff=~/cds\t(\d+)\t.*cds\t\d+\t(\d+)\t/i;

$rep="gene\t$1\t$2\t";

$homo_head=~s/gene\t\d+\t\d+\t/$rep/;

my ($S, $E)=$ab_head=~/(S=\d);(E=\d)/;

my ($start, $stop)=$ab_head=~/(start_c=\d);(stop_c=\d)/;

my $cds_num=($ab_gff=~s/\tcds\t/\tCDS\t/ig);

$homo_head=~s/S=\d/$S/ and $homo_head=~s/start_c=\d/$start/ if ($strand eq "+" and $a) or ($strand eq "-" and $b);

$homo_head=~s/E=\d/$E/ and $homo_head=~s/stop_c=\d/$stop/ if ($strand eq "+" and $b) or ($strand eq "+" and $a);

$homo_head=~s/cds=\d+;/cds=$cds_num;/;

return "$homo_head$ab_gff# coding sequence = [$cds]_break_# protein sequence = [$pep]_break_";

}
