# Supplementary material for: Identification of the male-specific region on the guppy Y Chromosome from a haplotype-resolved assembly
Source: Genome Res. 2025 Mar;35(3):489–98. doi: 10.1101/gr.279582.124 (PMC11960691; doi:10.1101/gr.279582.124)
Supplement: Supplement 9 [file Supplemental_code_2.docx]

**Supplemental code 2. Bash script to align two DNA sequences.**

>>>>>>>>>>>>>>>>>> <<<<<<<<<<<<<<<<<<<<<<<<<<<<<<

#!/bin/bash

function usage(){

echo "

minimap aligning (intra/cross species) followed by chain and net

Usage: $0 (-r) target.fa query.fa (asm5/asm20/map-hifi)

-r raw output of minimap without further chain & net or sort

asm5 for sequence divergence below 5%, good for intra-species alignment

asm20 for divergence not more than 20%, good for cross-species alignment (default)

map-hifi Align PacBio HiFi reads

* target, or so called reference, will be the first line of output.maf, which is also required by multiz

* https://github.com/hillerlab/GenomeAlignmentTools

* https://darencard.net/blog/2019-11-01-whole-genome-alignment-tutorial/

* https://github.com/lh3/minimap2/blob/master/cookbook.md

* last-dotplot --bed1 can be used to mark out contig joint point (Ns)

Du Kang 2021-8-3

"

}

test $1 || { usage; exit; }

asm="asm20"

while [ $1 ]; do

case $1 in

-r) raw=1 ;;

asm5) asm="asm5" ;;

asm20) asm="asm20" ;;

map-hifi) asm="map-hifi" ;;

*) P+=($1) ;;

esac

shift

done

file1=${P[0]}

file2=${P[1]}

p=${P[2]:-"default"}

t=`basename $file1`

q=`basename $file2`

o=$t"_"$q

target=`readlink -f $file1`

query=`readlink -f $file2`

tmp="tmp.minimap.sh.$$"

if [ -n "$raw" ]

then

echo "raw output without further chain&net !!!"

minimap2 $target $query -cx $asm --cs=long |paftools.js view -f maf - >$o.maf

else

mkdir $tmp

cd $tmp

minimap2 $target $query -cx $asm --cs=long |paftools.js view -f maf - |maf-convert psl >minimap.psl

# align - paf to maf to psl

axtChain -linearGap=loose -psl minimap.psl -faQ $target -faT $query minimap.chain &

# psl to chain

faToTwoBit $target target.2bit &

faToTwoBit $query query.2bit &

falen $target >target.size &

falen $query >query.size &

wait

patchChain.perl minimap.chain target.2bit query.2bit target.size query.size -lastzParameters "--format=axt K=1500 L=2500 M=0 T=0 W=5 Q=/home/k_d239/usr/local/GenomeAlignmentTools/example/HoxD55.q"

cat jobList |waitn

cat doPatchChain/*.psl >>minimap.psl

sort -k10,10V -k12,12n minimap.psl -o minimap.psl

axtChain -linearGap=loose -psl minimap.psl -faQ $target -faT $query minimap.chain

# patchChain.perl improve the chains by adding flanked loci aligning

RepeatFiller.py -c minimap.chain -T2 target.2bit -Q2 query.2bit >tmp.chain

mv tmp.chain minimap.chain

# RepeatFiller.py improve the chains by adding TE alignments

chainCleaner minimap.chain target.2bit query.2bit -tSizes=target.size -qSizes=query.size tmp.chain removedSuspects.bed -linearGap=loose

mv tmp.chain minimap.chain

# remove obscure loci alignments

chainPreNet minimap.chain target.size query.size tmp.chain

mv tmp.chain minimap.chain

# remove chains that do not have a chance of being netted

chainNet -rescore minimap.chain target.size query.size target.prenet query.prenet -tNibDir=target.2bit -qNibDir=query.2bit -linearGap=loose

# Hiller's version of netting

netSyntenic target.prenet target.net

# add synteny information

NetFilterNonNested.perl -doUCSCSynFilter -keepSynNetsWithScore 5000 -keepInvNetsWithScore 5000 target.net >target.filter.net

mv target.filter.net target.net

# filter net

netToAxt target.net minimap.chain target.2bit query.2bit out.axt

axtToMaf out.axt target.size query.size minimap.maf

# convert net to maf

flat '^a|^#' minimap.maf |sort -k1,1Vr -k4,4n |flat bak >../$o.maf

# sort according to the reference

cd ../

fi

/bin/rm -rf $tmp

last-dotplot $o.maf $o.maf.gif

# plot
