# Supplementary material for: Identification of the male-specific region on the guppy Y Chromosome from a haplotype-resolved assembly
Source: Genome Res. 2025 Mar;35(3):489–98. doi: 10.1101/gr.279582.124 (PMC11960691; doi:10.1101/gr.279582.124)
Supplement: Supplement 10 [file Supplemental_code_3.docx]

**Supplemental code 3. Perl script to calculate sequence differences from a pairwise DNA sequence alignment.**

#!/usr/bin/perl -w

my $usage=<<EOF;

----------------------------------------------

I calculate sequence difference (p distance) from a .maf file, indel will be count as one change. *The .maf file must contain the final blank line !!!

Usage: $0 input.maf(or stdin) (-r 1) (-w 50000)

-r set which line as the reference/target sequence? default: 1

-w set the window size, default: 50000

demo of output: "subject start end diff(%) SNP+indel align_len marker4retrieve query"

see maf2diff_pair.pl which will use me to calculate all pairwise-diff in multiz.maf (multiple alignment)

Du Kang 2021-2-10

----------------------------------------------

EOF

$win=50000;

$refl=1;

while ($_=shift @ARGV) {

/^-w$/? $win=shift @ARGV:

/^-r$/? $refl=shift @ARGV:

!/^-/? push @file, $_:

die "Unrecognized parameter $_ !!!";

}

die $usage if (!@file and -t STDIN);

open IN, "cat @file |" or die $!;

$sline=0;

while(<IN>){

if (/^s/){

$sline++;

my @F=split;

if ($sline==$refl) {

$ref=$F[1];

$S=$F[2];

$strand=$F[4];

$length=$F[5];

$refseq=$F[-1];

$align_l=length $refseq;

}else{

push @seq, $F[-1];

push @seq, $F[1];

}

}elsif (/^\s*$/ and @seq) {

while ($seq= shift @seq) {

my $qname=shift @seq;

my $seq_left=$refseq;

my $start=$S;

foreach $i (0..$align_l-1) {

my $a=substr($seq_left,$i,1);

my $b=substr($seq,$i,1);

# substr($seq_left,$i,1)= ($b eq "-" and $a ne "-")? "+" : ($b eq "-" and $a eq "-")? "&" : ($b ne "-" and $a ne "-" and $b!~/$a/i)? "S" : $a;

substr($seq_left,$i,1)= ($b eq "-" and $a ne "-")? "+" : ($b eq "-" and $a eq "-")? "&" : $b=~/N/i? "N" : ($a ne "-" and $b!~/$a/i)? "S" : $a;

}

while ($seq_left) {

($seq_cut,$seq_left)=&seqshift($seq_left,$win);

$seq_cut=~s/&//g;

next unless $seq_cut;

my $l=length $seq_cut; # this is alignment length

my $snp=($seq_cut=~s/S/S/g);

my $in=($seq_cut=~s/(\++)/$1/g);

my $del=($seq_cut=~s/(\-+)//g);

my $diff=$snp+$in+$del;

my $per=sprintf "%.2f", $diff*100/$l;

$l=length $seq_cut; # this is sequence length

my $end=$start+$l-1;

my ($s,$e)= $strand eq "+"? ($start,$end) : ($length-$end-1,$length-$start-1);

print "$ref\t$s\t$e\t$per\t$diff\t$l\t$S\t$qname\n";

$start=$end+1;

}

}

$sline=0;

}

}

##################### sub ######################

sub seqshift {

# input: seq, window_size

# output: seq_cut, seq_left

my $seq=shift @_;

my $win=shift @_;

my $l=length $seq;

my $block= $win<$l? $win : $l;

my $seq_cut=substr($seq,0,$block);

my $seq_left=$win<$l? substr($seq,$block) : "";

return ($seq_cut,$seq_left);

}
