# Supplementary material for: Identification of the male-specific region on the guppy Y Chromosome from a haplotype-resolved assembly
Source: Genome Res. 2025 Mar;35(3):489–98. doi: 10.1101/gr.279582.124 (PMC11960691; doi:10.1101/gr.279582.124)
Supplement: Supplement 11 [file Supplemental_dataset_S1.doc]

>79529

TGCAGGGACCTGAAAGCTGACGGCTTCAGGAATCTATGCTGAGGTTGGTCTCAGTCTCTCTTTCATTTATGTTTCATTTCAAACTCAGAGCTGTGA

>22961

TGCAGGTGAAAACACATGAGGAGCTGTACCTTGTAGACATTGATTGGGCCGTCTATGACGATGTATAGTATGTCTCCCATGGCGAGGCTGGCTATC

>216971

TGCAGGCGGCAGAACTTCAGGTGCGTCTTCGCTGATGTTCAACATCCTGCGGCTTCAAGGGCGCCGGCGGCTGCTTCAGCACGCTGAGACAATGGC

>122314

TGCAGCCAGAGGAAGCTTCGCTCAGGTAAACACACCTGAGCAGCAGGAACTGTTTGCTTCCTTTCGAACGCTCAAGTTTCTGCCTGTCAGCCTGAA

>325226

TGCAGCTCTGCTGTGTTCATCTTGATTTATTCTTTTTCAAACTTTCAGTCCTTCTGCTTTGAGTTGAAGCATTGCTTTTCTTGGAGTGTTTTTTCC

>202300

TGCAGTTTTTCTTTGCTTGTTTTTATATCGCTGTGCATGGCCTGCATGAGTTCATGTGTGATTTACTAGCAGGACGGAGGCAGTCATAACGAAAGA

>155086

TGCAGCTGAGAAGCACAGCTCATACTCTGCCATGAAGCAGACCTCTCTTTTTTTTTTTTCCTCAAGTGCTGGACAGTACAAGAAGGAGGAAGCGAG

>89068

TGCAGGATGCTGCGTTCACAGAGTCTGTTCGAATCAGTCGCAGCCCTTGACCTCTGAACTGTTTCATTATTCGCTTCCCTGTGTGAAGCATCAAAG

>318733

TGCAGAAATCTATGATGAGCAGCTCTACGTCACAGCAACGGCGACTCGGAACCTTTTCAAACACCAAGACGATTAAGATCGGAAGAGCGGTTCAGC

>1155

TGCAGGGCTTTGGCTAAATGGCCACGATGGAAGGGAACAAAAAAAACAAAAAAAACAAGAGGAAGCCCAAAGTGAATCACAATGAGAGATCAGACT

>241289

TGCAGTCGCCGGGGCAGCGGCGGAACCCGTCCCACAGCTGCCCCAGCTGGCGAGCCGAGCCTGGGTCGCTAAAGCCAGCGCCTGGGTGGCCGTGGT

>170954

TGCAGCAGCGGCCGCAGCGAGTGCGCGGAGGCGGCGCGGGGCGGCGGCGCGGCGCGGCGGTTAGCGTGCTGGATGGTCAGGCGGCAGCGCAGCACC

>216034

TGCAGCTGACGAGGAACCAGAACCAGCTTTTCAGTAAGACTTCAGTTTCCATCCCACCTGTGAAAGAGACGATGCATCCACGGACACGGTTCCCGT

>85810

TGCAGGCCAAGATCATGGGTGGGTCCAACAGAACCAGAACCTCCCACTTCCTCCATGTTTCTGCTCCCATACATGTAATGGATTAGATTATTTTAG

>109676

TGCAGGAGCTGTTCTCTTTGAAATGAAGACAAGCGGTTTGCACCCTCGTGAACTTTATAAAACAATCCCCCTCCCTTCTGCTGTCACACAGTGGGA

>94784

TGCAGTCAGACGTTTATTGATTGTTTAGTTTGATAGCAGGTTTCCTGCTACTGTAAAACGCCCCCACCAATCAGAATAAGCTCTTCATGGTGTGTG

>222174

TGCAGATCCGTTTCTTTTTCTCTAATGATGGATCTTCATCATCTGGAGGTGATGATGATGATGATGACCCCAGGAGGCCCGGTCCAAACTGGGCAG

>139011

TGCAGCTGAAATATTCAGCTGTTCCAAAGCAGGATGCTGATTTATCCAGCTTGATCAATAAACGATAACCTGGATCAATAACCATCAGGTTATAAC

>24416

TGCAGGTGAACTGGAGACGCTAACCTGCCGTTTCTCTCTGGGTGTTTGCTAATGACAAACTGATGTCAGAGGAAGTTCAGTGAAACCTGAGATCAT

>40602

TGCAGGCAATTTACTCAGCAGGCGGGCTACATGTGATCCACATTTTAGCTCAGCATCACCCTCTGGGCCCAATGTTCTGAGCAACCCGACCAGAGA

>191417

TGCAGTCCTTCGGGTTTTCTTACCGTTCCACAGACAAATGGCTGTCACTGAAACCTCTTTAGTCTCATCTTACTCAGACAAGCTGCCAATGGGTGA

>179407

TGCAGCCAGTTCTTCGGGGAAAAGCGATAACCGTTCTGTACTTCTCTCATTTTCCTTTGCAGTGTTATGTTTTTCATAAACAATGTTGTTTCCTTG

>236993

TGCAGAAAAAGTCACAGAGGTTATCTGACCCCTTTGCGAGAGGTCAAAGGTTTGCTGCTACTGTAGCTGCTGGGAGGTAAACAACAAACCAGGAAG

>94194

TGCAGGGTTAGTCAAAGACTCTGGGAACTCTTTTTTTGTTTTATGTGCATAGTGTGGGAAGACTACAATTTCTCAACACTTCCAGACTTGTCTCAG

>31717

TGCAGGACGACGAAGAGTTTGACGATGACTACGAGAGTCCGTACAGCGACGAAGATGACAGGGACTACGAGTCCCCGAATGATGACCTGGACGCGG

>329493

TGCAGATGGCAGTGACCACAGTGGATATGCAAAGGGAAGACTGCACAGTCTCTGTTTCTGTGCCCGTGTGGAGCAGCGTAAGTCATTAAGATCGGA

>123244

TGCAGCACTTTCCCAGCGGCGTGACGCTCATGAACGCATGCAATCATGCATGCGTTCCTGCTGCACAGAGCCCAGAACGAGCAGAGCAGCAGCAGG

>56234

TGCAGCGACGCCTCCAGCTACTACTCTCAGTTTGGACGCTCACCAGGCTTCACCTCTGTTGCTGGGCGACGCTTCAAAGGGCTGCTGGAGGAACAT

>213603

TGCAGGTCAAGTACAACTTGGTAGCCGTGACTTTCTCCCATCTGTGAATGTACCATAATTGACATGCCATCGCAGAGCTTTCTGCTGCTCTGGTTG

>241297

TGCAGCGTAATGAGAAAAAGCACCTCTGAAAGGCACTAAAACAACTTCAGTCGGAGTGACTCCGGCTGGAACGTTTCTAGGGAAATGCTGTGTTTT

>41808

TGCAGTTTTTATTTCTCGGCTTCCTGCTGGTTTCCTACTGGGGCAGATTCTCTAACTGGTCTGCGTTCCCATTAGCTGAGCTCATCCAACACTCCC

>264988

TGCAGTTTCTAAGCTTCCACCTCTCATGGCTGAGGAGGAGCAGAGTAAATGTTCAGAAAGGTCAGAAAGCATGGGAAGCGGGGGGAGTTGTCCCGT

>156615

TGCAGGAGGAAACGACAGGTGATGGATGGATGGATGGATGGATGGATGGATGGATGGATGGATGGATGGATGGATGGATGGATGGATGGATGGATG

>84992

TGCAGCTCCATCAGGACTTCATGGAGTCTGGGACGGAAACGAATCAGAAGCCTTTCAGTCACGCGGCTGGGCTCGTTATCCCCCAAAGCAGTCTTC

>178160

TGCAGCTTCATTTATTGCTGGTTCTCCGAGTCTCATTATTATGTAGCCTAAAGCACATATAATCCACCATGGACTTTTCTGCATCCTACCAACTGG

>23308

TGCAGCTCCCACAGAGTCAGTGGCCGGAGCATCCAATTATACCATGCACCGACTTCGTTGAGGAGCAGCGTAGAGCAGCCACTTGCTTGGTTGTAA

>155533

TGCAGGTAATCTTCCCATTTACAGCTGTGCCCACAGACAAGTGACAGCAAGTGGGTAATTCAGGTCAACTCTTTCATCGAGTTATATAAAGAGCCC

>57076

TGCAGAGCAACAACAAATCCCTTTTTCTCTTCTCTCGCTGCACAGAGGAGCGTGGTTCATGTGTGTGTGCCGTTTGCAGAAGGGCACGGCTCTTTT

>12408

TGCAGGTCACGACCTCTTCACACCAGCTCCGGGTAAATCTTCCCAGTCAGGTTTCTCAGAATGAGATCAACGAGGTAGAACAGCCTGACTGTGGTT

>34039

TGCAGCTCTATAGAATACGAGACAAATCCACCGAAGAAGAAACACAAACTGAAACCAGACTGTCGCATCAGCTGCACCTGGTATTAGCAGGGTTGG

>19054

TGCAGGTGGATCCATCATGGCGCTGGTGTCGGAGTTCCTGGGTCAGCTGACGTTGTCGTATGGGGGGGTGAGTCTGACGGTCCACTGAGCTCCAGC

>278005

TGCAGAAGGTCCTCCACGCCTTTGTTAGCTCGAGGTTTGATTATTGTAATATTTTATATGTCTGTATCAAGCAGTTTTCCATCTGCTTGTCCAAAC

>119113

TGCAGATCAACATGCAACACAGCACATGCCAACATGCAGTGAATCACACGCAGTTCTGCAAAACAGAGTAAAAAAACAGCTGGAGCAAAAAAACAA

>165191

TGCAGCGCCCCCTTCAGCAGGTGCCACACTCTGCAATCACCAAAAGCCAATTCACTTCACTGAAAAGCTCACGACGATCTGAAGCAACTTTATTTC

>206447

TGCAGGGCAGCAGTAATTGGAATAGACAAGGTCAAGTGCAGCACTCCCAGTTACGAACAGAGCATACTGAGCTTTCTTTCTACCCTAACTATGATG

>148782

TGCAGCAGCAGCAGAGGAACCTCTCCCTACACGAATACATGAGCATAGGCCTGCTGAAAGAAGCCGGCATCTCCGTACCGGCGGGGAAGGGGGGCA

>295385

TGCAGGACATTATGAAAACTTTCCGTCACACACCGCATTTTAGAATTGGCACATTATGAAAATATGATTTCACAGCATATTTAGAGTGAGCTTTAG

>195423

TGCAGACAGAACTGGAGTTGTGGAGGATGACAGAGAGCGTAAGATGGGAATACATCCAGCTTCGGTGGTAGTAGTTCGCCAGTCATTACTACCAGG

>8161

TGCAGCTGAGTGCAGTGCTTTTCTGCCTCAGTGAGTGAATATATTTCTGTGCTTCACCTGTAACTGACTGACGCACCCCGATCCCTGAGGCGCCTC

>110073

TGCAGATGTTGGAAACACTCATGTGAAGTAACAGCAACATCAAAGGGAGGAGGAAGTATATATGTAAATAGGGGGAAGTAGGAGAAGAAAAGAAAG

>213534

TGCAGGAGCATCCAGTCCTTGCAGCCAGACTGTCTTATGGTAGAGGTGGTGTCATGCTGTGGGCTGGGTCATCTCCTCTCTAGTCCTTCATGAACA

>67539

TGCAGGTGAGTCTGAGTGCACACGTGCCTTAGATTACACCGGTTGGAAGCCAATTAGCACACGCCATCAGGTAGAGTAACGCTTGAGGAGATGGCC

>11853

TGCAGCGACCTTCAACCACAGCCTGCCATCACAGCGCCGCCGGGCCGCGGCGGAACCGGCCCGGTCCAGTGAGGAGAGCAAACCCAGCCAGTCTGA

>119534

TGCAGGTTGCGGCGGCGCAACCCCACTGATGCACCTACTGACGCAAACTTATTGGATCAGATGCTACTTGGCCTGCGTGAGGGTCCCCTGTCCAAT

>208210

TGCAGCCAGAGTTTGACAGTATGTCCCAATAAAAGGGAGAGAGCTAGCAGTGTCAGTGCTGACCTTCCTTACACAGACAACATTCATTATGACAAC

>282811

TGCAGGTGACTCGATCTCTGCTGTTATCGGGCCGTCTGAGTTCTGCTGTTGCCATGGTGTTGTCACTGTGCTGAAGGTTTTTAAGATCGGAAGAGC

>50695

TGCAGCCCCATTTGAGTTCACAACAGTTGATCTCTTTGGACCATACCTCGTCAAAGATATCATCAAGAAAAGGGTCTCCACGAAAGTGTGGGGTGT

>318823

TGCAGCAGCCTGGCGTCTCGTATAAAGCCCAAGGTTACTGTGAGTGGCCTTACAGATGTGAATGGATCTGAGCATGTTTTTTCAGCTGGAAGAGCC

>178333

TGCAGCTTTGCTCCACTGTTGCTGCATTTGCAGATGCGCATCAACCAGATGAAGTACTCAGTGTGGGAGCTTTTCAATATTTGAACGCAGGATAAT

>168796

TGCAGAAGTGACTGAAGCAGTTTGACTTTCTGTATAAAACCCAGAAACCTTTGTTCACAGCCTGCTGCGGTTCTGCTGACATGTTGATCATAAACA

>92148

TGCAGCAACCATCAACTGACAACAACAAAAAAGACAATAGGAAGCACAACATAGCTGGCTGAGCGCAGAAAAGTGCGTGCTCATCCCTTACACCTG

>244123

TGCAGATAAACAAAATGGCACCGGCGTGGTCACCCAGCACAGCGAATGCACTGCGGACGGTAAGAATCGGCGTCGTCGCCACTAAAACCAGCGCTT

>316778

TGCAGATAAGCCGGTCTTGGACTAGGACACTCCGGACTCCGTCCACAGCTTTCGTCTTGTGCTAACATTCAGCGCCACATCGAGTCTTAAGATCGG

>8983

TGCAGGCATGTGAAACACAAACACTGATCTGGCTCCAGAAGGACTGACATGGGTCAAGCTGTAAACGACAGCGGCCTCTCATTTCAAACCACTGCT

>211335

TGCAGTGGTGCTTGGAAGGCGGCGAACAGGCACTTTCCATATTTCCGTATAAATTTGACCTGTCCACGTCTCGCGCTCCCTCCGGCTCCGCTAACT

>105642

TGCAGCCCAAATAGCTGCTCAACCCGTTCAGGCGTTTCCCTTTATTCATTTTCCTCTGTTCATACAGAGAATCAAAGGTTTTAGTCTGTGAAAAGA

>9003

TGCAGGCAGCACAAACATGTGGAGGGGAATGCAGCGGCACCATGAACTATGTCATGCCACTCTGGCTACATACTGACCCTGTAAAGGCACCAGAAG

>231136

TGCAGCAATGCGGCCGGCCAGACGGGCGGACGCGCTACGGACAGGCGGCCGGTTAGAGGAGCAGCATCTCGCCCCGTCAAGGCGATCCGAGTCGAG

>323478

TGCAGCTGTCAGTATACAAGGTTCAGTTCGCATAACTGCTAAGAAGTCTGAGAGAAAAGCAGCTACAGCTCTTGGTGTTGTTGTAATTGTGTTTAT

>45881

TGCAGCCCGACAGCGGCAGGGTTCGGTTCTGGTCGGGGTTCAGAGCTGTCACTGCTTCGTTCAGCCTGTAAGTCAACGCTCCGGAGCTGAGCGGCT

>248829

TGCAGTAAAACCAAAAGCTCTACAAAAACAAGAACATTGATAAAAACAAACTCCAATCACAAAAGGGGACGAAACGGCCAAACAAAATGCCAGCTG

>65899

TGCAGAGGAGGAAGACGAAGGCACAGGAGAGGAAGAGGAAAAACAGGAAGAGAGTGAGGAGCAGGAAAGAGAGGCTTCTGAGGAGGAGAGAGCTTC

>202151

TGCAGCAGAGGAGATCAGCAGCTTTATTTCACTTTTGTCCACTAGGATCTTCAGTCCAGTGGTTCTTTGCATCCCTGTTTCTCCTGAAGGTGAGTG

>161615

TGCAGGAAGCTCCTTTTTACTGCCGTTGTTGTTGAACATGGCTATCCTCGACTTGAAAGAGTCATCCTGACAAACAGAAATTCATTCATTCATCAA

>153903

TGCAGAAATGAGAATCCACACAGATCAGCACTGAAACCCAAACGGGCCGATGTGACGATCTGAGTCACTGCACAGCAAAACAAGGGAGGAAAGAAA

>136423

TGCAGGCACTTCATTGCTCACTTTCAGCATGTATCCCAAAGCCCATCCAACAGAAGCTCCTGTTGCCTGTAGAGACACATTTGAGTCTCAGTGAAA

>175110

TGCAGACCTTTTTCTGCATCTGTTGTTGGTTATCCACTTCAGTTAGCAACTTCATTTGTATTAGCTGAATGTTTAGCTGAATGTTTAGCTGAATGT

>78837

TGCAGACTCGAGCATAAAAAGTCCATTTGGACAGTGACTATTGACTCGCCATTGGGAGGCTGGCTAATAGCAGTGAAAGAACATGTTTTTGTGCAG

>275480

TGCAGCACCAGGCCAGTGTTCCTCCACCTGATTCCCCATCAATAATCTCTCTATATTTCACCATTTCTATCTCTCTTCCCTCTTCCTGCATCATAT

>76944

TGCAGAATAATTCAAATGCAATGGACCAGGGACACCGTTACAAATCTTTGCCACACGCGAGCAGACGCCAGCCCCTTCACACTTGCAGTTTGTCAG

>148454

TGCAGGTGGCAGGAATGAAGGACACCTGTGGCCAAAAGCATTAGCCTTTGTGTAGTCCATGCCTAATGTTGTGTGCATGCATAAAGAGAGAACTGC

>290090

TGCAGCATATGAAAGCTACAGAATAAAATCCTTTCAGCGCAAACCCCACATCCAGGCTGACAGGGAGCTGGCCACTCTTATAAAGTGGGAATACAG

>94946

TGCAGCTCCAGCGCAGCCTGGGAACCTCCCACACTTTGCATGTCGTTATTCGTTTCTGTGGCTTTTTGACAAAAACCTATAAACACCAAAATAATT

>326688

TGCAGACATTCGTACACCTTTGCAATGAATGAAAAACAATTTGAGTTAGAAAAAAGGAAGGAGATTTCCTATTTCTATCCCGTCTCTCTACATCGA

>227736

TGCAGAACAGTAAATTTGCAGGATACTCCCCATCAGCCATTAGGCGTGAGGAACATTACGCTGTGAGATGGATGGCAGACAGGCCCACAAATGCAC

>248462

TGCAGATCCACCCGAGCAGCTGTTCACCACTGCTGCTCATATCTGGCTGTGAGAGACGCATTCATGTAGCGTCACTGTGGGGCTGGACGTCTGACT

>205011

TGCAGGTGAACGGGATTTCAGTGTGTTGGGTGAGAGCAGCAGGGTCCACAAAGAAACATTTCTTTCCCCCAGAGTGTTGCTGAGAGCTTGGTAAAA

>135310

TGCAGCCCATGAGAAATGCCAGTTTTAGCTGGCTGAAAAGTCGCATTCACTGTGCTGATCTTCATGATGGCTTTGGGCTCCCTGGCCTGGCGGGAG

>307721

TGCAGCCAGTAGAGTTCAAGGCTTTTATTGTTTATTTAGAAAAAACTGGAAGTGAATAGGCATTTGTTGCTGCTTTCTGGATACATGAGCTGCTGT

>191639

TGCAGCAACACACCTGGGGTCAGGGTGGGGTCACGCCGCCTGGTAACCATAGCAACCAGGACGGGAACTCACCTGAGCCTTTTTACTCTTGTTTAA

>166693

TGCAGGCTGGACCTGTTCCAGGTCAGGGAACCAATAAAGCGGCTCTGCTAAAGCTGTACGGCTGTGGAGGCCATGCCAGGATATGGCTCACTGCGC

>197007

TGCAGTCACAGGGCTGCCAGGAAAATGGGATTATCTGCCATAAATGCCAGCAAATGGGAGATGAGTATTTGTAGTCACAGACTTCAATGGAAAAAT

>180314

TGCAGTGACATTTGAACTCCTTTATATGTAGATACTGTTTTAGAAGCAGGAACGTAAGTGGAGACTGTCTGCTGCCATAGAAGCGTGAAAAACCCA

>61598

TGCAGACGCACAGCCCCAGGAAACGCACTGCGGCCGAGTGTGTCCTCTCCCTGCGCCAGCTCGGTTCCCAGGAGTCGGAGCACTGGCTCGACCTCA

>218857

TGCAGGCAGTTATCCATGAAAACACGACCAACGCAGATCTCTGACTTCAGCTCTGCCAACTTGTGCTGCACCGTCTGAACATGACAGAGAACTTGC

>43570

TGCAGACACACAGAGAGTCAACAGGTTTTCTCCAAAACACGAGAATTCATCAACAAAATCATTCAACCTTCGTCACAATAATTCGTGTTTCTCCAT

>24093

TGCAGTGATCACAGCGAAGCGTCTGAAAATGGTTTCTGCTGGCCTTGGTGACGGTCCGAGTTATTTTCAGGTTTTTCTGTGCGTGTGCGTCTCTGG

>22273

TGCAGTCATTCCTCTGGTTCTGTTCGACCCGATTCACCACCAGCTGATGAGCCTCTGTGACCCAGAAGGAACCACAACTGAAGAAATTGATTTGAT

>94805

TGCAGGTGGAGATTTACATAATTATGGTGCACAGCACAAGCCCCAAAAGGCACATTATTTTCTATGGCAAGAAGAGCCCCAAAGAAAAACACGGCA

>316487

TGCAGCCGAGCCAAACTGCACTCAGCCAATCAGAGCGGGAGGAGGCTCAGCCAATCAGAGCGGGAGGAGGCTCAGCCAATCAGAGCGGGAGGAGGC

>32671

TGCAGCAGTTTCAGGTACTCAAACCGCTGGTGCTGGGCTGTGAACTTTCCACGTGCTCCACTTGTTCCACATGCTCCAATGTTGGCTCCACCGCTG

>27245

TGCAGTTTGGCTCACACAGCAAAGACCTGGATGACCTCTTACCAAATCAAACCAAAACTGAGCTATTTTGTCTACAAACATACTTTGACTGGGCCA

>4994

TGCAGACGTGGTCCAGTCTCTTCCATCAGATCAGATAATGGCACAAATATTGTGGGAGCCAATGGAGAGCTAAAAGAAAGTTTAGCTGTCTTGAAC

>167779

TGCAGGAAGAAAGTCATGACTTCTTGGCTCTCGATGTTTTGGGGCTGCTCCTGGGCAATCGTAATGGACGTGTAAAGGGGGGAATATTCTGAAATG

>3335

TGCAGCTCCGTCTCCTAGGAAACGGGCCTGGAGTCGCCTCCATGTTGGATGGCGTCTCACTTTACCCACAATCCTCTTTCCAGTCATCTGCAAACA

>105165

TGCAGAGGTGTCACTTCACACCTCTGCGGCCTAGCCCGGACTTATAAACCAGTGGAGGAGAGCGGAGCAGCACTCACAGCTTCCCAGTTCTTCCAG

>312289

TGCAGATGTGACCACCCTGCTAGTCCAGCATCAGATCAGTGCACCCAGAGGGGTGGGGTCCCCTCAAGATCTCTGAATTCCTGTCATCTTGTGTTG

>219549

TGCAGGAAAACCGAGTTCACAGCAACAGGAAGAGGAAACAAATGTAGCAAAAGAGACGAGACGTTCATGTTCCACCGCCGCATCGCAGGACAGGAA

>275309

TGCAGGAGGCCTTCCCAGGTATGTGTGGCCAATGCTTCGCTGAGAAATTCCCTCAGAAAAGCCCGGTACAACTGTTACTTTAAGATCGGAAGAGCG

>676

TGCAGCGCTGCTGCTCTGACTGATTTACAGGAACAAACACTTGAGAAAGGGCAGGAAAACAGTTTCATCAGATCTGGGCTTAGTGGAGGAATGTTC

>119764

TGCAGCCACTTCCTGTCTGTGGTGTTGATCCAATAAGACGGTTCCGAGCTCAATTCTGATCCGCTGTCCTCCATCTGTCTCCGTCTGTCTCTGTGG

>75914

TGCAGAGGACAAGAGTGATGAAGAAGAGGCTGCTGCTGAGGAAGAGGAGGGAGAGAAGGAGGCCACGACAGAAGAGGTGAGGAAGAACTGCGTTCA

>118124

TGCAGCAGAGCCGTCTCTGTACTGGACAGGCCAGAAAATTAGTTGCGTTTCTACACAAAGACACTCTGAAGCCTAATGAACTGATATGGATCTGAA

>164764

TGCAGTAACAAGATAGCTCCCACAGAGCCTGCTCGGTAACCTATACAACTAAAGCTGGCAACTCAGTCATTAGAAATAAAAAAAGAGAAATATAAA

>22600

TGCAGATGTGATGCCCCAGCCATCAGCCATTCATCTATTTTACCGCCACTAAAATCCATACAGGGACATCAGTAAAGCAGCGATGACAAATGACCG

>170105

TGCAGGAGGATCAGGAGTTGAGGGGTTTTGTGCTCAGGTGTGCTTCCTGCTGGGCGGGGTGGGGGTAAAAAGCGCGAGCCCAGGTGAGCCAGCCTC

>138752

TGCAGGAACAAGGCAACTCTGACGAACTCCATCAGCAAACTGGAGAGGAAGATCAACGAGCTGAGCGACCAGCTGGAGGAGGAAACCCGCATCGCT

>148293

TGCAGATGGTGAACGTGACGCTGCGAGTTCTGTCCCGGCCGCTGGCCTCCAACCTGCCGGTTCTGTACCAGCGTTTGGGTCAGGACTACGATGAGC

>266133

TGCAGGACCTGGGCCGGGTCCAGCCTCTCCAGACAGACGGTGCTTGGGGACGTCTGACCGCTCAGCACTCCTATGGAGAGACAAACACAAAACGAA

>269200

TGCAGATGATCAATATGGACTTCCAGATTGAATTGACCCTAATGGTCCTCCATGTGTGCAGGACGTGTAAATATTCATTTGACAAATGAGCCTCGT

>27846

TGCAGTTCTTGGCTGAACTCTCCGTCACAGTCATGATGATGACGTCAGTTCCTCCATGGCGGTGAACTGCATTCCCTTTGCTGCCATCTAGTGGAC

>298414

TGCAGATGCTCCGGTTTCACCTCTGTAACGACATCGATCCCATCGGCCCACAGGTTGACACAGCTGATCTGCTCTTAAGATCGGAAGAGCGGTTCA

>131290

TGCAGAAACTCAGCCAGTGGCGGCGACAACACAGTGGAGAGGAGACGGTCATAGAGAATGTACTGGACTTTCCTGGGAACGCTGTAACCAAGGAGG

>77071

TGCAGACAAACTGTGCGGCCAGTGGCGCCTCAATCAGCCAGCTGAAGTTGGGGACACCAAGATTCTACCGGCCGCCACTACAAATGATCTCATTAT

>43632

TGCAGCAGCAAAAGGCCTCTAGGGGTCGCAGCAAAGTTCAGCAGACTGCCCAACATCGTTTCCTCCCAGACGAGCCTGTTCCACATCCACAAAAAA

>157965

TGCAGAGGCTTCCTGCTCACACATGTCCACACACCAGGTCTTGAGCAGAAACACTGTTCACTCTTTCGCTAGCAGCTTTTGTTACTGCTCTTCTGC

>214304

TGCAGGCAGGGAGCGTCTGCGTGTGTTAGAGTCCACTTGGGTTTCAGACAACCAGGAGAACATGTGTGCTCCTTCATCATGATCATTTTACAATGA

>289580

TGCAGAGGAGAGCGATGTGATGCGAGACAGGCTCCATGTGTGGGAGGGAAACCAACATGGAAGACATAAACACACTCTGGTCCCTTTAAGATCGGA

>24129

TGCAGGCAGCAGGTAAAGTTCAGGGCTGAATAATTGATGGATCGTTGGAGGGTCACAGGGCGGCGGGAGGAAGAGGAGGAGGAGGAAGGCAGAGAT

>60877

TGCAGTTTCACAGGGAAACTAGAACCCCCTGAACTGGGGAGCCAAAGTTCAGGAGGGGTGTTTTTGGGCTCACGGACAGGTGGATTCTCTGATTTG

>313062

TGCAGACCAGGGAGCGGGACGACCCTGCTTTGGGCGAACAAACTTAGGTTTCAGTCAGACCAGAGATTCGCAGAGTGCTTTAAGATCGGAAGAGCG

>85800

TGCAGCGCCTTTTGTTGCATGTTTCAGCCTTAGAGCTGGTGCTTGCAGGAATGAGGTCACAGGTAGCTATGCGTTGTAGACAGATAGACAGACACA

>306734

TGCAGCTGCTCCGGGTTTTATGGCTTCTGGTTCTGACGGGTCGGCCGTGACCGGGTCTGTTAGCCTGCCCACAGCCCGGTTCTGCTGGGCGCAGCC

>163379

TGCAGTGGAAGATTTCTGACGTCGGTGTGTTTTTTCTTCACGTTGATTTTTCAGGGGACGCCGTCAGAGGAACTCCAGTTAGCAGATTAGCGGCGC

>286574

TGCAGTGCAAGGTCTTAGGCCATAGTCAAAATGTCAAAGAATCAAGTTGAGCAATTACTTTGGTGGTCCTTTTTGGGAATAAATAATTCTGTGTTT

>308076

TGCAGGCATATTTGAGTAGCAGTTGCCCCCATTTTGTAACAATGGAGTACATGTGAGGTATAAAAGCCATAAGGGAGCTGCGGCCACAAAAGTTCT

>11542

TGCAGCAGACTGAGGAAATGTTTCCAACCTCCAGCAGGAAGATCGTTTCCTCATGTGAGTCTCCCAAACTCTCATCCCGAGCAGCACAAACAGGAT

>27687

TGCAGGTGGAGGAGGCCACGGTGGTTCTGGAGGACGACGGGCCGGCCGAGCCGCTGGCGGCGCCTCACCAGAGGCTCCTGTCGGCCTGGAGCGTCT

>116679

TGCAGACCTCATTATCCTGTAAGCAAACTGACCGTGTCGGAGTCCTACGATACCTACATCAGCAGGAGCTTCCAAGTGACCAGAGAGATTCTGACA

>134955

TGCAGCAGGTTTCCCCATGAGGGAATGAAACTGGGACACAGTCCTGTTACATACCTTGACTTATCCTATAACACACAGAGACAAAAACGTCTTATA

>79417

TGCAGTAATCGTGGGAGGTGTGTGAACGGCAAGTGTGTATGCAACCCTGGTTCCACAGGTCCGAGTTGTTCTGAAGAATCTTGTCCTAAAAACTGC

>116035

TGCAGCGCCGACACGACGGCGTACAGGGAGGAGAAATTCTTCCTCGCTCTGCATTCCTGTTCAAAGACACAGAGCAGGACTGCTGAATAAAGGCTT

>214075

TGCAGCCGCTTTGACAGTGGTGGGGTAGTTTTGACGATGTTTCTTTGAGACTTTGGTAGGTGATGTAAAACTGTTTGGCTTCTCATGTTTCTTGCA

>50819

TGCAGTTTTATCACTACAATCTCTTCTGCTGAGAAATCCAGTGACTCCAGTTATGGCTAACAGTGACCTGATGAGGAGCATTTGTGTTTCTGAGGT

>205897

TGCAGCCGCACTGAGCTGTATTGAGCGGGCAAATCAAGGACACTCACTCAAAAGCTTCTGTCAACTTCCTGTATGAAAAGCATCACGCAAGCAGTT

>238757

TGCAGTTCTACTCTGGATTACCAATAATGACTGCCGTCCTTATCTGAGCAGCGAAGTAAATACAATCGAACATTTTTCTCCTAGTTTCTTGTTTTC

>144228

TGCAGGAGTCTTCTCCTTCTCCTCTTCCTCTCTGAGCGAGGGACGATGTCCCCTCCGTCCATCATGTCTCCGCTCTTCGTTGACGCGTCCAGGACC

>3993

TGCAGCTGAGCAGAACACAAGGAGCTACTGCTCCTCCCCCACCAACACCAAGCACCCCCGACACATCATATATAGCAACAGCACAAGGCAGAGGCT

>38

TGCAGGAGTTCAGGTCGTAATACCGGGGCGAGCGCATATATGCTCGGTTTGGAAACCGGAGCGGTAAATCCAAACAGAAGAGAGGGGGAGGGGAGG

>200776

TGCAGAGCAGCAGAACTTCATCGCTACCAGCTTGTCTAAACAGACGTGTTCTGGGCGTGCTAACCGGCTCGTTTTCACTTCTTCTTTCTACCCGAC

>833

TGCAGTCATCTCACTGTGGGCCTCATATGTTACGGGTTTGAAACAGCGCCATCAGGTGATTGGTCCTCATCAGCCACCATCTCACTGTCATGTCTT

>161211

TGCAGGGGAGGCAGGCGGCCCACACAGCGCAGGAAACCGCTGGAAACCAGACAGACCGACCAATCAGCATCCAGGAAACTCAGCCTACATGAACAG

>45445

TGCAGCTCACGGCATTCCAACAGGGAGACACTCAGGCTGAGTGGAGTGGCTCCACTCCTAAACACAACCAGCCGCTCTGACACTCGCTGTGGATCC

>163765

TGCAGCTGCAAATGAGGTCAAAGGTAAAAGGTCATTCTACAAAGAAGCACCACAGAGGGACTGAGCACAATCTCAACAACTATGTGTAATTTTCCT

>188116

TGCAGCTCTTACACACAGCTCAGGGATTCCACAGGAGTTTCTATTCAGATGACTGGGACTCCTAGTGGCAAAATGGGAGCATTGCAGCATGAATTA

>209030

TGCAGCGCGGCATGGAGCTCACCTGGAGGGAGTTCAGCGTTTCCCACAATGCAACGCTGAAGGACTTCATCAGCAGGAACGAGTCCCGCCTCAACA

>20845

TGCAGTCGTGAAAGGAAATCCCCCTGTGATCCCTCCCATTGTTACTTCATTCAAACGCCGCCGCATTCTTCTTCACTGCTGCTGAACAAACAGAAG

>141648

TGCAGATGTTTATGGAGTCTGAAGGCGTCTCGCTTGTTCCCAGTTTGGTTTCTGAACATCTTCAGCTTCAGTGATACCGGTCCATTCCCCTTCCAG

>3833

TGCAGCACAGTAGGAAGTCAGTTTGGTTACTCTGCATAAATGAGAATGAGGAACCTAAATAGTAAATATTCACGTCTTCTCATGTGGGAGAGGCTG

>305020

TGCAGAGCTAACTGTGAAAACACGAACTGGACCAGAGACTAAGCTCACCAACAGCTACCGGAGGGAGGAGTCCGACGGACGCCGGTCTCTTTAAGA

>317183

TGCAGTGGAGGGCCGGGGAGAAGTTGTGAGGGTCCTGCACCGTGTTCCTCTGGGTGGGGTCTCTGGTCTCTGGCCTACGAGCTGAGACCAGACCAA

>109661

TGCAGCGTTCTACTGAAGACCATCTCAAGATGCCACACGGAACCTTCTGGCTTTCCATAATTATAGCCACACTCGAGCAAGACATGCATAAGCACA

>53243

TGCAGCAGCAGCAGCCGGAAAACTTCAGCCAAACTTCACCGTTTTCAGCCGTATTCCGGTTTTTGTCTCGGAATAAACGGACTGACAGAAACGTCA

>122247

TGCAGTGAAGCGCATGTGCACAGCAGGAAATCAACTAGCTTAGCGAGCTAATGCGGCTGGCTAGCAGCTGTTATCAACAATTAGCTTGCCGGCTAC

>91623

TGCAGTGGCGCCTCCGGCCAGCAGGTGGAGCCGTCAGAATGAAGTTTCTCTGACCTGCTGTCCGGCCTGCGACTCCCCCATGATGAAACGGTCTCC

>259922

TGCAGATCCATCACACCTCTGATAAACTCTCCCCAACACTAGGAGATAAAGTCATTTGCAAGGCAGGAGTGAGCCTGCAACAAAATGAATCCAAAA

>25062

TGCAGACGGTCAGCGGGGGTCAGAACCAAGACCGACAACAGGCGGAAAGACCGGGAGAGGAAGAGGAGGAGGAAGAAAACTAAACCAACCACGTTG

>219087

TGCAGAGGAAGTAGCCAAAGTGGCTGAGGCAGTGGAAGAGGCTGCCCAGGCTGTGGCCGAACCTGCTGCTGTCGGCGCAGAGGAGACGCAAAGCGA

>65490

TGCAGTCATACCAACGTCCACCAGAGGGAGCTGTCTGCACGGAGCGCCATCAAGGTGAAAGTAATAAGAAAAGGTTTCTAATGGTGTGTGGACAGC

>89209

TGCAGTGACCAATGGGTCTTGCTGCATCACCTACTTTAGAAGAGTGGTGAATGTAGCAGCCAAAACATTTGATTTAGAAAAGTGAAAATGCAGAAA

>202054

TGCAGGCCTCTTCCTGTGGCACGTACGCACATACTTTCCCATTCCCGTCTCATCCACTTCCCAAAACCCCCCCAAGGATCTCTAGCTTTCTCCTCC

>142035

TGCAGGGATGAACTTCAACATCTCTGCAAAGTGTGAACTGGACTGACTGGATGAAGAAAATTCACAGATGGGTTGCACATCTGGTTTTCCATCTGT

>126334

TGCAGTGTGTGATAATGAAGTCGTGGTTCAGCAACTGGTGAAGCTGCTGAGTTCAGAAGGAGATGCCATGAACACTAAGGTAGGTCCCATTACATT

>65460

TGCAGGCCTTGACTCTATGGCGAGGCCTGCCAATCTAAATAGAATGAGCATTTAGATTGGCACCACCTATTGGTCGGCTCCATTCTGTGCAGTCAT

>100286

TGCAGACAAGAGGAGCAGGATCTGGAGAGGAGATTTGAGCTTCTCACCAGAGAACTACGAGTTATGATGGCTATAGAAGGTCAGTGTTTGGTTGAA

>102456

TGCAGCCATGGCGACCATGAGAAACCTCTGAATGCTCTAAACGATTGTTCGGTGTCTGCTCTGAAACTGATTATCGGCTAAATATGATCGGCAGTG

>229313

TGCAGCTGCTGTTTCCTCCAGAACCTCACAGGAACCTTCATTGTTGTTTGCTGGTCCATCCATCCTGAAGCGGGCCCTTCCTGTTCAGAACCGGGT

>116723

TGCAGCGTTCTGGCCAACCTGCTGCTGCTGTTTCCAATGGGAGAAATCACCTACATCCAGCAGGACCGACTCGCCAGCTACATCTGGTACTTTGGT

>46749

TGCAGCTTAGTGTTGGATATTTTCCCCGGCGCAGTAAGTCCCGAGTGTGTTCCTCATTAGACCACCTTGAAACCATGTAAGTGCGCTGCGACAGAC

>3793

TGCAGCCTGGGCCGCTCCGTCGACCTGGAGAAGGACGACCACCAGCGGGTGCTCTGCAACAACGAGCTCTGCCCGTACGGCAACTGGATGCACCTG

>191427

TGCAGCTGTGAGGCTGGAGCGACTCGTGTTTTCTCACATCAGCCGTCTCAGAGTGAACCGGGTCATCATCCGCTGGAGAGCAGCCGAAGATTTAAG

>99215

TGCAGGTTTTAGAGCAGACCTGAGGTCCATATTCTCTATATAGCTGCCATGCAAATAAATCGTAGGAAAACCTTTTTGGCTCTTCTGGCAGCTTGT

>138662

TGCAGAACCGCCCATTTTAGCTTTCAAACACCAAATATTCCACTTTATGATGGGACGTTGTAAGCGCTAGCCGTCTCAGCGCTACGGCAGAAGCTA

>43861

TGCAGGCCGGCCTGCGGGCTTTTCTTGCCCACACATATGCAGTGAAATCCCCTATAATCAAGCAGCTGCCGGGTCTGCTTTGAAGAGATTTGGAGC

>183535

TGCAGCTCCTCCAGAGGAACTGATCAACCCTCTCTTTGCCTGGACGCTCTGTTTGCCATTGTCTTTCCATCTTTGTATAATGGACTAAATTGGTGG

>333312

TGCAGCGTCCCGAGACGCGCCGCGTTGGACGCAGTTCTTCCTTCCACCAAACCGTCAGCTGGTATTTTTCTCGTCTCCTGCGTTAAGATCGGAAGA

>253501

TGCAGCCTTCTTCCACACTTGCTAGTGTCAGAGTTTGTTACAGCAATAAAAACCTTTCAAAACATAAATCCAGATGTTCTACAAATCTTTATCAGC

>291538

TGCAGCCTCAAGCGAACCTGCATCCATTAGTCTTCAATGGCGTTTGCATCTCTTTGTATTTTGGATGACCTGCTTTATAAACCCAGTACAAAATGA

>46256

TGCAGAGTCAAAAATCCGACAGCTGGTTGAGCTTTCATGAAAAGGCAACTCGTCTTTTCTAGGGACCAGAATCCATCCGCCTGTTGGCGCGGTTCT

>180518

TGCAGAGTCTCATAGTTGGGTGTGTGCTTCCTCCTCAGGCTTCTTCATGTCAAAGTCAGTTTGCACTGACATGAATGTGACAGAGCAGACAGAAGG

>29275

TGCAGTGATGTAATTAGTGTAGGTTTGGTGATGAACTTGTTATCTGCCTCTGTATTAGCCTGTCCCCTGGTAGGCGTGGTCCCTCAACTTTCTGTT

>165706

TGCAGTCATTGGCAGAGACACCCAGAGATGACAGTTTATGAAGGAACATTCAGTTGGACACAATGAGCCAATCAAGGTTGCCAAACTGACCAACTG

>300106

TGCAGACAGCGTGCACTCACAGCTTGGCTGCTTTTCCAAAAACACCCCCCTACCCCATCCACCTCTCCAAAACTCCCTTTTTCGGCCCCTCTTAGT

>75309

TGCAGAGGAAGTGATGTCGTCTCACAGGAAACTCTTCTCTTCCTCTCTTCTTTCAGTCCATCTTCTCCGTGAGACAATTCAACTTGACAGAGTTTC

>143719

TGCAGTACATTCAGCCTTCCTTCTACCTGCTGAACTTGTCTTTGTGCCAGGATGATCCATCAGTAGTAGGACAAAATGTGCCAGTGGTGGTGAATT

>116901

TGCAGTCCGCTGAGCAGATTGCTTTGTTTCATATCAAATTGTCACATCACAAACACACAACGGAACAAAAACAATGCATTGCAGTGATTTGTTCTC

>297541

TGCAGGCACAGCGGCTCTCTTTCCAGGACTGGAGGGACGAAGGCCGGCGGTTTCATCTGGGCGTTCGCCGGGTATTCTCGGCGTTGGGTCAGACTT

>84277

TGCAGAGCAAGAAGCAGGTCTGTATGTAAGGAGGACACAACCGTCCATCACAGCTGTGCAGCAGCGCAGCAGCTGTGACGGGGAGGGGTGAGAGGG

>41546

TGCAGAAGATAGGTGTGGGAGAAACATAACCGGTGGCACCGAGAGGGAGAGAGAGAAACTAAGTGGGTGGAAGTGATTGAACATCGCTTTGTGTAA

>312431

TGCAGGCCAGCCTGTGGAACACGTTGCCACGGTAACGAGCATCACTTCCTCAAACAAAGCCGGCCAGATTTATTTCTGGCTGGTTTCCTGTGGAGA

>228398

TGCAGCTCCAGAAGGTTCTGGGCTTCACCGCCTCATCCGCCCGACAATCAGCTTCCTGGGCCGGCAGAGGAGCGGCGTCCAGGTGAAGGAGGGCAA

>205782

TGCAGAGCTTTCACAGTCACCGCCATCGCCCAGATGTGCATACTGGGACTGATGTGGGTGTTTGGAGCCTTCTTGTTCAGCGAGGGCATGATGGCG

>88875

TGCAGCTTGGATTTCAATCTTGAGTCCAGATGAAGGGATGTGAACATCTGTATCTATGCTGCTGCTTCCATCAGGACGTGAACTGTTGTTCTCTGA

>54219

TGCAGCCTGGCCTCCCAGCTGGCCCTCAGGTGATGGTTCTGGTCCGGACCGGTCCAACCCACTTTCAGGCTGTCTACAGGGTTCTGGAGGTTCTTC

>316009

TGCAGGAAGTGCTGCTGTTCTACTTCCTGTCGTCTTCCTCTCTGTGTGTCTGTGGATCCGGTGAGCGGTTCTGATCCATTAAGATCGGAAGAGCGG

>116184

TGCAGGTGACGGTTGACTCTGCCTCATGTACGTCCTCCCCATACAGAGTGATGAGTCTCGGATGAACCCTAAACACAAACAGCGATTACAATGCAT

>46541

TGCAGTACCTACATTAGTCCTCTAGATGGAGACTCACACTATCAGGAGACAATCCACTCCCTTCTGGATACATTTCCCCATTTTTCTGATTTGAAA

>159539

TGCAGGGGAAAGAGACAGCAGAGGGTCAGAGTTCCTCTGGAGGGGTGATCACATCATCAGCACCCTCCTCATCGTCACCTTCAGCTTGGTGCTGTC

>92297

TGCAGCGCCTCAATCTGAGGATCAAACAGCTTAGCAGAATCAGATTGAGGCGCCGGCAGCGGCAGCTAGCCAGCTAACGCTTCAGCATCAAATCCT

>91423

TGCAGAAAATCACTGGAGCCCGACTGAGTCTGAGGACATGAACACGGAGACGGAGGGACACGTCCACAGGCCGGGCCCGAAACCAGGAATCACCTG

>195870

TGCAGTGTTGAGGCAACAGGACTTCATTTCCAACAGTCTTTATTCCTGGTTTAGTGTTTACTAGGTGATTTAGTGAACCTTACATAATGGCTTGTT

>90915

TGCAGATAACAGGTGAAAGTCAATGATGTAAGAGGCTACACTCTTACCCCCCTGTTTCAAGGATAGTAGCCCCCTAGCTGCCTCCCTCTCAGGGAG

>20470

TGCAGGCATGGCCACCCAGCCCTCCCAGATCAGCAACGGAGGAGTCGGCCTGAGACACAACGTCGTCCTGCCAGTGGGCCTGCTGGTCCTGCTGGC

>277472

TGCAGTGCTAACAAGAGAGGGATATTCTTTTGAACATCGAATGTGAACGTATCCATAGAGACGTAAGATGCACATGTGCCACTTCTACAGAATAGC

>20719

TGCAGGAGGATGAACGAGTCCCTGAGGCGCCAGGAGAATCAAACCGATTCACAGCACAAAGATCACAGAAAAACGTGACGGTAAGTGTCTTTAGTT

>125797

TGCAGTAGGCAGATGCTCAGCTACCATGTGATAAAATGTGTGGTCTCCAGCAGCTGAATGGAAGCGTAGAGTAATTGATGCTCATAAAAGAACAAT

>117292

TGCAGTGTCCAAACTCTGCCAGCATGACGCCGAGGATGATGCAGACCAATCAAACCGTGAAATGAAATCCTGGATTCGTTTGTTTCCTGTGAGAGG

>71864

TGCAGGACCCGGCCGGCAGTCGCAGTCCCATCCAGAAGCTACCGGACAGAACCGGAGCCAGCCGGGCCAACAGCTGGTGGCTGCTGTGAGCCTGCA

>4200

TGCAGGTGGTCAGAGGGTCCTCTGGGTCTCCCCCCACACAGGCAGCACGGCGGGAGCAACGAGACTCACCATCAGCGGAGACGGTAGGAGGAGGAG

>335378

TGCAGCAGCAGAAGAGACCAGACTTCAGATCAGCTAATCACTACTCTGAGCCAAGCTGTGCAGTTCGGTCATGAATATCGAGCGGCGTTTAGTTGC

>64761

TGCAGCAGCGCATTCAACCTGCTGCTTCAGTTTGTCTGATGACATCCGTGGCTTTGGTGTAATATATGCAAATTTTACTGCTTTAGCTTTAGTTTT

>197894

TGCAGCCTGTTGCTCCTACGGACCGGGCCCTGAATGTCAAGAACCGCTGCCAGCCTGGAGCCTACTGCTTCCTCTACCACCCCCCCGACCGGCCCA

>1510

TGCAGGAATAAATGCCACAGGTAGGAGATCCATCTGGTTCTGATCAGAAGTATAATTGTCATGTTAGTATAAGGGAATATCATGCATTTTGAAATG

>240371

TGCAGCAGAGCCTCGGCGTTACGATAATGTCTCAGAATGTGTTGAAATGTAATGTTGGAGAGATTCAAATAAAATGTTTCAAGTATTTGAGTTGCA

>256724

TGCAGCTTAGCTGGTTCCTGATTCGCCACATCGCCGTTCTGAGCTTTGAAGCTTTTCATGCCTCAGTTATTGACTTTAGAGTTCAGTGGTTTATCT

>27263

TGCAGGGTTTCAGATGTCTTTCCTGATTCTTTTGAGAACGTATAGATGTGTGAACAGTGTGCTCTTGGAGTACATCTGGTAGAATTTGGAAAGGTT

>91432

TGCAGCTCAACGTCACGATGCCGGCCACCGTTTCCTGAAACGGACCAGAACCGAATCACCTCACGGATATCGATCATCAGCCTCAATTTATTACGA

>131325

TGCAGAGGCTGCGGGTGGAGATTCCCGTAGGCCACAATGGTGGAGTCGTCTGAGAGGATCAGTCTTTGGTGGGCTGTGATGGGGTCAAGGGTCAGA

>209382

TGCAGACGTACCGTGGTGGGAACCCGCGTCGTCACGTGACCGGACCGCGGCGCTTTCTCTCAGCATTTCACACCACACGTAGTCAAACAATTAAGA

>108825

TGCAGGCACCGCACAAAAGACAGAAGAATGAAAAAAAGCTTTTTGGAACACAATTGGTTACGCTGTGTCAGAAGAGCAGGTTTGTGTAAGTCTTGA

>54622

TGCAGGTAAGATACCCTGCCAACTACCTAGGTGGGTAGGCAGGTACCCACCCACTCATGGTTTTATTGATATTTTTGTTCGTTCGTTCCACTAGCG

>220340

TGCAGTCAGAAAACTTTACACAAAGAAACACGTGCAGTGAATTTTCTTCTTCTTGTTCTAATAATAGGCAGCGGCTGCCAATCAGCAGCTGCACAT

>281531

TGCAGCCTGGAGCCTCCGTCCAGAGGCCGACCGGCGGCTCGGCTGCCACAACGTCTGGAAACACAAAACATCTGTTTGCATTTCAGTTTCAGCTAA

>156298

TGCAGTGAGCATCATCAGAGTTTCTTTCCTCCCTTGTTTTGCTGTGCAGTGACTCAGATCGTCACATCGGCCCGTTTGGGTTTCAGTGCTGATCTG

>9325

TGCAGCAGTCGGTCCGGCCCGGTTCGCTCTCTGGACCCAGACAGACTGAACCTTATTTTTGGCTCCAAGGCTGCGCGCCTGGAGCGCCGGATCAGT

>185711

TGCAGCGCCGAACAGAACCACCTGACGTTACTCATTGGATCGGTCAATAAAAAAAAAAAGACAAACACAAGATTCCTTTGAACCTGCATCAGAATC

>116683

TGCAGCACTGGGTGGTAGGAAACATTTGCTGCCAAACAGGAGATATTCTGAGTTGTCACAAACTGATCAGGCAGCTGGGAGGAGAGGAAGCTCTCA

>230670

TGCAGTGTTTATTCTTCCAAATCAGTTTGTGTTCACTAATGAATTTATGAGTATTTGATGCCTGAGGCCAGACATTCCTGCACTCTGCTCCAATCA

>47625

TGCAGGAGGGACGCCTCGGAGGGAGTCACAAACCAACCGCCATGTGTCAGATGTTCCATCAAAGGGGCTCCAGTCAACCGCATGTTTACAGACACA

>75925

TGCAGCTGCTGATGGAAAAGCTGTGGTTACTGCTTGAACTTTGACCTCTTGTCTGGCTACATCCACGGTGCTGTGGTCACCGTGGTTCTGTTTGAC

>39605

TGCAGCTGTTTTCATTTTACATGTTCACAATTAGATGCCATCAGCTCCTTTGGGAGTTCGCTGCTGCTCGGATATTGAGTTGTCATTTCCTAATGA

>123470

TGCAGCCTGGATTCATTGTGCTGCTATTTTCCTCATCCTTCACTTTCCCCTCTGTGCTGAAACATTTCCTTCCCGGCTCTGACGGTTCACTTTGCG

>111826

TGCAGCGGAGGGCAGGGAGCCGGCCAACTGAGGCAGAGCATCTGAGAGGGAACACACAACACGTTCATCCCATTATGGAAGATTATTGTTTTACAG

>183876

TGCAGTCAGCCGTTGGTCCAGGTGTCTCCTCCAGGCATCTGGGGTCGAACTGTTTGGACCATCATCAACTTCGACCACAAGGTCCCTCTGCAACCA

>298721

TGCAGGTTACCTCTTTGGCCCAGGCCGGCTTGTCGTTGCTGCCCGGCGCCTGGTCCTTGTAGTGGTACAGCTGCTTCTTGTCCTGCGGCGGCCGCG

>133254

TGCAGCCACTTGAGAGGCGCACACAAGGCGGCCCGTTGTGCAGCTGAGGAAACGGACAGAGCCAACACCAACTGCACCAGTTTCAGCTTTTCAATG

>130311

TGCAGGAGAATATCGGGAAGAGACCTTCGAACCAACTCTGAGTTGGAAGCGATAGTCCGGCTGATGGCAGGTGAGGAGCCTGTTTGTCTCCCCACA

>24907

TGCAGAAAGGTCAGGTCTGGGTGCCGAGAGTTGGCCTCAGATTTCATCACCCTGAAGGTACAAGACATAAGGTGGTTACACATACTGCACGTCTGC

>170858

TGCAGACGTCACGTTGCAGCTTTTCTCCGTTTTTACTGCCCGGTTTGGTCCCGTCTACCACTGGGTGAGTCCCGGTACTGGCTAATTCCTGCCAGA

>333650

TGCAGCTTCCCTGAAGGGTTCTGCTCTGCTTTGGCTGGCATGCACTTCACATGTTTCTGAGGACACTGACTTGAATAGGAAATAATTCTTTAAGAT

>279544

TGCAGGTAAATGGTAATAACAGAGCTGTACTTGCAGACCGCAGCTCTGCAACAATGATTGACCTGGATGATAATGACATTTCCCGCCTGGCCATTT

>126626

TGCAGGTCCAGATGCCCTCCAGAACCTTCCTGACAGAGATCCACGGTGAAGAGGTCTCACTCTGATGTATTTCCTGGTTCCGTCCCTCCCTGCTGT

>169762

TGCAGCTTAGAGGAGCGACTTTGAGTGTTCAGAGAGTCGAACGATTTGATCCGTAGATTGGAGGGGTGCGGCACGCATACGCCGTCTCCTGGGACG

>1657

TGCAGGCGCAGCGTGAACACAAAGGTCTCCATGAAGACGACATTCTGAAAGCAAAGCAAAGCAGAGCAGGTGAAAAATCATCCTGTCGCCTCAAGA

>144492

TGCAGTAGCTTCCCGCCTGTCTCTCTTTTGGATTATATAACGAATAAATGCCGGAAATACAATCTGCTCAAATGAGACAAAACATCGCTGACTGCT

>276706

TGCAGGTCGTCATCCACCAGCTCTCAGATCGACCTCACAGCAAGGTGCCCCCTCATTACAAAGAGCTGGGCCAGCTGATCCGGCAGCTGTCTCTGT

>102176

TGCAGGCCGACATGATTATCAGCGAGATCAGCAAGAAGCAGAAATCCATGGCGAGGAAAACGTCTTTCAGCACGAACAAGCTCAGCCTTACGACGC

>328308

TGCAGCTCAAACCAGGAAACGCAAAGACGAGAGCAGAGAAGAGAAACCCATCAACTCATCATCACATGGAGACTAAACTTCCTCTGGGTTTGCTTC

>177293

TGCAGCTCCACGGAGGCTGGGGCTACATGTGGGAGTACCCCATCGCTAAGTAGGTGGAGCAGTTTGGGAGTGTGGGGGGCTGAGTGATGTCTGCTT

>105286

TGCAGCCTCTCTGAAGTGAGGCTGGGAGGGTTTCTTATCTCTCCATGGTGAAATAAAGGCTTGTTCTTGGTCTCATTGTCAATATTGTCTTTGCTG

>104620

TGCAGCCAAGTTGGGTTTGTCTTCATTGCAATCTGGGCGAGAAAATGGGCAGAAACGTGAAAAACATGGGCTGGCTGCGAGTGCGAAGGAGCTGTG

>172771

TGCAGAGATTCACAGTCCCTGGATGGTACCGCTGTAAGAAACAAACTTGTAAAAGATTTGAAGCTGCAACTGTCGTAATATGTTCTGATGCGAAAC

>302528

TGCAGCTGGGCGGGGGTCACCCTGCTCTGGGAGGGGACGGACATAACCCCGTGAGCCATATGTGATACTCAGGTACGTCTGCTGAAGCTTCACTAC

>194265

TGCAGCCAGAACCAGACCAGAACCCAGTAACCCAGAGACGTGACGGCCCGGCCCGAAGAACCGAACCGCTCTGAGTTTCTGCATGTGGATGTGATT

>20244

TGCAGCTGGTTCTGGTTTCAGCTCTCTTCTGGGCTCTGTCTACTTCCTGGATTACCTGGAAACATTTCCTCTGCTGTTCTATGTGCGGAGTCCGGA

>190092

TGCAGCTCATATGGTTCTGATGTTCTAAAGACAGCAGCTTCACTTTCTCTGCTTCTTCCAGATTCTCCACAATCACATCGGACCCTGATGGCATAT

>166097

TGCAGCAGCAGAGGGATGTTCAAGAATGTAAGCATGACCATCCTGCTGGCTGTTGCTCGAAGTCTCAAACTCATGGCCAGCTTCCATTTATCTTTT

>113676

TGCAGCTCCAACCAAAGCTTCACGATCTGCTGAACAACAACGCCACCAAAAGCTCCAACAGCTACTCCACAAAACACTGCTGACGCCCCCAACCAA

>90565

TGCAGGAGCAACTGTGAGTCTCTAAACATGAACTCTTCATCGTGTCTGTCATAAGATCAGATCAGGGGAATCTTCCAAACTTGAAGGTTATAGCAG

>129717

TGCAGTCAACAACTTGAATCTCCAGATTGGAGCTGACCAGACCCCAGCCAGTCCAAAAAGTTATTAGATTCCTTCGGATGTCCGACGTCCCTTTAC

>1580

TGCAGCTGGAGGAGAGAGAGGAATGATCAATTTGCTCTGGGTTTCCTGTTTCTGCTTCTGGTCTCTCTGCCTCTGTTTACAGAAATAACCAAGTGA

>312884

TGCAGCAGGAACAGGAAGGCATGGTTCACCACACAGCTGGGGATTACCAACCAAGGTGGTTCTGGTTGAAGAACAACAACCAGCCTGTTGAGACCA

>158671

TGCAGATCTGGGTTACCTGTCTTTGATCTGATGAGGCGCGAGCCGCAGCGTTCTGCTTCCTGGGTCACTGAGTCTGAAGGAGGCTTTAGCTTTGAG

>189071

TGCAGGCTTTACAGAATACCAGAGGCCTTTCTTGGTGAAGCTTATCTTGGCTATCCAGAGCCTTCTCAGCTTGTAGGGATATTCTTATGGTATGTA

>243329

TGCAGCCTCCGTCCACCGGGGGGCGGCAGAGTGACATAGAAACATGACAACTACAGTTAGAAACTTTCCCCCTGTTTGTGATCACACAGTATGATT

>94547

TGCAGTGCAGTCTTCCAACTGTTCCAGGCAGTGGACCAGAAAATTGCTCAACAACACCATAATAAATCCCTGTCATGATAAATTGTATCTATCACA

>44100

TGCAGCCCCAACCGCACCATGTGTGATTGATGAGCGCTGGTTCCCACGGGCATTTACAACATTTCCTCAAGCAATGTCCCCTGAAGAACATGTGCC

>137788

TGCAGGACGAACCGAGACTGAAACAGAATCAGACGTTTGGTTCAGGACGTCCCGCCTGTTCCGGGTCCAGATGTAGGCCTGATGATCAGCCTGGCA

>158211

TGCAGGACAGAGCAAATCTGCTGTAGCCCAACAATGGCGCAGCAAATGCACACCCACATGTAAAGGGGATCCAAGGTCAGCGCGAAACCGACACAG

>97748

TGCAGGGCCACCCAGGACAAGCTGGGTCCAGAGGGAAGCCAGGGGCAGACGGCTGTAACGGGACCAAAGGAGAACCTGGATTCCCAGGATTACCTG

>309428

TGCAGCTGTATTTACAGTCCGGGCCGAGAAACAAACACGCCTCACGGCTCCGAACCGCAGGGTCAGGAGCAACTAAACGCCGCTCGATATTCATGA

>337563

TGCAGCGCAGCAGCACTACTTCCTGTGTGAATCGGACTCTGAGCTCCTGGTTCTGTTTGGTTCCGACTGGAGGAACCGACCCGAACAGACGGGTCC

>296265

TGCAGACAGATCTGAACTTCCTGTTCCGGCGGGACAGGAAGTCGTCCCTCCAAACGAAGCCCAAAGTGACTCTGGTCTCTCTTTGACTGCGTCTCA

>144210

TGCAGGAGTCCTTCTGCCTTTTTATAGGGTGACAGCAGCACCAGAACATTTTGGTGTAGTTATGTTTAGACATCCTTCATGTACGTTGAACCTTGA

>309966

TGCAGCAGTCTGGTGTCTCCCGGCCGTCCTAACCGGAGCGTTTTCTCCTCCGCAGAGCGCCGTGACACTCCGGGCTTCGCCAGACGTCTTCGCTCC

>159702

TGCAGAGCCGCTTTATGACGCCTGAACCAACCTGTCTGGTTCTGCCTCAGACCGTCAGGTGATCCAGGAGCTGCCTGATGACACCAGGATCAAACC

>173030

TGCAGCCAATCAGCTGCTGGACAAATCAAGACTCAAAATGTTGGAGCTTCTGGTTCAGAGCTGAACACATCAGATGAGATGCTCCTCATTTCTTTG

>4823

TGCAGCTCCCAGGACGTGGCGTCCATGACGTCTCCGCGGATCTCCATCCTGCGGCCGCACATTCCCGGCTGGTCGAAGATGTAAATCTGTTGAGAG

>135639

TGCAGAGCGGCGCCAGCGAGCGGATCCTCGTTTCTGACCCAAAAACTCCACTTTCTGTTTCTGTCACTAAAAGCCAGCAGAGAAATACAGAAACAT

>174904

TGCAGGCAGCTGGCCGGTTCATGCAGCGTTAGCATCATGCTAAGAGACGCTAAAACAATCTAAAGGATAAAAACACAAGCTAATATAAACAACGCC

>88876

TGCAGTGACTTCCTCCTGCTGGCATTTCTTCTGCTTTCCTTACAGCAACAAGTCAGACCAGTTCAAGCGATGGGAGACTCATTCATCATAGCTATA

>229927

TGCAGAGCAGGAAGATTATGCAAAGAGAATAGAGAACGGTCCGTCGTATTGAAAAGCATCGGTGAAACAAATGGCCTCAGGCTAATCCATCAGCAC

>309861

TGCAGTGTAAAAAGCTCCATTTGATTCACCGTCTCTCATCTTTACAGTCTTCTAAAAGCAAAACTAGAAACATGGCTAAGAAAGGAGAAACTGTTA

>258356

TGCAGTTCCCCTCCTGACCAGATTCATGGCGCACCTGGACAAGCACTCCCCGCAGCTGCTGAAGATCTTCAGAAAGAAAGGAGGGATCACTAAAGC

>264413

TGCAGGGACCGGCCGGCCTCTCTGATCAGTCCCTCCGTCTCCTGGGCGCTCAGACCGGCCAGGTTTCCCTCCATGGCGATGGTGCAGCAAGTCGGA

>7920

TGCAGGAGGAGGATTTTATGATCAAGATGGAGAGATGGGAGAAGAGAGATGGATGAAGGGAGAGAGGAAAGAGAGATGGATCAGGAGAGAGATGGA

>311716

TGCAGATTGAAGTTTCGGATCCTTTCAGATCTGTTCCGTCTTTGGTGCTGTGCGCTTGAATCCCCACTTCAGAGGAGTCATCACAGGAGGAGGAAC

>98675

TGCAGCATTTCAGCACTAAACAGTCCTTCAGCACTAGACCTTCATCAGATGATATGCGGTAAAGGTTTTGGCCTGATGAAGGCCGGCTACTTCCAA

>213614

TGCAGAGGCTATTTTCTAATATTATTTACGCAGCTAAGCTGGCTTTTTCCTAGCTTTTCACTGAAAGGCTAAAAACACACAAATCCTCCCCAAAAG

>118579

TGCAGGGAGGGCATTAGGCCCCAAACCCTTCACTAGGTCAGCAGCTGGAGGTAGTAAACCTTCCAAAATGCGCCTTGTAACATGTAGAGGTGCTAG

>45290

TGCAGACCGGATCAAGCCATCCGCTCCCGGGAATACTCTGCTGATCGCTCCAATAGGCCATGAGGCTCTGGGGAGTTGTGGATCCACTATCATCAC

>5743

TGCAGCTTGGAGGTGTGATGAGTTTCACTTCGGATGTCAGAACCATTACACAAGCCTGTTTATCAAGCACAGCCTGCCCTTGGATCAGAGCTGACA

>202100

TGCAGGTTTCCTCGGGAGGACGTTCACTTCACCATAGTGGATTCCTTCATCTGGCTTCTTCTCTGGTGTTTGAACCTCCATCTCTGTTTGAGTCTG

>189120

TGCAGGGCCGCTGTGACTCATTGAGAAATAACTCTGGCTCTCCAGGCTGATGCATGAATGAGCGCCACTGAAGACAACCCGCAGTCAGGAAAGACG

>155603

TGCAGATTGGAAACGTTTTCACTGATGAGCAGCGGAACCAGAACCAGAACCAGAACCGGAACCGGAACCAGAACCGGAACCAGAACCGGAACCAGA

>82205

TGCAGATAGTCACAAAGATTGGAGGAGGTAAAGGGATCGATACGGTGCCTCCACAGAAGATCTACAGCTTTCTGATCGATTTCTCCCTCTGATCGG

>186779

TGCAGCCTTTGCTGTATTGCAGCACTACAGCTTTGCTGTTCCAGAGACCGAGCACACCTTGGATATTCCGCAGCACAACACACACACACACACACA

>84483

TGCAGGTGATGCTGCCACTTACATAGGTGGACTGCACTCTCCGCCAGTGTCCCAGCCCACAGCAATGCATCAAGACATCAAGTTTTAGGGAGAGAA

>101844

TGCAGCCCAGGTTCCTTGAAATAGCAGCCTCGGCGTTTTTGATGATAAATGTATGGTGTATCAGCTGTAATAGAGCTCGCTACGGGGGATCCTTCC

>244230

TGCAGATCTGCTTCACAACAAGTTTAGGTTGATGTTTGAGTATTTCTATTTTTTATTTCTTTAGGTTTTTGTTTTCAGATTTTTCAAGCGACAGAT

>33679

TGCAGACGTTTCCTTTGCATTTTGACGTGAGCTAAAAGTGTCCAGATGCAGAACGGCAGAACCGTTGGCTTTCTTCATGCCAGATGAGTTTTGTTC

>173203

TGCAGGAGGCGCCGTCCTGGACGCCTCCAGCTGTGAACCCGGTTCAGTTTGACTAAACGGATCCATCAATTGAAAAGTTACTTGTTTATAGGGGCA

>102870

TGCAGGACAGGAAGCTTCAGACAGTAATTGGATGAGAGCTGTGAGGAACATGAGCTGCTTCGCTGCTGAAGGAGGTCAAACTCTCCTCCTCCACCT

>29115

TGCAGTCCATGATGAAAGATAATGAAAGACCTTGAAAATGTGATTCGAGCCATTAGAACAAACCATTCGAAACGCGACAGCTCATCTTTTCACAAG

>110806

TGCAGCACACACACACCGACACACACGGCTCGGGGCCGGGAAAGCCCCGGGTCGCAGAAAAAGCCGTGGGCATCACTGGCCAAGAGAAGACATGAC

>147472

TGCAGCAGCTTCACTCCAAGACGGAGCAGCAGGAGGAAAACCTCTTTGTTTGATGACATCAACAGAAAATATCAAAATGTTTCCAATCAGGGGAAT

>141241

TGCAGAGACTCGTCCCTGCGCTCAGCGGGGAGCGAGCATGTGATAAATAGAAGTGTGAATTATGAATGAGTGAAGATGAAGGGCTGCCGGTGCCTG

>43318

TGCAGGATCAGCTGTGACTCTGATGACGGCGATAAAAGCGAACATGTGTTTCTACGTTTTGTTCTTGGTTCATTTCCAAGCCCGAGGAGTGAAAAA

>118960

TGCAGGCGCTGCTGACGTCACTCCCATTGATAAGAATGTTGGTGGGGAAATATCAGTGTGAGTCTAGGCTGGCCCGAGCTGATGAATGAACGGAAA

>281141

TGCAGTTTCCCCGTGTGGTGCGCGTGTGTGGCCGGTACCGTCACTCCCGGGATCTGCGGCAGGCAGCACGGCGGCGTCCTGGCGAGAGGGGAGTTC

>195400

TGCAGTGCAGCTTCCCGCCATCAGTGCGTGTGATGTGCAGAGCAGCGGTTCTCGCCTAAGGCGGCCTTTCTTCTTCAGCTATTGAAAAGCTCTGCA

>201205

TGCAGTCCCCCTCTCTCCCCGTCCTCTACCCCAAACCTTACACCAAGGCCAAGGACGGTTGCACAGATGGACCGAGTCAAACTCAGTCAAACTGGG

>71028

TGCAGAATTTCTCAAGTTGTGGAGAAACAACAAAGACGTAAACAAACCGTGCTAATGGGACGAAAGTGGAGCGAAAACATCAAAACAATGTATGAA

>133509

TGCAGAGACACATCAGGAGCATGAATGATCAAATGTGGAGCGAGAGTGAAAAATTAGATGAACGGACCGCGTTGGAAGAATGGAAAACTCAGAGGG

>138400

TGCAGAAGACGAAACGAGTGACCGGCCCCAAAAGATGCTGCTGGTCACTGTGACCTCTGACCCTGAGGCTTCACATGTGGTGTGATGATAACATGA

>185662

TGCAGATTAGACAGATGTTTCTCCAGGTGGATGTGTGAAGACTACTCACTCCCTTGCTGACAGGAGAGCCGGCGTAGGTTTCATACGGACCAAATT

>308634

TGCAGAAAAAGTCAACATGCTCACTCTAAAGCATTGATTGTCTAGAATTTCCGCTGGCACAATGGAATCCTTTGTGCTTTCAAAGGCATGAAGACA

>32884

TGCAGGTGGCTGTGAACGGATGACCAGGGGGAGAGAAAGGAGGGACTGTGACCGCTGCTGTCTCCAAACAGAAGCAAACGGCGAGGTGCGCCGCCG

>33666

TGCAGGGAAACCTCTAAATCTCCTCCACCGCGAGGAACCGGCATGCTGGCGTCTTCGTCGTCCCCCTCGTCTTCCTCAGCCGAGCACGCTGTCGTT

>149533

TGCAGTTTTGCAGTAGCAGCACCCAGCGCATGCATGTTGTTGTTTTTCTGGAAACCTGAGCAGCAGCAGAAATACAGACTGACAGCATCTATTCAC

>52359

TGCAGACAGCAAAACACACACAGGAAACTTTCTTTCTTCGTGCAAGCACATAATAAGAGCTACATTTCAAGTTCTGAGGCCGACGGCTCAGCAGTT

>157573

TGCAGAGACTTTTGTTTTGGCGAAGTGCTTGAAGACACTCATCACGTAGGACTTTGGTAGGACCGTTTTCGTCTTGGGCACCCCAGTTCGCCGCCG

>268139

TGCAGCAGACAGCCTATTGGACCCCTGGGGCCCCATTAGGATTTTACATTATAAGCTGTAAAATCCTCTATTTCTGTCCCATCATAACATGACGTA

>260065

TGCAGAGCGGTGGTGTAATATCTATTCTCTTGCAAAGTGTCATGCAAAACCCGACTTCGATAACCCAACAAAGGAAGTAGCACCTAGATAAACCCG

>217155

TGCAGAGCAGTTTGATTCCAAGAAAAAACCCACCCAGGACCAGACATCCTCCCAAAAAGCTGTCGTATGAAGCACATGTCACAAAAACAGAAGAGG

>133803

TGCAGCCAGCTAATTTTACATGCAGCACCGTCAGTACAGCAGACAACACGGAGCGTTCCCCACAGCCCGGAAACACTACACGGGAAAATAAAAGTC

>317266

TGCAGTTGTTGCATTTTCTACATCCATGCGGGCGGCTAAGACGCCGCTTTTCAATTTCATTCCAGGTTTACTTCTGATTCTGAGTAGCACGCAGGC

>90270

TGCAGACTGAAGCCGACTCCACAGAGCCAGGCAAATTCCTGCTGCCTTTTTATGTTATTTTCAGAGACCACAATGACAGAGCAGAAAAAAGGGAAT

>269353

TGCAGAGTGCAATAGGTCAGGTGCAAACAGAAAGGTTGATTGTATTAGAAAATCCCCCCACCTGCCCCATGTCCCCCTCCAAAAAATAAAATCTAA

>273307

TGCAGCAAACAGCCGCACATCCAGCTGATGATACCGGATCATCAATACTGACAGTGAGGCCCGGTGAAACCACCATGACTTAAGATCGGAAGAGCG

>78221

TGCAGGATGACATCAACTGATAGGTGGTGGTAACTTTGATCCTGAATAAGTTTATGATAGTAATTAGCCTATCTAAAGCAATAAGATGTCCTGTTC

>154791

TGCAGCAACTAAAGCAACATTTGAATTTGCTCTGTAATCTGAGACATTGTGGACAGTGGGCCCAGAGGACCAGCAGCTCTGTGGAAGAAAATACAG

>65647

TGCAGGGGGCGCCAGAGGCTCCAGGGGTACGGAGAAACTGCGCCGGAGCCGGGTGATGAAAGACGAGGCGGAGCAGATCACACACACACACACACA

>153279

TGCAGGATCTGAGCAACGTGGCGGGGGATGGCACGCAGCAGCCGGCGGCTGGAGTGGCGACCGGTGGCGTGCCAAAAACCCGAGCCGAGCGACAGG

>155776

TGCAGCTGAGACAGATTCATCCATCACACACATCTACACACAAACACACATGCGCTCGTAGTATGCACATGCTCTGGGGGCCAGTATAAATGCACT

>66010

TGCAGATGTCCCAACACACAGACCTGAACACCACTGCACATTTATACAGTGTATCTCCTGCTATGATGTGATGTCACAACTACCGTCATTGTGCTT

>70068

TGCAGTGTACAGCATTTTGTTGCACAGTCAGTCCTGAGTGCTTCTAGAAATGGCACCCAAGAAGAAGGAAGAGCCAAAGCCTGCTGCACCGGCGGC

>101863

TGCAGGTCAGTGGCCGCTAGCCAGCTGATCGCACAGGTTTCTCACCAGAACTTGGCCATGGAGGCGGTGGTGGGGTCCAGGCGGCGCTCTGAGAGG

>110788

TGCAGGAAATTACGTCCTGCGCTGCATTTGAGGTTTTCTAGTTCCTCGTGAAATTTCTCTTTTTCATGAAATGTTTGCTTCTTCGGCCTAAAGGAA

>20913

TGCAGTGTAAACACTCATAAAGCTGGTTAGCATCTGTAACAGTTGCTAACCTTTACCTTTTACAGTTGCTGCAACGTCAATCAAGGTTTTTCCATT

>303083

TGCAGATAAAAGCAAGGTGTGTGGAAACAAACAGGAGTCGTGCAGGAATTGTGATTGATAACTTTTTAGCTCTACCTTTATTTACATATAGATGAA

>205099

TGCAGGTCAGATCCAGGCTGCCTGGCTTCCTGTTTCTCCACTTAGTCTGGAGGCAGAACTGATGGCTGCCTGTGTTGAAGCGCCACCTTGTGGTGA

>44549

TGCAGCTACTGGCTCCCATCAGCCTCTGCCTACACTGGCTCTGCTTGGACCCAGACCCCGACACCAATAGAGACGGGTTCACAGAGAAAGTTCTAG

>224225

TGCAGACACTTGGGCGCAGTGTGTGGTTACTCACTAACTGATGTTACATTTTTGACGAAAACATTCAAGACAAGAGTTTTTCCAAATGGAAAACTT

>327503

TGCAGCTGGGGGGCGCTGTGCTGCTGCTGCGCCACGGGGGCATCAAACCCCCCCAGCTCGTCGGCTGAAGTCGTCTGTTTTACATTAAGATCGGAA

>1812

TGCAGGTTTGGGTCGGAGGAGCCGGGTTCTCACCGGTCAGGTCGTTGGTGTCGTTGGTGACGCAGTAGCAGAACCGCCTCCTGAACAGACCGCTTT

>121237

TGCAGTTTTGGCATGCTGACCCACCGTAGGCGCTTGCTACAAACTCTGGATTGTTCTCCTGCTCTACTGTAAGCAGGTCGTCAGCTGTCTGTGTAC

>51695

TGCAGGATTCAGAGAGCAGCAAACAGACGACAGACAGAGCAGCATCACAGCAGCATCACAGCAGCATCACAGCAGCATCGCAGCAGCATCACAGCA

>139217

TGCAGCGCCGCAGATCTGTCCGTCAGCCTGCTGCACGGCGGCGGCATGTGTCTGTTCAACGTGCCGCAGCCCGACCGCCTGCTGGGCGGACCGCGC

>233895

TGCAGCTTTATGGGCGAGGAGGCAAAGCACAAGCCGAAATGAAATGTTGAGTGTGGAAAGTTTTATCCTGAAAAATCTGCAAAAACTCTCAATTTC

>255333

TGCAGATAGAATATAAACAGCTTCTCATTTTGGTGTTTTCAGTTTTTTACCTTCTGCTCAAGTTTTCATTATCCTGTCAATGACTGAGTCATGTAA

>6193

TGCAGTTACCAGGTATGGAAGACGCATGGAGGTGAGAGGGGACCGCAGAGATGGAGATCGCAGGGGTGAAGCGGCACCCTTTTATCCCCGTACCCG

>299955

TGCAGCTTCTCATGCAGTGCCAACAGAGGTCACACACATTCAGCACCTGCTTCTGACACTCTGAGATATTCCTCTAGCTACCTTTTTATAACTGAT

>152344

TGCAGCTGCCAGGATATTTTTTAGTGAATTTTCCCCATGTTTATTTACACTCAGCTTTCTTAGCTGTAGGTGCCAATTCTGCACAGATCAACACTG

>58789

TGCAGAATGACTCTGCAACCTTCCTCTTCCTCCTCTTCCTCTTCCCCTCTGCTGGCTTCAGCACCCATGGCCCACCGCATTGCTGCCACCGGCACA

>297992

TGCAGAGAAAATACAGTAAACAAACGTTAGAAATGATGGTTCCTATTTCTGCCCCCACAAATCCCCAAATCCGAGCTATCAGACCGACCAGCCACG

>83117

TGCAGGTTGCAGCAGATCGCAGACACAGCACGTTTTTAGCTGTTGTTCTGTAGGTGAACAGCAGAGGGCAGCATCACTAACCTGACTGGAGGCATA

>238353

TGCAGCTGCTGCTCCGCCTGGAGCCACAGAGAGAAACCAGCAGAACCAGTGACCCAGTCAGCTGGGAACACTGGGAAACAGTTCAGCAGCAGGTGG

>150160

TGCAGCAGCATGCTGCCAACACTTAGCACAGCAGGCAGAGGAACATCAGAACCACAGGGAAGCTGGAGGAATGTTTTTGTGGTGCAGAGCTTCTGC

>276350

TGCAGAGTCCAGGACGGATACCGATTTTCATTTCCCTACCTCAGTATCAGGCTGAGGCTACCTGTCCCTTACTGTTGTTTTTGGGTGCAATGTTAA

>299961

TGCAGCTCACAGTCAGCACTTCCTCGCCTCAGGGGCGAGTCACTCGAGCTCACTTCCTGTTTCTGCCTGGAACCACAGCAGCTGCTTTCTTCCTTA

>241349

TGCAGGTTCTTCATGGTCGATAGCAACGTCTTTGTTTTCGATGAAAGTCAGTGAAGATTAGACACCAGGATCAACAAAAGGCTCATGAAACAAAAT

>286978

TGCAGGTACAAGGGATGTCGCCCAGGGCACCATTCAACCACGGGGCGCCACTGACTGAGAATTTATTGAATATTTTATTAAGATCGGAAGAGCGGT

>124448

TGCAGGTCGTTGAGTCATTCTTCCTTAGCAACCCGAGATCACGTAAACATTCATGAGTGTTCGTACTTATTGTTCCGAGATCGCTTGATGATCGTA

>32723

TGCAGGGTCTCCTGCCGTTCCTGTGCCGCTGCCTGCTGGTCAGTGTGACCCAGAAACAGCGGCCCGGTCGGCCTGAATATGAATAACCTGCCTGGA

>104631

TGCAGTCAGCAGGGACAGAGTCTTTGTTTTACTCAGACTGTGACTCTGCAACAAACTGATTTCAGTGAAGAAGAACCAAACTTTGAGCATCTGGAC

>81314

TGCAGGAAGCCGTCCTCATGCAGCTGACTGAAATCATATGCATGAGTGTGAGGGTCATTGTGGAGAGCAGCAGCGTTTCCTCAGGACGCCACCGAG

>2897

TGCAGGCTGTGTGCGAGCTGCTGACTTTAGTCCATCTGAAATTATCAAGTAAAGCTGAAGTAGGAAGTAGCATGAATGTCCCTCCTGGTGGGCACC

>184235

TGCAGGCCTGTATTTATCAAGAACTTGTCCAGAATCCTTAGATTGGCAGTCATTCCATTCCAGGAATTGCTTATTATGACCAAATCATCAGCATAG

>339822

TGCAGTACAAAGTAGCATTTTGGGATCACTGTATCTGAATCATAACCACTAGGTAATATCAATGGTGAAAACCAGGATTTCTGGGAGGAACCTCAG

>69898

TGCAGCCCTCCGAATGAGCGGGCCCTGAATGACTCGATGATAAAGAGGCCCATTACCGAGCCGTGATTGACCAGTACCCAGCCAGGCCCGCTCGCT

>190150

TGCAGGAAGACGCGGCTGGATTAGACATCTGGACCGAACTGCTTCACTTCTGATCCTGATTAGGACGAGAACGATAATCATCTGGAGGAACACGGC

>55773

TGCAGACCGACCCGAGGGCATGGCGCTTTACCCACAATGCACCAGGGTTCAACTCGGATCCAAATCTGCCGACACGCGATAACGTAACCATGGCGA

>228794

TGCAGTACATTCCTGGTCCTGACCGGCATCCTGGTTAGGATAATAAGTTCTGTTCGCCTATTTTGAGAGTTCATTATGGATGACAGGCGTTTAGAG

>179989

TGCAGAAACTGACATCTTGCTCCAGTATGGCTGTTGATGTGGAGGCAGAGGTTTCTGCCAAGAAAAGGTTGCAAGTTGAGGTAAGAAAGATTTTTT

>219358

TGCAGGTCGTTTCTCCAGAAGCCGACGCCCCTGGAGGAGCAGACGGCGCTTTATCATCATCAGTGGGACAGGTGAGATGACATCATCATGTTTGTG

>248541

TGCAGGCTTGTTCTCTGGATCAGATTCTGACTCCAACATGTCAGAATCTAATAAACTTTGAAGAAGAAAGTTTCTCTGCCAGTATATAATGTGTTA

>23239

TGCAGCTCACACAAAAGAATGTCTTCGTTTTGTCTCCGTTTGGACCGTGCAAGGAAATACCAAGCATCATATAACAAAGACAGCTTCGATCTGAGC

>229544

TGCAGCACCAAATGACACCAGTAAACAAGTTTGGGAAAATGTGGGAGAGAATTTCAAAAATATATGAAAACATGACCTAAATAAAAAGTACACCTC

>148291

TGCAGTTTTCCAGTTTTTGCCTCCTGGATCTCCTGCTTCGCCCCCAGACGGGCTTCCAGCCGGACTGCCAGCAGTAAGACGCTCTGCTCTCCCTGT

>109199

TGCAGGGACATGCTGACAGTTTGAACACAGCAGGACAAACACTGATGCCAACAGAAGGTTTATATAAATAGAGAGAACGGACCAGGCTCACAGTTC

>2785

TGCAGCCTCCATCCCCTCCCACAGCGCCCTCAGACTCTGCTCCATCTGAACAAGCTGCTCGGAATCCATTCCTGCTGGTGGAGCTGGTGACAGAGC

>184193

TGCAGCGGTTCTGGAGAGGAAACCCGACCTGAGCTGCCTTGGATGTTCCCTCTGTGGTCGACATCCTGAGTTTCTATTAAGATCGGAAGAGCGGTT

>134968

TGCAGTTACATGGCGGACACACGAATCACCTCTCCCCCCCCAAAAAAAGTGGAAGGTACAAACTTCACTTTGCCCTCTGTGAAAAGCTGCATCCTC

>295868

TGCAGGTTGGAAAATGCAGGCAAGTTACGCAACGTTCGTTGCTACTCAAAGATGTGACATTAGCGCCCTCTGGTGGCAGTATTTTGTCCTGCCGCT

>43958

TGCAGTGCTTATTAGATGCAACCAAATTCCAAAAATAGTCTTGCTTTCACAGTCATGCTTACCGTTTTTTTCAGCTTCCTGCCTGAGCTTTGATTC

>218251

TGCAGGACTAACGAGGACTTTCAACTGAATGAGCCGGATTTTCACCGTGGACCGAGCTCTGATTATCAGGACATTTTACTGAGCAATGAAGCCTGC

>68362

TGCAGCTGAAATCCAGATGGTTCTGGATGAAAATCCTCTAATCCAGCAGCTTCTCGTCACATGACCTCCTTCCTCACGTCTCCATGAGGCGTTTTT

>132058

TGCAGAAATCACGCTGATTGGAGAAGGAGATAGAAGGAGCTGCTCTTCCTTTGTGCGCGCGCGCCCACACGCTCTCACGAGCGCGCCGCCTTCCGC

>299263

TGCAGCTCCTGCCCGTCCAGGAAGGTTCAGGCTTCAGCTTACAGCGCTGCTATCAAGGGGCCTCCAATTAAGATCGGAAGAGCGGTTCAGCAGGAA

>293137

TGCAGGCAGACGAGTCGGCCTGTGCTATAGCCAGCAGGGAGGAATTCCACCACGCTTTCCATCTCTTGAGCTCATGCAGAGGTCTTTCTTTAAGAT

>24837

TGCAGACACGTACTCGGGGAATAGTGCTCCACGCAGGCGGAGGCGCAGCCCGAAGTGAGCGGCCCGGAGGAGAGCAGGAGAGAGGAGCCCGGTGCG

>17818

TGCAGCTGGAACACCGAGTGTGAGCGGGAGGAGAGATCGTTCACGGCCGTCTGGGCCGTGGAGCGGTTCTCTTTGGCCAAAGCGATCAAACCAAGA

>42935

TGCAGCCTTTATAAGCGGCGTGCTGCGTGAAGGCTCACTGAGCCCAGCAGCATTCTCTGCTCGGACTCTGAACTGATACTCCATATCTTCTTTCAG

>133068

TGCAGTTCCAGGGAGCCCGGACGCACCTGGAGGGCCTCATCCCGTACACAGGTCAGCAGGCGGCAAAATGGATACAAAGTGTTACATCTGGAGTGA

>109632

TGCAGACAGACATAAATGAATCCACAGATGGTGGCTCTCAGGCAGTCACTCCTGGACTGTCTGTCTACGTGCCAGCGATGATAACGCACACACACA

>172864

TGCAGCATCAAACTAGGCGTCTGCTTAGCGCGTCTAAGCCAGCAGGGGACACCCGAGGATACTGATAGTCAAAATACAGGCCTCAAAACTGTAAAG

>137953

TGCAGCTCCACCGTGAACCCTGCTGAGCCTCCTTATTCCAGCAAATAAAGTTTTTGTTGTTTTTTGTCGTTTGACTCCCCGGTCGCCTGATTTATG

>248247

TGCAGCAGTCTGTCAGAAGCGCAGGCTGCACCTGTTACACAAAGACACTTGTTGAGATCCGATTTCATCCTTTAGTGTCTCTGGCAAGAGATTCTT

>248337

TGCAGTTCAGCCCGACGGTCCGGTCAGACGGTTCTGGACCGGTTCTGATTAGAGTCGGGTTCTGTCTCTGCCGCGGCAGGGCTGGATGAGGTCTGA

>32782

TGCAGAATATGACAGGGCCTTTACATGTGCTGTGTTTGTGTCAGCTGGTGTTGCTACAACTTTCTCCTATGTTAGTGTCATTATAGCGGCCAGGTC

>59171

TGCAGCAACAAAGCATTCAAAACAACACAGATTCTCCACTAACTCGGCTTCCATCCATCTTCTGAAGGCTGATCCTCCGGCGGATCTGTTCTTGGC

>30388

TGCAGGTGTTACCAGAGGGATATGAAAATACTTCCAAGTTTGGAGGGGATTGTCACAATTGAAATTGATATTCTCTTCCTGGTCACATTATTCTGT

>87444

TGCAGTCGAGGTCAGACGCGCTAACAGTCGGTTAGCTGCATATTTACTGCATGGAGATGAGCAGAAACACACACATGTTGGTTTTTATAGAAATCT

>83548

TGCAGCTGTGTGGGCTTCAAACGCAGCACATGGTAGCGTCCACCAGCTTCTTCTGGCGACGGAAACATATAGGCACTTAAGATCGGAAGAGCGGTT

>200369

TGCAGCTGCTTCACACCTACTGACCACATGGACCGACTGGAACCAATTCAGCAGCTAACAGGTGCTTCTGCCCTCTCACTCATCCACCGGGTCGTC

>246172

TGCAGACGTGAACTGTTGCATTTTTAGTTTTACAAACCACAGAATTTTACTGAGCAATTTGCCTAAGAAAACGCCTAAAACTGTGGAGATATCAGA

>190419

TGCAGCCATGAGGGGGAGCAAGCACATCTACCATCAGCTCAGTGTTTGAACGTCCAGCGTCCGTTCTACCTAGCATCACAATATCGGAAACGTCCA

>69623

TGCAGCTGTTTGGAGCATGTGGGATGTTCTTGTTATCGCATCAGAACAACATGTTGCTGATCCCACACAGCACTTTGCTGTCCAAGGATATGACTT

>177560

TGCAGCTGTAGTTCAGGTCCCATCCACTTCATCTTCCCTGCCCAACATGGCCGCTTTCTTGCATACATTTTCAACAAAAAGTCCCAGAAGCCTTTG

>13377

TGCAGGGGCTGGTTGGGGAGGTGTCTGTGGCTGTGCCTGGTCAGGAGGAGAAGACATGTTACGAAGCGTGGAAATGATTTCATTCATGCCGGTATT

>24923

TGCAGGAAAACATCCAACCACTGAAGCCACGCCCACCACTGAAGCCACGCCCATCGAAAGAGCCCCGCCTCCTCCCCCCTTCTACCTCCACATCAG

>178408

TGCAGAAACTCGAAACACAGGTGCCGATTGCTCAGCTGAGACGCTGGGCCAAGCTGCTCCAATCTGAAAACACACTATGCAAATCCCTATCTGCTG

>245243

TGCAGCATCGTGCTCAACTCCGTGGAACAGTACCAGTCTCACATCAGCGGCGCCAGGCATAAGAACCAGTAAGTCACGATTCAACAGACCATTTAA

>86359

TGCAGAACTGCTGGAGTGAACTCCTCATCCTCGACCATGTTTTCCGTCAGGTGGTACATGCAAAGGAGGGCTCAATCCTGTTGGTCACAGGCCAGC

>40430

TGCAGTTTTCACACTGATAACTCGGATGTGACTGAACTGAAAACTGATTGGAGGAAGAAAACTCATAATATCACCAGAGCAATTAGCAGCTGTGGG

>248111

TGCAGACCGAATCAGGACATCTCCCCGGTCCTCAGCTATCAGGCCCGGTTTCTGGATCAGACGTTCTTCATGGATCAGTGGAGCCACAGCTGGATG

>39982

TGCAGCGCAGCCTCCTCCAAGCTGCACCACATCGTCTGCTGCAAAGAAGGCTGCCATCTCTGTGAGAGGGAAGTGTGTGCCTCATGCAGAGGATCG

>225012

TGCAGTGTCTCCAGCTGCTAAACACTCTAAAGTGTTAGCAACAATTTGATCTCAAAGTAACAGTCTTTGTGTGAAAAAGCACACTTGCATTATTTA

>125853

TGCAGAAAGGATGAGCAAATTGGAAGAGCTGGGATGGAAAAACAGACAAGCTGACAAAAGGAATAGAGATAAGACAACGTACACACAGTTCCTGAC

>21161

TGCAGTGGAGGACAAACTACTGCTAATCGTGGCGCTAACTTTAGCTTAGCATGCTACATGCTGTTATAGCATTAGCTCCCGCTAAACACTGTGTTG

>212822

TGCAGAGGAAACTAGCTGAAGGTTCTGGTTCTGGTGGAGGTCCCACTGGTTCTGGGTCCAGTCTGTGTTCTTTGCTGGTTCCGTCAGTGCCACAGC

>167841

TGCAGAGGCCTGCTGTAGCAGCCTCTCCATTCCCGACATGAAGGAACTTACTGGAGGTTCTGCTGAGAAACTCCGGCTGCGCAGTATTTTGGATAT

>227597

TGCAGTCTTCAGCTGAAGCAGTAATGATTGCTATGCTTGAAGTGATCCCAGCGGAACAAACAAAAAAATATCCCTCACCACCTTTGTAGTTTTTTC

>191235

TGCAGATGAATCTGGAGCAGCGCGACCTGGTCACCACACAGCGTCTGAGTCCGTCTACGTCAGTTTTCTGAAGTTTTATGCATTTTGTCTCGCAAC

>42665

TGCAGCCGTCAATACTACAGTTGACCTGATAAAGATTCTGAAGACACTTGGTTGTTGTATGTTTTGAAGAGGATGCTTAGAGTTGTTATTCATTCT

>23307

TGCAGCTCCCACAGAGTCAGTGGCCGGAGCATCCAATTATAACATGCACCGACTTCGTTGAGGAGCAGCGTAGAGCAGCCACTTGCTTGGTCGTAA

>91510

TGCAGCAGCAGCGAGACGGGGAGGAGCAGGCAGATAGCGACCAAACGCCGCGGGACGAGGACGTCTGTGAATGTCGGATGACAACCGAGACAAAGA

>170680

TGCAGTGTCTATAAAACAGGGAACGGTTAGAAGTGCTGTACAGTCCGACAACAAAACTAGGAACTGCTGCTAAGTACACTGTGTATATATGTACAT

>251171

TGCAGCCATGTGGGCCGGTGTGACACACAACAGATGTGGTGTGGACTATGACCTTTACACGCCTGCATCAGATGTACGATAGTGGAAAAAAAAAAA

>210052

TGCAGTGGCAAAATATTCCACCAAGTAGCTGCATATTGGTCAGTATTAGTTCAGAACAGCTTCGTTATCTGGTTCAATAGCTTGATAATAGCAGGT

>44364

TGCAGAGGAAAGAAATGCAATATTCATTCAGACAAATGTCCGGCTCCATTTCTTTTCAGACCCTGAGAGGATGACACACTTCCTCTGACACAGCCG

>8817

TGCAGCTTCAAGTGCTTTGAACACTTTTGCCGTTGTTCTTTGCAGAACAGCTGAAGGTCCATCAGACTGGTCAGAGGTCAGAGCGTCTGGTAGTTT

>137597

TGCAGTTGTATCTCGTTGCCCTTCGGCGCGGCTATCAGCAGCTACGCTGATCCGTTTCTGCGTCTGTTTGCGCAGCTGTCACTCACGCTGTTTACG

>59626

TGCAGGAAAGCGCTCGTATTGCTAGGGGCGACGTGACAGATCTGGTCAAGCTGGATGTCTCAGCGTTTGATGCTGCCATCATTCCAGGTGAGTTCA

>5573

TGCAGCCGAGCCGCTTTCTCTGTCTGCGGACTGTCTGCTGGCGGCGGAACAACAGAACCTCCAGACCCCCACAGAGCCGCTGCGGACCGAACCATC

>34376

TGCAGCCGCTTCAGGTGAAACCCTTCGCTCCGTACCAGCGGTACCTCCAGCTCCCTTTCCAACACGGGCGACATGTTCAGACCTCGTCTCCGAGGC

>111229

TGCAGTCCTGGGGCAGCAGCGCTTCGAACGGACCCATTAGTTCAAACCAATCCATTAGAGCCAGGCAAAAACAAAACAAGATCCTCAAGAGGAGGC

>156846

TGCAGTGACCCTTGATCCTGAAAATTAGCCTGGTCTCACTCTGTGAGGTAGGAGCAGAGGCTCCTGGCTCTCTCTGGCTGCTCGTCTCCATCCAGA

>163459

TGCAGGGACTGAGGCACAAATGCGAGTCAGGCACAAAGGAGCAGAGGGTGGAAAGAGAAAGAAGGAAGACAGGAGAGGAGTTCGGTTGTCTGTATT

>40507

TGCAGGAGGTCACCAGTAAGTGTCACACTAACACTCCAACACTTGGCATCTATATAGGAAACTCAATTGGTTGGATAGAGTCTCACTGCTGCACTG

>181461

TGCAGTGTGTGTTTCCTGTGTGTCCTCAGGATGGCCAGTCATGGTCGAACTATCATCATGTCCATCCACCAGCCGCGCTACTCCATCTACCGGCTG

>8575

TGCAGCTGCACTCACTGGGGCTTCGGTTATGGTACGTCATGTTTGCAGACATAATGACAGAAGAACAGTTCCCTGTGTTTTTGTTGGGATCAGCCT

>137671

TGCAGAGGGATCGCTCCCGGCACCATCAACCATCCCATCCGCGCTACGGTGAGTGGAGCCTGAACGCAGCACCACGCCAGGACCAGAACCGGCAGC

>108848

TGCAGGCCATGTGGCAGACCATGTTTGTGGACCTGAAGAGCCTGCTGTCCTGGCAGTACCTGATGAGGGACGTCCACATCATCAACTCCTGGAACG

>257693

TGCAGCCACAGACGGGGTGTGACTGCTTGTGAAGCCATCTGCATAAAGACAACTGGAAGGGCAAGAGCAGAATTAGGGCTCCAACACGAAGAGGTG

>60595

TGCAGGGAGGGCGAGCCGGTGTCCCAGAACGGGCTGCCGCCCGACCAGGAGTCGCCCCGGGTTAGTTACCGCTCTCCTGCCTACCAGACATACAGC

>140263

TGCAGCAGACCCAACGGTCCACACAGAGATCTGGGACACCAGCAACCTGGTGAAACTGGATCTGGATGTTCGGACAGACCAGGTTGTGGAACTTCA

>207483

TGCAGACGAGGTGAATGAGACTGAAACCCTCTGCTTTCATCATCCAGACCTCCACTGACCTCCAACAGCTCTGGGTTTTATTTAGCGTCTCGTTGG

>328593

TGCAGCATCGTTCAAAGTTTTTCTGTTATGAAAGAGTGAGAGGATTGACTCATGGGAAACTTTTGTGTTTGTGAGGAGAAGAGTTGAATGGACTTG

>69882

TGCAGCACGAGCAGATGCAGCAGCAGCAGCAGCAGATGCAGCAGCAGCAGCAGCAGATCAGGCAGCAGATCCAGGAGATGCGGCAGCAGCAGCAGC

>94248

TGCAGCTCTGAAACAAACCCATCCTGGGAAGTGATTGAGAGTAATAATCACTCGGTTTCTGATGGCTTCTGTAACCAGAAACTCACCAGCAACATG

>104346

TGCAGTGAAGGTTTCATGTAACATGCAGTGAGTTTTTTCCAGCTGTAGTTTGTCCAGTATTTTTCTGAATCGTTCTGGTTCTGCCTGGTTCTGGTT

>98612

TGCAGCCCCAGTGGCTAGCTGCTGCCCAGGACGTCCTCCTGTTCTGGCCTCAGTGGCTGATTATCATCCTGCTGGAGTCGTTATGGCTGTGCGTCA

>96430

TGCAGACGCTCCAGCCGCCGCTGCTCCTTCTCCGCCTCGCGTTCCGACTGCTTCTTCAGCCAGTCCGCCATCCTACCGGAACCAGAACCAGAACCT

>230680

TGCAGCAGAATCATTTCTTATAAACACTAAATTAGTTTATTTTGCTCCATGTTTCTGTGTTTCCAGGTGACAGTGCTGAGCTACAGTGCAAAGTTT

>66553

TGCAGTCACCTTCTTTCTTACCGCCGCCACTGCCCCATCCACGGACCCCCCATGATCTTTGTTTTTACTCCTGGCCTCAAACGGCTTAGATTGCTG

>70031

TGCAGCGCGCGAGAGGCATGAAGGAGAAGTAAGGGGAGAATAAATATCAGACTGTGAATATTCCTGCAATAAACCAAGATACCTCACTTTGAAGCA

>6440

TGCAGCCTGATTCTCCCAGCATGCGCTTCATTCAGGATCTTCCTCCACGCAGCTTGCAGGCCATCAATGATTTACATGCGGAGTGTCTGAGTGGGC

>159614

TGCAGGTGTGAAAATCAAACAGAACTTCAGAACAAACCGAGAAATAAAACGGGAAACAAAAACTGAAAATCCAGCTGAGAAACTGAAAATAAATCA

>49378

TGCAGGTCAACGAGGCCTTCACACACGTGTACTCTGGGGGCAGGGACAAGAAGATTTACTGCACGGACCTGAGAAACCCGGACATCCGTGTGCTCA

>287917

TGCAGGCGACCGTGGTCCTCTGTCAGTCGCTGCGCTGCACGACGCGCTCGTCTGGCGACACTCTGCCGCGACTCAACCGGGTGTGGAAGCAGTTCA

>153105

TGCAGCGGGAAAAGTTGGGGAAACGTTTGGGGTTGAGGGTTTTATAATATTGGTGCCTGTAGCCAACCTGCCTGGGCTTGTGTGGCCGTTTGGCAT

>186198

TGCAGCTCCAGACTGCACATACAGAAGCATGTCTGTCTATACAGCTGACATCTACCAGGGCCAAGTTGGAGGAAAAAAGTGTCTCTGGGTTCATGG

>311102

TGCAGTGACGTAGATCTGAGCAAACTGAGCTGGCGCCATCTTGCCCTGGGCGCTCTCGCTGAGGTTGACGGCGCTCAGAGACAGAGACAAAGCCCA

>144185

TGCAGGTTGCCGTCATCAGTCAACCAGAACGCCATGGACTCTCCCAGATTCGCCTCGTCTCCCTCGTCTTCGTCTCCTGGGACGGACGCCCCTCGT

>91551

TGCAGCTGAGGCTGAGCTCGTTATTTACTCAAAGTGCTGTTGTTTGCAGTGCAACTCAAATTTGAAGTTCTCCTCGTTGGGGGAACAAGCAGGAGG

>223172

TGCAGGTGTTTGTGGTTCCTCATTCTCACAACGATCCAGGTGAGTTGTGCTTTTGAATGTCAGATATTTATTGGAGACTGGAAGCAGAACTTGCCA

>336136

TGCAGGGTTGCCAGTGAAATTTCATCACTGGCTTTCATGAATACAGATAAGAATACATATGACTTACCCATCGTAACTGTGCTGCCCGTTTTTAGT

>125696

TGCAGATTCAGAACTACATGGAACAAGGCGGAGGAGCCATGCCGCGGCTGCTCTCCTCTCTTCCAGCTGCCTGTCTTCAACGAGCCGTCCTCTTTC

>262852

TGCAGACTTGCGCTCATCAATGGCGGATTGGTGCGTAATTCAGCTGTGAGCGTATCGCTTCTGCTGCTTGAATGCACCATTTCCAGAGGAATAAAC

>249626

TGCAGAGACTCTGAGAGAAGATCCGGAGTGCTGTGTGTCCTTCTCATCAGTCAAACATTCAGGCTGCGTTCGGATCCATTTCTAGGGGATTAAGAT

>218525

TGCAGCAAAACGTTTCAGAACCAGAACCTGATCATCATTCTGGTCACAGACGAACCAGGAGGACATCAGAACCGTTTCGGTCCAACAGAACCGAGA

>35106

TGCAGCTGAAGCGTGAAAAGATGCGACAGGCACTGGAAGCCAAGTCTGGCTTTGGAAACGACAGCTTCTGCAACATCCGCACTCAGCAGCAGAAAG

>295113

TGCAGCAGAGAGGACAGTTCATGTGTTTCGGTTATGAACAGATCGGAGAACAGACCATTGAACTATAAATCCTAAGATAAAAAACATTATGACAGG

>118111

TGCAGCGTCTCTGACGGTCCAACATGGACGTTTGTTTCATAACTGAACACAAACCGTCTGTAGACAACATCAGCAACTTGGTACATTATTTATAAT

>133824

TGCAGTCTGTCAGAGCGTGGATCTCCTCCAAACTGTCCAGCACTTGAGACACAGCGCCTGCGAGGTCAGAGGTCACGAGACTCGTCAAGAGCTCGT

>122884

TGCAGCAGTTTGTATGGATTTTCTTGGGCTTTGTGATGTGCAGACCATCATCCGACTCGTGTCCTTTTTGACATGAACACAACTTCCTCGTGCCAC

>61430

TGCAGAATACCTTATGGATTGGCTTCTCGAGTTCTTGTGTAGAAGTGTCAACCAAGACTAAGCTCATTACAGCTAGATACCAGAGCTACTCTAACC

>79712

TGCAGCCAGCGGAGGGAGTCAGACGACCTGCTGACCAGGTAGCTTCCTCTTCATCATCAGCAGCAGGATGAAGAACCACCAGCAAACAAAAAACTG

>257135

TGCAGCTGGCTGAATCTGACTAGAAAGTTTCTATAAAAGTTTAGTCCATTCACCGAAAACTGGGGTTTGAAACAAAACAGCCGTGGCCGACTTGAC

>74270

TGCAGCGACCTTGGCTCACCTTGTAAGCTCCCCTGACACAGATTGCTGTGGCGGCAGCAGACAGTCGGCGTGAACTCATCAGATTTACTTACAGTC

>150296

TGCAGCAGAACCGAACACGCAGCGTCAGCCGGGTCCGAAACCCAGAAGAACTAAAACCTGGAACCGGATTCTCCTGACGAGTCAAACTTGAAAAGT

>249419

TGCAGCTCCAACTTTCTTCTTCTCTGAAGTGCAGCGGAGACGCAGCAAACCTGCGCCAAGATTGCATTGGAATCTTTATTATCATGTTTCCATCAT

>166538

TGCAGCCTTTGTCTGCGGCGAGGAAGGCCAAACAGAGAAATCTGAGCAAATTTCACAAACACTGAAGGACGCAGCCATTACTCATTCCATGCAGAA

>160277

TGCAGGGGAAGCGGCCCAGATGAGCGGACCCGGTGCCGCCGCTCCACCGGTGCTCCTCAGCGGGCTGTGGCACTGCGGGTCCGTCCAGAGGAACAG

>4140

TGCAGTACTGCGTGTCGCCGCTGGGTGTCGCTCTATCTCCATTTTATTCCTGCCTGTCTAACGGAGCAGATTAAGATCGGAAGAGCGGTTCAGCAG

>259948

TGCAGGACGGAAGAAACGATGAATTCCCGAGTGACAGGCAAAGCTGACCAGCTGTGATCTGCTTCACCGCAGGCAATGCTAGATTGTAAAAGGGGC

>176774

TGCAGGGATCCGATCTGGAAAACCGATCCGTCCGTTTTCATTCAGCTTCCAGACGGAGTTACGTTGCTGAACTCGGTTTCAGGGCTGCTGTTACCA

>234297

TGCAGTCTGACTAAACAACGCCTGGTTGTGACGGACAAGCCAATGTGCTTCCTGTAATCTTATGGATGCATGTTGAGCTGTAGCTTGAGCAGAGCG

>157502

TGCAGCCTGTGGTGGTTTCCTGTGCAATTTTAGAAGACCCCAAAGTATTTTGAGCAGTTGCCGTCATGATATGGAGCAGTACTAAAATACATTAGT

>319298

TGCAGCTCAGGATGAGTGTCCTATCGAGTCTGAGCTGGACCAGGAAGAGTTCTCGGGACAGACCTTCATCCATCACAGTAAGTTGATTTAAGATCG

>131735

TGCAGTCATACAGTATGTGTATTGTGCATAAGATTACCTGTACTGTCCATCCATCCATCCATCCATCCATCCATCCATCCATCCATCCATCCATCC

>11006

TGCAGCAAAGACTCCCTCAGAGGCAAAACACAACTCTTCCCTCAACTCCAGCTCATTCCAAGATGCACAGTGTTTGTCCACGTTCGCTTTCCAAAC

>69187

TGCAGTAATTCCTGTTTATAACTTTTCCTCTACACCTTTTCCTGCAACTCAACTTTTCAGGAATGGATTTTCATGCAATACTCTGCAAAGAACAAG

>63457

TGCAGGTCAGACTAACCAAAGAAAACACAGGTGGGTTATGCATCGTTCAGTTCCTCCAGGATGTTTCATGCAATAGCTTCAGCCCAAAGTTACTGA

>2613

TGCAGAGTCTGGGATGCATCACGTGGACAAGCAGAAACCCGTAAAGCTAAACATGGACAAATGTATCAACATGGTCTCTTTTATTTCAATCAATCA

>296267

TGCAGGCAGCAGAAAGTCCTGGTTGCTTTTGTTGTTGCTCAAGATGAATCCTCATTCAGTCAATGAGCGCCGAACGACATCGTTCAGAAAAGAAAA

>323103

TGCAGATGAATTTCTGCCTCATCAGCCCGAGAACTACTTGCGCGTCACCGACGAAATCTCGAGTCTGGAGCAGTTAGTGCCGCGCGTAGAGTCTTC

>118664

TGCAGACATCATGTCTAAAGCTCTCATCTCTCCCACTGGGTCTCCTGGTTTGTTGGGACGTCGTCTTGCTGAAAGTCTTCAGTCTGAGCTGCAGAG

>173244

TGCAGTGCCGTATATCCAGTCCCCAGAGAAAAACAAGCAGTCAGAGCAGAAAGTCTGACTGGATGTGACAGAACTACAGAGTTATGAGCATAAATA

>47648

TGCAGCCTGATGAGGACGTTTCCTGACGGCCGGCTGCTTTCTGACGCTGAAGCCAGAAACATGCAGCCTGCATGAGGCTGGCTGGACGCTGGCTGG

>279076

TGCAGAGATTCATACTGAAAATCCATCTTCTCCTCCAGATCAGGTCTCACTCTAGAACCAAACTTCTTGGTGTGAGTGGCGAGGTTCTTCTCTCCA

>137751

TGCAGATCCAGGCGCCCCGGGAGAAACCGCCAAGTGACGGGAAGGAACGGATTGTCCCCATCAACGTCACCTCTGCCCCAGCCATCACCTCAGAGA

>265853

TGCAGGGCGGAAAGGGGGGAAAAATGGATTCCATACCAGGAAATCCATTAGGGCAGGGAGCGGCGTGGAGCTGGCGGTACAGAGTGGCGGCCGCGT

>134693

TGCAGTTTGTGTTTATGTTTCATCAGTGTTACAGCTTGGTGATGACGGTGTTATTTACAGCAGCTGCTCCACCACCACCTCCTGCTTGTCCTTCTT

>105660

TGCAGAGCTCCATGGATTGTACTTCGGGTTCCTGACCTGCTCCATGGTCAGCCGAGGCTCCAAAGCTCATATTTGAGCAGGCGCTGTGTAAACACG

>198123

TGCAGATCAACGGCGGTGGTAGTTTTCCACCGTAGGGGCAGGGTGCAGCAGAGGGAAATGTACTCGTATTTGGACAGTCGGGAGGGGGAAATGAGC

>2976

TGCAGAAAAATATGTGTTCTGTTCAGAAGTGAAGACAGCATGTTCGTACCTGTCATGGTTTTTTCAAGTTTATATTTCAAGTGCCATAACTTTGTA

>282914

TGCAGGGCGAGCAGCTCGTCTTCCTGCTCCTCCAAGTCCGTATTCATGAAGTCCAGAGACGCTAAATTGTTCAGCCGCCGCTCGAAGTGACAGCGA

>266769

TGCAGTAAAAATGGAAACTGATCGGATTCAAAGAGTGCATTCAGTCCGTCAACCCTGAACATTCGAGACGGGAACAAAAACTTTTATAGAGCGTAG

>198607

TGCAGCGTTTTATGCCAGAGCACAGCAGTAAACTCCTGGACTCTTGTCCTTTCAGCTCCATAAAGCAGAGAAACGGCTGATGAAGAGTCTGGATCA

>240451

TGCAGCAGGGCCATGTAGGGGAAGAAGCGTGAACAGAAGGGTAAAGCTTCATGGTAGCACACCTAGATGGACAAAACATTTCCATTAGGTGAGGCA

>73317

TGCAGCGGTTTGCGTCCAACATCACCCGTCTGGTTTTGTAGAGCCAGTGAGGGTTCTACAGGTTTCACTGTGGGCGTCCAACCAAGGAAATGGTGC

>208381

TGCAGTGACCGTGGCCAGTCTGGAGGAGGCAGGCAGGTGGAAAAGATACGTCAGACTGCGAGGGTTGTCGTCCTCTTTGTCTTCCTCAGAGGACGG

>323074

TGCAGTTAGTTGGTTCCAGTCTGAACCAATAGCAGCATCCGACCTGTCAGTGATGTCACGTTTGATTGGTGCTCAGCTCGAGGCCAGCGGTCTGTC

>84891

TGCAGGGACGTGATCAGGAAGTGATGTATGGAGTGCCATTAGTGCGTTTACATCATCAGAAACAGCAGCAGTGGTTATTTAGGAGCAAAATGGAGG

>8429

TGCAGCACCTGATGGTGTGAGGCGAGAGCAGGAGGCAGACAGAGGAGAGGGAGAGAGAAACTGGTACAGGGGGCAAGAGAGAGAACACCGGGGAGC

>158431

TGCAGACGCGTCAGGAATGTTTTCTTTTTTCTCCACTGGTTCCGACATCTCCAAAAAACCTCCCAAACAACTTTCCCTAAACTTCTCCTCGGATTC

>257059

TGCAGCGCCCTCTGCTGTTTGGGTGAAACTCCCAGGAGCACTGGGACAGTCAGGACCCATCAATTTAGAAAAAGTCTAAATTTGTTCCCCCCAAAT

>2014

TGCAGTGAGCCTCACCCATGCCACAGCCAATCATATCACAGCTCAAAGAGAGGGGAGAACGACAGGGGTGAAAGCCATGCTGAGGATGGAGATTGG

>159219

TGCAGTGGTTCGGAGACGGAAAGTTCTCCGAGGTGAAAGCCGTCTTGAAGAAAAGAGCAGGAAAAGCTGTTTATCTTGGGTTTTGAACCTTTCCAT

>133119

TGCAGCAGGTTTTTCTCTGGGCGTTCAGGTCCATCAGTCTCCTCCGGCTCTCCTGGGAAGAGAAGGACAAAGCGTGCAGCTGGTGTGCAGCCATGA

>228901

TGCAGCTCATAAGTACTGAACACTCTGATCACCATGAAACATGGAAGCTGTCAGACTGAAACACAGTTTCTTCATGTTATCAGTCCTTCTGCTCTG

>311655

TGCAGTGCGAAAACGCCTTTAGTTAGTGCATGTGGATCAGTTCAGAATCGCTTTCTGTATCATGTGAGGCACTGTTTGATCCATCAACTAGAGCAG

>271105

TGCAGCTCGTCACTCAAGGCGTATCTGAACCTGAAGCAGGTGAACCTGGAGCGCTGCTGGACCTCCTCTTTCACAGCATGTCCATCGCTCAAGTGC

>72251

TGCAGGTGGAGCGCCAAACATCTCCAACATGCTGGAGCTGTTAGTAGAGCTGCTGCTGCTGGAGTGACATGGAAATGGTTAGAAATGGTCCAATAT

>56960

TGCAGGTACAGCTCGTCCTGCTTGTCTCCAAGGATGAAATGGTAGCGAGGCAGGGGCCGTCCAGGTCCAGGCAGGGCCAGCGGAAGTCTCAGCAGA

>19390

TGCAGAGCGATCGCTGCTTTCCCCTCGTTTGGAGCCTGGATTCCGAAATGTCCTCCAGTCAGAAAACCAGGTCATTTCTGAGAGTCGCCTGGGAGC

>149720

TGCAGGGAAAAGTGGAGGCAGAGTTCCACCTGGTGACGACTGAAGAGGCTGAAAAGAATCCTGTTGGCAAAGCGCGGAAGGAGCCGGAGCCACTGC

>133430

TGCAGCACTCCCGTGGATTTCCAGTAACCCTGCCGACTGTGTGCTGGAAGAGGCGTGTTCGAAAAATCACCATCTTTATATCTTTCTCTAAAACTG

>231547

TGCAGGAGCACTATAATGATATAATGTTTTATATTATAGCTACTCATTGTGAGGCTGTGAGATGTTGAGTCAGAATAAATCCCAGAACAACAAAAT

>263892

TGCAGCCAACCTCATGAAGAGCTACTGGCTTGGAGTGAACAGCAGTGAAGAGTGTGGTGGGCAGTGGTGGGGAGTAACTGTACTGTACTTAAGATC

>271873

TGCAGAAAGCGAAATCTCAAATCTGTTTTCCATTCAGTCTTTTTGTTTGGTTATGAGCTTTATTATGTTCCCCCACCAAACACACACACACACACA

>109760

TGCAGAAGGAAGATGTTGTTGTAGTTTCTGATAAAACCAGTGGTGTCCTTTCAGTGGCCGGCAATGAGAATGATGTCAACAGACTCGAGAAACCTA

>165304

TGCAGACTTACACTCAGGGAATCCTTCCTGCCGCCTACTCCAGGCAGTTCTCCCCGAGCTTCAGCCTGGAGGGCGGACAGATGGAGCAGCAGAGGT

>230781

TGCAGGGAGTTTGGATCAGAACCAGGTTTCAGTAACGACCCAAAACCTCCGACCTTCTGCAATACGATGGACAACAAACCGAACAAGATCTGCGTT

>260236

TGCAGGGCTGGAGAGGAGGAGATTAGGTGAGGTGGACAGAGGGAGGCATGGTCAGGATGGGGAGATAGAGAGATTAGAGAGATAGAGCGATAAGAC

>105550

TGCAGCACAAATCAACACCTCACCTGTCTGAGGCACTTCACCTGGTAAGACTTCACTTACTTGTTTTGATACTAACAAGAGCTAAACCTTTGTCAC

>335835

TGCAGTTTCTGTCCTCTGAGTTCAGCAGGCAGCGCCGGCTGGGAGCCGCAGATTAGGATCCAGCAGAAGGCAGGAAAAGGGAAAGTGGGCGAGATA

>12452

TGCAGGTGGGTTTCCCTACGAGCTGACAGAGGGCGACCTCATCTGCGTCTTTTCCCAGTAAGTCCCGCTCGCTCTGACCTCCAGAGGTCGTCCTGT

>303913

TGCAGCTGTTCCACTGGGTGCCAACATGGCCGCTCTGTCAGTCCTGCTCTATCAAGGTGTTTCTCTTTCAGCCTTGTTCCTCCTTCAGGGTTCAGA

>189899

TGCAGACGGAGGTCGCACGAGATGAAGCAGTCAGAAAGAGGAGCTAAAAGTGAATGATCACAACTTATCAGGTGTCAAAGAGGCAGCAGGAAGCAT

>17004

TGCAGGTGCTGAAAGAGAATCATTGATCCGGGAAGTCTGAAGTCTTTCCAAGTCTGGAAGGTTTTCACGGCAGCCAGTCCTCTGTTGTGCAGGCAG

>315104

TGCAGAGGAGTAAGTGGGAAGTAACTGGACTCACCTGTGGGGAAGGAGATAAACGGGAACAGGAGCAGAGACGGAGGAAGTTAAGATCGGAAGAGC

>189482

TGCAGGAAAGACTAAATTTGGCCCCTGTCTTATGAAAAGTTCCTAAAAGTGACTTTTACAAACAAGCTTGAGGTCTCGGGCCCCTGAGTGCCGCGT

>87506

TGCAGGGAGCCACAGAAAAACCTGCTCAGGTACCAGAACCGGGTCTGGTACCTGAGTTCTGGTACACAGGTCCATGTGTTCTACCATGGACCTGGT

>37142

TGCAGATATTTCATAAAAGCATTCAGAAAAGTACAAAACAGCCACACATGACTTTGTAGCTGCCGTTTGCAGACCTCTGTTGTAGACGACCCCATG

>210308

TGCAGAGAGCTCAACACGGCCGTGTTTCATTTGTGCAGAATCGCTTTGACAAAGCTGGACATCTTGGACACCTTGCCAGAGATTAAGATCGGAAGA

>79822

TGCAGGTGGCCTGTGGCGGCAGCCACACGGTGGCGCTAACAGGTTAGCCATCACACACCGTCTGGCGAAGAATAAGAGCTTTAGCGACGAACCTGC

>97060

TGCAGAGCGCCCCACCCAGACACAATGCAGCTCTGACCAGGCTCAAAGACGTGGGACGGCGGGGGAATGCAGACAGGCTGGATGTAGGAGTTGAAG

>78207

TGCAGCAGGTTCTGAAACTCAACCTCCTGAGAAACGGACCTCAGGGTTCTGCTGCTTTTCTTAGAGAAACTTGTTCCTGTTTGTAGAGGAACTGAA

>316809

TGCAGTGACGACACATATTTATGTTGCATATATGGAAAAAACTACATGTGAGGTTGAAAACTAACAGTGCACACCAATAAAAGTATGAACCATGGT

>84974

TGCAGAACCAGCTGAACCAGGAGAAGAATGACATGCAGATGAAGAGCGAGGAGCTCAACATCCTCATCAGGTCTCTCCTCAGGACCGATGGACCTT

>202218

TGCAGGACGATCAGTGAAGGTCAGCTCCCTGCCAGGCTAATGCTAACAGTTGTTAGCTTTCATTTTATGGCCCATTTTCAGGAACAAGGCCGTTTA

>5106

TGCAGAGGCTGGTCTCAGGAACCCTCACTAAAGGTACGGTGGCTTTTGGCTTATGCACTTCCAAATTTCTCAACGTTTTGTTTATGCAGTCAGTCT

>214491

TGCAGGCTGACGTTGTCTCCTTCCGGTTGTTTCTCAGGGTGTGACTCTCCTCCTCAGGCTGTGTGTGAGGGCTGCTCGCGCATCATCTTTGACCGC

>293021

TGCAGGCCTTTGGGGGCATGCCACTAACAAATAACTACAGCTCAACCATTGGGATGGAGAGCTCCCGGAAGACAGCAGATTCCAAGTTTTGACTCA

>41324

TGCAGACGACCCTGTAACCAGAATCTGTTGTGACGGCTCTGATCCTCTGACTTGATTTACTGTAAACACCCTTAGTCTGCAAATGCTGATTTACTG

>203533

TGCAGAAATGAGTGGGTTGGGAAAGACTCGAGACTTTCTCTCTGCCCCCCTTTTGCTTTTCTTCTTTTCCTTCTCTTTCGTCTTCTTCTTCTTCTC

>32001

TGCAGCCTGCTCTGGTGGTAAGGTGGGCGGTGGTAGATACAGAGAGGGAGGGGAGGCTGGAGGGACAGACATCGAGGGTTGAACCGCGAAACTTGT

>20221

TGCAGGGCTCCAACTGAAAGCCCTGACAGTCCCTTCTCCCGCATCTCAGCTCGCTGTTACCTGAACCGGGTGACCTCAATCAAAACTTCAGTAGTC

>93235

TGCAGTGACCCGCGCCCCGCAGCGTGCCAGAGCCAGAGCCGTGGCCCTGCCGATACCTGAAACACAAAACATAGATCATCATCCAAACTGAAGCTG

>306105

TGCAGCAGCGACTGGCCGTAGCTCATGTCGGACTCGGCGCTGGAAGAGGAGAAGGCGGACGAGGACGACGGCGTCAGCTCCTCCAGCCGGAAGAAG

>143926

TGCAGCTTCTCCGCCGGGCCAGACCGATTCTGATCCTCCGGCAACATCAACCTCCACAAGGCCAGTCAGAGGACTTGCCCTGCTGCCATTTGCTCC

>177332

TGCAGCAGCACACCACAACGGGCTGTCGATGAACCAAGCCCTTCTCGGTCTGTCCCCCAACATTGCCCCTGGGCCTAAAGAGGGTGACCTGGCTCA

>124810

TGCAGGTGGAGGTCAGCATCACTTGAACATTCATGATGAACTCAATCATGCATTGATCTGTTTTTGGCATCAAACACTGGAGGGACTCACGATGCC

>36978

TGCAGGTGGGAGGCAGAACGGCTATTCATCACGAACGATGGGACGCGGAGCAGCCGACTACGACAGCTGTTCATCCTTCATGAGCAGCGAGCTGGA

>233180

TGCAGCTTCTGAAACTACTTGACAGAACATTTCATTTGAAGAATATGTGAATTTGTGAGAAGAGGGGAAAAAATACACCAAAACACACACATGCAT

>116706

TGCAGAGGAAGCACAAGTGCGGGACAGCTGTCAGGGGAACCACCTCTGTTACATAAGGCCCACTCTGGCACAGCGACGGTGGGATGGTTGTAAATG

>137747

TGCAGATCATTGATCCGGGTCGTACTACGTCTGAACACGAAGCTCCTGGAGTGAGCAGCAGCTTGGAGACATTGGAAATGGCAGAACAGAACCTTC

>191025

TGCAGAAGCTTTGGCTCCGCAGGCTCATAGTAACACATTTTTAGGGAACAACAAGTTACAGAGGACAGAGCAGCCATTCAGAGATGAGATTATAAT

>98613

TGCAGTGTCCGGGCGGGCCGAGCGGCGCGCTCAGTGCGCACCAGACGTCCTCTGCTCTGCACCGGGATGGAGGGGCGTGTTGTGCTGCACTCACCT

>170502

TGCAGTTACATTCTGGACAGCAGCTCAATGGTGAAGGCGTCCTCTCTCTCATTTGAACTGAAGCCTCTTCAGGAAACCATTGATGAGGTGAGAAAA

>195702

TGCAGGACGGCCGGACGGCCGGCGGACGACCACCGGCCGGTTCACCCAGGTGAGAGCTGGTGAGGCTGATGCCATCCAGCAGGAACTTAGTCTGCC

>110404

TGCAGACTTCAACTGGAAACACGGATACCCAGCTGACAAAACACGGGACACGTCAGGGTTTTCCAAGAGCTGAATGTCGCTGGTCTCTTGTGGACT

>136644

TGCAGCGAGAGACTCTGAGCTCATACGCCAACCAGCAGAACCACAATTCGGAGCACTGGTTCGCAAAATGCATGTCATCATTAAGATCGGAAGAGC

>31930

TGCAGGCATCATCCAATCACTGCAATGACATCATCCATCCATCCATCCATCCATCCATCCATCCATCCATCCATCCATCCATCCATCCATCCATCC

>325434

TGCAGCTGTGTTTATGTCCCCCCCTGCTGGACGATTTCTCCACTTTTATACAAAATAAAACTTCTGATATTTTTCTCTATGCGCCTCTGAGTCTCA

>333794

TGCAGGATAGTAATCTATCACAGAGTCGCACCAAATGTCACTGTGCTAAATTTACAGTCATGCTTATTGTCACTCTAAGCCATGTGTGTAAAACAT

>193263

TGCAGTTTGGAGGCTTGATGACACGTTTATCTGGAAATGTCACAACTACAGCAGCTGCGTTTGGGGATCTTCCTCCTCCAGGAGGGAAAACCTGCG

>321558

TGCAGCAGCTTCTATACGAACCGATTCTATAGCCGCTGCTGTTCCCACTGAACACCTGACTGCACCAGACGTGTTCATTCACAAACTGCTGCAGT

>303520

TGCAGCACTGCGGGGAAGGCGGCAACAGCAGAGCCGCGTCGGACGGGTTGGAGTGAGACGGCCCGGTGCCCTCGGGGCCCGACTGAGGAGCAGCGG

>195971

TGCAGCAGCCAGCCAATGACTGCTCTCAGATTAGGCAGGAAGTCAATCCAATAGAGTTTTTACCTTGCCTGCACAAATCTACAAATCTTACTGATC

>316748

TGCAGGTGAACATCGACCAATCACAACCAGCCGCCGTCCTAACGACCCTGTGAGGTCATCAGGGAGTCACAGCTGGATAGGAACTGGTTACATTTA

>241113

TGCAGAAATAAAACCGCTTTGTGGAGACAGAAAACAAAACATGTTCAAAAAGGGGGGGAAGGTGAAGGGAGACGAGAGCAGAGGAGGAACGGAGTC

>143978

TGCAGGTCAGACCATCGATTCAGACTTTCAAAATAAAAGCATCCATTTCCTTCAGGTGAAGCTACTCTTTGCTCAGTTTGCCTCCATTTGGCATCA

>11532

TGCAGTAAAGATACCAGAATAATCCGAACAGTTTGGGGAGTGGAATCATGGAACTGAGACTCTCAGAAATCTTTACTTGGATCATAATTTGTTATT

>139028

TGCAGGCCCCCCAGACCAGGAGGAACCCCGGAGTCTCCTCGGAGGACGCCCGCCGCCCCCCAGCCCTCCAACCCGGCGCTACAGGCCCAGATCAAA

>50510

TGCAGGGAGGATCCAGGTTACAAACTGGTTCAGCCTGGAATGTTGTGGTTCACCATCGCTCCCAGTTCCAGGAGCAAACTGGATTTTCAGAGGAAA

>12451

TGCAGACTTTGTATGCACCTGTATGGCTGAGTGACCGCAGCTCAGGATGGAAAAAGCCTTGCTTCAGCTCTCGGTTGCTCCAAGCACCCATCTGTT

>166209

TGCAGGTTCGGGTTGGATCTCGGCCGGGCGGCCGGCGGGCGGGCCAGCCTTCCCGGCCTGGCCGCGCAGCCCGAACCGGACAATGGACAAAGCGGC

>135253

TGCAGGGTGAGGCCCTACACACAGAAATGTCTTCACCGTTGCTGCTTCATCCCACAACACAGATTGCCCTTCATGATGTGTCCAAAATCCACTTTC

>73542

TGCAGCTCATGGGGTCAGGTCAGCGGGAGGAGCATCATCACCACTGTGTGTCCTTCTGTGACGGGTGTCACTTACAGCTCGTCCTGAACTTGTTAT

>141845

TGCAGGTTCTGCTGCATCAGTTTCTGGGTTGAAAACAAACAGTCTGCACCCCATCCCATAATAACCACACCTAACAGCCCCAGCAGCTGCAATCAG

>276518

TGCAGTGCCTTTCCCTCCGGCTGCTCGGACCGCCTCTTTGCTTGTTTTCCGGCGACGGGAGGAGGAGGGGAAATGAGGCGGCATCATGCTTTAAGA

>29760

TGCAGGTGGAAGCTGTGGGACGTTTCAGACCCGGGTTCTGAGGACCAGAACCAGTTTGGAGCAGACGGAGCGGCTGCTTCTGGACACTCGCTCAGA

>77763

TGCAGCTGAAGTGTAGTTCTGTTTTGTTTTGTGGTGTATGTCTCCTTACTGTACATTCTGTATCACTTGTTGAAGTTATTGTTGTGCTGATGTTTG

>163246

TGCAGCCGGGATTGGCCGAGCTGCTGCCTCCACCTGCTGCGTCCTGCGCTGCTCCTCCTCCTCCTCCTGCCGCCGCGGCGCGGCCCTCCTTCAGCA

>204588

TGCAGCTTTTTAGCTGAACGTCCTCTACAAACCGTAGGCATGAAACTCCTCCTTCTCCTCGGCAGCTAGCACCAGCCTCTGCACGGAACACCGTCC

>204924

TGCAGGCTGACTGACTGCATGACCAGAGAAACCAGCCAGACCAGCAGGGACGGTTCACTGGGAATGCTGGGCTTCTTATTATTTAAGATCGGAAGA

>149369

TGCAGCCGTCCACCCAGAAAAGACCCCGGAGACAAAACGTCTCTGGTTGAGCGTCAGAACAAGAAAAGCAGCTGAAACTAATCAGATGTTGAGGAA

>234572

TGCAGAACCAGAACCGGAACCAGAGCTTTCTCATTCTGGACCCATCAGTTGATGACATCAGAACTTCAAGAGTTTCCGGACAGCTGGCCGATCTGA

>6894

TGCAGGTCATCAGCCGACTTCCCCAGACTTTGACTAGACACGTATTCATAACGATGTTCGTCGTACCTCAACAAACGACACAAACGCAGCATCTGT

>221861

TGCAGGAACGCTCCCGGTCAAAAACACGTTCAGGGTAAAGTCGACTCACCCTTCGTCAGGTTGCTCCTTCACTAATTTTCATCCCCATTTAAGATC

>125163

TGCAGATACTGTGATGTAACAGTTCAAAACAAACATAATACAGAGGAACCTGAAAGGGTTGAAAAACCTGCGCATGTACTCAGCAGCAGGAGAAAC

>190410

TGCAGCAGACACTTTGTCACGCTCAACCAGACACTCACTTATTTGGTCATGCAACATAAACACATGTATACAAACACAAGATCACTCAGGAAGCAT

>311705

TGCAGCCAATTCTCAGTCTGAGGAAGAGGAGGCTGCAACGAGCTCTCAAGCCGAGGATGAAGATGTGGAGCAATCTCAGACTGAGGAAGAGGACGG

>195022

TGCAGATCAGATCCATGCAGACATCTTCACGCTCGCAGCTGGGAGCAATTCACAGCGAGCGCTTCACTTTCTCCACGTCGCGTCACAGCCTCCAAA

>65968

TGCAGTACACTACACCTGGCGAACCATTACTGAAAAGCATCCAGGCTTTGCTCTATAGTTTGAGCTCTGCTTTCTCCTTTGCCCTCCAAGAAGACA

>284486

TGCAGTCAGACGAGCGCCGACTGGTTCCGACCAGAAACTGGATGACAGAACAAATCGGACCAGAACCGCTGAGGCTGTTCAAACCCTCAATGTGG

>88506

TGCAGGCCGGGTCCATGGTGGCAGTGGGACTGGCTCTGGCAGCTGCTGGGTTTGCAGGTAAACCGTGGAGCCCAGCAACTCTAAACAGTTTTATGT

>26360

TGCAGCAGAACCCAGAAGTATTCACACCCTTAGGTAACATGGCTTCAACTGAAACACGCTGAAAAGTGTGGCTGGCTTTTAGCCTCTCTCACTTCA

>57360

TGCAGTCTCATCGTTGGCTTCATTTTGACTGCTTGTATGTTTTTTTTACATGTAGCTGTGTGCTTATACTGCCTCAGGTTGGTGCATCGCAGCATT

>210953

TGCAGCAATCTGTGTCGTCATGCTTTGCACTGTCCCATTTTTATATGCACAGCCAGCAAAATTGTACTTTATGCCACACAGATTATTTTATTGATC

>229199

TGCAGGTTTCTCCAGGCAGCTCTCTGTCGGAGCAGGACCGTCTGAACCTGGCGCTGCTCTGCTCTCAGGGTTTGGGTCGGCTGCTGGGCGTCTGGC

>60149

TGCAGCCTTTTGTTGTCCCATTTAGAGATAAATGAACAGATTTCACCTGTTATTGTGTAAAACTGGAGTTCATCGCTCTGTGACCCGTTTAGTTTC

>146977

TGCAGCCAGAGCTTTGTGCCGGTGGCAGCAATGCGGTGGGCCATGGGTGCTGAAGCCAGCAGAGGGGAAGAGGAAGAGGAGGAAGAGGAAGGTTGC

>83590

TGCAGCAGAGTTTGAAGTCGACGGAGGTAGTCAGATGCCTTCTCCCCACTGTTCTGATGTGTCTCTAGAAACTGAATATACAGCTCCTCCCCTCCT

>4735

TGCAGAACATAAACTGTCCTATGAGGAACCTTCTGCATCTCATCAATAATCAGTGAATCAGTAAATGGTCAAACGTTCACAAAGGGATTGCTGCTA

>201547

TGCAGATGTGGAAATGCGCGTAAAAAGCGCCTCCAGTCTGCATCAGCGGCCACTGGATCAGCGATCCGGGCCCAAGACGCTCTAATTACAATACAA

>244512

TGCAGTGACAGGAAGGTTTGTTGATGGGGAGATGAGTGGAGCTTGCACAATAACAGAGTAAGAAAACCTGAGAGGCTGCAAAAAAATAAAATAAAA

>263025

TGCAGGGCTTCACAGAAATCAGCAAGCCAGTGGGGAAAATGTACATTTTTATATGGAGCATGTTGGTGTTTTGTTTTAGTCCACTTCAGTCCAACT

>134901

TGCAGCAGGAGTCATGGGTTCCTCCAGGAGAACCAGGAGTCAGCGCTCCTCCTCCTGGAGGCAGAATCCCAGCAGGAGGAGAAGGAGTCAGTCTCT

>170673

TGCAGCTAATGATTATTTCAAACTGCGATCCTGACAGAATCATGAAGGAAAAGAGCTGCGACAGGATCGAGTTTTACTGGACAGAGCTTTTTATTT

>303191

TGCAGAAATCACTAGAGAAAAACTGAAAAACAAGAGGTGGGAAAGAAAAGGAAAAGAAGGAAGTAAGAATGGAGCAGAGAAAGAAGTGCAAAGAAA

>185469

TGCAGATGTGACATGGCACAACAAGAGTTTCAGACAGGGCATGACGTACCAGGTACTAATCTGCTTCTCCGTTACTCGGGAGACCGTTAAGATCGG

>165914

TGCAGTGCTGACTTTGACGGCCTCCTTTCTCAGCGTCCCTTGATTGCAGAACTGAATGTCTTGCTGCATCTTTGTAGAATGTATCATTTAGTGGAG

>23972

TGCAGAGATGTTGGTGCGTCTCTGCCGGCTTTAGTTCTTCTTATTAGACTCGGATTTTCCTCCAATCCACAGAGAGATCACCAGACTTTCTCCTTT

>3

TGCAGGTCCGACGGAGCCGGTGCCGGCCCCTCTGGTGCGTTCTGGCTGTCTTTGACTACGTTCTGATTGGACCCTGTTCTAACAGTCAGACTGCAG

>300175

TGCAGGCTGGTCTTAGAGGGAGGGCAGAGCTGCCAGTAGCTGCTCTCTGGGGAAACACAAACAGGAAGTGATTCTACCGAGATGAATTTTATTTTA

>96404

TGCAGGGAGATTTTGTGTCCGACGGCTTCCACAGAACCAGAGCCGTGTTGTTGTAGGTCTGAGCTACTTCAGGGGTTCCGGGTCGCTTTGGAACAG

>249545

TGCAGCAGAATGAAGCTCCTGACAGGGAGAGATCGGCTCACGTTTCCTGCTCTGCCTGGACCCAGTACACCCAGCACACCCAGCACCGGTACCGGT

>53725

TGCAGGCCTGGATAAAATCATACAGACAGACTAAAGAACGTGAAGCAGAACAGCCTGATCTACACAGATTTACAAATGACGCTGTATGTGCTGTTC

>335626

TGCAGTGATGTATGAAGAGCATGTATGCTCCTCATCTACCATAAAGCATTTCAGCTCTGCTGTGACCACTCATGATTTTCTTGAATGGTGGAAGTG

>111571

TGCAGCCTCCGGCCAACCTCCTCCATCCTCAGGAGGAGGCTGGTCACGTGGGCTACGGCCCGGTACAGGGTCTCCTCCTCTGGGTCGCCGTGGAAG

>61234

TGCAGCGCTGCACAACACAACCCACATTTCTTCACGTCGTTCTGCGTCGTCTTGAATAGATTTCGGTCTTTGTCTTCATGCGGCTGCACATGAAGT

>200148

TGCAGATGAATCTGACCCCCAGCTTGCTGTCCTAACCTGACCTTTGACCTTCAGCTAAAGAATAAAAACGATGCAGAGGAAGAAAAACCTCTGATG

>141163

TGCAGCCTGTCGTTGTTTCCTGACGGATCGACGCTTTGCAGCGTTCAACGCCTGGACAGACGGTTCAGGGTTTATGAATCTGTTGCATCAGCAGGT

>33707

TGCAGCACAATCTAATCTGAGCAGTTTCAGCGCCCTACGATTGGCTAAAAAGCGTCGGTGCTCTCCCTCCCCAGTACTTCGTAGCTACAAGCAGTC

>135987

TGCAGCTGCTCCTGATATGTCCTCACTGCACTGCATGGCGGTGGGAGCGCATGTTGGGCTCTGACTGTGGGACGGAAAGCTACAGTAGTGTGGCCC

>90706

TGCAGACAAACACGCCCGGCGAGCGGGGGAGCCGGTGTAAGCGGATAAACGACGGGTCGCTTGGGCCGGAGGAATAAAAGCGGCTCTGTGGATTGC

>32172

TGCAGTGTGTGGCCCTGAGAGGAGCACCGAGCTCATCAGCGGACTATTTCTGGAAACTAGCTCATTAGCCTCAAGTATTTAGACAAAGAAAAGAGG

>246032

TGCAGTCAGTGCATTTCCCGACACATTTTCACATCGGTATGCAGCGTTTCTAAAGTTTTTGGAGTCATTTTCTTCAGTTTATAAATCCACACTGGT

>441

TGCAGAAAGGCACAAAACAACATGCGACCAAACCTCCTCCCTTCAAAAAATAAACAACTTACTGGAGTCAAATGGAGTCCCTGTTGGATGCGCTGC

>127987

TGCAGTGAGGGTCAGTGGTTTGCAGTTGCTGGGATGCAAGCAAAGCAAAGCAAGAGGACGATTTTTTAGTGAGGTGGGGAAAAAATGCTGAAGGCT

>184659

TGCAGGAGCTGCTGGAAACATGTGCTAAGCCTCAAACATGCAAGGAAACTCTGAAAGAATATGGTGAAAAATTCTTCATAAATGCTGAAACCTGGC

>78057

TGCAGGTTGTGACGTCACAACTGGGACATGACTGGGCAGATTGGTGTTTCAGGAATGTCTCTAGTCCGTCCCCTTCACGCTGCTTTTTCCCTGAAT

>64227

TGCAGTTCCAACACCAAGGGTGAGGGAGTGCCCTTCTAAGAGTGTTGTGAATTCCCACTTGCGATTTGTCTCTGCTTTTGCCAGTAACGCCAAACT

>108598

TGCAGAGACGAGAAAGGATCAAACAGGAAGCTGCCTTCAAAGACCAAAACAAGGATCCCAGAGCGGCGAAGAAGACATGGAGGCAGACATGGCTGA

>296532

TGCAGCAGGGTGAGTTACCTGGACTTTACACACCTGAGCAGCAGGTGGACTGGAGCTGAAGCTGTGAGCTGCATGTCTGTGTTTCCAGGTGAGCTG

>332641

TGCAGTCAATGCAACATTTCTCTCACCAAAACATGAAGGAGATTTTCTAGCAGAATATGCAGAACTGAAGCTACTTCAGCTTTTCTGATAAGAAAA

>234399

TGCAGCTCCTGCTGGCAGCATCAAAATGCCACTTTAGAACAAACTTTGCACATTTTCCTTCTTCTTTGGGAAGCTGGCAGATGTCCAGAGACGTAA

>240779

TGCAGAGACCAGCAGAACCTTCAACAGCCGGACAAAATGGCCAGAAGTCTGGGCCCATCGGCCCCAAACGGCCAGAGTTCTGAAGTTCTGGACCCC

>341733

TGCAGGAACAGTGGAACCTGAACGGAGGGCTCTGATAGGCCCAGTGGGGAGGAGATTGCTCTGTCCCTGATTGGATGATTAGGGGCTGCAG

>210566

TGCAGAGCCTTCGGGGTTCTGACCGCCACTGACCTGTGTCCAGATCCAGACCGCCAAGACGCAGGAGCTGAACCTGCTGAGGGAGCAGCAGGAGGC

>54511

TGCAGTTATTTCATAAATCTATGTCGTTTCGGTAAAACGAATCATTCCACCCGCAACACCCTACCCGAGCATGCTCGCCTTTTCCCCTCCCTCCGG

>293361

TGCAGGTGGTGAGGAGGCGATGGCACTTTGGAAAGCTAAAGTTTGTTCCGGCCCAAGCGAAATATTCCTTCCTTCCTTTCTGTGTGAGATGACATT

>50211

TGCAGCTCTTGTTTGCTCACTCCTCTTTCTCTCCTCACTACTTCTTCTCTCACTTCTTTTCACCTTACCCTAAACTCTCTTTTCTCATTTTAGTCT

>41108

TGCAGGTCATATTCACCAATTCCCAGTGTTAGACGTTCATATATTTACTGTACATCAGTCTCAGTACCAGGAGGCTGGTTGGGGTTCATAATCTTG

>139512

TGCAGTTCACAGGCTCCGGTCTGCTGTCAGCGCCCAGTCGGACCAACCGGGCCCTCTGGCTGATCAGTGTGTGTGTGCGTGTGTGTGCGTGTGTGT

>131168

TGCAGGAGGCGCTCTCTGCTCCTGGGTCACTTCCAAGCGGCGCAGATTGTAATTGATGATTCCGAGCGAGCGTTCCGCCCTGATCCGTCTTTGTCG

>18335

TGCAGATCAGCTTCGACGGGGAAACTGCTGCCGGTAACATTCGGCCGTCTGGAGCAGGGAAACCGAAAGGTCACGACGCATTTAAGATCGGAAGAG

>180852

TGCAGCTTGAGCTAAAGCAAAGGCCAGCCAGAGTGTCTGAACAGCTTTTCAATTATTCTCACAGCTTGTTCGGTCAGAAAGTGGTGACACAGCGTG

>18888

TGCAGTGTAATCTGTAGTAGTGCCTTCCTTTCAGTCAGTGAACTGTCTGGCAGCAGATAGCAAGGGGTTTCTGCCCAAAGACACCTTGAGGTGTCA

>104475

TGCAGACACAACGGCAGCAGGAACAACGGCTCTGTTTTACTGGCATCAACCACGACGGCAGGCATGTGTCAGCAAAAACTGTTGAAATACTTTCAA

>173229

TGCAGTCCAAAGCAATGCGGGAGCGATGGCTTCTCCAGGGAATGGGCGCAGAGGAAGAGGCGGCACGGCGGAAACAGCTGGAACACGACGAAGAAC

>192774

TGCAGTAAAAGACACTCCTGCGGTTTTCTACCTGTGTCATGTGTCTTGCCGTTTCCCCAGACTGTCTTCAGTTTAGGGTGGAGCTGAGTAGAGAGC

>159900

TGCAGGTCGGAGGAGGCCAACAAGGCGTTCTCGGCAGCGGTGCAGATGCACGACGTCCTGGTGAAGGCCTGGGCCATGTGGGGAGATTACCTGGAG

>251515

TGCAGCTGAAGTCAGACGCTGAATCTCCCCAGATCTCCTTTCTGCCAGTAACATCCGTCATGCATTTCCAGCAGAAACAGGAGATCTCTGAGAACA

>165820

TGCAGAGTCGGCGTCGGGTTTCTGTCAGAGGGCGCGACCCGGAGTCAACACGGTGAGTTCAGCCTGAAGCGCAGCCGCTTCCTCTGTTAGCCGTTT

>139840

TGCAGCAAAGTTTACAGAACCAACAGAGGAGAAGCCGCCGCTTTCCTCCCTCTGGAAGTTTGTTTGTTTCCCAGGGAGACGGATATGAAGCTCCAA

>289631

TGCAGCATGCTCAGCTGTGGTTTCCTCCATCTCTTGTTGCTATGCAATGTCGGCTGCACACATACGTGAGGGAGGATAACACAAGGCAACGACTTA

>177808

TGCAGCAGCAGAGCTGAATGGAGGTCGAAGACGATGCTAACAGGTTCGCATCATGATCCTGGTGGCGGCTTGGAATATTTAGCATATTTTTATGAG

>288104

TGCAGTTTATAAAATAACAGATTTTCAAGCTTTTCAAATGTTTTTCCATCTCTACCAGTGAATGTTTAGGGTGTTTCAGTTGTTGTTTACCAGTTC

>61743

TGCAGGATGCAGAAATGAGACATGGAAACAAAAGAGGTGAACACCACCTGGTCTAAGGGAGAGATGCTAGAAAGTGGTCCAGCATTCTGAGGGGAA

>43970

TGCAGCTCTCTCCCTCTTTCTGTCTCTCTCTCCCTTTGTCTCTCTCTCTCTTTTTCTCTCTGACCTATAAAACAAGTCTCCCTCTCTCCAGCTCCT

>259634

TGCAGCTAGAGTCACTGTGACATTTAGCAGCAAACCCCCTGCTGCTGAGTCATGATAATTTACTGAGACAAGACAGCATAACAAACATCATAATGA

>115415

TGCAGCAGGACGGGAAGACGAGCGGCGGAGGGACGAAGGCCCTGATCCCCGCCCCATCTGTCCTGCTCTGCACTCCTTCATCCGCCCTCCCTCCTC

>80217

TGCAGCGTTCTTGACGGAATGCATCGAAGCGTGCTGCACAGCCTAATGGACCCACAAGTTCCCATCTGTTAGTGGAGGAGATCTCAGTGGGTCCTT

>43741

TGCAGGAGCCGCGAAGACAGGAGGCGAGATGAGCGCAAACGCCGAGACTTTGAGCGGCACAGCAAAGCCGGCGAAGCCCACCGCGAGCGAGAGCGC

>242601

TGCAGGCAGAGGGTCCTCCTCAGACACAAACAGGAAAAAAATCAGACCAAATAACAAACAACTAGTGCAAAACCAGAAAAACAGACAGAATGTATT

>134762

TGCAGCTTGGTGTCGGGCTTATCTGGCGCCGGAACCCGTCCCTGTCCGATTGCCGCCTATTGATCAAACCTTCAAACCGTGTCTGATAAAAAACGG

>121583

TGCAGAAATGATGTCAGTAATTCAAACTAAATTGAATGGATTTGCAGAAGGCGAAATAGTTTTTCCAGACCTGAAAGTGATCTCACAAACGGACAC

>240756

TGCAGCACCTTTTCTGGCCTTAGACCATCCTGTCCCTTACAAAAGGAAAATATATTTAGCCATGTTTGACTTGAAATATATTGAGTCTGGTTCTGA

>63390

TGCAGCCCTGTCCAGCTTCTCCTCCCTCTCTTCTTCTTGCAAATGCGCTTAGGTTGCGTTTTCAAGAGACCCAAACCAAACAAATTATTCCTTATC

>140716

TGCAGCAGCGATTCCCGGGTTCATCTCAGCAGAAACGTTTCTCCTAACTGGTGATGCTGTAAATTTCGGGGATCAAACTTCGTGACGATCCGGCAG

>161259

TGCAGCATCAGCCCCAAGCGATGCTCCATCCTCTGAGTTTGGCCCACTGACACATATCGGCGCACAAACGCAGTTGCATGTTGACATCCTGAAGCT

>172106

TGCAGCCAAAGTCTTTATTGAACAGGCCTGCTCTGAGGAGATGAAAGACCGACATTCAGGGAGCGAAGGAAGAGAGAGAGAGAGCAATGGAAGGAG

>126970

TGCAGCTTGCTCTGGAGCCGCTGACCTTTCAGACTCTCAAGTGACCAGCTAAAAGTGTCAGTTGAAAGCAAATTGATCCACTACAGTGTTTGTCTT

>103836

TGCAGGTTATCAAGTTATGAAAAAATTTTACCCAGTATTGTTGAAAGAAATAGTCTCTACTTTTCTCAGGTAAGCAAAATCAGGGTTTCCCCCGGT

>40389

TGCAGAATGTGAGCTGTGTGGAAACACAGAGCAGACAGTGAGAGCAAGGTGTGAAACTACCAGAGGTCCAGATTCTGAAAAGTAACAGAGTACAGA

>93076

TGCAGACTGATAGGAGGGATTCCAAAGCAGCAGATAGGACCTCAACCAAGAGCCATAAATCTGGCGAGTGGGCTGGATTAGGTTACAGAAACGCCC

>162398

TGCAGGCAGAGCTGCACCCAAACCACAACTTCCTCGGCCGATATCAGGTTTCAGGGCGGCTCGGTGTTTTCCAGACATTTCGCAGCTCGCCGGGGA

>24611

TGCAGGGGGGGACGTGACTTTGCTGGCTGTGTCCGACATGGAGCCTGGCTGGATTGAACTCATCAGGAAGGGCGCCACTGCACCGCCCGCTACAAA

>280228

TGCAGCCTGAAAACAGCCCAACATAACACACATTGACACGGTGGCTGGTATGCACAGCGAGGGGCTGATTCAGTTAAGATCGGAAGAGCGGTTCAG

>278849

TGCAGGAGTCGGAGGCGTGATGCTTCATACGTTATCGTTCCTCCACCGCTGCTCCTTTCTTCCCCACATGGAGGCAAAATGCAGAGGATGTGGCGA

>138855

TGCAGGCGACCCGCAGCGTCACTCACCCTCGCAGATCATTGGCTCAATGGTGAACACATGACCCGGCTTCATGACCCCAACCGCTTTGTTTTCTGG

>187798

TGCAGGTCTGCAACAAGCGCGAGAAACGACTGACCGACCCGGTTCTGGAGAAATACATCATCAACGTGGTTTTCGACACCATCACCGCCTTCTTCA

>215985

TGCAGCAGGCTGAGCCCCTCTGAGACCCTCATTCATATTCTGCAAACATCCCTCTGCTTTGCAAGTCGTTTCATTCTTCAGCCAGACTTCCAGTCT

>240857

TGCAGGGGCGACTTTGTCATGAACCACTGGGGACTTCACAGGCTCTGAAAAGCAGAACCAAACGACATGGATGTAGAAAGGAAAACAAAAACAACA

>87625

TGCAGGAGACGCAGCTGCTGACTCCGCCCACCCCACCCAGAACGGGCAGGACGCAGAGGAGGCGGTCGCTACGGAAACCATCAGCAAGGAGGAAGC

>324085

TGCAGAGCAGGATTTCAGTATCGTTCTCGCCCTCTGGGGGGCGCTGGAGAGCCGCCCAGAGGCCTTTCATTGTCTTACTGACGACCTGCAGG

>52984

TGCAGTCTCCCAGTGATGTCACTGTGATGCAGCTGACAGGTGAGAGGGAGCAGTACACACCACCTGACCTGAGACAGGTTAGAGGTCAAAGTTCAG

>3638

TGCAGAATCCCCTCAGTTCCGCAGAAAATGATCGGCAGCGCTAACAGGAAGTGGCTCCCATTCGACACGGCCTGGCATCTCTGGTCCCGCAGCGTC

>24344

TGCAGCTGCTGAACTGCTGACCTGGCATGAAGCTGAGGTCAGAGGTGAACAGGTGAGCCACAACACAGGGAGATCAGACAGGCTAATCCTGTTAGC

>277019

TGCAGAAATTATAGGAAAAATGAGAAACGGTCACCAACGTCCACCTGTCTGGAACAGGTAAAGGGATTCCAGCAGGAAAACCAGATGAGGGAAACA

>222413

TGCAGTCTGGTCCTGTAGCAGAGGGAAGGACTGAATTTGTCGTTTCTAAGTGAGTCACACAAAGTATGATTGTTATATTGTGCAGCCAAGAGGGGA

>237165

TGCAGAGGAAACACACCTAAAATGGAGAACAAGGGTTGGAGCCTTGTGGTGAGCCATTTGAAGGGAACACGGAAAATAAGTCCTGTAAAGTTGGAA

>163866

TGCAGCTAGGAATTCAGTCAGACTGGAATCAATATGAACAATGATAACGAATCAATAAGTGAATTGATTCTGATGCTGAGGGAACCAGCCTCTGAT

>224836

TGCAGCCGCTGACGGACCTGGAGACCGTTTCATCATGGGGGAGTCGCAGGCCGGACAGCAGGTCAGAGAAACTTCATTCTGACGGCTCCACCTGCT

>47478

TGCAGCTGTGCACATGGTTTCTGCTGCTGCACTGAATGCGCCAGCTGCACAGGGCTCCACTCGGCCCGTCGTCATGAGAGAGCGAGAGCCAAACCT

>126457

TGCAGGCCGCCTACCTTTCTCCAGCTCGCTGATCCGCTCCACGAGCGCGTTCAGAGTGCTCTCGGTCTTCAGCCGGTAGGCGGCCGTGGCGTTGGA

>60911

TGCAGAGGAAAACCACATCCATCACTCCTCTAACACAAACCACACACACATATGCAGTGCTTTCCAGGCATTGTACAGGCAAAGACACACACACAC

>331581

TGCAGCAGCTGCTGGTTTATTTGTTTGTTTCTGACTGGAATCCAGGTGAAGTTTCTCCCACAACAGAAGCAAACCCAGAGGAAGTTTAGTCTCCAT

>26962

TGCAGGGACGACAAGACAAAATAAACCCTTCATCAGCTCAGTGATGAAATGACGGCACATTTTTTTGTGCACTATAAAATACCGATCAGGGTTTCG

>8000

TGCAGCCAGATTATCCCGACTCTGGTGATGTCATCCTGCAATGTGAGCGTTCCCATGATGCAATGCTCTGGAGACTGAAGCAGGTTTGATCTGCTG

>270169

TGCAGGTCGGCCCTCAGCTCGGCGACGCTGCGCTCTCTCTCCTCCCTCTCCAGCCGTCTCTCTTGCTCGCCCTCGCTCAGCTGCTCCTCCCTCGCC

>100245

TGCAGGGGCCGTTCAAGGCGCAGATGGACACAGAAGAAAGTCGTTTGAAAGATCAGGTGAGGACAGAGGATTCTGTCTTCGCTACCTTTTTTATTT

>67052

TGCAGGGCCAGAACCTCGCCCCGACCCGGAGCCGCCTCGGTGCCGGACAGCTACGGGTTGGGGAGGGGCGGCGGGTCAGTTAGCTGCACTGTACAA

>127197

TGCAGCAGCTGAACCTCTTCAGCTTGTTCAGGGCTTTATCTTACCTCTGCTCCTGTCATGAAAGGACTTCGGAGCGCCTCAGGTAGCAGACCGCAT

>49676

TGCAGCCGTTCCGGGAGCAGCTGGCCTCCACCACGGACCCGGATAAGAAGCAGATGTTGGAGCGTCTGGACGCCGCCGTTGCCACCGCTCTGCAGA

>205945

TGCAGGTACGCAGCTGATGTGTGTCACATTGAAACCGGTGGCACGGTTTACTCCGTACGGACGGACTGATGTTGTTTTTCTGGTTCCCAGGTTGCA

>270506

TGCAGGTCCGGAGACGAACCGACAGCCTGAGGAGCTGCTGCTGGAAACCGGAACGTCGTGGCTGCCGAGTCGCCTGTTACCGGGCAGAACAGGCGA

>152965

TGCAGGTCTGAAGCTGCGTCTCTCTGTGATTGAACTCTGACCTCCGCAGTCGATCATGTTGTACTCGTTGGGTCGGTCCAGAGGCCAGCAGTCGTA

>8021

TGCAGTGTTGCATTATATTCCCACACATTGCTTCAAATACTTTCAGCTTACAGGTTTTCTTCATAAATGTTAGATTCTTGCTCCGAAACAGGGATT

>175727

TGCAGCACAAGGCCTGTATATACATATGGTGATGTAAACCAGTGGAGTGACTGGTTCCAGTCTCTCCATCTTCATCTGGGTCGCTGCATTTAGGTC

>21222

TGCAGCTCCAGCTTTGCGTCCCTCCAGGTTCTGCTTTTCATCTGGACTGAATGTCAGTAAAATGTTCCCCTACCTTGTTGACACTTTATCAATTGC

>36563

TGCAGCAGCTTCTCTGGGTTTTTGTACATCAAAACTCCTTCCAGCAGTTACTGAGGATATTGGAATCAGGAAACCAGTGTAGGGCTCCCGAGAGGC

>8777

TGCAGCTGATTTGGTCAAAGGTTTGAGCCCCAACACAGTACCAACAGTTTTGCTAAATGTACTAGATTCTGCCTTAGGAACAGTGCAGGACGGGGA

>198451

TGCAGCAGCTAGTCCTGGAAAAAGTGGAGAAAATGAAGCCATTAGACGAACATCATCAACTGTAGAAATGTCAGTTCAACTTCCAGGGTGATGAAA

>5579

TGCAGCAAGAACTATTCTATTCTATTAGGGAAGACGACAGTTGGCCCTGGACTCATGAAGAAAGTATCACAAATTCTTGAAGAATATGAGACCACA

>221878

TGCAGCCATCGTGACATATGCCACCCTTTCATGAGGTGAGAAAATACAATGCATTTGGTGAAGGAGGACTTATTTGTAACCCCAAAACTACCATTC

>224545

TGCAGTGGTTTTCCACCATTTCATTTTCTTTTTTGTCTGGAGGATCTTTAGGTTGGATTAGGAACCAGGTGACTCACTTCATGTCTAAGACGTGTA

>71904

TGCAGACGGTGCGGAAGGTGGAGGATGAGGACATGGAGGGACAAGAGGAGCCACCGTCTCAAATGCTGACGCATCACTTCAAGGAGGAAGAGGAAG

>33272

TGCAGGAAGCCAGTCCAGGAAGATGCCCATCAAGGTGAAGGTGTTTGGAGGAGATCCGCAGTTCATAGGCGCTCTATCTGTTCTGGATCTACAGAA

>115100

TGCAGAACCTCCCGTCTGAGCGGGTCCAGAATCTGCCAGGACGTCAGAACCAGCAGGTCCAGCAGGAGGATCCAGAGCTGAACCAGAACCTGCAGC

>221162

TGCAGCTTCTGGTTTTACCGCCTTGCTTCAAAGCAGCATCAACCTGTAACAAGCTGCTGTAACAAGCTGCTGCCACAAGGTGGCGGTAATGCAACA

>85342

TGCAGCCGTGCATGCTGCTTCTCAGAGTGCATGTAGAGTCCCGCCTACACGCTCTCATAGAAACGGCATGCATCAGATAAAGTCTTTCAACCAGGG

>154371

TGCAGCTTCTCTCTCTCTCTCTCTTTCTCACACACTCATTCACTCACTTCCTCTGTTTCATTTTCCTCCTCTCCTCTTCTTTGCTCTCTTCCCCCC

>150999

TGCAGTGCAGGTTGATCTTTGCTGTGGTGGAAATACTCGAGGCTGCTGTACCGCTTGCAGGAGCAGCCCTGCGCATGCAGGGGCACCGTATTGTTG

>163796

TGCAGACACGGTAAACACACCAACCGCAGGCAGCATATCGATTCATGTGTTTTCACATGGGCACCAGCTAAAGGTTGCTTGTTGTGCCATGCAGAT

>328462

TGCAGTTGTAGTTGTTTGGGTTGCAGTTGTAGTTGTTGGACCCATCGTTGTAGTTGTTTGGGTTGCAGTTGTAGTTGTTGGACCCATCGTTGTAGT

>185179

TGCAGGACCGAAGTGGACATGGACGTCCACCTGGACTGACTGACTGTCATGGTCATAAATCTGACCTTTGAGGAAGAGTGAGAAGAAGACAGCCAA

>257594

TGCAGATCAATGATCATCATCGATTCTGAGGGACAGCAGCGCCTCCCTCAGGTCACACAAAGAACTTCCGCCATCAGTTTCACATCTAAAGGTTTA

>19448

TGCAGTTCAGCTGCTTCCAGCCTCAGGAAGGTTCTGGTTCTGTCCGTTAGAAACCCGACGGGTCCCATCAGGGTGGGAACCTGCTGACATCCTTGT

>40384

TGCAGCTCTCAGATGGATTCCTACTCAACCTGAATGTCTCGACAATGTCTCGTCTCATTGGACTGCAACAAGTGGTACTACTGGCCAAGAACCCCC

>220497

TGCAGTCTCCTATCTATCCTCTGTCATTTTCGCTATAAGACTCACTTTGCATTGACTTGAATGAGAGATTTTTCTCTTCTTTTTTTTCAGGATTTG

>160403

TGCAGGTGGCTCACCCGCAAGACCGAGGCAGCGATGCCAGGGAAAATTGCCCTCCTTCTTTTACAGTCTGCATGCAGCCCTGAACTAAAAAAAAAA

>9288

TGCAGCTGAGGAAGAATCGATCTGCTCAGAAGGAAGCTCCAGCAGATTGTGGATGTCCAGCTGCTTCAGGGTTTCCTCTCTCAGAAACTGGATACA

>5985

TGCAGCACACAAAGAAACACCCTGCATTATGTCTTTGTGGAGTGTGGACAACGGTGCTCTCTGGTGGGACCGGGCTCCCACGCAGTTTAGCTGCAG

>36430

TGCAGGAATCCCAAACATCTGCTGCTCCACGGTTCTGCTTCTGGCTGATGGTTCTGGTTATGAGCCCAGTTTGCATGGGAGCCTTCATGACCCAAT

>195982

TGCAGTGAGTCGAGGCGTCCAACGTCGGGCTTTGTTTAGGATGCCAGAACCGGTTCAACAACGAACGACTCACTGACACTCCTCGACCTTTTCCAC

>290611

TGCAGAGGAAAAATAAAGATGCTGCTCAGCATTTCAGACGCAGCAGATCAAACCTGCTTCAGTCTCCAGAGCATTGCATCATGGGAACGCTCACAT

>94575

TGCAGCGAGGGCAGCGCATGTTTCCAACCCCTCCAAACAGCCCCGCCAGCTTCACGTGCCGGTCTCTGTGCTTCCGCCAGCTCTGGTACAGCCGCT

>185624

TGCAGTGGTTGCTCCCACCACAGGCCTCCGAGCGGCTCTCACTGGCTGACGGAGGAAAGCTAGAGTGGTGTTGGCGTTGTAAAATGGCCTGTAGCA

>27279

TGCAGAGTTAGCTTCCTCCTCATCTGAGACAGCAACACAAACATCATTTATGCCATTAGAAGAAGACAAATAAAATACTGACTGTGCACAGAAACC

>130719

TGCAGTACCAGCTGCTGCCAGTAGAGGGAGTTGGCACAATGACCTCTCTGTTGAGAACCACAAGACATTTCCAGCCAAGTCAGCAGCAGCAGATTT

>60544

TGCAGAAAGAACCTTGTCCAGATGTTGCCAGTTCTCCTAAATTGCCATTGATAGCCCAATGCTAGCTGGCTCCTCCAAGAGAAAGAGGGAACAGCC

>209343

TGCAGCACCTGGCTTAGCAACTCATCCTCCTGCACACTGACGCTCACCAGAGAGTTGAAGAGGTGTTGAGAGGGAGAGACATTCACCACTGCAGAC

>31622

TGCAGGGTTGAGTGGCTGCTGTGCGGAGGTGGGCAGCATTATGTCATTTTTTCTGGAGAGATTACAAGAACAAGTGCAGGTACAGCAACGTCTGGG

>143336

TGCAGCTGGGCCCGCAGACTCCTGACCTCCCCCAGCAGCTCCCCCAGCTCCAGTGGGAAACCCGCCACCTCACACAGATACCCTGAACAGGAAATA

>39460

TGCAGGGACAAGGCCAGATGTCCAAACCTGATAACGATATATGTAAAAAATGATCGTCTGGTCTATGACAGACCCACTGAAGAAGAAATCCCACAG

>129015

TGCAGTCTTTGCAAATCGTTAGCAGAAACAGGAGAAAACTGATGAGCAACTGGAATGATTCACATGAGGAACAAGGAAGAAATTCCCTCTCAAGAC

>63926

TGCAGTCTGGTTTTGGCCTCAGCTTCTTTTTCTGCCGTCCGTCTCAGGAAAAGCTCGATGTCGGCCATCTCCCTGAAAACGACGTCAAAAATGTCA

>185433

TGCAGTCAGACAGGATCGACGTTCCTCCTCCTGGAAACGTTGTCCCTCAGCTCCTCTCCCTGATGAGACGAGGTGCAAATCATCTTTTCTACTTTC

>296434

TGCAGCTGAACTGGAACAGAGAGACGAGATACTCAGTCACAAATCCACATGATTACATCTAGAGTTGAAATGGGAAACCGTTGTGGTCAGATCAGC

>30046

TGCAGGCCCAGGCGAAGAGCAGCGGATCAGAACCACGTAGTACCTTCAGATACAGCTGCATGTCACACACTTCCTGCTTTCAAAGACAGAAAGGCT

>136111

TGCAGATGGAGCGCCGGGAGATGGAGGCCGCCGACGAGGAGGAGGACATAGTCGGCGAGTTGGAGGAGTGGTTGGATGCGTGCGAGGACGACAGGG

>189241

TGCAGCTCACCTGAGCTAACGGGGGACAGGTTAGCAGAGGGACGCTTTGTCTCATCAGCGCTTGACCTCCGACCCAGAGGTTCTTGCTGCCGGGCC

>162690

TGCAGTTCTACCATGAGACGGTACACACACCTACACACTTTTACACCCAAACAGAGGAACTATATCTTTACGAACAGATCCCAGCCACAAGCCAGA

>280861

TGCAGCTGGTTTTCTGGATCGGATCCGTGTCGGCCGGTTCTTGACCTCAGTGGATCGGTGCAACACGAGCTTCACGTTTTTAAGATCGGAAGAGCG

>211782

TGCAGTTGGCCTTCTCACCACATGGTGGTGCTGTAGAACCTGATGGGAAACTAACTGTTGGCAGACGGGGGAGCTAATGCGGCCGGTTTCTATGGC

>72194

TGCAGCCTCCCTCTTTGTCTCTGCTCTGTCACCTTCCCCCTCCCCCCTTTTCACTCTGCCTGCTGCTTTCCCAGAGAAGTCGACCTGTTATCGGCA

>92820

TGCAGTGGAGGTCTCTGTTCTGACAGGTGTGTCCTTGTCTCCGTAACGACGGAGGAGATTTGCCAGATAACGACCGACCCTCCCAGCTGCCTCTGC

>109053

TGCAGGTTGTCATTGCTCCTAATTGGCTCATGCTACCAAAAAGGGAACAGTCACTACTTATAAATTAGAGCCGGCTAACTGCACCTTTCACCAAAG

>267906

TGCAGCCAACTGGGGAATAACTCATCTCCACCAGCTGGGCGGTTTGTCATCCGCCACGTCCCAGCAGGGGTATGAGCTCATTTTTCAGGACAGGAT

>61247

TGCAGCCATGAACTCTGCTGTGGTTTCATCACAGACACACTGTGACACGACGATTCAGAACCTGGACGACATCAGAACCTCCTGCTAACATCTTCA

>64323

TGCAGGTGTCGGTCAGAAAACTGCTGAGGGAAGATCCAGATATGCAGAAAATCATGGAAGAGGCAGAAACTGGAGTCTCACTCACTGCATTTCCAA

>229196

TGCAGTGAAAAAAGAGCTCATGCAAATGAGGGAAAAAATGCCACAATAACACAAGAGTTATGTGCACAAATTACCTAAGCGATGTTTCATCACAAT

>151560

TGCAGCGTAGGTTTGTCGTCTTTCCAAAGAGGGGTAATCACGTCTGCGGTTTTGCCACCTTTCACCGGATGGATAGTCGTCCCTTCGTCCTAGCCG

>227014

TGCAGAGATCAGATGCTTTGTGTTGCCGTAAATGACGCCATGGCGTAGTTTTTGGTGGGTTCGATCACTCTGCTAAGCTGAACTGGATCCATTAAG

>162348

TGCAGGATGTTGCTTATTGTTTTGGACATCCTCAGTCGTACGGTTCTGTTTCTGATCAGATCTGAACAAGACGCTGGAGGAGAGTTATCTGATCCA

>23892

TGCAGTGTGAACATAGCCTATGTCAGCTTCTCAATCACCTCCCAACACACCTCAAAGACACAAAGTGCACATCTCTGCGATCTTATCAAGCACCCT

>134605

TGCAGGTTTCAGCAGATTCTTGTTACATAACCAGGAAGCTTCAGGCGTTTAGACAGTTTCATAAGAGCAGCAAGTCTGGACCGAACCGGAGCAGGA

>127803

TGCAGGCCCTAACCCCTTTATGGCAGCGAACAGAATAACTCGATGCTCCATTCAGACGATGCCGAGTCCAGAGAACCGGCACGGATCGATACGGAT

>218208

TGCAGCTGAACGCGGACCCGCACTTTTTCACCGAGGTTTTCCAGGACCTGAAAAACCAGCGCGATTCCAGCGAGTCCAGCTTGGATCGCTGACCTG

>254348

TGCAGATCCCGGTCAACTGTCCCTTCAGGACCCGCGTGGCCAACTACCAACGAGACGGCCCCATGTGCATCTCTGACAACCAGGGTGAGTTGTTCA

>142919

TGCAGCCTCAATGAGGTTTACCTCCCCCATCGCCCTGAAGTTTCTTCATCATGAGGATGCCATTGCTATGTTTGACAGCAGAATTACTGTGTTGTG

>154224

TGCAGGGTTTATTAGAGGGAACGCGTTTGAATGACTTACCTGCGCTGTAACTGCTTTGGATGAAACAGTTAGCTGAGCAATTTTCCAACAGGAAAG

>330312

TGCAGAGTGGAAAACAAGAATCTGAAGAACTTGTGTGTTCATAACTGTAAATATGATCAGATTCGGTTCTTCTGCACTGCGCGGCTGTTGGTCTGT

>310737

TGCAGTGAGAAATAGAGGTAAGACGTATCAGTTCTTTAGTTCAGAATGATCCAGTTCCCTGCCCCGGAGTGTAAGGACACATTTAGGATGACAGTA

>45450

TGCAGGCGTCAGAATCATTTACTGAACTCGTTTGGTGAAGTTTTCTCCTCACTCTCCAAGCTTTCACTTCTTTATCTTCCTCACAGCTTACAGAGA

>142558

TGCAGCTGTCCCATGCCATGAGCACCGCCTATATTGGTGCAACCGGGATTCAAATATTGCAACAGTGATGCAATAATAACACCACTTTATCTCAAG

>303671

TGCAGGGATCTGAGATCTGATGCAGAATCTTCAGATTCATCATAAAAACCAAACAGCAGCTTTATGATCCCGAGACAAGCAGCTGGTCCCAGTTTG

>147245

TGCAGCGAAAACACTCGGTGCTATCGACGACGTGGGATGCTCCATCCTCTGGGAAAGTTCTGGTGAACTGTTGGAGGATTCATGTTGTTGTTGATG

>232680

TGCAGGCCTGCTGCTCCTGGGGCCCTGGTCTGCCTCGCCGCTTCTTTTGCCGGGTCCCACTTCCTTCTCTGGGTCTCGGTCTTTAGGAGCAGCAGG

>193165

TGCAGCTTCCCGTACCTGTTGTTACCTGGCTGGGAGGCGGAGCTTGATTCGTGATGTAAAGCGGTGAGTTTACGGAGGTTTCCTGAAAACAGAAAC

>325898

TGCAGGGATGTCCAGAGATGTTCCCACTCAGGAATAATCTTCCTCTGTGGAGACGGATGGACATCTGATAATTCTGAGAAGAAACCTTACAACATT

>217681

TGCAGGAGCGCAGCAGACCACCGTGTCGGCAGCGGCAGCCGGAGGTTCAGGTGAGTCGGACCCGATCGGGGTTTCCCTCCTGAAAAGTCCGCTTGT

>299695

TGCAGAAGGGGAGGTGCTGTGGGAGTACTGTGTGTGTCTGTTTGTGTGTGTGGTTGACAGCTGACTTCCAGATGTGTGCAGGCTCTGCGTGCTAAG

>186518

TGCAGATGCTTTCCCGTCTCCGGCTGATCTCAGCTGGTTTCAACTCTCCACCTCCTCAAGCTTCACTTCTCCGTTTTCAGCCTTCTCTGCCCATCT

>19797

TGCAGGGCCCGTATTTCACTCAAGTTTCACAAACAGTAATCAAGCAATCCAGGGTGTGAAGTTGAATTGCTGTTTCTAAAATTGCCAGCGTTCCTC

>10453

TGCAGTGGCAGCGGGGGACATATGCACCGTCAGGAGGATGCTGGAGCAGGGCTACTCGCCTAAAATACGGGACGCCAACGGGTGGACGCTGCTGCA

>40989

TGCAGGTTTGAAGGCCGAGCTGACCGGGGAATTGAAGGAAAATTGATTGTGCAAGCTCGCCCTCGAACATCTGCAAAAGTAAATATTAGACGGGAT

>293427

TGCAGGCGTGCTATTTTGCAGCAGCGGAGACACCTCTGCGAAATGCCTTTTGTATGCCCGGCTGGTGTTTTTTCGTTGTTTTTTTTTTTTTTTTTT

>22216

TGCAGGAGATCGAGGTGATGGTGCAACCTTTCTGATGTATTGCTTGAAGACACACCTCCTTCACAAAACCGTCGATTTGACCTAAAATTCGGCCTA

>65077

TGCAGTGCAGCCGGCAGCTGGGGAGTGTGGACCAAACGCTGCGCTGTGCATTTGATGAGAATAAAACCCTGGATTTTGACATCGTGGGACTGCAGT

>243762

TGCAGCCTGGGAAAGGTTGGAAGTGCGACACACTGCTCCTGCATGCAGGTCATTTGCCTGTGTGAGTGGAAGCCATTTTGTTGGTCGACTGACCCA

>66897

TGCAGCCCCTCGCTCTCTTATTGGCTCGACTATCATGTCAGTCATCAGAGGTTCTCTCATCAGCACCGCTGCGCCTGATTCGATGCAATGTTTACC

>192701

TGCAGCTATAAATAGAAAATAGCTCAGCAGAGGAATGGATTGACACGCTCAAGTTCCAGGAGGGAAACGGAGAAAGATATTGTAGCTGAACGTGAG

>274081

TGCAGTCTGAAGCCACACTGAGGAAAAGCAGGTAAAAACATTTTCATGTGCATGAAAATGTGCAACACATCTTCATCTGGAGCCCAAACTTTTGAG

>163442

TGCAGCCCAGCGAGTGGATCGTCACCAAGGAGACGCCCAAAGCGGACGGTGAGTAGCGGAGCACGCTAATGCTACGCTAATGCTACGCTAATGCTA

>215419

TGCAGACTTCCTGCTAATAGGAACAATGTTTGCTGTCTTGAGGGAGGTGGGATCTCTCCTGTGCCTGTGATATTTCGAAAGTGTGAGTAGATTCTT

>223653

TGCAGTCTTTGCTTCCTTTATCAATTTTTTTCTCCAAGTATTTTCAATCTCTGTGGGGCAGAAGTATTCAGGTGTTCAGAGTTGCATTCAGTGTTA

>151333

TGCAGAGGGCGGGCGGCGCCCGTCGGAGTCGCTCTGTAAGGTCATCTTTGAGAACGAGAACGAGCAGCCGGAGCCGGCGACGCCCGGCAGGCCGCC

>165082

TGCAGCTCGGCCCCGCGGTTCCAAACTATGGGAATTTCACGCCAGGAGCGAGCAAATGAATGTTTCAATTATAGAACAGCAGGAAATACGGTAACA

>25803

TGCAGAACCGCTGATTGGGACAGCTTTACAAGATTACAAGAGAAACGTGACACGGACAACTAAAGCACGGCTTTTATCCACAGACAATACTGACAG

>156026

TGCAGGATGGGAGGGAAGAGGGGAAGAAGGGGAGGGGGAGGGAAGGTGAGAGGCAAGGGGGCGATCAGGGAAGGAAGGAAGGAGAGAGGAAACCAG

>320185

TGCAGTTTTTATTCGGGTTTTGCTGTGAGGCCATTTTCCTGCTGGTCTGAATCAGCTCCTGCTTCCTGTGTCGCATCTCGACAGATTCAGTCTGTG

>80141

TGCAGCTCTAGAGATGACAACAGGTGCTGCCTGTGGTACCTATTGTGCATCAGGGGTTTGATGCACAGATGTGACTCACAGCATGAAACCAAACAG

>225157

TGCAGAGCAGGGCCACACGCAGAGGCCCACAGGCCTGAGGCTGTTTTCCTGGCCTCCACGGCCCCCAGGGGCCCAACGATCGGGCCGTTAAGATCG

>210215

TGCAGACGTAAAAGTCCCTTTGCTTCTCTTATACATCTTAGCTGACATCCTGCCAAGCTGGACCGTATCCTTGCAGCAGGCAGTAGCGTCAAAGTG

>113745

TGCAGCAGCACAAAGAGATGCTGCTGATGAGCGAGCGTCGCAGCGGGAGCCGCTTCCTGCTTTATGAGCACAATACACCCGAGGACCTGAGGAAGA

>265936

TGCAGAGGACAGAGGCGGCATGAGGCTAATTTGGAAGCTGGAAAGTCAGGAGAAAAACAAAATCACTCACGGAACATTTTATATTTTGTTTGGACT

>114144

TGCAGGGCGGAGGTTTGCTAGCCGCCGTGGCGTGATGCCCCAGTGCCTGAACCGTGAGCGGCTGAACAGGAAGTGGGTCAGACTGTCACATCGCTG

>189479

TGCAGGTCCTGGGAATGTGCTTCGCCATGACGCTCTTCTGCCACATCAGCAGGTCTGGACTGGGATACAAGTTATGAACAGCCTGTGGCCTGACAT

>42179

TGCAGGACTGAGTGGATTTTGATCCGATTCCAGATGTTCAACCACAGCTGCACAAAGCCTGTCGATTATCCCTGTAGTTTGTTGACTATAGGAAGC

>237238

TGCAGTTGCAAATGAATGAAAGCACTTTCGGTTGAAGCAAGCCAGTCTATTGTAATGGGAATTTGTCTAGCAGCTATAGGCACCGGTTTGTCATTG

>201672

TGCAGTGCTGCTGCAACCGCAATATGCAAACGCGGCGGGGTCGGCGTGACACCGCTTTCATTTCAATAAAATGCTGAGAAACAAAAAGTCTTCTTA

>118255

TGCAGTGGTGGCAGCCAAGTCCCTACACTGAATTCCCTTTAGGAGATAATTCTCCTGGCTCTTGCTGGGCTTTCTTGGGCGTTCAGAAGTTCATAA

>63929

TGCAGCTGTAGGGCTGGATCCACTCTTATTGAAGAGACAATTCCTATTTCTAGTTTGGCTGATAGGGTTGTGCTTTGAACTCCCTGTTATACTTCT

>68737

TGCAGAATGTCGGAGGAAGTAGCAGATTATACTTGATGCAGGGCAGCTCCTCCCTGATGCCAGGTGAGGAGGAGAAGCTCCTCCCTGATGCCAGGT

>217111

TGCAGGTACATCTCCAGCTTCACGCCTGCGTGCTGCTCAGAATCAGAAGTAAACCTGGAATGAAATTGAAAAGCGGCGTCTTAGCTGCCCGCATGG

>37109

TGCAGCGCCGCGTTTCCTCTGAGTTTCAGGCTGCGGCGCTTTTCTGGTCTGTTGAATCCAAACTCCGCGCAGACCAGGAAGAGAAGAAAAGAAAGG

>271186

TGCAGGCCAACAGACAGTTATTTTGTACAGAGGCGCAGAAAAACACAACGGATTTCACGGAAGGGCCCACCCTGTCTGAAGGAAAAACAGGCCAAC

>205644

TGCAGAGGCGGATCGGTTGGGTTTCCTCATGTTGGTCTCTACCGTCTCCTCCGGTACCTGGATACTCAGGTGTTCTGGTGTTCATTTGTCTCTTTC

>19050

TGCAGGCCTGAGAAAACAAATAGAGACCCTCGTGATGGTCAGATGGAAAGCATGTCTTTGTTGCCTCGCCCTGCCAAGATCTGACGTCACTTAGGT

>12911

TGCAGAAGAAGAATCACAGACTCCTGAAGAGAAACATGAAAAGAGCAAATTCAGAGAGAGAGAAAGGAGGCGAAGATGAGGAGGAGGATGGTACCT

>152552

TGCAGGAACGAAAAGAGACACTCGAGTTGTAGCCGTTAGCCGCCGGCAGCTTCTACGCTAACAGGAAGTCATTCTGTTAGCTGCCTGTCGGTGCAT

>16603

TGCAGCTTCAGTGATTATAATCGGCCCGGCGCTCGGAACGCCACATGGAACTCCTTATATGGTCAACAAGCAGAAACACCGGGGCAACGGAGCGTA

>28557

TGCAGGGATAACCTCCACCCCCAGCCTGTGAACTTGCAGGATCCCTCATGCACATGCGTGTTTCAAGTATCAAATAAAGCACACTTCTGACAAGGG

>16733

TGCAGGTCGGGGAAGCCGTCACAGTTTGGACCTGGGGGTCCGGTCCAGCAGAACCTTCATGTCCAGCAGAGCCTCCTCCACGGCCGCCGCCAGGCC

>278010

TGCAGGTGAGCTCACATCACCTGACGGGTCTCCACGTTGCTGTCCTCCTCTCTCATGGACTGTTCTGTGATTGGCTGCTCAGGAGCTGGGCGTGTC

>98882

TGCAGGAGGATGAACTCAGACCTGGCTACCATAGCAACCATGGAGGACATGGACACGCTGAACAAGATGGCGGTCATGAAGAACATGGTCTACGCT

>20446

TGCAGCTCCAACCACAACAACTGAAGCAACAACAACAACCACAGCAGCTCCAACCACAACAACTGAAGTACCAACAACAACCACAGCAGCTCCAAC

>27113

TGCAGCAGGTTCTGAAACTCAACCCCCTGAGAAACGGACCTCAGGTTTCTGCTGCTTTGGCTTTTCTTAGAAAAAATACTTCCTGTTAGAGGAACT

>135125

TGCAGCGCAAGCAGGACGACCCAGAGGACGGGACGCAACACGCCACAGGTTCATCTTTCTAACCAAACTCAGAACTTAGACATCAGGGAGTTGAAC

>342817

TGCAGACGGGCGCGCACACACGCACGGGTAAACACGAATACAAAACTGCGAGAAACAGGGGATGGAAGGGCTGGAGGGAGAGTTGGGGGGGGGGAT

>312641

TGCAGGAAAACAGAACCCTGTTCTAGTCAAACAGATGATGCTGTCACCACCGTGCCTCACAACCTGGACTCCTCCAGATCCCACCACCATGTGACC

>124409

TGCAGAATCCAGGAGAACGACCGTCCACTTACCTTCAAAGACTTCTGCTCACTCTCAACACTGTTGTGAAGCAAGGAGGAATCTCACCTGCTGACA

>224249

TGCAGCCTGATGTCTCCAGAAAGTTTGCATACATTTTTGACCTGTTCAATGAGCTCCTCAACTGTGTCCGGCACCCTTGATAATTCCATCCTCTCA

>282735

TGCAGTTCGCAGCAGACAGCATGCTGACAAACACAGGCTGGTCAGAGGTTCACAGCAAACATACGCAACAAAGTTAAGATCGGAAGAGCGGTTCAG

>297681

TGCAGATCGCTGCTAATGCAATGACACTGATCTGATTTCCTCAGTCCCGGGTGACAGACGGCAGCAAATAAATCACATACAAACACGGGACGGCTC

>212266

TGCAGGGAGCCTCATGTATGAGCTGGAAAGTGAAGCCAAGACTGCCATCTATTGTTCCCACTCTAACAGCCTGCACTGCGCTGCTTTAAGATCGGA

>284862

TGCAGCAGAGGGAAGAGGAAGACTGGCTGATGAAGGCCTACTCAAAGTCCTCTCTCTTGGCTGCTTTCTTCTTTTTCTTCCCTTTCTGCTCTTTGG

>144069

TGCAGTGCTAACCAGCTTATTCTCAAACGGAAGAGCAGGTGGATATTCAGCTTGTTGTGTTGTGAGTATATTTGTCCCTTCTGTTATTGCTAGAAA

>62152

TGCAGAGTTGAGCCTCTGGCCGATTGTGTGTTTGTGACGTCACTGACATGTCCCGTAATGTCCTTTACAGGTGCGGAGCAGCTGTCGGTGCGGCAC

>52923

TGCAGCTCAGCTCCCTGCGCTTCCTCCAGCATCTTCTCTGCTTCAGCCTCAGTTTTGAACTCAATGTAGGCAATGCTGTGAAAAGAAAAGAAAATT

>8266

TGCAGATGAACATGAAATAAACACAAACTCAAATGGTGACAGTTTCAAATCGGCTTTAGATCACTATGAAAGGTAAAGGATTTGGATGCTTTTTTC

>61849

TGCAGTGCCGGCTCTGGAAATAACCTTTCTCTTTATGCCAAGAAGAGCGTCTAATGTTAGAACAGGCACAGCAAATTGTGTACACTCTGCACTCTG

>125314

TGCAGCAGGGCCATCCCTGGAACCAACTCTTCATCGTCCAGAACTTTTTAGGACGTTTGACCCACAGTTTGACCTTTGACCCACAGAACCGCTTAG

>173194

TGCAGCAGCCCGAGCCCGTTGGGTCGGAGATCCCCCAGGCCCGCTCTACAAGGCCATCTGTCCCAGAAACAAGACGCTCCGCTCGGCCTCAAGGAG

>165051

TGCAGCCTTATACGGATCTGTTCACCACACACCAAGTGGGAGAAGACCTTTAGCTAAGGTGAAAACATGACAGTGTTGTGCAAAAAATAAGCAGCG

>184736

TGCAGGCAAAACGAGGAACATGTTTGGATCTGAATGGATTCGGGTTTTTGTGGGAATCTGTGTGGAAGCCAAAGATGGTGGTGCGTTAAGATCGGA

>238148

TGCAGCTCTGGAACAAACAGTTAGAACTGAGCAGCTGCCAGCAGGGGGCGCCGCTGGATTATAAGCCTGGCAGCTCGCTCCGCTATACAAGCCCCA

>90610

TGCAGCTTGAGTTGCTATATGAGAGTTTGCCAATCTGATCTCTGAACCCTTTTACAATGAGTCCATCGCCTTCATCTGTGTTTCACAGGAGAGCTC

>97759

TGCAGCTATGAACAGACTAAAAGTTTTCCTGAGGTCTGAATTTCAAACTTCCCTTTCAAACACAGTGGGTGTCAGTGGTGGTTCCAAGTAAATCAG

>323695

TGCAGTTTCAGCTCTACAAAAACACAATTACAACTAAAGTTGGACACAAATGCACAATCGGCAATCTGTGGGTTTCATTTTCCTTGTTTCTTATAC

>133514

TGCAGGCGGCAGGCATGAAAGGAGGAGGACAGGAGCAAGAAGGAGATGAAGGTGAGGAAGCAGGAGGCAGGAAAGACAGAGGAAGAGGAGTGGAGA

>272131

TGCAGGTCAGATGGGCACTGACCGACTGAGAAACCGAGTAGTTTGAGATGTTTGGGTCAGAGCCGTCTGATTCTTTTTGTAGAATCTATGCTATTA

>298105

TGCAGTAGGTGGAGTTGCATCTCGTTCCAAACTCTTCTTCTCGTGTAACTGGCAGCCTGGACAACTTATGCAATCAATAGGTGAAAGGTTTTGTTC

>35590

TGCAGAACCTTCAGAACCAAATAATCCTCCCAGAACTTCTAACACCTATAATGGACCCTTCAATCTGAAACCGCATCAGAACCCAACAGAACTAGC

>31050

TGCAGTGTCGCTGTTCACTGAGAGGCAAATATAACAACATAGAATAAGAGGCTACAGTCAAGACATGTCCGACTGCTCTAAAATGGGAGTTACTTA

>77187

TGCAGGGAGGAAGTGAACAAGGCGTACAGGAAGCTGGCTGTCCTGCTGCACCCGGATAAATGCGTCGCCCCCGGCAGCGAAGACGCCTTCAAGGCG

>122135

TGCAGGTGACGGCTTCTTGCTTGTGCAGCTGTCTCCCAGACAGTGACCCACCACTGCTGTACCTATTCATAAATTTGGTTGAAATAATAAACGATG

>149617

TGCAGTCATGACCCGCTCCGACTGAGGCTGGCTCACCTGGGCTCGCGCTTTTTACCCCCACCCCGCCCAGCAGGAAGCACACCTGAGCACAAAACC

>30069

TGCAGACCTGGCTGCACAATCACCAGTCGGACTGCGATGATCCTGGCCGGGCAGAGATAAAGGATGATCCAGAGGAGCAGGGAGCAGGAACGAAGA

>311366

TGCAGTATTGGGTGTTTTACAGAAGGTTGTAGCCAATAGGTTGCTGAGATACTTTGAAATAAACAATAAAACCCTGAACAGTTTGGTTTTGTCCAT

>201408

TGCAGCTTTGCAGTAAATAGGTTGTGTGCTGATCACAGTCTGGTTCTATTCACCTGCATAGAGCACATGTCTTTTCTCTATAAGCTACTTTTATTT

>58840

TGCAGGTAAAAAGGGAATCATGAAAGAGATCAGAGTTGCTATCCGTAATTCTCTCTGGCACACAAACACATGTACACAGACGCTGCATTGCCGTGG

>149125

TGCAGTGAGAGGTTTGACTGACCGGCCACCGGGCCGATCTGAGAGAAATCGGTTCACTGACCCATGTAAGACGACGGGCATAAAACTTTATCCCTC

>48198

TGCAGTTCAGTGTTTGTTGTGTGAAGATCTCAGCGACACTTTACTGCTGGGAAACTGGAAGCAGCTCGACGTGACGGAGGATTATTTGCAGTTCTT

>202021

TGCAGACTCATGGCTGAGTCAGACTGTAGCTCCACCTTCTGGTCGACACAAGAACTCCAGATTATAAAGTCAATAATGGACTGAAAGACAGTCCAT

>232119

TGCAGGTGTCTTTGCTCTGCTCTGCTGTGCACTTTGTTGCCTATGGTAGAAATATTTGACGTGGTTGTATAGACTGTCAGAATATCAGCAGAACAG

>91973

TGCAGTCCCAGCAGGAAACAGAAATAATCCCGATTGACCTTCAGCCCGTCCAGGATGTCCTCCATCATCCACAGCTCCCTCTGAGCCTGACACTGC

>205528

TGCAGGACGGGAGGCGGAGAATCTCCGCCCAGCTGGCTGTCGGTCAGAGGTCAGAGTTCAGCATGAAGGATAAATGAGTTCAAGGCTTCTGGGTTT

>93339

TGCAGCAGATAAACTTGGACAAAATCCATTCACCCCAGTGAGAGAGGCCCAGGAGGTAGTTACCATCATCTGGAAATCATCAGGACAAACAATGGA

>62035

TGCAGCAGATGGAGCCCCGATTGGACACAAGTGAGACTTTTTGCGCTCACCACATTTCCAGTGGCTGTTGTGTTCTGGTAAACAGCAGGCGGGGGA

>44367

TGCAGGGCAACACAGAGACAGACAGGACAAACAACCATACACACACATACAGGCACGCGCGCACACACACACACACGCACCTACACACACGCACAC

>31601

TGCAGACAATCAGCCATTATAGTGGAGCTCCAAGCGTCTCGAGTCTTTCCTCTATTCAAATGCAGGGCAGAACGCTGGCAGCTAAATGTCATCTTG

>135283

TGCAGACACCAGCACGCAGCCGGCTGTTTCTGTGCCGCGCAGCATCATCTAGGTGAAGATAATCGCATTCAGACTGATGTTGTGGTAACGCCCAAG

>98064

TGCAGGCAGGACGACGTGAGGCTGCCGCCGTGCACAGACTCTGACCACGACACACATCTCACCAGAACACAGGCTGCTCACTCAGGACCCAGAGCC

>83847

TGCAGTACGGAGCCAAGGTCAACTGCTCAGACAAGGTAGCCAACGCCTCGCAGATGTTTATCTCTGCTTCAGACCCACACAGGGCAGAGACGACCG

>34960

TGCAGAGGCTATTTCAAGCATCAGCAGCCCTGACGGCGAGCGCATCATGTTTACATAGATGGCTGGAGCGACTCGCCGCTTCATGGCTGCGTGGGG

>190376

TGCAGTCCCGGAGAATTCAAAACCTGGCATATCGCCCAGGTGGGAGCAGCTTTATTGCAAAGCTCTGACAGGTAAATGCACAAGATGTGTAAAGGA

>73448

TGCAGGAGCTATGCAGAGCGACCAGTCAGCAGGAACCTAACATGGGTGGAGGAGGGGTGACGCAGCATTTTGGACTCAAAGAGAACCAAATTCAAT

>89438

TGCAGGAGTAATGCGGCGGTCCATCAGCGCGGCTGCATCTATCTGCTGATTGTTTCCAGGCTGGGTTATTATTTGGATCCTCACCTGGAAAAGTTG

>75547

TGCAGACACCAAGATGAGGAGCCGCCATCCAACATGGCCGACGTCTGGCCTCAGTCCGGAGGTGAACCGCCGGCGAGGCAACAACACGAAGCTAAC

>240147

TGCAGCCAACAACGCGCTCTTATCCTGGGAGCAGCCGCCCCTCCTCTCTCTTTCCTCCCTCACACATCCCTCACCCTGCCTCACGTATTCCACGCG

>136876

TGCAGCGATGGCGAGACTGAATCGTGTTCAGCGCGCCCTGCCACTCATTCTGGGCACGATCAGAGGAGCTTTTCCTTTGCCAGTTCATTTATATAG

>118869

TGCAGTTTCTGAGCCGGAACCAGAGTCCATGAAGAAAAGCAGAACTTCCCGGTTCTGCTGATGTTTTAGCCGAACACACAGCAGACCGAGTCTTTC

>26338

TGCAGCATAATCAGCCTGTTGAATGTGGTAAAGAAGCAGTTCTGCTAAGACGTGTGTTACAAAGATTTTCTGATCATCTACGGCGTAACGTGGAGG

>336819

TGCAGGTTGTCTGGGAATCTCTTAGCCGTCAGCTAACTCGTATCAATTTGCATATTTATGGAGAGATTATGCTGGAGGCCTTTGTAATGAGTTTTG

>296535

TGCAGGAACCTGAAGAATCCTGGAGGGAAAGAAACTCCAGAAAGTGTTCATCACACCACAGAGTGACACCAGACTTCACTCCACCAGACACACACT

>11143

TGCAGCAAGATCAGCAAACAGGAAGTCGACACAAACAAACAGGAAGTCAGGTTTTCTGCTCTCTGATTGGTTGACTCACGTATGAGCCAGCAGCAG

>215501

TGCAGGTCAACATCAGAGAGAGCGAGGCTCGGAACCAGGTACCAGACCCGGCCTCTGGGCCAGACCCGGCCTCTGGGCCGGTCCGGTTCCGAGATG

>163198

TGCAGCCAAATATTGTAGCTTAGGTCTAAATAATATTTAGGGTTATTTCTCACAACGTGCTGGTGTCGGTGCTTCATTGAAGGCATCACCTGTGTG

>83566

TGCAGCAGAACTAAGATGGAAGGAAGCCTGGATCTGGAAGCAAACTTCACAATCTTGACCCAAACGTTGCTGCTCTGGCAAACACACTCCAAGACT

>77042

TGCAGCTTGGTCATGAAAGCGAAGCGGTTCTGATCCGAGGCGGGAAGGAAACAGAGGAACACAAACAGCTTCTGGACCCGTTCAGAACCGGTTCTG

>285496

TGCAGCCGCGGCGGCGACTGCGAGTGTTTCTGCACCAGCGTGGCGGCGTACGCAGGGCGCTGCTGCCACGAGGGCGTTCCTGTCGACTGGCGCTCG

>312038

TGCAGCAGAAACAAAACTGATGTCATCACCTCTGGTTTGGATGAAACCCAGCGAGCTGGAAACGGACGACGTGGAACCACATCTGAAGACAATCTG

>7468

TGCAGCTCTGGAACCGGTACCGCTCCGGTCCACCTTCATCACCTCATCATCCTCAGGAATCACTCCTGGGATCAGATGGGACAGATTCCCAGAGGT

>268827

TGCAGGAGTCGTGGCAATTTACAAATCCTCAATTTATGATCTTTACAGCCGTTTTGTTGAGTATTTTGTCAAGTGTTTCCAAAGATATGACGAACA

>266783

TGCAGGTGGCGAACACGCGGGCCGAGGCGCAGCAGGCCCGGGCCCAGGTGGCGCAGGCGGAGCGGGACGCCCAGGCCGTCCGGGTGCAGCTGGAGC

>113024

TGCAGGTTACACCCTGATCATTCATCATGATAACAGCTGGATGGGGTCTGAATGTGTGTATGTGTGCTAGAGAGACGGTTTCAAACAGGAAAGCCA

>118411

TGCAGGTCTCTTGTTTTTGTTTTCAGGCTGAGTTCAGTTGCATAATGTTTCGTAATTTGTTATTTTGACTCGGGTACCTTCACCATCTGCCATCAA

>30720

TGCAGCAGGGAAAAAAGTTTGGTTATAAATGAACTTTCTCACAAGAAAAAGGCTGATTAGCCTGATCAGCAGGTGCATGGGAACCAGCTACAAACT

>137209

TGCAGCTATGAGGTGCCATCGGATCTGGAGGAAAGCCAGGAGGGCCATGATCAAAATGTCAGAGGTGCAGTCCAGATCTGCTAATCGGCACCGTGT

>86866

TGCAGGCAACAAGCTGACACCTCCAGCCCAACGGCTTCCAGTATCATTTCAGGGCTTTCTGAAGGATTCTCAGATGCATAAAGCTTTCAACCATCA

>48144

TGCAGCACTGCATGCACATTGTTCTTTAGGAAGATGGTCTAATGGCGACTGTGGCTCAGTCCGATCTCTTGCCATCAATCTCCTATAATTCCTCCA

>31734

TGCAGCCGATCGGATCAGAACCGGGCCAGAACTTTACGCTTTCTGCCTGATCCACAGAACCGGGTCAGCGCTTCACACTTCGACCCGGTCCGCGGG

>63699

TGCAGACAAGAAAGATCTCATAGTCATCCTCCTACCAAGAAAACCTAAATGTAGTGGAATTATGCTTCAACTATGCTGATCTCATAAGGTATGTTC

>159554

TGCAGGGAAAAAATGAACATGAAACGCACTCAGAGCCACAGCCAGTCGTTTCCGTAACTTGTATGTTCTTCCTCGTCTTTGGATAACCAACTCTAC

>258719

TGCAGCAAACCTCGCTGGATGGGGTTCAAACACAGGTAAAAACCAACAGTAAATAAGCTTCGTGTCCACACACAAATCCATACAATCCTCCACACA

>214641

TGCAGGGCCAAATAAGGCAAACTGGTGCTGATGTGATTTATCAAAAAGGCAGGAAAGGAAAATGGAAAAAGGTTTTTTCTATAAAGGGCTAAAAAG

>155101

TGCAGCCAGTCGTCCATCTGGTCCTCAATGTCCTCGATGGACGTCGTCTGGTCGTAGTTCTGCTGTATCTCTGGAGGAACGAAAACCGGAAACTCC

>99800

TGCAGGAGATGAAAGCATCCAGAATGCCTAAAGCGTTGCCAAAATGTTTCTGCTAAGATTATCGATGGCAGCTTCCCAGACGTCGGCCTTCCCTTG

>72271

TGCAGCCGTGTGCAGGTTTGACCTCACTTTGAGTCAAATCAATCGATGGCTAAATCAGTCTTGTCATCTGTGATGCATCCACATGATGTCAGAACA

>31578

TGCAGCCTGTAGTTTCCGACATTTTTGTGGAATGAAATTTCAACAACGTGCACACAAGCAACTGGTTATTTGAACAATTTCTCCTATGACGTGTTG

>285251

TGCAGCAGCACGCGCTAGGGCCAAAGCCGAAGCAGCTCGTGCACATGTTTCATTGTCCAGAGAGAGAGATCAGCATTAAGATCGGAAGAGCGGTTC

>119870

TGCAGTTTCGCTCTGAAGCATGACGTCTTTATGAAACCAGATAACTCGACGAAGCTTTGTACAGATGACCTCTTGTTGGCATGGAAACAGTACAAT

>191803

TGCAGAGACGGACGAGACGCTAAGCTAACGTACAAGATGGCCGCTCCAGGTGTTCGCATGACGGAGCATATCTGTTACTTTCCTTCTGATTTTGTT

>101856

TGCAGGCCGCAGGCTCAGTTTGGATCTGAGGGAGGACCCTGACACCACAATGCCAGCATGGGACCATGGAGCCCCGACCAGGGAGAGAATGAGTGG

>215892

TGCAGTGGCCCCTCTGCTCCCAGCTAGCAGCTAATGCTAACGCTCCCAGCTAGCAGCTAATGCTAACGCTCCCAGCTAGCAGCTAATGCTAACGCT

>118509

TGCAGCATTTTCTCCATGAACTGCTGGACCCGCAGCTTCACCTGGAGGAGCCAAAATCTGGATTATTCATCCTGTCATCAGAGGAAAATGGGGAGT

>242567

TGCAGCTCCATCACTTCCTGCTCTTCACCTTCCAGAATTTTATATCGACTTTTACTGCGGCGAACTTTACTCCTTCGTCTTCCAAGACACAAAACA

>309896

TGCAGCTCCAACCACAACCACTGCAACTCCAACCACAACAACAGCAGCTCCAACAACAACAGCATCTCTAACCACAACAACTGAAGCTCCAACAAC

>160091

TGCAGCCAGGGAAGGAGATTTATGTAGGCTTACATGACTTTGGCGAGCTCTCCACCGTCTTTAGGCCTCCACATCGCCGGGACACCTTGCCAACTT

>19856

TGCAGCCTCATGACCATATAAGGAGCTGCTGGCGGCACCACGTGACTCAGCAGCCAATCAGAAGCCGCTGCTCTGATTCCTGGTCCACTAAAGTGA

>117147

TGCAGGGCAGCGGCTGCCGCAGCTGATGCGCCGTCATCCTCCTCGGCTGGTTCCGTCAGAACCGGGCCGGAGCGTCCAGAACCCGTCCCGCTCTCC

>312099

TGCAGGATCTGCGGTCCGGTCCGGTCCAGGTTGTTCTGCTGCTGGTCTATCAGTGGAAACTCTGCGGTTTTGATTCTGAGCAGCAGGTAAGCAGTG

>264127

TGCAGTCAAAACAGCATCAAACATCGAAACCGTGGTTATGTTCCCGTTTGATTTCCACCCATGTCAAATCTCAGAATTTAGTGACACCTAGTGGGC

>7412

TGCAGTGCCATGCGTGTGCTGCTGTCCAAACTGCCGAGGCCGTGGATCGTCGACGAGAAGAAGGACGACGGTTACACCGCTCTGCATCTGGCTGCT

>341827

TGCAGACTGGCAGCTGTTGTGGATCTGGATCGTGTTTCAGCTGAAGATAGACGGCCAGTAAACATTCAGATTTTATGTTCCTCTGTGATGGACGTA

>41196

TGCAGCTGTTATAGAGAAACAACAGCGACAAAGCGCAGAGTTTACTTTCAGCAATTTGTCCAAAGTCAGCATCTAATAACAAAACTTGGCCCGTGG

>210569

TGCAGTGAGTTTGTTCCTCAGATGACCCGTCATGTCTCCCCTCCGCCCGCCGCCTCAGGTAAACGGTGGCGCGGCGGCGGCGCCCCAGCCTGCGGC

>210933

TGCAGCCATGTGTTCTAAGCCTGTGCTCTTTCACCCTGTAGAGTCTTTCACAGGTGTGAAAGTGAAAACATGACATGGTTTCCATAAGTGCTGGTG

>174757

TGCAGGCAAAAGTCCTGGCTTTTCTATGTAAATGTCGTGTAGATAGGAGAACGGTGTGGTCTGAAATTTTGTTTGGACTTACAATACTGTGCAAAA

>20582

TGCAGGCGGACGAACCGGTGGGAACGATCCAGCCTCTCCGGATCCGACTCTTCCTCCCCATGAGGTCGAGTTTTATCCTCAAACCGCTGGAAGGAG

>95668

TGCAGCTTGGTAAGAAATATGTCAGAAACTCACCAACACCGCAATAATGACTGTTTTTACGACGCACAGGAAAACTAGTGACTCCATGAAGATCTG

>241158

TGCAGAGATTTTCAAGAAACCAGAAAGAAACCAAATGTTTGCACCAACAGGTCCAGTTTGGCTGAAGGAAGAAGCGTTCCAAGGATTCTGATTCCA

>106090

TGCAGCACTCCGGTCCATCACTGCCGCACATCTAATCCTGCCTCGCCTCTACTTTCACTAACGACCTCACTAAGAGGCTGCGGCCCGCTGGGCCTT

>131195

TGCAGGACCTTTTTCCCACCATAGAAGGACATAGCCTCCTGCCGTCGCTGCTGCTGCTGAGAGTGTGCATGCCTCGGACATTTCAGCCATAACATT

>135082

TGCAGCTGTTCCCTCCCTGCTTTAGCCACCTGCGTAGGAAACATGAAGACTTCAGGTGCCCAGTTCATTCAGATCTTCCTTTCATCGACGGACCAC

>103734

TGCAGACAAGGTGAACTTCCATCTCACTTCCCGGCCCACTATTGTCCTGAGCCCTCACAGAAAATGTCCGCCCTCTGGTTGCCACAGAGACAGGAC

>137828

TGCAGTGAGCAAATGACATGGCCTCCCTTTGAATAATCAGCTGCATTATCTGTCAACTCCATAGTTTTGAAAGCCCAGTCAATACTTTATAATGGC

>297882

TGCAGCTCCTTCCAAACAGCAATTCATTCTGGATCCATTCAGTCTCCCCATTATCCTCCCCACGCTGTGTCTGGGAGGCCGATATTATTTTCACAG

>215210

TGCAGCTCTGAGAGATAGCTGCCAAGGCTCCCCTCTTCCACTTATTATATCGTGGATGGCTGCATTTTCTCCAATGTACATTACAGCTTTCATGGC

>308393

TGCAGCTAACCACAGCAGCTAACCACAGCAGCTAACCACAGCAGCTAACCACAGCAGCTAACCACAGCAGCTAACCACAGCAGCTAACCACTGCAG

>314215

TGCAGCTTCTTAGCAGGCGTGCAATCTGAAGAGCAGAGCTCGGAGTAACTGGGGTTGGAGAAGCTGGAGCAGCTGGAGTCATAGCTGTCACTGTCT

>177858

TGCAGCCCGGTTCTGGACTGGAATAATGATCATGAATAATCCGACCAGAACCTCCTGATCAGAACCTCTGGACCTGCTCTGTCCGGGTTTCTGTGG

>325858

TGCAGCAGGAGAGGGATTCTGCACAAGCAAATTCAAATCAACAGGATGATGAACAAGCTGGCCCGAGTACAAGTGGAGTTCCGATGAACACTGGGA

>332424

TGCAGTCAAAATATTTCTCTCCCAGAATCTGTTTTCAACCAAGTTTTCAACAGGCAGAGCTCACACAAACACAGCAGCCTCGTTCCGTAACGCTTC

>113661

TGCAGGGGCAGCGTGGGATAGGTCACCCCTCCTAAATGCATCTGCTCCCTGGCCGCACGCACATCTAAGGGAGGAGAGCACAGACGAGAGGCCGTC

>58016

TGCAGGAAGTTGTGGATCCGGAGCTGAAGGCAGTCAAGGTTCTGGTGGCGTCGCTCAACAGGTACGGCTCCACGCTCTCCTCCAGAACTGAAACCG

>178648

TGCAGGTTGTACCTTCACACAGAGACTGAGCTAAAGCAGCGGCTTGCTGACCGATCTGGTCAAACATCCTGAACACTTACAGAGCATCTGAGTTGG

>48325

TGCAGGTTGCAGCTGCTTCTCCCTCCCTCCGAGCTCCTCTCCTCTGTAGGCTTCATCACTGACATCTTTACGAGTCGTTTATGAAGCTGGACACGG

>284303

TGCAGATCATTTTATTGGCTGCTGAAACTTCTCACCTGGTCAGGTGAGAGGTTCCTGTAAGCTAAGCTAACTTGGCGCCGTCTCGTTCCAGATTTC

>141379

TGCAGCATGCTGCTCTCCGCCCACTAAGAACCCCCACACTCTGACGCCCCCCGCCAGGCTTCCCACAGCCCAGGATCTGGGTCTCACTCAGCATCG

>176992

TGCAGCTCAAGAACTCAGAGAAGTGAAAACTATAGGCCATCATGTTTATATCAGAATATACACTGATCAGGCATAGCATTACGACCACTAGTGATA

>31192

TGCAGAAAGAAATGCAACTTGCCTTGCAGGGAGATTTTTAGAAACTGAAGGCAATGCTGGAGGACTGGAGGTAAAAAACTTTATTTTCTCTCTGAC

>18567

TGCAGGGTTTCACTGGCTCCACATATGTTTTATGAGCTTTTTCCTGCCAACCTGCTCACATGGTAGATCACATGTCTGGCATTAGGGGATCACCAC

>29570

TGCAGGGGGATCTGAGGGACTGTGGAGACTGTGAGCAGGACAAAGAACAAGTTATGGATCAATTCCAGGACGTCCATGTTCAGGCTCCCTACACAG

>7925

TGCAGGCGTTGGTTTCTGCACCATGGAGCTTTTAGGCCTCGTCCCTAATTGTCGCAGAAGCACAGCTCAGAAATATCATACCGCTACTGTACTGAT

>200138

TGCAGGTATTCTGTGTGCAGAACTTCACACAGGGTCAGTGACGTCCATGCAGAGTCTTGTAACCAAACACAAGCGGTTTGGAGGTTTTGGCCAGTT

>91578

TGCAGCTCCACTCGCCGTCACATGGTTCAAGATGTCTTGTTCGGGAGAAGATAGAGTTACCGCCTTTTCCATCTGCCTTTAGAGCTCGCATGGGAT

>190030

TGCAGAAACGCCAGGAACGAAAACTGACAAAAAGCTGACAGAACGAAAGGCTGACGAACTTTTCAAGGTTCTGATGTGAAGATTATCTAATTAAGA

>193975

TGCAGCTCCCTGGCCACCCGGCACAAATAATGGTTTCCTCCTTGCGCTTGTGAGATAACGGGGACAACCAGCCTCCTCCGTCCTCCAGGCTGCCTC

>128720

TGCAGCAGCAGAGCACAAGCAGCAACCCGTCGCAGCGCCCGTTATGCAACAGACCCACAACCCGCCACCTTGTCCTGCCCGGACGCCGCTCTCCGA

>306250

TGCAGAATATAAACGGCTCCAAACCTAATCCAGAAAACGATCCGTGTCCGGACCGGGAGCGCCGCGTCTGCCCTGCCGACCCGTTTCGCTTTGGAC

>266361

TGCAGCTGTAGGTGAGAAATTATTCATTTTTAGCAATAGAAAGGACAATCATGTTCTTTACGATCCAAACATGAGGAAACGTGCACCATAAACACT

>45986

TGCAGTGTGTACAGTCTCCTCAGACAGAAAGAACCAGTGCTCCCAGTGCATCTGCCCAAGTCCCCGTCTGCCTCCCCTTCCTGCTTCAACACTCCT

>72535

TGCAGCAGGATATTGAGCAGCATCAGTTCGTTAGCCGAAGCCTCAAGGCTACGATGGAACAGACATGAAGCTTCTGCTGGTCTCAGATTTATAGCT

>84775

TGCAGTCACAAAGACCAGCTTGTTCAAGCTGGCTCTGTGTGTTTAGTAACTGGAAACACTTCCTTCGAGAACAAATCCTCCCGTATCACATCTACT

>119155

TGCAGACTTGACCTACTTTCGTCCTGGATCATAAATAGTTCTGTCTCCGAAGCTCGCAGGTTCAGCGTGAAGAATCAGTCATTGTGTGTGAACGTC

>116780

TGCAGCTAACATCCCAGTGCCAGATACTATTTCCCGCATTCAGGGATCTGGTGGAGGCCATACCTTCACAGACCAGCGCTGTTTTGGAAGAAAACG

>32745

TGCAGATATAACGGGACACAAACCTTCCAGAGTTCAGCTCATCGGTCCACAAAGTTTCCCAGACGTCTTTGGGATCATCAGGAAAACCGAAGATAA

>69776

TGCAGAAACGCTTCTGGTGTCGACAGCAGCCGCTGCTCTTTGCATTTACACCAGTCACCATGGCAACCATCCGGATCCACTGTCACCGGCCACATA

>232806

TGCAGTTGGTTTGGATCGAGGCCTGTCTGAAGCGAACTGCACCGGAGCCGGTTTGTGGTGTGCTATTTACTATGCTAACTAGCGTTAAGATCGGAA

>181497

TGCAGGTGCCATTTCTTTCACCACTGGAGGGCCACATCTACCTGAAACTGGACAGTGAGGGCCACTCCGATGTGCAGCACCAAGGCTTCCTGGTCA

>8862

TGCAGACCGTTTGAAATCCCTTCCCAGTCCTATCGGCTGTTCGGGCTGCGAGGGCCTCGTGATGCCTTTATGGTCCCGACTCGGTTCGCAGATTGA

>36830

TGCAGAGAGTACGTGGAGAAATGCTTAGTTCTTCGATAAAGTTATTCAAACGTGTACTTGTAGGTGCCAAGCTTGTTGCAAAGGAGAGCCTCGTGG

>294934

TGCAGCAACAACAACAACCACAGCTTCTCCAACCACAAACACTGAAGCAACAACAACCACAGGGGCTCCAACAACTACCACATCTTTTCCATCCAC

>225969

TGCAGAAAGACCTACATGGAAGTGACAGGCACTGCACGCAGGCGGCCATGTGCACTACTAATCCAAAGATCACCGAGCTCAACTACATCTGTGGGG

>118295

TGCAGCTCCAACAACCACAGCAACTCCAACCACAACAGCAGCAGCTCCAACCATAACAACTTCTCCAACCACAACAACTTCTCCAACCACAACAAC

>156029

TGCAGACTAATCCCTCACAGAGCGAACGCCGAGTCAGCACAGCCAAATGACAAGGTTCCACGTGTATTACTGTATTTCAGCTGATTCCACCTCAGA

>294703

TGCAGCTGCTGGACCCTCAGGTCGGTTCTGGACCGGTCGGTTCTGGCTCGGTTCAGCTGAGCGGCTCTCTGTCATTAGCTTTATCTGCTTTTGTTC

>180810

TGCAGGCGACTGGAGGAACAAATTCCCTGGCAGTTCTTATGACGACGTGTCGGATCAGACGGGAATCTGACGGAGGATAATTACTAACAAAGTCCT

>326475

TGCAGCTTGGCTCCAACCCTCCATCTAACTATAATAAATATTCATATTCTTACTAACATTAGTAACAGATTGTGCTCATAAAGTCCTTGGATGAGT

>83395

TGCAGCGTTCCTCAGACACTGGGATCCTAAACAGGCTGACAGCCGTCCTCATGCTGACCTTTGCCACCTCAGCTGACCGTCACACCAATCAGGCAA

>41624

TGCAGGATTAGACGCAGGTTTTCCCTCCTGGAGGAGGAAGATCCCCAAACGCAGCTGCTGTAGTTGTGACATTTCCAGATAAACGTGTCATCAAGC

>274456

TGCAGATTCGGTGCAGCTGAACGGCCAGTTGGGACATTTCACCTCGCGTCACCCTGGAAGAAGCATTTAGATTTTATTTAGTTTTTTATGAAAGGA

>242467

TGCAGAAAATCAGCTTCAGGAGACAAAACAAGCTACCAGCCAAGGAAACATGCATGGTTTCTCTCCTCTTCAGATATCAGGTAAACAGCTAAAATA

>152793

TGCAGATTCGCTAATTTCAAACCTTCTCTCTTCCTTTTCAAATACAGGAGCATTGTCATTTATATCAGTGATTTCTACGGTAACGGAAAATATTTC

>192775

TGCAGTACTGTATATACAGGAATACAAAAGCCAAATATTACAGCTTACCTACAAAATGTAGCAGGACTTATCCAGAGGGATTGAACTCTAAAGCTG

>76411

TGCAGAACCGAGAGATCAGAGTGAAGCTGCGGATCCGCTCCTTTTCAGTCAGCCGGTGGCGTATCCGACCGGAGTAAAACAACAAAACCTGGGCAA

>69039

TGCAGAGCTCAGCAGCTGCTGCTGGTAAAGATTCAGAAGTTCGTTTGTCGAGGAGGGAAGGAAGAAAATGAAGGCCACCGCACCAGCAGGGTGAGG

>220291

TGCAGAAGGACGACGTTACAGACCATCAGCGGAGGAGACGGGATGGAGGACGGGAGGCTGGTGGAGGGGCTGCGGTCGCCGGGTCGAGGGGTTTTG

>26119

TGCAGGTGGAGTAGAGCTGAGTAAACAGAGCAACATGGTTCAAGGTCTTTTACTGGATAAAAACCATATCAACAGGTTCTACTGACCTGTGAGACC

>79540

TGCAGCATAAAATCTGATCTTATTACGTTTTATTCTAGTGGAGGTGGGATGTAAACTACTGTGAGGCGTTTGGTAGAAAGTTTTTCTGTCATGTAA

>85595

TGCAGCCTGGGCCTTTAGTAGATCCACTGCCGGTTCTGCTGGCACAAACAGGGACCGCTGGGCCCGGTTCTGGTGGTAATGGACTCCGGACCAGCG

>225558

TGCAGACAGCCTGTCATTTTCTCTCTACTGTGTTAGTGCCGCTCTGCTTTCTTCCTTTTTTCTCCTTCTCTAGGCTCCTTGACCCTAGAGAATTAT

>316933

TGCAGAGTTTCTCCTCCTGCTGATGCGGTTTCTGTTGCCTAGGCGACCTGAAGAACCCGGATAACTTCCAGTACGGCGATGAGACGATGCGGGCCT

>155645

TGCAGATCGAGGGAGTGAACGCTGGCAGGGACATCAAATGCAAGTGTGAGTGTTTTTATCTCCAAGTGGTGGAGTTGGGTTCACCTTTTGTACTTA

>68909

TGCAGAAGTCACAGAGCAAGCAGGTTTGTCTCCAAACAAGTGATTGAGTGTAAAACATTTGACAGTTTTTGGATTCATCTACATAAAAAGGATAAA

>28791

TGCAGTGCTAAGGCTTGTGTGCTAATGCGGATCAGATCCATCTAACCTGAATATTGACAGAAATTCAGCATTTCGTCTTGTAGTTTATGGCAAAAG

>390

TGCAGGGATTACCCAGTGGTGCACAGACATGAATGTTTCACTGGACTCTAGCTTGGCCACAGTGATGATACTGTTGGTGTGTCTCAGGACTACGGG

>120652

TGCAGACAATCTGACGCGCCTCTGCTTCTCCAGCAGACTCTTTCGACCCTCACTCAGAACTGGAAGGGGAGGCACCAGATGGGTCGTTTGCAAACC

>318901

TGCAGTTGTTGTGGTTGGAGAAGTTGTTGTGGTTGGAGAAGTTGTTATGGTTGGAGCTGCTGCTGTTGTGGTTGGAGTTGCTGTGGTTGTTGGAGC

>250176

TGCAGAACGACACGCTCCACAAATATGGCCTGTCTGATCGAGTTCAGGTTCGTGTCTCTGATTGGCCCTCATCCCTTTTCATACACACACTCTCAC

>317585

TGCAGCTGCGGCTAACCTGGCTAAGTGGATAATCCCTGTGGTAATCTGGGAGCAGCTGGAGTGATGTAACAGATTATTTCTGCGTCTTCGTTCCAC

>43399

TGCAGCAAGAAGGAGGGACACGTGGTCCGCAGCAGCCCCCGCTGACATTCAGAAAGGACACAGGGTAAAGGCAAGTGTTTGCACAGGAAAGTACTG

>277562

TGCAGATCCAACGAGGAGGAAAGACCAAGGACACCCATAAGGGGCAAAGAATGAGAAGGAAAAAACAAATCTAGTAACAAAAAAACTGGATAAACA

>104140

TGCAGAAAGTCGCAGAGGGGGCAGGAGTCGGGGAGAATGGGAGGGATCGGGCCGTATAGCAGCACCTACGTACATACCCCAATGGTGATTGGCAAT

>175015

TGCAGGATGCTCTGAAGATCTGCTGGAGCCAGAAAATGTTGACGAGTTTCACTGAACACAGATTCCTTCAAGAGACAGAAATGTGGCCAGTTTGTC

>2375

TGCAGTGGAGGTCAACGTTTGTGACAATGGCATAATCTCTCAGTCTATGTCCAATTTTCATTAGAATGCAAGTGCAGATGCAGAAGAAGGGAAACA

>285419

TGCAGAACAGCTGACATTCTTTTGAGTTGCACTTCCACCGGCTTTTAGACACTGACATCTTCGCTCGTTCTTTTTTGCAAAGGAGCTCGGATTGAA

>35319

TGCAGCTGATCCTGGAGCGCTCAAAGCTCCGCTCATCAATTTTTCCGGCCGACCTGAACACAAGATATGACCCATTAGTTTCATAACGATCTATAA

>278940

TGCAGCAGCTTCATAAAAGAATCAAAGGGAGGACTGTGGTGGGCTCCTGCTCTTCCCTCTCCACCTAAAAATCAGAACACAAACACTTCAACGGTG

>131231

TGCAGAACGCGTGCGACGAGGCCATCTACCAGGTCAGACGGTCGCATTCAGAGATCTAAAGTTGCCGAATCCGAGCAACTCAGCCGTTGTGAGTTT

>86850

TGCAGGCCTCTGTGGAGGCTGTGGTTGTTGACGTCACCCCTGAGTCACCAACTGTACTCGGTATGTTTTCAAGCATGCTCCTCGTAAGGTGACCTA

>307877

TGCAGAGCAGCGTCCAGGAAAACAGCGCTCTCAAACAGCTGGAGCTCCACAAGCAGCTGGAGCAGAAGCTAGGCGTGGGGTCGCCGCGGGCTCCGT

>91684

TGCAGGTTTATGTTTCTCTTGGGTCTGTAATTTATCAGCTGCTCATCTTTAGAGCTGCACTCGGGTAAATGAGTGACAGCAGAGCGGAGCAAAGTT

>207846

TGCAGCAGAGCCCTGGAGGTTCTGACGGGTCAGAGAGCAGAACCTTCAGCCGCAGCTCCGTCAGGTTGCCGCTCAGCTCCAGGCTGCGCCTCTCGC

>3540

TGCAGGGTAGTTAGCTTCACAAGCTGCTTATCAGCAGCGCAGTCTCACCACACCCACACACACTCAGAGCTGCTGGCATCTCACACAACAGGTCTG

>46607

TGCAGATTGCAGAATGCAGTGGAACAGTTAGTGCGCCGTAGCGCCGCTCAAGGCTTGTTTGTCCAGCTCCAGCCCATTATGCTCTTCTGTAATCTT

>197048

TGCAGCGCCGCACGGCGACAGGGGTCATGGCTACTGGGACGGAGGATGAAGGTTTCCAGGAATCTGACTGGGCCCAAAACATTTCTCTGTAGATTC

>16102

TGCAGTTTTGCTAGTGTCTGGCAAGCATCCTCATATCGACCATATTTGTATAGTTTGTTCTGACATGCAAAAAGACCTCACACAGATTCTAGCTTC

>29703

TGCAGCCATAGCGGTCGGTTGCAGGAGCCCAGCTTCTCCATTACGACTTGTTTTAGCTTTACTACAGTTGGAAATGGGCACTGGGGTTACCGAAGC

>98608

TGCAGGGACCTTCCGTCTTCCAGCTCTTATAGCCGTCCCAAAGGCCACACCAGACCCCCACTGACCACTTCCCCTGCGTCAAACTAATCCTTTACC

>86945

TGCAGTTTACAGCTAGCTTCAAGCGTCCAGCATGCTGATGTTCAGGTGAATTTAGTGTATGCTTGTCGTAGCTCTAGGTCAGTGTTGCTGCTGCTT

>259621

TGCAGCTTTACTTCATCCAGCCGGTTCCGGCACCGTCACGAGCAGCAGAATCACGACGCCACCAGTGCAGAGCTCAGAAATATGAAGCAGACATCT

>117530

TGCAGAGCAAATCTACAGGATCCTTGAAGATGTCCTTGCAGATGGGGCAAAACAAGTCCAGAGATTTTGGAGGCATTTCTTCTGGTTTCCTTGGAA

>50890

TGCAGCAGGAGGTCAAAGTTCATCTGGTTAGATCAGAACCAGTCCTACAATCCGGATCATTTCAACCCTTCACCACTTCCACATGTTATATTTTCA

>262452

TGCAGTTTCAGACTGTTGTCTTTAGATGTCCTCAGTTGCACTTATATGTAGCTGCTCCACCTGTTCTCTGCTTCCAATTAGTTCACCTGCATTTAT

>52721

TGCAGTTTGAACCTGAACAGTTCAGGAATCAAACCGTTTCACTCAAATTGCTTCCAGTTCTGGATGAAGTTTTTGCAGACGATAATCTGCTGTTCC

>172727

TGCAGATGGACATCCGGGATTCAGCACACAGGAAGCCAACACGCCATGTTGCTTTATCCCTGCATGTCCTGCCTTTCCTGTTTTCAGAGCGGCCGA

>285391

TGCAGCATAACAGTAAGTAGGACAATGGTTAGAGCTTGTTTCCATGACAGGCACATCCAGCAGTGTATTTCACTGGCCACCAAAGCTTTTGCTTTC

>139804

TGCAGGGATGCACTCTCACTGAAACACACTGTAATTGTGACACCGTACTGTCATCAGAGCCAAACTCATCTGAGAGACACACTCGGCCGTGTTTCT

>154993

TGCAGCTTTTCGTGCCTTCTGTCCATCTGTTCTCAGTCTCGACTACATCTCTTTATACCTTTGTCTATCACTTGATCTCATACTCTCATCTGCCTC

>221687

TGCAGGAGGTGTTCCGTCGGGCTGCACCAGCGACAAAATAGAAGGTGAAGAGCCTCGCTAAGGGATTTAGATCAGTTAAGATCGGAAGAGCGGTTC

>67358

TGCAGATTATTTCCACGTTTTGCAGTGAAACGTGATCACGGTGTGAACAACCTGGCCAGACACCTGGATATTACATTTTGTTTCATAATTGCCACA

>302264

TGCAGATGTGGCTAAGTTCCCACTTCACCGGGGAATGCGTTCAGTCTTTCTTGAGCTCAGTGAATATTGTCTCCTACGAAGTAACCTCCACTGTGG

>92424

TGCAGATTGTTGTTACTGTGATGGTGCATTGTTGATCAGGAAAAAAATTTTGATTTTCGGGTTTCCAACTGACCAGTCGTCAGTTTGGGCCATTCG

>133368

TGCAGGGAGGCTTCAAGGTGTGAAAGCAACTTTTTCTGAGGCGTCACAATACTTCTCCAGGTCTCCCGTGGGCCCCTCGCTCATCTCTGCGCTCGG

>3882

TGCAGGACCACAACACACACACATATACACACACCCACAGCTGATCACACACAGTGTAGATCATCATTTTTCTGTGTTCACACCCTGTTGCATCAC

>128502

TGCAGCTATCACTTCAACAGAGAAGACGGTGAGCTTGAAGAGCCTGGACCTCATCCTCTGAGCTGAAACAACAATATGATCAACATTCATAGAAAC

>213677

TGCAGGGGGTCGCCCCACTGACCTCTGCTGCCGCAGGGCCGCTCTCTGCGGAACGTCTCCAGGATCGGCTCCAGAAACGCGGTGGGTCGGTTTCCA

>290056

TGCAGAGGGAATCTCTGAATTATCAGGTCTCCAAAAAGAGACGTTGCCTCTCACCCCATTGTTGAATCTAAGCTGCTGTCATTGTTTCGAGAGAGA

>44418

TGCAGCTCCTGGGGTTGATGGTAATTGGTGGTGAAACTCTAGAGAAAGGAGTAAAGGCCAAGATTACAGGGAACGCTAACAAAGACAGCGTCTGTA

>111760

TGCAGTGTGTGGAGGATGAAAAGCAGAGAGCAGCGATTGATCAACAGAACGTGAGCACTGATGATTCAAAGACCCTTTGGCAGGTCTTCAGCGAGT

>310184

TGCAGTGCAACAAACAATAGACTTCCGGTTATGACTGCAAGCTAGATGGTTCCACTGAAATCCTGAGAAAAAGCTGAGATTTCCAATGTGTGTGAT

>175264

TGCAGCCGAGGAAGGAGGGAAGAAAACAAATTAGGAGAGAAGGGAGGAGGATGAAAGAGGGACCTCATGTTGATCAGCCTGGGCAGACATGAATA

>51553

TGCAGAAACGGTCAGAAACAGCTCTGTTCTGCTGATATTCTGACAGTCTATACAACCACGTCAAATATTTCTACCATAGCCAACAAAGTGCACAGC

>77688

TGCAGCTCTGCTGGGTTTGTTCTCCTTTAGATCAGACTGGATGGAAAACAGCAGTTTTCTTCCCACAGAGCCAAAGTTGGATTCAGATCTGGACTT

>248432

TGCAGGCATCACAAGTTGTTCAACAAGGAGCTGTATGCAGACTTCATCGCTGCCCAGATCAAGACGCTGTCCTTCCTGGCCTACATCATCCGGATC

>212626

TGCAGTAGCCTCCTTCCAGATGCGCTGACACTTACGGATATGATGTTGAACCGAAGTGACAGCATTCTGAGGTTCGTCTGAAGGGAGGAGAGGAGG

>216226

TGCAGGACAAACCAGCTGGTGTCTGTTTATGAAGTTCCCAACTCCGCTGCAATGTGTGTGAGTGTGCAACATGTGCGGTACCTGCTGCTGATTCAG

>259556

TGCAGAGTTTGCGTGGGCTGTGAACTCGGTTGTTCTGATTCATGCTGGTGGCAAACGGACATCCACAGTCCTGGACTCCAAACTGCCCTGGACGCC

>1234

TGCAGCAGCAGACCTGCGACATCCGGCAGCATCCCAGTATGCTCCCAAATGGCCGGCCCCTCCCTCCCCAACCAAAGGGCTCAGGAATGGAAACTC

>129767

TGCAGGCGAAGCAGCGGGGCGCTGTGGCAGGGCAGCTGCTGCATCTGGCACATGGAGGCCCCACTGTCACCCACGCTGGGGCTGTACAGGTCAATA

>266547

TGCAGGAAGCGGAATGTTGGCCACTCTGGAACAAAGACTTTTATTTTGGAGGAATCAGGAAGTGCTCACCTCATCAGCTGGTTGTACTTGGCCAGA

>261555

TGCAGCGTTCCCTCCGGATCCCGGTCTGAATCATTCCGACTGGTTCTAGCAGACTCAGTCTGAGTTTGGACTGGGTTTAAGATCGGAAGAGCGGTT

>297457

TGCAGACCGGCCGGCTCTGCGGGAGGAGGTCGGAGCGGATGCTCCGGCATACAAACCCACTGATTTCAGGATGCTGTTCACATGTCGTCTCCTAGC

>283720

TGCAGGTCCAACCACAACAACTGAAGCTGCAACTCCAACCACAACAACTGAAGCACCTACAACAACCACTCCTCCTCCAACCACAACCACTGCCGC

>43319

TGCAGGTGAACCAGGCGGAGAAGCAGAAGCAGGCATGGTGACAGCATGAGGAGAAGAGTCTGTACTTGATATCGGCCGTGGTGCCCGGCGTGGGGT

>190519

TGCAGATTTACTTTCTTCCGTTGAGTGAGTGAATAAAGAAAATGCGTCATCTAGAGAGGATTCCTGAGATTCAAGAGAAGACTGAGAGAAAAGAAA

>298419

TGCAGCGCGACGTTACGACCAAAACGAGCTTCAGATTCAGAGTTTTACTCACAGCTGGAGTCCAGAGAACGAAGCCAGGCAGCGAGCGTCGGCGGC

>296024

TGCAGGAGGAGCTCACTTTTGGCACTTTGTCCACCAGGATATCTCACAGTGCACTCCACCTTCTTGTGGTCTTCTTCTACATTCCAAGTTCGTTCT

>45283

TGCAGGACAGGAAACGGACCTGCTGCTCAGACACGACCCAACAACATGGCTGCCATTGCTCCGGGGTCCCGCGGATGACCCTGGGTGAGACGAGCT

>184836

TGCAGCCTATCCGGTACATTCTAACAATAACCAACAATCATCTAGTTTACGCAAGCCTCTTCAAAGTTCTTTACCAAGGTCTCCACATTGGCTTTG

>18417

TGCAGTGTGCGGGCGCAGGGGAAACTGTTTGTCAGTCCATCTCGAGGTCCATCAGCTTGGTGCGTGAGCGGGGAGTGTGCGCCGTGTTGCGGCAGC

>332502

TGCAGATGAGTGCTTCAAACGTGACATCTTTGCACACGTGATTGAAACATTTAGAAGTGAAGAAGTAGGAAGTTTTTGACATAATCAACATTAGTT

>150956

TGCAGGAAACAAAAAGATGAGGAGGATATGTTTACAGTTCCAGAGAGAAAATGAGGACCAAATATGTCTGTGTAAGCGACACCGAAATGTGCTGAA

>165377

TGCAGAGAGTGCTGCGGTGCGCTGGAATACTACGACAAGGCCTTCGACCGCATCACCACCCGCAACGAGAAGCAGCTGAAGAGCATCAAGAGGATC

>68393

TGCAGCAGCTTCTTCATCTGAGGAAGAGGAGGCTGCGGCTGGCTCCCATCCCGAGGATGAAGGTGGATCAGAAGAAGTTTATCAACTCCAAAGTGA

>185004

TGCAGCTGATCCGGCTGGACCGCTTCTGGTCCAGGTGGTTCTGGTTTCGGTGGCGTTTGCAGCAGATTTTCTGTAAATGTGTCAGGTTTTAGCGGC

>325554

TGCAGGGGAGAGAGCAAAAGGGACAGAACCAGGCAGCACCATGCAAGTGGTTCTGATGTGTCCCTGTGTGGGTAGAACAGGTGCTAAAAGTCCCCC

>58157

TGCAGATTCAAGGAAGAAATTGTGTGGTGCCCTGTAAAACAATTATTGGAACTGTGAAATCTTCCACACTTCATTGTGGTGAATTACACGTCCACT

>157451

TGCAGGAGAGAGCGCTGAGTTTCATTTCATTACCGCCCACAGGCACGGCGGCGGGCATCTGTTCAGAGAAGGGCCCTGTTAGGGGCCCCCCACCCC

>238801

TGCAGCTGTTGGGACCCACCTTGCCCCATGCTGGGTGGGGACGGAGTGGGAACGGCGAGGGGGACCCCGACCCCGCTGCCGCCGCTGTTCTCCCGG

>103244

TGCAGGCCTTCAGCCATCGATGATGCAGTTCAGTTGGAGACCAGATAGAAATAAAGGTGAGAAATTTGATGGGGACATTTTTAGATGTAACACCTC

>216989

TGCAGATTAGTCACAAAAATCTGCACAAAATGCAGAAAATAAACCAAACCAGAAGCAAAAAGAAGCTCAGAGGAGCATCCAGACCCCATTCAGGCA

>274387

TGCAGCCGGGAGGAAGGAGGGAGGAAGAGGAGGAGAGAGGGGGAAGGGCGGCAGGCGGAGGCTCCCCTCCCCCCATCAGAGGGAGGAGAGTCTGGA

>65971

TGCAGCAGCAGCACTGTAAAAATCCAAAGCTCCCAAACTGCTGTCACCATCAGCGTGATGCAGCGTGGCTCGTTTCCATCCAGATGGGCACAGCTT

>131982

TGCAGAATGAAGCAAGAGACTGTAGAATGAAGCAAGAGACTGTAGAATGAAGCAAGAGACTGTAGAATGAAGCAAGAGACTGTAGAATGAAGCAAG

>282420

TGCAGCATAACTGGCTGGATTTTATTGGCCCAGCAGACGGCTTGGACCCCTGGGAGGAGACCATCAGATGATATCTGTTAAGATCGGAAGAGCGGT

>58532

TGCAGACACGGGTCAGACTCTGCTGGCAGACATGTCTAGATATTCAGTTGTGGTTTTACAGTTTTCACTGGTAAAAAACAAAAAAAGCAACAGCAA

>167256

TGCAGAAGCTCCAAACGATGCAGTTCAGAGTGTGTGTTATGCAAAAGCTGCACTAGATGAAGCCTGGAGTGTGTACTACGCAGAAGCTGCAAAAGA

>342615

TGCAGCATTAGCCTCATGACAGACAGCAGTCCTGTGGATTCTCCTCCGTGTGCGTGTCTGTGAGCCTCCGTCTAAGAAGGTTAAGATCGGAAGAGC

>216680

TGCAGCTAATAATAGCAGCAGCAGAGCAAACTGTTCCCGTTTGACCTGGAGCGAATCGTTTCCTCTGTTTGCTTCTCGTCTGTTTGGACTTTCAGG

>299472

TGCAGTGCAGAAGCCACACTTGTTTTGGTGGTGGGGGGGTTTATTCCTCTGGAAATGGTGCATTCAAGCAGCAGAAGCGATACGCTCACAGCTGAA

>260476

TGCAGCCGGGGAGCCCAATCCGCGCTGCGGTTCTGATCCGGGTCGGCACCAAGCAGAGGTCCTCTCTGCTGCGGCGTCACCAGACTGTTATTAAGA

>40413

TGCAGCGGCTCGGTTGTCGGATTAGAGACTGAAGTGGCGTCCTTCACTGCTGTTCCTGCGAGCAAAGTGGCCATTATGCCTCCGCGTCCCTCCTCC

>310314

TGCAGGCGGTGAGGGTAGTAGGACAGCCGGAACTTACTGAACAGAACCAGAACAGGGATTACTCTGGACGGGTCTGACTCGTTTGCTCACCACCTG

>170207

TGCAGAGGAGGAAGAGGAGAATGTTGTGTGATGCTGATGCAGAGAGAGAGGTGGAGAATTTAGACCTGGAGCAGGAATGCAGGAGGAATGTAAGGA

>242540

TGCAGAGATGGCAGAGAAAGGAGCTGGTCCAGTAGTTATCAAAAGCAGCAAACGGCCCAAATCAGAAGGTAAGCTCATCTTCTTCTGTATCTACAA

>169063

TGCAGACCAGAAGACGCTCTTCTACATCGACAGCTTGGCTATGAGCGTCGATGCCTTCGACTACGACTCGGCCTCTGGAAACCTGGGTAACCACGC

>283325

TGCAGGGTGACATCATCAGGCAGGAGCGGAGCAACAGGAAGTTGGAGCAGCTAATACTGGAAACCCAAAACGGCGACGCAGCCGACCAGAGCGAGC

>187447

TGCAGTAATTATTTCACAGAACAGCACAGCCTGGTAGTTCACATGTTTGGCAGCCTGAGATGCACATAAAGAAAAAATTCCATCCGTCTTGTTTTC

>114640

TGCAGATCGTGGCAACAGGAGTGTGTCAGTCGGATCTGTACTTCCTGTACAAATGTCCGAATCAGACGGCGTTCCCGGCGGTTTTTGGCCATGAAG

>322232

TGCAGGAGCCTGAACGCAGAAGCTGAACCCATTCTGGGAGGACGGATCTGGGTTGGAGGGTTTTTTTATGTGGATCAAAATATGATCAAATACCCT

>302415

TGCAGGGAGAGAAACCCAAACGCCAACAAACTGCCGCCACTTGTTGTACAAACACAGTGCATGTGGTGGATATTTTAGAGCATGCACCGGCTAATA

>280048

TGCAGATCGTAAGATCACAGCCCTGGAAAGCAGATTACAGCTAATCACCACAGACACTTAGATCACCGTTCTGATCAAATAACAGCTCAGCTGTAA

>172522

TGCAGGCAGATGTACTGTGGCAGATGGGACACACAGGTGCGAAAGTGACTTGTACTTTCAAGGAAAAAGAAACATAATAATCTTTGCATTTCATTC

>228264

TGCAGGTGAGTTTGAGAAACGTCCGAGCGTTGGCGTCCAACCAGGCCCATATTGAATGGCACCTAACAGTTTTAAGATCGGAAGAGCGGTTCAGCA

>211316

TGCAGCCGGCCCAGACATCCAGGCAGTTCTTCTCCGGTCGCAGACACGTTCTCTAGGATCGCTGTCCCGTCTCTCTTTAAGATCGGAAGAGCGGTT

>143355

TGCAGCTCCAACCACAACAACTGAAGCTACAACCACAACAACTGAAACACCAACAACCACAGCTTCTCCAACCACAACAACTGAAGCACCAACAAC

>305709

TGCAGCGACTGCTGGAGATCCAGCAGAGGAGGAAGGAGCGTCACAACCTCCAGAGGAGGTTCATGAGGCTTGGGAGGAAGCTGGACTAGGTGAGGA

>152270

TGCAGCTGAGGGGCTTCCGTGGTGGGCTCTGCCCAGCCCAGTCTGATCATCAGACCCGCTGCTGATCATCAGACTGAGGGACGGGAGGAAGGACCG

>234992

TGCAGGATGGGGCAGTAGATTGCATGGTTGAAACAAAGGCTGCATATTCTGTTTGTCCTCTCTGGGATCTCTGGGATCTCTGGGATCTCTGGGATC

>210565

TGCAGAAGATGGTCGGCTAACACCTGTGGCTCGAGGCGGTTGTGAGCACCGGACAGATGTCTGTCCACCCTCGGACCATTGTATCCACACGCTCTG

>190890

TGCAGAACTCTGGCCTGTCTCTCTCAGGTTTGCTTCCGTACTGCATGCGGGGGTGCAATAAGAGGTGAGGGGAGGTCAGTCATGTCTTTTCACGTT

>187832

TGCAGACTGGAGCTGAGTTCAAGTGCAGCTTCACAAACCGACCAATCAGATCCTCCGGTGGTCTGGAAGAACTTTATGAAAGTCATTTCAGTTCAT

>106952

TGCAGCTGCCTAGTGATGCTTTGTGTTTTTATTGAGTAATTTCACAATATCTGACAGATTATCTGTTTTGTACTATATTGTCATGTACAAGAGTAA

>293426

TGCAGCTAAAAACGCACAGAAGAGCGCGGAGGCCAGAGAGGAACGGGGTGTTACTCACCCGGACAGGAAGGACCTGGTGACGCCATCAGCAGGGAA

>165387

TGCAGAGTGTCATCAATCAGAGTCAAGATGTATTGGACTGTCTGCTCCTTGGAGATGTGGGCCATCAGGTTCAGAAATGTCTTTGCACACTGGAAA

>266712

TGCAGATGTTCTGCTGCTCTTCACTCACCACAGAGTTTTAGGCAGGTCCAGCCTATCAGCGCTCCTTTAGGATAGGTGTTAAGATCGGAAGAGCGG

>61783

TGCAGCCGCTTTGACAGTGGTGGGGTAGTTTTGACAATGTTTCTTTGAGACTTTGGTAGGTGATGTAAAAATGTTTGGTTTCTCATGTTTCTTGCA

>16181

TGCAGCTCCAACCACAACAACGGAAGCACCAACAACAACCACTACAGCTCCAACCACAACAACTGAAGCACCAACAACAACCACTGCAGCTCCAAC

>76352

TGCAGCACGCTCAGCTCCTGGTTGGTGAAATGTGAGCGGATTTGGCTGAGGTACGTCATAACGATGAGCCGGTCAGGGACGGACAGCAGAACCATG

>156769

TGCAGTGGATCTGTGTCAGGAGCAGAGCCTGTGTGTCGTGACTCTTCTCCAGCGGTCAGTGGTTCGTAAATTTACGGATAAATCTCTGTTGTTCCA

>204698

TGCAGTATTTGATCTTGCCAACGAGGATCTCGTCAAGGAAGGGGTGGAAGACGACAAACCTGAAGTGGACTAAAATAAAAAAATAAATAAAAAAAG

>206225

TGCAGAGGAAGCTGTCGGAGCCGCGGCATCAGTTCGCCGACCCGCAGTACCAGCAGCAGTGCCACGACCTGAGCGAGCGTCTGGAGGACTCGGTTC

>105352

TGCAGCAGTCAAGTGCCTATTTGGACTATGGGCGTAAGGAAATAACTCTGTTTGGAGAGAAGGTTCCCCAGTTGGGTTTTGACCTCCAGGCTGGGG

>184724

TGCAGAACTACTCGAGTAGGAAGACTGCAAACACACAACGACTTACAGAACCGGATCACTACACACCGACGGGAAACAGGGCCACGTTCTCAGGGG

>204154

TGCAGATGTGTGGAGGGAAGTCCAGGATGCCATTGCTGTAGTTTTGAAGTTACAAAGGGGTAGATAATTATTTGAGCTGTGGAATCAGAGACAAAG

>2103

TGCAGTTATATTAGACTGCTAGAGGGTGAACCACACATCTGAACCACTAACAGCTGAACACAAGCATTAGCTCCCCACAGCATTCAGCAGAAGAAG

>311220

TGCAGCCAAAGCTAAGGTCAAAGGAAATGAGCCACTAGGAAACTCCTGGGACCCAACAGGAATCAGAATCTGTTGCTGGGCCCCATAAGCACAGCA

>75822

TGCAGCGTGTGAAGGTCCAAAGCTTTTCACCAAATTACTGTTTCACTGCGAGAAAGACTTTTAGTTCTCACTTTACTTTTGGTTTGATTCAACTAA

>86498

TGCAGGTCAGACCCAGAACCGAGTCCCCAGCAGAAAAACTGGGAGACCACTTAGAAGGACCAGGACAGAGAGGGCTGATGACCTGCCCTGTTTGTG

>175876

TGCAGAGCAAACATGCCTCATAAAGACATGCTAACGCTTTTCTGTAAAGGGATTTGATTTATTCTGAAAGCCACTTTACTATCATTGGTGATAATA

>90392

TGCAGATAGAAACAGCAACCATGGCAGATTCAGGCCTCTACTACTGCGCTCTAGGAACACAGTGGTGCAACATGTAGAGGAGGCTGTACAAAAACC

>266873

TGCAGAGTCAGATCTGTCATGTGAGTCACCTCAACTTCAACACACGATCAGATCATCACTGAAAACAGAGTCAGTAGCATCAAAACGGAGATTCTG

>209482

TGCAGTGTGGGAGATAATGAATAGGCTGGGTCAGGGTCTCATCGGTGCTCTACATCTCACCCTATGCTTCTTTAGCCTGGGATCTGCTGCTCCCAT

>67642

TGCAGCAATGCCGGTACTGCAAGTGGCTTATATGGCACAGGTGTTTGTATCACAGAAGATAAATCAAGAAGATACAATTATTACAGTTCAATTCAG

>60190

TGCAGTTTTACAGTGCACTGATTTATACCCGCTCTGCGTTCACTCGCCGCTTTTTTCCTTTGTTTCTCACTCGTTGCCGCTGCCGGGGAAGCTTTG

>25325

TGCAGCGGCTGAACCGGACCCACCTCCTGCCTCGGCGCCGCAGCTCGTTCAGCAGCGTCACAACCTGCCGGGCGCCGGTGCAGCCCAGAGGGTGAC

>41193

TGCAGTTAGGAACCCAGTTGGGCTTTTGCCCCCGAGTCACAGTGAAACGATGAGGGTTTGTGTCTAAAGCAGGAGCAGCATCACGACACAAGCATG

>340855

TGCAGGAAATTTCTCAGCCTTGCCCTGCTTTCATTGCCTTTTGTTCAGGGGTCCTCTATAGCTGTGAAGAAATTATAAACAGCAGCCACTGCATTT

>73308

TGCAGGACGTCCGACACCACGATGGACGTCTCGCCCGTGTCCTCGTTCGGGTTGTCCCGTCCTCCTGAAACCGCGATCCCAAAACCCATTTTGGAA

>29354

TGCAGACGGTTTCTTTGCTATTGGTAAGAAGCAAAATAAAAATGCAACGAACGTAGCAAACATGGCAACACTCATGTAAAGGTTGGTCACAAAGTT

>133091

TGCAGCAGAGCTGACAGTCTTTTCAGATCGAGCAGGCATGCAACAAGCGCTCATGCTCACCTACCGATGGTTCATGAATTACATGGCGACATCGCC

>329228

TGCAGCTACCACCATGTTTCATGGTGGCTGCGGTGCGTTCTTAGTAGTGTCAACTATCAGATCTCTGGATAGTTCGACCTCTGGAGAAGCAAAACT

>280776

TGCAGAAACTAATTATTCATAACATAAGATGAGGGTAGTGTGTTAGACTGACTTGATGTCAAATAAGCACCTTTCAGTATTGTAAAAGCAACTCTG

>262962

TGCAGCAACTTCCACGTACGGATATGAGAGGATCCTCCAACAGAGATCCTGTAGAGGACAGATGTCTCACCTGGACCCATTCCTGACTCCACTGAG

>33804

TGCAGGGGTTCTAGGTGGTTCTGGACGGGTTCTGGAGGGGTGTGGACAGAACAAATATGATTTCCTGAGCTGCGGCTGAGAGTTTTCTTCTGAGAC

>230556

TGCAGACCAATGACCCAAACGGTCGGTCCAGTTGATGGCGAACAGAAGAGGCGGCTTCATCGCAAGCTGATTTCATAGAAAGCTTTGTTCTTTCCG

>148257

TGCAGGGCTGGAGCTCCGGGTGAGACAGCTCCGCCCGCAGCCGCTGCTGACACACCGGAAAATAAACCGGCAGGAACCGAACCTACAGAACCGAGC

>337360

TGCAGCAACGTTACAGCAAATCAAAATGTTCAGACTGGAAGTGTAAGTCAGAAGAATAAAGATTCCAGCCTGACAGGCAGGAAACCATGAGGCCTT

>171544

TGCAGGAAGAGGCCGGGTCCAGAATCAGGCTTTAGTGACCAGTAGCTGGTCCCAGTGCTCCCAGTTTGCAGCGCTGTGGTTCTGTGAGGGTTTCTG

>110958

TGCAGAGAGCAAACACAGCAGCAGGTTGAAAGGAGATTTGCTCCCGACTCCTGGGTCAGGGTCAAAGGTCAGCGCTGTTTGATGAAGGCCTGACTG

>139161

TGCAGAACCGTGACCTTTGGTGACCTTGACCTTTGGTGCTAGCATGGCTTATCAGGAAACTCCACCTGACCTGATTGGGCCCTCTGTCGGACCTGT

>264065

TGCAGCAGAGTGAGATGCAGCAGGGTGAGATGCAGCAGAGTGAGATGCAGCAGGGTGAGATGCAGCAGGGTGAGATGCAGCAGGGTGAGATGCAGC

>206581

TGCAGATCTGGGGTCAAAGTTCACACAACATGCGGATTCTGTTTGCTTAGGTTCGGCCAGACATTCAGTGCCGAGCAGCCGGCTTTAGATAAGGAA

>190625

TGCAGCAGGCGTCCATGTTTCTCAACTACATGTGGCAGAAAATGCGAGTAGCTGGAGCCTCATCTGGGTGAGCTGTTTCCCTCCGCAGTTTGAAAT

>259455

TGCAGGTGATTCTCAAAACCCTCAACCTTGGTTTCATTTATCCAAATAACATTGTACCAGAACCTCATCGTTGCAAACCCAAGTTGTGCTGCCATG

>239521

TGCAGAATCATACATGAAGCTGTCAGAGTGAGGACGATTGTTCATTTGCGTGAAGCTGCCTCCCCCCAGGAACGTAGCCTTGGTTTTCCTTGGCAC

>30674

TGCAGCCGTGGGATAGTCAGGACACCGGCAGCCTGTGGCAGGTCGTAGCAGATACTAGCAGCAGTAAGATTGCAACCCCACACCTCCCCCCTGCGA

>284259

TGCAGCAACATCCAATCAGACGAGCCGATCTCAGGATTTAGAGACTTTCTCTGAACTTGAAACATTTGAGGGCAAAAGTTCTGGTCCTTCAGAACC

>199236

TGCAGTTTATACTGGCAACGGCTCACTAGTGAAGCCTGATTCAGAGAACATCTACACCCAAACAGAAAATACGTTAGCTACAACCTAAATATCTGA

>73853

TGCAGGCTGCTTGCGCTGACTGGTGAGTTTATGACACAATGTTCGTGTCAAAATCATTCCGGATCATAATCTCACGATAAAGGTTTTTTGACTCCA

>160453

TGCAGTGCGAGCACCACCCGACCTGGTACAGCGACACACTGAAGCAGAAGACCACCTGGACCTGCCTGCTGGACGGCATGCAGTACTTTGCCACCA

>33364

TGCAGTCCGGACGCAGATGAAGAGTTCCCATGGAAGTTTGTCCAGTGTTGGATGGGATTGGTGGGCTGGGTTGAGCAGAAAGGTGGCGCCCTCTGG

>154090

TGCAGCGCCTCGCTTTGGGTTTTCAGGTTAGAAGTGGATATTTGTTGTTGCCACTAGAGAAGCTTGAAGTCGTTATGTTCACGTTGTAAGTCCTGA

>271288

TGCAGACAGGCGGCTCTCACAGGAGTCATTAGCTTTACGGGCCACAGCTGACTGCTGCCTGATGACATGCAGCTTCTTTTTTATGTTAAGATCGGA

>665

TGCAGGGAGCTGCTGTATGTCTGGTAGGCAGGAGAGCGGTAACTAACCCGGGGCGACTCCTGGTCGGGCGGCAGCCCGTTCTGGGACACCGGCTCC

>139068

TGCAGCCGACTCCATCGGCACCGCGGAGTGGTGATCACTCGACTCCATCCCATCATGACCAGCAATAAAAAACTATGGAAACTGTAGGAGCCGTTT

>240868

TGCAGCCGCTCTCGCTTTGTGTTCCCCTACAATTATACTCGCTTTGAATGGCAGAGAGGAGGCGAGGGGAGAAGGGGGCGGCACGGAGATCTGAGA

>285341

TGCAGATAAAAACAATCAGAAACCGTTCAAACCGCGGAAACCTCTGGCAAATCATATGGTACGTAACAGCTACTGGATCTCACTGGATTAGCAGAT

>302840

TGCAGCAGCTTGTCCCGAGCCGATGATCGTAGAAACCGAACCAGGATGGTTCGGGGTGGTTTATTGGCCTCTGGTTTGGGCGCCAGTGAGCGATGG

>164106

TGCAGGCAAAGCAGTAGGAGGAGTCTTTACTAACAGAATATTCTAACCAAGAATAGTCTTTGTACCAGGAGCTGTGAAAAGTTCTCTTTCTTGTTC

>179338

TGCAGACATTTTTACAAAACAACAAAAGCGCAGAGACGTGGAGAAAACGCACAAAGACACAGAAAACAACGAAACACACAGAAAATGTGGAAACAC

>128676

TGCAGCTCCGGTGACGTCACGCGCGGCCCCTGCGTGTGAATCGAGGCTTTGAGGTTCAGCTGAGCAGCAGAACCGGCAGGAAGGAGGAATTTATTG

>36098

TGCAGTCAGACAAAGATCACTGAGCTTCAACAATCAGGCCTACTGATGTCTCATGGCAGAGCTGCTGCGTATTACTCACCTGTTGCTGGATGATAA

>336893

TGCAGCTCCCTCTTATCTCAGTCCACAGCCTGGAGATTAGCCCGAGACGTGAGGCTGAGATTCAATACGCCAATATGTCAAAATGAATAGTAGTCG

>340725

TGCAGGTTGCTCACATCAAGGGCTCCCTGCAACAGAGACTCGCTCACAAAATGACATCTTCATGCAGACAGACAGCAATTTAAGATCGGAAGAGCG

>152745

TGCAGGACCCCCGCTCCAACACTAAGCATTGACTACACTCTTGAAATGTCAATACAATGGTCTTCCTGACCAAAATCCACCCAAAAGACCCGATTT

>194436

TGCAGAGTTGTCCGTCCCAGGAGACGGCGTATGCGTGCCGCACCCCTCCAATCTACGGATCAAATCGTTCGACTCTCTGAACACTCAAAGTCGCTC

>196125

TGCAGGAAACACTGAAAATCCATTTTGAAATGATAGCCGAAAGCTTTTTGCTTACATTTCGACTGGTAATGGAGATCAAAGATCAATGTTGAGCAC

>297824

TGCAGCCCAGAGGGAGGAGCTTAGCCTGGAGGAGGCGGGGCCTGTCGGTGACGTCACATGGCGGCTGCTGAGGCGTCTCCATGGTAACAGGAGAGG

>139845

TGCAGCGGCGGTCTGAGACCTGGAGAAAGCTCTGAAACCCGCCGCAGGTTTTGGTTTCTCCTGCCATGAAAATGTTCCAGGGTTGCTGCGTTGTAA

>38582

TGCAGTGTGAACGTCCAGACGGAGAGTCCTGAGCCGTCGTCGTCATGGCGATCCCTCATCCCAGTGATCAAGCTGAACATCAGCACAGTGAGCGAC

>313727

TGCAGGAAAATCTCTAACTATGAATGAAACTCTGGATGTCTACAATGCAGAGGAGCACTCAAACGTCACACTGACATGGATCCGCCTTGTAAAAAC

>145890

TGCAGGGCGGAAATGTGTCTGTACCAGCGCAGAGCGTGGACGTGCTGCGGGGCGGGGGGGCGAGGGAGGAGCCTAAAGGCTCTCAGGTCGGCCTGG

>222439

TGCAGGTTTTCTCTGCTGGTTGTTTCCCTCATCTGGTTTTCCTGCTGGAATCCCTTTACCTGTTCCAGACAGGTGGACGTTGGTGACCGTTTCTCA

>312518

TGCAGATAAAACCTACTAACTGTTGTCTTACTAAGATTATTATGTAATTTATGCCCTGTGAACCTCTGGACTTAGATAAACTAATACTGAGGAAGT

>188529

TGCAGCGCAGCTCTCAGCTGGAGGGGCGACAGGTAAAAGGTGTGAGAGAAGCCGTTGCATCACGAGTGAGGTTCAGGTCACTTTGTTTATCCCACA

>75541

TGCAGTGGGCCGGACAGTGCAGAGCTTCCAGAACTACTCTTGCTGGAGCAGCACTAGTGAAATTGGAAGAGGGAATAAGGAGGACATTGATTCAGC

>223826

TGCAGCTCTTCTCCATCGTCCTCATCATCTTCATCTGTGAGACGGCAGCTGGAGTCGTGGCTCTGCTGTACTCCTCCTTCGTGAGTTTATTAAGAT

>44092

TGCAGCAGCTGGAAGCGGGAGTGGACCTGGATCAACCAGTCCACCAGGATCGCCCTCATGGCTGACGTGACCTCCGACCCCTTCAGGTAGTCGGCC

>41498

TGCAGGACCTGAGCCGCCTGCGGCCGCTTCCCTCTGTACTGCTCCAGGAGGAAGTTCTGCCGGGCCCGTTTGATGATCTGAACCGTGAAGAACCAG

>102742

TGCAGCTGCTGTGGTTTCTCTCCACCTAGTGGCGGACAGCTGGTATTACAGCTCAGCTGATGTTTTCATTCTCCTTCAGGTCTCAGAGTCTCTCTG

>134020

TGCAGCTTTCCCTGAACCTCACCGTCTCGTTCTTTTGTCTGCTGTGACGATTCAGCTGCAAACAACAGAAATGACGACGTTTCGCTTCTCGGTGAG

>47611

TGCAGACAAGCTGCCCTCCTCTCAGCCGGCCAATCGCGTCCCGCCACCTCACAGGGCCTCAATGCCTCCCCCCAAGGTCGTCATAGCAACCCTGAA

>325291

TGCAGGAACAGCCCAGAGGGGTGCTGTACAAAAACATGGTGAGAGTTTCTCAGAGTGTGCTCAGCATGATGAAAAAACAACATTCTTTTACATCCC

>148283

TGCAGCACCTGCTCGGGCCCAAGCTGGAGCGCATCCAGATGATGGGCAACAAGCTGGAGCCGAAGCAGACGCCGTGCTCCCAGCCGACCACCGCCG

>169748

TGCAGAGACGTTGGACCTTCTTTCACTGCGGCTGTCTGTGTATTTTCACTTCATCGTGTTGACCTGCGTCACAGAATCCCGATAAAACACGTTTCC

>121073

TGCAGAATGTCCGATTAGGAGCCCCTTACTTGCGATAGGACAGTGTTTTCATTCTCAACACGTCACCACTTTGTCCGTCAGCATGTCAGTACATCA

>62004

TGCAGGTCTGCGGGTCTGAGCGGAGAGCCGGAGCAATGCATGCTGGGACAGCTGAGACATGACTGGGAGCGCTGGGAGGAGCCGGCGGACGGCCGG

>193695

TGCAGGAACCAGGAGAGCTCCTGATGAGAGCTGACCTGACCCAACTAACACTCGGTACCGGGTCAGAACCAGCTGCTGGATGTTCTACAACCAAAC

>31664

TGCAGACCAGAACCGGCCCGGTCCACTTTCCCCAGACGTTGGCCGGCTGCTTGCGTCGCCATGGAGACGCGTGTGATCGGCGTCTCGGTGATCTGT

>36536

TGCAGTTCTCTCCTGAAATAGACACGTTTTGACTTTTAGATCAAACTAAATGTGTTCGGACCCTTAGGTACTTGGAGGCCAAGAAGGACAGAGTGA

>200585

TGCAGTTCCTCAAATGCAAACTCAACAGAACCTGCATCAAAAGTAGTTTTCCTATTTACTTGATCATGAACAAATCATTTTTGTTTGTTGATTTGG

>323664

TGCAGTTATAATCCCAATATTTGTCGAGGGCCCCCAAGTGCCAATCCTCGGGTCCCAATTATTTGCCCTGCTGTCTCCGTCTCTGTTGCTTCTCTG

>12082

TGCAGTGTGTGTGTGTGTGTGTGTGTGTGTGTGTGTGTGTGTGTGTGTGTGTGTGTGTGTGTGTGTGTGTGTGTGTGTGTGTGTGTGTGTGTGTGT

>328608

TGCAGGCACCAGTTTAGGCTGCTTTGTCTACTTTTCCCATGACTGTTGCACGGGAGAGTGCTGCTCCCCATTAAGATCGGAAGAGCGGTTCAGCAG

>94461

TGCAGATGGACAGCAGGAAGTGCAGCAGGACGTCCTCAGTGTCTCCGTCTGTCCCCTGGAAGAGACACAGAGGACAGAGACTCACCTGACAGGTAG

>285902

TGCAGCAGAACCAGGCGCAGCTACGATTACACGACGTCAGCAGAACATCCAACTCAAACACACACTGGATTAAGATCGGAAGAGCGGTTCAGCAGG

>186771

TGCAGTGGACGCCATCATGTGGAACCTCTGTGTTTCTCTGCCATCGTTTCACTGATGCTTATCTAAACAGCGCAACGCATTCTTCCTCACGCTGCT

>40682

TGCAGCAGAACAGGAAGTAGGCAAGCAGAGGCAGGAAGTAGGCAGGCGCCAGGCAGGAAGTAGGCAGGCGCCAGGCAGGAAGTAGGCAGGCGCCAG

>10233

TGCAGGGCTCCGCGCGACTCCTCCAGAGAACGCAGATGCTCGTTGATATAAGAAACTAACAAACGCTGCGAACACAACAGAAATATATGCCTTGAG

>310509

TGCAGCATATAAAATTGATGGGCTCATTCACAATGTGCTCCCAAGTCATTGCCTCATCACACTGATGTACATACACTTTACCTGAAAGAAGAAAAG

>335308

TGCAGCGCCGCTGAGCCGTAACCTTACGCCAACCAGCTTTTCCCTCAACATCCATTCAAACACGGCTTGTTTCCTCAATTGATTGAGCTCTTTACA

>276601

TGCAGTGATTACAAGCTTCTTTGGAATTGAACAGCAGCTTTATTGTTCTCTCTGGTGGGAAACATGTTGGTGATTTGAGCTAAAATTCAGCAGAAC

>75575

TGCAGCCACCGGAGCCCCGTCTCCTGCAGCTTCTCCGCCGGGCCAGACCGACTCTGATTCCCCTGCAACATCAACCTCCACAAGGCCAGTCAGAGG

>248890

TGCAGACATCCCAGAACCGAACGGATCAGACGCAGCTGCTGCTGGAGAACCTTTCAGACGGTTCTGTCCTCACCCGTAACCGTTGGAGACGATTTT

>125667

TGCAGACGAAACGCCGCGAGCGTCGGCAACATTCCCTCCTCTGAAAATATTCCCCTTTTCCGTTGTGAAGGTAAGAGACGTTGCCGACTTCCTGTC

>274997

TGCAGATAACTGAAGGCTTCCGACGACTTCTTGAAAGCAGAAGATGCTCCCAGCAGTGGAATGACGAGGCCTTTTATCTCAAGTACAGAAACGGGG

>104210

TGCAGGAGCCCCTGCACTGGTTGATGCTCACCATGGGCCTCCCAGGACTACCCACAGAGGAGGAGGCAATAAAGGCCTGGGGAAGGAGCCATAAAT

>138301

TGCAGGTGGGGCGGTACCTGCAACTTAGCTAGCGCAGCGCGTCTGTTTGTCGTTCGTCGCTGTTTCTAACTTACTCTGAGATGTGAACGAAGATGT

>26829

TGCAGAGTGTTCTCTTTCCGATCGGACCTACGAACCGCAGATGGAAAATGTAATAATGAAATAGGTTGATGCATCACGATGATTCCCTGTGTCTGG

>127566

TGCAGCCGAACCTCGGTGACAAGAATCCGGGGGGGTTCGCAGAAAGCGCACAGTGTTATTATTAGACAGGAAGGGAGGAAAATAGCCGAATATCAC

>171185

TGCAGGTAGTTCTGGTAGCCACTGGTGTTCTTCCCATACTGGATCTGCTTCTGGCGCCGCTTCAGGACTGCTGCGTTCGTCTCGGTGTTGGCCGGG

>171763

TGCAGGACACAGGCGCCTTCTTCGCTGCGTCGCAGGCGAAAAGACCCCCAAAGCTGGGACAGATCGGCAGAGCCAAGCACGGTAAAGGATGCTCTT

>229508

TGCAGAACGCATCAGGGCTGAAGGTACCGATCCAAATGGACTGAAGCCGCTTCTCTTTGCTGTGCTCATTGCACTACAACAAAGGGTTAAGATCGG

>29501

TGCAGCAGCCTGCTGAATGTGTTCAGGCTGGATCACTAACATCCAACAGTCCTTATTGTTTTCCTGGCTGCTGCCATCTGTTTGTTCCAGCACAGC

>60979

TGCAGCTCTCAAAGCCCCGGCCTGCTGCTGTAAATCACCGGTTTCATCGCAGCCGTTTTCCTTTTATTGCTCCATGGAAAGAAATTGTTGGGTTGG

>226490

TGCAGCAGGAGGAGGAGGAAGGAGACCAGAGGGCACTCCAGCCGTCCTATCGTTTCCAGAGAGACGATATGAGGAACAGATTTATCCCTTTAAGAT

>253582

TGCAGGTTTTTCCACAAACAACAGCAGTGAGGACAGGTGAAATTACTTATTTTAGACAGGGATCAATCCTTGAATCTCCCATGGCAACCGTTTGGC

>2627

TGCAGCGTGTGTGCGTCTGTGTGTAAGTGCGTCTGTGTGTAGGTGCATGTGTGTACCTGTTCGGCCAGGCCCTGCTGTGTGCCCCAGTTTGTGTGT

>196409

TGCAGAACCACCCGGAGCACATGTAGCCTCCAACTGGCTGCAACCTCAACCTCCCCCCACTAGAGGGTGGCAGAGATCCACAAGACAAGCGCACAA

>114498

TGCAGTTAGCAAACCTGTCACAATGAATAAAAGCAGGCCTCCTTTTGCAGGTGAGTCTTGGCTTACCTTTGTGCAAAGAACATCTTGCAGCTCCCT

>202147

TGCAGCTTCGTGTTTTCTGCTAAACGCTGTGGTCCTCTGGGTCGGTCTACATCAGGGTCTCTGTTCAGGACGCCGTGACATTAAGATCGGAAGAGC

>147783

TGCAGAAAGCTGAGTGAGGCCGAATGGATGGCGCTGATGATTCAGAGCCGGCCTGAAGGAGAGGACGCTCCGGTTTCACCACTAATGCAGCGGCCA

>224465

TGCAGCTTGACTTCATAAAGTCAGTTATTTGGTGATTTATGGTGTCAATCAGGAGAAGCATTCATTAGGAGACCTAGCAAGCTGGCCCCATGGCAG

>177601

TGCAGGTGTCTGTCTGTCTGTCTGTCTGTCTGTCTGTCTGTCTGTCTGTCTGTCTGTCTGTCTGTCTGTCTGTCTGTCTGTCTGTCTGTCTGTCTG

>329187

TGCAGGAATCTTTTCCAGGTATGTAAACTTCTGGTCTCACAGGTCAGGAAGGCGTTGAGGTTCTGGACGAAGCTGTTCCAGCTCTCTGGGTCCGAC

>289814

TGCAGTTTGAACGTGACGTGATGATCTGGAACAACAAGACGTATATTTCCAAGCCGCTGCTAGTGAAGGAGGATGCTGCGGTTCGGAGGCACCGCC

>9686

TGCAGCCTTTACCTTGTTCGCTGAAGAGCAAAGAGTGATGACGTGAGTTTATCTGAGAGTGCGTTTGTCCTGTTTGTCTGCTGTCATGCCACTCTG

>140315

TGCAGAGAAACAGGGAATGAGACCGGCCCGGTTTGGACCCAATGCTTGGACCTGAGGTTCTGGTTCTGGTACCTCAGCAGTGGAAATGTTCTTCTC

>237434

TGCAGCACAAGAAAGGCCGTTTTTTTATTACTTCTCCCCTCGAAGAGACTTGGAGAGCGTTGGCTGTTTGCACTGCGAAACAGGATTATACCTAGA

>282208

TGCAGATAGTGACAGTGGCAACCGCACTAGCATGCTAGGTGACCTTTGACCTGTCAATAAAGTTTGTGCACCTTATGTCTGGTGATCAGACATGAG

>155723

TGCAGCAATGTGCTTTCAACCAAAGTAGAGATGTTTGGCTGTCATTCAGTGTGTTTTCTAGAAAACCACACAGCACATCAGTTCAAACATCTCATG

>316511

TGCAGGTTTCTGGTTTCAGACATTCAGAGCAGAGAAACTGGAACCACCAGCAGGTTTTATTCACCTTCCTCCTCCTCCTCTTCCTCCCCGGAGGCT

>178790

TGCAGCTCCCTCCAGGAGGTGAAGTGGCTTCAAACCACACAAGAACTAATAACTCCACTAGATAACGCAGATCAGGGGTCTGAGATTTTTACTTTG

>3744

TGCAGGGGTCAAAGGTCAGGGGGGCAGGAAGGAGGTGAGACCAACTAGCAACCAGAAACTCTAAAAATCGGATAGAAAAGCTCAAAATGTTTAGAC

>141236

TGCAGGTGCATCGGGCCACGGCTCCAGGACTGTGTGGGGATGATGGACAGGTAAGGCATCTCCTGGTGCATTTTGGGAAGCGGGGACCAGGGGGAG

>336571

TGCAGAGGCAGATGAGGACGCCATGCTGATCACCTGGCCGGGCTTATCGACGATAATGTACGGGTTGTTGAGGACAGAGGAGGTGCTCTGCTTGCT

>126723

TGCAGCTGATCTAGTGACTGATATTTTAGATGGCTTGTCACCAGGCCTTTGTAGAGCTGCGTTACTGCTAATAAGGGAAGTTCATCTTTTTACTCA

>115104

TGCAGCGTCTCGGGGGAGACCAGCGTCGGGTTCGGCCGCTGAGCCTCCTCCGTCAGGAGCTGGATGATCAGCGCCTCCACGTCGAAGAACAAAACG

>145122

TGCAGCCAACAGGAATGGACTACAGTCTTCCATCCCTTGGTTCAGGCTCTCAGAGCATTGAGGACGACATAGATCCTAAACAGGTAGTTGTCTCAA

>37114

TGCAGGTCAGGACTTTTCCATTTTGCTGAAAAGAAAGAAAGATAAAAAGGCCCAAAATGCAAAACACGATGTGCATCCCCTTGCTTGTGCTTGCGT

>205487

TGCAGCACAGGATTTGAGTTGGTGGCTGCTGAAGAGGCGTTTGCTGATTTGTGGTCATCATGTTGAAACAGTTCCGATGGGCTCACCACCTGTCG

>257022

TGCAGTTTTTTCAGAGTGAAGCCTTGTAATGCTGAGGACTGAACAGCGAGTGGCTGCCTCGCCCTACAGATACATCTCACTGTGTACTGTCAAAAT

>247928

TGCAGAAAATAAAAGCAAATCAAGGTCAGGACAGCGCAAACGCTCGAGTAAACACATTCTCACATGAAGATGCAAATTGTGTATGCGAGTTTGCAC

>136485

TGCAGGGGGCCGCGTCCCCGGCGACGGTATCCAGGGCCACCGCGGGGTCCGACTCAGCCAAGACAGAGGGCGGGGCGGAGGTAGAAGAAGATGGTC

>13088

TGCAGGGCGAGACGTGTCCACTGGAGAGTGGACGACCTGATCAGACTGAGTCAGCTCTTCACCTTCCACTAAGCCCAGGTTGGTTATGTTACTACC

>290159

TGCAGAGCTTTTCAATAGCTGAAGAAGAAAGGCCGCCTTAGGCGAGAACCGCTGCTCTGCACATCACACGCACTGATGGCGGGAAGCTGCACTGCA

>18147

TGCAGCTCTTATTCTGTACTCTACTCTCTGTCTAAGGGGCGTCACCCTTATACCTCTGATGTTCAGGAGTCGTGTGTCTAAATAGCAGCTGTCAGA

>211422

TGCAGTCTGCATTCAGATAAATCTGTAATATTTTGTTGTCCATTCTTACTATTTGGATGGGAGAATTGCTAAATTAGTCTTCAGTAATAGACCTTT

>250361

TGCAGTTGAGAATATCCACTGATAAGTGTGAGGAGCACAAATGCCAGGCTTTACACTCCGTTTCAACATCCCTCTGTTCCCTTTTGAAACATCGAA

>19572

TGCAGGAAGTCGAACTTTGCAGAGACACTTTCACACTTCGGCACCATTAGAGCTCCGTAATCCCGGTGGAAACTCGGCCCGGTTCCACAGGAACTG

>87953

TGCAGTGGTGGTGGTTGGAGAAGTTGTTGTGGTTGGAGAAGTTGTTGTGGTTGGAGCTGCTGTTGTGGTTGGAGTTGCTGTGGTTGTTGATGGAGC

>289245

TGCAGAAATCAACTGCCGGGACGATTTGATGGGCTCAGATCTGAGAGGAGTGCAGGAGGAAAGGGAGACGATGATGATTCGAGGTCAAAGATTGCA

>146093

TGCAGCGCGTCGCTTTTATTTCTTCTTCTTGAGCGGAAGCGTCTAAACATGACGCTGGGCCGCCGGCCAGCAGGGGGCGGTGGAGGCGCCGCTCCA

>335441

TGCAGCACTGTTAGCTGTAAACAAGCCAACAGGGCTGCCTTTATCTCTTCCTGTTCTGTCTTCACATAAGCAAAGCAGGACAAAATATACACATCG

>325039

TGCAGATGAGTTGTTGTTTTCTACTATAACAGGATGAAGAAACTGTCCACAATATTGCAATGTTTCTGGCTTGAAATCATACCTGACCTGCTCTCA

>313616

TGCAGTCAGAACTGCGGGACTAACTGGGGTGGCTAGCTGAGTCAGATGAACAGAAAACTCAAAAGAAGACAAACCGTTTGAGAAGGAGGAGGAACT

>87096

TGCAGCTCCCTCATCCTGAGCTCCAGCTCCTCCTGCTCCGCCTGCTGCCTGAGCCGCAGGTCTCGCCGCTTCTGCCGGGCTTCGTGGTGCGGCGCG

>50575

TGCAGTTTGTAGAATGGTGCTGCTGAGAATTTTTCCCACACAGACAGTAGGTTAGTAACGGTGACTTTATTCCTGGAATTTACCCCCCGTACAGAA

>131929

TGCAGAAAATGGATGGATGGATGGATGGATGGATGGATGGATGGATGGATGGATGGATGGATGGATGGATGGATGGATGGATGGATGGATGGAAAG

>27372

TGCAGGCAGCGAGAGCTAAAGTAGAGGAAGTGCAGTCCCAGTCATTGTGGGGCCTGACTAGCCTTTGCTGCGCATTGTTGTGTGTCCAAAAGTGGC

>104226

TGCAGATGAAATCTGCCTGAATTGAACATCTGATATCAAAGAGTCTAAAGTAGACGTGCATTCCTAATTGAGCGCTAATCACAAAGTTTACCGTCC

>117341

TGCAGTCGGCCAAACAGAAGCATCTTCGGGCTCAGCAGCAACAGCAGCCGCTGCACAGCTCCGGACAGGGGCTTACTATCTTTCACGATGTAAGTG

>78032

TGCAGGCAGAGAGACAACATTCAACACATGAAGTGTATTTCCTATTATAAGTACAAACTCTTCACACCTTTACCTTCATGTCAAACAAGTCTTCCT

>34661

TGCAGTTTGTCTCTGTGAGCTGAACTGGTTGAAGTGGAACCACAAACAGAAGCTGGTCTGTTTTTCTACCGCTGAGGTTCATCCAGAGTCCTGATG

>238255

TGCAGCTGTTCTGTTGTTTTCCTCATGTTGGCTAGAGGCAAACACAGAGATCTGCTGCTTTTATGGGGCCATTTTGGACAATGACAACCGTTTATT

>317403

TGCAGAGAAGGAGGGGCCTATGGAGGAGGAGGGGCTTGTAGAAGAGGAGGAGCTTATAGAAGAAGAGGAGGAGCCTGTGGAGGAGGCGGAACACAT

>37055

TGCAGGTCTGAGTGATTAGTTTTAGGTTTCTGTTCAGGGTTCAGTCTGGTGCTGCATGCAAAAACAGCAGTTTGCTGACTTTACTAATATCCAGTG

>334967

TGCAGGGAGGGAAACATCCAGCGCCGCACTCTGGCGGTTCTGGACTGGGAACCAGCTGGAACCATTCGGACTCGGAGGCGAGCGGCTTCCCGGCCC

>330776

TGCAGCTCTCTGTGAACAATCACAGTCTGCCCTCAGAACCATCAATCATCTGTTTTCTGTATTCGCTCCTGCACCTACTCAGGATCGCAGAGGGGG

>313846

TGCAGGCATGTTTCCTCGCGTGCACCTCATTAGGTCACCAACATGAGCTCACCTCTGTTCACTCTACCCTCGCAATCAGACCCTGCTGCTTACTTA

>304364

TGCAGCTGGGTGTGAATGGATTTTCACAGATTATCTGAGAGGCTCTCACCTCCTGCACACCAGATCACATTATTCTGAGGCTGTAGATTAGTTGCT

>156447

TGCAGACGGAGGAAGTTTTCCACCTTGCAAACCAGTTACAAGTTGTGATGCGTCAGTGGCGACTGAGCCGCCTCGTTTACGAGGACAGTGGGAGTC

>26130

TGCAGCGAAGAACTGCAAATAATCCTCCGTCACGTCGAGCTGCTTCCAGTTTCCCAGCAGTAAAGTGTCGCTGAGATCTTCACACAACAAACACTG

>326932

TGCAGGGATAATGCCACTCCTTCTGCTGCTGAGGAGGGACTTACAGGTCTGCTCACCAACCATGAAGGAGCTGTTGGTTGCTATGGTGACCACCGT

>323264

TGCAGGTGGAGGCGGGTCGGTCGGCTGATCCAAACCAAGGAGCTTAGAGATCATTTTTCTTGGTTTTTATTTTTCTCAAAACAAACCTCAGAAAAC

>6138

TGCAGAATAAGTGGATGGATGGATGGATGGATGGATGGATGGATGGATGGATGGATGGATGGATGGATGGATGGATGGATGGATGGATGGATGGAT

>135809

TGCAGTTATCCGCCTTGTAAATCAGCACACAGGAGCAAAGTGTGTATCACTACCAAAGCGTTTATGGGTTAGTGCAATCAAACGGCACGCTGAGCC

>328864

TGCAGACCGCAGGGCTACACAATCAAAAAGTAGTGTTCTAATGATGCCTGACAAGACTGTCAGATGATATCCTCCAGGGTGCGATGATGCTTTACA

>106409

TGCAGTGACAGACGAAGCTTTACGCTGACATCCGTCTTGACTGACGGCGCTGACCCCGGCCGTGACCCTGCGAATCAAGACACGCTCGCAAGCATC

>284411

TGCAGCCTCAGCACGAACCCAGAAGTTGGGCTTTGATTAGACCCCACAGGGGAACCACAGCAGACTGGTGGCTGTGGGCCAGTCGGCCCAGTTTAA

>312494

TGCAGTTTCTGAAGCAGGAGAAGTGTATTCTGAATTCTCTCCATCAACTGAAACTGCAAAAAAATGCACTGGGAGCTAATTTAGAGTGATAACAGT

>328147

TGCAGTTCCCTGCCGGCGAACAGAACCCGGAGCGCCTGGGCAGGAACTCCCTGCTGACTCGCCACGATGTCCTTCAGCTCGGCCACTCGGGCCGCC

>78894

TGCAGGGCGATTCCTCATTCAGCCCAAAACCAATGAATAAATAAAAATATGCATGAAGACAATGAGATCAGAGAACAATGAATATGAAAACAGCTC

>202807

TGCAGCATGAACACCAGAACGGCTCCAAATCAACCAAAGTCTGCATCGATTCCACCTGAGGGGACTTTGAATAGGACATCATGCTGGAAACTCATC

>235237

TGCAGTTCCCCCTTGGTGCCTCCCAACCTCCCAACTCCCAGAGGTCTGTTCGATTATTGGCAGACACCTGGATGTGAACATGGCCTTGAAAAGGCA

>167755

TGCAGGACCTGAGACGTGTCCTCTAGGAGGACGATGGACGGACTGATGTCCCCTGACACGCTTTGTCCTGGACGTCGCCATGGCAACAAGGCCTTT

>105578

TGCAGCTACAGCCTGACTCCAGCAAGGGGAGATGCTCTAATCCCTGCGGCCATGTTGGTCTGGACAAAATGGCCGCTCTGCTGGCTGTTCGGGCAG

>105390

TGCAGCTGAAACGCACCGACACGGTTTCCACGACGGGGGAAAGAAAGGAGTAAAAATCCCCCCAATAACCTGCTCCTTCGTTTTTCTAGAGTAAAA

>247094

TGCAGGAGGCGGTGAGGCAGCACCTTGCTCAGATCAGACGCCTCGAGAAGCAGATCTACTCCAACATCAGGGTGAGCTGCACCGCCTCGCTTTATT

>174344

TGCAGATTCCCCTATAATATTCAGCCTATTTCTGAGGGGGGTTTTTTAGCGTCGATGGAGCAGTTGAAAATCTCATCGGCTGCCATACCGAAAAAT

>234274

TGCAGTCTGACTGTTAGAACAGGGTCCAATCAGAACGTAGTCAAAGACAGCCAGAACGCACCAGAGGGGCCGGCACCGGCTCCGTCGGACCTGCAG

>65880

TGCAGGATAGTTTTCCATCCTCCTGCCACTTCAGTGGCCAAACAACACCAGTGACAAGAAAGTAACATTTGAGGACAGCATTTCTGTTTATTGACA

>100371

TGCAGAAACAAGAGGGCAGACGTTAGCAGCCGAGCAGCTGCAACTGTCCTCATCCTCTGGAGAGAAACAAATCAGAGCAAAACCTCTGATCAAATC

>137999

TGCAGGGTGTAAATATTGAGTGGTAGGTTGCATCGCAGAGACTCCCACTGCATCTGTGATTTCATTTGAACTTCAGCTGCACTGAAAACGTCTGAC

>21149

TGCAGCGTGTGAAAAGCTTTTCACCAAATGACTGTTTCACTGCGAGAAAGACTTTTAGTTCTCACTTTACTTTTGGTTTGATTCAACCAATTTATT

>213116

TGCAGGTGAAGCGAGTGATGAATGGAGTGTTTCACTCCCTCAGAGCAGAATTTGACCTCAGTGAGTCCTACAGTGGCCAGAGTGTGCTGGGGGTCA

>64627

TGCAGGTTCCTCGTCTTCACACGGGGCAGCTCAGTGATGCTCCATTCCGCCGCTTGTAAAACACACAGATACAAATAAATGAGGGAATCACGATTG

>286540

TGCAGCCACTTAGAGCCTGGCAGTCTGAGCTGGCAGTGCAAACGCAGACTTGGCAGCGTTTCCTTTGTCTTCCACGCTTCCTTCACAGTGGCAGCA

>73216

TGCAGCGACGATATCATTTATAACCCTAAGCACGCTCCACAGAGCAGCTTCACAGCCGTAAAAACCCGACTCGTCAAACGGCGCGCTCGTTTTTCA

>167884

TGCAGCAGCCAGACTTGATGTTTTTGTGCAGCGACTCTGTGACACGTGGGAGCTAGAGTTATGAGGTGCGAACGTTTCATTGTACAGGGCAGCCTG

>292048

TGCAGACGTCATGTGACGCGGCGTGTTTACGGCGGAAATCGCAGCTTTAGGAGCTTTAGGTCCAGAGATTCTGCGCCCTCGGGTTGAGCCTGAAGT

>177474

TGCAGGGTCAAAGGTCAGGCATCACATCAAGGAAACTCCTGAGATCTGTTGACTGGATCCATCCGCTGCTGAAAGTACCGTATTTTCAGCACTATT

>82309

TGCAGGTGGTGAACAGTTCCCTGACTGAGTTCAGCTGTGCCATCAAAGACCAGAACAAGGCGCAGAGGCTGACGATTCACCTGGAGAACTTTCTCA

>95749

TGCAGCTGCTGAGTTTCCTGAAGAGCCTCTGAGACGACCTTTGACCTCAGGCCTCCTGTCACAGAGTCATCATGAGAAACAGCAGGAACCATGAAA

>40998

TGCAGTAAAGGCCACGCCCCCACACACACCTAGCAGCACACAGCCTCTCCTGTTACCATGGAGACGCCTCAGCAGCCGCCATGTGACGTCACCGAC

>175201

TGCAGAACCAATCAGCTTCATGGCTTCAGTAGCCCCTCCCACCGAGATTGGGTGTGCTATCTGTGCTCCATGCTGATGGGCGGGGCCTGTGCTCTG

>248367

TGCAGAACTGCCTGGGAGGAAACGGCAAAACGTGAGTTCTTCATCATCGACCCTGCAAAAGTCACTCATCTGTTTAAGATCGGAAGAGCGGTTCAG

>127399

TGCAGTCGCTCAGGTTTTCACACCTGTTTGTCCTGCATTCATGGCTCCATTATCTCTGCCTTTTTGTTCGCCGTGGAGCCTCAGAAAAGTAGGACA

>45012

TGCAGTAAAAGCAGAGGAACAGGGAAGATTGGGATAAGATGCACCATTTTTCAACCTCAGATTAGTTGCCAACTGATAAATCTGCATATTCATAAC

>2954

TGCAGCGGCGACTCTGAAAGCACACCGTCCCAGGTGGCCAGTTAGCTTCTGCCGAAGAGCTGGAACGTCCCTGGGCCTTTTTTTATTCTCCAGAAT

>207744

TGCAGTCGCCCTGCTGACTCGGTTCCGGTTGACATCATTATTTGTGAGGCGACCCGGAGCTCGGCTTGTTCAGTCACTGCTGATTAGAAGCGGCTT

>232926

TGCAGCCTCAGAGATAAACAGGCCCAGCAGGAACAGGCTGTGTCTGTTGGTGTTGTTCGCTCTGAGATCCAGTTTGTTGAGTGGGCGTAACCATGG

>166722

TGCAGGAGGATCTGGAAGGCAGAGGAAACAACATTTCACTCCTGCTCAGCTGCTGCTAAACATTCAGGAAAGGTAAAAGCTGAACTGTCTAAACTG

>204980

TGCAGCCGCAGGGATCCGACGGGCAGCGGGTTCGGTGAAAATGCGCCAGGAAGTTCTGGTTCTGGGTGACCTGGCAGCGGTTTCTGCCCTCGGCTC

>39163

TGCAGGTTGTGTTGCAACCCCCTGCACTCACTTGGACCATAGCGTGCTTTACCTTGGTTGTGCAAAAAATGATGCATGAAGAGTGGCAGGGAGGGG

>183560

TGCAGCCCAACAACAACAGCTCCTTCAGCTCAGCATGACGATCCGCCCACCCATCGTTTTAGGTGGGGCAATCAATCAATCAATCAATCAATCAAT

>278887

TGCAGGCAGAAAGATGGGTGGCCTTGCTAATATCAGCTGGTTGTACTCTGATATCAGAGCAAAATGTCAAAAAGTAACATTTCTTTCCACAAATAC

>157643

TGCAGTTTGCTGACAGACTGCTCACAGAATACTTTTATCGTCCACATGTGGAAAATACCCCAGATCTGTAGATGCTGACCAAAACATGATGTTTCA

>19093

TGCAGGCGCACGTGTCAGCGAGGCAGTTCATGTAGAACCAGGTGACATCCACCTGAGGACACATAACAGGCTTCAGGGAATGAAAAATCATTTTTT

>76229

TGCAGAGATTGCAAGTTCTCCCTGATCATGGGTTCTCATCAGAACATCGTAACCAAACAGCAGTATTGCCCAGAACATCCTGAAGATCTGAACCTG

>82865

TGCAGCATGGAACGAGTACAACAAGGTGTGCAGAAAAACCTGAATGCAATCATTTCGGTGTACTTCTTCCTGAGTAGGTAAGAAAAAGTCTATTTG

>225355

TGCAGGTCCAGATTTGATGCGCTTTTACACCTTGACCAGTGGAGGGCAGTCTTTCTTTAGCTTGTCCTTGTCAAAACAATGCGCAGTGACACCAAA

>258807

TGCAGAGGAGGCTTGTTTATGGAGCAACATCGCTGTCAAATGGAGGCGCGACGTCAGAAGTCAATAAAACGTCTAAAAACAGCCGAGCGGTTTTTC

>160362

TGCAGCACCGGATGGAGGTGAGGAGCCTCCGGACCGGTTCTGGCCCGGTCCAGAACGTGTGAAGTGAAATGATTTGACGGGAGAATTTAGTTTATC

>140655

TGCAGAGCTAATAACAGAGTTAGCCTCTGGTCAATATTTCCATTTTGCATGTCCTTGAAACATAATGGACATGCATGTTTCCTTGAAACATGCATG

>14502

TGCAGACCATTGAGCTCCTGGAATCCCGTGGTCCGACCTGGAGCCAGGAAGGTTTTCCCGGGCTGGAAGTCGCCGCCGCCGCCCTGCTGGATGAAG

>31691

TGCAGCCTCCTTCTTTGGTCAAAGGGCGACGGTTTGTCCCGCTCCAACCTGTCAGTCAGGAGAACAGGAACATGAACTCCTTCATGTCTGACCCGG

>262785

TGCAGGGAGATAGAAGGTGGATAGATGAGTTTTCCCGTCAGTACTACTGTGTGTTGAGTGACTGATAGGAAAACATTTACATAAATATGACAAATT

>273244

TGCAGTAAAAAGCAACTTTCATTGGTATGTTTTGTGAGTAATCTCACTGGACTAACCTGAAGGTTGAAGTGGCAATCAGAGCTGAGCCAACGGCAA

>112869

TGCAGCGTTGCCTTGGCAGAACTGGCCAGGGAGTCGTCCAGCCGGGCCGACAGCACTGCAACACACCCACACAAGATGCTGAAGTTTACAGCTCAC

>188658

TGCAGCGCATCCTAATGAGAGAGATGGGAAGATCAGAGAGATCAGAGCGCAAAGGCGTGCAAATTGTTTCCATCTCATCAACAGCTATCCACTGTT

>109432

TGCAGACCCTGTTGGTACGTAAAACTATCTGTTTTGACTGAAGGAAAAGTTTTTCTGTTAGATACTACATCACTTTCTTTTTACTTTCAGACAAAC

>309137

TGCAGGTGAAGGTTTGCTGCTTGGCGTTTAGCCTCCTTGCTGCCGTGTGGCCAGATGTTTGTTTCTTCCCGACCAGAACATCTTAAGATCGGAAGA

>302291

TGCAGCTGATGAAGTGAAGAAGATCCTGACTGGAGTCGTTGGAGGAAACGTCACGTTCCCAGATCCTCTGCTGGAGTTTGGGTTTCTTTTAAGATC

>31405

TGCAGGGCTCACACACACACACACACACACACACACACACACACACACACACACACACACACACACACACACACACACACACACACACACACACAC

>47425

TGCAGAGCAAAGCCTCTTTCCGATTACGTCCCACTTTGAAGATGGGAGTGGAACTAAGCTGGTGTTCGATGTGTGGGAACGTTCAACGATCCAAAC

>107061

TGCAGGGGGCGTAGCAGCAGCATGCCGCCCCGGTCTGTCTGAGATGAGTTGGAGCTGCTGCCTCCATGTTGAGGCGGTTTATGACGGGATGTGCTG

>145148

TGCAGCCGATTTCTGTCGACTTCTAAACCAGCCGACCTGCATAACCATCCTCACAGCTGGAAACACATCCAGTGTCATGAAATGTTGAAACGGGCT

>271053

TGCAGCTGTGGTTTTCATTTGGAGATGCTGCCCTCTGGTGTCTGTGGACAGAACTGCATCTCATGAAGTGTTTCTGTTTTCTGTCTCAGAGTTTAA

>149192

TGCAGGAGATCTGAGGCAGGAAAAACAGCTGATGGATGGATGGATGGATGGATGGATGGATGGATGGATGGATGGATGGATGGATGGATGGATGGA

>231150

TGCAGCAGGATAAACAAACATCCACTTGTTTGCTCATTTTAGGTTTATCTTCTGCTAATTGCTTTCAGCATTCTGAATCCAAAGGTTTGTGTTCA

>64562

TGCAGTGCTGACATTGTGAAGCAGGGGTTTGCTCCAGGCTCCAGCGCCGTCCTTAGAATTAGGCTGGACTCCAAACTCGTAAACGGCATGGGGCTT

>54855

TGCAGGAGGTAAAAACCCTCTGATCTTTGATCGTGGCCTGAAACGTGAGCTAACCTTTCAGCTTCCCTGCTCCAGATCTTCGACGGACAGACGGTT

>181818

TGCAGCAATCACAGGAACCATTTACACGCTTGTTCGGGTCGGTTCTGACCAACAGAACCCCGGTCTGGTCAGAGGTCAGAGGTCAATCGAAGAATC

>88895

TGCAGAAGCCCGTACGGCATCCTGGGTTCAGGCACAGTCGTCATGTGGGAGCGAAGACTTACCCTCTTCTGATGGGAAGTCTTCTGCCCCCCTTGG

>27528

TGCAGCAGAGGAGATTGTCAGATTTATGTTTCCTCCCTCCACATTGATGTCCAGTCTAGCAGTTTGCTCCGTCAGTATTTTTCCTGATCCCAGGTG

>111265

TGCAGGGTCGACACGAAGCCCCAGAGGCAGCGAGACCACCAAGCAGGAAAACCAGGCAACCCGCTTCGGCAGAGACACGGCGACCTCAGACGCACT

>109036

TGCAGCAGGTTGATGCACACATGTCACATTGTTTCTATAGATTCACTGAAAACACCAATAAACCCTGAAAACCACCTCATTTCTCGTCTGTCACAG

>142944

TGCAGGAGAAGTTTCAGCAGCATATTTGCTCATTTCCTCCATCTGCCGCAAACTTTCATAAGCCGAGAGTTGCTCCACATGTGCGGTAGACAAGCA

>225948

TGCAGCTGAGGGTAAAAGCCAGTACATTCAACAGCATTTTCTTTGCAAAGACATCAACAGTGGCAGACATGCACATTAGTCTCTTACCCTCATGCC

>277273

TGCAGAGTCGCAATGCTGTGTTCTGGAGCTTTAGATGAAAATGGCGGTCTAGTTCAACCCAAGAAATTAGTTCGAAGAATACTGGGAGCTACAAAT

>60090

TGCAGCAATCGCTTATTGTGTGGAAGCATTGAAGGCGACTTGATGTTGTGGTGGCATAGCTGTAGACCTAATGATGTGATAATATGCCAAATATGC

>246366

TGCAGCCACGTTCAAGTAGCGAGCCTGCGGGCCAGACAGCGGCTTCCAGCAGAGTCAGACCTGTTGGGTGGAAGCAGGGCTGCGAGTGAAGCACGA

>229928

TGCAGGACTCTTGAGGACTTCAGCTCAAGAACAGGAACAGGCAGCCAGGAACTTTGGACCCCAAGTGTGCAGCTAGGGACTTGGACGGGGTCATTT

>45827

TGCAGAGCCTAAAATCAACAGTTAGTATAAGAAGTGAGAGTCGATTCTGATGGAGCAAAACCGAAAGATTCAAATCAACTGAAGCAGAGTTGATGG

>96156

TGCAGATTTTTGAATGAAACCAGGACTTAGTCTTTTGGTTGGGCCAATATAGTCACCTGCATAGTTTTGGATTCAGAGCTCCTGCGGTTTCTGGAG

>168155

TGCAGCTTTTGCCAGCAGCTATCAGTGATGACAGTTTTGGTCCCGTCCTGCCCGCCTGGCCTCCTCTGCACGCCACTGACTGCTGCCGTCGCCGTG

>130498

TGCAGATGTTGTTGCAAGGTTCTGCTGAAGCTCATGCTTACACTGATCAGTGTTTGGTATGACCTTTGCTGACAGTGAGTCACCAACGAACTTCTT

>275803

TGCAGGAGACGCGCAGGCACCGACGCGGTTAGCAGGTCATCAAGCTGGGGACGTGAGACGTAAACAGCGTCCAGCTGGTTAGTGAAACTCGGGAAA

>88724

TGCAGCACTGCCAGCCAATCATAAGTGAGCATTGTGTGGGAAAACTGCATTCTGAGAATGTTGCATTTTTTCCTATATTGACTCTTTGAAGAGTTC

>46160

TGCAGAGACGCCGACTCACTTCCGGTCCCTGCTGGAGCCGCCTCACTCACAGAGAGCGCCGCTGAGCCCCAGTAGAGACAGGGAGCCGTTACGCTG

>78316

TGCAGCCGGCCTGCTCTGTGTGTCCCTGATTCTTTTATTTGCTCTTCGTTGGGTTTCATCAACTTTCTTCCTGCTTCTGTTCTGGTTCTGAAGAAG

>217942

TGCAGCGTCTCTCTGCGTCTCTAAACAGCCGATCCATTTTCATCCAGCTTGTTTCAGACGAGGCCCGCAGCATGCCTGAACAGAACCGCTGCTTCC

>53177

TGCAGTAACTAACAGAAACGGTTCAGCATCGTTCAGCTCTGCTGGGTAAACAGCTCCCAGGCTGGACTCTGGTCCTCCAGCCCTCCAGGAGCTTCC

>179187

TGCAGCAGGAAGTTTGTTCTGGATGAATCTAGTGAAGCAGCACATGGAGTCAGATGATGTTTGCTTCAACGCTTCAGATCTGAGCGCACACACACA

>57866

TGCAGGCTTCAGCCACCTGCTGCCCTGGTACTACATCATCTACTTCTTCATCCTCCTGGTGCACAGAGCCTCCCGCGACATGACCACGTGCAGACG

>305428

TGCAGGACTTCAGCTGCCTCAAGGTGGGACAGCGTCACGGCATCTCACATGTCTGTCATGATGCATGTTTCGATTTAAGATCGGAAGAGCGGTTCA

>59021

TGCAGCTCCGACATGCCACCTCTGATTCGTACCTGCCAAGCACAATTTTCAGCTGCTTGGCTCTGAGCTCAGGCTGGAGGAGGAGAGTCTCTCTGG

>37648

TGCAGTGAACTTTCCGTTGGCCGGGAAGGAGCAGGTTATGTCTGAGGAGTAGCAGTAGTACTCTCCTCCCATGTCAAACAAACTGCAAAGGAAAGC

>128274

TGCAGCTGGAATATCCCAGGGGAGTCGCCTGAGGAAAATGGAAACCGGTTTCACAAGGAGTCACAGCAGGAAGTCAGAGGAGATGAGAGAAACTCT

>101225

TGCAGTCGTCTAGCGCAGCTCCATTCGTCTCTGTTGTTGCCCGCCTGGCTGCCTGCTCTCCAGCTCCCTGTTCTGCTCCACTCAGAAATCTCTCAC

>208848

TGCAGTCTGGGATCAAATGGTTAGGAGCTGGAAAAACCCTTTTTCTTTTAGGTCAGTGAACACCTCACAGCTCTAACAGCAACACTCCAGTGAGAT

>278697

TGCAGCAGAACCAGGAAACTCTGGAAGAGACTCAGATGTGGAGAACAGAATCAGGAGGTTTGTCTGCTCTTCTCTTGACTCTGAAGTCTTTCTTTG

>59671

TGCAGCCACCATGCCCGGCAGCCAGACCCGGAGTCGGTCCAGGGACCGGAATAATGTCCTGAACCGGCCCGAGTTCATGTCCCTGAACCAGCCGGT

>195102

TGCAGCTTGGAGCAACTGTTCGTGTCAAAAATCGATGTACAGAGTAGAAAAACATAAGATAGTAATTACATGGAAAGCATAGGGCGAACGGACTAA

>10658

TGCAGAGTTATGAACGTACCCTGGAGTGAGCGACCACATGAAACGTCTACAGACAAACATTTTCTATCAGGATACCGTATTATAGTTGAGGACAAA

>40334

TGCAGAGCAACCATCAGACGTGAGACAGAAACCGTTTGGTGTTTGGGTTTATCAGAACCGGGCAGCTGAAAATCCTGATCGAGCCTAAAAATAACA

>111293

TGCAGAAAAGCAGGTTTTGTGTGGCGGTCTTGAAGGCAACTCTGTTCCTTTCTCAGTCTGCCGGGTTATTCGCAGGTCGGCAGTTTCCTCCTGGTC

>68172

TGCAGCGCCCACACGGCATGACAGCTGCTGTCTGTCCTCTGCTGGTTATGACAGTTATGGATGATAAGTCTGGTCCTCCACTGAATAAAAGTCATC

>36392

TGCAGACTTTCTCCCATGGGAGAAGTTTTGCAGCGTTCACTTCTTTTTACTCATCCTGTCCAGAACCAACCCAACATGCCATCATTTCTCATGCAT

>123013

TGCAGATCTACTGCTGCCGTAAAACTCAGCAGCGTCACTTCCTGTCACCCACCAACCAGCTGATGGAGTCAAACACCTCAAGCTAACCTAGCATAG

>83997

TGCAGGTCGTCTGCTCTGAGCTCAGGTGTTTCCTTGGAGCTCCTCTTCTTCCTGCTGAGGAGGATTGATGAAGAGCAGAGGACTGAGCCTGTTTAG

>24823

TGCAGCTGCCTTATCTTCAGGTTTGGCAGCATCAGCTGGCTCTGCCCAGTCAGAGTTTAGCGCAATGCCCACTTTCCCTCCCTGTGTCTTTCTGTA

>275139

TGCAGATGGGAAGTGAAGGGAAAGTATACCTCACCTGATCATGCAGCTCTAAAAAGAAAAAAGACAACTTCAAAGTAATTTATTTGAATCTTTAGA

>190338

TGCAGATATGTATCTTGTCGCTTCTACTTATAGTCATGTTGTAATGCAATTTTGCAATTACTGGGAACCAGAGCCATAAAGAGGCCTTTCTTTTCC

>206407

TGCAGCTCAGTCGATGTGTCTGTTGTTACTGATCTGTAACGATCGTTGGGAACGTACGACTTCTCACTTTCGCTTTTTGCTCCTTTCCACAGCAGG

>314849

TGCAGGTTTACAGAAAAACACTGGATCTCTGCTCTGACACACAGGAACCTGGAATGACTTGAGCCATGCACTTACTGCACAGTTTGAAGTTCATAG

>213055

TGCAGGGACGCCATGTAGGGTCTGGAGTGGGGTTTGGAAACTTTGCCTCCAACTATGCTGCTCCCAGAAGCGCCTGACATATGGAGAAATTCTTAA

>200365

TGCAGCAGCAGCAGCAGTTCCTCCAGCTCCAGATCCTAACCCAGCAGCAGCAGAAGTACAACTGCCAGGCCGTTGTACCTGTCACACTGAAGTATG

>25530

TGCAGACGTTCTGGTTCAGGTCCAGATGAAACCGACTCATCCAGAACCTTCATGATCCTCCAGGTTTACCACACGGCTTAAGATCGGAAGAGCGGT

>168930

TGCAGGGGGCGCTGATCAGCACCGCTGTGTGTTTGTTTCATTTGTGTGTGTGTGTGTGTGTGTGTGTGTGTGTGTGTGTGTGTGTGTGTGTGTGTG

>161142

TGCAGCTCTAATTTGTACTCAGTAACAAAAAGGTTGTTACTGTCCACACCTTCACCCCGTGTTTGCTTAGGAGGAAGAACGGCTGAAACAACGCAG

>54339

TGCAGCCGCAAAATGATCCCAGAGATACAAGCGCCAGAAACACAATTTCAGCCATCTGATGTGGACCCCCACATCAGTACACATCAGGTAGCAAAC

>188635

TGCAGAAAGCTCAGTTATGATACACCATGTAGCACATTTTATAAATCGCCACTGACAGGGAAAAACCTCTTCTCTCCATATTGATCATGCTCCACA

>87355

TGCAGAAGCAGAGTGTTCGAGTATTCCCAGCATGCAAGCCAAATGCAAAAACCTCTCTGATGCCGCAAACGAGCTGTCTGTGAAAGGCTGCGTGGG

>38643

TGCAGAAAGTTAGCTATTTCCTCTAGCCGGCGGTTCGTTTCTGCTTGGCAGGTGCTAAGATCACGAAGGACCTTATCATGGGACTGAATCGCTGTT

>198888

TGCAGCAGACGCACTGAAGGAGGAGAACCCCCTCCACGGCTCTGTGCAGCACCGATTACCACCCGGTACCGGTCCGGTACCGCTAACCGCTTTTTT

>273793

TGCAGCGAGGTCCTTCTCGAAACGAGCAGGGACAGTGTACACGACAGACCCAAATTTTAGAAATGAGACCATTTCCTTCTCCGGAAATGAAGAGGG

>149063

TGCAGAAATAAAAAATAATCCAGCTGGAGCAAAACAAGCCCGCAGCGCAGGGGATGATGGGAGCTGTAGTTCCCAGGCCGACAGCAGCTTGTTTAC

>259961

TGCAGTGGCTCAGGTATCAGAAAGAGAAGCTGCTGTGGGACGAGAGCTGGATCATCGACTTCTCCACAATAAAACAAGGTACCGGTGGCGCGAGGC

>22976

TGCAGATCAAATCCTGACCTCGATGTCCCAGATTTTCTTCAACACCAAAGAAAGTCAAGAAATGCCTGGATTTATTCAGTGCTTCAATTACAAACA

>206235

TGCAGCTCCAACCACAACAACTGAAGCTACAACAACAACCACAGCAACTCCAACCACAACAGGATCTCCAACCACAACAACAGAAGCTCCAACGAC

>94828

TGCAGCATCTAGCTGCACCCATCCGCTCCTGCCCTTCATCCTCTTGGCACTTGTTTGTTTAGATGGAAAGGAGGAGCAGCAGCTGCCTGTCAGTAG

>266518

TGCAGTTTGCCCAATAACATGCCTCGGATTTGCTGTCAGCAGGTAGTGAAAAGCTCCTCCAGTTGAGCAGATCTGGAAGTTTACTTTAAGATCGGA

>221237

TGCAGGTTACTTTCTAGAGCCTTCTGTAGCTTCTGTAGGGATATTTTCTTGCTTCGACGCAGAACTTGCCAAACTAACACAACTTTCAGGAACCAG

>275904

TGCAGGTGGGACGCTGGCTGAATACATCCAGAAGCGCTGCAACTCCCTCCTAGATGAGGACACCATCCTCCACTTCTTTGTGCAGATCTTGCTCGC

>263188

TGCAGCATGGTGTGTTACCCAGGAAACTGCACTAGTATGTTGAGTCTGATACGAGAGGATTTTTATGATACTAAATCTGGCATGTAATGTTGCTTT

>265590

TGCAGCGCCTGTTTCGCCTCCCGGAGCCTCAGAGCCGTGAACAATCGTTCACTGACTGAGACAACAAAGAATCTTCTGTTGTCTTTTACTTTGATT

>20377

TGCAGTGGGAGGATCGATGAAAACTTCCCTGGTGTTCTGGACTTTGAGGATGGATTATCCTCACCGCAGAGAACTTTATTCTGCATCTTGCCACTG

>158330

TGCAGCCGAACATCCCGAGCAGAGCCGCGGTTAGAAACATCTACAGATATTAGGGGGAAAATTCACATGCAAGCATTCAGGCATGCAGCTAAATGA

>123451

TGCAGACAAAGGAACAAGCATAGAGGAGGATCACCACTGAATGCCAGCAGAGGAAGAGGGGCTTACGGGTCCAAACTGACAAGATAACATGCAGCA

>127503

TGCAGAAATTACCACAAACGTTTTTTCACTTTTGTAATTGATGCTTATCGACTTTGTGGCAGATTGGATAAGTTCTTCGACCGTGTTGCTGAGGAC

>297835

TGCAGCTGTTTCCTGCTGGCTGGGTCAGAACCGGTCTGCTTGGTTGCACTGTGTCACTGTTTGGTTCTGAGTCTAGACCCAATTCTGGTTCTGCAG

>246267

TGCAGCAGATTGCGGCTCAGTCGTGAATGGTTTCCTGTTTTTACTATTTTAGTATAAATGTGTGATCTCCTCCTCTGGCTTCACTCTGCTGCTGTC

>325864

TGCAGCTCCTCAGACTCACCTGGATGTTGGTGGTGATCTCCTTCACCTCCTTCTCCATCAGCCGGACGTCGTCCAGCTCGCTCTGGTCGGCTGCGT

>245804

TGCAGGTAAAACACCAAGGCTGCCATACCAGCCCTCAGGGTCACTCCTCCTCCACCGTGCCTGGTACCAGGAGCTTAGAACCATGTTAAGATCGGA

>185896

TGCAGCCTGATAGATCTGATGACACTCAGCCACTGGACTCACCTGGTCCAGGTGTTCCTGTCAGACTGAGATCATTTAGTGACTCAGTTAAGATCG

>55739

TGCAGGGCCGTGCAAGTGGACTCCAAACCCTGGGGGGGACCGTACAGACTCAGAAACGTTGATGACAGAGAGAGGTGGCCCACCAAGTTCTTCAAT

>86393

TGCAGCGCCGCGTTTCGGACCTTCCATTCGATAAAGTTGTGAGTGGGAGGATGAACTGGATCAGAACCAATCTGAGAAGGCGTGACTTCAGAAACC

>271361

TGCAGCAAACAAATCCAATTTATACCACCTTTTGTGGGCTATATAAATGTGATTCCACAAGGGATTCAGACCTAATGGCATCATAAATTGATGGCA

>145135

TGCAGTCTTCACGCCGTCTGTGCCGCCGTCCTCCATGTTCTGTCGCAGCTCGGCCCGCCGCCGCCCCTCCGTCAGCACGTCACTCAGGTCATCAGA

>121930

TGCAGTTCAAACCTGAATCTATAAAGTCAAGGTTCATATGGGGGTTTCATCCAGTCACAGCAGCAGTGAGGAGATCTAACACACTGACACTCAAGA

>102438

TGCAGTCGATCAGGTGGATTTCTTGTGCGGTTGGAACGTCTCAGAGGTTGGTCCTCAGTGTGCAGAGGTACTGAATCCATAGGTGAGTGAGGTTCT

>179772

TGCAGTTCTATAATATCAGTTTCATACTTACTCCTTAGCTCCTCATATGCAAAGTGGACCTGATCAAGTTCATTTAGTATGTATTTCTCCCTCTTT

>57282

TGCAGAGACCCTTTTGGAGCAAGAGCATTTTCACGAGGCAGTCGGCTGACAACTAGCCATCTATTTAGGGATAATAGGGCCTACTTACGCCAGAAG

>4667

TGCAGCCTGAACATGACGCCTTCTGTATTTTTTTTCGTTGAAAACCAGTTTTCCGCTCAGTGCTTCTCGGGATCTCTGTGCTGTTAGCTCAGAGTG

>136469

TGCAGCTGCTGGCAGCTGAAGCAGCTTCACATGAACAACAAGCTGGTCTGTGATGATCATCACAGCCTGACAGGATCGTTTCTCTGAGACGCTCAG

>250523

TGCAGGGACGATGCTGAGAGGAGACGGGGTGAAGGACGAGAGGTCAGAGACGCAACGGGAAGGCTACTAACACCTGGAGACACCTGGAGACACCTG

>148708

TGCAGAGGGACGAGGTTCTGCTCCCAGAACCGGCCGGTTCTGATGAAGCAAAGCCCGCTTTCCTTCTGTTTCTGACCAGCCGACTCCCAGTAGACA

>207296

TGCAGAAAGAGCAGCAGCAGCATCATCGGGTCTGATGCTGAGTTTATTTCAGCTTCGTAAAGGAGGTCAGACGGTTCTGCTGGAGGATCCGGCTCC

>4504

TGCAGAGGTCACCGAGGTCAGCTGAGCTAACCAGAAAAATGTTCTGACCCAAAGCTTCAAATGTTCCCACCAGAGGTCGCGCTAGACCTTTTTGTT

>135127

TGCAGCCCAAATACGGCGACTCCATCCGCCAAATATAAGCAGGTAAACACCGGGCGAGCTGTCAAACAGACATGATGAGTTCAGCACTCATGTCTA

>223185

TGCAGACAGGCCCCCCAAACCCCACAAACCCCACAAGATCAGCCAAGGAGCAGTCAGCCTGAGACCCAACGTCGTCCTGCCAGTGGGCCTGCTGGT

>7643

TGCAGTACCTGAGAAACTCTGCTGATGTCATCAACCAGCCAGGTAAGCTTTCTATATCAACACCCTGGTGTCTTGGCCATGGCTTTGTTGATGGTA

>129531

TGCAGAGAGAAACCCAGAAGCCTTGAACTCATTTATCCTTCATGCTGAACTCTGACCTCTGACCGACAGCCAGCTGGGCGGAGATTCTCCGCCTCC

>332085

TGCAGATAAAATAATGGTCTGACTATTACAGCTGTGATAACGTTACCGTATATTTATTGTTCATTCTGTTGAGATAAACAGTGAGTATATATAAAA

>320810

TGCAGCGGCTTCATGACGTCGGTGATGAACTGCGGGTGCGTGTAAGCGTCTCTGATTTTCTCGTGCAGCAGAGCTCTGACGCTGCCCTGCAGAA

>325519

TGCAGACACGAGTGGAGCAGTTTGTGCACAAGGTGAGAGGTCAGCAGTGGGGTGAGGGGAGTTAGACAGTAAAACAATAACAAACCAACATACAAA

>133748

TGCAGGAAGGTCGAAGGTCCGCGGGCCCCCAGAGACGAGAGGCGACGAGCTCAGCACAACGAAGGTTCGTCACTTTTACTCCACTGAACAATTCCT

>205705

TGCAGCTCGGAAGTAACTGATGCTCAAAGAGTAAATTGACTGGAGAAACTTTCAGCTGAGAACAGAGACGGAGTTCATCTAAAGGTGAAACTGAGG

>328360

TGCAGGCATCATGCTGAGTTTTGTAGCAACGGTATCCGAGTCATCAGACATTACAATCATTGTCTTCCTGCGTGGCAAAATTTATTATCTATGTGA

>30816

TGCAGACAGCATCCTCGAAATGTCCAAGTCGCCGTACGGGAAACACATAGTGAAGAAGCTGCTGATGTATGGGTGAGTTGGATTGGACCCTTCTGT

>206490

TGCAGTTGAATTTTTCTGCTGATGTCTCCGTCTCGCTCAGACGACCCCCTGGTGACGAACACGTTAGCGTCACGCGGCGTTTCAAAGGGTCAGCAG

>256737

TGCAGTCCGTTAGAACTTTTTTTGTCCAGCGTCGACGACGACGGTAGACGGTAGAGGAGGCGGACGAGCAACTTGAAGTTCTACTTTAAGATCGGA

>268749

TGCAGGCTGCATTCTGGAGAATAAAAAAAGGCCCAGGGACGTTCCAGCTCTTCGGCAGAAGCTAACTGGCCACCTGGGACGGTGTGCTTTCAGAGT

>172532

TGCAGGGAGCAGATGAAGACGGCCGGGTATGTGGGGAGGAGTGGGGAAAAAAGGGGGGAGAAACGGGAGATCAAGGATCCGATGGGACACCAGGAT

>152731

TGCAGCGGTTCAACACAGACAGACTCTCCTGATTGGACACGCCCAGCTCCTGAGCAGCCAATCACAGAACAGTCCATGAGAGAGGAGGACAGCAAC

>109601

TGCAGGTCATCAGTCCCAGATGCTGTAAAATCAGCAGCTAATCGCAGCAGCGCCGCCGACCCCCACATGGCCGCCACAAATCTCCTTCATATGTAC

>145726

TGCAGGGACCAGGATGTGTTCGCGGTCTGCCGCTGCTTACGCGACGGAGCCACGGTGATGATGGAGGCGATGGGCCGCAGCGTGGAGATCACGGCG

>161354

TGCAGACAGGTGAGGGAAGAACATCGGGCTAAAAATAGAACTGAGAGGATCTCGGTTTTCACTTGGAAACCCAGCCGAAATAAAGCCAAACTTCAG

>126941

TGCAGTTTGCAGAAAACCATGACCTGTGTAAATCCTCTGAACGTACTCAGTCGTTATATTCCGCTGAGCGCAAGACATTACGGTGAGATGTATGGG

>275307

TGCAGGCAATTTTTAGCTAATCTTTGCAGTAAACATCCACATTTTTTTCCTAAATGCTCTTATTGCATTTAGTGCTTTTCTATTCGGATCCCTTGA

>197816

TGCAGGAACCTGATGATCCAGAGATCAGAACATCCAGTCAGTGGACCGAGTCGGTCACTTTACTTTGTGATCCTTCACAGGAACCACCTACAGGGC

>120224

TGCAGGGGGCCGCGGCCGGGCTTAGAGAAAGGTAACGCACAGGTCAACGGTGAATCATGAGGCGATAAACACATGAGCAGTAACACACGGAGTCAA

>114377

TGCAGGGAGAGATTAGATACTTGGACCCAAACATTAGATCATTTACAATAAACACTTGACTGGAGGAGGAAAAGTTTAGAGGTGAAGTAAATGAGC

>318559

TGCAGTGATTTATTTCCAGACCGGCTCATAAATATTCATACACCCCTTATCTGGGTTCTGATGAAGTTCTGGAGCCATCCCGGTAGAACGCTGATC

>17599

TGCAGGCCTGATAAACGTGATGGTATTTACTGTTGTAAACCAGCAGGGTGAGTTTGTGCAGGTCCAGAGCGCTGTAGGACGAGACGCTGAGCAGGG

>23423

TGCAGTGCAACAGCCTTTTCAGGCCTGTCTCTGCCTGACACTGGCAGCCAGAACCACGACTATGAAATGTAATGTGAAATGTATCGCTAAGCCATA

>94717

TGCAGTCGATTACGTCTGTGAGAGAGAAAAACAGCAGCGGCAACAGAAACTACATGATGGCCTTCAGAGTTTGGTCTGCTGGCTCTAAAGCAGAGG

>259100

TGCAGACACACTACAAACACGACCGGTCCGACTTTCACCTGCTCAGAGACGCTTCCTGAGCCGTCAGCCTGGTTGTTGGTGCTTTCACAGTTAGAG

>260867

TGCAGGTGAGGCCTCACCACTTGTCACTTCAATTTCCCATATAAGTAAGAGCCTTATGGAGACCTGACCACTGTTTATAGGGGCAGTATTATGTAT

>115762

TGCAGCAGCTCACCTCTGCTGGAGGAAACACGGAGGAACCTGAAGACGACGAGCACGGCGACATTCAGAAACACCGTGAATATGAAGAGCGCTCGG

>199202

TGCAGCTTGATGGCTACAAGATGTGTGAAATTTCTAACTTGCTCAGACATTTATCCAAAGTATAAGGACATATTTGAGCACCTGATGTGTTTTGTG

>226383

TGCAGGCTGTTGAGGACGGTGAAGATGTATGCGACCGCCATCATGCCCTCGCTGAACAAGAAGGCTCCAAACACCCACATCAGTCCCAGTATGCAC

>79200

TGCAGTTAGCGCAGTGCGGGGAGAAGAGCGTGAAATAGTCCGACTCGCAATATGGCCGCCCGTCCTTTTCGAAGAAGTTGCGGCTGCCCAGCTCCG

>102380

TGCAGCCGAAAGGCTCAGGAGAAAACTCAGCGTGAAAGTCTTCATGTTTCCTTGTTGCACTCAACTTGTGTCTCCCACCTTGAGGGAGCCGTTATA

>232772

TGCAGACCCTGAAGAACAACTCGAGGAAAATAGAAATAGATAAAATAGGAAATAGATTCTCCCACACACACACGCACACGCACACACACACGCACA

>315956

TGCAGCTGCTCCTGTCCAGTGATGGAGTGCTGGTTCGTTCAGGAGAAACCAGGCAGAGGAGGAGGACTGACAGGAGCCACAACACAGGAGAAATCT

>313013

TGCAGTTCCCTTGGTGCTGTAAACCCCGCAAAGACATTCCTGGAAACATCACACAAGTGAAAGTTATGTACAACGTTTTCAGAGTAGAAGAGTTCA

>263479

TGCAGACATTTCTTTTGGCTCTGGAAGTCATAAAAAGTGAAAGATCCGACTTCACGACTAACTGCGAAGTAAAACCAGAGTCACCAACAACACTCA

>42575

TGCAGTTTCCAAACATCCTGAAACACCACAAAGCTGGAATCATTTCACAACATTGACAAAGAATGAAGCAAACAATAAGGACACAAAGAAGGAAGC

>140552

TGCAGCCTCCAGGTAAGCGAAAGGGAATGAAGCTTTCTTCACGGCGACACGGAGCGAACCCAGCGGCTAAGATGAGTCCCATCTCCGTCCCTTCAG

>165595

TGCAGCTTTTCTCCTTCTCTCCGTCTTTCTTGCCCCTTCCTCATCCCTCTTTGCCTTTTCCTCCCTGTCATCCCGTGTTTTCCTCTCCCTCCTCTC

>318185

TGCAGCTCTAGGACGCGGCACTCAGGGGCCCGAGACCTCAAGCTTGTTTGTAAAAGTCACTTTTAGGAACTTTTCATAAGACAGGGGCCAAATTTA

>141120

TGCAGACATTCGCAGACTGTTGGGCCTCCCGGGTCAGCACCCTGAAGACGGGGAGAGACGGAGCAGGATTTACTGCTAATGTGGCCGCCGTGACAC

>22414

TGCAGACACACAGGGGTTCTGTGAGTCCATCAGAACCAGAACCTGGTTCTGGTCCTAAAGCGCCCCCTGCTGCTGCTTCTGGGATCTGCTAGAAAA

>86727

TGCAGACAAGCACATGTGTTGGCTGTTTGTTGAATATTATTTTTGGCCAGGCATAGCCTGTTGTCATGCATGAAATGTGTACCGAGTGACATCTAT

>119967

TGCAGAACATCACCGACTATCTGATCGAGGAGGTCAGCGCCGAGGAGGAGGAGCTTATCGGCGCCGCGGGGGCCGGCGCCAACGACGACGACCCCA

>100232

TGCAGAGGCTGCTATTGACAGCTCATGGATGAATAGTGGGAATATGCTGGAAGTTCACACTTTATGCTTCTCAGACAGCACAGAGAAACTATTTAG

>193110

TGCAGTGCCCTTGACGGTAAAGACTGGGGGGTCCTCTGTAAATCATTCAGCTAACCCTGAAAGGTGTGAAAAGATGGTGGTGAAACGGGTGGAGCT

>219098

TGCAGCTGGAGTTCGGGGACACCGTCTGGCTCCGTCTCCATGGCGACCCCCGCTACGCGCTCTACAGCAACACCGGGCACTACACCACCTTTAAGA

>303231

TGCAGTGAACCGTTGGTTCTTTCCAGCTGCTCCTCCAGCTGAGCCTTTCAGGGATCACAGAACCCGACCGAACCGCCCCACTTTCACCCGGTTCCT

>210637

TGCAGATTGTTTGTCGCAGATCCAAACAGCAGTTAGTGACGGAGCCTCAGCGGCTTCGGGCTGGACGCTTCAAACCAGCACGAAAAATTCAGCAGC

>58670

TGCAGTAGTGATGACGGCGCGGCTGCCCTGAAGATGCCTGACTATAAAGGGAAACATTAGACACTGAAAACACTGAACGGTTGAAACATGGTGATT

>107502

TGCAGCTCGGCAGAGCTCCAGCCCGCAGTCCCCGCACCGCGGTGGACCAGGTGTCCGAGGCCGACATCCAGCGGCTGCTGCACGGCGTCATGGAGC

>253814

TGCAGGACTTTGTCCTCTTTGATATTCCATGTCTCTGGGGCTTGCATAAAGCACATTGAGAGAAGAGTCAAGGTCACTCCCACTCTGTCGCTGGGA

>337784

TGCAGAAACATTGCACTCAGACTGAGGATCATGCAATGCAACTACAATGCACCACAGCCACCTCCTTCCACCCACACTGAGCGCACGCACACACAC

>79396

TGCAGAGAGCCAAGCAAGTTGGAGAGGATAATCTGAACAGATCACTGAGGGAGAAAGACGGCATCATCCACCAGCTACAGCTCTCTTTAGAGGGCA

>124831

TGCAGAGAGAACCCACAAACTCCATGCAGAAAGATCCTAATGAAATGACTGAAAGTGGAGAGTTCACACAGCTGCACCTGTGAGGAGGTTTTTGCA

>26250

TGCAGATAGTAGAACTGATCAAACCGACCCAGCTGGAGAGGTTTCAGGGAACGTCACAGCTTCCAAGCTGTTTTTGGGAGGGATTGTTCCCAAAAA

>20121

TGCAGACAATGGGGGAAATGTAGACAAATGTCCGGCTCCCGTTGGAAGCTTTTGTCCGGCCGCAGTTTTTGCATTTATTTCAGGGCCCGAAATAAT

>151589

TGCAGATCCGGTCCATGCTGAGCAGAATACCGACCTCCGAACCCGTCTGGCACCGGTCCTGAGTGGGTCGGGAACCTGGTTGTAAGCGAACTGCAG

>209774

TGCAGTGAAGATTCTTGTCAAGAAGGGAATACCACGTTGGTTTATTTCATGCGCGTCGAGGAAGCAAAACAACTACTGACGCGGGAGGATGACGTA

>4919

TGCAGAAAGCCTGCGCCAATTCCTAATGACCAAGTCCAAAAACGTGTCGACGTGGCATGCACCGCAGAACCGCTGGACGACGGACATTAGCTGAAT

>211025

TGCAGTTAGAAAAAGCCCAGAAAAATCTTTTCTTCAAAGTGTTCAGAGGTTGGTGCAGTTTTTGGTCAAGCATCACCTGGCCAGCATGTTCCCCGA

>136497

TGCAGACAGACATTGCGGCACTGTGTGAGCCTCTGCTCAGTCTGTCAAATACGAACGCCATTTTTTTCCCCCCCACATCCATCTGGAGTGCCAGCA

>96334

TGCAGGAAAAGCCTCCAACACTTCAGTCCCAATGCTGCGTCGCTGCCTGAGCCATGAAATGAAAAACGCTGAGGAAATAGTAAGTTTGTCAGAAAT

>5276

TGCAGGACAGAAATACGATTACACGACTGCTAACGCGGAGTGTCGCTCTGCACGGGAGTGTGCTAATGAATGCAGCACCACCTGTTCAGTGAGCGG

>207897

TGCAGGGTGTGTGTATCCGTCTAGGTGGGCGTGGCTCAGCTCGGCGCTGTGGGTCATGTGACCGGGGCTGGGGTCCACAGGCGGCGTCTCATCGTC

>154368

TGCAGGAACTTCAAGAGATAGGGAGACTGAAAGCTGTTGCTGCTGCCAGCTTTCAAAATGCCCTGAAGAAGTTTTATTTGTTAGTAGGAATTATAG

>124067

TGCAGGTCCTTCTGCTTCTCCGCTGCTCCTCCTCCATCTGCTGAACGGATCGCCTTCAGGGTTGAGATGTTCTGATAATCAGGATCAATCTGATCA

>170389

TGCAGAGAAACGTCTTCAGATGCGTCTGCATGGCTGCTTCCCTCTGAGCTCCAGTCACACCGGAAATGTTCGGAGAATATTTCTGCTTACGCCCAG

>214887

TGCAGAGCAGCATTACACAGATATGAAGCACAAGGCAAAAGAAAAAACCAAACATGAAAGGGACCAAACGACGTGCATCCCACACAACAAAAGCAA

>227154

TGCAGTTTGGTAGTATTGATCTTTATGGCAACCATTGAAAGTTGCATGAAAGTTGACATTTTTCCAACTTTCATGCACAGGTCCACCACTAGATAG

>144608

TGCAGCTGCTGTCTGTATTTGGTCCAAACAGTCCGAGTCACAAAAAGCCGTGAAGGCCGGAAACAACAAACAAAACACATAAAGACATGTCGGATG

>26296

TGCAGAGGTCCAAGGGTTTCATAATCTTTCCCGCTCACTTCCACATGAATGCCCCGGTCAGCTCCCATGGCAAGAGCAGTACGAATGGTTTCCTGT

>288308

TGCAGGCCCTCCTCGGTAATTCACACACTACGCCCGCACAAACACGCACACAGACGTGCAGCCTCGCACGTGCACCGCTCGGCCGGGGCGTTAAGA

>166810

TGCAGCTGGACTCACAGGGAGGTGACAGACGGGCCGAGGAGGAGGAAGCAGAAGGTTTAGTGGTCCAAAGTTGGCTCCGTTTTCCGGACAATTCAG

>214993

TGCAGGCGGCATAAAGAGAAGAGGCAAATGAGAAGTGAATTAGTGTTTTTACAACCGGACACGCAGCGACGCCTCGGAGGGTTTTATCTTTGTTGT

>274203

TGCAGCACTCCTCCGCTCCACACCAGTAAACAGCAGAGAGTCTGAGCAAACCTCTGAAAATAACAGAAAAACACTAAAACTCAACATCCAGACACT

>324861

TGCAGCGATGTCCCGCCATATGCAGTCCACGCAGTGAAGGGATAACCTCATGATATAACAATCATCCTGGGTGTGTGTGTGTGTGTGTGTGTGGAG

>18606

TGCAGTGGCTGTCACCATCAGCCTCCCTCTGCTCCTTTCAGGCCAATGTTTGCCAACTGGCTGGGCTTCCTGTCCCTCACAGCCTCTAAGCGGATA

>238523

TGCAGACAGGTGTTTCTGTCTCCTGCACTGAAACTTTTTTGAACTTAGCACACGAAGCAGATCACATGACACAAAAATGGGCATAAAGCATAAAAA

>321931

TGCAGCAGTTTGTGAATGAACACGTCTGGTGCAGTCAGGTGTTCAGTGGGAACAGCAGCGGCTATAGAATCGGTTCGTATAGAAGCTGCTGCAGA

>95077

TGCAGAGGCCAACCAGTGCAATGCGGAGTTCCGGCCGCTGTTTGTCACCGTCATGAAAACAAGCCAGAGGAAAACAAGCTAGAGGCTAATTTTTTA

>50687

TGCAGCAGCTGTGAAAGTCATGATCACACTAACTACACCTCTAATCACAGCTGCTCATGGGAAAAGGTCTTGTTGTTTGTGCTTTTCCTTTTTTTT

>192427

TGCAGCTACTTTACGGGCCGCCGCTCCAAGTCACCCTGAGGGGAAACTTTAGGACGATCAGGAGGAGTGAAGGGAAGCAGGAACTGGTGACGGATT

>188847

TGCAGATTTGTTTGCCTGTTCTGTTGGTTTTTCACATATTTTGTCATTGATTTGTTTTGCTCTCGTTTTTCTTTCTTCTGCATTGATTTTCTCACA

>279464

TGCAGACCGACCGACCCGAGAGAGAGGGACAAAGGGAGAGAGAGAGAGAGGGTTTGTGTGGTAAAGAAAGGACAGAAGCTACAGTACATTAAGATC

>181066

TGCAGCAAACGGTGACCTGAAAAAGAAAGAGCACCTGAAATGAACCATTAGACCGTTCTTCATCACAGATTGAATCACTGCTAACGTGGTGGAGAC

>51803

TGCAGGGGGCGCCACCGTTACCACTGAACACCCGGCCCGCTGGGCCTCCAGAACCAGAACCGGTCCACTTACAGCCAGGATCTTATCCCCGATCTG

>16055

TGCAGAGAAAACAACCAGAAAGGGTTCGACGGACAGAACGTTCCCGTCTGAAGAGGATTTTTTTTCTTCTTTCCCCCCACAGATGCAGCATCACCT

>172275

TGCAGGCACATAGTGATCATCTCGGCGCAAGCTAACATCTTTCCAAACGCTTCGAGCTTCTTTATCACTTCTTTATCGGGGTGAAGGTTTCAGTAA

>290967

TGCAGTGATACGGTTTGTAGTCGCCGTTGCAGATGTTGACGTCAGGAATGGGAACGCCGTCCACACTGACATGAACCACATATTGAGCCTCCGGCA

>293255

TGCAGCAGATGGAAGAACTTGTGTAAGAGCACTTTAGCTTTGCTCCAGAGTTTTGCGTCAATCCTCAGCAGGTGCCACTGTTCTGATCAAATGAAG

>264446

TGCAGGTCAAACTGGGGATCACACAGAAGTCACAACATTTAGATTTTGCTCTTGCAGCCTAAAAGCTTTCAGGTCAATATTCTGCTTATGCTTCCT

>200446

TGCAGGAACGTCGGCTCAGAAACAGGTGGGGGTCACTCTGACGCTCCAGCTGAGATGAATTTCCTCTCTGGTTGGTTCTGGAGAATCATCACTGCT

>238858

TGCAGAGCAAGGAAGAAATGACTGATCTGGGTAAACCACAATTCACCCTAACACGACAAGTTGTCACTGAAAAGAACAGAGAACTATTTTTATAC

>315248

TGCAGGCGCTGTTGTTAGATGTGCAATCCCCACGGTTTTAGTTGTGACACATAACTAAAACGACTGTCTGTCAGTGTAAATACTTTGGCTTTTCGA

>263636

TGCAGTCAGGCAAAATGCATTCCTTCTCATTTAGATACAGTAGAAAGGGGAACACGCCTCCTTGGAAAAGGCTTGGAACCGGAACAGTGGGCTCTT

>303121

TGCAGAATGCAAAAAAAAAGTCTAACTGTGTTTCCCTGTCAGGGCAGTAGGAGAAAACAAACATGTGAAGTCCTTCAGAGTCAACACTTGGTTTGT

>39542

TGCAGAGGGAATAACACGCCAGGATCAAACATCCTGTCAGCACACACAGAGGCTGAGCTGTAGATTTACAAACGATGTGCCGTTTACTCACAGGGA

>315630

TGCAGGAGGGAGACAGGGCAAAGGTCACACAGAGGACCAGTCAGCATGGAGAGCGCCACCTGCTGCTCAACCCGTCCTGACCAGAACCAGAACCAG

>212952

TGCAGCGCCCGAGCACCGGCCACCGGGTCTTTGTTGCCGTAAGGGTCCTTCTTGTCGTACGCCTCCGCAGGCAGGTGGCGCCAGAAGACGTTAAGA

>83352

TGCAGTAACCCACCAGACCGAACCAGACCAGCCACTCGGGTTCTGCTGGGTTCTGCTAAAGGCTGCGGGTCAGTTGGTCCCAGTTTCAAACCAGAC

>35001

TGCAGCTCTCTGCTTTTATACTCCGTTATTTTTGACCCGCCGTGCTTACGCAAAACAAAATTCCGGTCCAGCCTGCGAGCCAAGTGGAAATGGACT

>155656

TGCAGCTGCTGCTGCTGCTGCCGCATCTCCTGGATCTGCTGCCTGATCTGCTGCTGCTGCTGCTGCATCTGCTGCTGCTGCTGCTGCATCTGCTCG

>88309

TGCAGCGACGCAGGGACCGACACACATCGGAGGGGCCGAGAGTTTGGGGATTCCCAAAGGGCGGTTCGCAGTCAGGATGAGGTCACCGCGGTGCTG

>314078

TGCAGTAGCATGACGTCTTTCTTTTCTTTTTTTTTTGGGCTGTAATCAACTTTTCTGCCCACGCAGAGAGAGTGCAGGGCGAGGGAGCTGAAAAGA

>296840

TGCAGACACATTCAAGAACTTGCTATAGATTCAGTGCAGTCTGAGGCAGCTGAGACAGTTTGCATGAAGGCTGAGGTTTACAAGTAGTTATGATGA

>165516

TGCAGTGACATGCAAATAGTTGGTCCCAGCCAACTTTACCCACTTTGAACTCTCACATTCACCTCAAACTGCTTCTGGTGTCTGTTGATGGAGCTT

>89006

TGCAGCTTCAGTGGCGCTCAACTCTCCGCGCCATCGCAGAGCTTCGCTCCACGGATCCGAACCCGCCGCTGGCATCCGGCCTGGACGCCCAAGACC

>144469

TGCAGAAGAAGGAATGTTGACACGCTGAGACATTACCTAACCTCCACAAACATACACAACCTGAGACATTAGCTAAGCATTGACAGGCCCCCAAAG

>4121

TGCAGGTCACTCTCAGTTCTCGGTCGCGTTGGCCTTCCCCTCTCAGGCTTTGCCGTTCATTTCCAAAGCAGCAAAATGCCATTTATCTGTCAGTTG

>288769

TGCAGCTCATACCTGGAATTGTTTGCAAAACTGTACTTTCCAAATACAATTGCAATCAAATTGAGCTTCTTTATACCTATTTTGTACATAACGAAC

>256047

TGCAGCATCATGACCAGCGTCAACTCTCCAGCCTGCCTGCTCGCCCGGAACCACTTCTACAGAAGTACGACAAAACCCCGACACAAACACGGCTTC

>111392

TGCAGCGACACCGATGGTGAGATGTGCTACTGCACCGAAACTCTATGCATAACTGAGGGAGAGAAGATTTGGGGCCTCGTTCCGTTAGTACAATCA

>74430

TGCAGCGCCGCTCTCCTGAGGGTTCTCCTGGCGAACAGAAACGGTTCATTACGATTCAAAGGCCGACTCAGGAGATGATCCTGATAGCACGGAAAC

>207685

TGCAGGGTAACTGGGAGATCTTTGAGGAGTTCCCTGAGGAACTCACTGATTCCCTGGCCAAAATGCTTCAGCGGCTCTGTGATGAAGGTTGTGATG

>302822

TGCAGTTCTAGATGCTCTGACCGATCTGAATGGCCATTTCAACTTGGCCCTTGTCAAACTCACTCACTTACACTTTTTTCTGCTTCTCACTGATAA

>27525

TGCAGGTTACGGACAAGGCGTCTACCCTGCTGCTGGATACGGAAACCTATATGGAGGTTTGACGCTAAATACGACTCCTTACCTGTTCAGAACCAT

>135807

TGCAGGAAGTAGAGAACCATCAGGATGTAGGCGTAAGACGAGAGGCTTCCTCTGGAGGCGTCGCCGATGTCGCACCGCTGAGGAGACACAGACCGT

>232204

TGCAGAGAGGAGACGGAGAGAAGACGCCGCAAAACGGTTCCTTACGCCGAGAAGCCGTTGACCATGTGGGCGTATCGGACCAGAATCAGGTCCAGC

>90730

TGCAGGTCCGGGCCTTCTACACCCAGGTGCTGAACGAGGACGAGCGCCAGCGGCTGTGCCAGAACGTTGCCGGGGCGCTGAAGGGAGCCCAGCTCT

>217696

TGCAGAGATGTTAGCTGCCGTTTCTCGACCCTGTGCTTCCTGTGTCGCCATATGAAGCATAGAGTCCATCACTGGTTCAGAGAAAGAAATCAATGG

>130712

TGCAGAAAAGTGAAGCAGAGCTATCGACTTGCTAAATGAATTCCACTCCAGCTCTCTTTCCAGGCCTCTGGTTGTAGTCCAGGTCTAGAGGGAGAC

>188455

TGCAGTGCAATAATTGTTACTGACCTTTAGCATCTTCAAAATGCTAAAGGTCACAGGAAGCACACACGACAATGACAGAAACACCAAAACAGGCAT

>321413

TGCAGCCTCATTTATCTGGCTGATATCTGCATGAATTACTTGTATTTTCATGACTGTCCACTAAGTGCAGCTGTTGGTGAAACCTTTGCTCTGATG

>38127

TGCAGGCGTTTTTACGGCTGATGCGTCGCACATTCCGGTCCAAAGATGCACAAACGCCCACGGAGAAGTTTTAGCTTGAAATCTTGACAAGTTCGT

>52050

TGCAGACTCGTCCTGCTTTTATAGGGTCTCCTGGACGGGGTGGGCTCTCCTTATGGGCTGCGTTTACTTTTCATTCACTGGCTGCGTAAATGAAGA

>111252

TGCAGGTTGGGTAGGTACTCCTTCACCCACCGAGTCCAGAAAATGTCGGCTAAATACTGGACTTGGCGCCAACGTCGACGGTACAGATCCTGTTTT

>88550

TGCAGCAAGTGATGGCAAAGCCGTGCGCGACATTTCAATGCCTTTGCTATATTTTTTCAGCCCTTCTGTTGTCGAGTTACTGTCTATTTGCTGTGT

>83400

TGCAGCTTTTATGCTCTGTCTGCCTTGCATCAGCAATCTGTCTGTTCAGCTGCCAGTTTCATATCAAAGTAGCTATAGACCTGGAGCAGAAACATG

>28215

TGCAGAACTCAGAGACATCCCGACACCGAGTCGCCGGAACCAGAACCGCAGGTGAGTCACACTTCAAGCTGCGGTCCGACCCGGTTCGGACTGTGA

>118061

TGCAGCTCTAAAACAAAAGTAAGGGGAATCGTGTGGAGAGACTTCACCACCTCGTCACTTCCCTTCGCCATCATCCATCATTATGGCTAATGCTGA

>266824

TGCAGACGGATTCGACGTAAAACTCCAGGATCACGACGGAGAAAATGATCATCGCAAACGGAACGACGAGCGACGACCAGGAGTCGACTTTACTCT

>161251

TGCAGCTGCTGCTGGTGATACTGTTGCTGTATTTGCTGCATTTGCTGTTGCTGTATTTGGTGCTGTTGCATTTGGTGTTGCTGCATATGCTGTTGC

>132421

TGCAGAAAGTCTTTTGGTGATGACATTGGTGTCAAAGTCAGAGAATCATTTCTTGGAAACAAGGAGAGCTGAAATGATGTGGGAGGCACGAGGAAT

>321079

TGCAGCATGAAGCAACAAAACGCTTCAGCTTTGAGTGTCTGGATGTTGAGTTTTAGTGTTTTTCTGTTATTTTCAGAGGTTTGCTCAGACTCTCTG

>45964

TGCAGAGTCCGATACTCTGCATGATCAGCTCTGCCTTTATGAAATGGCCACGGATTCAGATTGTACGCCTCCGCCCAAGTATTAGCATGGCGGCCC

>37185

TGCAGCTGCCTTATCTTCATCTTTGGCACCCTCAGCTGGCTCTGCCCAGTCAGAGTTTAGTGCAATGCCCACTTTCCCTCCTTGTGTCTTTCTGTA

>196610

TGCAGGAAGAAAAAGAAACTGCATAACCAGTCTGGTGCTGGAGGTAAACAGGAAACCAGAGAGGCAGGCGGCGCCCCCTGCTGGGGGGGTGAAGGA

>64351

TGCAGCTCCAACAACAACCACAGCAGCTCCAACCACAACAGCAGCTCCAACCACAACAACTTCTCCAACCACAACAACTTCTCCAACCACAACAAC

>17568

TGCAGCTTGGTGGCTTTTTAGCCTCCATCCATTTTGCCACTGCGAAATGGAGCCTTCTCCATCCATCCATCCATCCATCCATCCATCCATCCATCC

>2979

TGCAGACATAATATCTGCTGTTAGCCATGAAGTCAGAGGGACAGAAGGACAACCTGTCTCACTCAGCTGTAACTACGAGACATCATTTGACTATGC

>163409

TGCAGAGAACAGACACCCACACTGGAATTTGGAGGACAAAGCAGGGGGAGAAAAAAGAGATAGTTGATGATCTGTGCAAGCAGTGTTGTGGTGGCC

>289179

TGCAGCAGGTAGGTCAGCTTGGAGTTCCTGTATGGGACGTGGCTCTCCTGAAACGGTTAGTTTTTGTTTTAGAAGCAGAATGAAAACTGGCTCATG

>33311

TGCAGTGTTTACGTCACACGTCGACTCGCAGGATAATCGTTTGATTGGGTTTGATCGCATCTGCTGGAAGCAAACCCAATCTAAGACCACCCGTCA

>9627

TGCAGCAAACCGATACTGAACGTGAGACATAGAACCACACTAATAAAACCAGATCGATAACGGTTAGCTACAGCTGTTTCCCTCACCTTTGTTTCT

>196481

TGCAGCAGACACTCACCATCAGGCATAGATGATGGAGTTTATGTTGTACAGATCTTGATGTCAAGTAGAAGTTTTCTGCTCTTTTTATTCCTGTTT

>169724

TGCAGGCTACATTTTCTGTTCACTTCCGAAGAGGGCACCAAAATAATAGCATTGTGAGCTGCCCCAAGGTCAAGTTCCAATGCAAAAAAGCTTTAT

>198503

TGCAGGTTGGGTAGGTACTCCTTCACCCACCAAATGTCTGCTAAATACTGGACTTGGTGCCGACTTTGACGATACAAATCCTGTTTTACAAAGTGA

>183364

TGCAGAGCTGAGATCAACGTCAACAAGCTGCAACTTGAACTAACCAGGGCGGACTGGCACTAATGCGGAAATACAGGATTAGCACCCACAGTTAGC

>204133

TGCAGATTGTGAAAACTCTCCTAATTTCTTTATTGATAAAGTTGTAAAAACTGGGACTCTAATCTCATGAGCTGATTGTGATTCATTGGTTTCTGT

>232190

TGCAGGTCCTGGTCAAACTGGCCGGTCAATGGTTTGTACTGCACTGTATTTAGTCAGAGTGACGCCTCCTGTCCTCATAACCTATCTCTGCGGCCT

>166861

TGCAGGCTCCAACACCGGTTTGACGAGACTGTCATTCAAGCTGGGCTGCTGATGTCATGACAGGGCAGGCTACACCCCACTGTCCTGTGCTACAAG

>262287

TGCAGAAATAAACTCAATTGAATGCCATGAGCAATTTACAATCTTCAACTCCACATAAAACATATACCGCAACCCAGAAACATGCAAGACTTTCAC

>87147

TGCAGCAGGGGGTCAGAGGTCACCGAGGCGCCACATTCATTATGGATTATTACACAGAAATGGATCCTTTGCTGGACAAACACAATAAAGTTTTAT

>292205

TGCAGAAGTTCTTCAAAGGGATTCCTGGGATGTTCGTCTTTATTCCTCGTTGAAGTTATTGCTGTGGGAACCTTTTGTGATGTCACTTTGAACTTT

>209786

TGCAGACACCAGCTAGTGGTGCTGAATGTAGACGGCCCCACGGTGACCTCTGACCTCTAAACAGCTGCCTCTGCTATTTCCTCTGAGTGTAGATAC

>260084

TGCAGAGTTCCCACTGCAAACTGGAAATTCTCCGGTCAGACATTAGCTTCTATAACTTCATTCAATGATAACTTAGTTTTATTTTTCTTATTTACT

>36432

TGCAGGCGGTGAGGAAGAAGTACTCCAGCAGCAAACTGATGAAGATCAGCCTCCTCCCTCAGCTGAGCTCCTCCATCATCCAGAAGTTGGCGGCTC

>80084

TGCAGGCCTGAACAAAACAAGGTGGAAATCTGTGACTAAACATGTGGGGAAATCACCTTCATTACACACTCTGTACACACAGACAATCCCTCACAA

>240000

TGCAGCCCTTAGATCTTTTGCAACCTGTAATATATTTTTTTCCAGTACTTCCACAGCATGATGGCGCCACCATCATGGTGTTATGTTTTTTTTTTT

>128789

TGCAGGTGAGAGATGAGTACGTCCAGTTCCTTTCCTGCGTCTGCACGACGCAAAAGAAGAGGTACTCAGACTTCAGCAGCACCAATCCGTCCAGCA

>318316

TGCAGCGCAGCGGACTCGCCTAGCAGCACTAGCCGGAAGAGAAGAAAGAAGAGAAGAAAGCGAAGGCGGTGTGAACGGCAGCAGAAGCGCGGCTGT

>87651

TGCAGTCTACCCAATGTGAAACATAATAGTGGTAGCAATACGATGGGCCAGGAAGTACGTGGCCCCAATATGAAGTCTTTTACTACGTCTAACCAC

>275632

TGCAGATTATCGTCCCTCACTGCTGTTATTCTGAGAAAGTGGCAGCTGGCAACACGGCCAACATGTGGTGTTGTCCCTGTGTTATGACTAAACGCT

>186409

TGCAGATCAGCTGGGTTCGTCTGCGGTGTTCACAGAACAGAATCCAAGAACTGCTTTGATTTCATTGCATGTGTTCTTAGAGATGTAGGAAGGCAT

>175548

TGCAGACTATAACTTGTTCGTTCTGACATCCAGCAGAGCTGAGGATGATGTATTTTCTCTGACTGAACTAGGAGGAAAACGCTGAAGCTGCAAGGA

>165464

TGCAGCTCGCTGCTCCTGACCTGAGGAGGGTTTTATGAATGTGACGCAACAAAACCACCAACATGTCCAGGAGTTTTCAGTTATTTACAGATTCTG

>70471

TGCAGTCAGGGGGCGGAGCTACCTGCTGGTGAGGGATCATGGGAATCACGGCGTCCATTCTGGGCGGCGCCATGGGAACGGGACGCTTCGACCTGC

>29089

TGCAGGCTGCGCCGCTTTGCTTTGTGCGCATCGGTATCTGCGCTCCAATCTCAGCGGAGCCGCACTTCTGTCGACGTTTTTCTGCTGCGGTGAAAA

>166698

TGCAGTGAATGTTTGGGTTGTGAGGTAGATTCTGTTCAGATTCACTCTGAATGTAACTTCATTTGAAAATGAGCTGAAACATTCAGATGAGTTTTA

>337022

TGCAGTCAGTTTCGGCGTCGTTTGGAGACTGTACTAATCATTCAAGTGCAGCGCTAAATTAGTGCCTTGGAGGTGTGAGAACAGGAATGTTTGCAC

>197949

TGCAGACAGAGGTGGAGGAGAACGCGGCCCTCGGGGAGGCGATGAAGCTGCTGGTGGGCGAGCGCTGTCTGCCAGTGGAGCTGGAGAGGTACAACC

>197660

TGCAGTGTCTTCAGGAGGACCGTCCTCTTCCTCAGTCTGAGATTGCTCCACATCTTCATCCTCGGCTTGAGAACTCGTTGCAGCCTCCTCTTCCTC

>303162

TGCAGTTTCTCCTCCAGATTGATCCCAGACCATAATTATTCATACCACGCTCTGGTGGATTTACAATGAAATTCAAGAAATCAAGAAGGAAAAGAA

>295044

TGCAGCGTTAGACGTTCAAGGTGCTAACTCCTTCCTGTTGGTCTGTCTGGGGTCATCTATGTCCAGCATGTCTTCCAGTATGAAGTAGGGTTTAAG

>187959

TGCAGAGCAGGTGACACTAATGGAGCTCCTACCTTTAGAACAATGCATCCCCTCTGAGGCTCACAATGCTGCTTGAAGAGGAGTTTCAGTCTCTCT

>122794

TGCAGACAGGAAGCAGCTCTGCCAGCTCCCCGTGTCTCAGTGTTTCTGTGTCCAGATGTTCTGCCTCAGCGCGGTTCTGGACCCGCGGTGTTCTGT

>175122

TGCAGAAAACGTTTCTCGTTCCAAGCTTGTTTTTGTTCAGCGGTTCCAGTGAGGAAGTGCGTTCTGCTGAAACTGTAGTTTTGCTTTTGACGGGAG

>30366

TGCAGGGGACCGTAATGAGCCACTCATCATCACCATGGCAATAAGAAGCTGGAAAGGCTTTCTGTCAAACGTGGCTCGACAGCATCACCCACTGGG

>147906

TGCAGGAGGGGGCGGAGGTGCTGAGGAGGAAGAGGGAGGAAGAGGAGCAGCACCTCAGGAAGATAGAGGAGGAGCTGAGGAGCCGACAGGAGGAGC

>153280

TGCAGAGACACTCCACCCGGCTGAAGCTTTTACCTGTCGGACTCCCGCTGTGGTCTGTGTCACTCTGAGCTGCGGCGTGATCGCTTCATAAACCGG

>86184

TGCAGTAAGCTATATTGTAAGCAACAGGGTTTTTTGTTGATGTTCAACTAAAACACTGGTGGATGGCTAGGGGATATTTTCAGTAGTCCAACATGT

>23998

TGCAGAGCGAGCTGCACGTTTATGACCGAGTGTGTTCCAGCACCAAGGCTAACAAAGGTCAGTCTGTCCTTTTTTTTTTACCCACATTTTAGTAAT

>275698

TGCAGCAGCACTCCACCCCACCCTCATAAATTGGCATCTCCCTGCCAGCCTGCACTCCAGCGGCCGGCCTCACACATGCTTTATGACAAGAGGATC

>126147

TGCAGACGTCTGCTCATTACGAGAGACCTTCAGACTCCCCCCAGTCTGGAGGAAAAGGGGGATTCTACCATAAATGTTTCATTACATCCCTAAAAG

>257876

TGCAGGCTTTCACATCCGAATAAGCGGCCAGGACGTCGGACGAGGCACGTTCAGTCAGCGACACGCCATGCTGGTGTGTCAGGATACCAACGACAC

>274176

TGCAGGCCATTTTCTAACACACACACACAATCAAACACACCATCGATCTCTTCTGCCAGGCAACAGTTGCACTGGGACACCTATTTACCTCATGCC

>204585

TGCAGCCGTGGATGTGGAGAGGGGCCTGAAGCTTCGCAGCACATCAAAGTTGTCACAACAATGCTGTGGGCAGATATGAAATAAGCCAGAACTGTC

>245326

TGCAGAAGGAAGAGCAACGCGCTCTGGCTGTTCTCAATGGAAAGCTGGAATCCCAGAGGGAGCAAATCCAGAAACGCTTTGACCAGGAAATAAATG

>114230

TGCAGAGAATACTCCAGAGTTCAGCAAAGACAGATTTGATTCTGATCTCAAAATCGAGGCAAAAGAGAAATCAGTCAACTTGACGATCCAGGATCT

>71078

TGCAGAAATCTTGCATCAGCACATCGGCCAGCTGAAACTCTCAAAAGCTCGCTTTTTCCGGCAACCTCTCCTCACAAGCGTCTTCTTCCCTCCCTT

>17356

TGCAGCCGTCATGACCTCAGCATGGAAACAAGCATGTTGTTCATCCTGGCCAGCGTGGATCTCCACTTTCTCCTGGGTTTTGGGAACGAGGCAGCA

>96176

TGCAGTTGACGTGTGGAAGGGTTACACCTCCAGTTTCACACCAGCAAGCGACGAGGCCATGCGGCCTGTGTGGCTGAAAACAGGAAGTTCATACAG

>4435

TGCAGTGGTTGACAAGCATGCACCTGTTGAAAGCTATGAACATCACAAAAGATCTGGGGATCGATCCTTTCCACAGGAGGTCAATACAGGTGGAGG

>158346

TGCAGCTTCAATGCCTCCTTCATTAGGCAGGAGGGGAAACTGCCTTGTCAACATAATCAGAGACACCATCTCCTCTTGAGGAATCTATAATCTGAT

>259813

TGCAGGGAGAAATGTGAAGCAGGAAACGTTCTTGTTCAAACACGAGGCCTGGACGCTCAGTCGGGTCGATACAGAGTTAGATATTCTACAGCATTT

>151242

TGCAGGTGTGGCTGTGTCTGATAGCGCTGGGCGTGTCCTGGCTGGATCCGAGCTCCAGGAGGGGAAACGACACGACCATCCTGATACAGGAGCAGC

>155849

TGCAGCGGGCAAACAAGCTGGAGCTGCGAAAGCCGCCAGAAGACGTGGGTGTGGTGCGCAGGACGGAGATCTACTTCGCTCAGGCTCGCTGGTGTC

>287893

TGCAGGTCTAGACCCTAAAGGTCCCTGCTGACGCATGATGAGTGCGCTCTGATGGATGAGATATCTGGCCGCAGGGACGCGATACAGGCCGGTCTG

>68857

TGCAGCTGAACATTTGTTATTCACAGGGTGTTAGACAAAGCAGATGTTATGCAAATTAGACATTTGAAATGAGTGAAGCAAAACACCAAGGCTGAA

>261299

TGCAGCGGAACTTCACCTGCTCAGTGAGAAATGCGCGCGGCTCCATGGCCCGCTGGGTCCAGCTGGAGGAAGAGGGTGAGGAAGACTCTGGGTTTA

>105612

TGCAGTTCTCTCTGGATCACCTGGTCATCCTGAGCCCCTCTGTTACCATGGCAACACACCTGAGGATGAGGGGGGCGGAGCCAGGAAGTGACAACC

>138216

TGCAGCTCCCCGTGCCCAGAGCTCCTGGCCAGCATGTAGGCCAGCCTGGAGTCGTCGCTGTCCCGGTCCGAGGCCCACAGGAAGTCCGCCGTCAGC

>165778

TGCAGCTCACATTCTTTCAGTCAGGAAAGAAAGAAATAATAGAATAATAGAGGGGGTGGTTTGGGAGTGTGTTGGCCCTATGGGGGTGTGGGGGGG

>162244

TGCAGTCGCACTCACTTCCTGTCTACATGCTGGGCAGACTACTGTCTGCAACACTAACGACCTCTTCCAAATTACCGCTCAATGCAATATTGACTT

>260803

TGCAGTGATGCTATCAGCTGTGATTTATGGAAACAAGGTGAGTAACCGTGACAACCAGCATCTCCTTTCTCTTGTACGATCACATTGTTTGATACT

>229770

TGCAGCTCTGCACATGTGCAGAATGGAGGGAACCACAGATTGGAATTTGACTAGGCCGTTCTAAAACACAAAGCAATAATTCCAAATAAATGTAAA

>176248

TGCAGGACAGGGTCAAAGGTCAGAATATAGGAGCAAACCGAACCAGATCCAGGTTCATTTGTGTTGCGTTCAGGAACCGTAGGAACCCCAAGGATG

>127346

TGCAGACAGGTGATGAACTCCTTCACCTCCTTCCCTTCATAGCAAATCTGGTAGACAGCAACAAACAAAGGAAACCTGTTGGACACAAACATTTTA

>259485

TGCAGAGAAATCATTTCAGCAACAAACCTGAGAAAAAGGTGAGAAAGTTTGTGAGACTTCAACAGTTGGAGGACGACGATCCGGCAGGCGAACGGG

>207180

TGCAGGCGACGGGGCTAAAAGCGTCCATCACAGGTGGACCGGTCCGCCTCCAGCTGCCTCCGTCCTCGGAGCCGGACATCGACTAGGACAATCCTG

>163629

TGCAGACTTTGATTAGCATCCTGTAGCCTGCTTTCAGGATGAACACAAACCTCATGGATTTTTTTGCCACTTTGTTTGATATACAACACAAAGTGG

>150303

TGCAGTAAAAACTGACCAATTCAAGGCTATATAGTGAAAACGGACTAATCAAACATCGGTGGACAATATCCCAGCAGTAGGAGAAGAGGCCCTCAG

>168263

TGCAGGCTTGACGATGGGTTGCGTCAGGAGTAAAGAAGCCAAGGGGCCGGCGCTGAAGTACCAACCTGATAACTCTAATGTGGTGCCAGTCGGTAC

>328362

TGCAGACACGGAGGACTCCCAACAACAAGAGATTTTGAATTGTTTTCGCACAGCCTCTGCGTGCTCGCCGCTTTATTCCTCGAAGCTACGGAGAGC

>292561

TGCAGAGAAAACAAACAGCCGGTGAGAACCCAACAGAACCCTGGAGGCATGTCAGGTTCTGCCAACATGGTGATGGACTGAACTTATACAGCACTT

>218478

TGCAGCTGTCAGGATCCCCAGAAGCAGCACAGCAGAGATGATGATCGCACAAAGTGTCCCTGGTGACATCTGTGTCTGCTGATTTGGTTCTGGGAC

>78638

TGCAGCGGTTCACAGTAAAAACAGGAGCTGCTCGATGGCACGCCGTCAGATCCACGCAGGAAGTTTCTGCGCTTCGGCTCGTTCTTATGATTCATG

>133072

TGCAGACCAGGCTGCGACCCGGTAAACGGCAACTGCTCTGCTCCAGGCAAATGCGAGTAAGAAATATTTTTTATTTTTGAGCTTCTGATGGTAGAT

>149240

TGCAGGACGCGGCGGTGTTGTCGCTCTCGCAGCTCCTGGCGGACTCCCTGCTGGAGCTGGACCTGACGTCCTGCGTCAACGTGACGGACCTGTCGG

>56638

TGCAGCATGGGATCTTCCTCCCCCTGCAATTGACAGATCTCATTATAAGATAAACGTGACAAAGATAGCTCATAATGCTTCAGAGAAATGTAACAC

>260458

TGCAGGGAGAAAATAGTGCACTTACCTTTGCTGCACAGAAAAAGGAAACTTGTGAGTTTTCTTTTTCTGCTCCCAGAAAAGCAGAAAGCCAAAACA

>89587

TGCAGTAAATAACCTGAACACCGCGGCGATCTGGCCAGCGGCGCTGCTGCTGACAACATGAGCGTCCGCTCCGAAGAGCCCAGGAAAAATAAATCT

>303062

TGCAGAGTGCCCTAATAAAATAATAATGCACCGACGGCACTCTGAGAGGTGGACAGAAACAGCTGCCTGGCCTGGAAAAAAATAAATACATCTATT

>51474

TGCAGCTCTTCAACAGCCGGATAAACGTTTCTCATTTGTTCTGCCTGGGAGACAAGAACCCCTGATGGCAGCTGGTCTGTGGAGTGACAGAGAGCT

>59443

TGCAGTCCCACGTCAAAAGCCAGTTTGTCTGTAGACGATCGTGAGGTGGACAGACAGCACAAGAACTAGAATGAGGAACAGAAAACAAAACAAGTT

>183781

TGCAGGTTGTGTGAGGTAAAGCAGCTGCTTTTTTGCTCTGTATATGTTTTTGTATGTTATAACCAGCTTCTCTTTCTCTCTCTCTCCGTCTCTCTC

>211928

TGCAGCTGCTCCCCTGCGCATCCTCAACAGGCATTCTGCAACTCAGACGTCGGTGAGTTCACTTTACGGCTACCATTTAAGATCGGAAGAGCGGTT

>100113

TGCAGTGACGGCGGTTTGATGCTAATGTTTAGTTTTTCCTGGAGCCGGAATAGTTGATGGGATTTTTCACAGGTTCTGCTCAGGATCGTTGGGTCG

>126873

TGCAGTGCCCGGGGCGCAGGACGCCCTCAACCGGTAGGAGACACGCCACAAACCAAAAGAAAAGACTCTACTTACACTTTACAAAGGCAGACTTTT

>90923

TGCAGACGCCACGAGATGAGAGAGGTGCAACATGACGTTCGGTGTTTAGGCCCGTCATCGTCTTTTTGATTTGCCATCGTGCGCTTATGATGTGTT

>183988

TGCAGATCCACCAGAAAACTCTCCGACAGACAAGAAGTCTAAGAAGGCAACTCCGACCTCAGAGAGTGAGTTTCCAAAATATGCAGCCGCACCAGC

>155310

TGCAGAGTTATTTGATATTCCGGTATTCAGATTTTTGATCATTTTCCAATCGCAATCAGCTCATAAGCAGCACGCCGACCTCAGGAATGCTGTACG

>215568

TGCAGAGCGACATCTGTACGCTTCATTTACGCTTTATGATTAGTACGATGTACTCCTGATGCACAAAGAAATCCAGATGCCATTTCAGTGTGCTTG

>52243

TGCAGAGCATATTTGGTTCCGAAGAGTTCGTCCGAAATGAGTCGAAATCTGCCGGAGAATTCCGAGTTTCCGTGGAGCTGCCAGCGGGATTCACTG

>95345

TGCAGTTGTTATGGCAGCAAAGTGGTGTGACCGGCCCGGCCCGCCCGGTCTTTGCCGAAAGAAGGGAAGGAACAATGTGTCAGAGGATAAGTAGTC

>338702

TGCAGTGGAGCTAAACATGCAGAGCGTAATGGCTGCTGGACTTTTCGTCTGCATCAAGCAGCTTTCAGGAGTAATCAGAGTCCATCAGATCTCTGT

>239126

TGCAGGATTCGTAGGGGTTTTTGCTCCAAGCTAATCTCTCGTGGCGATCAAAAGCTGAAGGCCCACCAACTCCGTCCTCCTGACTTTCACTCCTCT

>139497

TGCAGGCTCGGCTCCAGGTTCATTACTGCTGAGTGCTGTTCTGCTCAGGCATAACCGACTCTCTGGCTCGCCGTCACTTTGATCAATTTATAATTT

>155041

TGCAGCAGGTGGGGAAAACCAAACTTTTAGAAACAAGCCACTGGAGATAAACTACAAGACAGAGCGAGGGTGTGACCGGTGGGGAAGACCAGATCT

>332616

TGCAGCCCACGGACGCAAATGCACCCTGTGCCCGCTTGCTTCAAATAAGCAGCTGCAAAGCCAAAAAAATAGAAAAGAAAAAAAGAGAAAAAGAAA

>127152

TGCAGATATGACACAGTTTGTGTGCCGCTGATTACACATCGGGCACAGATGGATGGAGCCGGCGTCGGGTGAAGATGGCCGCTTTTGGCACGGCTG

>265069

TGCAGATGTTTCATATAAGCCACCGCCAGGAAATATGAGACCTGAACTGCATCTGCAAAGCTCTGTATTTGACTTACTTGGACTACCTTCATACTG

>276390

TGCAGGTGGGGACCACAGATCACTTCTCGACGGTTGACGGACTTGTTTGGACGAGAGTCAGGAAGTTCGGGAAGAAGGTGGACAAGCTAATCAAGA

>42493

TGCAGACTGGGTGTGGCACTAGGAAGCTGGTGGCAGTAGATGGTGTTGTTGGTCAAATTGCTGCACCGAGGAATCGCAGATTCAATGGATAATTTG

>135504

TGCAGTGGCAGGTTTCCATCGCATTCTGGTCAGCTAAGCATGGAGGGAGGCGCAGCAGCAGCTGAGGACGTGCAGCTGTAACCCGTTCGCTCTCAT

>117650

TGCAGAGGACCGCCTTCGGGAAGGCCGTGTACAACATGACCCATTCGGAGAGGGTGATGGACGACCTGACGTTTGGACGGCGCGTGGGATTTTACG

>174373

TGCAGAAACAATGAAGAAGAGGAGAAATGCTACTTTCTCCCATTGTGCAGTTTGAAAAATAAACCCTGCTCAGCTCGGTGGTTATTGTTATGTAAA

>15813

TGCAGGATGGTGAACCTCCATCTTGACTTCAACTGTTTTGAAACCTCTGACCTCCTTTCCATTTGCTCTTACCAGTTTTCCTGATGAGGACACCAC

>152591

TGCAGCCTCAAACGGTCCGAACAGTAAATTCCCATGAAACAGAAACTAAAGTCATGGACACTCAGTCTGGTCAGCATCCGGACTTCATCACTGAGA

>317085

TGCAGTGCTGATATAAATTCTATAACCAGCAGTGCAGGTAAATAGTGCTATAGGAAAGTGTCGTTCCTGCTGATGGATATGTTGAACAAATTACCT

>41857

TGCAGGTTCCAGCAGTGAGACGTCAGTTATTCACTGACTCATGGTGTAAAAGGAACCTGAAAACACGAAGCAGCAACTGTTTGCTCTTCATCACTG

>184020

TGCAGCTGTTTCTGATTGCCACTCTCCTCTGTGACGTCTGAGGAGGAATTCAGGTCTCACTGGCCGACCGATCCGGTTGCAGAACTCGGGTCCAGA

>118783

TGCAGAGATCGAGGGAGCTGGCGCCTCCAAAGAGCAGAAAGGGAAGAAAAAGAAGAAAGCAGCCAAGAGAGAGGACTTTGAGTAGGCCTTCATCAG

>173370

TGCAGCTTAGGGTCACCAAATATCAACCACTAAACATGATTTACACTTCGTTCAGTAGTTTGTGAGGCACTTACAGTATAAGAAAGGCCCTTCTAT

>58951

TGCAGGATATGGTTGACATTGACACAGAGGTACCGTATGAGGAGCCCGAGTTGGATTTATGTTTCGCTGTTTGGCAGTTATCTCCAGAGAGATGTG

>26437

TGCAGAGACGTGCTGTTCTCAGAGAAAGGAGAGCTGAAGAAGGCGGTGATGGTGTCGAAAACCACGTTGATGATGTATTTCTCCAGAACCGGGTCG

>70985

TGCAGTTCCTCTGGCGGCCACTGGGCGCCGCTGCGAACTGAACTTCTCAAAGTTTATCCTCCAACACAGAAAAAGAAACAGGTTCTCATGTGAAAA

>129078

TGCAGCGTCGCATCCCATGCGCGGATCCCGGCCACAGGGCGGTATGCCGAAGAACCGCAACACATGAGTGACCGACGGAGCCTCCCCTCGCCCTCG

>78328

TGCAGCTTTATCTAGCCAGAACCAGAACCAGAAACTGGTACCGTCTGCTGCTCTGGGGCTTCTAGGTGAGATTAGCTCCACACCAGGTGGAAAGAC

>275384

TGCAGGTTCTGGAGTCCGGTGGGTCGGTGTCGCCGTCGGTTCTGTTCGAGTACAGAGCGAGGAACGCCAGCTCTCTGCCCAGCTCCCAGCTGGACT

>30379

TGCAGACAGGCTGAGAGGACGTCCTCAGTTAGGACAGGAGGGACAGACGGGGGACAGGGACATGTCTCTGCACCAGGACCAGAGTAAGGACTGTCC

>138962

TGCAGGTCCAACCAGGCTCTGTTACTGATGTCTCAGTTCATGAGTCAGAGCACCGGATGTCCAGACATCAGTAACACATGATGCACCTTGTTGCCC

>224522

TGCAGGTTCGATTCCTGGAGCTGAAACTTTCCCCTCGGTTTCTGCTGAGCTTCAAGTTTCCTTCACTGATCTCAGATCTGGAGGTTTTTCTTTCTG

>18708

TGCAGTGACATTTTAGGCACTCTAACAATCTACTCTTTGGTCCAGACAATTATCTGTCGATGTTTGTGGTCTTTGGGCATAAAGCACTTCCATAAT

>266034

TGCAGTGTCTTTTCCCTTTGGTGACCAGTTCCAGCTATGCAGGAACCGGTGACCGGTTCCTATTGCAGCATAGCTGCATAGGGTTAGGTTCCTATG

>260440

TGCAGTGTATTCACTCGGGGACCTTGATACAAACACGCATCACATTTTTTGGTTTCCCTTTGGAAATCCAGAATTGGCTTTATTGGCGAAGATTGT

>209472

TGCAGATTAGCTTCAAAGGAGGACGAGTGTAGCTGGACTGCTGTCACCGCTTGCATGATGTCTGGGCCCCAACCAGAAACCCCGTCAGTGTTGAAG

>266027

TGCAGTGAGAGGAAAAACTATTATGGGATGGTGATGCGTATTTGGTCAGGATTGATTTTACTCTTCAAACTCTCCTATCGAAGGCATTTCTGTCCA

>5268

TGCAGGCCCCGCTGGCAGCCAATCAAATGGGAATTGGGACACTTATAGATGACAATAGGGTTTGGGACGTTGGACTTCAACTCAACTCATTGAATG

>216584

TGCAGAAGAAGAGCAACTGTTAGCAGAAAAGTGCTGAAGAAGCTAAATTGTTTATGGATCATATTTTATGTGCAATAAACGAAAAACACAACAGTC

>150765

TGCAGCTTCCTCAAACCCGGACCAGGAGTGTAGTCGCAGTCGTGGTTCTGTAGAACCTGTGGTGACCCAGCATCAGAGATGCAGCTTCTTCTTCTG

>207123

TGCAGTCATCTTTCCTATTGATCCCCCACTGTAATGTAAAGCATCATCGCACCCTGGAGGATATCATCTGACAGTCTTGTCAGGCATCATTAGAAC

>190883

TGCAGAACAAAGTAACGCTAAATTGGTGTGGCTGCATAGCTTCGTTCTGGCTGATGTCCATGCTGATAAAGAGGCTTAGGATGCTAGTGTTTCTAT

>43497

TGCAGATGCTGACTAAGTCTATCGGCTGGACTTATGCCGCAGTGAACTGCTTTACACCCCCACCCTCAAAACAAGAACAAAGAATTTTATCTCCTC

>228630

TGCAGGTTCGTGTGCAGGTGTTTGTTCTCAGGATGAACCGAAGCAGAAACAGAGACAAAACCGGGCCCAGTTCAGAACTGTTCACCTGAGGACATC

>121151

TGCAGTCTTTTTATGAGTTGTTTAGCACATGGCTATCATGCAGTGCTAATGCTAGCCAATACAGTGGTCTAAGATGAAACCAACAAAAGGAGATGT

>153062

TGCAGGCGCTGCTGGTTACAGCGCTGCAACACATCGCTTTTTGTTTGGCTGTTACACCATGGACGCCCACCTATTTATACTGCGGGATTTGCAGGT

>17260

TGCAGCAGAGATTGGAGGAGTCACATGAAAAGCAAACATACCCTGAGCTGTGCTGCATGAAAGGTGCCAGGGTTTGTTTTGGTGGGGTCTATTTTT

>87587

TGCAGGAGAAACTCGGTTTCTGGGATGTACCTTTGAAACCAGTGATCAATTCCCAAGATTGTTCTGGTACAGACAAAACAGAAATTATTTCCCAAA

>32125

TGCAGATGTTCTGAAAGTAGCATCCAGTCCAGGCCTGGTCCTCTGCTGGACACCTGAGAAATGTAGTGCAGTGTGTTTATGCAGGGCTTCCCACCA

>48470

TGCAGGTATGGGGTCAATAAGGTGGAGGATTTGCTGCGGCCGCTTGTTGACAACGGTTTGAAGTGCGTCCTCATTTTTGGCGTCCCAGCAAAAATA

>216800

TGCAGGCAGGAACACAAACTAGACAGGGATTCTTTTTTATCATTACTACACAAATAATTCGGCCGAGCTGACGGTGACGGCTGTGAGCCAAGCACT

>209576

TGCAGGGCGCCGTCTCACCGTCCCGTTGTGCGGCTCACTTCGGCTCTTCTGCGCCAGGCCCAGCAGGCCCCAGTCGCTGACCTCCCTGCACCAGCC

>225097

TGCAGTAGTCTGTGTTAGCAGCGATTTTGTGCGACCTGGCCAGAATTTTCCAGGTCGTGCACTTACATAAGTCTGCGTACAGGACTGCGGTGTTGG

>228381

TGCAGACATTTACAATACGAGTAAAAATCAGTGTTACTGGGAGCAATGACCATTAGTGAGTGGAGGTTGTTGTCGTGGCTTTGAGTGTAAACCAGT

>267259

TGCAGCACATTTCCAGGTTTTTACTGGAAATCTACCTGCCATAAACCCAGTGCTTGACCGTTTAGCCTGAAGGTTTGCATCCACCGAACTGATCAA

>250322

TGCAGGCCAGCGCAGCTTCCTGAAGGAAACACCTGAATCTCCCCCGGTTCCCCGAAACGGCGCTGATCCGCCCAGCATGAGCGGAAGTCACTGCGA

>201257

TGCAGAGACCGGATGCTTTAGGCTTCCTTGTCTTCTAAAAAGAGGAAGAGACAAAACCATCTGAATTCAGAGGAACGATGAGACACCAGTTTGTCT

>296022

TGCAGCTGCTGCTTGCAGTTCGTTACTTTGCAGCTGGATCGTGACTCAAACACAAGAGGCGTTTACAAAAAGTCACAAATCCAAGGATTAAGATCG

>251939

TGCAGCAGCCGCACCCACCATCAGTGGGGAGCTGGTGAAGGCAGTTTCCATCGCAACCGGGTGTATCTCTGGATGGAATTTCTTTTCGCAGACAGG

>281752

TGCAGGATGTAGACCGATCCGGACTCCGCACATAGAACAGCAGAGGAAATGTTTCCAGGTAATCCAGGAAGTAGACAGAGCCCAGAAGAGAGCTGA

>67918

TGCAGTTGTTGCCCTCGCCCACCCTGTCCACCTCCCCTCTGCCTATTCTTTGCCACTGGGCAATCCCTTCCCACCTATTGAGGACGTTCCCATCCA

>166928

TGCAGGATATGTGGCATATGGCGGACACTCCAGCTTCTCCGGCCAGCACCTTTTGCTTTTAGGAAGTTTCTGTGCATTTGCATAAATATTTAAGAT

>251837

TGCAGCGCCAACATGGACGCAAGTCCTGCTGTCCAGCGACAGAGATGAAATCTCAGTCGCTGCTGTAGATATGTCTGAATGTTTGCTATGCGGCTC

>113969

TGCAGTCTCACCTCCCACATACTTCCTGCTCCAACGCTTCAGCAAATACTCCAGGAAGACTTTGTCCATAAAGCTGTCAGAAACATACAGAATATG

>115013

TGCAGAATACTAATGATCACTCGCAATACACTGAAGTGTGGTGGAGTTCCTCAGACCGAACACTGTTGTTTTTGGAAGATAATGCAGTGTATGTTG

>114513

TGCAGGGTCAGCAGTAGAGCATGTATCTTTGTGTAAAGCACTTGGCTATGAAGGGTGCTACGTAAACAGAGTTTACTTTACCATCACTACCGTAGC

>206925

TGCAGCCGGCTCACGCTCTGCGTCGCCGCGGAGACGGGCGTGAACAGCAGGTCCTTCTCCTTCAGGTAGACGCGTCCTGTCAGGGGGGTGGACGGG

>37083

TGCAGCTCAGACTCTAAGCTGAGAGTTGGACTTTTATGAAGGGAGGCGGTTCGGTTACCGTTTCCCAGACCGACTCTGGTTCTGTTCACATGAAAC

>291325

TGCAGACGAACCTTCATCTTTTTCCTTTCCTTCATCCTTCTCAGCACTCCTTCCCTGCTTGTTTCTTCCTCCTCCTCCTTTGTCACCTCCTCCTTG

>181599

TGCAGCGCCCAGCAGGATCAGTCAGAAAAACGAGACAGAAAATGATGGAGGGCACATGGGGGCCACAGTCCAGACTGGTTTACAGGAGCTAAAGTT

>167085

TGCAGTCTGTTCTTTGCAGTTTTGATGGGAAATAAATTGTTTGGACCAGATTCAACACAACACATGGGTGTGTGTTGTTGTCTGTTGGCCTTTTTC

>18040

TGCAGCGCTGGTGGAGCTGGTTAGAAACAGCCCCATCAACTCCGTACAGGACCTCCAGCAGCTGCTACACACTTATTCCGTAGGTAAAGACCCAAA

>267042

TGCAGGATGCCACAGACACTCTGGACGTCTACTTGTGGAGACATGCTGTGAGTTTCTGACTTTCATGTCCTTGTCAGACAGACGGGTGAAATCTGA

>81670

TGCAGCACTCACTTCAACGCCCTTCAAAGCCACCTCAATATTTCCCCCTCGTCCTCGTCCTTGTCCTCTGTCCTGAGTGGCTGACACCAGCTTGTC

>285317

TGCAGATGTTTTGTCCACAATGCGCCGCCACCAATGGTTCTGTACTGAGAACGAAACGTAAAAATCTTGCTGGAGTAGAAAATCGAAGATGCTTCA

>182429

TGCAGCAAACCAGTGAAGTCTGGACTGACAGACAGACCGCTGGCCTCGAGCTGAGCACCAATCAAACGTGACATCACTGACAGGTCGGATGCTGCT

>109842

TGCAGTGGTGAACTGCTCGTTTGTCTCTGTTACCAGTTGAGTGAGCCACAGCCTCTGGTGCTGAACATGTTCTGTTACTGGATGAATACTGACAGA

>257931

TGCAGCTCCAGCTCTGCGATGCCCAGCTGCCCGTCCTCTTCGGTCAGACACCAGCGAGCCTGAGCGAAGTAGATCTCCGTCCTGCGCACCACACCC

>60589

TGCAGGCTGACCCGCTGTGCCTCTCCTCCTGGGTTACACAGGCAGAGGGAGATACTCGTTTCAGGTCAAGCTTCTCCAAACAGATAAAAACAGATA

>43438

TGCAGCAGAAGAAGAGCAGAGCTCTGATTGGAGGACGTCACTGGGAAGCTCTGAAGCTGCTAATCAGTAGAAAACACAAACCAGATGCTTTGATTC

>302560

TGCAGCTAAAACTGGCGCCGACCGGCCCATCTCTCTCGGACCGCTTGTTTCCATAATAACCCACCAAGGCCCAAAGCATTACGTGGACACCTCAAG

>16663

TGCAGCAGCAGTTCGACCTCTCTGTATGACTGCTGCGTATACTTTGGTGTTTTGCCAGTGGAAATTCTGAACAATATTTCATCCAGACAGGCTGTT

>214248

TGCAGGGGCACAGCAGCGGAGATCCCAGCATCACCCCCCATGGATCCGCAGAAACATTCAGCAGTCACCAACCGACAGACAAGATGGTCATTTGTT

>253191

TGCAGAACTTCATGGCGTCCACAGTGGAGGTTACTTCGTAGGAGACAATATCCACTGAGCTCAAGAAAGACTGAACGCATTCCCCGGTGAAGTGGG

>260708

TGCAGACAATAAAAACGTCTCATCCAGATTCTCGAGTCGAGGCAGGCCGCTGTCGAGTCCCACATCTGAAACTTTTCATGTCCTTTCAAGCATCAT

>220449

TGCAGGTCCTACATGGGGGGGATGCCCTGCCAGCTGCTGAGGCCCAACTCAGATGCTCTGTAAGTGTTCAGGATGTTTGACCAGATCGGTCAGCAA

>128650

TGCAGCTCAGAGTCCAGCATGAATCCCATCAGGCTTCAGATATCTGGACATGGAGAACTGGGACAATTCCTCCTGACAGAACTGGGTACGGTTTGT

>328819

TGCAGACGGTTCAAAGCCTCAATAATCTGCCCCTCAGTGAATTGCTGCTCTGTTTGCGCTGCGGCTCGTGTTTATGTCTGTGTATGTAAGGCCATT

>96897

TGCAGCTCCTCTGATGGTGCGCAGCGTGGTGAACTCCCAGTCCATCCCCATCCCGGCGGCGGCGGCCGACTCTCCTGGAAGAACCAGACAACCTTC

>64720

TGCAGACGCAACAGCAGTAAAAGTGTTGACTGAACATGCCCTGCATGCCCTTCACTGTATCATTTGCCCTCCATTATTATTTTCACATCTTCTGAC

>288890

TGCAGCTAGCATAGCCTGTCATGAACGCATATTTCAAGTATTTCAAGAGACCCAGGAGATCCAGGACAGGAGGTCAGAGGACAGGGATTCCCCAAA

>168619

TGCAGCAGGCAGTTCAACATCCTGCCTGAGACACTCCCACGTCAGACGGAGCTCAAACGTTTTCACCAACACAACCGGCGGTACCAGAAGCTGGTC

>308071

TGCAGGTTCACTGAACAGAACCAGGTTGAACAGAACCAGGTTGAACAGAACCAGGTTGAACAGAACCAGGTTCACTGAACAGAACCTCAGCTGGTT

>159960

TGCAGGAAGAAGGAGTGTCTTTATGCTCTTGTGAGACATCATGTGATTCCTCCCACCCTAAATATGTCCAAAAAGCCCCTAAAAATTACCCTCATT

>193018

TGCAGACGATGGCCGATGTTACAACACAAGAAACTACAAATGAAAAGATTCAAGCCTCCTGAGACGGTCCAACCTCTTCTTGCTCAGCAGCTTGAG

>3326

TGCAGCAGCATCTCAGACTGAGGGCGAAAGGGAGAGAGTGCAACACCCTGTGAGCAGCATAGCACCTCATGTAAAATAGACACGCACTCACAAACC

>328657

TGCAGGAGGAGGAGGATGATGATGATGAAGAAGAAGAGGAGGAGGAACAAGAAGTCAAGGAGGTAGTGAAGGAAAACAGTGTCCCGCAAGGTAAAA

>307018

TGCAGCAGCTCCTCGGGCGGCAGGTGGGTCAGCAGCACCTGGAGGACCGCCTGGGCGTCCAGACAGTTCCTGGCGTTGGTGTTCCACACGACACAG

>313589

TGCAGATGGTGTAGCAACAACCGCTAAGGAGCCAGAGAACTATAGAAGCAGATTCCTGCCAAAATCCGCTGGTACAGAATATGACAAGTTCACAAA

>109801

TGCAGGGCCGGTGTCTCTGCTTCATCTTCAGGTCATTAGCAGGTCTCCGGCCCCGCAGGCCTGCATCCTTGGACTAGGCCGTCACAGAGGTTCAGA

>29005

TGCAGGATCTCCTCCAGGAAGAACTGGTTGGCCCGTTTGGATCGCAGGAACACGGCGTGGGCCCCAGGAGCCACACGAAACCCTAGAAAGAGCGAG

>33898

TGCAGATGAAGGCCATTGCTGATATTATTAGCAGCACCTGAGCTTGTCCCGCTATGACAAATCAAAATGTCTGCTGTGATAAAAAGGCCTGTGGCT

>162371

TGCAGAGCTCATAAATAGTACAGGATTGAATTAGGAAAAACATCTTAGGAGTCAAATGTCATAACAGTGGCACAACTTGCAGTTTCAACGTCGGCG

>211425

TGCAGCCGATGTTTGGCTCGGCGGTGAATTGTGAAGGTTACAAGCCGCGTTATGAGTGCAGGGAATCCCTGGTTGTGATGGAGCGGGACGTGGAGA

>215650

TGCAGTTAGAAAACCTCAGGTGATGATGCCTAATAATAATAATACTTCAGATGAGTCAAAGAAAGACATCATGGATGAGAATCAGGAGGAACTTCA

>227311

TGCAGTGCATTACCAGATCTTTCTGTTCGTTAGCGATGCGGGTTTCCTCCTCCAGCTGGTCGCTCAGCTCGTTGATCTTCCTCTCCAGTTTGCTGA

>299314

TGCAGCCGCTCCGGATCCATCGATCACCGCGATCCGCTCGCAAACGTGACGCTTCTGTCAACGCCGGAGTCAAGAAACAGACTGGTGGACGTCTGG

>196258

TGCAGGCTCCCTCCTTCTGTCCCTCCCTCTCTCCCTTCTTCCTCCTCTCCTCAGTTTCTTATTCCTGTTAGTCCTCTTTATATCTCTTCTTCTCTG

>225343

TGCAGTCAGTTATATTGGTGATCACTGTTTCTGTTCTTCTTGTTGTCTCACAGTTGTTTCCACATCCGTCCTGCTGCTAATCTAATCCGGTTGCAT

>305633

TGCAGGTCAGAGCTCATACACAGGATGTGATGTAATAAGTTGTGGTTCTTTCTGGTCGGCTAGATGTGTCATTCAGGGACAGCCATCAGAAGCTGA

>217927

TGCAGGTTGAAAGCAGATGTATGCGTTCGGTGCCTTACAGTCCTTCAAACATAGAGCCATTTCACATTCAGCTATACAGGTACAGGCCATTTACCA

>194352

TGCAGCGCTTTGCATCAGAGTGCATGTGCGTGCATGCAGGCCGCAATCGGCAGGAGGATTGTGTGTCAGGTGGAACATGCATAGGTCAACGCTGGT

>330640

TGCAGCGCGTTCAGAACAGAGACCCGGTTCATCCGTGTTCTGCTGTCTGAGGCCCATTACTCACGGTTTCCTGCTCTGTTATGGTGTTGCTAGGCA

>13125

TGCAGGGATTTTACAGCCTTTTCCTCAGCGAGTTCAGGTTCTGCCGCTCTGCTGGGCTGGCATTGGTTCCTGTGATCCATCATCTCGCAGAGTCAC

>147335

TGCAGATTGCTGCTGGGACGTTCGTGGTAGCTTGGAGTGGGAGGTCTCTGACATCTGAACCAATAAACTGACAACGCTGCATGCCTCTTTACTGGT

>301464

TGCAGTCCAACCTTTTTATCTGTTTCTTGCCCTCAAACGATGCTAGTTAGAGTGAACAGCTGCCTCGCTGTCCACCATTATTTTCTCACCCAGTTC

>76503

TGCAGGAACGCTGGTGACGATCCGAGGCCGGATCTACTCTGACGTTTACGGGAGCAACACGGACCTGAGCTCCAGCGGCCTCAATGTCAGGTTCCT

>64074

TGCAGATCCATCGTTACTCACAGCTTCTCTATTCATCTGTTCAGGCTCTTCCAGCTGAAAAAACATGCTCAGATCCATTCACATCTGTAAGGCCAC

>184055

TGCAGAGTAAGGCAGAGTAAATGAAGATCAGATGCTTCACCCACATTCAGGTTACATCACAAAGCAGGGGGGAGAAGATGCCATGAGGTCTGATAA

>52615

TGCAGCCTCGCTTCATTCACACAGAGCTGCGGGCAGAGCGACTTCACCCTCCCACTTGTTGGTCACATAAGTCAAGATCAGTGTCCAGAGGTGCCG

>177157

TGCAGCTCGGCCCCTTCGCTGCGACGAAGACGGGAAAATAACTGGATGAAGATGAATGATTCAGCACCTCCAGTTCTGCTCTGCTGCTTCCTGCTT

>104148

TGCAGCACACACACTCAACCCCAACACACACACACACCCCATCACACACACACCCAATGGATCAGTACTGGATGGACTAAACCAATCCCATCAGAG

>114066

TGCAGGTTTTCTGAAGCGAAGCGTCTTCAGATCATTCTCTGTTTTCCCACTTTTCCTGAAACGTTTTTCACTTTGATGCCAAGAATCTCACATTTC

>82812

TGCAGTGGAACTCAGATACATATTGTGCACTATACACACAGTGAGGTGAGTGTACATTGAAAATAATTGTTCGGCACCTGTGTAATCGGTTTGTTC

>241854

TGCAGGGGGGCGTGAACTTTTGCGCGTCACACACTTTATATGGCTTTTACACGATTGTTTCCAATCTTCGGCGAGTCAGCCAGAAATGTAGATGAT

>111296

TGCAGCCCAGAGGGGAGGCGCACTGGACCTCTGAGGGGACAGACAGAAGAGCAGCCAGCTGGTAAAACATCCACAGTTCACATCCGGAGCCTGCAT

>39867

TGCAGAAGATGTGCACGAGGCTTCACATCTTCTGCTTCTCTGGATCAAATGTGTGCGGTTTTGCAGTTGCCCATTTCCACAAAAGCTGCTCTTTGG

>174428

TGCAGCTGCGTGTTGACTCTTCAGTAACTTGTTCAGCTGGAGGAAGTCGTGGCACATCCACCCACTACCACATGTAGAGAAAATAAACGCATCTGT

>75530

TGCAGAGCTTCGGCGACCTGCTGAGCGACGCCTTCAGCGCCAACATGGAGCACACCGTCTACGGAGACTTCAAGGCCTTCCGGCCGGCCCACGACC

>255801

TGCAGAGTAAAGTCGACTCCTGGTCGTCGCTCGTCGTTCCGTTTGCGATGATCATTTTCTCCGTCGTGATCCTGGAGTTTTACGTCGAATCCGTCT

>50782

TGCAGCATGCAGAACTGGGCCGAGGTTCTGCTGCGTTGCATGAGGAGCCTCAGTCCGACACCAGAAGGTTGAAGGGACGGGTGGTTCTGATCGGGT

>280563

TGCAGTGTCAGGCAGAGGGCATGGTGCTGACACTAATACGGTGTTGTGTGTTTGTGTCGCAGCTATTCCAGTCAAACCCCGGCCGCTGTCGGAGGA

>286502

TGCAGCCAGGAACAAAATAAGGTATTCATCTGTTTAGAGATATCTACCGAAGATAAATTGTAGAGCATGTCTACTGACAGCAGGTCTATGACCCCT

>313610

TGCAGCATCCAGGAGGAGAGTCCAGACACGCTGCTCTGCCTCAGCCCCGCCCACCCCGAGGGGCCGGCCGCCGTCAGCGTCCAGGTGTTCGCCGTC

>1170

TGCAGCCGAAGGTCCAGAGAATCGTCTGACCGGGTTTCAGTAATCAGATTACAGAGCCGCCCAGGAATTACAGGTTCTGGTTTCAGACAAGCAGCC

>191665

TGCAGACCAGTGACCCAAAACCAACAATGTAAATAAAAGCCATATGTGATTCTGGATGAGTCACACCAAAGATAAGATAAAGTGAAAACCTAAACA

>87207

TGCAGGGACGCCACGACTCGATCGACGGCCGGATCGAGGGGAATCAGAGCTGAGACAACCAAGGGACACGGCGATGTTTTATCTGTCGACACAATA

>207558

TGCAGTGAAAACCTAGAAGTTAGGTGGTGGCACAATGGCTGAAGCAGCTGGGAACTTTGTTTGAGGGACGGTGGGGTGTATGTGGGTGCTGCCGTC

>313086

TGCAGTCCTGCTCTAGTTGATGGATCCAACAGTGCCTCACATGATACAGAAAGCGATTCTGAACTGATCCACATGCACTAACTAAAGGCGTTTTCG

>227898

TGCAGTCTGGCCGACACCGATCCACACAACTGTAAAAGCAGACAAAAATGATTTATTTCTGAACTCGACCAATTGAAACTGTTTATTAGTACTTCT

>312993

TGCAGCTCCAGGATTACAGCCTCCAGCTCCTTCTTCTCTTGTTGGCTTTGGCTCTGAATTTCCTCCAGCTGGGATTTCAGCTTCTTGTTCTCCGTC

>171783

TGCAGGCGTGTTGGGGAGAGGGTGGGGGTGGCTGCTGTCTGCCTGCCAGTCACTGTTCTGCGATGCATCACTTCCAGATGTGCAAAGCTTATTCAT

>146595

TGCAGAATGGGTTTTCCATAAACAGTGGGTGTTGTGTTTGGGTTTTGTGAAACGGCAGAAACAGGGGCCCCACCCAGAGTGCCGTTCATCCATGAA

>61083

TGCAGGAAAGCCTGCGCGTTCTCGGCTCGTCCCGGCTGAGATGAAACCCGCTGCTGGACGCGCGTGACCTTCGAGTCCTCAGGCTGCGCCGCGTGA

>258573

TGCAGCCCGGATCCTTCAGTTTGGACCTCCAGCCCTGTAGGTGGTTCCTGTGAAGGATCACAAAGTAAAGTGACCGACTCGGTCCACTGACTGGAT

>218739

TGCAGGCCTGGAGGCACGCAGCGCTGCTGAACACCCTCCTCTGCTGCCAGCCAATCCCAGCCGGGGACGAGAACCCCGGTTACACCGCGAGGCTGT

>185099

TGCAGCATGCAGACCAAACTCTTCCAGTTTTCCTGGAGGACCATGAATGCAGCACAGCTGTTTCCATTTCCTGGTTTGGAGTGAACCAATCAAACG

>317558

TGCAGTTATAAGAAAGCTTCTCTAGATGTGGAAGAACGGCGCCGCAGTTCTCACTGGTTGTTTTCCACCACAAACCGCCAAAACTTTGGTTTAAGA

>16861

TGCAGCAATCTTCAGAAGTCTCACCTTCCCGTGCTTCTCCTCCTCCCTGCCATCTCACCCTTCTTTCATCTCCCTTCTTCGTAATTTCAAGCTTCC

>130746

TGCAGCGTTCCTCTGGAGGAGATCATCATCTGTCTTCATGCTGGTGCAAAACGCCTCGTCCAAAACAAACATTTCACAGAAACAACGTCACATTCC

>211865

TGCAGAGATCAGAGGAACTAAAGAGATTATGAAATTATCAGCTGCTATATCCCCGTGATGAAAGGTCAGAGGTCAGAGGGTTTGGTTGTCCATCCT

>310546

TGCAGAGCAGTCAGAAATTAGGGGATTGTAATAAACTCAATGTTTACAATATAAAACCTGTATTTTCAGTTCACTTACAATAGCTCCAGGTTATCC

>36149

TGCAGGAACACATTGGGGGGGCAGTGAGGGGCGGAGCCTGTGCAGTACTCAGGTAGGTCGCACTCTCCCAGCGGCTCCCGGCACACCGAGCCCGCA

>76131

TGCAGGCTAACTACCTGGTTAGCCACCCCAACCCGCTCAGCCTCCCAGTGCTCACTGCCATCGTTGTTCTCAAACGTGAGTAGTATCATAGCAACC

>233921

TGCAGCGGCTTCCAGAGGCCTCTGCCAGCTCCCGTTATTTCCCCTGAGCGCGCGATGATGGCCTCTCTTGTTGCATTTTGTTGCGCCGTTTTAAGA

>215297

TGCAGGCACTACAGCAGCACAGAGAAGACAAATGGAATCAACCAACCAACCTTGCTACCCTCAAACGGCATCCCTTCAAAGAATCTGTAAAGAACA

>173296

TGCAGAGCATGAAAAGCCCAGATCCAGACCATTGATCCAATAGATAACTTGTGGCGTGAACTGAAGAGAAGCAAACACAAGACATGATCTTGAAAG

>137921

TGCAGAAGGCGCGAGCAGCAGAGAGGCCGAGGGCAGCAGGCAGAAAACCGGCTGCTAAACTGGACCACAGAGCGCCGTTCAGAGTGACCGCCGCTC

>246605

TGCAGTCTCTCCAACCAAGCGTTTCATCAGAATACATTTTTTTTAGCGTTACCCCTTACACTGCTCTACTGCAATTCCTTTAGGTGCTCCGAGCAC

>101005

TGCAGACATTCACCCACCTCCCCTCCCCTCCAGACATGCAAGGACTCTCCCAAGTTTCAGCCCCCCGGCACCCACCAGATATTCAAAGGCTCCTAG

>106603

TGCAGTGGGATGTTGTCAATAACAATGTCAGCTTCATCCTCCAATATAATGAAACAGAGTTGAATATAGCACCACCTGCTGGACAACAACCATTAC

>139145

TGCAGGATTCCAGCTTATATCCAACTCAGCCTGTAGACAAAGGCAGATCAACGGTGTAAGGATTTCCTCAGCTTGTCACTAGAATATTTCAGCACA

>234910

TGCAGGGCCTGGCGCTTGTGTCGCACTTTGCTGCCGCTGGGACGACCCCTGTGCACCAGCAAGGACAGCATTTAGACGCAGGCAAACAACCGGACA

>188558

TGCAGGTCAAACTGGTTTCTGACTCACACAGAAGTCGCAACATTTAGATTTTGCTCTTGCAGCCTAAAAGCTTTCAGGTCAATATTCTGCTTATGC

>185667

TGCAGCAGAAACCCAACCTGGTGGTGGAAGCAGACGAGCTGAAGAGGAAAACTGATAAGAATCGTCACCAGACTGGCAGCTAAAAGGCTTTAAGAT

>187772

TGCAGGAGGGATAGCCGGGAGGGTTGCTGGATCTTATCTGTGTGTGTCAGGGAGAGGGACAATGCAGCGGATAGATGCCAAGCCAACTGCCACTTC

>71775

TGCAGGTACTTCTTCATGAGCGTGGTCTGGTCCACCACGGTGGGGCTGGGCGTGGGGACGAACGCCATGGCGACGGCTGGAGCTGACCGAGCAGCA

>146804

TGCAGCGGCAGCTGCTGCTCCGCCAGGCGGTCAGAGACTCAACATTCACAGCAACGCTTCATTCTTCCCTCTGGAGCCGGAAATGTACAACTTCTT

>46975

TGCAGGGTCGTCCTGGGTGAGTGCACAGAGGGGTCAAGAGGTCAACCTGAACACTGACCGGTCCTGACTGACTGTGTTGTGTCCAACAGAGAAGGA

>184188

TGCAGCTCCTAGAAGGGCTCATCTCCGAGGTTGGACTTCCGTCCTCCGCTTTGAAAGACCAGCAGCAGAATCCTGCTTCAAAGCAGACGGGCAAAC

>279717

TGCAGCTAACAATTTTTGAGGTTGTTGATGGTGTTATCAACAACTGTTGAGGTTGTGGATGGTGTCAGCTGACCTAAAGAAGCAAATATATTTTTG

>105978

TGCAGATGGACAGCACCAAGCTGAAGGTGACGATGAGGAGGGTGCTGATGATGTGATCACCCCTCCAGAGGAACTCTGACCCTCTGCTGTCTCTTT

>257663

TGCAGGTGAAGGACGATCTGAAGAGTCTCTGAAGAACTGGCACGCTATAGGAAGCTGATGTGCTTTATTTGATGCTTGTGGTTTGATAATGATCTA

>306165

TGCAGCGGATGTGCGCTCACCACTGTTAGCTGGTTTTCAGGGAAAGCTTCATGGATCTAAATGGAAAACAACCAAACCAAAAACAAGCTTTCATAA

>8652

TGCAGGTTTATCCTGTCTGCTCTGAGTGCGAGCCAGAGGAACGTGGTCAACTGCGCTCTGTGCCACCGCTCGCTGCCCGTGTTCGAGCAGTTCCCC

>154967

TGCAGCTCATTCTAGGTGTCCTGGAGGTGTTTGATGGAGTTTCTGTCAGGGGTCTGCACAGGCTTGTTCAAAGTCCTGTTCACACTTTTATCAAAA

>112508

TGCAGCAGCCAGCAAGCAGCTACAGACGGGGCTGGCTGCGTCAAGCTGCACGCAAAAAACCCGCACGCAAGAGCGGAAATGCTTCCTGAAAGTAGA

>40806

TGCAGTGTGGGCAGGTAGTGGCGTATGAACTGTGTCCAGAAACGATCTGATAACACTTGACAGGTCCTCCAATGTCGCTGTCCAATGAGCTCTGAG

>236054

TGCAGGGGAGGGATCCAATATGTTCTGGAAGCAATAAAGGCTGACAGATTGGTCTATGGAGTAACAAAGACAATACAGAGCAGGGGAAGATACACA

>167028

TGCAGGACAGCAGGTGCTGCGCGTCAGGATGTGATTCGTTCACGTCGTTATGTAGCGCCACATCAGGACGTCCATGCCAGAAATAAACAAGTAAAC

>23245

TGCAGCATGACGCTCTGGCTCTGCACCTGAGGATTTCAGCAGATGGTGAAACATGATCCAATCACAGAGGGATAAGCTTCTATGACTCATCGTAAA

>88859

TGCAGGTCCAGAAACGCCCGACGGAACCGGCTGGCGCTGAGCAGCAGAACCTCTGAACATTTTCATCCTCTCTCCGGATTCCTGCTGGATCCGGCC

>93426

TGCAGATGATGGCCCAGCAGAGGAAGTGCAACGCAAAGCAGAGCAAAGGGAACCAGACACTCCAGGGTGCATGGCGCACTGACCACAGACAGGGGG

>309884

TGCAGAACCCGGCCAGCCTGCAATGCCTCGACGATTTCAGGAAGAACCCGGAGCCGCAGGGTGAGTCCTGATGAGTCCTGATGAGTCCTGATGCAG

>86229

TGCAGTTTTCAGAGCTGCTCTGCTGGAACCAGGCATGGTTCTGTTTCACCGAGTAGGAACTGATCAGAACCAGCTCTAACTGCAAAACCTGGTTCT

>253989

TGCAGGTAGGTGTCAGAAATGGGAGGCAGAGTATAGAAACAAGCACATAATTCAGACAAGATGAACACGTAGGCATGTATCCACTGTGGTTATACC

>56496

TGCAGGGCGGCCCGGCAGAGGGGCTGCTGTTGTGAGTGTCATCTCAGAGGGTCGGGGTCGGACCTGTGAGTCGGAGCACTGACCCGTGTGTGTGTG

>37814

TGCAGACTCGGTCGAAGTGGCAAAAGGACACTTTTCCTATTCAGATGGGTGCTGTGGTGATGATAGTGGATCCACAACTCCCCAGAGCCTCATGGC

>158536

TGCAGGTTGAGCTTCACTACAAAGTGAGCTATGACCAATGCGCATACACTGACATTTGGTCTTGCAGTGGAAACTGTGGGACTGAGACCCAGATCC

>203243

TGCAGCATTATGTATGAATGTATTTCTGCTTTTCACCATGTGCTTCATTTAGTTCATCTTGTACCATGACAAACCTCCTTCACAGCCTACAGCAGC

>233592

TGCAGGGCTCAGTTTGGTCTGGTTCTCCTCTCTCATTGCATGTCTGCTTACAGAGTGTCCACAGAGCACATCTCTCATTATACAAAGCTGTTCAAA

>67044

TGCAGTCAGCCAGCTGCTGCATGTTGAGCGACGAGGTCACGCATTCCTGCTGGATTCCGCAGGCAAAGTGCGGGAATGGTTGACAACAACAGAGGG

>101724

TGCAGCTACTCCAGTATTATCGTGGGACTCTTCCTAATTGATGTAACGGCCCTTTTTCTACACACCCTCATCATCCACTCGACTCTGAACTTGCGA

>205310

TGCAGCTTCAGCCTCAGTTTCCCAGACCGTCAGCAACTGGACCGTCCAGAAAACACAACGAGCCTCTGATCCAGAACCACAGCCTGACTCAGCGCT

>280829

TGCAGGCGTGGAGACTTCAGCAGGAAACACTATGGTTCGGTCGAGCTGGTGAGTCCCACCCACGATGCCCCCATCAACCTGTTGCTCTCTCTGCTT

>104139

TGCAGTATTGACTCGCGGCCACTAGATGGCGTAAAAGTCTGAACAGTGAGAAGGCAAAGACAGCGGACTGAGAAACGAAGGTTTATCTGTAGGATT

>50070

TGCAGCAGTGCCACTCTCATGCGCAGCTCCTCCAGCTGTTGTAGCTCCTGCTGTGTGCTGTAGGTTACGGTAAGGGGCAGAGGTGAAGCCGCCCCA

>309743

TGCAGGTTACTTCATCACCTTCCTCACCATCTTCATCATAGTCCTCATGATCGGATCCACAGCTTTCAGCAACTGGTGGCTAAGCTACTGGCTGGG

>237916

TGCAGCAGTGAAACCAGTAAAGAGGCATGCAGCGTTGTCAGTTTATTGGTTCAGATGTCAGAGACCTCCCACTCCAAGCTACCACGAACGTCCCAG

>33223

TGCAGCGTGAAGGTAGGGGTCAGGGAGAGGTCAGGAGAGGCGTTCCCACGGCAACACGCTGATGTCACACACACTGAGGCTTCAGGAAGCCCCACC

>69224

TGCAGCTTCCTCTCCCTGACAGAGAGCCGCAGACGTTGGCAGGATTGGAGCCCAGAGCAGGGCAGCACTGCTTGGACCGGATCCCCTCCACTGTGC

>69630

TGCAGAGAGGATCGTCAAGGCTGAGGACCAGCACAGGTCAGCAGGAGGGAAGCCAACATCTCTGCCGACTCAGAGAGCCCAAATCCATATCTGTGA

>116522

TGCAGGCGACTGATGGCGAAGCAGAGCTCATGTTCCTGCCGCCGCGTCGGAAAGGAGGAAACGGACACAGACGCAAACCGTCGGTGCGTTTAGGAT

>220102

TGCAGGAACTGGACCGAACCAGAACCTTCAGCCTCTCTGAGAGGTTCTGGTGTTTATCCTGCCGGGTCTTGAGGTCCGATCCAGAAACACTCGATG

>52633

TGCAGATCCTCACCACCACAAAGGATGGACAAATACAAACAGCAGCAGTCTGCTGCTGTACAGAGGTTGAAAAGGAATGGAGAGAGAAAATATCTC

>91811

TGCAGTGCTGGGTGGGAGTCACTAATGAATGAACCGATGGACAGGACAGGATTTTATTGTGGCGCACTCAGCACTCTCCTTCCACTCCTCTGATTC

>68950

TGCAGTGCAATACTGAAGTACTGTAACGCTTTCCTGTCGCTACCTGAGCTGCTCAGCTGCCAGCCTGCCTGTCGCACCATCAGGGACCCATCTGAC

>33200

TGCAGGGCAGGTTCTACGTAGCGTTACGATATACAACTCTTCTGTCTCATAGAGGGGAAAAAATAGAAAGTCGTACATCTAAATGCGACCGCCGCC

>154640

TGCAGGCAGAAATCATGTATTTGAGGAACACACTCAGAAGCACAGCTGTCTAATGAGTCTGCATTGTCCTTACAGGAGACGCCACGGCTTCATTCA

>136195

TGCAGAGATGTAGCTTTCAGCCATTCAGTCTCAGAGCAGAGTGCAAGGCTGCATGAGTTACCGTCCAAAGACCGTAAAGACTCCGAACTCTAATGA

>136388

TGCAGACAAGATGAAGAGCAACAGCCTGCCAGCTAGACTCACCCTGACAGCACGGACAGCGCCAGGTTGGTTCATACTAGGGCTGGACGTTTTGGG

>235912

TGCAGGGTTTGTCGCCTGGTTAGTTTGGTGTGTTCAGGGCGATGTGCAGTGTTAGATTTCAAGGTAGAGAGCCACGTCGTGGTAATTTTGCAGTTG

>270564

TGCAGATCACGGCATGCTGTCTGTCCCACAGTACTTTGCAAGGAAGCACACTATCAGTTGCACCATTGTGTTTCAGCAAGGGGTTAAGATCGGAAG

>203339

TGCAGGAGCGACGTGAGTCAGAACCGCTGGCCGGACCGTTTGGGTTCCGGTTTGCTCCGGCGCCACGCTACCAGGAGCGAATTGGATCCCAAAGAA

>98537

TGCAGCTCTTGCGTTACTGCGAAGGTCGCCACATGCAACCAGAGGAATTGTGTCCAACTGAAACCAAACCTTCCTAATCTGAATTCAGATGTTCCC

>98413

TGCAGCACCAAGATGCTGCGGTGTCAGAATCTGAACATTCTGACGTTTCTCCAGTTTTTCCTGAATTATTTCTGCTTCCTGTCGATGAACCGATGC

>246276

TGCAGGATTTTGGGGCTGTGAAAGACTTTCTGAGTCGGAGTAGGTTCTTATTTGTTCTTTGTCTAACTGCTGCGTCACGCTGTGTGGAAATGCCAT

>118987

TGCAGGTCAGCTTCCCTCGCACGATATAGTCGATATGCAGCAAGAGAGCCAGCAGAGCCAGAATAATTTCATCCGTTTGTTCCTTGCATCACTTCG

>258374

TGCAGAAGCAGCAGAAAGCAAAGGTTTGGAAACACAAATCTAGATAAATCCCAGTCTGCATGTTGGATCACAGCTACCCCAGTATTTACTCATGAC

>41237

TGCAGGAGACGACATGATGATGACTGACGGCTGCGTAGCGAGACGGCGGCGCGGAGCCGTGGCTTCAGCTCCCACAGGAGGGAGGGAGAAACAAGC

>56958

TGCAGCCGTTTCTTCTGTTGGTTCCATGAATCAGACCTGAGCTGCTCGTTCCCGTCCCAGTAAGAGTCTGACTGGTATTTGATGAGGAAGAACAGC

>78868

TGCAGGTCCTGGAAAATACGTTATAAAAGGATTAGAAGTGAGCAGAAGTAACTTTGCACTTTTAGAGGAGGAGCTCTTGGTCTCAGAGAAAGTCAT

>130830

TGCAGAACATTCATAGTAAGAAACTCCTGAGACTGGGTTGGAGGTTCGGTTCCTTGACATTCATTTATTGCTCTCTGCAAACCAACTGGATCGATG

>71728

TGCAGTTCTTTAGATCTCTTAGAGATGGGGCACGTGCTCAAGGGTCAAAGGTCACTCATACTAATACGCGCTTGTTCTAAGAGGATCCTGTTCAGC

>279431

TGCAGCTTGTCCTGCCGGACGGTTTGATGGTCGTTGATGTTCTGTTGGAGCGTTTCTGTCGGGCTCCTGGGAAGGTTCTCCACTCAGGTTCTCTGG

>151452

TGCAGGGAAGAAGAAGAGGAGTAAAGGAGGGAGGAGGGGATAGAGAAGAAGGTGAGGTGGTGCTGCTGCTGGTGGTGGTGGTAGTGGTGGAGGTGG

>271329

TGCAGAAAAGAAGCCAGCTAACAGTTCTATTAGCACCTGCACTGGAGCACTAATGACTGTGAAGATGCCATCCAGCGCTCCCTGGCACCAGCCGGA

>107252

TGCAGTCAGAACACGCCGGGGCCCGATGTTCTGGTACCAGAAGCTACACTGGACCGGCTCTGAGTTAGAACGATCCGGAAAATAAAACCGACTGAT

>240963

TGCAGTTTGTCATCGCCTCCGTCTCTGCTGGTTCAAAGTGTGTAATAATGACAGTTGGAAAAGAGGTGCTGCTTCTTTTTGCTTCTTCTCTCTGTG

>225020

TGCAGATACCGGGTCTGTCTGTACATCTTTGACGTTTGAGTTTGCCTATTTTCATCATAAAAGGACTGACAGTCTGAATGATGAAGTTATAAAGGC

>187277

TGCAGATGGGTGGGTGACATGACCGCTGATGGTCCTGTCCATGTCATATTGGGGTCAAAGCTGAGCCAGAAGTCATCAATATTCTTCTTCTAACAG

>145097

TGCAGCTCTCTGCTGTCAGAGGTCCTTGTTCCAATACCAATCACAATGACGCCCTGCTGTTTGAGAGCAGAGGCTGGGGCGTCAACATTGTCAAAA

>145833

TGCAGCGCCGTTTATTTTGCCACATCGCTGTTTGTTGTTCTGATCTTTCGCCCTTTCTGTGGACCGAACCGCACGCAGCCACTGCATGACTGAACA

>24037

TGCAGGAAGCAGTTCCTGGCTGCTTACTGCATACCAGAAGAAAATGTTTTGCCGTGAGATGTGCAGCAATATCCACAGAGCATTTCTATATGTCAC

>61262

TGCAGGTCCGGACGCTGAAGTGGAACCGGTTCTGGTCGCAGGGCGGGACGACGTCCGCGTCGGTTCCTGGAGGAACCAGGACAGCGTGATTAGAGC

>48789

TGCAGAAAACTGGAAAAATCCGAGCAACAAGTGTGTACCACGGTAGAGATGAGCAATTCATGCGCCTACATCATGCGTTCATGTAACTGCTGCAG

>138336

TGCAGAGCAGAGATGGGCTACAGTCAGAACATGTGCACTTACCTGTGTGCACATGAGTGCAGCTCGCCGCTGACTACAGGTCCAGCTGTAGAATTT

>73939

TGCAGTGCCTCTTCTCTGGCCACCCTCATCTTTCTCTGCTTTCTCTGACTTTGTGCCATAGGATCCTCATTGGTCATGAAGCTGATTTGGCCGAAC

>35635

TGCAGAATCGTTTTCCTCACTGTGATTCACTGATTCTACCTGAGGCAGAGTCAGAACAAAACTTTCCCAAAGGTCCGGATCTGATTCTGGATGATT

>225053

TGCAGAGAAGTCGAGGAGGCTCCCAACCAATCACAGCAGGGATACAGGTGATGATGCAATAAGAAAGGGTGATGTCACAGTGATGATGTAATGACA

>62001

TGCAGAGACGCGTTCGGATCCGGTCCGTCGCAGTCAGACGTCCAGCCCCACAGTGACGCTACATGAATGCGTCTCTCACAGCCAGATATGAGCAGC

>242256

TGCAGTAGCCGCACTCACCTCTTTCTTCTGTCATTACTCTGCGAGGAGAAGACAAAGATGACCCACAGTTCTCTCAGGAGCAGAGGCACAGATGGA

>155056

TGCAGCCAGGACTGATTTTACGCTATAATGCTGACCTTTGTTCTGAGAGATTTACAGTGGATTCCTGGGACCACATCCATATCAATCGCAGAGCAA

>124319

TGCAGTCTAATGCCTCTTATGTCCTCCATTTTTGTCTCCAAGCAGCCACTTTCTAATGCATTTTTTCCTACATGCTAAGTCTTTGACTTTTTTATG

>313967

TGCAGAGGAAAAATGTAAAGTCTACTGAAGCGACGTATTCCAGATATGTTTATGTTTGGAACCGATAAAGTCCCGATTACTGGGACTTTGATGCCT

>81163

TGCAGACGGCCAAGAAGTCTGACAGCGCCCTCTACTGGTGTGAGTCTCCGTCCATGCAGCGGAGCAACACGGTTCAGGTCACCGTGTACGGTAGGC

>321367

TGCAGTCGTGTATGGGTTGGAAGCAGATGTCATATACAAGTCCTCAGTTTCACCTTTAGATGAACTCCGTCTCTGTTCTCAGCTGAAAGTTTCTCC

>143824

TGCAGCAAGCGCCGTGCCCTTCTAATATTTGCAGATGCTGCATATGGGAATTGCGTCAGGCGGAGAGAAGTGGTTTACGCTGGCTGCCAGATGTAA

>175203

TGCAGAGCTCTGCGGCTCAGTGCGCCCCCTGCTGGACACGCCGCGTGTCACGTTCTGCTGTAAATATGATGACGTCAAAATAAGAGATTTTCTAAC

>284534

TGCAGCTTCATTAGCCTGTGTGCTAAAAAGCATCAGAGCAAAGGTTTCACCAACAGCTGCACTTAGTGGACAGTCATGAAAATACAAGTAATTCAT

>155097

TGCAGTATATTGTTCTGCAACATCATCATTGGCTTCAAGAACTTTACTGGCTTCTGCTGTGAGCTTTGCAAAGTTGTCCCTGAGCTCTTCCTCAGA

>298040

TGCAGAGGCAATGCTTCAATGTTGTCAAACTGCCACTGTAACAAGAAGTGATGGGGTCCAATCATAAAATCACAAAGGGAGGAGAAATCTGCACGG

>126675

TGCAGCTGCCTCCACTTCCTGTCCCACCTGGGCGACTGCCTCGGCAGCCTCTGAAACCGCCTCGGTGGCTTCTGCGACCACCTCAGCTGCTTCCTC

>312061

TGCAGTGGAGGTGGAGCTGTTTTGGGGAACCTTAGCGTAGAAACAAGACAACAAGAGTTATTGCTTTAGAGATTTCTGTGCTGTTCTCACAGTGTA

>91835

TGCAGACCTGTGAGTGAAAGAATCGGTCACTCAGTTGCATGAAATGTAATAAGAACCCTGTCTGACTCGTCGCTGTAAATAACGAGCCTCTGGCTG

>195254

TGCAGAAAAATAATCCTTCATCCCACTCCTTTTTCCATTTCATTCTTCCTTGTGTCCTTCCTTCTTTTTTGTCCTTCCCTTCCTTTCCTCCTTGTG

>257836

TGCAGGCATGTGATCCAAGCCTGCATAGGAACCTAACCCTATGCAGCTATGCTGCAATAGGAACCGGTCACCGGTTCCTGCATAGCTGGAACTGG

>41657

TGCAGATCAAACAGGAAGTGCAGCATGACAGATCAGGAGACTTCCTGCGGGAGCCTTACATCACCACCAGGGAGCCGGGGAAAGTAGTTCAGTTCA

>71245

TGCAGTAAACAACGATTATGTCTCTATTATCTCAACTACTAATACCAAAGCTATAAGACAAACTAATTCTGTTGATGGTGGTGACAGAAATTTGTC

>3364

TGCAGGTTTGTTTGTGATGAGCGGAGCCATCATCTATACGCTGATGAGTACTGACTGGGTCCAGACTGGGAACTCGTTTGGGTACGCCTACATCCT

>97751

TGCAGACCGCTCCCAACGCCACATGGTACAAATTTTCAAAACAGGTCTTCTCTGACATAATCCATGGGTGTGTTTGAACCTGAATGTACAGCTCTC

>253210

TGCAGCCTCAGAACCGGGTTCAGAACCGTCAGCTCAGATCCCAGCTGGGCTCCACTGGTTCCCAGTCCGGACTGGTTTTTGCTGGGCCGTGCGTCT

>181725

TGCAGAGTCCGAGATGGAGCTCTTGGGGTTTCCACAGAGTCTTGAAGTGCTAAAGGGTCTGACCCTGAGGTGAACTTGCTGGGATGTACTCTCCTG

>55994

TGCAGCAACATGAAGCAAACAACAGGAAACAGGAAGCAACAGGAAACAACAGGAAGAAACAACATGAAGCAACAGGAAACAAGAAGAAACAACGCG

>112679

TGCAGAAGTCCTACTGCCCTCCAGGAAAATGTGGGCTTGATTGTAAAATTGTGTTGCTCCTTCCTAAAGCCAAACGTTGTCTGGCGAACACATTTC

>330108

TGCAGCGGCCCAGTCAAAGTAAAACATCTTCAGGAAGTTGTTTCAAACTTCAGGGACGAGATGACCTGGAAGCAATCCGACCGGATCAATTATTAA

>124348

TGCAGCTTGGTTTCCCCCTCAGAGATCCTGAAAGACGTTTTTCTCTGTGGCTCCTGGAAACCACACACTGACTCCCAGCAGCTCCATGGAAACCAG

>66417

TGCAGCTCAGACCAGAGTTCAACCTTTGACCTTTTCCTGGGACGTTTAGCGAACGATTCCTGCTGAGATAAAAGCCAAAGGAAACCTGTGAAGCTC

>143621

TGCAGCCGACCAACATTTTCCTAAAAGCATTTCTCTCTGAACATTGGGAGACAAAGGGCTGTCAGCAGTCAGAGACACAGTTTGTCTTTGAAAAGA

>177234

TGCAGACGTTTACATGAACAAACCAAACAGCCTGAAGGCTCCGAGCCGAGAACCCGCCGAGGTGAGGAGCCGATGTGGTACCAACGGTTCTCCTAC

>138532

TGCAGCTTCATGCTGATTTTTCCGGGATTATCCCACGTATTTGGGAATGCAGCCCTCCACCTCCACCATCTCAGGCCAGCTCTCGTACTTGTTTTT

>129508

TGCAGCTGTCTGATGAAGAGTCATCATCATCCTCGCCAGACGAGGCGAAGAACAAACAGAAAAAGAAAACTGATGACATCACAGTGCGGCCAGGGT

>149906

TGCAGCAACTATTTGCTTTCTTGAGGAGCTTTTGGCTCAGTCAGCAGAAAGACGGGTTTGAATCCCGACCTGAAGCCTTTCTGCATGTGGTCAGCA

>89551

TGCAGCTCTCTCGAGGGCGGTGCTATGGTAACCAGATGAACTGAACTACCACCATCACCCCCCACCCCCCTTCAACAAGCTCCTGTCGCCAACGCA

>7077

TGCAGGTCGGGGAGAACACGCCTTCTGGCCGTCCGACACCAACCTGATCGACAGGAGCAGCATCAACGGTACCAGAACCACATCTCTGCTTCTCAG

>91082

TGCAGCTCCTCTTTCAGCAGCTCCTGCTGCACCTGGATGACCCTGACAGCTCCATGCAGGAGCAAGTGCTGGGTGAGACCGGGTGGGGTGATGATG

>46289

TGCAGGAAAAGCTGCTGAGAGCCAACGACCGCATTTACTGGCCTTATTTCACTAAACCTGAAACGTTCTGTTTCAAACTCACTATGGAGACAACTG

>57434

TGCAGCGAACAGACCCGCTGAGCTCCTTTCTTCATATAAAGAATTGGACAGGGAGACTTCAGGGTACAGCGCGCCACCTGTCAGGTGTAATCCTTC

>8194

TGCAGGTTCAGGTGGAGGACCGGTTTGGGCTCAACGGACAGAACCAGCAGAACGACACAGCTGCTGGTTTCCTCCAAGCGTTCAGTGGATGCTAGG

>256354

TGCAGAGGTATTCATAAATCTTGAACTAAATCTTGAATCACATGTTGCTTATTTGGATTTTTACAAAAACAGATGAACACAAAGCCGCCTGTAAAT

>332313

TGCAGTTCGCTTACAACCAGGTTCCCGACCCACTCAGGACCGGTGCCAGACGGGTTCGGAGGTCGGTATTCTGCTCAGCATGGACCGGATCTGCAG

>6198

TGCAGCTAAACCAAAGCAGGCGAGACGGCGGCGAACTGGGGTGCCCAAGACGAAAACGGTCCTACCAAAGTCCTACGTGATGAGTGTCTTCAAGCA

>294658

TGCAGCCACGTTAGCATCAGACCGCAGCCTGACCCGCTGAGTTATACGGGGATTCTCAGTAAAAATATACAGAGAATCTCAAAATGTTGCAGAGAC

>267829

TGCAGTAAAAACGTCCGGCAGAAGCGTTGTTGTCCGGAGAAGTTTGCAGCAGCGAGGGTGGAACGGGCAGCTGGGTCCTATTGAGCCTGATTGCGA

>18304

TGCAGTTGAATTCATTAGCATCAACAACTAGCTACAGATGTTCACTTTGATGCCGACGTTCAGCACCTGGATGAAACACGTCTCACCTAGTGAGCA

>301094

TGCAGGTTTGGTTCATCTGTCACAATAAAAACATTTTTCTTCTTCTTGTGCTGTTTTAGTGCGTCTGACCCAAAAGGCACCGGCTGTCCTTCGTCT

>240565

TGCAGTGTGGAAGATTGGACCATGCCTCAGTTTTATATACTGAGAGTGTTGAGAGCTGGTACTAGAGTCACAGCGTCTGGCAGGTTTTGGTTCTGG

>212707

TGCAGAGCTCAAGCTCCACATCAGAATAAGTCCTTTGTGTGCCTGTGCTTGAAAGAGCAGCACACCGTGAAGCTACTGTAGGACTGTTTTCCAAAT

>205496

TGCAGAGCGTTTCAGAGAGAAACGGAGCCGAACCGCAGCCCTGAGACACGACGACGAGTACTGAGTACTACTGACAGGTACTACTGACAGGTACTA

>133929

TGCAGGTTACCTGATCGTTTCCATGGATCCCCTCCCACCCGTCGTCGTCGGCGACACGATCACGTTGAAGTGCAACTTCCAGACGGACGGCACCCT

>237051

TGCAGCCTCACCTGCTGGGACTGCGGCTCCGGTGGGGGGTCGTCGAAGCTGCCGGTGTGGGTGGGGAGGGAGGAGTTCTGGTTCTGGTTCTGGTTC

>64715

TGCAGTAATCTACTGCGTTGGGCAAATACAACACAACGTGAAGCAGTCAGGAATCAATCCCATCATGCTTTGTGTAAGGAAAGCACAATTCCATTG

>108265

TGCAGGTCACATGAGGACAAAAAACAGAGCGAGGAAAAAGGACAGGAATAAATCTGTCGGTGTCTTATTGTGTGTGGGCGTAAACACATGTCAGTG

>200348

TGCAGATAACTTCGGGTTTCTGCCTCACTATGTTGGCATTTTGATGGAATGAATCCAGTTTGTTGGCGCTGATTCCGTTTTGCTCTGTGAAGTTGA

>326236

TGCAGGCTCACACTCAGGAGCAGGGCCAGCAGATTCACGCTCTGAGGCAGGAGCGACAGGCCAACCAGGACAAAACCAACAGGTTGCTGTCGCTTC

>293952

TGCAGATTGCGTAAGTAAGACAGATCAGTCGTATACAAACTAATCTCTATAGCTTAGTCAGCCGTTACATATTCAGCTTTTTTTTTTCTTTTCTTT

>18114

TGCAGGCCACGCCCATGTTTGTGTGAAATTCCTGAGCCGATCAGCTGGAGACGCAAACCGTCACGGACAACGAGACGGGAGGAGAAACTAACCTTC

>39938

TGCAGAGTTGGAAAGCTGGTGGATTCTAGCTTGGACATTCAGGCGATCCAGCTGCCGGCCCTTCAGTACCAACATGAGAGACGCTCCCATGACTGG

>179496

TGCAGGGAAATCCGTGTCGGTGCAATAAGTCACTTCATTCATCTAAGTGGCTCGGACTCGGTGATTTCTGTACTACAGAAGCAGTGAGGGATCTGT

>182702

TGCAGCCGGAGACTCGACGTTTGTTGAGACGTAAAGTCGCCGAGCTGGAAACGTTTTGGTTCTGATTGATTATTGATCCTCCCAAAGATTTTACCC

>216025

TGCAGGGGCGCTGCACCGCAGCTCCTCCACGAAGTAACGCACCGCCACGCACATGTAGGCGCCGTGGGAGACAACCAGCGCGTGGGCGTGGAGGGC

>61130

TGCAGAAATCAAAGCTCCGTCCGACGCCAGTCAGAACAATCGTCTGTGATATCCGCGTTACAGGGATGCATTCACTAAAAGCTCAGCTTTACCAAG

>206007

TGCAGAAGCATGTTGCTGTCCAGGCCACCATGCATCCATTTCTGTGCTGCACAGACACACACATAAGTGGCGTCATCTCGTTATGAAACAGCAGGA

>10051

TGCAGGAGAGCGATCGCGAGGACGACAGAAAACGCAGCATGTGTGGCTTGACAAGACAGTAGCCATAACGCAGTTGACACAACATGTCAATTAGTG

>159523

TGCAGTCTATTTACAGGTTACAAAAACTAAAACTTCTATCAGAATAAGAGCACAAAGCCAAACATCTGACCATGTCTTGCATCAACACAACTGCTC

>19125

TGCAGGATTTTAGGTCATGTCAGCTTCAGTCCTGGTGAGTTGCAGCTTTTCAGCTCTGTCCAGGATGCTGACATGAAGCCAATCTCAACATATGCG

>13663

TGCAGAACAAGACAACGTTACCCTACTGGAAAAATGACTCTGGGGAGGCATCAGCTTGGCAAAATGGCTGGCATGGTTATGATAATGAGTTCCTTG

>290366

TGCAGGAAGCATTTGTCCTTTTGAAATGGAGCGAGGAGGGGAGGAAAATGAGGTGGCTGAGGCGATATGAAGGTGTGCAAGGCAGGAACAGAAATT

>45960

TGCAGGAATGTGTGGCCCGTCAGAGCCGCTGAACGGAGCCGAGGTGGCGGATCAGGGGCAGGATGAATGATCCGGACCCATGAACCTGGATCGGGT

>272953

TGCAGTGTGTGAAACGGTTCATGTCGAAATCATCTGAGGCAACTCAGGCACAACTAAAGACTCCCGGGTGTCGATCTGGCCTCTGTACCTGGGAGA

>46499

TGCAGCTGGATGGTCACAGCGTCACCGCAGGGCCCGTGTTTGGCTACTGTCACTGTCAGATGGCAATGCTGGAGTCATGAGAGCAGGTCAGAGGAA

>72948

TGCAGCGCTGGTGGAGCTGGTTAGAAACAGCCCCATCAACTCCATAGAGGACCTCCAGCAGCTGCTCACTGACTCCGTAGGTAAAGACCCAAAGCT

>21091

TGCAGGAGATGATAAATGAGCGTACCTGTCTGCGCTCCCAACAATAAGCGACTGCTTTTTTCTGCTAAAGCGCCATTTTTAGGTTTACTCAGGCAA

>37296

TGCAGACAAGCAGCCCATTTTTCTTCATGAAACCAAACACTCTGCATGTGCAGCTGAGGACAAAATCCACCTGATTCCCAAATGGATCATCTCCTT

>84240

TGCAGCGCTGGTGGAGCTGGTTAGAAACAGCCCCATCAACTCCATAGAGGACCTCCAGCAGCTGCTGCTCACTTACTCCGTAGGTAAAGACCCAAA

>153800

TGCAGAGACGGTGTCTGTGTATGTTATGAAGGTTTCACGGGGCTGGACTGCTCTGCGCTGAGCTGTCCAGAAGACTGCCACTACCAGGGGCGCTGT

>141342

TGCAGAAAAAACGCACATGCACACACACTCGAGCGAGTTCTCTTTTCCGTCGCTTCGAATTATAACGTATAGCAGGAAAGCTTCTTCTAACCCCAC

>131637

TGCAGTGTTGTCCTCTTCTGTCTCCTTCATACCACACAAAGCCCAGATGATTCTCTCTAGCAAAGATGCTGTAAGTCAAATTATGGCCGCCTAATC

>113392

TGCAGTTGTGACCACGCTCTGACCTGCACGCCGCCTGTTCTCCCCATACAGAATATGACATGACGTACAAGCACTGCTTCGTCAGAGCTCATAACG

>247769

TGCAGAGCGCGCTCCGTATCTGGAGGAGACGGAACCAGACGGTCAGCGGTTCTGATGGGTCTGACCCAGAAAGAGTCTGGACCCGGTTCTGGTTCT

>218464

TGCAGATCTCCGGGCCTATGACCCAAATCCGCCCCTCAAACCCAGCCACCGTCACCAGCGTGCTTCTCCCACAGCACCCCGCCCCAGCTCCTCAAA

>92162

TGCAGAGCCCCTCCCCCGGTGTGACACCCCGCTCTGTCTCACCCTCCGATCTGACCACCGATCTCCCTGCGTCCTTCCACCTTCGCCTCCTTCCCT

>49873

TGCAGAGCTGCTTTCAACTCTGACTGGGAATGAAAAGTGGGAGAAAAGCGGAGAATCGTTTAGAAACCAATAAATCAACTCGTCATGAAAGGCCGT

>86709

TGCAGCACTAAGTGGTGGTGATCAAACTGGGTCTGGACCGGGCCGGACCGGACTGCTGAGGACTGGTCTCTGAGTAACTCAGTAGAAACAACCAGC

>152390

TGCAGAGAGTCTTAGCGTGATGAAGGCCCTCCCCTCAGGGCCTTGGAAGGGCATCATTGTCATCTTTATTGCATTTGTTTTGTTTGTGCTAGGATA

>254670

TGCAGCCTGCGCGACTGTATGAGCGGATGCAGGCCTGTTCATAAACTGTGTACCCAACACTTGCTCAGTGCCAAGACTCCCCCTGGTGGAAGGGAC

>172792

TGCAGCTCCTTCCTCTCTCTGCTTATTTCAGCCTAACAACCAGCTGATCCAAGCAAACCAAACTTTCTTCTCTTCTAATTGCTTCCTGTTCCAGAC

>217812

TGCAGCACAATCATGTCTGACTTGTCTGCTTCTCCAGCGTTGAGTGCAGGATGAGGCACTAGAGGCGTTTCTGGTTGGAACTTCCTGTTTCATCCG

>15864

TGCAGCTCCTCCTGTCGGCTCCTCAGCTCCTCCTCTATCTTCCTGAGGTGCTGCTCCTCTTCCTCCCTCTTCCTCCTCAGCACCTCCGCCCCCTCC

>92773

TGCAGTCCCTCTGAAATGCAGTCTGGTTTGAGCACCAAAAAGGTTTTTATGTCCCCTGATCTGATTGTGCTAAGGCAATGGGACATGAAGTATTGG

>323802

TGCAGCGACTCCTCATCCTCTGTCCCCTTTCTGTTTCTCTCCACCTTGTTGTTCTCCTTTGGTGATGCCAAAGTCAGGATTTCTTCTTGATGGGTG

>12330

TGCAGGAGCGATGAGGACAGTGTAGATGAGTCTCTGAGTCCCGCCGGGCGGCAGACCCACGACAGCCTGTACCGGGTCCACATGCCCAGCCTGTAC

>122069

TGCAGTCAGTGAAACTCTGACGAGATCCAGACTTCCAGCCGCAGCGTTGGGTTGCTTGTTGACACTCCAGCAGCTTCAGCGGATCCATCTGGTGAT

>175389

TGCAGGAAGGAAAAGCACAGCGGAGCTAAATCTGGATCCAATGTGTTCAACTATCTGGGTCATTCAATAAAACACAGCAAGGAAAGAATAAGAGGA

>64140

TGCAGCGCTTCAGCTCAGCTCCCATCAGCTCTGGTCAGATTAGCTCAGCGGCGCCACCACAGGCTGCTGGGTTGTTCTGGTTCCGGACCGCCTTCT

>148661

TGCAGCTCAGACTGAAGACTTTCAGCAAGACGACGTCCCAACAAACCAGGAGACCCAGTGGGAGAGATGAGAGCTTTAGACATGATGTCTGCAGCT

>270670

TGCAGCGCAGCGCAGCCCAAACCCAGAACCCGAAGAAAGCTCCGGTCAACGATGACAAGATTCAGCCTGAAACCCAGAAACTGATAGAGATCACTG

>119300

TGCAGTGTGCCTATAAGCGTGTCTAGGACTGGGGCTGGCTTTAGATCTTTTCTATCTAATTGACAGCTGCTTTGGCGAACAAATGTCTGTTTTATA

>328315

TGCAGGTCCGAACGGACTGGATCAGGATTCCCTCATCCTGAGGGAGGATTCGTCACAGAAAACAGCTTTGCTCCGGTCCTCTGCTGGTCATTTCCT

>52175

TGCAGGCAGCACTTTTGGCCACAGATCAGTTTTGCATGTTTTTGTTCAAATACACTGAAACCTCGTTGAAATCATTGTTACTTGAAGTTTTCTTTG

>200081

TGCAGAGCTCCCTCCTCTCTCGGTCGTTCTCCTCGGCTCTCGCCCGGAGCCAGGCCTCGTTCTGGGATCGATGTTTCTCCAGCATTTCCTTCAGCT

>285368

TGCAGTGACCGCAGGTGACAAGGTGTCGACATGTCGTCATTACTTTCAGTCAGGCTGGGTGGATTCACAACAATCAAATGATTCCTGGGTGGTTTA

>38684

TGCAGTCTTGCTCACACAGCTCCTGATTGCTGGATGACTCACCAATAATGTTTACAGAAATCTCTTCATGGGATAAACGCCATCTCAAGACTCGCC

>260290

TGCAGAAACAGGTATGTGTTCAACGTGTGTTGAACAGTAGCACTTCTAATAACATAGCGCTGTCGTTGTCTGACAATCAGGCTGGCGTACCTGATA

>206398

TGCAGTACTTCAGTGTGTTACGCTAGTTTCGTCTGTTACCTCCTGGATGAATTGATAAGTCAGTTTCCTGTGTGCTCTGTGGTTTTTGGCCTCACC

>251650

TGCAGCAGGAGAGAGGCAGTGAATGTGAGGAGGTTGACGGTGCGTTCCGGTGCGTTCAGATGTACCTTGGCCTTTCCTCCCTGAGCCTCCAGAGAA

>85982

TGCAGCTACCCACATGAATGAGGAGAGGAGAGCATGGCTCCCCTGAGGTTCCATGTTTTAGATTTCTGTCACCCTGGTGGTGGCAGAAAAGCCAGA

>138724

TGCAGGTTTGGGGTATTTTGGGTTCCTCAGACAGGATTGCCATATATTTAGCTAAACCCATCTTGCAGCTCTGACTAGTTCAAGCAAGCGTTTCCA

>52620

TGCAGAACCTGGGCGTTCTGGCGCTGGCCGCGGGCTCCAGGGCCGCCGCCGAGGATGAGCTCAAACAGATCGCCTTTCCCGACAGCATGGTGTTCA

>310335

TGCAGAACCTTGTTAGGAGTCTACATGCTGCACAAGCTCTTCCATTATGATCCCACTCTTCTTTTTCCCACGGCTGAAATCAATACTGTGTGAGAG

>267586

TGCAGCAGCAGCAGGCTCCGCCCCCTCCGGCCGCCCCGCTGCTGCCCGGCTCTGCCGCTCCCGCCGCCGACCTGGCCCCCCCGGTGGGGCCCGTCC

>197925

TGCAGAGGCTACGCTGGTACCCACAGATCCTGTCTGTCCTTGAGGGAGCTCCATGTTCAGGTCCAATCTGAACAGAAAGCAAAAGCGTACCATTAT

>276704

TGCAGGGTCGTCCAGTACTACTACAACCTGGGAGTGCAGGTGGGTAACTGGGGGTAACTGGTGGGTAATCGGGGGTAACTAGGTTGTTGCCACATT

>114457

TGCAGCATTTACAATTTACTCCTCTGTATTTATCATTGCTATTGTGTAAATATGGAAGAATTCAGACAATTTTGAGCTGGTGGTAAGGATTTTTGG

>208795

TGCAGAATTGACTAGACGCCAGGGTTTTTTGTTTATGTCCTGGCAACTCTTACACAGAGAGGATCGGATCTGACTGCCCACGCTCACATCTGAGGC

>203076

TGCAGGGGGCGCCATAACGATGTGGGGACATTTTCTCCCCTCAACTGTTTATGCAGTAGATATTTATCCGCCCTAAACATCCTGCATCCAACAGGA

>35971

TGCAGCTGTCCTGCTCCAGCTTCAGGATGGTCACCTGACCAGACTGGTCCCCTACAAAGGCGTGCCTGGTCTCTACATCAAACCTGGTCGAAGGTT

>202214

TGCAGTTGGTTTGGAGACAGAAGTTGATGCCAGTTCAGCAACATCAATAGTTTGATGCACAATTAGATGTTGAGGTCAACTCGTCTGTGAAGGCAA

>101635

TGCAGAGCTAAGCATGGAGCCACCAGATCTTTCTCTGATTTAGCTCCAGTTCCTTAGCGTCACCTTTCCTGTTGCCATAGCAACAAAACCAAACCA

>223102

TGCAGTTCTCTGAGCTGGAGCTGAGGGAGGGAGGAGGAAGAAGGCAGGAGGAGGAGGAGCTGGCAGACGGCCAGCAGGAAGCTGGAAGAAGGGATG

>99322

TGCAGAGGGGCAGCATGAGCGAGAAGACCGAGTTTGACCCCCAGGGTCGCATCCTGTCCCGGTCGTTTGTGGACGGCAAGATCTGGAGCTACAGCT

>175954

TGCAGGAGAGACGGGGCGGTGAGCAGGAACCGGACGCCGTTTTTCATCCACATTTAGAGCCACCAGAACGGGCATAGGGAGCTTCTGCAAGCTGCT

>6854

TGCAGGCCCTGAAGAGGCAGAGACAGCTCCAAAGTGGTTACAAGCTGTGCTGGAAAATCAAAATCAACAAACAAGACATTTTCAAATGATGTTAGC

>205181

TGCAGACTGTATTGAGACTGATAGGATAAGGTCATTACAGGCTAGACTAGACTGTACAGGAAGTTATGTATAATGCATGCAGTCTGCTAAGGAGAT

>9236

TGCAGAGCGCCAGCGGCAGCAGAGCCAGGCCCAAAGACCGGGCGAAACCCACCGAGCAGCACATCGAAAAACCAAAACACCAAAATCACAACGAGC

>256417

TGCAGCTGCTCCCCTGATGCAGTGATGTATGCCAAAAAAATCTGACTGCATTTGCTGGTAGGCAGAAAATTGCTAACGTTGGCAGCTGTTTAAGAT

>268011

TGCAGACACACAAACTGAAATCTCAGCCTGACTGGTTGATGCTGGTCTGGTTTCCGAATGTAGTTTAGACTATTTGATGGATGGTAAACTGATCAA

>140503

TGCAGCCAAATGGAGATGTTTTTTGTCTCCTTTCCCCCCTCCCCAACCCGCTGCTACTCAGGGCACTTGGAGTTTATTGTCATAAAATTGAAATAA

>297362

TGCAGGTCGAAAATGAACAGAACAAAAAACTTCATGTTGAGATCAGAGAATCTAAAAACTTCAGTTTAGCAGCAAACTGACAAAACAAAGTTCATC

>73416

TGCAGTAATGGTGGGGCTAACTGGTTCAACTTGCTCCATTGTACATAAATTTGTCGCCTGGTGATCATTGTTGACAGTTGAGGTGGATGATGCTGT

>154563

TGCAGATGCCACACGCCAACAGACATCCACAAACCCAAAGTCAAAATAAAACCAATAAAATCACGCCTGCCTTTGAAAACAGCACGCTCTCTCTCC

>241114

TGCAGACAGCAAAAAGCTGTGAAGCATCAGCAGCTAGCGCTGGAGCTCTTTGGATGATCCAAAAAAACTAGTTAGTAAACTTTGACTGTTCCAGGA

>89088

TGCAGTGATGAAGATAAATTCACCTCCAATGGGACTTGACTACTTTCTTAGGTTTTTCCAAATTGACACCATCTGTCCCAAATCTTTATCTCTCCC

>38130

TGCAGCGATGCTTTTTCATGCGTACAGGACGCGACTTGTGTTTCTGAAATATGCAAACCCTCCTCTAAAAAGATGTTATCACACCGAGAGGCCGCG

>108676

TGCAGGAACTGGACACACCAAATCATCATCACACCAGCAAAGAAGCCCCACGTTATCACACGGACTTCAGACAAACCCCATTCTTCATCAGTGCTT

>134880

TGCAGCAGAGCATGGTGTCGCGGGGCCAGTCGTCTATGGAGGCCACTGAGCTCCACCGTTCCGCCACAAGTTCAAGTCCCTTTGCTGCTTTTCTTT

>221435

TGCAGTGTGTGCAGAAATGTCTGGAAAGATCCAGGCCAGACTGATTTATCTGTTGGTCAAATGTTGTTTGCTAGTGTTTACTTTTGCAGTTTGAGA

>328091

TGCAGTGGCCAATAAAAAGGAGGAAAAATGTCCATGAAGAGGGTCAGATTCATACAGCATCATTCAGGTGTGATTGAGAGTCTGAGTGGGGGAAGC

>223427

TGCAGTTCTGGTCCTCCCACCCAGTGGTCCAGCTCCAGTTCTGGTTCTGGTTCTGTCCAGGAGCCCGGCTAGCAGCAGATGTCCAGCTGCTATTAA

>165739

TGCAGCAATTTTCTGCTGAGGTCTCCTGGTGCCGAGCCCAGCTATCGCTACAAACACAGTAATAGATCTACTGGCTTGAAAACAGACCTGTCCCCT

>114857

TGCAGGTAGCGGGCGAACCCGTCCAGGCCCAGCAGGCCGCGCTCCCGGCCCTGCGCCGACGGCTCGTAGCGCTTCAGCAGCTCCCAGCAGCCCTCG

>194421

TGCAGATGGATAACGCGCTTATTTTTCAGCGCACAGATTAGTCGGGAATTATGATGATACCCAGGGGGCAGGAATCTTATTTTCTGTGCTCCTGAT

>219774

TGCAGTCGGAGCTCAGAGGAAGTTTCACCAGAGCGGATCTGAAATGTGTCAGCAGCTGCCAGGCTGATCATCAGGCCTACATCTGGACCCGGAACA

>15511

TGCAGGTGTGTACATCATTTATGAGAGTAATTGAGAATAATATGAAGAAGCTCTGGAGTGTTTGTAGCATTGGGACAAATGAAAGCCATGGTGAGT

>46832

TGCAGCTGTTTTCTTTGCATGCAGGAAGGAGGCGAGCTGCCTTTTCTGCCAGCTGGACTGATGTTTGTCCTGCTCATCAAACCAGATCGCGTCCAG

>159863

TGCAGGTCATGGAGGCCTGCCAGGCCCAGGCTGCTGACGGGGTCAAAGGTCGAGCTGAGAGGCTGCTCAGCATCTTCCAGAGTGACCTGTTCCAGG

>92883

TGCAGCTGGATGAGCCTCGAGAGACCCCGAGATTACAAAGGCTTCTGCGACACACGATTTACGACGAACGTACGTGTGATCGATCTGTGCGGCAAG

>61845

TGCAGCCTCTGCACTGACAGGAAGGAGGAACGATGCCGACAAGTAAAATGTGCTCCGAATGTGGATTAGTTTGGATGCTTTGCTTCCATCGTAGAT

>118318

TGCAGAAAGTGATAGTCGTGCCAGCTCAGTTATCTTTCAAGTTGATCCGTTTTGCCCTTTGAAGCTGTTGGTCACGAGTAACTCAGCCAGCCTCCA

>254631

TGCAGCCTGTAGGGGTGCTGTGTTTCAGATATTCTATTCCCACTCATGGAAACATGTCTGTGGTTCTCCATTCCTCCAGTCTCTGTGCAGGGAGAT

>24654

TGCAGCCAGACGCACAGACCGACGGACGGCTGTTAGTCCAACTGGCCGGTTCTGCTGTCAGGACCGGATCAGCCCAGAACCGGGCACCGCTAACTA

>339112

TGCAGTGTCATGATGTTTTTATCTGCTTGAAGGGAAAAAGTTTCATCACTAGATGGCGTCATATTAGACTTTTCTCTGTTGTTTTCTCGTAGGCTA

>184003

TGCAGTTAGCCCGCCATCCGTCATGACGGGGACGGCGTTTAGGCAGTAGGTAACTTGAATAAAAGCCGGTGATCGCTCTTCACCTGTCGGCAGTAA

>172400

TGCAGTGGTGTGAGCACTGGAGGCCGTCATGGAGCAGAGCTGAACCGAGAGGTGATGGATGCAGGTGGGTGGAAGGAGTTTCCTCCTCCAGGTGTC

>27195

TGCAGCTTCGTCCTTCTCCTGCCCTCCTCTTCCTCTCTTTAGCTCTCTTTCCTCGCCCCTCCTCACCCTCGTTAGCTCTCTTTCGTCACTCTCTCT

>149091

TGCAGTAAGCCTCCTCTAATCTCAGCGCTCCTTCCGCCACGTGCATTTTAGGAACTGAGAAATCCATCCAAATATTCAGCTCAGATCCAGATATCC

>230444

TGCAGCGTATATGTCACAAGCTTTGCTATTTGCTGGTGTGAAGCCGACGAATCCAAAAAAAAGGATTTGACCGAGCGCGACAGTGAAACACGCCAT

>41499

TGCAGCTCTTTGGGGAGTGAAATGTAAAATGGAGGAGAGGAGGGTGATCAGGACAGAGACAGACAAATGGTCCGAAGCTTTTAGGTTTGGGACAGA

>36238

TGCAGTGAACAGTGCAATGGGAGCTCTGGGAGTGGTTCACCTGCCCTGCTGCACGGACAAAATAAACACAAAAACAACGGAAAACATGAGATCAGT

>86410

TGCAGTTATGAATGGTAGAAAAGGGATAAGGATGTGACAGGTTGGCAACCTAATTGCTTATGTGTTGACACGGAATTCTGAATTGTGTTGGATTTG

>274972

TGCAGTAGCCGAGGGCTGAATGTTTGTCATTCCGAGTCTCCAGAGAGTTTGTGCAGCACGGTGACAAACATGCCATTCATCAGAGTTGACGTATGC

>270918

TGCAGCGGTCCAGTAACCGGCGGCTCGCCCTCCAGGCTCTGATCATTCCCAGAACCGGATCATGAATTATGGAGGAGCTCTTGTGACTCCGTCTCT

>326356

TGCAGCTCAATGGAAAGAAAAGGTAATTAGACGGTTTGACTCAATCTTCCTCCATCAATTTATATTTGTAGAGCTGAAATGTCTGATTGTAGCTGT

>200574

TGCAGTACTTTCCTGTGCAAACACTTGCCTTTACCCTGTGTCCTTTCTGAATGTCAGCGGGGGCTGCTGCGGACCACGTGTCCCTCCTTCTTGCTG

>55970

TGCAGGTGGAGGTGAGGCGCTCCGCTGCGCTCCTTCCTCCTCTTCATCTCGTGTTCTGGGACAAAAGGCTGAGCTGTAATCTGTGATAATCCAGCA

>100578

TGCAGTCGCCACAGCTCTGGATTTTCACCATGAGGATGAAGGTGGCTCTGGTTTTCCTGCTGTTTCTTCTCTTCTCCTCTGTTTCCATGAATCAGA

>6050

TGCAGGACGAAACGACAAGTCAGACAGACGGGACTTGGAAGAGAACGCAGACGAAGATTTCTCATTCGTGACTCAGATGTTGCCAGCATCTGCGAA

>96407

TGCAGGCTGTGCAGCAGGTAGGAGCGCTGACTGCACCTGAAGGCTCCATGAGGCAGCCTGATGAAGGCACACTGTGGGTGTACGGCAGCTGTAAGC

>312053

TGCAGTCGGACAGCATCCCTCTCATCATGGCCCAACAGTGAGAAAATCTCCTGCTAACTGCTATTGTTTCTGAGCCGCAACAGTCATCCTAATTTC

>159488

TGCAGAGTCCATATTATGTATTACCTCTTTCATTTTTCATTTGCCTCAAGAGTGTCGGTTTTGATTTGAAATGCCACTTGAGTGTGCCCACTCGAG

>217716

TGCAGTCTGAGTTTTCATATTGGTTTTTCCTTGCTGAATGGCCTTTCTGCCCATGATTATTTGGAATTATCTCTGTTATCAGAAAGCATCTTCACA

>157721

TGCAGGAAGGAGTTCACATTTTGATCTCTGTTCCTCCTCCTGTGATGACTCCTCTGAAGTGGGGATTCAAGCGCACAGCACCAAAGACGGAACAGA

>48813

TGCAGGTGAAGTGATGGTAGTTAGTGATTCTGAAGCTATTGGAGGAAATGATAGTAGTGTTCAGGGTAGAGGGACCATGTCTGCCTGTGGGGACCA

>145809

TGCAGGGAGCAGTCGGACCAGGCCGGTGTCGCTTCGCTCTGATTGGTGACAATAAAACACTTGGGACACGTTTCAGTCACATCCAGAGGATGAAGC

>30562

TGCAGGAGCGACTACCAGAGGTAGGAGAGCCCCGCTGCTTGGCTGTAGGGGCCAAGGTCCGGGGCTCGGCCTGCGGTGCGGGAAGCTGCGGGGCCG

>225443

TGCAGGCTGATCGGGTTCCTAGTCCATATCCATCTGCATTTGGAGTCGCACCTCTTTTTACACCAGCTTCTCTTTACTTCAGTCCTGCTGTCTCAG

>24963

TGCAGCTCCAACCACAACAACAGGAGCTACAACCACAACAACTGAAGCAACAACAACAACCACAGCAGCTCCAACCACAACAACTGAAGTACCAAC

>145935

TGCAGCTGTCGTTTCAGACTTTCGTTTTCTCTCTGTGTTCTGAAGGTTCTTTCTTGGTACTCGCACACCGTCTCTTTCACCAGCACCAGAACCTCC

>230700

TGCAGAAAGTTTTCAAAGGAGCAGCCAAATTGGGGCCACTGATTTATTTAGGCAATAACAATAAGCACCTCAAAGCAAACAAAACAAACAAAAAAA

>85214

TGCAGGAGGCCCACGCCCCCCACCGAGACCACACCCAGGACGAGTACCTGGACTCCATCAAGGAGCTGACCCAGCCGGCCTGCTACCCGCTGCACG

>4142

TGCAGGGTCGTTGTTTGTTGATGATGTCGTTGTGTGTCTGCGTTTGTTGATGATGTCGTTGTGTGTTTGCGTTTGTTGATGATGTCGTTGTGTGTC

>265469

TGCAGTATGTTGAGCCCAGCTGTTTATTGAAGAAACAGAGTTATATTTCTAATATCTCTGATTTATGAGACATCCATGCAGCTACATCTAACAGGA

>10783

TGCAGTTTCTCCACACTGAGCCCAGCAGCATGGGTCAGAACCAGAACCGGGTCCAGACTCTTTCTGGGTCAGACCCATCAGAACCGCTGACCGTCT

>280934

TGCAGCAGAGGCTGCATATCCGCTTCATCCAGTTTCTGCCTTCCTCAGAGGAAACAGACAAACAAAAAGCTCTTTTTATGTCCTCTGAGCCGGTTT

>70003

TGCAGCAGGTGGATTAGCAGCCGTGACCTTTGACCCACTGCGTGTCGAGGCAACATGGATCCTGAAAAGCTCCACGGTCACGGCTGAGGAAGAAAA

>92789

TGCAGCCGAAGCGCAAAATCTGCCAAACTGAGCGCCACAAGGCGACACTGCGGCGTAATTCAACCTGGACAGAAAATCCAGCAGCAAATCAAATTA

>281453

TGCAGGTTTCACCTGGATGCCACATTACAGCAACACTTCCACGGCTTCCACACCGACTGCAACGTGTTTTAGGCAAATATATTTGCCCTGCCAAGA

>288597

TGCAGCTCAGGGTGTGTCGTGACCAGCATCTGGACTGGAGATGGAAGATGGAGGAAATGGGAGCGGAAGAGAGGAGGGCATGAATGAAGAATGCAA

>21406

TGCAGGTGAGAGTGAAGGAGGGAGGGGCAGAGGACGGGTAATCTCCAGGAAGTTCAAAGGTCAGAAGCAGTGGCGGAAGGAAAGAGACGTCATACT

>47576

TGCAGCTTCAGCATTTGCTTCCTCAAATCTTCAATAGCACCCGAGTCTGACAGATGGCCACATGAGCATGCACTGGTGGTATGAGCTTGTACATTA

>287349

TGCAGTGACTGGAGCCGAGTACGAGGCCATGCTGACAGAGATCATGTCTATGGGCTACGAGAGGGAGCGAGTGGTCGCTGCACTACGGGCCAGCTT

>44675

TGCAGAGGTCCAAGAGGTTCTGGTTTATCTCCAGCACCAAGGAGCAGAAACAGAGTCTGGTCTGGTCCAGTTCGGGTCGTGTTCCAGGTACGACTG

>317612

TGCAGAGTATTTTGAGTACTTTGCTCCTGACTTCACGCTGCATCCTGATGTCAGCACCAGGATAGAAAACCAGAACTCCAGACAGGTTAAGATCGG

>259364

TGCAGCTGGTTCCTATCCACAACTGTGCAGGGTACATCAGCAGATGGGCGATGCAGTGGGGTACTAAAGTCCTCCGGGAGAGACACAGATGAGAGG

>8055

TGCAGAGAGACAGAGAACAATGGTTGACCGGAGAGAGATGGAAACAGGTGCAAAATGATGGAAAACGGTGCAAACTTGCAGCAAACAGTAAGAAAT

>198698

TGCAGGAAGGAAGTCATGACTTCTTGGCTCTCAATATTTTCTGGCTGCTCCTGAGCAATCGTAATGGACGTGTAAAGGGGGGAAAATTCTGAAATG

>205952

TGCAGCACACACACACACGCACGCACACGCAAACATTCATACACACACACACACGCACGCACGCACGCATGCACATGCACATACACGTGCACACAC

>204278

TGCAGAGCCCACGTCAGCGGCGAACGTGTTTTATGCGATGATTGTTCAGCTTCGCCGATGTTTCAGTCTCATAAGACGCTGCATCAAACACTAACG

>62618

TGCAGCCTAACCATCAGCCCAGTTCTGGACTTTCTGTCCTCAGGAAAAGCTCAGGTGACCAGAACTGCCTGGAAAAGTGTGGCTCCATGGTCATGC

>70783

TGCAGCTCTACCTAGCACCCTATAATCTGAGCATGGATGAAATGTGAAACATCTTTCTGCGAGGGACCATAGAAGTTCAGACCATTTTTTTCTTTG

>68766

TGCAGAGCTAACAGCAGGAAGATGTGTGAAAGCTGCTTGAAGTGTTTCCTTCTTTGCAGGGCACCATGACCAAAGTGTTGAAGGGAGTCAAAGGAG

>164886

TGCAGCAGGAGGCAAACAGGGAAACTGAGGGAGACGAGTTAGACTCAAAACCGAGTCAGAAGACAAACACTGATGTAAGGGAAATAAATCCAAATG

>29622

TGCAGGCAGCGAGACTCTACAGGATACCTGCGGTCCTTATATGACTTATCATCAAAAGCACAGAAAGGGAATGTGTAACCTCCCGTTGATGCAGCC

>19025

TGCAGATTTCTCTCTTTGTGGTGATTTCACTGTCTGCTGTCTTTCAGACATTTAGTGAAAATGTTGAAAACCTGAGCTTCTAGTTTCTAGATCTGG

>282079

TGCAGAGGTCCAGCCCGGTTTGGTCCCAGCCGGATTCCTCACGCGGAGCCACAGTGACACCTGGTGGCCGGTGGCTGCGCTGCGGATTTTATTTTT

>22030

TGCAGAGCTGGACGGAGAGGCGTTGCATTGTTCTGGGCTCAGGAGGCCCAGCTGGGAGAGCTGCGCATCAGTCAGGCACTCCTGTGGGTAGGGCTC

>304664

TGCAGTCAGAACCTCATAATCTGCCGTGCAGAGATGGATCCATCAGAACCGCCTGGGCTGACCAGAACCAGAACCAGAAGGTCTCAGAGTCTGGAG

>180866

TGCAGTTTGCCTTTTAGCAGCAAACATGGAGGGAAAACGCTCAGAGGAAACATCATGTGAACCAAGTGAAGCTAAAACCTCCACATGACAAGTTCT

>194538

TGCAGTGAGCGCGACGACCTCAGCGGTTCTAGGAACAAGTCAAAGAGCCAAAGTGAGACGCAGGAAGCGGTGAGTCTTTCCTTCTGTCGGCTCCTT

>218008

TGCAGGAACTTTGCTGTCTCTTCTCCCTGCATGCTTCTTTGTTCCTGCTTTTGAGATTGACTGGAGCTTGGCAGCTTTTTTTTTTTTTTTTTTTAC

>292318

TGCAGCCGCTCCAAGCAGTGCAGAGGAAGAGGATGGTGAGGAAGACATGCACTGAACACGGGCTGCTATAAACTGCCCTCCACTCTCTAAAGTTCA

>138770

TGCAGGCTGCGGGTCTCTGAGGTTCCCGTTTCATGATTTAGGTCAAAATATGTGAATTTTGCTTCCAGGATGGCGAGAAGAGCCACTGCTGGAAAC

>214401

TGCAGAGCAGAAAGGCATAAAGCCAAGGATTACAGTAGAGTATCAGAGAAAATGGTCTTCAACATGGGAGCTGAACCCATCACAAGGCTTCTATAC

>139490

TGCAGGTGGGGGAACCTTCCAGTTGATGACGCCAGGCAGTTTGGACATGAAGAAGCTGTGAAGCTGCTAAGAGAATACCAGCAAGACTGGAAACAG

>202794

TGCAGCTGGCCATCATCCGCGAGCGCTCCAGCTGCGTACGGATCTTGCTGGACCACAGCGCCAACATCGATATTCAGAATGGTTTCCTGCTGCGCT

>204428

TGCAGTAAGCAGAGCATCCATCAATGTGTCTGAGGTATACAGAGTTCAACCATCGTAATGATTCCTTTGAAGAAGAAAGTACAAGTCACAGGCGTG

>255098

TGCAGGAGGCAACATGAGGAATTTACATCATTTATATCTATAAATATATTTGTTTCCAACATGCTAACCCGCAGCGAGGCGGCCATCTTGGAGTTC

>297135

TGCAGCTAAATAAATGTGCTGCAACAGCATGAGGTCCCCCAGGCTGCAAACCTGTGGGTTGGTGCATGGAAAAAAAATGGGCAAAATAACTCTAAT

>99070

TGCAGCCTGAACTGGCATGAAGGTCCAGATGGACTGAGAGAGACGGAACCTCCTGCTGCATCTCTGACCAAAGAAACAACTGAGGTGGAAACAAAG

>195090

TGCAGACTACCGTGAAATTGATCAAGCTCCTTGAAAAGATGTTGTAGTATGTCTCAGTGACACGTCGAATGAGGGAAATGAGGATAACAGCTCTGT

>228064

TGCAGAATCCAGACGCCCAGTCCCACAGCCATGGAGGAGGAAACGGCTCCGGCCGGAGGCTCCGCCCCCTGCTGGGAGGCCGCAGAGCGACGGCCG

>174920

TGCAGGGGCCAGAAAGTAACTAAACTCGATATCTTGGAATGTTTCCAGACAAATCTAATATTTCCCAGATTGCAGAGCCTGGATTTCAAAATCTGC

>81322

TGCAGATCTGCACAGTGATACAGAGTGACAGGCTTGAGTGCAGACATATAGATCATTTGGTTTGTGTGTGTGTGCGCGTGGGTGCGCGCGCGCGTG

>130461

TGCAGCTTCAAACTGAGCTGCCGACTGACATCCAGATCCTCTGGCAGAGGCCAGAGCATCCAAAGGGAATTAGGAGACATTTTATTGGCTGCATAT

>65433

TGCAGTTGACAACCATTTACTTGGTACACGTGAGATCGTCCTTGGCTTTGTCAGGTCTTTCTTGATTCGCATGGATTGGCGCTGAGGCGACCGCTG

>190389

TGCAGCCTTACGGCTGTGACATTTTTCAGCAGCATAATGCGTCCTGCCACAAACCAAGATTGGTTTGAGGTGCTGACTTGGCAGCCAAATTCCCAG

>143099

TGCAGACTTCCCAGTAAGGAAGTTGCTTTGAAAGGCTGTGATAGCATCGGCCGTACCCCTTCCTCACCCTTGCTTGGCTTGTCTAATCGGCCCTCC

>327009

TGCAGTTAGCCACAGCTTGCAATTGGACAGGAATAACCAATGTTGCAACATCCATAATGTTCCCCAATGAGCAGTGTGGTTTATCGTACCTGTCCC

>305641

TGCAGGCTGAGCCTCAGGCTGTGGTTTGGTCTTCAGCTGCCGACTCAAACCTGACAAACTGTTTGCTTCTCACTGTGATGTGGAAACGTCTCACTG

>73968

TGCAGCTCTCGTTTTGGTTTTGGGGGCAACAAATCTGTCCTTTCTCTGACTGGTTTTGGGGAAGGTTCAGAAAGGGCTACTGGTTGCACAAAAGGT

>21356

TGCAGGTTGGTGTCAGAGCTCAGGTTCTGGTGTTGCTTTTGACAGTTCCGGCTCTGTGTTCACCTAGCTGTTCTGCTGCCAGGGGGCGCCGCTGAG

>167207

TGCAGGAAAACAGCCCCAAAACATGACCCTGCCAACCATAGGCCTGAAGATCTATGTTCCTGTATGCATTTTCAGGCTTGATCATTTCTGTTTCTT

>243666

TGCAGCGATCGGCAGGAGACCGGGAGACAAAGAGCGGCCTCGCAAAATCCTCAACTCCATTTCTCATTTAGAGGAAAACCGTGAAGCTAATCATTT

>21714

TGCAGCTCCACAGACGGCTGTCGTCCCCCAACAGGAGGACGCACATCAGCCGAGGACGGAGGACGGGGACGGTGAGCATGTTGTCCCTCTTCAGTC

>8196

TGCAGGTTCAGGTGAAGGACCGGTTTGGGCTCAACGGACAGAACCAGCAGAATGACGCAGCTGCTGGTTTCCTCCAACTGTTCAGTGGATGCTAGG

>165556

TGCAGCTGAGAAACCTTGGGAAACCTCACCAAAGAGCAATTCAAGTCCACTCTGACAGACTTCGCATCTCTCCTGACTGACAGCCCACTCAAAGAG

>244628

TGCAGGCCGGGTTCCAGTCTCACCAGGATTCCCAGTTCAAAGCTGTTCTTCCTCATCAAATACCAGTCAGACTCTTACTGGGACGGAAACGAGCAG

>51913

TGCAGCATCTTGTCGGGGCTGGCCTCGATGCCTGGAGGCATGTTGCTGGGGTCAAAGGCCAGCTGCTCCACCTCTGCGAAGTAGTTGACCGGGTTC

>326581

TGCAGCTTCTCCCTCTCTATCCCCAGACTTCTTCTTCTGTATCTGAAGGTTTTCTCTGAAGAGCTGAAGTGCTTGCACAGGGCATTGTCAGTAAAA

>75357

TGCAGCAACAGCAGAAAAAGAAGTAGACACAGCAGCAGCAGCGGAAAAATCACCAGAGGCACAAGAGGTGCCAACAGTAGAAAAGGGAAAAGCAGC

>46654

TGCAGCAAGATCTGATGTCTCATATTCACCCTTCCAAAAATGCTCATTCTCAGCGTAATTCATGATAATTCCCTCTGTGTTTTATTGTTTACTATC

>188190

TGCAGCTGCATGTGCTGCTTCAGGGGCTCCTGGGGCCTCAGTAAGTCTGACCACAAATAAGACAGAAATGAGCAATATGTCTGTACATAAACACTG

>182086

TGCAGGGACATTATTGATAGTCTATATTTTGATCCTGATTCAGAGGCTCTATGACTGAGTCAGCCTGCTCTGACTCAGTCATAAGTGGATCTCCCC

>195339

TGCAGCTCATGTGCGACTCACACATGTGTGAGTGATTGCGTGTTTCTAAAAACCCAGAGATTTATATGTTGTTCTCAATTTAGCCTGAGTGCCGCC

>76117

TGCAGCAATGCAAACCTTTTACACATTTTTGAGAATTCCTGGAAGTTTTGGGAACATAAACTCTTTCTCTTGCAGGCTGTGATGTTTTGGCCGTAC

>227828

TGCAGCATCTTGTTGGGTGAATAAAAGAGCAGCACATGAAAGAGGCATCAGCCCAGACAAACCAGATGACTCAATTGATAGGGGAAGTTGCTGATG

>54892

TGCAGAATATGACAGGGCTTATATATGTGTTGTGTTTGTATCAGCTGGTGTTGCTACAACTTTCTCCTATGTTAGTGTCATTATAGCGGCCAGGTC

>79395

TGCAGCACTCAGACACACTCTGCGCGCTACATCCCAGCTGAGCAAGCAGCAGTATCAACATGAACGCCAGAGTCTTCTGTTCGGGGAAGAAAAGTA

>185168

TGCAGCTGAATACTTTATACATGTTTGAGGTTCTGTTTGGTTTTGGTTTGCACATCTGGACTTGGTGAATTTGCTCATTTCTTTGTGTCTTTGTGT

>281623

TGCAGAGTGCTCCTACCAGGATCTGTCAGAACCACCCCACAGAGACCCCGGCTGCTCTCGTTGTCACCTTTTTAAGATCGGAAGAGCGGTTCAGCA

>318778

TGCAGTATTGATGGCTCTGCTGACTCACAGCCAGAGCTGAACACCTGAAGGAGGTCATGTGACATCTCTGAGGTCATGTGACTGTCTGGTTTTACC

>253157

TGCAGGAAGAGAATCCTGTTTGTGCTGCTCGGGATGAGAGTTTGGGAGACTCACATGAGGAAACGATCTTCCTGCTGGAGGTTGGAAACATTTCCT

>130385

TGCAGTGTTCTGCATGCCGGTGTCGCAGCCTACACAGCAGGATGAGCAGTTTGCAGAGGTATGTGCACTTATAACATGAATTGAACGTTCTGTGTA

>134378

TGCAGGTGGAGGATTTCTGCTCTGCTGTTATTTGGCTCTGAGAAACAGGAACGCTGAGGTTTTGTTTGAGGTAGATTTTGGAAAAGTGAATTTCCT

>98743

TGCAGTTGCCTCATCTTCCTGTAGCACACCGGGATGGTGGACACCTTGTTGCACGAGAAGTCGAACTTCACCAGCGGGAGGTCGGCCAGGTCTGAG

>159834

TGCAGTTGCCATCTCTCTCAGCTGTCTTGTCTCTTCCCTCTCAAAGGAAAGGCATGACGGCAACAGCAATGGGAACCCCCGGCTGCCTCACCTGCC

>290049

TGCAGGAAGTGAAGGCAAATCAGTAGCAGTCTGTCACTCCACCACACATCCTCTGTTTGTTTGAGCTGCTGCTTATCTGGTATTCTCTCTATCCGT

>216699

TGCAGACATGCTAGCTACTCAGTCCATCTGAACGTCAAGGTGACATCATGCTGCCACCGCCGTGCCTCGTTTCAAACAACCTGCTAAAAAAAAGAG

>31768

TGCAGCTCTTTCATGGTCATTTGGAACCTCTTGACCTCTTGTTTTTCTTGCCTGGCTTGGAGTCTCAACACTACATGGGTTTGCATTTGTGCATGA

>45369

TGCAGATTTGAAGGACAGACGTATCCCATGCTGTATATCGGGTTGGGTTGTTTCCCACTTCAAGCTACGCTATGACCTGCTTCAAGCTAGGTTCCA

>143903

TGCAGCGTCCGGACCAAAAATGACATTCTGTGTCCTTCATTCTCCCTGTTCACTCTCAGGTTGTGGAAAAACAAGAAGACTGCGTCCTCATGTTGT

>200713

TGCAGCGGCGTTGGATGCCCAGACTGTACCTTCTATACTTCGGTTCTTTTTCTGTACTTCATTCACCATTTGTTCTGGCAGAAAGGAAAACGTTGA

>12310

TGCAGTCTCTTGCTTCATTCTACAGTCTCTTGCTTCATTCTACAGTCTCTTGCTTCATTCTACAGTCTCTTGCTTCATTCTACAGTCTCTTGCATC

>33593

TGCAGTAGCCTGTGATGCCCTTATGACTGCCTGGTGTCTGTTGTTGCTGATGATGTCTGTTGGAAAGAGACAAACGATGTGTGAACGAGTATTTGG

>13708

TGCAGGACGTTCCCCCACCCCACCATCCCTCCACTTACCTCTGTCACCGTCACCGTCCTCCTCAGGAGATCCAGTTAGTTGAGCCAATCTGTTTTT

>119682

TGCAGATGAGCCAGGGCTAATAATCCACACACTAACTTTTACATCCGTTCACTAAGAACAGCCTACAGCTGGTTGGCTATTAGAACCAGATGTTTG

>205193

TGCAGGTTGTCACTTCCTGGCTCCGCCCCCCTCATCCTCAGGTGTGTTGCCATGGTAACAGAGGGGCTCAGGATGACCAGGTGATCCAGAGAGAAC

>52864

TGCAGCGATGAAGGGGGGAGACGTGTGCGGCTCGGGGCAAAGTGTGAGGTTTATCTCAGCCACTCTGAGGAGAACCACAACGATCCAGCCGTCACT

>306032

TGCAGGCTGCATTACACTTATATATTCAAGGTTTATTGGATGCAGTTGTTAGTCTTCTGTCTATTTTGGTGTCATGGGATATATATGTCTGTCTGT

>123253

TGCAGACCTGTGACATCCGGCAGCTTCCCCTTATGCTCCCAACTGGCTGGCCTCTACCTCCTCAACCAAAGGGCTCAGGAATGGAAACTCGCTCTC

>287623

TGCAGAAAGACTTCTGAGAGGTTCGGAAAGAGACTGACGCACAATGGTGTCTGAGCACAGCGAGGAGCAGTTCACTGTTCACTTCTGCTCTCGCTC

>107425

TGCAGAGGCCGTTCCAGCCGTCCAACAACAATACATTTAGGGATGGAAATGTTCTGTTCAGCGTCCTAAAATCTTGATGCTTTTCGCATTGGCGGA

>269263

TGCAGCCGTGCATTCATCACAGTCCTTCCTGTTTCCTTCATTTGGTAATAATTGCCCCAAAACACTCATCGACTGTTGATCACAATATTTGCTATG

>111452

TGCAGTAACGCCACTTACTTCTTTCATTTTTGTCATGCGTTTTCTAGTGTATACAGCTGCACGCTCTTCACAATCCCATGATCACACGGATCAAAT

>44867

TGCAGCTGAGCACCAACACCAAGCACCCCCGACACATCATATATAGCAACAGCACAAGGCAGAGGCTTGCTTGATAGACCACCCTCATATGATTCG

>73517

TGCAGGCTAACGATCTTGCCCGTGAACACATCACAGCTGGAAGAAGAATATTCCACCCACTGAGCCACAGGCGGCGCATAAAACAAAAGTCTAAAA

>219672

TGCAGAGAAATATTTGGGATCGTTTCAAGAGCTTCGAAAGGAGAAAACTGAGGTTGGACTCTCAGGGTGAGCGATTCTGGGAATCGCCAAGTAAAT

>40730

TGCAGCCTCAGACGAGCAATCGCTTTTACTTTGACTAGACCAGATGCAGTCCCTCAGACTCTAAAGGAAGCTAGCAGAGCGACTCAGCAGGCCGAG

>322924

TGCAGGGGTGTACTTACTTACTTAGTTTTCCCATACCTGTTTTGACCGTCATGGTTTTTCCCGGCTGCTGTATGGATGCATTATGAATTCTGAATA

>193504

TGCAGTGGCGGCCCCATCTGCTGGGGTTCCCTGTTGCAGGCAGAGGCCAGCCAAGCTGTCTAATACATGACGGCTTCAGTCTGCTGTGGGTCGGCT

>27086

TGCAGCTGCTGAATATGTTCAGACATAATCAGTCCAGGGATTTCATCTCTGCGTTGTGGAAATAATATCAATGAGAGGACCGCTCTGGTTGCCTTG

>335055

TGCAGTCCAGACATGATGCTCATCCAGGTGCTGTGGATCTTCCAGGACCTGGAAAGAAGAGGAAACCGGGTTCTGACCTGGAGACGTCCGGTTCTG

>196664

TGCAGTGGGATTACTCCACCTTTAGGTGGAGCGAAATGCAGCCAGTGAGTGCCCCACACTTACTCCTGTAGATGTAATGGCACAAACACCCAGGAG

>342822

TGCAGCATCCAATGGGAATGATGTCCCCATGCCGAGACAGGGCAGACGCTGATGGATGGCCTAGCTTTCTGCCAGTTTCTAGTCCCACGTCGGAGC

>32272

TGCAGACTGCTGACTCAGGCGGCCAGATAGAGTCCCAGAGTGAATGGAGGGTGTGGGAGTGCGTGCGTGGGGTGGAATGCCAACAAAGTGGTGATT

>23347

TGCAGAACAGGGAGGGGAGTGAAGGTCAGTGGCCGGTTCATGTGACGCAACACCCAACAGGACGGGACGACGCAGAACCCATGCCTGAGGCGAGAG

>97130

TGCAGCTTGTTGAAGTATGATGGTCTTGTGCTGCCATCTAGTGGAAACAGTTATGGTTGCACAAAGTGTTCCATTTCACAGAAATGAGGTTGATAT

>85432

TGCAGTGCTTAGGGCTGAGGCAGGTTTTTATGCAAGGTATCATTCTGAAAGTCAAATAAGCGTAACATCTACAAGAACAACAGCATAGCATGAAAG

>215206

TGCAGAACACGTGAGCGCCGACTACATGAAGCACTTCCTGTCACCTTCCAGAGTAAAAGCCGCTCTGACCTCCTGCCCAGGTTATCGTGGAAGACG

>210401

TGCAGAAATCTCCCCCACTCTTGTTCGTCTGTGAGGGATTCGTGCTCCTTGGCTGCTCACACCGCAGCGCATTTACCTTTGAAATTTGGTCTGATG

>29119

TGCAGAGACACAACCCACAGACACGGTCAGACAGACAGACAGACACACAGATGGACAAACAGACGCAACGAAACACAGAGAAGGACAGACCTCGTT

>9165

TGCAGTTCCCCCATCCTGCTCAGCTCTTCTGCACATATGACCAAGTGTGTACTTCAATACGTTTGACCCCTGATTATTGTAATGTTTTTTGTATAC

>160229

TGCAGCGGAGGAATACCTGATGAGGAGGACATGTTTATATGGATGAGGGCCGCTGCCACATTTTATATCGCCGTGAAGCGCCGCAGCAGATGGTAA

>284359

TGCAGCCAATCTCCATCCTCAGCATGGCTTTCACCCCTGTCGTTCTCCCCTCTCTTTGAGCTGTGATATGATTGGCTGTGGCATGGGTGAGGCTCA

>27677

TGCAGACAATTTTGTCTCGAATAAAGGTGAAAAATAGCTGACAGTGAAACTGCTCGGGTATGTTCAGCTTGTGTCTGCCGATGCTAAATAACTGGA

>313460

TGCAGGGCAGCGTCAGAGCTCTGCTGCACGAGAAAATCAGAGACGCTTACACGCACCCGCAGTTCATCACCGACGTCATGAAGCCGCTGCAGCA

>214044

TGCAGCAGCTCAGACTGGGTTTCCTGAGAGTCGAGTCCAGGTTCTTCTGCGATGCAGAACCGAAGAGTCGACCTGAGAAAAACTGACAAACTGCTT

>137244

TGCAGTTCACCAGCAGGAGGCGCTGTGGCACTGACGGAACCAGCAAAGAACACAGACTGGACCCAGAACCAGTGGGACCTCCACCAGAACCAGAAC

>304025

TGCAGCAGTCAGTGTCTCCTCTTTGACCTTTGCCCTCTGGCGTACGGGGCTGAACTGGGCCTGGAACATTTGTTTTCTGCATCCTGTCAGATTAAG

>139482

TGCAGAACAAGCTGCTCCAGTCCACTGATGCAGAAATCCTTCGTTTCAACACTATTGCTCACATGTGCAGATCAATGTACGCTCGGGTTATATTTA

>8090

TGCAGAACGCGGCTTTATTTTTGACCTGGATATTTTTAGCTCGGTATTTGTGCTATTTTTTCCTTTGCGCAGCGCAGAGGCTCATCTTTCTGGGAA

>317034

TGCAGCAGCCGTCCACTGGGACAGAACGAATGTCCTCCAGAGACGTCTGACGGTCACATGGTGGTGGGATCTGGAGGAGTCCAGGTTGTGAGGCA

>98165

TGCAGCTATGAGTACTGGAATGAAGATGTGTTCTCTTTGACTCGGTTTCACAGGCACAGAGAGCTTACTCTCACGTGTGCCTAGCTCATCTGTAGA

>269092

TGCAGCTTGAACTTTTGAACTTCAACTTTTTTATTTCATTCTCATTCAGTCTGAATGAGCCCCAAATTGTTTACCTCTCCTTCACCAGATGTGATC

>1754

TGCAGGACACCTTGAACACATCTATGCCGTGTTTTTACAGGGCTTACAGTGACAGCATGTGCCGCTCGATGGATGGAAAATGTGGTCTTATTTCTC

>71333

TGCAGTGGTGGTCACTCTGGAGCCGCTGGTGGGAGCGGAGACGTACGTGAACGGGAAGCAGATCACCGAGGCGGTCGTCCTGAAACAAGGTCAGAC

>112554

TGCAGGTGGTTCTGATGTTTCTAAACAAGAAATTCGTTTCAGACAAAAGGGAGTGGGCGGGGCTTGTTGGTTCAGGTGATCCATCAGTGCTACCTC

>77964

TGCAGGAGGTAAAAACCCTCTGATCTTTGACCTTTGGCCTGAAACGTGAGCTAACCTTTCAGCTTCCCTGCTCCAGATCTTCGACAGACAGACGGT

>57964

TGCAGCAACCGGGGTGTTGCCTCGTTCAGGTGACGTCAGCTTGGATGAAGCAGCCAGCCGTTACCACCTGCCAACCAGGCCAGCCAGAGGTGAAGA

>69296

TGCAGAGTTCAGCTGCCCGGACCCTTCAACGACCCGACGTCCATAGTCCACCTCATCATCATCCGGCGTACGCTCGCTCCTCCACCTCTGACTCTG

>279898

TGCAGAGTTCTGCTCCGCTTCCCAGAGAGGACCAAGATGCATGAGAGAACACATGGCAGCATCATGCCGTGGGACAGGTGGGTGGGGCTGAATGAG

>224527

TGCAGGGGGAGGCTGGGGTGCAGCACCAGGTGGCTATGGCGCGGTAATTGAATCCTTATTTCTGCATCTCTGAAGTGGAATGTCTTAAGATCGGAA

>307646

TGCAGGAGTCATGTAAGACAGACATGCCAATGTTCTGGGAGGGCAGCGGGAATACAATGAGCTGTAAAGAGTTCTCCAGCTACAGAAAATATCGAC

>71427

TGCAGGTAGGTGGCGATGGCGCACACCGACAGGTCCGTCACGTTGACGCAGGACGTCAGGTCCAGCTCCAGCAGGGAGTCCGCCAGGAGCTGCGAG

>58276

TGCAGCTTTTCTGGTCATTATTTCCGTGCTGCCTTTAGTTTCTGAGTTTGGGTTAGCCGCTTCCTCCTCTCTGCTCGGTACGTTACGAGCTCATGA

>44880

TGCAGCAGCTTGACGAGCCGTGAGACGAGAGCAGAGGTCCAGAAAGGAAACTTAGCTTCTGTTTGTGTTCACATGGTGGGATCCAGAACCGGGTCT

>166322

TGCAGCGACTCGGCGGCCTCTGAGCAGGTACGTCCTGGAGGAGGATCTGGACAAATAGCGAGCCTGAGGGGTACCGTCGGCAGGACCAATGCTTTA

>186750

TGCAGGCTACTGGAAGACCAGTCAAATACGTCTCTATGTGCAAAAAAAAAAGATTTGCATTTCAGAAAATGGATAGAAAACGCACAAAAAAACCCC

>161944

TGCAGGTGTAGACGTTGGTTTCTAGCTGAAACCTAAATGTTCACAGATAAGTGATGTTTTTCTGAGCGTCGCCATGGAAACGGGGTTCGATGGGAT

>123305

TGCAGGTAAAGCTTCGCTTGCACTACTGCAAGGATAAAAAACCAGCAGTAAGTAGGAGAAACGTTAGATTTCACCGGGGAACGCTTGTTCAACGCT

>123434

TGCAGTGATGACGGTCGGTTACGTCAACAAGCATCACCACGGTTACATCACTACAGATCTCTATCATTTACATCGCGCTCTCCTTTGTAACAGTAT

>262014

TGCAGGATGGACGAGGCTCTATGGTTCTTGTTATCTTGTGTATGGATGCGGTTCCTGGGATGCAGCCAGAGAATACTGCGTGAAGAAAAACGCCCA

>342644

TGCAGTTTGACCTGTGACCCGACCGCTGGTCATCCTGTGTCCAGAACAGAAGCAGGTGGGGGAGGGACGCTTCTGGATGAGGAGAACCGCCGTAAA

>228636

TGCAGTGTGTGTGTGTCGCTGTGTGTTGCTGCAATGTGTTGTGATTGTTCTCTTCCTGTTTCCTACCGTCTGCTGTGTGAATCTCCCTCAGCACCA

>280696

TGCAGAACCAGAATTGGGTCTAGACTCAGAACCAAACAGTGACACAGTGCAACCAAGCAGACCGGTTCTGACCCAGCCAGCAGGAAACAGCTGCAG

>107192

TGCAGAGCTGTGATGATGATGGAGGCGGTAAAGACGGTCATTAGCATCATCGCTGTAATCCAACTCGCTGTTATGGACGTTACTGTCAGATGAATA

>262584

TGCAGACTCACCAAGTAAAGAATGTGCAAGTCTTTACTGGTAATGCAGACAGTAAGATGCTGGTGGAAGACTGGATCCGTGATATGCAATACCTGT

>27665

TGCAGGTGTATGATATTGTCTTCAACTCCACAACATTCTTGCTGCAAGGTAACAGGGCTGCAACTTCTCCGTCAAACTCCTGACTGAAGTTGGCTC

>18736

TGCAGAATACAGACACTAAGGTAAGGCACCATCATTGGTTACTGTCTTTGGCTCAGGAGTTTGGGCTTCATCCCGTGACAGGTGGGTTACCAGTTC

>125376

TGCAGTGAGAGCTGTTTCCATCCCCGCAGCGCCTCGCTGGGCTGCTGCCACCGCAGAGCCCCGCTGCTCTCTGAAAACGCCACCTGGATGTGGTTT

>2404

TGCAGGCGTCCGACTTGGCTGCTGGTCTCATTACAGGAAATGAATCTCTCTGTCCGGCCGGATCATAAATCCATAAGAAAGCAGCAAACAGGCTGA

>220196

TGCAGACGCCTCTCAGGTGACTGATGTTGTAAAACACAGCGCTGCGTCTCCTGTTGACGGACTCCTCGCTAAACAACATTTATCTCCCCAGGAACC

>125469

TGCAGCAGCAGTTGGTTGAGAAGAAACCGTTTAGTCGCTCGGGAAGAAGATCCGTGCAACATAAATCCCGACTGCGGCGGCAGAGTCCTGCTGGAA

>109797

TGCAGGTTTCCCAGGTCAAATCCCTTTCCTGTCTCCTCAGGTTTACAGTAACTTGCGGTATGCCGTGTCCACGATGTGGCGCTCTGAGGGACCTGT

>86215

TGCAGCTTGGAGCGAAACACGGAGCTAAAGCGGACAGTAAAACCTCAGTCTGTCACATTTACATCACAGAAATGCCCTGAGTTCAGAGGATCAGGA

>299167

TGCAGCCATGTTGTCCGGTCTCCATGGAAACACAGAGAACGTGATGATCTTTGACCTTTAGACAGATTCTGTGGGAGAAAGGCAAGAAGAAAGGTT

>286773

TGCAGGCTTCACAGACACACACTCCATCCCTGCATCCTTTCCTCTGCCAGTCCAGTTCAGTTCCTCCTCCTTCTCAAACGGTTTGTCTTCTTTTGA

>178520

TGCAGGTTTCCTTGCAGTGGGTGAAAAGTTCATCAGAAGTTGCTTTCCTGCTAAAGACGAACTCTGCAAGTCGTTTTGACTCACCAGGTCAAACAC

>22615

TGCAGAGGAAGAGGAGGCGAAAGAGGAAGAGGATGAAGAAGAGAAAGAAGGAGACAAGGAGGAGAAAGGAAAAGAGGAGGAGGAGATGAGGAGAAG

>5542

TGCAGACCGGCTAAATATATGAATGAACTGAAGGTCTGTGAATGGAAAGCAGGCATCTGGAAGGTTCCTCTAAGCAGCTGAGCCTAAATAATCCAA

>243361

TGCAGTTCAGAACCTCAAAGCGTCGAATCTGTGTTGAGTTTGCGGGTTTGAGCCACCAGAGGGCGCCACTCAGGATTTATTCTGCATTCACGTCAC

>219262

TGCAGCCGCGCAGCTACCTCACAGCATCAGGGTTCACTGCAAACTCTCCGCTCTCATTGAGTCATTTGATATAAAAGCTCTATGTGATTTGATCAA

>211035

TGCAGATGTTTCAGCGCCTGACCAGGTGGGGGCGCCGTGTCCTTCGTCACCCCCGGCAGGATGCCTCAGACCAGGAGGCGACTCACTACGCTAGCG

>215108

TGCAGACTCGTCTTTCCTTTCCTTTCTGACTTGAAGCGCTTTGTTCTGCTGACATGAACGTTTTCTCGCCCTGCTTGCGTCTTTTCTTTCGTCGCG

>180385

TGCAGCAGTGGGTCAAAGCTCAGCCTTTCACAGGGAACAACGAGCAATGAATGGGTCAAGTACAGTAAATTGCACAAAATGCAGGGTTTCATCTGG

>162567

TGCAGTCCTGTTGTGTGCAGTCGGTGGGTGTCTTTACTCTGCGCTGTGCAACATCCACTCGACAATGAGTGCAGACTTTATTTATGCTATGAGGAA

>39618

TGCAGCGTCTAAACGAAAGCCGTGAAGCGGAGGTGAAAACATGTCGAATGCTAAGCTAAATCAGGCCCAGGCTAATGAATAAACCACAAAGAGCCA

>184603

TGCAGTGCGGCTCCCTCGCCCCGTCCACCCCCCTGACAGGACGCGTCTACCTGAAGGAGAAGGACCTGCTGTTCACGCCCGTCTCCGCGGCGACGC

>67403

TGCAGATCCAGTAGAACTGACAGAACTAACACAAAACACTGGAGCTGCCTGTTGTGTATTTCATCGTTCCTGTAAGCCTGAACATGAAATGAGCAG

>28205

TGCAGTGGCCAGCTTACCAATATCACTCCACGATCACACCAACGACCCATATAGGAGGAGAACCCAGGACAGCATCAGTTTGTATCTTTTAGATCA

>128769

TGCAGGGAAATAGCTCACGAGTGAGCATCTGTCACAAAGATATGTTGACCTGACAGACTCCCACTCCAAGCGAAGGAAAACCTGGGCTAGTTTCAG

>11923

TGCAGGTTCAGTGTTATCATATTTCTGGTCTCCCTGCTGAGCTGATCTCAGCTGTTATTACTCTGAGGTTTGTTGCTGCTCTGCTTCTCTTCCCAT

>10474

TGCAGTCCGTCCTGCCGCTCTGCCCACATCAGACAGGCAACGGCAGGCTGGGCAGAGCCAGCGGGAAGCTAGCAGGGCGTGCTCTGACTCAGCAGC

>29592

TGCAGCCTCCGAACAAGTGCTCTGCTTTATGTCCCGCCTATCCGCGGTGATGAGCCCCACCGCACAACACCTCGACACTTCCGAGTTTCATCCGCT

>24875

TGCAGCCAAACGGCGTTTCTGGTTGATACATTTGCTCCGTGTGTGTTCGCCTTCTTTCCTTTCTTCCTGGACAGGAATCGCACATCGTGCACGACT

>161551

TGCAGTGTTTTATGCAGGATGTTTTCTAATGATCTGAACACATGGGGAATGACGAATGACTGAGAAAATCGAAGTTTTTCCCGTTCTTGGGGTCGA

>216887

TGCAGCCGGAGTGCAGCGGAGCAGACCGGGAGGCGGAATGGCCTGAGCTCGGCTCGACTCGGATCGCCTTGACGGGGCGAGATGCTGCTCCTCTAA

>207642

TGCAGAAGAAAATTGGGAGCAAAAAATGTGGAGAGGCCAATGCAAGGAGAAGAAGCCTTTGGAAGAAGCCAAAGTCCCCGACGGTCTCGAGAATTT

>214762

TGCAGGGGGTGGAGGTGGTGCAGGTGGGACTGCACCACCAGAGGGAGGGGGAGGAGGAGGTGGAGGTGCTGGTGGGGTTGGAGCTGGAGGAGGTGG

>246830

TGCAGGAAAAGCTGCTGCTATTGAAAGCTAGAGGCTGCCTTTTATATTAGAAAATGATTGGCAAAGGAGACAGACTGCTGGCTATTTTGGAGAAAA

>50231

TGCAGCTCTGGCCCTGACTGGGTGTGGACAGAGCATCACAGTTAGCGCACAACGTTCACCACATTCACTGAAACACCAATCCATGCATCTTGTCTT

>300757

TGCAGCACCGAGGCACTGACTCAGGCCAGCTGAACTCACAAACCAGTAGACGCTTTTCAGAGTTTCACAAATATGAATTTAAGATCGGAAGAGCGG

>103401

TGCAGGTAAGAGCCAGGCTGCTCTTACCTACGTGTTGCCGTGGAAACTGCATGTTTTCGACGTGTTTCAGCTGAGCGCCTTGGATGGGAGACGGGC

>97624

TGCAGAATGAAAACTCCCTCCCACCTCCGCGGTGTTCACCCAGCATGCAGAATGAAAATCATAGCTCCCCAAGTCAACCAAACAGGTGAGGTCAGA

>126503

TGCAGAACAACAACTCCAAACAAACAGCCAGAGAGAGATGAGTGGCCTAGTCAAAGCCCAGCCCTGAGCCTATTTGTGGCAGGGCTGAACTGATGT

>161823

TGCAGAACCAGAACATCTGGAATGTGGAGTAACAAGCTTCCATTCAGAACCAACAGGACATTCTGGGGTTCAGGTTCTACAGGACAAACCGGGTTT

>231628

TGCAGGAGCAGAGAGCAGCAGCAGCAGGTCAGATCCCGTCCTGATTTGGATCTAACTTCACCGAGCACCGACTGGACAGCCGTGCCCTTTCACCGC

>268718

TGCAGCAGCTCCTCCTCTTCCTCCTTCCTCCTCTTCTCATCTGCCTTATTCCTCTTTATTTCTTCTTCTGCCTCGTCTTTCTTCTTCCTCTGCTC

>229766

TGCAGGATTCAACTCCAACCACAAATGCTAAGCTACCGACCCAAACTGACTCCTGAGTCACATTCAGGTGTGACTGAACACACACTTTCCCTGGTG

>296439

TGCAGCATCCAAAGTGGGATTGATTAGGAAGACGGGAGGAACACGACCCGGTTCGGCCCAGGGCTTCACTCACTACTACGCCCAAGCAGACAGAAC

>124432

TGCAGGATCTGGTCGGCACGTCGCTCACAGACTTCTCTGCTGCTCAGCGGGCGGAAAAAGGCCTGACAGGCTAAAACCACCGGCTCTGCTCCTCTG

>331822

TGCAGCTGGCTGGGTCAAGCAGTCCATCTGTAAATCAGCAAATGATTTGAAGAACATCACTGGTACATAGTACCTCTGAAGATAACAACTGTTGGA

>9450

TGCAGTTGTGAAGTGTGTTGAGATGGAGGCAGTGGAAGACAGTGAGCAAATGTTACCCTTACGCTGTGCTCATAAAGAGAAGACTGCTTCGAACTA

>211029

TGCAGGAGGAAACGTTTCAACGCAGCTGTGAGGCGTTCAGGTGCTCATGACAGACTGTGGGAAACTCCAGCATTTAAGATCGGAAGAGCGGTTCAG

>155683

TGCAGAAACCCAACTCCACCCCCCTCCAGCAGCAGCATCCATAACAACCGGCCCTGGAAACAGCCCAGCTCGGCCTGCCATCCCCGCTAATTCGGG

>137764

TGCAGGATGTTGGCGGCATGAGAAGCAGACCACAGCATTTAGAAGCCTTGGGGTCAGGGGTCATGTATTGAGCTCATCAGAAAACCCACTTCCTGT

>12025

TGCAGGGTTCACGTGAGGGAAAATTGATAGTGAATATCACTGCGCCACAACGCGAATGCCAGCCTCCCCACGTCTGAATCGTTGCATCACAAAGCT

>142674

TGCAGGCGCTGAAGCTGGAGGAAAACGACAAGCTGACCTTCGTTTATGAGACGGTTTATAACTCTGGACCTGTGACCATCGTGAAGAAGGACAAGA

>27800

TGCAGCAGGCTGTCCAGGTCATCCTGAGCCTGGAGAAGCAGGTGCGAGGTAGGTCAGCCGAGTGCAAGCAGGTGCGATCCACTTTAGACAGCTGGA

>262979

TGCAGCCCCAGCCTCCGCTGCCCGGATCCGCGCCCAGCCTCCGGGCATGCCGCCCCGCCGCGCCGCTGATCGGGACGCCGGGCAGCAGCCGCCGCC

>175843

TGCAGTGTGGACGCAGCCTATGAGAGCTGATCTCCAGCTGGATGTTTAGTGTTCTGTGGGCTCCTGACCCACAGATGGGTGCTTGCACAGAAAGTA

>163190

TGCAGGTTCTGGAAATGAAAGTGAACATTTCCCCTGTTGATGGATGGAAAACACTCTGAACCATGTGAGGCTCGTTCTGGTGGGAACAGCAAAATG

>337134

TGCAGGCTATAGATGGATCTGGTCCACTCTTGTGTCAGCTATCTTTTAGTTTCGACTAGCCTCACTATTTACTTATTCATGGGAGTAAAAATGGAG

>32197

TGCAGCGGCGCTGACGCACAATAAACACTCTGATAACGGAGCAATCTGCAACTGTGGTGGAAACAAACAACACGGCGGCCCTTACAACACTTCTTT

>189018

TGCAGCAAATGAACGTGACTGAGGACCATAGAAACGGGGAGTCCACGGGGGAGGCTCTGGCCGGCTAGGGTAGGGGAACTGATTTCTCCTTCTAGA

>47842

TGCAGCAAAAAGTCAAATCCAGAGTGTCACATCGGGCTGAAACGCCACCAGCTGTCATGGCAGTGCACACTCCAAATAGACGTTCTGATGCGGATG

>16983

TGCAGTGAACTCTGGTTTGCTTCGTTTGTTCTGTCGTTTAGGACTCCTCACCTCGGCTGCTGTGTCCTTTGAACTCTGCTGCTCTTCCACAGTCCT

>264656

TGCAGCAGGTGGGCCTTTTGATTTGTTACCCAGGAAGAACCGAGTCTCATTTGCATACAGATGCCCCCTTCAGCGCAGAGGAAGGGTTTAAGATCG

>35150

TGCAGAGGAACTGGCTGCAAATGACGACGATTGTGCCATCTGCTGGGACACCATGCTAACAGCACGCAAACTGCCATGTGGCCATCTTTTCCACAA

>45129

TGCAGTCAAACAAATAAACGACCTTCAGCTTCAGCAGCAAAGAAACGTTCAGAGGAAACAGCAGAAACTGATTCAAACTGGAACAAAGGACAGTTT

>74856

TGCAGATCAGATGAAGATCTCAGCTGATCCTGGACTCTCCGTCACTCTGAGCTGCTGGGATCAGATGGATCCAGGAAACCAGCATCAGGACGGAGA

>290212

TGCAGGGCAGAAGAGTTAGTTCAGCTTACTGACACCGGATCTGACCCCTCTGACAACTTTTTACCTCTGTTCCACGAATGCTACTGTTTTGGACAG

>246498

TGCAGACGGACAGAGAGACGGACAGCTGACTAAATTTCCTCACTTCCCTTTTCCATCCAACCGGGTATTTATAGAACCAGACTGTTGTTACCAAAC

>219406

TGCAGGTAATAGAAAGACAATGTCACCAGATTAGCAGGCAGAGAATGGGCTGCTGTTTCCATGGCAACGGAGCACGAGGGAGGACGGGACGGGGTG

>290500

TGCAGGTAGAAACACACATCCGCCTCACACCCTGTTACTCATCACCGCAACATTTTCTCTCTCTCAGAGGAAATGTTGCAGGTGTAACTGCTAATG

>274311

TGCAGTTTGTTTCCATCCATCCATCCATCCATCCATCCATCCATCCATCCATCCATCCATCCATCCATCCATCCATCCATCCATCCATCCATCCAT

>163918

TGCAGCTCCCGTCCAAAGTTTTCTCCATCTGCAAGGTCCAACAGAAGAAGGTAAGACCCACAGAGGTTCTGGTTCTGGTTCTGGTTCTGGTTCTGG

>218594

TGCAGCACAAAGCGCCCAGTCACCTGAAAGACTGGGAGGAAATGACTAACGACTAATCAGAGCACCTGGCCTGGAAAGACTTGAACACATTAAGAT

>157067

TGCAGCGCCTACCTGGCCGGGAACGTGGTGGCCTGGCTGCCCGGCAGCGTGGAGGTGTTCGGCAGGGGCGCCTCCAAGCGCCCCCTGGAGGGCTGC

>270001

TGCAGAGCGTAGACCTGCTGCCAGCTGGTTTCCATGGATACGCACCAGCTGGTTTCCATGGATACGCACCATGAAAAAGGAACAGAAATCCATCCA

>10532

TGCAGATCAGAGATGGAGGCGGAGCTAAAGCTGCTGTTTCCAGAGCTTTACAGAGAAATCCTGCTCATCGCCACGGAAACCGACTCAACCAGCTTC

>61686

TGCAGAGGAACTTTTCTCTTTTCCCCAGACGGAGTTTCATCCTCTGCTCTCTGGAGAAACTTGAGCAGAAGTGAAAACTAATCGTGCAATAAATCC

>242629

TGCAGAGCTTTACAGTGGCTGAAAGTCTACCTGCGATTATCACCGGTTTCTCATTTTTTATTCATACACATTTCTCTGCTCATTTTTGTATTGAGT

>93634

TGCAGGTCTGTCCTGTACCTGCAACACGTCAATAGTCAACTTCATCGACCACGTGTTCACACAACTCAGTAATTTCATCATCAATGCAGCATTAGT

>267465

TGCAGCACAAACCCATTGACTAGCTGCCACTTCCCCCTCTGATCAACACCTCAGCAGTTGGACGAACCTGCAAGGAGCAGCGCTTCCTTCTCTGCA

>240114

TGCAGCGCCGCAGCAGCCACTCATGCATCTCTAATCCTGCGCCGCCTGGAGCTTTGGGCTGAACGGAGCTGCTGTTCCCCCGGTGGCCTGGAAGCT

>242085

TGCAGCGAAGGCTAGAAAAAGGTCAGACAAGCAGATCCTGTTCAGACATTTCACTTTGTGTTCATATGAAGAATCTTGCTGATGTCCAGAATAAAA

>235325

TGCAGGTCACAGGAACTGTTCACCTGAAGCCGTGAACTCTGACCTCCAGGCTCCTGCCGTCGTCACAGCTCCTCAGCTTCTGCTGCATCTCTGACA

>324768

TGCAGTTTAGAGGTTTGGTTTATAACTTCAAAATTGTCTTCAGGCTCTTCCAAACCCAGCTAATGTCTATCTTCTATCTTCTTTCTGTAGAGATTT

>30977

TGCAGCTGCCGGTGTTTGGACCCGCAGTGATTTATGGGCCTGTGGTGGAGCCTTCAGAGGGTGTAATTAGCCAGTCTCAGTCTGGTCGCGTGGAAC

>138357

TGCAGAAAGTAAGCCAGAGGAAATGAAATGGGATGGTTGGGGAGGGGCTCCTCTGCTTATTGCTGTGAAAAATTGTCTGCGTACATTCTTCTGAAA

>274982

TGCAGAAGAAGTTTCTTATATAATGATGTAAAAACAAAAGGCAGAATTATGTACGACGAGATGAAGTAAGAGCATCACTGTGGCTGGTAAACCCAG

>189714

TGCAGCAAGTTTCCTCATTGAAATCTTACTTTCCAACAAATGCAGCTTGTGTGCACCGATGATGCCTTGAGCTGCAAGGAGCTGTTGTGTGAGCAC

>260197

TGCAGGGTGCTTGTGGTCTCCAACAGCCTGTGTTGGAAAACGGTTGCTTACACATTTTTGCTTTATATGCAAAACTTTTTTTCTTGTCATAGCTCC

>54962

TGCAGGAGGTAAATGTATTTCCCACTTGTTGCTGAGCCTGAGTGTAAAAGTTTGAAGATGTAAAAGTCTGGAAACATAAAGCAGCTTGAAAAGTGA

>97899

TGCAGGCGATCTGAGCATCTCAGCAGACAGACTGAGTGAGAAGAAGTCCCAGAACGACTTCGCTCTGTGGAAAGCCTCGAAGCCCGGCGAGCCGTC

>217415

TGCAGTGTTTCTGTGGACAACGATCGAAGTCGACCACTATCACCGACGGTGAGAGACAAACATAGCTTCAACAGCTGCCTTTGCTTTGCATGCTTT

>55742

TGCAGCCAACAACCAGAACCGCTGGTGCGTGCACGTGTATAAAAACGTGCACGTATAACTGAATATACTTTTATGGGTCAGTGATTCGGTAAGACA

>145461

TGCAGACTGACTACAGCTTTACTGGCCCCCTACATGCTCTGCCCACCATCTCCAAAAGTCTTCTGCCGTCCACCCCGAGCTCCATCACCTCTACAG

>107541

TGCAGGAACCCGACTCGTCGTTGGTTCAGGTCGGTTCAGGTCCAAAATGACCCGATCCAGTTCTGATAACTCCTGGTTCTGGTTCTGGTCCTGGTT

>141513

TGCAGCAGGAGGATTTTATTTCTGCGGATGAGCCAGACAGGAAGTGACGGAGGAATCCCTCCCAGCGGGCTTCGCTCCGAACTAATGACTCAAAAA

>314515

TGCAGCGCGACTCGGGAGCAGCATGTGTGGTTCGACGCCCTCATGATGCTCAGAGGAGGTTGGCTCTGAATCAGCAGCGACGGTTTAAGATCGGAA

>101912

TGCAGCCTAGTTAGCATGTACATGTTTGCATTTGATTAGCTTGTGTATGTCAGAGTGCACAGATCTGTGGAGACAGCGACCCTGTGATTCTCAAAA

>112947

TGCAGGATTTCATCAACTTCTGCCTGAACTACATGGCCAAGATCAAACTGCCCAGGAAAAGGTACCGACCTAACGCGGTTCTGATGAGACATTTAC

>215806

TGCAGAGCGTGTGGCTGCCGTCCCAGATGCTCACTGTATGCTCCTGTCATCAGGAGGGAACTCCAGGAATTCCCATCTAAATCAAACCGCACAACA

>247892

TGCAGGGAAGTGTGTGTGTGTGTGTGTGTGTGTGTGTGTGTGTGTGTGTGTGTGTGTGTGTGTGTGTGCGTGTGTGTGTGTGTGTGTGTGTGTGTG

>216633

TGCAGCAAAAACTGGTAAAACAAAGAGCAGCTCTGAGTCTGACAGCTCAGAGTCATCAGAGGATGAAGCAGCCAAGAAACCAGCCAGCAAACCTGC

>331072

TGCAGGAGCTGACGGAGCAGAACGCCTCCGGCCAGCTGGAGACCAAGAAGCTGAAGGTGGGATCGGGTCAGAACCTCCCCTGCCGTCCCGGTCCGG

>100096

TGCAGACCCCTCTACAACGGCCTCTGACTCGCTCTGATGGTATTTTTCTTCCATCTTCGCTGACGTGATATTTTCAAATAACTTTGTTCAGTTGGT

>161762

TGCAGACGGCAACTGTTGCCGGGTTCATAGAGGAGGGCAGAGGAGCCAAAAACTAAACTCGAGGAAGACTTGAAGATATTCTCCTCAAGTAAACAC

>290736

TGCAGATTTTATTTCAATCAGTTCTGATATCAACCTGTTGACCAGCTGCTTCTCACCATTCTGTTGTTGAATGTCGATGTTTTGATCTACGTCAAG

>256728

TGCAGAGTTCACCTTCAAACAACAGTGTAATCTGTTGTCTAAAAATAGTCCCGCTGTGACATATTTGGTGCTCGCATGCTGGCTCTCACTTTTCTT

>206972

TGCAGTTCAGGCTCCGCCCCCAACGCCGCTCTGGATTGGCCAGGAGGGTTTGACGGACAGCCGGCGGGTGGAGCTAAAGCAGCAGGTGGAGGAGTA

>186304

TGCAGCGTAAACTCAAACGGTGCACTTCATGGGCATAAACGAAACTTTTCCGAAAAGTTTCACTGGAGTCAAAAAGTGTCTCTGGTCTCTGGTGTC

>193435

TGCAGGACAGACAACTGTTAGACTGACTGTAGGTCCTTTTAGGAGCAAGGGGGAATCTAAGCATCCTATGCTGCTTGCTCATGACAGAGCAATGCT

>281745

TGCAGGTAGTTGGCTCCCAGGCGATGGCGATGCGTGTCTGGGTAGGAGAAGAGCCGACCCTACGCCGAGCCGGACCAGGTTAAGATCGGAAGAGCG

>234582

TGCAGCATGAGAACTCCCGGAAGCAGAGTGTGTCCGACCTGAAAAGGGGACTCGCTCCCCGTCACACTCACTGGCATAGTGGCCAAACTGATTACA

>158050

TGCAGACACCAGATCAGCTCTACCACTAATTTATCTCACATCAGAAGCTACAGAAAATGGGCTGCATCATGATCTGAAGGCTGACTCTTCAGAATG

>276090

TGCAGTCAGATTTTCCAGCTCAGATTTCACCCGTCTGTCTGACAAGGACATGAAAGTCAGAAACTCACAGCATGTCTCCACAAGTAGACGTCCAGA

>140969

TGCAGCGTCAAGGCGGCTCCCCGAAACGCTGTTTTCAGTCGTCCACATCTGATCCGCGTCACCTTCCAGTGGCGAGGGGACGTTTCTCTGACGGCC

>1907

TGCAGACCCTCCAGTCTCTGGTTGGACCTGGACTCCTCTGTAAACAGTGAAGCTTCATCCAAACCGGGTCAACAGCAGAACCTTGATCCATTCAGC

>14116

TGCAGAACAGTGAGGTGTAGAACCAGAACCAGGAGGAGGTTCTGCGGACCTTCTGATTCTGAAGGCGCCGGCGGCCTCCGGCTTCCGGTCGGCAGC

>87265

TGCAGAGAGACGGAGCTGTCAGGGAACAACCCTCACACACAAATTTATTCTAAAGGACCATGTTTACATACACGGACCAAAGTTTTGCTTTATCAC

>172708

TGCAGCACCTCCTTCTCGCTTGCTGCTTCCCAGTCCATCTACCAGTATGTATTTTTCTAAATGGAAGATGAAGGAAGAATATGTCTCTAAAATAAC

>36070

TGCAGCTGCCAGTGTTACAGACCTGACTATGTCTTTGTTGGCAGACAGGGAGAAAAGCTGCTGTGATCTTCTGCTTGGAGTGGAGCGTTTGTCTCT

>12652

TGCAGCAGCCAGACTTGATGTTTTTGTGCAGCGACTCTGTGACACGTGGGGGCTAGAGTTATGTGAGGTGCGAACGTTTCATTGTACAGGGCAGCC

>84593

TGCAGACACAGCATGATAGGCTGGAAACAACATCAACAGTGCACGCGTGCTGGCAGCGACAACGCTGTCAGCCATGCGTGTCTGACATATCATTTT

>243116

TGCAGCATCTTCCTGAGCTCCTCATCCACGCCGCGCTGCCCCAGTTGCTCCAGGCTGAAGTAGCGGTGCACCACGGCGTCGGCCGTCACGGCCACC

>59939

TGCAGATCTAATCCATGCTAATCCAGCAGCTACCAGACCCGATCAGCTCCTTGGATGACCTTTGACCCTTTATGTTCTGCCAATCAGCTTTGACCT

>130077

TGCAGCATATATTTCGTTATGTCTGCATCTTGAGGGATGAACACACACAAGGTTGCACTACAGGAGACCCTCATTGGCATAACTCATAGCTGTCAG

>116835

TGCAGGTCTGACCTGCGGCTCCTTCAGGGACAAGAATCCACATGTCTGATTCCCATCAGGGTTTCTGAAGAAATGTTTCACAACATTTTCCACACC

>287681

TGCAGCACAGGGCACACACTAGTAAACTGAGACCCCCCAGAGAATAAAACCAACGTCCTTGTGCCATTTCCTTTCTCCTTTTACACAAACAAGGAT

>205390

TGCAGCTGCAGCTCTGGAACAAACAGTTAGAACTGAGCAGCTGCCAGCAGGGGGCGCCACTGGATTATAAGCCTGGCAGGTCGCTCCGCTATACAA

>190267

TGCAGTTTGAGACCTTTGTCCTCAGTAAATTCTGCCAATTTTACAATGAAATTGTGAGTTCTGATTGAGGGTCCCTGTAGCAGACGAAATATTTTT

>100137

TGCAGGTCAAACCATCAGGACCTGGTCAGTTTTTCTGGCAACATTTTCGACGACATCTTCTTGGAGCGGGAGATTCCCACCTGGATCGGACTCCAC

>132529

TGCAGCAGCTGGGTGATGCTAACATGTCAGCATGGTTCAAAGTTCTCAAGGGACATTTCTAACATTTTGTTGAACCCAAGTCATGGAAAAGGCAGA

>63258

TGCAGCCTGTATTTCTTCTGATTTGGAATGGAGCTAAGGTGCTGGCACATAAAACATTATCACAAACGGAACAGACTCTTTCAGGCTTCATGCATG

>19746

TGCAGCAACAACAAATAAAGTTGTAAGAACCTCAAATGGTCCATGCGCCTCAACGACGCTCTGGCGTATGTTGCAGTTGCAAACCTGCCGGCGCTT

>128482

TGCAGCCCTTTGAAGCCACGACAGCACGCTTTTATCTTGTACCTTGTAGAAAGCAACTTTATATTCCTCAGGAGCCTGACTGACTGGTGGAGCCGT

>82301

TGCAGCTTGTTTTGCTTCAGCAGAGACATAGCCAATGAGATAAACAAGCTGAACCTGAGGTTCCACCAAGTCTAAATCTGCTATGATACAGGATTA

>252484

TGCAGCGGGACGAAGGCAGTCACACCAGCCGGAACCAGCTGGATCCCCCCGACCGGCACCATGCTGTAGGGCGACCGGGCCGGCTGCTGCGAGTGC

>27256

TGCAGAGGTACAGAAACACCAAAACACGGATCAATTATTTACACAAACAGACATAATTCAGGGTGAAACATGGTTTTTACCAACATGGACTGAAAT

>317846

TGCAGAGGTAATTAGCTTCAACAGTGTGAAGGAGGAAAGAAGCCCGGATGACAGCTTAGCGACTGTCGCTGTATTTATCAACCTTCAAAGTCCAGT

>295651

TGCAGCTCCTCTCAGAGCGCCGCCTGGACCCCACCACCGCCTCCATGGCCAAGTTCTGGTGAGAAACCTGTGCGATCAGCTGGCTAGCGGCCACTG

>36546

TGCAGGAGCCCAGCAGCCCGCCCGCCGAGGCCACGCAGCCTGCCAGCACCGCTGCTTCTGTGGAGGCGTCGTCGACCCAAGAGGCCTCCGGCAACC

>55706

TGCAGGAAATGCGTTTTTCCTCCGGTTCTGGCCTCGGTCGGCTCCAGGGGGCCTGATAAGAGCCTCGTCCCCCCAAAACGAGCTGCTATCGCTCAG

>151175

TGCAGTTCAGCGGATGAAGGTGGCCAACGAGCGGCACAGCAAGACGATCACACAGCGGGGACACGTCCAGAAAACCTCGGTGGGTTCGGACATGCA

>194433

TGCAGCACTTCCTGTTTGCACAGTGGACCTCAGGAATTAGGAACCAGCATTTGCTGTTTGAGCGACTTGCTAAAAGTCACAAATACTTGAGACTCC

>330425

TGCAGCTCAGAGCATCAGCAAAATAACCCGTCATTCTGTGAGGTAATTGACTGTGTGGATGACTATTGCAAGGATGCCATTGACTTGACTACCATC

>22376

TGCAGTCACCAGTAGGTCCCAGCCAGTCGCAGCCTTTCTGCTGATCCCACTGAAGCCAACAGAACCTGCCTGGTTCCGGCCGCTCGGATCCGATTG

>140204

TGCAGCACTCTGAAGGTACCTCACACCTGCTAGTCTCGTTATGCAACGAGGAAGGATATTTGAAACCTCATCTTTTTGCAGGTTTTTGATAGCAAA

>143437

TGCAGCTGCTGGGCCTCCTCTGCCAGACGGAGAAACAGGAGAGGAGCTGCGTTTTGCTCCACTAATCTCAAAAACTTCACAACTAAAATAAGAAGG

>88088

TGCAGTGATTCCAGGCTTGATCTCACGTCCATTGAAATGTCCAGTGGCAGGATCGTCAAGCTTTCCATGAGGCCAGTGACTCTGAAAAGAAGAAAG

>218131

TGCAGAGGCACTTTTTGGATTTATGCGTTGGATCCAAACTGCCTGCAACACTGAAAAATGATAAATCAGCATATAAATGACCGCATTATTGAGAGA

>53077

TGCAGAACACAGCAATTTCTCCCAAGTCCCAGATGAGACCCAGTCAAGGTGAACACAAGTACATGATGAAACTTGAAGTAATCTAGCCCCCAGCAG

>143212

TGCAGGAGAACAGGAAGGTTCTGGTCGTTTCTCTGTTTGCGTTTCTGTCTGACTCTGAGTCGCGATGCGGCACCGGGTTTCCATGAGCACCTGGTA

>319497

TGCAGCTGCTGCCTGTCAGAAGACTCATTACTCAGACGGATGTCTGGTGGAGTTTCTGCTCCTCTGCGGAGTTTTAAGATCGGAAGAGCGGTTCAG

>275676

TGCAGCCTCCCATTTATCACCATTTATAATGCAGACTCAAAACAAACGTCCACCCAACTCGTCCGAACAAAGGTATATTTTTATGACACACTGACT

>139684

TGCAGGAAGCTGGGCGCCAGCGAGGGAACCATCAACCAGGAGATCCAGCGCTACCAGCAGCTGGAGTCGGTGGCCGTCCAGGACATCCGGAGGGAC

>188031

TGCAGCGGTTCGCCTGCGCCGTGGTCTGCATCCAGAGGGCCGACATGTGAGTCCGGCTCGGGTTTCTAACCGGACCTGGGAGCCGAGCCCGGATCC

>154326

TGCAGGTTCTTCGGGGGAACCAACATGGGCCTGGACTTCTTCCGGCTGGAGGAGCTGACGCCGTCGTCCTCGTCCGCCTTCTCCTCTTCCAGCGCC

>65857

TGCAGTTGATCTATTTGAAGAGTACGTACAGCTAAGTAAAATTGCATTTCCAAGAGCCCTTACTCCTCCAGATGTCACTACTGAACCCATTGCAGT

>89867

TGCAGGACAGCTCTCGCTAACGTTTTTTTTCATCTGATAAATAGATTACTGCAACTTTTTGGCTCTCATGCTTGGTGGGCAAAAATCCTCATTGTT

>102371

TGCAGGACCATAGCATGGAATAAACTCCTGAGCTACACTACAGCTAGTTCACTACTAGTCTGCCATGAAACAGCTTATAAAACAGTTTTCCTGGAA

>112007

TGCAGCACACAGAGCAGGTTCCCCCCATCCGCATGGCTGCACCGCATGATTGGACCAGGCTGGCCGTCTGGGAAGCTACTGGAGCTGTGTGTGTGT

>15677

TGCAGCCAGGGTCCTGACCAACACCAGGAAAATGAGCTGTAATATTCTGCTGCTCATCTATAAATCACTGAATTGTTTCTGAGTTGGTCATCAAGT

>41997

TGCAGAGCCGGATGCCACGGAGAAGCAGCTGAACCACCGCCGCCACCGCGTCACCCTGTTCTCAACGCTGTCGCGCTTTTAGTTGATATATTTGGT

>124492

TGCAGGCTCACAGCAGCCACCAGCTGTTGGCCCGGCTGGCTCCGGTTCTGTCCGGTAGCTTCTGGATGGGACTGCGACTGCCGGCCGGGTCCTGCA

>221108

TGCAGGTGTGCTGAGAGTGTGGGAGGCAAGCACTGCTCGCTGTGTTTACACCCAGAGCCTTCCCTCGACCCTCACGCCGTCCTCTGAGGAAGACAA

>143592

TGCAGACAGAATAGTGCGGCGCACAATGCCTGGTCTTTCATCGCGGCGCCTAATGCAACCAGGTAGAAACACCTGCCACTCGGAAGCTTTCATCTC

>140812

TGCAGGCCGACAGCCTCCCACTTCACCCACCTTGGCATCCAGCAGACGGACAATAATTCCTCCTGAAAGCTGCCCAGCTGTCTCTGACACAGCAGA

>37786

TGCAGACGCTGCACAGCCAGACTCTGATTTTCGTTTAGCTCAGCAAACCCAGCTGCTGCCAGCAACTTTGTTTCCTGCAAGCAAACTGGCTCTTTA

>222916

TGCAGATAAATCCTGTAACCTTCAGCTCGGCGGAGTTTTAGGAGCCACACGGCACACGCACATTTGTGCTCCCGTTGCAATGACGATTAAGATCGG

>312930

TGCAGACGTAGAAATAAGGGAAACAGGAAGAGCGGAGAAGGAAAGGAGAAAGACAGAGAAGCGCGAGAGAAGAGAGAGAATTTGTAGGTGATGGCG

>162031

TGCAGCAGAGGGTGAGCTCCTCCATCTCATTAGTTATTTTTGGAAAAGAAATGTTCAGCTGTCTGGTATCCCTCAACCTTTGCTAGAGTCCAACCA

>104558

TGCAGAGCATCTATCTCCGGTGCTGGAAGCCGGAGCGGAGAAAAGAGAGGCGCGGGATGGAGATGGAGATGGTGGGATGGAGGGATAGAGGGATGG

>122847

TGCAGCATGGCGTATCCTCAGGGCTACTTGTACCAGCCGTCCGCCTCTCTAGCCCTGTACTCTTGCCCCGCGTACAGCACCAGCGTCATATCGGGA

>241417

TGCAGTCATGTGAGCAGCAGATACTGATATGAGAAGCGTTATGTGGAGGCGGTTATTTCCTCAGGCCGCCAGGCTCCTCCCAGCAGAACCGCCTGA

>228847

TGCAGCTAAAAGAGCCAGTTATTTTGTTTTCCTTCACTCAGCCAACTTCACATCAACTACAGTTACGGTAAATCTCATTCCTCAGTGTTTTACTCT

>273544

TGCAGTACAAGGGAGAGCTGCGAACACGTTTTATCCACTGAAGCACAATCCATGCAAAAACAGAACTGCTGTCAAAAGAAGATACATTGCAGACAC

>120597

TGCAGGAACATACGGCAGTAGGAATGGTTTCCTTTGATGACGGCGTAGCGCAGCAGGAAACCATTCTGAATATCGATGTTGGCGCTGTGGTCCAGC

>216469

TGCAGCCGCGCTGTTCGGTCCAGAAGCAGCACATCGAGGCGCCTGGGGACGGCACTCCGCTATGGCAAGCTGTTTTAAGATCGGAAGAGCGGTTCA

>302566

TGCAGCACAACCGACCTGCTCCTCACAGCAACAGGTGGGGGAGGGGAAGCCGCTTTAGACAAACCAAATATAACACTTTAGTTTGTTAAGATCGGA

>106122

TGCAGGGGTCAGAGGTCAGCGCCCCGGCCGGCCACGACGGAGAGGTCCGGGCTCGCCGCTGCGCCTCGACGGCCAGAGGCTGCTGGGAGATCTGGA

>192057

TGCAGTGGACCTGCGGCCTGAGCGCCACCAGGTCTGTTTCCTGTTATTTTGCTTTTGCTCCTAAAAGCCAATCTGAGTCTCAGGTTTGATCCGATT

>165127

TGCAGGCAGACCAACGCCAAACAGACCCGAATGGAGGCTCTCGACGTGGAGGTGGTGAGGAAAGCTGCCGTTGAATATGCACGGCGAGTTAAGATC

>293006

TGCAGCGCGAGGCCGGGGATTTCCTTCCTGCTCACTAAACAACATTGTCTGGGAGTCACAGGACTTCAATTTCAGCTTCATGCATTCATGACTTGT

>339650

TGCAGACAGCTGAGAAAACCTGTTACTGTCAATTTAGTCCTGTCAGGACCCAGTCTGGCAAAAATAATGACTATGCAAAAGGGTTTTTTAGCCAGA

>125085

TGCAGTGACCAATCAGCAGAGGGTTGCTGCATCACTGCGGTTGGTTTGGAGAGTTCCTTGATTTAGATTTGAAGCCTCTAGATCAGTGGAGCCCAA

>148615

TGCAGGAAGAAAAATGACATGTAAAGTTTTCCTCCTTTGTCAGCAAGCCCTCGCGTCACGTGGGGAGAGCTTTGGGTTATCACCAGCGGACACTTT

>46825

TGCAGGTGATAGAGAGGTTGTGGGTCCAGATGAACCAGTCACAGTGGAGGTGGGACAGGTTGTTGTCCTGCCGTGTCATTTGGAGCCCCAGTCTGC

>304816

TGCAGAAACAAGAAGCGATCACGTTGCATTCGGACAGCGAACATTTTATCTGCAATGTGTTTGTGCTCCTTTCCAAAAGTGGCTCTCATCGAGTCT

>252938

TGCAGAAGCATTGCTTGAAAGTTCACCGGCAGCCCATATCTACGGGAGAACAACATGGCAGACATGTTCACTACCACTGGGTTTATTTACAGCAGA

>258966

TGCAGAGAGACTGAGCATGGCAGGTTGTTGGGAATCTGGAAAAACAAAATGAAGCAGCTTCTAATCAAATAAGTCATTTTTTGTTACACTGTTTAC

>181019

TGCAGACAGAATAGAGCAGAACCTCACTTAGGTTGCAGAAGACACCGGACTTTTGATATGCTGCAACAACTACTGTGTAAAACCCTTATAAAGGAA

>109015

TGCAGCTGATTCCTCCGGGAACGTGTAATTTTCTCATTTAGCTCACAAGCGTTTGGCCCGACACCAATTAGCACAGGGCCCTGACCCCAGACGTCT

>86136

TGCAGGATGATTAGGTCTGTGTCGTGGGACAACGGGCACTTGGTGCCGTTGGGACACCAGCCGAAAGCCTGAAAACAAAAGCAAAAACAATAAACA

>18311

TGCAGATCAGCAGTAACTCCAGCCTCAGAAAGGTCAAAGGTCAGGCTGGATGTGCAGAGAAAACCAGAGTCCTCCGACATCAAGACGTTTCTAAAC

>77410

TGCAGCTGTTCTCCTCGTCTGTGGACTGCGGCGTTCGGCTGTGGGACCTGCGCTCCAGCCAGTGTGTGTGCGTCCTGGAGAGCCACTACAGCGCCG

>170732

TGCAGTTGCCCTTCAGCCATCAGTTGAAATATAATGCAGCCAATGTTTCCTAATCTCTGGTGTTTTGTCTCAAAATTTCATCAGCTGTTTTCCTCA

>78419

TGCAGAGCAGTTACACAATGCAGCACAGCACCAGTGGGAAAAACTGGTGTTTATAGCAATCCCTGCCCAGTGCTTAGGTTCTCATGTTCGCTCAGC

>97564

TGCAGTGTTCTGCATGCCGGTGTCGCAGCCTACACAGCAGGATGAGCAGTTTGCAGAGGTATGTGCACTTGACCTGTATTGAACGTACTGAGTAGT

>328393

TGCAGGCACCTGTTCCCCAGAGCTGCACCTGGAGCCTCCAGTGGCCCAGTTCTGCTCACCTACCAGAACCAGCAGAACCTGGGCTGGTTCTGGTGG

>193890

TGCAGCAGTTCCCACCTGGCAGACGGCTTAGATTCACACAGATGCAAAACACACACAGAAAAACACACTACTTACACTGACTCTGTGTAGCTCAGC

>86641

TGCAGTAAAAGGTCATTCATTGTGAGTGTGTGGTTGTTATTTGGTCACACTTTCTTCCTATAACCAGTATCTCTTCTTTCCTGGCAACTTTTACCT

>283853

TGCAGTGTTTGTCCGGTTCAACCGCGGTCCCGGCGTTGCCGTGGAGCTGAGTGCGGCGGCCCGAGTGGCCGAGCTGAAGGACATCGTGGCGAGTCA

>317467

TGCAGCCGGAGGAAAACTCCTCCATTCCTTACATTCCTCCTGCATTCCTGCTCCAGGTCTAAATTCTCCACCTCTCTCTCTGCATCAGCATCACAC

>48159

TGCAGTGATTGGAGATGTAGTCCAGGTGTACAGCGGCAGCCATGGCAGAACAATCGTCTTCTGCGAGACTAAGAAGGAAGCCAATGAACTGGCCAT

>57468

TGCAGTGAGCGTACACTGAAATGACACCGAGGTCCAGGGGTCAAAAATAGACTCACACAGCTTTCACTGTTAGGGGCCACCAAAAAGAGGAGCCTA

>88253

TGCAGAGGTGCATGGTATTTTGACTGTTCAAGTCAAAATACCATGACATACCATCGAACTCCAAGTTGTGGAAATTGGCAGGCTCAGGTGCCATTG

>184844

TGCAGGCTCCTGTTGGGCCGTCATGTCCTCCATGTGTCTGTTTTATCGTTTTACGGCTGGCAGCATTTACTCTTTCTGCTCGTCATGAGAACTCTG

>101891

TGCAGCTCGTCCAGGATCTGGCAGCACTGCTCCAGCTCGGGAACGTCGCCGTCGGGGAACGGCGCGTGGTGCACCGTGAAGCCGCGTTGCTCGTAG

>107942

TGCAGATGAGAATAAAATGTGACGTGCAGAAAAACTTCCCTGTAACGAGACACAGAGACTCAATAAAATGTGTCAGTGAGCTTTGGAGAAGCTTTC

>163752

TGCAGAGACATCAGGAGTGATGTCATCACCAAAACCTCCCCTGGTCACAGACCCGTCCATTGAGTGAAGTCATTTCTGCTCTTCTTTCAGAACCAT

>209959

TGCAGTGTGGCCCACATGCTTGTGCTGCTGGAGGGAGCGTAGTGCAGAATTTGAATACTCTTAGGGGTACCAAAGACTTGACTTGTGCTCAGACAG

>307785

TGCAGTTCCGCCTGGCTGCGAGGGCGGGGAAAAGTCACCAAACAGTCATTCATAGCGAGGATGTAAATACTGGCGCATGTGAAGAATATCAAACAT

>133276

TGCAGTCCACTTCTGCCTAGCAGGCTGTCCACAGGGTCGGTTCACGGAGTTGTTTTGATATGAGACCCTCCCTGCCAGTTGGGCTATTTTTATATT

>166708

TGCAGAGTTTGGACCAGTTTGGACTCTGACGCTTCGGAACAGATTTACAGACGGAAGGTTTTTATGTCTTTCTGCGGTTTTGCGGCTCTAAGCTGG

>106265

TGCAGATAAGACTCTCAAGCAGCCGTCCTGAATGCAGATGTCTCCCAAAGGAATCAAAGACTTGATGTGGGACCAGAACCCGTCTAATTCCTGGAC

>195521

TGCAGAAACATCTCCTACCGTATTGAGATGCTTTTCTTTTTCTCTTTGTTTTTCGGGTCTCTGAACTCGGTGACCGTCTGTGTCTCAGTCCCGATG

>254041

TGCAGACTCTGGAGAGGGCGCTGCACGCTCTGGACCAGCTTCCGGCGGAGTACCGGGACTTCTACGGCCGGCGGATGGTCCAACGCATCCAGAACC

>237751

TGCAGCCAGGCAGGCAGGGATCCACTCTGGACAGAAGACACAGAGCACAACAGTGCAGGGAAACGTGATCCGATAACTGAAGGCGCGCTCACAGGC

>108260

TGCAGCCTGGACACACAGCGGACATTTTCCTCACACCTGGAGCCACGCTGAGGGTCAGCTTCCAGACGAAGTCCAGTCATCAGTCATGAAGGACGT

>237613

TGCAGTTGTCCACCAGAGGGAGCCAGGAGGTTCCCAGGTGTTGGTCTGATGCACCTGGTCGCCGGTCTTCTTCTCTCTGAAGCTTCCAGTTCTGCA

>31572

TGCAGGCAAACATCATCCATTTGACTGTTGCAGATGTTTACAGCGTCGCATTCAAGTCCAAATGTAGGTATTCTGGTGGCAGAGATGCAGAAGGAG

>36709

TGCAGCTCCAACAGCTGGTTCCAATCACACCGCAGAAGATACAGTTATAGCCCTTTGAAACACTTGTCATATGATGTAAACGTGCCACTTCAGACA

>164436

TGCAGCTGAGATTGGGTCAGTTCCTCTTACACTTGCCTCAACAGATTTTGGTGCCCAGATCTTGCCCAACGTCTTGGGTAAAATGTTCTTCAGCAG

>211137

TGCAGGTGGAGGAGGTGCAGATGAAAGAGGAGGAGATGAAGAGGGAGATCGACAGACTGAGGAGAGAGAGAGACAGATTGGAGGAAAGAACCCGAC

>14076

TGCAGCTGAGCAGCAACGCCAAGAACCACAAGGAGTTACTGCTCCTCCCCCACCAACACCAAGCACCCCCGCCACATCATATATAGCAACAGCACA

>224611

TGCAGCGTTGAACAAGCGTTCCCCGGTGAAATCTAACGTTTCTCCTACTTACTGCTGGTTTTTTATCCTTGCAGTAGTGCAAGCGAAGCTTTACCT

>49789

TGCAGCTGTGAACAGAGTCTAAACAGCCGAATGTTTCAGCTTCATGCTGCTGCTAATGAGGCCCAGATTAGACCCGTTTGAAGTGCAGAAGATCTC

>114438

TGCAGGTCTGCTGTGGTTGAAGAGCTGAAGGTTCTCAGTAGGTTGAGATGCTATGACTCAATCTTGGCTCTAACCGGGAAATACCACTACGAGGAT

>281132

TGCAGCCACTCCATGGGAAGACACAAGGGATGAGAGTGGAGAGGATTCGTAATTTTGTAATTCTGCTTTTTTTATCTGGATATCTCAACTACAGGC

>83531

TGCAGTCTGTGTTTCATGAGATGCTCATGCCTACTGCTGCAACCTGCCGTCTTCTTCTCCTCCCCCGAGTTTCTGCTTGATACTCTTCTGTCTCTC

>56985

TGCAGTAGCAGGGAAATAACAGGAGTGTTACCTACTCTCTGGCTGGCAGCCTGAGTTAGAGAAACATACCTGGTAATTTTATCAGCGAATAGGTGA

>64614

TGCAGCGCTCTGACCCTTTTTTCTCTCCGTAGTGAGGACGACATCTTGAAGGAGCTGGAGGAGCTTTCTCTGGAGGCTCAGGGAGGAAAGGCCAAG

>162717

TGCAGCTCTTTCTGGATGTATAACTTTGCTTCCTTATCTGTTCTTGAATATATTTAGCTAAAAACATGATGTGCTGTCTTTATCATCCTCATTGCA

>50905

TGCAGAGTCACATGCAGATCATGTGGACACCGTGAACCCAAGTAAAAATGCAGATGCATATGGGTTCATGATAGAACATCTGCAAAATCTAGTAAA

>160763

TGCAGAATCAGCGGCAAGCATGTCATAACGTTAGGGTCTGTTGTTTTACTCTACACCAACTTGTAATGCTTTGCCATGTAATCGCATAATTTCAGC

>39233

TGCAGTTCTTTGTGGCTCACAGCTCAGCGGCGGACAAAGGGACGATCTACAGAGGTGAGACTCATTCAGGCCTCTGATCCCTTACGATTCTGTGTG

>178630

TGCAGTTCCCCAGCTGAAGAAGCCAACGCTCACAGACAGAGCTTTAGACTGCCACGAATGTGTCTCTGCTCATTTTTTTGCTGAAGATGTTTATGT

>158358

TGCAGCTCCGATCAAAACCTTCTGATCGATAACCAGCAGAAAACTCAGCGGTTCTGTTATCGGCTTCATCTGGACTCTGGTTTGGACTTTACCAGA

>23862

TGCAGGACGGGGATGACCGGCGGCTGCCTCTGCTGTAGGAAGTAGAGAACCATCAGGATGTAGGCGTAAGACTAGAGGCTTCCTCTGGAGGCGTCG

>89346

TGCAGGTTGTCCCATGTGACGCGGTTTTAGATGTAGCCCTCACTTCCTGTCCCCGCTAAACGTCACGCTCCGTCTTTAGTACTTACTTAAGATCGG

>186517

TGCAGCAGGATGCTCCCTTTCTGCTGTCCCTTTCCATCGCTCTTATCGCTCCGTGCTATAATCCGTCATATTCCTCCTCTCCTGCCCCAGCCACCT

>264242

TGCAGAGAAACCAAGCTGCATCGAGACTCTACAGGCCTCTAAAGTTTGCCTGTTGAAACATAACATGACAGCACATTTCCCAAAAAGACCAAGCGA

>303011

TGCAGTGTGTAGGAGTGTTTGAAGAGAAACCTGATGATTTCTTGCTGAGTGCTCCACACAGGTACGTTCTGTTGGTGTGTGTAGGAGGACGGATGG

>169620

TGCAGCCTCAGGCACCGTCTGCCCAGGGCTCAGGTCTGTTTTATGGACCAGCTGCACTGAAACATCAGTTTCGTCCCCAAGGGACTAACAAACATT

>138506

TGCAGGTCAGAGGTCACCCGGAGGTCAGGCCGCAGCTCTGACCACCTCCGTCTTCTTCTTGTATGGAAACAAAGAGCAGCTGGAGAGAAGCAGCTT

>207081

TGCAGGAACGCAAACATAATAGTCACCCACATCTAAATCATAAAAAAGGGACTTCCTGCGAAGGCGTTATGAAACAGAAATATTACATATTCAGTG

>337042

TGCAGGCATTTCTGGAGGCTGTAAAGTGCTGGAGTGAGGTGGAGGTTTTCCTTCCAGTACACCAACAACCTCAAACCTCATCCTGATTGGATCCTT

>272113

TGCAGTTTTCAACCAATAAGCTTTGGAAACATTTTTGGACCTCTATTGCATCCACCTTACAGCAACATAAACTTGGATCAGCATCCAACCATGCAA

>315118

TGCAGCAGGAGGAGGACCATCCAGTGAGAGTCACTCAAGGCTGGGATGCAGTGGAGGAACCAGGAGTTGTTTTAAGATCGGAAGAGCGGTTCAGCA

>61684

TGCAGCGTTATGGATACATATCCTCCTCCAAACACAGCAGCAGATGAGGGTGAGCTCATCCTGTCATCAGCAGAGGATCGGACACGTCCCTCTTTG

>74675

TGCAGGTATTGGAGGAAATGATGGTAGTGTTCAGGGTAGAGGGATCATGTCTGCCTGTGGGGACCATAGGAGTGTCTTGGATGGGGCGGATCATGG

>86380

TGCAGGGATTCGCCTGAGGCTGAAACCAAAACCTGTCCGGTGCAAACGAGCCCAGTTTGAGTTCCGATCCCAGATTCTGTCAGACGGAACCAACAG

>17182

TGCAGCGCGTTGTTGGCGCCGCGCAGGTAGTGGGTTCCTGAGGAAGAGGAGGAGGAAGAGGACGGGTCAGAAAATAAAGATTCTTCAAGTCAACGT

>306826

TGCAGTTTTAGTGGTGCATGCACCTGGGGGCCTGGTATAATTAGGTGGAAATGTTCGGGGCCTTGAAAGAATTCAGAGGTCTCTGAGTGGATCCAA

>43844

TGCAGGAGCCATCGCCTTCCTTGTCATCAACGCTTTCCTTATGCTGATTGGTAAGTTTTACCTATGCATTTTCTGTTGTCAAGATTTCTTTCTGTT

>154285

TGCAGTCCCACCCTCACATCGTGGGTCCGGGAGCGGCAGAGGTGTCACCCAGACAAACGGCTCAGCCTGGCAGTCATCGAGCTGGAGAGCTATTTC

>181406

TGCAGATCATGTGTGACTGGCTGAAAACGTGTAGCGCACTAAATAAAAGCGTGGCAATCTAACAGAAAACTGAATGTATGAAACTCCCAAATGGGA

>318077

TGCAGCCAGCAGAACCTCCCCTCACGCCGCATCACTTCCTCCCAGGTTTGCGTGCACCGCGGGGGCTTATCGTCTTAAGATCGGAAGAGCGGTTCA

>23333

TGCAGGTGCATCCAGTTGCCGTACGGGCAGAGCTCGTTGTTGCAGAGCACCCGCTGGTGGTCGTCCTTCTCCAGGTCGACGGAGCGGCCCAGGCTG

>30961

TGCAGCTGGAGGCCGCCGTGATCCCAGAGCATCAGGACTCACCGAGCTGCCGCCGTCCTCCAGCGGCCCGTTCGCTTCCTAGTGTGTATCAGGCAG

>257897

TGCAGTAGATAACTTGTGGAGACTGACTTTAGTATAAATTGAGTGATTCCAAAAGATAAAGGTCATGTCTCATTTAGAGAAGCCAAATCAAAAATG

>310485

TGCAGGTTCTTCTGGGTCAGCGCCAGCTCCTCCGTCTTCCACGATAACCTGGGCAGGAGGTCAGAGCGGCTTTTACTCTGGAAGGTGACAGGAAGT

>109873

TGCAGATCTGAAGTCGGGCTTGAGGCTCTGATCTGAAACTCATCTCTATGTGTAAAATTCACACTACGTGTGTTCAGCTTTATGTTCTCTGCACAG

>101691

TGCAGGGAGGAACACAGACTGCTACTGCTGCACCGCCATCCATCCAGTGATCAGGTCTAGATTGGGTCAGGTGCCTCGATGTGGAGGCTCCTGACG

>220446

TGCAGAGCGATGGATGGATGGATGGATGGATGGATGGATGGATGGATGGATGGATGGATGGATGGATGGATGGATGGATGGATGGATGGATGGATG

>230462

TGCAGTTGTTCCTCTTCGGCTGCTGGACTGAATAAGACTCTTCAAAGTATTACTCTGTAATCTCATCAGTAAATCTCTTCACGACTTTTTTCTCAA

>27490

TGCAGAGGAAGAGGAGGAGGAAGAGGAGGCTGAGGAGGTAAAGGGAGAAGAGGAGGAGGAGCAGGAAGAAGAGGAGGGTGGAGAAGAGCAGAAAGA

>69318

TGCAGCTGTAATCAGAGACAATCACTTTATATGGACCCTTTGTTACCATGGTGATGAACTCGGACACACTGGCTGTTTCCACTCTGTGATTCCTGC

>146976

TGCAGATCTTCATGCTCTTCATGCTGGCAGAGCGAAGCGCCCAGTGAATTCTGAATCTGAAAGGAGAATTGTTTGTGAACTTTGTAGTTCTAGGAC

>124474

TGCAGCTACAGGTAACAGGTGTGACAGATTTATGAGAAACCTGCCTCACCTGGCCTGCTCCTCCACCAGGTGCTCCACCTCCGTCTTCAGCGCCTT

>37538

TGCAGACCTGCTCGGTTCCACCTCATCTGGGTTCAATTAGAATCTGGGTGATGGATCGAAGTTTCACAGCCAGGCAGAACCGGTTCTGAAGCAAAC

>303521

TGCAGATGAAGCTTCCAAGGTCAGCAGAAGGTCACAGTTCAGACTGGTCCAGAAACCCAGTTTGTCAGGAGGACTGTCCCTTTAAGATCGGAAGAG

>11414

TGCAGCTGGAATTGAAGCAAAAACTGGTGCTAAACCAAGGTGCATGCCACACTCTTCAGATTTGATTTGTTGTTTAGAAAAAGAAAACTCTGAAAA

>238142

TGCAGTTTACATCCACCACACCTCTGCCGCTCCGTGGGAGGGTCACGACCCCGTAGCAAGAGCTCCTTCTACCAGCAGAGATCAGACAGACACATG

>86778

TGCAGGAAACAGAAGCCGAGCCGTCACCATGGAGACTAACTGCCATAAAGCAGACCGAAATATGCTGCGCAAATCAGCCGAGGTTCAAACAAACCC

>120082

TGCAGATGTGCCGGCGTTCCCATTCCAGACATGAAAGCTGCCGTCGTGTCCAGATCGAGTTACATCGCTCATTTACGCAGCAGAACCAGAGCTGCC

>180641

TGCAGCGTCAGCACGCTGATCCCTCAGGTCGTAGGGTTTCCATAGAACCTCCGGAGGTTTTATGGACTCTTCAGAATCACTTGAATTTCAGAATGA

>129819

TGCAGAGATCCACGTCTCACATTGGAGTATATGTTGCTAAGGCAACTATTAGTTGCACATTTCAAAGATGTAGCTGTGCTGGACCTGTGGCAAGAA

>39972

TGCAGCAGCGGCAGCCGCAGCAGCAGCAGGACGTGGTGATACCTGACTCCGGAGCAAAAAACCCGCAAATACCGATGGCTTCATGTGTAAATATTT

>312684

TGCAGAGCAGCAGCAGGGACTGGGCTAAAATGACTAAACACTGCAAGCTGCTAAATGACAAAGACCATGAAATCTGGGATATTTATGCAAAGATCT

>31835

TGCAGACACAAAGTTCAGCTTTCAGTAAAAACATAAACAAACCATCCAACAATTTATGGGCATCGTCGTCCATTTTACCTGATAAACTTCTCTGCT

>314366

TGCAGCAACATCTGGAGAAGAAGAAGCGGAAGGTCGGTTGCAAAAAATCGCACAATTTACTGAGTTTTCTTTTTGTTTTGAACCTTTTTTGTTTGT

>171481

TGCAGGCACACAGCAGGAAGCAAAACCATAACACCCGAAAAGAGATGAACACCTAAGATGGCCCCAAACACCTGACTCTTACCTTCAGTGGCTTTG

>103879

TGCAGGAAACGCAGAGAAAGCAAGAGATTCTCAGATTGTTCCTACAATGAAAAAGCAGGACGTTTGCTGGAAGTCTGGAAAAGCCAGAAAGAAACA

>148561

TGCAGGTCTGTTATTACCGCTGGATCCGTGTCCACGCCGTTCCTGGAACACTGCGGCGTATAGGAGACGGTTCTGAGCTGATCCTCGAGACACACT

>290830

TGCAGGATTGAGCACAACATGGAGCAAAGACCAGGAGATGAATAACGGGTTGCAACAGAAGGAGGCAGTAAGAAAATGGAGAATGAAAGCTAAATA

>149380

TGCAGTGCCTTCTGGTCTCCCACCGCCTCTGGCACCAGCTTGCCGTAGGAATGCTGCGGACTGGTATCAAACCTCTGCGTGTCGAAGTAGACGGAC

>234113

TGCAGAAAAACGGCCGTTATGAGCACCAGAGTCAAAGGAAGTCCAATCCCGATGGTGATCACATTTATCCAGTCTAAGAATCCACCGTTCTCATCA

>68014

TGCAGGCCAGTCCCATTTCCCTGATCAGCACATAAACAAACCAAAACGGCGTCTTTGTGTGTAGAAGCATGAGAGCATTTCCTGCCTCCTCCGGCT

>97679

TGCAGCTATCGGAGTCGAACAGAGCTGGGAGCCCGGGAGGGGGAAGTGCGCTATCTGCGTCCCCATGTGCACCAACATGATCCGATACGATCTATA

>120685

TGCAGGAGGACAGGCCCAGCAGGGGCCTGGTTTGGAGCTGAAATGGGTTTTCAGTGGTCAGGCTGTTGTGAGTGGTGGTAGTTCTGTGGGTGCCAC

>197068

TGCAGATGTTTGATTTCCTTCCTTAGAGCATTTTATTGTTTTATGGAACAAAATGAGCCTAAAGTGACAAAAGCAGCGCAGCAGGGAAACTGAGAA

>75658

TGCAGACATGTGGTCGTGTCCAATAACAAGCAGGAGTTTGTGTTCACCACTAAGAGACATAACGAAGTGAGCGTCGGCTCCATCGCTTTCAGCCTG

>112616

TGCAGGTACCTGGAAAACACCAGAATGTTGCATTTCTTTCAACACAGAAAAGAGAATTACCGTAGTTTAGTTTTGCACAGCCAGAAAATCAGCTTG

>30053

TGCAGATCAAAGACCAGCAGACGTTCAGTAACAAGAACCCGATCCGCATCTGTTCAGCGGCTCCTTTTTAGTCTGCTGAGCGCACCTGAGGTTCTC

>252838

TGCAGCTCTCTGGGCCTATCACCAACATGCTGCCCATGCTCTCCTGCCTCCACCGCGTGGAATACGTGAGGCAGGGTGAGGGATGTGTGAGGGAGG

>322426

TGCAGAATGAACATTGCATTATCAGAGGCAGACATGTTGTTTGGTCATGGTTCTCAAACATTTCAGAACAAGTCCCACCAGATACCGCTTCCAGTC

>130521

TGCAGGTTTTGATGTGGGAGCGATGGAGACAGCTGAAGCAAAGACAACAGGCTGAGGACAGCCGGGTGACTGACGGAGAGGAGAGGAGACAGAGGA

>198271

TGCAGTTTTCAGCCAAATGCCAAGAACAGCAAGTGTGTGCCACCAAATCAGAGCATGTACCTATCGCAGTCAGGCAGACCGCCAGGCCAGTTTTAA

>134273

TGCAGGTCAGACTGTTTGCTGCTTCGCTCCTCATGGCATCTTCATCTCAGCTGCTGCTGCTTTGGCTTCTGCTGTTAGTCGTTTCTTCTGTGTCCA

>158928

TGCAGTAAATCTGATTGCTATGGTTCTTCCTTCATTTCACGCAATAACTATTGTGTTACATTCACTTTGCAACTACAATCAAACACCCTGTAAGGC

>223759

TGCAGGGCAGTCTCTGCAACGTCTGATCTTTATGATATGCACACTTCAGACTGTCAAGCAAATCTGAAGGTACCACCAGCATCAGCTCATGTTACC

>275410

TGCAGGCAAAAGAAAGACTGAACAACTGAACAATGACTGAAGACTAACAAATGTCAAAAGCTGATTAGTGAAAGTACAGTCATTCAATTCATAATC

>303317

TGCAGATTTACTTGATTTATCAAAAACACACAAAGAGCGCAACAGAGGCTGAGAGAAGAAAGGGTGAAAAATCATTTGAAATCTGCTGAATGGGCC

>6153

TGCAGCGCTGACCTGCCCAGCCCAGCCCAACCCGAACCCGGCCCGCTTTCTGCACTTGCCCTATATTTACCATGTGGATCTCTACTTGCAATACAC

>37421

TGCAGAGCAACACGCTGCTCCACTAGCCCCACCTTCAGCACAACGACAGAACGACAGACTCACTCTTTGGAGTAGGTGAGGACGTAGGCGGCCTGC

>86820

TGCAGAACAAACCCATGTGTGAGGAGTCGGAAGAAGCGAAGCAGGAAGAGGAGACGAGCTCAAAGGAGAGAGAGCTTCTCCAGCGCCTCAAAGTCC

>94062

TGCAGAACCTGGACCCAGATTGGACCCGAGGGAAAAACGGTTCTGATTCTCAGGTCTAGAACTACTGGAGGATCAGTAGAACCAGAACCACAGCTG

>158551

TGCAGGGTCACAAACATCACTCACATTTTCTGAAGAAGTCTGAAACACAAACAGCAGAATAAAAGATTTTTTTTCCCTGCCATTGTGACAGGGCTC

>47760

TGCAGGTACCTGGATAACGTTGTGAACAAGCAGACGGTGGCTCCGCCCACTCCCCACCTGCACGCCCTGCTGACCAGCGGAGACGACCCTCCGCCG

>242760

TGCAGGTTCATCCAGTGACAATAAATAATCAGTTACAATCAATCAATGAATCAAGGAACACGACCACAGAAGAAGAGTCACCACAGAACATGGCGG

>152454

TGCAGAGAGCCGTAAGACACAAACATGATGATGTCATCTCACCTGTCCCACTGATGATGATAAAGCGCCGTCTGCTCCTCCAGGGGCGTCGGCTTC

>12947

TGCAGCAGAAACGTCAGAACAACAGGAAGTTCGAGGTAAAACGATGGCGCAGCAAATCCTCAAAAACGGTCTATTTTCAGGAATTGTCACGACTGG

>9720

TGCAGAAAGCAGAAACCACAGAAGAAGTAGATGCTCCAGAGACTCCAAGACCAGAGAAGAAGAAAACCAGGAAGGGGAAGAAAGGCGGAGCCAGTC

>198527

TGCAGCTGTCATTATCAACAGCGCAGCTAGCTCGGTTAGCTTCAGCTAGCTTATGCTACATTCAGAGTGACTCCGACATTATCGTATCTCTTTATC

>110350

TGCAGCAGGAGTGTCCCTGCTGCCAGAAGGCCAGGTGTTACCCTTGATAAGATAAATCAAAATGATTCAGGGCGGTCTCCGTGGCATATAAATCTG

>244069

TGCAGCAGAGGAACACGTTACGTCTCCGTTTACTGAATATTTCTTCCTGAAGTTCGTATCATGAAGCTCCAAAGTAAAGCAGAGAACATTTCCTGA

>86678

TGCAGTTCCTCTAGCGGCCACCAGGGGCTTCCAGGCGTTTGGGCCTCCTCAGGTCAGCAACAAACATTTTGATGGTTGTTTCACTGACATCATCGT

>135774

TGCAGCAGCTTGTCACACCCACACAGACGCGTCAATACGGCCTGAAGCAGAAAAACTCAGGCTCATGTCCACTAAGATAAATAACTATTCATCTAT

>85103

TGCAGGTGTGTTGATGCCAAAGGACGTCTGTTTGGGGACTTTGGAGTTGATTACCCGAAAGAGCAGGTGACAGGAATCAGCAGAGGACTGAACGCT

>296452

TGCAGAGGTTTTCAACATGAGGGTCGTGGCCCTCGGGGTGGCTGTGAAAGTACTGGAGTGGGGTCATGAAATTTATATCATTTCACAGATTTAAGA

>288045

TGCAGACTCACTGCTACTACCACGGCAGCATCCGGGGATTCCCGCGCTCCAGAGCGGCTCTGAGCACCTGCTCCGGACTCCGGTCAGTACCGCCCA

>114996

TGCAGACGCTGTCAGCAACAATGAATACCTATCAGCCCTTTTGCTCTGATTCCAGCTGAGCGTCATGTCGCTTTGATTTCATTCAGCCAGCTGAGT

>90591

TGCAGTGCAGGCAGGCCGAGCGCACAGTGGACGACCTGACCATGGAGCTGCACAGCACCAACCAGGCCAAGGACGACCTGGCCAAGCAGCTCAAGG

>301384

TGCAGCTAAGAAAACGATCATTTCTTTGATTTTATAAATGGAGTTGTATTCTTGTTGCCCTGGTCTGCTTGTTGGATACATTCAGTGTGGATGTTT

>242480

TGCAGCTGAGCACCTCCACCCTCACCTGTCCGTATCTGAACACCTCCACCTTCAGGATTTCCAAAATACACATTACAGGTCAAAACAGAGCCAGGA

>64514

TGCAGTGTGAACGCAGCCTTAGAGTGGGATTGTGGGAAAAGACTGAACAGATTGAGTGAGTTTAGTTTAGAAAATTGGCGAGTCAGACCAAACAAA

>289993

TGCAGGTCAAATAGACAGCTGGTTTATAACCTCACCATGGCCCTTTGTCTTGTGCAAAATAGTCCTGGTTTGCTCCCAACCTGATCCAGTGGGGTG

>305422

TGCAGGAGCAGAAGAACGTTTGGAAAGACGAGGACCAAACTCGTCTTGCAACGTTTAGATTTGACTTTGCAGTAAAACACCTGAGGAGGTCATTAA

>144928

TGCAGATGGACTTCATCCAAGCAGAAGGCTAATGTGCACATAACTATTTGCATGTGACAGACGCAGACAGAGCATGTCGGGATGTGTGCAGGAGTA

>30921

TGCAGTGCAGCAGCAGCTGGTTATGGATGTGTCCCTGAACTTTACTGTGACGGAACAAACTGCGTGTTTTGCAGTAATGTCACTTCCAGGTGCAGA

>81476

TGCAGGGTCACCTCCTCCTCGCCTCCTCATCTCTCCTCACGTTTCAGTTTCTCCTGCTCGTCTCCAAACTCTGGTTTCCTGGATCGATTCTCGTTT

>274624

TGCAGTCACCACTATTTCTTTCCTCTCATCTTGTAGTGCCTCCTTCTATCGCAAGCAGTCGCACCAATGTGACGGTGATTGTCAACGTTCAGACCA

>326023

TGCAGAGGACAAACTGGACCACCAGCCTTCTGCTCTTCAGTCCAGTAAAAACATCAGTAACTCTTCATCAGCTCCTCCCCTGGGTGTGCAGTCCTA

>184452

TGCAGGCCTTTGGCACAGCAGGAGTTCTTCACCAAGCCAAAGTTCAGCCATCTTATTGACCCTGAGGACTGTCTCTCTGCTCCCTGTGATGTCATC

>188197

TGCAGCCTGACAAAGTCCTCACTGAGCTCCAGTCCAATGAGGCGAGAGGCTGAACTGTACACACAACCCTGAACACATGATATGTTCATCACAATA

>198777

TGCAGCGGCTAGCAGATCCTCACCGTAAAGATTTTTCTTGTCAGTCAGAAGAAGCTAATTCTGTCTCAGTAAGTCAAAAATGATCCAATGAGCTCC

>161111

TGCAGCTCATAGGTAGCAGCGTTCAATGGGAAACACAGAAATCGGAAGATTATCCTGTTTTATGTGTCAAACCTTTTATCAGTTCTGTCTGAAAAT

>39338

TGCAGCTGGAGCTGGAGGAAAGCGACTCCATGTTTGATCACATGACCTTTGGCCCGACCCGGAGGATGATCTGGAACCTGACGGAGAAACCGTTTT

>71545

TGCAGATTCTGAATTTGGTATGTAGTCCATATCAGACTCATGGTCATCATCTGACATTTCACATTGTTTGGAAGGTGATGTGTTGTTGTCGTCATC

>202348

TGCAGCAGGAGAGGGATTCTGCACGAGCAAATTCAAGTCAACAAGATGATGAACAAGCTGGCCCGAGTACAAGTGGAGCTCCGATGAATACTGGGA

>84995

TGCAGAGAACGTTCACATCAGAAAGGAAACGGGAAGGGTTTCACTGGTGCTGCACCTCCGTTGCAAGCGTTTGCGAAACTTTGTCGACATTCGGGA

>188979

TGCAGGAAGTCCAGCTGTTTGACTCGGACACTTTCTGCTCCATCAGGCCGGGACGCTCCAGGCGTGGAGGGTTTGGAAGCTGATCATAAGTCATTT

>3106

TGCAGCCACGCCTCGGCTCCCTGAACGCATCGCCTGCTGCTTTCGCAGAAAGAAGCTGAGCTACTGAGAAAACATCCGACCCGTTTACTTCCAGAG

>179140

TGCAGGATGGATGAAGAGAGAAGTCAGTGTCAGAATACATCAAGCACCATTAGCTTCATTACTCTGTTGGTCTGCATACAAACCCAGCAGGAGCAG

>185161

TGCAGCAGCTTCTGGATCAGCTGCAGCAGCTTCTGGATCAGCTGCAGCAGCTTCTGGATCAGCTGCAGCAGCTTCTGGATCAGCTGCAGCAGCTTC

>299404

TGCAGCAGGAATGAGAATCGGACACTGAGGAATGCACATGTGCTCAGATGCACATCAAGCAGAAGGATTTAGAGTCGGATCATAAAGATAAGAGCA

>122036

TGCAGGCACACATTCAGTGCTCTGGTTCCCCTTCCTCCTCATCATCCTCACATCTCCAAAGCAACGCAGGTCACGCACAACATCACCTCAGCCTTC

>200818

TGCAGCTGAAACTGTGGAGTGGAATGTCAAACTTGTTCTCTGCTCTGTGATGTTTTAGGTTGTTTTTTGTTGTTTTTGGTGTGGTTTGCGTTCCTC

>334367

TGCAGCATCACTTCCTTCCCAATCAAGCCCATGTGCCTCTTTTGACTGAGTTGCGTAATTTGGACGGATTTGTCTTTGTTTAGTTGTTTTAAGATC

>128611

TGCAGCAATATAATGATCTAAACAAAGCACATCTGTAGCTCCGCCTCTGACTGGCTCAAAAAGTGGGGTCAGGCTGGCCTAGTGTTTCCCCACCTT

>277903

TGCAGTTTAGCAATCCAGGGGCAGATATCACATCCGGTCAGAGACTTCAGCATCAACTTCCCTCTTCTTGTGAAAGTTTTACGAGCACCAACCAAA

>163926

TGCAGCGCAGAAGCTTCGAGGTTTTCGACATGACCATGTACAAAGGCAGGCGTCGCAACCAGAGATGTGAGTTGTTGTTGTTTCTAATTAGTGAAT

>139486

TGCAGCTTCTACATGGGCGACAAATAAAAGTATTTGCTGCGTTTCGGGTTTATGTTTTGTCAGAACAGTGCGAAAATCAATCAGCTCCTGCGGGGG

>231708

TGCAGACGTCAGCACTGTTACTAGTTCAGATGTTCATGGTGTTCTGACAACTGTGGAGGGTTTTTATTCCTGCTGTTTTTTATGCCCCAATCCCTC

>124483

TGCAGATTGTTTCAACGTTCACCTCAAGCACAACATCAAAGATCTCATCACAACCTGCCAACGACCCGACAGCGAGACTCCAAACTTGCAGCGATT

>201913

TGCAGGGGCAGAGAGTTGCGTTTCCGCATGCTTGTATCATGCCATGTGTCCTGCTGAATGACAGGCTTCAGAGAGTTGCATAACTAATAGTCTGTG

>172231

TGCAGATCGCTGGAGAAAAACGAGAAAAACGTCAAATTCTCACGTTGAAAAATGTCACAAATGGACGGTGAAGGTGGGAAAAGCATAATTTCACCG

>63720

TGCAGATGCACTGAAGAAGGATCCCCTGCTTTTGGAAGAGTCTTAGACTTTGAAACATAATTACAGGACGTCATGGCCAAAACGCACTCATTCCAG

>68420

TGCAGTGTGTCCAAGCCTGCTCTGTGTGTGTCCGTGTTCTCCACAGACCCGGCGCCGTCTCCGCCGGTCAGGGACATCGGTTTGTTTAAGATCGGA

>269622

TGCAGTGCCAGGCCTCCCTCCAGGCTACAGTGCTGCAAAAGTGACAACATAAGGTCCATTCTGTTCCCTGTCACTCACAGCCTCAGGCCCACTGTT

>214322

TGCAGCTGTCTGAGCTGATGACGGGTCGGGTTCGCCTTCAGTCATGGTCCAGTTTGAAGCTCTGGTTCCGGTGACATAAATAAACTCTGATTAGCA

>255585

TGCAGTGCACTTGTGACTCCCTGCCCACATGCAGGGGGAGATGGGCGGATCGATAGACGGCCCTCGGTGTTCTTCGTGCTGGGCGAGACGGGCGCT

>299716

TGCAGCGTCAGCAGCTCCCCGCCCCGCCTGCGGCACGACACCGCCGCCAAATCAAAGTCCAGTCCGGCCCAGTTCACCGTCAGGCAGCGGCCTTCA

>118880

TGCAGCAGCAGCAACAGCATATGCAGCAACACCAAATGCAACAGCACCAAATACAGCAACAGCAAATGCAGCAAATACAGCAACAGTATCACCAGC

>139941

TGCAGGCAGAACTTCTGAAGAACCTGGTGAGGACTGTAAGGACAAGGATGGAACAGTTCTGCTTCAGCAGCTTCGGTGCTTCCTCTTCTCTAATGA

>308138

TGCAGAAGCTGGCGGCCGTAATCGTAAGTGCTCTGCGAAGGAAAAAAAAGAAATAAAGTTTGAAAAGCAGAGCCTGTTACAAATCAGTGACAAAAC

>170771

TGCAGCCGGAGCAAGGGTGGAGCTGCTTTCTTATGCAGACTCCCACCACCACCTCCTCCTCTTCTCCTCTTCCTCAGGCCCTCCTCCTCTACCATG

>119090

TGCAGTACGATGACCTTCTTGGATCTTGTGTGAGGTACCTAAGTCAAGGAGCCAACAGTTTCACCTGCGCAGCTGGAAGTGGAAGATTTGAGGTCA

>26030

TGCAGCAGACCTGCCGCAGGAAAACCTGGCATAATACGCACAGGAAGGAAGAAATCACCAGAAAAGCCCCAACACCACGCTCTGTTAGAAAGACAA

>63588

TGCAGGGAGGTAAAATAAGTAGCTCCATAATAAGCATCACTTCAGAATACTGTAAATTGTCCTGGTAGACGGCAGTGATGACCTTGGACTTGATCA

>213927

TGCAGGATCACATCCAGCAGATGTTCTGGATCCACATGCCGGGTTTTCCAGTCTGGGATCCGATCCGCTGCTGGACCCGGGTCAGAACTTTAAGAT

>135923

TGCAGTCTGGGGGTGCAAACAGGAGGTAAGGCACCAATGTAGCTATCTGCAAACAGGAAGCAGAACAAGCCCAAGGCAGAGGCGCCTGTGTAGGCG

>130805

TGCAGAAGGTAACAGATTGTTGCACTGTGGCGCCATCTGCTGGCTGCGGACTGTCAGCGTTTCAGATTCATCTACCAATCACTGAACAGATGCAGT

>222380

TGCAGGCTGGCCTGAAAGGCGGAGCTCATCAGCTGGTCATGTGGTCCGCTGCGCTGGAGGATGCTCAGGTGGACGTAGAACCAGAACACATAGAGC

>212806

TGCAGGAAACACCAGAGTTAGAGCGGAAACGTTTCACCAACACGTCGAGGAAAAGCTGCGTCCACCTCGTTCACGTCCAGAACGTAGTGGTTCTGG

>150541

TGCAGTGCTTCCATCAATATCTCTCTGTCAGTCCAGCGGTAACAATGGTGTGCAGTAACGGGAGAGTGTGAGAATTACCTGTCAGTCTGTCTGTTT

>151684

TGCAGGTGCTGCTGGTTGATGTGCAGCAGGATGCGGCTGTAGCAGCAGCTCATCAGCAGCAGAGGGATGACGTACAGCGTGACGAAGTGGAACATG

>65306

TGCAGGGAGACAAGGCAGAAGAGGAAGAAGCTTCTGTCACGCCGATACAGCAAGCAGAGGCACTGAATAAGAAAGCGCCAGGTGTCCTTTTGACAT

>66693

TGCAGCCTTCTGGAACTCCAGTGTGGGGAGGTTTACAACGTCAGTGTTGCAGGAGCTGATAACAACTGTATAGGACTACAGAGCAACACACTGTCT

>219706

TGCAGAACAGGTCGCCTCGGATGCAACCGCAGCGGGAAAGCCGCCGGCGCCCCCTACCGGTCGCAGTACAGCTGCATGTGACGGGGCATCTCCCCG

>26319

TGCAGGGACAGAGAGGAGGAAGAGATGGAAGCAGGGAGGCAGAGGGAAAAACAGGAATAATAACGATGGAGAGCCTCCAAGACGCACTGGAGTGAG

>230401

TGCAGCTGGCTGTTTGCCCAGAGCATCTGCTAAGCATAAATGTATTTTTGTTTTTTAGGGAATAAAAAGAATTCAGAAATTGAGCCCATCAGCAAC

>70154

TGCAGCATTTTCCACTCACTGCTGCCACTACAGACTTCTTCAGACATCTAGCTAAAACTGCTTCGCAGCATAAAAGTGCAAAACAAATGGAGGCAT

>76673

TGCAGGCTATGGCCCTGCTAATAGCAGTGAGTGCAGTGGAAGCGGAGGACAGCACATGAACAGCTTGCAGTAGTGGCTGTTGTGTTGTTTGTTGTT

>141543

TGCAGAGAGCATCTGAGGACAGATGCAGCCTTGTTGTTATAGTGACAGAGAGAGAGAGGAGAGAATAGGATTATCAATTACAAAATGGAGAAACTT

>282427

TGCAGTTCTGGTTGTATTGATGACTGACGTATTCTGTTGACGCACACATTGACAGAGATGGCAGCTGTGTTTGGTTTTCCTCCGTAGCACTAATTG

>299385

TGCAGCTCGGACTGGCCAGTCAGCAGAATGTTATGAATGGGCACAATGAAAAAAAAACCCAAAGTCCATGCAGTCAACCCAAGAATGCTTGCCTCC

>16497

TGCAGGTTGAGCTCACTAAAGCAAGAGTGTTTGTATCTGAGGCGTCGTTCAACAATAACTAAGGTAGTTTATAGAGATGGAAACATGCCTGTCCAG

>10520

TGCAGAAGCCGCTGTAATGAAAACTGCGTATATCTTCCACAGCAAATAAAAAAACGACTCATGCAGACTAAACAAGAGGTCTACGCCGCGGACATC

>336635

TGCAGGAAGAAGGATAAAAAATGAGTATGAGTCAAATATCTGTGTGGTAAATATGAGGTTACGGCTGGCAGTCAGCACTAGTCTAGCTTTGGAGAC

>230780

TGCAGCTTCTGGACCAAACAGATCTGTTGGTTCTGTCCGTCCGGACCCGGACCGACCGGAACCAGAGTTCCTTCCCTCTACCTGCCGGACAGATGA

>230554

TGCAGTTGTATTTTGATGTCTGCGTTCAGCAATAAACCAGCTCACTGCAAGTCTAATAACACTAAAAAGTTGCTAATTTGGCAACAGCCCCCCCTC

>46506

TGCAGCAGCAGAGAGAGTTGCAGCAGAATTATCACTCAACCGGAGCTGGACTGGGTCAAACCTTTTGGCTCGTACTGTGTAGGAAACCATTGATTT

>20380

TGCAGCTATTTTGCCCAGATCCTGGAGGAGCACGGCCCGCTGGCGGACGAGGACCGCCTGCTGGTGGGCGAACTCGCCAACTTCCCGGCTGAGGCC

>110452

TGCAGAGTCAAATGTCAGCCCTGCTGTCCAAAGACCCCAATGCTGCTAAACCAGAAAATCAGCAGAAGAAACAGAAACCAAAATCTGGCCAAACCA

>2822

TGCAGCCGTGGAAATAGCACGCAGGACGGTGAGTAGGCTTGTGGGGCTTCGCCCTTAGCACGCCGTGCGGCGTGGCTGCACCTCAAGGCTCTATAG

>107536

TGCAGAGCGACGGGCGGAGGAGAGTCACAAGGAGCTGCTTCAGCTCTACACTGACCTCCGACAGCAGGCGTCCGGGCCCCAGCTCAGCGAGGACGA

>269778

TGCAGCTGGAGGGCAGTGCAGATCCTTTTGACTGCTGCTTGTCTGATGGACCAGATGTAAATGTCTAAGGATGTGTGTGCAGCAGCAGCATGCAGA

>257046

TGCAGTAATCAATTCAACAATAATTCGAAGATAAACGACTGGAGGAGGTTCATTGCAGGGTCTGGAGATCAGAAGAAAGGAGCCTCAAGTATTGAG

>21980

TGCAGGGACTCCAGCAGCCAGACGCCCAGCAGCCGACCCAAACCCTGAGAGCAGAGCAGCGCCAGGTTCAGACGGTCCTGCTCCGACAGAGAGCTG

>297448

TGCAGACAGTTGTTTCTCCACTTCGAATTGGGTGCTATGAGTCAATCTGACTATCTCATACAATGTGGTAGTTTTTTTTGTTGTTGTTTTCAAGGT

>160224

TGCAGCCCTCAGAGGTCCGGTGTGGTTGAGCCGAGGGCAGAAACCGCTGCCAGGTCACCCAGAACCAGAACTTCCTGGCGCATTTTCACCGAACCC

>119381

TGCAGCTTTTCAAACTGACCTTACACGGATGTGAGCTGTTAGTTGGTTGAAGTCTTGGGCTCTGAAAAGCCGCCCAGTCCATGGTGAGACCGCCGT

>13433

TGCAGGCTGTGATAAGTTATCATTATGAGATGCAGCCTGTGACACACACACACCTCCATTTGCATGAAGAGGGCTTTTAGCGATGCCCTTGTACCC

>283441

TGCAGCTCCAGCCTCTGATGTGAACATTTCTCTGATTCCTCCTGCTGAGCGGATCGGACCTCCTTCATCCTCTGGTCCATTCCCTTCTTTCTTCTA

>215447

TGCAGGGAAGGCAGACGAGCGGCCAGACCTCTCCTGTTTTACGGGACTAGGTCCTGGTGATGCACTCGCTGGAGGATGGAGGGCTGGATGGCGAAT

>170603

TGCAGAAATAAAATACAGGCACAGCAGAGTGATGTCACAACCAAACATACAAAAACAGAGACTTTTAGCAAACATGTACATCATTATTTACACTGA

>207722

TGCAGAGCTCCACGGTTCAGGAGGACGTTGTATCCCGGGCCGAGACCCGGGCCAAGGAGCTGGAGAGCGCGCTGAGGGCCGAGGAGAGGTGAGCCG

>307290

TGCAGAGAAGAGGTGGAGGGATGCGTCAGCAACAGGAGGTCACACCGACAGAGACTGGATTAGCTCAAATGATCCTCGCTGTCCTCAACAACTCAT

>101285

TGCAGCCTCCAGATCAAACCGCAGAAGAAGTGAATCACTGGATCTGACCGTCTGACGACAGGAAGCAGGCTGACAGAGCCGAAAGCAGGAACTGGA

>99020

TGCAGACAGCGTTGGAGATAAAGTTCCCACTGGCAGAGATGATGATGAAGAGTGTGCCGGGAAGACAAAGGGATCACAGATACAAATTTCTGAGTG

>163806

TGCAGCTTCCACAGCGTTTGTTTTTCTGAGAGACTCAGAGGAAAGATTACAAATAACGTACAGTGAACAAAGTAAAGATTGTAACATTGTCTGAAA

>116825

TGCAGATGATTCTTTGATTTGTCTTCATTGATTCTTGGAAACAGCTGGAGCTCAGTCAGAATGGATGCATAGAGAGCGTCTACCTACAGAAAACCT

>10148

TGCAGGCATTTTTCTTTGCCTAATGCACCAGACAGTGTCTTTGCCTCGCATTCCGGCTTCAGTAATTGGAAAAAGGCGTTGTCCAAGGAGTCTGGA

>157209

TGCAGAGTTTTCTTCCTCAGCCGTGACCGTGGAGCTTTTCAGGATCCATGTTGCCTCGACACGCAGTGGGTCAAAGGTCACGGCTGCTAATCCACC

>27186

TGCAGGATCTTCCTGAGTAAATGCCTGGGAGTTTCTCCATCGTCAAACATCATGGAGGAGGAGGGAGCTTGCTGTCTGAGCACAGCGGAGGCGGTT

>163750

TGCAGCTGGGGTCCCAGACAATTTTTACTGTTAGTCATATTTCAGCTAAACACTGATCTGGGTCAGTGGGGAGAAAGCCAATTACGAGCACACCGA

>288125

TGCAGGCTTCTGCCATTTCTTCAACGCGCCACAGAAACAGGATCGCCAGAGCGACCAAACAGCTGTTCATTGTGCAGCTCATCTTTAAGATCGGAA

>80062

TGCAGACTCTGCCTGGACCTGCCGGAGTTTGACCTCTCTGGTGCCGACGCAGCAGCAGGAAATCAGGCGTCTGCATTTTGCTCTGCTGCTGCTGCG

>93361

TGCAGGATGACCAAAGGTCAAAGGTCATCCTCAGGGATGTTATCAGTTGTTCTCAGAAGGAAGAACATCCAGTTATGAGGGTCAGGGTGTGAATAC

>16629

TGCAGCCCAGCGATCAGAACCAGCAGGACCAGCAGGCCCACTGGCAGGACGACGTTGGGTCTCAGGCTGACTGCTCCTTGGCTGATCTTGTGGGGT

>275479

TGCAGGGTCTGGGCATGCTCAGTTCGACCGACCGCAGTGCTCTGAAGCGCCGCATCAAAGACGTCCAAACTGCGGCGGAGAAAGAGAGGAAAGCTC

>114113

TGCAGGACGGGACGGGCCGGACGGAACCGGGCCAGATCAACATGACTGACTTCCTGCTGCCCCAGAACCAGCAGTCGGGTCGGCTGCACCCACCTG

>151829

TGCAGTGGCGGATTACTCTGTGACCAGAAACAATGTGATCCAGCTATGTCTCGAACTCACCACGATCGTGCAGCAGGTGAATGAACTGTGCAACAT

>24822

TGCAGCTGCCTTATCTTCATCTTTGGCAGCATCAGCTGGCTCTGCCCAGTCAGAGTTTAGTGCAATGCCCACTTTCCCTCCCTGTGTCTTTCTGTA

>253397

TGCAGTCTCACTGCATTTGAAACTCCTTCAAAAGGCAGACACATCATCGGTTCTCACTGTTTTGTAATATGTAAACATACGACATACGGCCAAGAG

>166507

TGCAGCTCAAAGAGCCGAGTTTGTGTTCGTGTGCTGACTCTGCTGGATCAGTTGCACTGAACCTGAATGGCTGGAATGTGACCTCACACACACACA

>50209

TGCAGATTCAAAACATGGTGTGTATGAACTGATATGTTGTCGGTCCGTAAACCTGGACAGACAGCACAAAGAGACGCCTTCTAAAAACACACAGCT

>157910

TGCAGTGTGAACGCAGCCGTAAAGTCTGAAGAGGTTCTGCTCTCGGCTGCAAAGCTGCTCTTCCATTCAGCGTTCAGAGGAAACGCCGTGTGACGC

>243213

TGCAGCGGAAGGGAGGAGGGGAACCTTCAGCTGTTGCAGCAGTTCAGGAACCGTCGGGAACCGGCTGGGCCAAACGGACCCAGTAGTCCCGGGGTC

>146870

TGCAGGTACGAGTCCAACGGGTCCGTCTACTTCGACACGCAGAGGTTTGATACCAGTCCGCAGCATTCCTACGGCAAGCTGGTGCCAGAGGCGGTG

>134367

TGCAGGGGGCGCTGCGGGGCTCAGCCAATCAGAGACTCAGGGCCCACAGGAACTCCCGCCCATCTCTTTCCTGATTGGCCGTTACTCTACATGATC

>72090

TGCAGGAACACAGACAGTTCTTTAGTTTGTCTGTCTTGTGACTCTTCCTGCTGTGTGACTGAGCCAGCAGAGTCCTCTGTGGTGAGCGGCATCTAA

>198794

TGCAGCAGGTTTGCTGGCTGGTTTCTTGGCTGCTTCATCCTCTGATGACTCTGAGCTGTCAGACTCAGAGCTGCTCTTTGTTTTACCAGTTTTTGC

>325317

TGCAGCCAGACTCCATTTGGGAGGAAAAGGCCATGAAAAACACAGCAGACAGGTGCGCTGATATATGGAAATGTTGTCCTCTTTTATTATGCGATA

>196338

TGCAGACCCTCATCTTGACTAACATAAGACGCGTTTGCCCCCAAACAGAACAGTTCTTTGAAACATCTGACCTTTAGTTTTCCTTTTCCTCTATGC

>24283

TGCAGTAACAGTAGTGTGTAGGTGTGCGAGTGCGCGTAACATTATTCATGATCGGGTACTGAATGCGCAGAGATGAGTGGTGTTTGCAGAGGTGTG

>94890

TGCAGTGAGAGGGTAGTTTCGTCAGCTTACCCAGGTTTTTACTGGTATCCGCAAAGAGTTAGCTGAACCAAAAGACAAGTTGTTGTCTATTGTGGA

>214040

TGCAGTGCCACGCCTCCCAGCCGCTGGTGGCGTCCTGCGGCCTGGACCGCTTCCTGCGGGTCCACAGCCTGGAGGACCGGCGGCCGCTGCATCGCG

>270223

TGCAGCAGCCTCAGTACCGCAGCCTGGTGAGTCATGCTGCCAACGTCGGCGTTGCAACTCCAGCCAGACGTTGCTGCGTTTTCCCATTAAGATCGG

>54752

TGCAGGAGTAGAAAAACCCGGAGAAGTATGGCTCACGACAGTTTGTAGAGACAATTTAGAGAATTTCAACTGGGAAGGCTCCAGCATGCTACTAGT

>288859

TGCAGTTCAGCTTGATGAACTCCTAGGGCCGCTGTTGGCTAGTCTGTGTTTTCAAAGGAACGCAATATTCAGTAACTCCCTTCTCAACGCAGGCAC

>73505

TGCAGAGTCGAAGGTTTCTCCTTCTTTCAAAGTGACGGCCGCCATGCCGATCCGACCCTCGTGACCTGATGGTTAGGAACAAGCAGGGGAGAGGAA

>23927

TGCAGCAGCCGCTCACTGTGGGGCCGTCACCGAACTCGCTTCAAGTCTCATCAGCAAACTTTAGATTTGATTAGCTTAGCAAGCAGCGGCTCCCGG

>126384

TGCAGGAAGTTGAGTCTGTGAGAGCAGAATGTTATCAGCTGTTATCGCCTCGCTGCCGCGGCGCTCGGGTATAAAACGGCCGACTGAGAGGAGGGG

>129143

TGCAGGATCTTGTGTTACCTGCATACGTCCTGTAAGGTGGAAAAACTTTGACCTGAAGGACGTTCAGTTGGTGGATTTGAACTTCCCATCATCCAA

>53949

TGCAGAGAGTTCACAGGCTGCGCTCACATGTCTCTGCCGAGTTGCTCGCCGTAGCAGCATCACTGGCTGCCAAGACACACACACACACACGCAGAG

>81883

TGCAGTCTCTGCGCGGAGGGCTCTCCGGGGGTCCCAGGTCAGCGCCCTCCACCGTGTCACTGGTCAGATCCAAGGAAGAGGCCACTGGAGGATGGG

>202629

TGCAGCTTCTGTCTTCCAGTTACCTTCACAAAGACACCATTGTTGCAGGTTTTTGATGAATGCCAACCGAAAACACTCCCAACTGCATTTCTTTGT

>114121

TGCAGCCTTGTCTCCTGGCTGCACGGACTGAGCTCAGTTTCTAAGTGCATATTGCCTGGCCTGATCACACCGATTCTGATTCTTTCACTGAATGAA

>125210

TGCAGTACAGAACAGTGTCATCAGCAAACATGTGAAATTTACAGCCTGTTACGGATGAAGCGATGTCTGCCATGTAGATGTTGAACAAAATAGGAC

>56226

TGCAGGTGAGCGCACGCTGACGTCTCTAATCCAGGGACTGCATGTCGGCGGTGTGACGCCTCACTGCGAGGTAACAAGATCACAAACTGGGCTTTT

>99114

TGCAGCTAATTATCCTTAGCTCTCTCATTCCCCAGCTTTGTAGAATGTCTCCTAAATTACAAGCACACAAAACCAAATGGGATATTTTATTGAAGG

>219495

TGCAGCCACTTTCTGACAGAAAAGTACTTCCAGTTTTATTTCAGGCATCATCAGTGTGAAGTTCTGCTGCTCTGGATGGGGAACCAGAACATCTGC

>11906

TGCAGATGTCCCAGTAATCCCTGCTTCTTCCCTCACAACTTATCCTTCCTTGAAGCCAGAGCTAAGGCAATGGAGAGCTGGAGAACTGCATTGTTA

>200607

TGCAGCCTTGCAGCCCGGCGTTCTGCCCCGCCAGGCGGTGCGGCCCTGGACCAAAGACCTGAGTCTGTACGAAGAATACAAACTGCGGCGGAACGA

>68074

TGCAGAGACTCACAAAAAGAAGCTTAGCAGGGGAGGAAATCAGTCTATTACCTGAATATGCATGCACACAAGAAGAAACAGGCGCTGAAAGAAAAA

>64988

TGCAGAGGGGAGGCTGGTGGAGGGGCTGAGGTCTCCAGCTTGAACCCGTTGGATCGTATCAAAGCCTTTTTCTGCTGCTTCAAGGCCCTGTCTCTC

>136290

TGCAGTAATGGCAGCCGTGATGGTGCTGGTCATGACGGCCATTTTCTTCTGCCTCCAGGCAGCAAAACTTCTCAATGCTTCATGGTATCAAACTGA

>248328

TGCAGGTGCTGCGGCTGGGCTGGTGCAGGGAGGTCAGCGACTGGGGCCTGCTGGGCCTGGCGCAGAAGAGCCGAAGTGAGCCGCACAACGGGACGG

>131692

TGCAGACCTGCCCGGGCTGGTCCCGAACCGACCCGAACTCAAAGCCTCCCCCCTTCTCCACTCGTTGCCAGGGGCAACGGGAATTTTCCACTGCCG

>54242

TGCAGACTTGAGGCCTTTTCAGGTGCTTCACATCTGCACTCGTTAGACCTAACTGATGTCACTGAGTCACCAAATCATGAGCAACAATATTTTAAG

>4021

TGCAGTTTGGCTCATCACTGTCCCACAAACCGACACGCCGTCTTCATTTCACGTTTTATTTCAAACTCAAGTTTGAACGCGAACGTTCTGGACATT

>18807

TGCAGAGGAGTGTTGGTTTCATCAACAGATGGAGACAGAGAGGGGCTGTCACACACTCTTTCCTAAAAACACAGGAAGAGGAGCGTCACACACCAA

>169605

TGCAGATGGCGACCCTGCACTCCTCTCTGGCTCTGCACGGGGAGGAGAGAGGTGAAGCGGGGTGCAAGTGGCAGAGCGCTAGCTCAGTGTCAAGGT

>261218

TGCAGCTGCGTCTCACCTGAGCGAACGGCGCCTCGGCTGTCCCTGACCTGCCTGCCGCTGGAGTCCCTGTGTCCTGCGGAGTTACACGGATCAGAC

>111992

TGCAGGATGGCGAACATTCCCTGGCTCTAGGCTGAAGCCTGGCTGAGTGGAAAGACTCGGACTGGCATTGTGTTTTGATGCAGGTTCATTACGATC

>266901

TGCAGATTCAGGTTTTCCAATAAAACATTATCAGCAGAAAACGGGAGGAAGCTGAAACTGCTTCTAAGCTAGCACGCTATCGCACAGCTAGCTGCT

>174560

TGCAGAACAGATGAATCATCTGTCACTGCTAAATGAGAAAAGCGTAATACATACTGGGCCTTTTTGAAGCTGGAGAAAACGATTCGTCTTGGCAGC

>37295

TGCAGCAAAACGGCTGCAATTTCTTCTTGTCTGTCCTGAGCTACGGCAGACACAACTTTGCCCATCCCACTCACACTGCAAAGATTGGAATAAATC

>120574

TGCAGACGGAGAAGGAGGCTCTGTACAACGACAGCAGGTCAGAACCGGAGCGGTGCCTGATGGGAGAATAAGCTCTGGATCGGTGACATCACTTCC

>25517

TGCAGCAAAACCTCTGCTCAGGTTTCCTCAGTTCAATCCGTCAAAGGTCAATCTGAGGTCAGGCTGACAGGCTGTCCCTTCTGGCACCGGTCTGGC

>49923

TGCAGGGAAAACGATGAAATAATGTTTTTCATTCTGAGGTGAAATTTGTGACACGTCGTGGAAAGAAAAGCCCAGGTGTGAATAATGAAGTCGCTG

>209927

TGCAGCGCCGCCGTGACGAACATCGCAACACGAACCGAACCTCCAGCAGCACAGATGGCGGCAGCACAAAGAAAACCACAAACTCACCCAAAAACC

>234225

TGCAGAGCGGCACCACAGGATGGAGCAGATCAGTAAGTCTCAGAGCAGCCAGCCCTCTGCCTTCATGAACCAACTGATGAAGCTTTCCAACAAGAG

>79762

TGCAGAGGGCTGAGAGTGCAGTAATAGTCCTGGATTATTTTAGGAGGAAGGTCTTGGAGCACATCCTCCTTCATCCTCCTCAGAAGAAACGGCAGG

>53622

TGCAGCTTCCATGAGAAGATGCAGGATTACAGGATATAATGGTGTAGAGATGATGGTGCAGATTCATGATTTGCTTGGTTTCAGCTGAGAAGTAAT

>289876

TGCAGGCAAACATGGGAGTATAAAATTAGCTGGACTATGAGAGCTAGACAGGCAGACGTAGAGTTCGTCACTGAGGCAGAACACAAAAGGATACAG

>237173

TGCAGAGGGAAACGGGCATCACCAGCCACGTGAAATGTGTTTCTGCTTTTCACATCTAAACAGTGCTGTACATATTTTTGATTCTAGTTTCCTCTT

>82832

TGCAGGCGAGGCCGTGGCCTCTTCTTCTCCTGTGTAAATAGGTGAGGGGATTATTTGGGCCACTCAGGTGTGTGTCTGTGGCCGGACCTGTCTGTC

>217592

TGCAGTGGAGATGAAAGTTCCCATCCATGTTCTCCTGCCCCTCCCCCACCTACTTCTGCTTTTCTCTGACATTTGGCCGGACAAAGGAGAGACACA

>66929

TGCAGCGCTTCGCCACACCCGTCTGCTTACCGTCAGCCTTCATTACGCCGCAGACGTTCACGTCTCTGTTCAGCCCGAACGCACGCGACACGACAG

>12306

TGCAGCAGTTACGTGAAAAGTTCTGGATAGAGATGGGTATGCATGCATCCGCCACAGCCTGATGCATCACACCTGCCTGACCTCCTGCACTGCATT

>212340

TGCAGGACCCACTCAGCAGCTGGAGAGCAAAATAAATGACTTCAAGCACAAACAGACGTCCTGGAACATGCCCGAGTGAGCAAACCGATTCTTTTT

>78176

TGCAGGAGTCGCCGGACGCCGTGCCGCACGGGGAGATGCCCCGTCACATGCAGCTGTACTGCGACCGGTAGGGGGCGCCGGCGGCTTTCCCGCTGC

>175643

TGCAGAATCTAAGCTCCTGGATATTTGCAAGCTGTTCCCCTGGGTTGATAATATGTCCAGTCCCTTCGCTCCCTCCGCCTCTCCCACTGTTCTTTG

>237826

TGCAGCCGGCTGAGCTGTCTTCTTAGAAACATGGAATAAACTTGTTCGGGGTGCAACTTATGGGTAAACAGCTCTGCCATCTGAAGAACTATTTAC

>254650

TGCAGATGACCTTATGACCTCTGACTCGGCGGTGAGCAAAGCTCTGACTCGCCCTTCCTGCTGCGTCACTTCTGATGGTTTAGAAGTGAGTAGGAG

>207270

TGCAGGAGCAGCTGGAGGAGATGCTGGAGGGACCTCGCAGGACCAGCGAGGAGAAAGAGGCGGAGGAAGACTCTGCTGACCTGCTGCCCATCGTGA

>70813

TGCAGCCTGTCCAGAGCACAATGCCAAGTCTACATCCAGCCTTGAAAGCAAATATTTACCAACCGCAATACCAAATGAGACATCCTGCCATCTGAA

>253074

TGCAGCTCATTCAAAGCTTTGAAGGTTCCCCCTGCCCCCTCCTCTGGTATTCGTCCCTCCAGCGACTTTTCCCCCTGATGGCCTTGTAAAACCTGA

>5725

TGCAGAAAGGCTCCTCTTCTTGTTACTGACTCCATACATTTATATATTTCCAGGATTTGTCAACAGTTACTCCAGTGACTCTACATGTGGATCCTC

>287074

TGCAGTTTATGTTTTTGTGTATGTTTTGGCTAAGCAGTGTGGATGTGAGATCTGTGGCTTGGCTAAAAATCCGACACAAAAACTTGGTGGTGGTAG

>15592

TGCAGGTCCAGAAAGGAAACCAGTCTTTATCCAGCACCATGTTTACGCTCAGATGTGAAACTGGAAGCTGGACGTCCGATTGTCATCAGCGCTAAG

>65981

TGCAGCGGTTCTCATTGATCTGGCGAAGTGGGTTCTAGATTGGTGTGCTGTGAATGTAGCATCTTCAAACAGCCCAGGGTGTGTTCCTGCTACTGT

>156683

TGCAGGGTTCCACTCTTTCTGACCCGCCTGTTGTGAAAGAGCTGGTGAGAGCTGAGGCCAAGATGCTACTGGAGGCACTCAAAGGAAGCGCCTGCA

>72349

TGCAGAGGTCGGCGGATGTGACCTTCAGTTGAGCAAACGAACAGTTCCCTGGTTTCGGTTGAACATTCTTCAGCGCGACACACCTGCTGCCGGTTC

>51269

TGCAGGAGTGCTTGCATGCATGGACAGAGAGTACTCTGAAGATCTACTCAAGTGTATAGCCGAATGTTGGAAAAAAATATGGTCAAAGACAAAGTC

>61720

TGCAGGGTTTCAGTTCGTTTCTCCTAAAGATTCAATTAGAAAACAAACAGAACCGGATCTGGAACGTTCTGCCTGAGCAAAACGGAACGTGACCAA

>209741

TGCAGTTCCCGGCTGGCCAGCAGGAGGCAGGTGAGCCGGGACTTGAGGTACACGCGATGCAGCGGCCGCCGGTCCTCCAGGCTGTGGACCCGCAGG

>50901

TGCAGGGCGGTGATAAGCAGCTGCACCACGTACGACGGCATCAACGTCCTCCCCATTCCGAACGCGCTGAAAAAACACTTGAAGGAATACCACTAC

>141684

TGCAGAAACACAATCCCAACACTAGGGAGACGTCAGCATGCAGGACGGATTTGTTTCCCATTGTCTTTATAACAACAGGAACTTTTTGTTGGTTTT

>166478

TGCAGCAGCACAGAGCGCCTCTTCTTCTTCTCTTCATCCTCATCAGAAACCTCCACCGGCTCCTTCAAAGCCGCCGCGGCGGATCGGCAGCCGGGA

>256040

TGCAGGCTGGACATTTAGAGGTCAAACATTCATGATCGGTAGTACACATCAACATTGGTCAATGCAAATCATTTTGCACACAAAATACTCTTTATG

>23745

TGCAGCTTGGATTACATACACACTGCACACAAAAACCATGTGGACATTTTTTCCCCGGCCCAAAAGCTGGTATTGATAAAAGGAGGAATGTGTAGA

>133322

TGCAGTTTAGATGCCAATTCATTCATGGAGCGAGTCATAAAGACGGAACTACGTACAGCTCGAGTGTTTCCGCTCGATTCTTTATTGAATGTTGTT

>160715

TGCAGACATGCATGCTGCCAGAGCAGATGGTTTCCTCCTGAGCAGAGAGGAAACGCTCAGGCGGTTCTGCTGGGAGGAGCCTGGCGGCCTGAGGAA

>171711

TGCAGGTATGACAGTGTAACGTGGGTGGTGTCAGTGAACATCTGTCTCAGAGGCCGACCGGCTTCCAGAGACGGGAGAGAGAGCCTCTTCTTTGAT

>13295

TGCAGGGGTGAGGGGGCCGGGGCCGCGGTCGGGGCAGGGCCAGCATTGCAGTGAGGGATTGTGGGAGGAGTCTCAGAGTCCCTGAGGTTGACCCTG

>62097

TGCAGTGTGAACGCAGCCTATGACTGTGTCTCTGTGTGATGCACTAGCGGCCATTCCAAGGTCGCCTCCAAGGTCGCCCCCTGAAGACAGGCGATT

>298969

TGCAGCACCAAACATGCAGTTGAGAATTTCAGTAGCATAGATCCAGAGCCAGATGGCGTGGGGCCCCACGGAGCCCTGATCTCCGCTGGATTAAGA

>72435

TGCAGGACGGCGGGGCGGCGGCTGCGGGCGGCCCTGGGCGCGCGGCCCCGGGTCCCTCTGCTCAGGAAGGGAACCGCGGTGAGTGGAGCGGTAGAA

>325733

TGCAGTTGGACACTAGGTGGCGGCATTTACAAAGACCTAGAGATTTTATTTGGCCAATCATTTCCGGTTTGTTGACTGTTTGTTCTCAAATGTTAG

>42648

TGCAGTTCATCACCGTCACCAGCAGGAGGCGCTGTTTCCCTTCAGGACCTGATCAATAAACTCTTCCTGCTGTGGATGACGTCACTTTGCGGCACA

>213537

TGCAGCTCACAGTGAGACACATGTTCACTCACACAATAACAGAGAAAAAATCCCGTTCCACTGCTGCAATTACTAAAATTGCTTCCCAGCCTTCGA

>306406

TGCAGAGATAAAAGCACAAAACCTTCAAGGCGCCCCCTAGTGGGACGCCGCCCTACAGGCTACAGGAGGTGAAACATTCAGAGTTTTAAGATCGGA

>176431

TGCAGTCAGACGGAAATGAGAAACTTTGTTTCTACTGCTGTTCACTGAGAGCCTGTTGTTTACATGAGGTGCACGTGTGTAAACAAACTGGTTGCC

>155896

TGCAGCTTGTTTGGCATCGCATGCTGACTCTTCATCTGCTCCATCTAACCAACAACTAAACCGTTGCTGCCTGTTCTCCCTGTTCTAGTTATTAAG

>4915

TGCAGAACGGACCGGGCCGGTTCTGCTGATCAGAACCTCATCAACCCGCCTCCATTGCAGGCTGGGCTCTGGTTCTGCTGCTTCATGGAGCTGTCC

>31537

TGCAGATCGAGCACAACAGCAGCCAGTCCAGTTACGGGCTCGCTCCATCTGGTCCCGCTCCCACCAACGTCGACGCCGCCGCCTTCTGCATGGCCA

>188471

TGCAGTGGACGCCATGTTGCTGATAAAACAAGTTCATTCCTTCTTTTCCATTTTTTTTCATAAACCAAGTAAACCCAACATTCCTACACCTACAGG

>166843

TGCAGTGCTCTAACAGGAGGATGACTCGGGTTTAGCCGTGGTTCTGACTGCGGCTGCACGGTGGCTCTGCGGTTCTGTGCAGCATTTGAAGGTTCT

>117886

TGCAGAAACGACCTGTTGCCTCATATTTATGTATCATGCTGGTTTCACTGAGCAGGCAGGACTCAGATGACCTGAGGCTGCTTCTGCACATGGTTC

>97121

TGCAGGTCGCTGAGCTGGATGGGCTGGTGAGGAGGAGGGCTTCCAACGGAGGCCGGCGTCACGGGGGCCTGAGCTGGAGCTGCAAGTGGGGGAAAA

>289184

TGCAGGGTTCTGGCCGTCTCTGATCTGGTTTCAGTGGTTCCTCCTGAACTGGACTCTGATCTGGATTCACGCTCGTCTTTAAGATCGGAAGAGCGG

>219120

TGCAGCAGTAACTTTATGAAAACAGGATGCTCAGCCTTTCAAAACCTACTGCAACAGGTGAGCTTTTTTGTTGAGACATAAAGGAAATCTAACTGT

>296977

TGCAGGCAGCCAATCACAGCTGCTCCTTACCTTCAGCCTCCACCTGGATGCTGTCCACAGAAAACTGGAAAAAAGCAAAAAATGTAATTAAGATCG

>210348

TGCAGGAGGACCGCAGGCAGACGGAGACGGACGCAGCAGAGGACAGATCAGAGGACAGAGCAGGATCTTCATCATCTTCATCATCTTCATCATGGA

>23993

TGCAGCTCCTCTGATGGTGCGCAGCGTGGTGAACTCCCAGTCCATCCCCATCCCGGCGGCGGTGGCGGCCGACTCTCCGGGAAGAACCAGACAACC

>21940

TGCAGGGAGGACGGGCTTCAGCTCGCTTTGGACGACAGAAAGAGGGAATAAGTAGTGAAGGGTGAGAGACGATGTGTTGCAACACATCATGTTTAA

>83247

TGCAGAGCGAACCACTCGAGTCGTAATTTCAACTCCAAGTGGTTCAAAATGTTTGACAGGGATCCACCGCGGTCCCTGGAGTCGTTGCTGCCAGTA

>50789

TGCAGATCCACGGCCTGGACGACCCGGTGGGTCCGCATGCCGACACTGCTGCCCGGTTCTGATCCGCTGTCCGGTTCTGATCCGCTGTCCGGTTCT

>99479

TGCAGCTTCCGTCCATAAGCTTCTAGTGGGCTTTGGTCCTGGAAAGGTCTCTCCAGATCAACTCCAGGACATCTTGCTCAGAGTCCCCCTCTCTAA

>156589

TGCAGGCCAGGTCCATCAGATGCAGGTAGAGTGATAATGGAGGGGCAGACATATCAGAATCACGACAAATCCTAGTTCAAGCACATGCCGATACTT

>31269

TGCAGCAGAGGAGATCAGCAGCTGAAGCGTCTTTGTTGTTTTCTCGTGTCTCAATGAAAGCCGTCCTGTTGTGTCTGAATATCCTGAGGTGACCAG

>151973

TGCAGGATTGAAAATGGCCGCCCCGACCAGACAGCGGTGCAGAAAGCATCACGCTTTGATTGTAAAGTCAGATTTTGAAGCAGTCCGTGAAGAGTT

>97099

TGCAGCGTGGAGGCAGTGTGTGGGAGACGGAGAACAGCATGCAGTGAGTCAGCTGAGGATTGTTCTGTTGTAACAGAGCCAAGACACACTGAACCA

>178754

TGCAGCCGCCATATTTTACCTTCCTGAAGGGAAACTGTTGGCAGGGAATGCGACTCTTTTCTCCTCCAAATCCTCAAAATTCCAGGGATTTGGATG

>306903

TGCAGGGGGCGGAGCCTCCTGGTCAGGAAGTCTCTGTCTCCTCGGCTGAAGCGTCTCACCTCTGATGGAGACAGAACCGGGTCAGAACCGGGTCGG

>218956

TGCAGAAATATTGAGCTACTTTGGCTCCGGATGCGAAGTGAAATACCGTGACAATGTGATAAATAGCTCTCAACTCTAAGGTTTACAACAAAGGCA

>260459

TGCAGGATCACACTGAGCGTAGCGCAGCCCTGACCTGTGTGACCGAGGAAGAACTTTTTACGAGCACAGTTTACAACTTGCAGTATACTATTCACT

>135048

TGCAGATGTTGAGCAGCCAAACACGTTGGCCGCACAGCGAAACGTCAGGACAGACAGTAGCATTTAGCATTTCGTTTCCCTTTTCCCACTCAAAGC

>269333

TGCAGCTCTTTGGCTCTTTTTGAGGACAGTGAATAGTATACTGCAAGTTGTAAACTGTGCTCGTAAAAAGTTCTTCCTCGGTCACACAGGTCAGGG

>180721

TGCAGCTAAACTGCGTGGGAGCCCGGTCCCACCAGAGAGCACCGTTGTCCACACTCCACAAAGACATAATGCAGGGTGTTTCTTTGTGTGCTGCAG

>282661

TGCAGCTCTCTGCTATCTGAAGCGCCGCAATCCGCAGCTGCTCAGCACAGTTTCTGTCTGTTTTCAGACTTTTGTTGCGTTTTCCTCTCAGTTTTA

>5585

TGCAGGTTGTAGCCCAGGACCAGGAAGCTGTGCTCAGTCAAACGAACTGGGGGAAAATGGTGCTGAAGCTGTCTCAGTTTGGAATCAGAACTGGAA

>318062

TGCAGTTCATAGTAGAAGACAATGGTAGGTAAAGGTTGATATTTGAATCCCGTCTTTTCTTTCTATAACCATGAAGTACACCTCTATCCAGCGAGC

>273970

TGCAGTTTTTGTTTCACTGTTGTTTCTTGCATTCAGTCATGTTTCATCATCAAACTTGCGTTGAATTTGATCTAGTTGTATTTTATTTGATGTAAC

>74941

TGCAGTTTGGTGCCAACCAGCATTTCTTTTCATTTATCCATCACCTGAATGCGAGTCAGTCTGAATCCTTCATGGATCTTCTTCTCCACAGCTGAT

>195966

TGCAGGCGGTGAAGGCATCGCTCACTGACCTTCCCAACTCCCCAGAGGTCGTTTCCTCCAAGAAGAATCTGTTTGAGGCTGGAGAGGCCTGGAGCC

>58030

TGCAGAGAGCGCCTCCCTGTGGCCTTTCAGACATGAGTGAAGAGGCAACGTCTCAGTGGTGAGCTTTTACAGAGGAACAACACCTGGACACCTGTT

>51556

TGCAGCGTCGGAGCGAACCACGCGTACGGACGGGACGTGGTGGAGTGTCACTACAAAGCCTGCCTCTACGCCGGCATCCAGATCTACGGCACCAAC

>119928

TGCAGCTCAGGTGAGACAAAAAAAAGAGGTTCGCTTTCCACCTTTATCTCTCACTGCGGATGGACGGCCAGCGCAGAAGCTCACAGGCCCCAGTAA

>219569

TGCAGTCCGCTCCATCAGAACCATCATCAGCAGCAGAACCTTCATCTTCACCAGAACCCGGCCGTCCAGCAGTCAGAATGAAGCTCAACTGCAGC

>55420

TGCAGCTACTCTCTGGAACGGAGAAGTGAAGGTGAGCAAGTCAGAAAACATCCTCGCAGCATCTGGGATGAAATGCCTGTTTTATTTGTGTGCTAC

>46028

TGCAGATTAGGATGGACCAACCCTCTTCTTGAGCTGCTGGCCTAATCTCCCTTTTGCATCTCAGCCGTACGGATATGAGCGTCTCATAATCCACTC

>325914

TGCAGACCCATCCTGCAAATGTAATACAATGTAGATCTGCCACATTTTCATTCTCAATATAGAGCAGCAACAAAAATATAACTGGCATCATTGTTC

>153885

TGCAGGCACCACCATGTCCCCCTTCAGAGAAAGCAATATCCCAAACAGAACCGGATGATCTAAGGTCGCTTCTCTTGTTTTGTTCCCAACAGGTGA

>123890

TGCAGGTTCTGGTTCAGCTCTGGATCCTCCTGCTGGACCTGCTGGTTCTGACGTCCTGGCAGATTCTGGACCCGCTCAGACGGGAGGTTCTGCAGG

>250463

TGCAGAGCACCGCCTCCATCCATCCTCCGCACACACCAACCTCATGCACAATTATGGGGTGTTTCTGTGAGCGGGGAAAGGAATAAATATTCATGA

>55431

TGCAGAATCGTTTCCTCCTGTCTGATCAAAGATGGCCGACGTCGCGCAGGTTCTGCCGGACCCGGAGCTTCGTCCAGCTCCTGCTGCGGTCGTGGA

>174738

TGCAGCTCCTCCATAGTTGCAACAGTATGAGCAGGCACACGCAAATTTGTAACGGCATGAGCAGCAAATGTTCTAACTTACTTGGGATATTGCTTC

>71746

TGCAGCCTGACGCGAGGTGCTCCCTGCCTGCTGAGGTTCGTGGCGTTCGTCTGACTCGACAGAACTTGACGCTGATTCCAACAGAAAGGTGTCCGA

>160591

TGCAGATCACCAGGGAAATCACAAATGTCTGTGGTGCTTCACGGTGAAAAGTCCCAGAAAGGTGTGAAATATAACAGCTTCACTCACAGAAAGACA

>124576

TGCAGCAGGAGAGGGATTCTGCACGAGCAAATTCAAATCAACAGGATGATGAACAAGCTGACCCGAGTACAAGTGGAGCTCCAATGAACACTGGGA

>330096

TGCAGCCAACAGAAAGACAAAGGAGAAAGAAAGGAAGAATAGGGAAAGAAGTATGCAAGTAAGGAAGGAAAGATAAGATATGAAGGAAGGACAAAA

>118694

TGCAGGGTTACTGGTCACCAGGAGGCCTTCATCAACCCCCCACCTCCGGCTCCGCGGCTACAGATGTGCAGCGTGGCGTGGCGTCGGCACAAGGAG

>182901

TGCAGCCACCCACATATAAGTTCTGCGACCAACATGTCAGGTTGCTTACAGATGTACCAAAGAAGGCACAGGATCATCAACACATGCAAGCGTTGT

>104127

TGCAGGATTCGTTCTGGCTACTGCTCTCCAACATCCTTCAGAGGTCAGGGGTCATTTCCTCTGAGACCATCTGCCAGCCGACGAGAGGTCAGTCTG

>330698

TGCAGGTCGGTGTGCTGGTCTTACTGGGAGGACTGGTTTTTGACTCGTTTGTGGATATGATTGATCAGATCTGATTAGTTTTCATCAATGGTCCTG

>25555

TGCAGCTCAGAGGTCAGCAGGTCAAACTCTGACCCCAGCGTCATCAGTTCTCTCTGCTGCTCCGAGATCATTTCTGCGTCAACGCTGACGTCTTTC

>22573

TGCAGGAAGATTCACTGCCGTCGACAGAAACTAGTGATGAGGCAACAAAACCAGAGTTTCCAACAGTAAAGCAGACCAGCAGCAGAGGCAGCACAC

>38785

TGCAGGCCTCGGCATTTTGATGAGCCGGCTGCTGTTTGTGAATGCAGCTAATTAGAGCCCTACAGGCTTACACAACCACCAGGCTGAGGTTCCCAA

>302699

TGCAGGCAGGGTCTGGTCTGAGGCTCCGTCTGGCTGGGAAAGCGTCTGTGTAAGACCGGGAGCTCTCATCTACACTGATGAACACCAGGAAGAGCC

>259876

TGCAGGAAGACCCAGTTTGAGATTCAAGCCCTTCCCCAGTGATAGCAGAGTCTTGTAACTTGTCATCAATGCTTCGTGGTTTACTTCTAAAGACTG

>184154

TGCAGGCTCAGCAGCCGGTTCTGGAGCGATAGGAGGTTCTGGACTAGATCACTGGATGTTTGTGTGCTCTCAGCTGTCACTCCGGTTCTGTTGGAC

>63157

TGCAGCTGATCTGTGAATGGGGAGGGGAGTTGTGATTTTTACAAAACTCTGATCTGAAAGAGGTTCCAACTGATCTATGTGGCACATCAGACTTTT

>156863

TGCAGGTCATCACTGACCTGGAAGCGGCTCCCAGCTGATGGCGCCCAGGGCTCACAGCGCCATCAGCTTCAGCCCAGAGACTCTAATGGTTCCCGT

>226851

TGCAGCGAACTTCCACACAGTCCAACCTCAACAGAAACGGCCCTTCTTCACGATTATCTACCAGAGACTGTAACAAGGAAAGAAAAGGGGGAAAAA

>131849

TGCAGACCCCTTCCAAGTAGAAGACCCCTTCAAATCTGACCTCTTGAAAGGTCGGAACAGCATCTTGCTGTTATTCATTCACGTCTTCATGCTTTC

>75124

TGCAGGAATGCCTCCACACTCCGTCCTCTACCAGAGGGTTTATATCAGCAAATCCTATGTGAAACGGTTTTGCATAGCAGTGGGGGGATAGAAGCC

>222215

TGCAGGGTGGTTTGCGGGGCAGTTTTGGGCAGTGGGGCGGTGTTGGGTAGCTGGGCGGGCTGCGGGGCGGAGTCAGGCAGTGGGGCGGGCTGCAGG

>50435

TGCAGCCTGCCATCCTCATCCTCACTTCGGGTTCTCCATCAGCGCACTAACCGAACCTCATACCTGGTAAAACCGGTAAAACCATCATATTGCCCA

>160339

TGCAGCTGTGGTGCTTGTTGCAGCTGGAGGAGGAGCAGAAGAAGGACAAGCAGGAGGTGGTGGTGGAGCAGCTGCTGTAAATAACACCGTCATCAC

>34168

TGCAGAACCGGACCGGGTTGAGGTTCTGAAGAACAATCCAGGTGAGACATGAATCAGAATATTTGATCTGTTTTCTCCATGCTGTTGTGACCAGCA

>313459

TGCAGTCTGCGGTCTTTTTTTGTGGAATTGCTGGACGGCTCACACCTGTTTCCCTAATAACATTTGTTCTGACCTGTGTCTGGTTGGCAGTAGTAT

>38375

TGCAGCAGCTTCTGGTCCGTTTCCACGGGAACGTCGGTGGCCTTGGGGTCGTCGTCGTTGGCGCCGGCCCCCGCGGCGCCGATAAGCTCCTCCTCC

>238764

TGCAGGTGGAGGAAGAGTTGGAACCAGAACAAGAACCGGTACCACAGGTAGAGGCAGAGCCTGTAGAGGGTCCCGACTTTCAGGTGGCGTCTGAAG

>176945

TGCAGCCACAGCTCCTGTTCTTCAGGCAGCCACAGATCCTGGCCCAGTCCTTTGTGAGAATAGGGCCAAACTAATTACAATCAGCTGCCTTCAATC

>36309

TGCAGAAACTCTTGGAAAAGCGAGGTCTGCTCAGCCGGCAGGCTGACGTCCATCGCGTTCCCATCCTGGTCCTCGGGTGAGCGTAGCCAAGGGCCG

>77626

TGCAGCAACAGATAACGTCACACCAAGAGACTTGTGTAATGGTTCCCCCTTATCTCCCAACGCCAGAACAGGCTGCTAGCAAAGCTCTGTCTGAAA

>24852

TGCAGAGTTGCAGGAGACCAGCGCGTCCGTTGTTTTTGTAAATCCGCTTTAGATCCGAGTGTGTTTTGTGTGCGCGGCGCGCTGCAAATCGGCTTT

>263886

TGCAGGCATTGTAATCAAACACAGAACTTGTCGGAGGAGATGATGTTTCTTTGGTACTCACTGTCAAGACATGGCGCACACTCTGTCTGCTTGGCG

>340285

TGCAGGACGGCCGGGTTCTGACCGGGGTTCTGTAGAACCTGAAGACACATCCCCCCAGGGCACATCCCACGTAGCCCTCGGTGGCGTCGGGCTGGT

>93537

TGCAGCTGATGGATGGAGCGCCGACACTAACACGGTTTCTCTTTATGGAGATGAGATCGGATCCAGAATATCTCTGTTTTCCTGATGAACATTCCC

>107163

TGCAGCACCGGTTGTGTAAGTCAAAAGTTTTCAGCCAGAGGGGCCCAGTAAAAGGTGCTGGATTTCTGTTCCAGTGGAGGCATCATAGGGCGTTTT

>186404

TGCAGGTGACGACAAAACCCAATCGCTGTTCGTCTCCCCTGGGAGACATTTGCCTCGCACAGGAAGAGTATGCTGCCCTGACACATTGCACTCCTC

>273891

TGCAGACTGTGTGGTGATTATCCAAGCGATCGAGCATGTTGTTTTTTTGTTTGTTTTCTTTTTCAATGTTTTCAATTTGCACATGATTTTCTCAGG

>170667

TGCAGGATGACGACGACGTTGATCTCAAACTCTGAGGACAAAACGAGCCGTTAGTTCCTGAAACGATTGGTAATCGATTAAGATCGGAAGAGCGGT

>180301

TGCAGCGGCATCACAACATCAGGTCATCATCAGGAGGATGAGAGGAGAAAAGGTGAAGATCATCTTCCTCATGGAAACATGTCCTCTGTTCTCACC

>111799

TGCAGGTAAACGGAGGCTCCGCCCCTGACCCGATTGTCCTCTGATTGTCCTCAGATGACCTACGTCCTGCTGGACATCGCCATCCTGGAGCTCTTC

>209748

TGCAGCATCCTGCTGCTCCATCCTGATTGCAGCTGCTGGGGCTGTTAGGTGTGGTTATTATGGGATGGGGTGCAGACTGTTTGTTTTCAACCCAGA

>78490

TGCAGCTCAGGCATCTACGCGACAGCAAATGAGGAAAACAAGTCTAGTCTGGCTTATTCTCAAACAATGTGTAGCAAGGATGTGTCATGATCCATT

>239826

TGCAGGACTATAGCGATGGTGAGCCGGACTGGAGGGGTCCAGAACTTCAGAACTCTGGCCGTTTGGGGCCGATGGGCCCAGACTTCTGGCCATTTT

>238361

TGCAGCCGCTCAGATCTGCGAATCCAAATGATAACTCAGCTTCAGGGTCATAGTTTGTAAAATTCACTGCTTCAACTTTGTTCCCTCCCACCAGAA

>238249

TGCAGCTGGAGTGAAGAAAAGGCGGGTCTGAGGTCAGGAGGTCACTTCCTGTGATTCATGGACTTTAGTTCATAACAGTTGGAGAAAACATTCATG

>119711

TGCAGGATCCAGCACATCTGGAAGGACGGAGGAGGACACGTGGTGTGGAAGGGGACGGTTCTGGACCAGGTGATCTGGGTTCAACCAGCCGGGTCA

>200552

TGCAGAAAGAAGGATGAGAAGGAGCGGGGGAATAACGCCGCCGCCTGTGAGCGCGCCTTCAGTTATCGGATCACGTTTCCCTGCACTGTTGTGCTC

>247592

TGCAGATGTTCACATGAGTCCCAAATATCAGGAGCACAGTAACAGTGACTGAGTGTTGCTCAGTTAGTGCGATGGTGTCTTCTTCTCTTGTTCAGT

>29020

TGCAGAGGTAAAGTGCAAATCCTGGGTCAGTGCAAGTGATGCTATGAGAAGACAAATATGCAAAGAGACAGGATGAAAGTTGACCTTTACAAATCA

>121958

TGCAGATCGAGAGCATCATCGACCAGGACGTAAGAACCGCAGATGAAACGCCACACCGTCAGCGAACGTAACGAATGGTTCATCTTTCTCTAGTTG

>319924

TGCAGCAGGAGACGGCGGCCCGTTCAGAGTTCAGCTCCACAGACTGAACCTGTCAAGATGCGACACAGGAAGCAGGAGCTGATTCAGACCAGCAG

>300809

TGCAGGCGGCGCCGCAGGCCGCAGAGACTCACCTGTTCAGCCACCTGCCGCTGCACTCGCAGCAGCCGGCCCGGTCGCCCTACAGCATGGTGCCGG

>231362

TGCAGAGGAGCTGAGGCTGTGGAGGTTCACCATAAACATGCAGCTCACACAAACAAAGCAGGTTCAGTCGTCTCTTTGGTTATCTTTAGCTTCTAT

>104579

TGCAGCTCCCGGTGGCCAGATGAACCACCGTCCTGCTTCTCGACCCGATACTCTGGTGTGTATCCGACCAGAACGATCAGCATGGACTGGCAGGCC

>24245

TGCAGGGATGCAGCTGGTTCCTCTCAGGGTTTTCCAAAAGTAAATAGGTCATATTTTATGATATTGTTCTGACAGCGCGGATGTGGTTTTCAACAA

>37708

TGCAGCAGGGGAAATGTGTTCTGCTGGCTTTTGTTCCCTCACATTGCCATCCGCCGGGATTGATGGTGCTACGCTGAGCCGTAATGAAAAGCCACA

>303906

TGCAGAGGAAGCTTCCGTTCGCCGCTGCTGAACAGAACATCTGTCTTCGCTGGTTCCTGGGGAGATAAATGTTGTTTAGCGAGGAGTCCGTCAACA

>165559

TGCAGGATGGCTCTGGCTCTCAGATCAACGGTCTGACAGGAAAACTCCCAAATAAGGCTCGAGGAAGTCGTGACGTCGCTGGAATATCTCCACGTG

>235016

TGCAGGTGTAGACGTTGGATGGATGACACTCCATCCAACGTCTGGAATATGGAAGAAACAAAACCGCTTGTTCTTAGTTTGATGTCTAAATGATTT

>139556

TGCAGCTGAGATGATGTAAAAACAAACACGGCCCACTGATATTGGGTGATGCTCCAGGAGGTTGTAGGGGAGGCGGGAGGGTGGAGCAATCTGTGC

>153300

TGCAGGGAACAGAGAAACAAACCCTCCATTCAGCCTGCTGCTCACTGCTGGTCAGCAGGCTTACAACGGCTGCTCTGCCTGTCCCTCTCTTACAAC

>163089

TGCAGGAGAGGGGTCGTTGTGCTAAAGTGAGCAGTCAAGACACAAATGGGTATGAGGAGTGGATATGAAGACACTCACTGACACACATGCTCAGGC

>46984

TGCAGAAAGCGGCAGCTGGAGAAGTGAGTCATGCCGGAGCAGAACCTCTGCGATGTTCGGACAGAAGCACCGGCGGCCCGGAGCTGTCCGCACAGA

>87410

TGCAGACGCTGCACTGATCTGTCTACTCATCTCCATTTTCTCCTCATTCATGCCTCGGATTGGGGCAGGACCTCTTCCCGACCAGAGGGGGCGCTC

>280491

TGCAGCCGCCCGCCGCCCGCCGCCTCCGGCTCACAGGCTAATAAAACCAAACACCGCGCAGCTGCGTCACACATTTGAATCCAACCTGAGGAAAGT

>15605

TGCAGGACACCAAGGCTCCCAAGAAAAAGGTGAGACGTGATTGATGGGATGCTGAACACTACAGAGACAGTTGTCCTCAGCCTGTCCCTCTGTCTT

>52174

TGCAGTCTTTACTGTTTCTGCATCAATGTGACCAACAACAGATGTTCTTGAACCGTAGAGAGCTCTACAACCCAACGAGTCGACTGAGCTCTTATT

>269018

TGCAGGAGGAGGAAGAGGAGGAGAGAGGATCAGGTGTCCGTCATGAATAAACCTTCTGTCTCCAGCAGGTGAAAGAAAGTCTGGGTTTATTAAGAT

>223002

TGCAGGTTTCATATGACTTTTAGGCAAAGAAGAAGTGAAAACTGAAAGGTGGTTTGGTGCTCCAACGTGTACGGCAACGCACCAAATAAATGACTA

>50290

TGCAGGCAGAAGCAGCTCTTGGACAACCTCAGCTCAGAAAAATGGGAAAATCCAAAACATTGTTCACCAAACAGCCATTTAGCCATTGCATTGGCC

>100354

TGCAGCCATCAGAAACCTGACGGCGGCCGTCGCTCTGCGGCCTCCCAGCAGGGGGCGGAGCCTCCGGCCGGAGCCGTTTCCTCCTCCATGGCTGTG

>85081

TGCAGATTGTCTTCACCTTTCTCTAAGGCGAGTCGTCGTATCTTGACTTCACTTGTCCTGAAACCCGAGTGTGGTACTCCAGGTGCTACAGCTTTG

>72884

TGCAGATACGCCGTAATCCTGGCGTTCGACGTGAAGGTGGAGAGGGAGAGCCAGGAGATGGCCGACAGTCTGGGCGTCCGCATCTTCTCTGCTGAG

>207294

TGCAGCGACTTCTGAAAGTTTTCTTCAGTTGGAAGAGACAAGGGATGTGTGTGACAGAAGAGAAGCAGTCGGAGTTTTGAGGTGAAGCCACTGGAG

>104294

TGCAGCTGGGAGCTGAGGTCGTCCCTCTGGGCCGCCAGCGCCTCCTGCTGCTCCCTCAGCAGGTTCAGCTCCTGCGTCTTGGCGGTCTGGATCTGG

>233650

TGCAGGTAGATTGTGCATGCACCGCTAATACTACAAATCCCAGAATGCTCCTGTGTTGACTTGTCAACCACAAGACCCCATCCTCTGTTTACATTA

>267354

TGCAGGATGCTGCGTTCCCACGGTAACAGGGTGGAAAAATAAAGCTTTCCTATGGGAAAACATGTCAGCAACGCAAATAATCTGCTCTGCAAACAT

>313924

TGCAGGCCCTGAAAGCCATAACGCTGATGTTTCAGCAGAGGATCAGAAAAACGGGGCCAGTTGTTTACCGCTCTCTATTAAGATCGGAAGAGCGGT

>210804

TGCAGCTGCTGAACCCTCCCGACCCGGTCTCATCACTGGACCGCCAGGAAGTGGGGACACCAACTGATATGGAGCTCCTCTCCAGGGTGAGATTAA

>218258

TGCAGGGTCAGCGTGTCGGCGTGGCAGCTGGTGGAGAGGACGGAGATCCGGACCTGGAGACGGGACGGAGAGAGTTCTGACCGTCTGGAATATTAA

>240513

TGCAGCTAAATTCCGCCCCCTGGTGGCGCTCTGTGGCATCGCATCGTGGTGAAGCAAAGATCCCGTTCCCGTTTGGACAAGATCACCGTGCAGGAT

>90865

TGCAGCTCTGAACCAGGACCAGTGAGAGTTCATTAGACGATCATCACAACGTTACGCTGCAAACAGAACCAGGAAGCGGCAGAGAATGACTCACTT

>141591

TGCAGCGACAGTCCACGGCTGAGTCATAAGACCAGACCTCTCAGCCACAAACCAAAACCTCACTGGAGGTGTAACTGGAACTGTGGCGATGCAAAC

>200727

TGCAGCTTAGTTGTGTGTGAACTCAGATTTCTTGCATGTGTGTCCAATGTGCCGCCGTACTGCTGGGTTTGTTTAGCTAATTATTTTCAGATCTTC

>250599

TGCAGCTACAAGCAACAAAACGGAAAGGAGGGAAAACGTCATGCATTCAAATACGGCTGCCGAAGAAAACCCGACGCATCCGCCAAACAGACAAAG

>305508

TGCAGCCATGTGGCAACGACTGAACCTGTTAGGTGATCTAACAGGTTGGGGAGGGTAAACGTTGCCCGCAGCCTGAGACAAACCTCTGAAAACATC

>157921

TGCAGGAAACAAACGACACTCAGGTGATGTTTCTAACCCGGCATCTCTGGAATCCGTGTCTACGTCCGTTTTAGCAGCAAAGCTAATTTCTTGTTT

>52623

TGCAGTAATGCATGCTAGAACATTCTGGAAGGGGGTAACCCTTTTAGCCTCAAAAACACCTTGGGATTGAGCTGTACATAAACGGAAGATGATGGA

>321804

TGCAGCTGTCTAGCTGCTGCTGGCTCTCTCACATCATGCTCTGTTCTTTGCTGGGGGATTGTCCTTGGCATTTTGCTCAAGAAACGAGTTTAAGAT

>35363

TGCAGGCCAGAAGAGGAGACACAAGGGCATCAGTCATCATGTGAGACCTGGGACGTTCAGGAAACTGACACCACTTGATTGGACCCCTGAGTGTGA

>5751

TGCAGAGAGTTTCTGTCCGTCGCCTTCTAATGGCTGGACGGGCAGTTTTCCTCCCCAAACGTCCAACTCTTCACCAGCGTAACCTTCATACAGACC

>205348

TGCAGCAGGAGGTCCAGGAACTCATAGCAGCGCCTGTTGGCACAGAAGAAGAGAAGAGCAGAGCGGCCATGTTGGACCAGTTCCAGACTATAGTCT

>258198

TGCAGGTACCCATCAGGCTCAGCGGCGCCCCCTGGCAGCAGAACAGCTAGGTGAACACAGAGCCGGAACTGTCAAAAGCAACACCAGAACCTGAGC

>290987

TGCAGCACGATTGGCATCATTTCCTCCACACAATGGAACATCATTCTCATATCTTTCAGTGTGAGGGAATGACAATAGGCCGTGCACCATTCTTTC

>174711

TGCAGACGGTTCTGGGACTGACTGGTTGAGACAGCTTGAAGCCTCCCAGGGAACAGAGCAGAGGAAAACCCAGCTGGGACCCAGTCCTGAGCCAGA

>103030

TGCAGATGCATCACGTCTATGTCTGTAGCAGCTAAGCTGCAACACTTTTTATTCTAGTTTATCCCACCAAGCATCAGCGTATTTGTTCACAGATTT

>284358

TGCAGGGCTACGAATTGGACCCCACCAATTTGGGCGGCTGGTCCCTGGACAAGCACCACATACTCAACACGCGCAGCGGTACGTGGAAGCGCCTCG

>298291

TGCAGGAGGACAGCGGTCCAAGCCGAACTTATGCTTCTGGTTCTGACCCAGAAGGCGGGTTCTGGTCCAAAAGCATCAGATCAGAACCGGCTGAGA

>113153

TGCAGGCGATCTTTGTCAGACACTCGTGATGTGAAGCAAGTGGCTTAGAAGACAATGATTTTATTGTAACAAAGGGCTCTGTTGACAGCTGTAGTT

>299212

TGCAGACCCACTTTTTGCGCCACTGGTCGCATCCACGAACAAGGCCTCCGTATCGTCTCACGCGCTCAACCCCAGCGTCCCGAAATGATTTTCTAA

>70780

TGCAGCTCTGACTCATACAAGGTGGAGGCAGAGACGACCAGGGTCTGATGACTGAAAGCGAACACACACACACATTAGCACATATATGTGTGTTCA

>128862

TGCAGGACGAAGAGCTGCTGCTGCTCCGGCCGTCAGGGATCCTCTGCTGCTCAGGACGCCCACCTGCGGCCCGCAGCCTCCTGTTGACCCACCCTG

>141218

TGCAGCAGAGGTGCCAATACATGAGGCCAACAGTGTGTATAACACTTTCGCTGTCGAGCTGCAACGGTAAATCAGGATAATTGAAGATAACAAGAA

>139988

TGCAGGGACTAATGCTACTTTTTTCTCTTTGTAACATTTCCCATCCTTGCAGATGATGTGGCAACGGAGTTGGGCAAAAACAAGTGAAACATCTTT

>239505

TGCAGAAACTCTGATGGCTCTGACATAATGATATCTGATGAGTACAGTGCACAACAATGAGTCAAGGCTTTTAGCAGGGAAAGCTACACCCACTGA

>230475

TGCAGCTGGCATCAAAACTGAAAGGTGCTCTACTACCACCTACTTGATTGGAGTGCAAACAGAAATAATGTGTGCACTTAGGATAGGAAATTGTGT

>158526

TGCAGCTAAAATAACAAGAACTGGTTATAATGGTTTCTTCGTCTGCCGCCCATCACAGTTATTTGAACAATTCACCCAGAAAAGCTCATTTGGGTG

>261222

TGCAGGACTGAAGCCAATCAGATTAGCTGCAATGATAGAAAGTGCTTAGTGCAAGGAGAAAGCATGTCGGAGCGTGAGTGGCTGTAGAGGATATTT

>282548

TGCAGTCCTCCCACAGTGCACCGGGTCTCTGTGGAGTTACAGGACAGCGGATGGCCCATTGCTCTGGAGACGACGGCGATGTAGTTCTCTGCAGT

>131529

TGCAGCTGACTCAGCCTTTGCTCCGTCTTTTTCGAACACTCATCAGACAAAACAAAAGAGCTAATCCTTACACAATCCAGTTTTCATGCTCTCAGC

>69294

TGCAGTGAGAAGACTGGATATGGGATCCTCCCTACATATATTAGGTCAAAGCATTGTGGGGTCAGGTTTCTAAATAAAAGATAAATGTGCCCGGGG

>193104

TGCAGGCTTGACAGCCGGCCAAACCTGCAACAAGCTGCTCCAGCTCCTGCTGGTTCCTTCAAACACTCATTTCAACCAGTTATCGCTTATCATTCA

>27423

TGCAGGACAAAGCGGACCACACACCAGCACACGTAATGCATGTTTTCACATCAAGCAGAAGCAACAACAGCCTAAATAAAATCTCCCATTGGTGTT

>158953

TGCAGGTAAACAGATCCGTTCTCTCTGGTGAACCACAGGGCTCCATTCCTGGATGTTTGCTTTTTTTCACTTGACCTGCTGCCGTTTAAGATCGGA

>176407

TGCAGAGGAAGGCTGGAGAACAACAGAGTGGGGAAGCCGGTCTGATCGACGCTTCCGCTCCCAGCAAGTAATTCATTCTGAGAGGGAGGCACAAGT

>96454

TGCAGACATTTTGTCCAGTTACAGCCACATGTTACTTTATTCACTCAAAGTGAATACTTTGTAACCCAGGCTGGACTGGCACTGCCCTTTGTAGCC

>149195

TGCAGCTGAATTGTTTCCTGCTAAAATGCGACATGCAGTTCAGACCGCGGAGCCGGAAGGGAACAGATGTTGAGGAAACATCTAGATGTACACCAC

>260703

TGCAGTAGTTAGCTTGAAAAATATGCTCGCAAGGACGAGGCGAAAACTGATGACGAAATGACACAAGAAAACAACAGTGCCTCACTTTTGTAGGGG

>18657

TGCAGATGTCCTCCAGACCAGGCCACCCTGACCCAAACCCGGCTGTGTGTGTTTCAGGATCGTGGTTCATGGACAGCCCTTCCTCATGTCCATCAT

>60133

TGCAGGTGAAGATGTGTGTGTCGTCCTCAGTTTTCCTCTCAGACGCCATCAGGTCCAGCTGCACGTCTTCAACTTCAACAACACAAACGTACTGAG

>56395

TGCAGCTCAAAGCCTGTCTGAAACACGCCGCCTGTCTGCCACTGCCCTCAAACGTTCATCATTTTCTCATTATTGAACCGGTTTGACAGGAATTGA

>185823

TGCAGCTCAAGAACAGGAACAGGCAGCCAGGAACTTTGGACCCCAAGTGTGCAGCTAGGGACTTGGACGGGGTCATTTAAGATCGGAAGAGCGGTT

>147529

TGCAGGACAGGACAGATGAGGCGTCCTCTGGACGAGGACAGCTTGCAGCAGTTCCTCACACCATTGTGAACGTCTCCACTGAGCCACCAAGAAATT

>154041

TGCAGAGCAGACAAAGACTCTGCGGCATTCGCCATCCAGCCCTCCATCCTCCAGCGAGTGCATCACCAGGACCTAGTCCCGTAAAACAGGAGAGGT

>140359

TGCAGTAGGAAGCATGTCAACATCGCCGCAGCGTATAACAACGCATTTGTGTCTCCTCCACAGCTGACAGCACATGCAAGACCACGCAGGGAGGCG

>257947

TGCAGCTTGGAGGAACGAGGAACCGAGACGCTGAAGTCGGAGCGCAGAGCTCTGCTCACGTTCAGCCTCAACGAAATACTTTAAGATCGGAAGAGC

>101337

TGCAGAGCATCAAGGCCTAATCTAGGTCACCGCACGCGCCCCGAGGCTTCCACGCATCAACACGCATCACGATGTTTACATGGAGTCACGTGACCA

>207447

TGCAGCTCGTGTTATTCTGAACTGGAGTTTGAACTGTTCTGCGAAGTATCAGCCTGGCTGAAAGAAAACATGTTATGCAATGGGGTTTGGACAGTT

>156248

TGCAGAACGACAGCAGTCAGAACCACCGACCCAACGCAGCCTTCGTCCTCCTCCTCCTCCTCACCTGGTTGGTGAAGGAGTTGCTCATGACGAAAG

>35042

TGCAGATAAAAAGCACTAGTAACAGAGGTTCTGAAACAACCTAATAGAGTGCAACAGACTGACCGCCCGATATTGTATTTGCTATGCCTGCGAGTA

>16769

TGCAGCTTCATGCTGATTTTCCGGGATTATCCCACGTATTTGGGAATGCAGCCCTCCACCTCCACCATCTCAGGCCAGCTCTCGTACTTGTTTTTG

>30782

TGCAGGGTGAGGTCGCCGTAGCGACGCAGGGTCAGGTCTCTCGCCTGCCTCTGCGGAGGCTTTTTGTTTTACTCCAGTCTTATTATGTTTCTCTGC

>145722

TGCAGGTGCTGCGCTGCCGCCTGACCATCCAGCACGCTAACCGCCGCGCCGCGCCGCCGCCCCGCGCCGCCTCCGCGCACTCGCTGCGGCCGCTGC

>188715

TGCAGCTTCTCTGGACGACAGCGGAGAACAAAATGTTTAGCTCTCCATTTTCATATATACTGTTTGCTGGAGGAAAATGTTGAGAAATAAAACCTA

>163908

TGCAGCCCTAATTTCTGATACATTTCCTTACCTTAGTGCAGAGATTCTTCAGAAGACCTTGCAGGTGACCGTTTTTGACCTGATGACTCACGTCCT

>63912

TGCAGCCCGGCTTTGTTCTGGCGCGACTGCAACGCCGTTATCAGAACCCGTTATCTCTGCTGGGGATTGGGCCGAGGTTCTGCCAGAGGAACCTTC

>237674

TGCAGAGCCGTGGCCAGCTGACGTGTCACTTCCTGTTTACACCTCCAAACAAACAAAAGCAGCCAGCAGCTTCCTGTCCTCTGCTCTGCCTTCTTT

>130443

TGCAGAGGAAGAGCTGCTGCTGGCTCATACGTGAGTCAACCAATCAGAGAGCAGAAAACCTGACTTCCTGTTTGTTTGTGTCGACTTCCTGTTTGC

>283760

TGCAGTGCATCCAACCTGTTACATCATCCGACCTCCAAAAAGGAGCAACCTGGTGTGAAACCCAAAGTAACATGCATGCAACGTGTTAAGATCGGA

>92647

TGCAGGTTCAGCTGCTCTGTTTCCCTTTTAGACATGGAAACCAAAATGGCTGCCTTGAGCTACAAGCTGGGGCAAAAATAACAAGGAAACGTTGGA

>260534

TGCAGTAGCTACAGCTGCTCTGGTGGCTACAAGCAGCAGCATCAGTCTGAGAGAGACCAGGTGAAGAAGTGGAAGGTGTTGAGGACAAAGTCTGAA

>27821

TGCAGAACCCCAGTGTGAGGAGACGGAGCAAATCAAAACGGTGCACAGGTGAGCAGCAGAACAAAAGCCTAAGATCCTCCAGGGTGGGACTGAGCA

>40284

TGCAGCTCCGGGACGCGGACAGGTAGAGTCGATAATTACCGCCATTCATCTTCGCCGGACCCCGGCTCCGCTTTACGAGGGCCGCATCCGAGTGCC

>110180

TGCAGGCCAGGCAGAGGACGAACACCAAGAACGGCAGGAGAGGAAGTTGATGAGGCACAAAACAGAGAAGGTGTAGAAGTTGGCCTTTGACTTGAA

>46210

TGCAGAAAGCCACTGTACAATCTCAGGTGTAGCTGATTTCTGTTTATCCTGCTATGCTGCCCCCGATGGTGACCTTAGGTGACCTTCAGTGTTTGT

>146648

TGCAGCCTCGGCGGTGCCAGCAGCGCCGTGCGCCCCGAAGCGCTCCAGAGAAACCAGGCTCTGTCTAGTCCGTGCTTCCAGCGAGTTATCCTCCAG

>296844

TGCAGTATTTTACTACTTCTCACCTTTCTCACCTCCTCCTCTGTTTTTTTCCTCTTTTTTCATCCCATGCCGAGTCCCGTCTCATCACCTCTCGTT

>12619

TGCAGTGATTTTTGCATTCCTTTGTGCTCAGTCCGCCTCACACATGCATTCACCTGATGAAAACCAGCTCTGGTGTCCCGGACAGTCTCTGTGGAG

>154840

TGCAGACAGACACCCAGGGACAGACGGACAGTCAGGGACAGACGGACACCCAGGGACAGACGGACACCCAGGGACAGACGGACACCCAGGGACAGA

>220689

TGCAGGAGGACAGTGTGAGAACTTCGTACCTGACCTGGCCTCCCTCAGCGGGGGGTCAGAAGTCACGCCGCCCTGTTTTCCTCCATCTAACCACAC

>234829

TGCAGCCAGGAGGGAGTTCATGGTAACTATTGACCTTCATCATTCATGATTCTGCCGACTCAAGCGGGTATTTATTTACGCAGAATGTAGAGTATA

>257958

TGCAGGACTCCATAACGCCAGAGATGACCCAGAACTTCTTACATAAACAGCATGTGGTCACATTGAGGTTATCTACAAACAAGGCCAGGATAAGCT

>320788

TGCAGGAAGCCGCACTGTGAACCATGATTTCATACATGTATTGGATTTAGATGGATGGGCGAGGGGGCCCTGAAAGGACACTTTGTGCTTGGAAAA

>318038

TGCAGCACCGCAGGGAGACGGAGCGACACAACCGGGTCAGCAGAGCCGCCGACTGACCCGACTGGCGCCGGCCGCAGAGGTTCTGATGGGTTTCTG

>124735

TGCAGAGGAAAGGCCTCATGGGAAGGTTTTGTTTTCAGATCTTTGATGAGATCTCTCTGCTGGTGCAGTCGGCTCTGGATGGGTACAACGTTTGCT

>193827

TGCAGGTGGAGACGTGATGCATTCAGGGTCGGTACCTGCAATGCAGCCGTGGGACTGCCGGACGTGGTGGAACCACAGAGATGGCAGGTAAAGCAT

>153961

TGCAGTCTTGGAAAACTTTGGGAGCCGTTTTCCAGATCCGAGGTTTCAGTCTGTGTTCGTCTCTGGGGTGAAAAGTTTTCACAAATTTTTATCTTT

>230201

TGCAGTCGGATGAAACAGGAAGTTCCAACCAGAAACGCCTCTAGTGCCTCATCCTGCACTCAACGCTGGAGAAGCAGACAAGTCAGACATGATTGT

>213620

TGCAGTGACAGGTTGGAAGGTGAAACCCTTCAGACATTATGAGGAGCTTTTTTATTCTTAGCCATCAACTTCTTTTTCCTCTTTTGGCCAGGCTGC

>302100

TGCAGTTGAGCTTCATTCTGACTGCTGGACGGCCGGGTTCTGGTGAAGATGAAGGTTCTGCTGCTGATGATGGTTCTGATGGAGCGGACTGCAGCA

>18667

TGCAGGCAGTAACCCGCTGGACCACCAGATCCTCAACGTTTCTCTGCCGGAGTATTTTCCCAGCGCGAAGCGACCCAAAGTCGGCCCCTGCAAGGC

>244220

TGCAGCGTAACGGCTCCCTGTCTCTACTGGGGCTCAGCGGCGCTCTCTGTGAGTGAGGCGGCTCCAGCAGGGACCGGAAGTGAGTCGGCGTCTCTG

>328459

TGCAGCAGCAGCTCAGCAGAAACGACCCGATCAGGAAGCCGTCAGGGCCACACAGACCCGAGTCGACTGACCAGAACCAAATGAAACGTCACCGAT

>22776

TGCAGCTGGGCTGGCCCACTGCCCGGTTCTGACCCGTCACCTTTCAGAACCGAGTCCTCCAGACGCTCGCTCAGGTCGTGGCACTGCTGCTGGTAC

>225207

TGCAGAGCTAAAGGACAGTGGCTGATTCTTTGTAGAGGAAGATGAGATTGGTTTCATATAGGGCTGCACCGATTGCAGTTTATCAACCGTTCCCTG

>3265

TGCAGGTCAGAGGATGATTCTTTCCTCTGCATCCACTTCCTTCAATGCTGAGGTGAAATCCTTTGGACAACAGGAACCACATGTATCATGCAGACC

>183280

TGCAGCCATGTCTCCATACCTGCTGGACGTGCCCTGGTCGGCGTAGGGCCGGCAGTATGACGAGGGGAACCAGCCGCGTCTGGACACACACACACA

>195807

TGCAGTCGGAGCAACAGTGCAGTCAGAATGTGAAGCTGCGGTGGAGTTTCTCTGCTGAAGCTTTCGTCTGTTTGATGAGCTGTTGGTTTTCTGTGA

>79388

TGCAGGGCGATCTCACCTCTTCAGCAGACGATGCAGACGATGCACCGCCGCCAAAGCTGCACGGCTGCAACTCGATGCAAGCTTGCCATGCCCTGG

>107655

TGCAGGGCCTCAACCAGGCGCGCAAACAGGTACCGCCCTCCCCGTGTTTACGCCCTGGGGCAAGAGCCGCCGCCGGCTGCCCAACGTCATCACAGC

>186901

TGCAGAGCGGTGGCAACGGCGGCGTCCAGACGCTCCAACATCTGCTTCTTATCCGGGTCCGTGGTGGAGGCCAGCTGCTCCCGGAACGGCTGCAGG

>53123

TGCAGCTTGTTTCTGGGGCCTCGCTCAATCATCACTTCAAACACAGTCACGTTTCACAGACTTGGCCAGCGAGTGTTTGCGGCGCAGTGAAAGCGT

>125957

TGCAGTACGCCGACGCCTCTGACCAAGTCTTGGCCTCGAGTACAAAGACGTAACTCGCACTAAACGTCCGACAGGTGAAGAACAGACGGTTTCCAC

>275330

TGCAGCACACAAAAACTTCCCTGTTATGAAAATAATTCAGACCTCCAGCAGATGATATCTGAGCTACTATAAGGCCCAAATGCATTCTGGGTAGGG

>52216

TGCAGTTCGTCTCATTGCTTACATTTTACAAAAAGATGCTAAGATGCCCCCTGGTGGCGACTTCAGGAGGGTTTTCAGTACCTTCTGTTTTCTGTC

>287574

TGCAGCAATTTTCTTCCATCCTTTTCCTTTTTTCTTTAGCTGTCGGAGAAAAGCAATCAGAGATTTTGGGAAATCGGCCTGTATGCGTTTGAAGAT

>46251

TGCAGCCTCATTATGTTCTCTAACCCAGAGTGGAAAATATCTGTGGCATCGACATGGGCATTGTTCGCGCTCTTCTTGGTGGTCCGCCCGCTGCCC

>596

TGCAGCAGGTTTGGCCCACTGCTTGTTACTTAGAATAGGAAAAGCCTCTTAGTAGTTGTTACTGGGGTATTACACTATCTGTTCTACTCCCAACCA

>73650

TGCAGCTCAAACCAGGTCAGAGGAACCGCACTCTACCTGCCGGTTCTGATTCTGACTCGATCCGATTCATTTTCAGCCACAAAACAATAAATATGC

>25002

TGCAGCGGGAAGAGGTAGACGTACGGGGAGAACATGGGCATGGAGTACGCCTGGAACACAAACAGCCAACAACGTTTGTTTTAGCTGAGAACAAGA

>37381

TGCAGCCTGAGGCGAGTCAGTGGGAACAACAGGCTGCGTACGTTCAGCTCAGAAAGTTGAATATTTCTGCTCCTGAATTCAGGCAGAAAACAAAGT

>110605

TGCAGCCGGCGGGCCGCTCTGCGGGGACAACCGCATGGAGTCCGCTACGGTACAAGATGGCCGACAGATCCATCGTTACAGCACAGACAGAGGAGA

>112224

TGCAGCCAGTCCAACTATGGCTGCTGCCCAGACGGACGGACCTCAGCCAGGGGCCCCCAGGGCCTGGGCTGCCCGCAGACTCCAGCTGCCACTCCT

>209725

TGCAGGGGGCGCCGAGAACGGCCTGCTGCCCCTCCCCCACGGCGCCTACATCCATCAGCACGGCGAGCGAGCCAGAGCCATGTCAGCGTCTGGGAA

>176688

TGCAGGAAGTAAGACTAAATGACACGGAAGAGCCACTTGTAGAGGAAGAATCGTAAGTCTGTAAACCTGTGCTGCTCTCTGAAATCACCTCTATTA

>136192

TGCAGAGGCGCTGAGCGGCAATCATATGACCCGTTCTGTTTGACAACAGCCTGTTTTTACCCCAAAAACATCTTCATGAGTCGCCAGGCGAACGCA

>173748

TGCAGAAACTGATGAAGACTCAGATATTAGCATCCCCTTTCAGCCCATCAAGTCTAAAGTTGGACAAATTCAAGGAAACCCAACAATACCAGATCT

>185739

TGCAGCTGCTTCTGGGTCCGGCATTCATGGTAAAGTGGTTCTGTTGTAAATAAAGGCTCAGTAAGACAGAGGAGGTTTGGATCCAAAGTAGCAATG

>138163

TGCAGGCAGGAACACAGGACTGAAATGAAAGGAGAAAGGATGGAAGTGATTCATACTCAAACTGTTGGGAAATACTCCCGCTGAAACAAACACAGG

>181673

TGCAGCGGCGCTGTAGCACAGAGCCCGGGCTTTCATTGGCTGAGCTCGCTCTCTGCACAATCTCAGGGTGCTGAGAGCATGTGGGCACATCTTTAA

>329714

TGCAGATAATAGGCTGCAAAAATCTCATTGTTCTTTGATGCTTTGGCTTTTGCAGAAACTCCACCATCGCTCCATCACCCGGTCCTACAACAGATC

>193775

TGCAGTTAGCGGAGCCGGAGGGAGCGCGAGACGTGGACAGGTCAAATTTATACGGAAATATGGAAAGTGCCTGTTCGCCGCCTTCCAAGCACCACT

>306389

TGCAGCATTTCAGACAAACAGTTTTTTTTGTTATTTGTCAGAAGGACATTGCTTTTGTTTCCCTTTGGTGCTGGAAAAAAACAGACTTACAATAAA

>154424

TGCAGGAGCCGCTGTGAGCCGGAGAGACGGGAGCATCTGGGAGACTGAAGAGTTGGTATAGATGGCGAGCTGAAGGTCCAGGTCAGACAGTCCAGA

>118364

TGCAGGGCGTGAGCAGACACTCCTCCACTATCTTCCCTCACAGTTTGGTTATCAGATGACAGCGCTAACGGCGATGCTAGCAGCTTCCACAGATGC

>124676

TGCAGTGCGGGGAGGTGGTGTGGCGCTGGGGGCTGCTGAAGAAAGGCTACGGCCTCTGCCACGGCGCCGCCGGCAACGCCTACACCTTCCTGGGCC

>246370

TGCAGGCCGCGGATCCCCGACACGCGGATCCTGAACCACAGAAAGTCACATTTCTTCATCATGAGACCCGAACAGAAAACTGATTTCTGTCTTTAC

>235047

TGCAGGAGACAGAAAGAGGAATCACAACACAAAGCTGGGGATCAGAAGCTGGTTTAGTTCTCAGAAAAACTCAATCAGAACAAGACTCAGTTCTTC

>159619

TGCAGCTCCTCCAGAGCTACAACAATTCTCTACTTTCAGTGGACGGCCATGGTTGATGGACCGAGCAGCACTGATGTTCTGGGACAGTTGCACTGA

>233813

TGCAGCTGTAAAGGTAAAATCTTTCAGAATTACTAATCTAATATTCCATCAATTTTATTGACGATAACTTCCCCAGAGGGAGACAGTTTTCACGCC

>22007

TGCAGCGCAAACCAGCCGGGTGTCGGTCGCTCTGATTCGCCAAACCACGCCGCCTGAGGAGCAGCGCAGAGTCAGTCAATCAATCAATCAATCAAT

>66749

TGCAGCAGTCATACATCACACCTTGCTCACAGTTCCAGTTCACACCAGTCTTCTTTGCTCCTGTGGGTATGTACTGTATGTGTTACAGGGCTTGAA

>4205

TGCAGATTTTAGGGTCTCTAAAGGGAATGCAGTGGAGTATCCAGATATATCTATTATACACAACTGCGCTGAAAGCATCTCTTATCTAACATGCAC

>87425

TGCAGCTCTTTGGTCTCGGCTCTCGATGGTCCAGCAGGAGTCGGCGTCTCTTCATTCATTACATCAGAACTTTGATCACTGCGAACAGACATTTTA

>29

TGCAGCCAGTACACCCACTACAACTACTGCTGGAACTACAACAACCACAACCACAACAACAACCACAACAACCTCCAATGCAGTTCACAACACATC

>332191

TGCAGTAAAACCACAGAGTGTTGAGAACATTGCAATGTTTTCTGGATAACTGGACCATGTTGCTGGTATCACAATGTCGGTGTGTAGAGTAGAAAA

>90150

TGCAGGTGAACCAGGCGGAGAAGCAGAAGCAGGCATGGTGACAGCATGAGGAGAAGAGTCTGTACTTGAAAGCGGTCGTGGCGCCCTGCGTTGTGT

>179700

TGCAGAGGCTTACCTTCCATAATCAGCGTCCAGGATGTAGCTCATGTTGTGATGACCTGTGGAAATCAAGCCCTGCTGAGTTGCTGTTCTGCCAGG

>29714

TGCAGTGTATGTGATAAGAGTCAAAAACAAGAAGAAAAATATGAGTCACACTCTCTAATCTCTTCCCTTCCATCCTCACCCGTGGACTATAAGCCT

>26132

TGCAGTTTGATTGGGTAGAAAATCGGCTTCAGGTGACAAAACAGGCTGGAAACATGCAAGGTTACTCTCCTCTTCAGATATCAGGTAAACAGCTAA

>173243

TGCAGGACGGAATCAGGTTTCCACATTTGTTTCAGCCTAAAAATAAAAACTGCTGCCTTCATCTCTTCCTGTTACTAAACCACCTCCGATGTTTCT

>200978

TGCAGAAGCCCGACAGAAGGACTACCGGTGGCGGTTCTCGCATGAGTGGCGCACTGGGCTAGACCCTTTCAAGAGCCAAAAGAGCCTCAAATTATA

>243745

TGCAGGCCTCCGTTTCCTCGCTGCTACCCCCGCCTGCCGGACCCGGCCAACACCGAGACGAACGCAGCAGTCCTGAAGCGGCGCCAGAAGCAGATC

>7718

TGCAGACAAACTTAGGATGCCATGTGATGAATCAACACACTGTGCAATTAGAAAGTGGAGGGACTCTAATCCTTAGAGTCAACGAAAAGTGATTAC

>71386

TGCAGCGCCTCGCTGTCCTCGGCCGCCTCTGCCGCCGCCGTCTGGGGCGGCTGTCTGGATCTCTGACGCTGCGGAGGCAAATGAGTGGCACCCTGA

>154956

TGCAGTAGACACCTCTTTTGCAGACATAATGGGCCACTCACACTCGGGGAGGTGCAGGAACTTTGGTCCTTCTTGCCACTCTGAATTAAGATCGGA

>134521

TGCAGCAGACGTCGCAGTCGCTGCCGCGCTTCATCAAGGATTCGCTCAAACAGATCCTGGAGGAATACGACTCCCGGCAGATCTTCTACTTCCTGT

>94514

TGCAGAGACAGAAATAACAGCAGCCAGCACAGACTTTGTTGAATCAAAGCTTGTGTGGATGTCCTAAGCTGATGCTTCTTGAACCAATCAGATTGT

>267139

TGCAGATGCGTCTTTCTGACATTGTTTGCAGTAATTGGGCACAGACTGGAAAAGCCAAAATCCAGTCAGTGCTGTAGACGGAGGGCAGAAAGCCAT

>93486

TGCAGTGGGAATTAGGTTACAACACCTTGTGTTATGCAGCTGTGAGTGTTTATCCTGCTGTCTGTTTGTTTTCTGATTTCATCTTTTCATATCTTG

>90894

TGCAGCTCGCTCCAGTCAACTCCAGCGTCGGACTCATAGAAGCTACGGATGTTGATTCAGAGCCGCTTTATTATCGCTTGGAGTCTGCAACTGTAA

>39852

TGCAGCTGTTGGCTCTGAAGGTCGCGACCGGGTCGTTTTCAGGTCATGTTCAGCTATCAGAGCTGCTGCTGCTGCTGCGTCACGCTGACATCTGAC

>182232

TGCAGTCAAAGGTTGAATTATTGAAGGGCATGCACTTTCAAAAACAGTCTGAGTGAAGTCCGTCGAAGATACAGCAGACTATAGCCTTTTGGCTCG

>229628

TGCAGTACTCAGAGTCATCTCCACAGGAAACCAGCCAGAAATAAATCTGGCCGGCTTTGTTTGAGGAAGTGATGCTCGTTACCGTGGCAACGTGTT

>180062

TGCAGTTCCTGCTTGTGGCCACCAGAGGTCGCTCTCAAACCACACATTACACAAAATTCTTTGACATTTAGATGTTGTGACATCAGCGTCCCGTTA

>214978

TGCAGGAACAAGTGCCAGAGCCCGACCTCACAGAGGTGTGTTTTCCATGTTTTACTTGGCTGTAATTGTAACATTCAGATTTCATAACACAAACAT

>193075

TGCAGCCTGAGCTGCCTCGCTGCTCCGTGCTTTTTGTGAAAACTCTCTCAGCGCCTTCGACTGGCACAGGCTCTGTGCCTCGCAGTGTCTGAATGC

>336368

TGCAGTGCAGAAAGGGCTGAAATGGAGTCGCAGTTCTGAACATTGAGGCTCCGTTTGGAAAGTTGGTGAAAAATGAATAATTTAGAATGTTTTATT

>22334

TGCAGCACCTCGGTCCTTACGGAAACATCCCAAACATCGTGGCTGAGCTGACAGGCGACAATGTTCCCAAAGACTTTAGCGATGACCACAGCTACC

>270386

TGCAGTGACTGACAGCTCGGCTGCTCCAAGGATGCATCTTCTTCTGCACATTAGATATGAAATGAAAGGAGGAATTCATAAATTATTTTGCTAAAA

>250670

TGCAGTAACGCTGAGCGTCGGCCCAGGTCATCGGCTCATAGACGAAACGATATTCATGAGACGGAGGAGAAGAAACGACGCCTGAGAGACGCAGCA

>268423

TGCAGCCCTTTGCTAAAGTAGTAGAGTCTGTGCTATTGGTGAGACACGTTCACAGCAATGGTTTCACCACTGGTGGTACATGTGGCCTAAGGTGCT

>17876

TGCAGCAGCAAAGACAGTTTTTAGAGTCACAGCTGTTCAGTAAGACACTTTGTCTAAAGAGTTTCTTCCTGCCGGGAGCCAGAGGAGCAGCCAGAT

>294431

TGCAGTCAGCAAACACGCTACAATACAGGCAGCAGTCTCACTGACTATCAACACAACATCTGTGTTCACAGGAAAGGAAAGCATGTGGGTTTTCTG

>144507

TGCAGCAGCTGGACATCTGGGCCGGACTCTGGTCCAGAACCAGGTCCAGCCTGACATAAAGAACCAGAACTACAAGAACCAGAGGAGGGATCAGAA

>128260

TGCAGAGCTTCACCTTGTTCCCCAAACCGTTGGACAGCAGAGAATGTTTGATGGCTCCGATCCGTCCATCCATCATGTCAGACGGAGCAATGATTT

>144605

TGCAGCTGCTGCTTCCCAAAATAAGAGCGTCAGTGAGGTGTTGCTTCTGTCAATCAGCCCAGTCTGAACTGAGGGGGGTCCAGGTGCTATAAATAC

>107405

TGCAGGGGTAAGAAAGAGGAGAGGAATGTGAGGAATGATGCACAGGAAGCGAAGAGTCAGACAGGTTGGGCTTACAGGAAGAGGAACTCTTGGCTC

>106179

TGCAGCAATGAGCAGACTAATCAGTTTTATTGAGTTTGGCCTTGACAGCACGCTGTGCTTTACAGATATCTGACTGTGTGGCTCCTGTTGGTGCTG

>39663

TGCAGCGTGTCAGGAGACGCAGTACAGTGTTGCACTGTGGCACTTCTCTGTGAAGGAACATAGTGACATTGCTAACATGTCCCTACGGCCAAATTA

>92781

TGCAGTTTTCCCATCTGAAACATACCCATAGGTCAGCATGCAGGGTGCAATGTAACTCGAGGGAACATGCTTGAATTTATCATTCTGGGAACAAAA

>156633

TGCAGCATCTTGTTGCTCAGCCGGTCGATGATCCGGAGCGACGTCTCCCAGAAAACGTCCTTGTTTGAGTCGATGATGAATGGCTTGAGCGGGTAG

>244560

TGCAGCAAAACTGACAAACCATCCATCACATCTTCAGATTTTGAATGTCTGTATACTTTGACTAAAATGTGTATTTTCTCCTTTCCAATTGTCTTT

>325343

TGCAGTGAAATCTCTGTCTCTGTGAAGCTGGGAAGAGTGGAAGACAGTGGCCAGGTTCGATGTCACCGACTCCCGGCTCAATCGATCAGCTCTGCA

>221103

TGCAGCCGCAGGTTGGCCGCCTTCACTTCTTCTTCCCTCCTCTGGTTGATCTCGTCAATCTTCGTCCTGAACACAGACACAGCGGGTCGGACCGGT

>112320

TGCAGGCAGAGGTGGGTGATTAGGGCCTCCCTTTATGTTCTGGCCACAGGTGTCACCTCCACCACCCCCTCCGTGCCCAACCAGGCAGCGAACAAC

>77351

TGCAGGAGAAATACACATGCATCAGCGCTGAATGATGGCAGACAGACCGAATATGCATCTTGGTTCATCTCACCAGTAAGACGAACCTCCACCCAC

>26898

TGCAGACAAGCTGGTTTGTCTACCGTCAATTCAAAGGAGAAGCAGGAAGTCGACGCAGTTTGTTGCATAACGGATGGATCAGTGGAAAGTTTCAGT

>272969

TGCAGATTTTCCAGATTTCCCGTCATCGCACTGTGATTGTTTTGGGTAAAGATGGTAGGAAAGCAAAGAAAGATGCTCAGAGGATGAGGATGTGAG

>296641

TGCAGCTGCCCGTTTCTTGCTAACGATGAAACTTCCTGTGTGTTTTCCAAAGTGCCAGAGTTTCTGCTCGCCTCATAAATGATTCATGAACATTAA

>205363

TGCAGGGAATCCAGGCAAACCTGTGTCATGCTGCCAGACATTCATGCACTGGGACTGTTTGGTAATCCTGCAAATTTGTCGTCACTCATCAATTAT

>320837

TGCAGTTTGTTCCACATGTTTTTCCGCCCTCATCGCCTTGTTTACCCCTGTGCGTATACATATTCTTACCTGTGTCACTGTTCTCTGCCGAAGTCT

>51739

TGCAGTTTGGACTGGATAACCTGGACCTCATGATCCCCACACAGGGCAGGCTGGTCATTCGCTGTGCTCCAACAGCTGTCATAAATGGTACATTGT

>332045

TGCAGGTTTACAGCTGTTCCGACTTCTGTCCTGATGAGACGTCCATAAAGGGCGTCACAATGGTTCTCTGGAAGACCGTCCACTAATGAAAATCCG

>113971

TGCAGCTCGTCCCGTTTGCTGCTGCTTTATGGTTTTTATCTTCTTTCTGAACACAAACCAATCCCAGGCTTAGCGCTGAGCTCTTTCCTCACCAAA

>119767

TGCAGGACGACAGTGAGGACAGCACTGAGAATAAAAGCAGACAGAGTGAGGAGAAGCAGACAGAGCAGTAAGTTCTGCTAGCTTCAGCTCAGCTAG

>219687

TGCAGGAAGTGACATCATCAGATCAGAGGCAGACAGACATGATGTCACACATCAGATTTATGTCCTCTGAGGACAGCAGGACGTCCAACAGAGAGC

>97183

TGCAGCCACCTACTCCTTGTGGTGCGGCACCACTTCCTCTTGGAAGAAGCGGCGGACGCTTTGCCTGAACATGTCGTGGTCCTCGTTGAAGATGCG

>134476

TGCAGCAGGTAGCGACCCGCCGGGTCGGTTCCGTCCCGCTCCGGGCCTCCCGTCCTCCTGTCCTGTTTGAGTCAGAGCCGCTACAGGAATAGCTCC

>281490

TGCAGCTCCTCTGAGGTTCTTCACTCTCATGCATTTTACAGCAGCAGCAGATTAGAAATGCACATAATCTGGATGGAGGGAGATGAGGCTGACGGA

>166842

TGCAGATCATCTTGCATATGAAGAGACAAGTAGTTCTTAGTGGGGTGTTTGTGGTTGTGAGATTATGGTGAAATCTGATCTAAAAAGTGACTTATC

>144221

TGCAGAGGCAGCCATGCTGGAAAAGCCACAAGCAGCTCCAGGCGAAGTGGAGGAGCATCTGCAACTGTCTGACTGAGCAGAGGGAGGAAAACAAGC

>146286

TGCAGCACAATCCAGTGTTGTGGGATGTTTATCCACCACAGCATCATGTAGGATATATGAATTTCACAGCACTATCACTACATTGTTGCCCAGAAG

>43461

TGCAGTGTCTTTGCGCCTCTGGATTGAAGGCTCTCTGGACACATCAGCTCTTGGACAGAGATAATAATGGTGCTCTGCATTGCGTTGCTCCTTGCA

>21875

TGCAGGTCGGCCTCACAGCTTCTCCTCCTCCGTCCTCCTGGATCTCCCCGCCAGCCGCTCCGGTTCCGTGGCGTTCCGGTTCCCTCCAGGCGTGGA

>139901

TGCAGCGTGACATGTGATTGCTCTGACCTCCTTTGATAAAGCTCACTCGATGTTTTGTTCTGTCTTGCATAATTGGCAAAGATTCAATAGGAAATG

>150460

TGCAGCTGTGCCTATGGCTATGACAGTGAAAGAGTTTACCATGAGTAAATTCTGCTGGACTAAACTTGGCACGAAGAGCAATCTGGAAATGGAAAA

>114216

TGCAGGAAGTCCAGCACAGAGAGTTACAGGTTAGGTTTATCCTGAAACACCTTAGGAAGCAAAGAGAGTTTCATTTGTGTCTGGAGGAAGATCCAC

>138757

TGCAGCGGCTGAGTAAACGTCGGCGCTTGTGTTGACATCACGGTCGGCACCCTGAGGACGACAACACAGAACCATCAAAAACAAGACGCTCCCCTT

>173401

TGCAGAGACTCATCACTTTTATACAAAAAGACTTCATAATGCTGAAAGTCTCCCTCGGGTGGGGACCACTGGAAAGACAGGCTGGCTGTGCTGCTG

>126335

TGCAGACAGAGAGCACAGCGTCCTGAAGGAGCCGGACCGGACGCTGATGAAGGCTACGCTAACGCTAACGCGCAGCAAACCGATCTCAGCTTATCG

>297128

TGCAGACGCGTCATAAATAAAAACAACCGCAGCATCAGCATCAGACCAGATCATTTGGTTTTATTCAAACTGAGCGTCTCTCTCTTCACTCAGGCT

>283626

TGCAGCGTTTTGTTGCGTCTGAGGAGAATTTAGATTTCGTTTCCGGTCAGGATTCTGTTCCTGTTGGGTTTTTGTTTCTGAGCCAACAGCAGGACG

>257223

TGCAGAGAACTACATCGCCGTCGTCTCCAGAGCAATGGGCCATCCGCTGTCCTGTAACTCCACAGAGACCCGGTGCACTGTGGGAGGACTGCAGT

>140674

TGCAGCAGCTCATGCTGCTGAAACCTGTCGGCAAACAAACATCGCTCACATTGGCAAAATGATCAAAACTTTGCCAAATGCAACAACGTACACGTA

>75356

TGCAGGAGGTAAAAACCCTCTGATCTTTGACCTTTGGCCTGAAGCGTGAGCTAACCTTTCAGCTTCCCTGCTCCAGATCTTCGATGGACAGACGGT

>186149

TGCAGTAAACATCGATCGGTACATCTGAGGCTAAAATCCCCCAAACATCCTCTAATGGATCTGATCGAACACTCAGCTTCCAGTCAGATGTCTTAT

>34844

TGCAGAGCCGCGGATCAGGTCAGTTTCAAACACGTCTGGATGTTTGAGAACAACGTTTGTAGATCCAACGAGCGTTATCCCTTCCTGAAGAGGAGC

>160968

TGCAGAAAAGCTGGTGAAGTTATAACAGTTACCAGGAAACTGTGTTGCTGCTTGGCAGCAGGTGAGCAGATTTCACTATATCTTTTCTCCTCAAAA

>67265

TGCAGCCCCAACAACTACAACGATGGGTCCAACAACTACAACTGCAACCCAAACAACTACAACGATGGGTCCAACAACTACAACTGCAACCCAAAC

>334136

TGCAGTGGTTAGCTGCTGTGGTTAGCTGCTGTGGTTAGCTGCTGTGGTTAGCTGCTGTGGTTAGCTGCTGTGGTTAGCTGCTGTGGATAGCTGCAG

>211252

TGCAGATCTGTGGAGGGACAGCTGGTCCAAGAGCGTTTCTGTCTGTTTGAGGACGGTTTGACTTGGTCTAGAGAAGTTTGGAAATCGGTTGTTAAG

>50923

TGCAGTTATCCCTGTGTGGGGGGGAGGCACCTTTTTTCCTTCCACTCAACTTTGCGTTTGCATGTTTGTCCTTCATGTTTTAGATATTTCCTGCAT

>67123

TGCAGCATCCCATCTCGTCTCTCTGTCGCCGCCCTCCTGGGCGAGTTGCTGTAAAACTGATCATTTTTCCAACCTGATGTATTCAGTGAAACTCCA

>317314

TGCAGGATGTGAATCCCGGCGCGCATACAAAACACAAATCTGATAGAAACAATTTACAGCGAGCCGGCACTCCCCTGCGTAAACGACGTCACCGTG

>220529

TGCAGTCATCACCACACTAGACTCTGGGAGGGAGGAAACCCAGTGAGCTGTTCGAGTATCACTGTCCCGCTCACCTGGAACCTCATCATCTGACGG

>23314

TGCAGATCGGGGATAAGATCCTGGCTGTAAGTGGACCGGTTCTGGTTCTGGAGGCCCAGCGGGCCGGGTGTTCAGTGGTAACGGTGGCGCCCCCTG

>129785

TGCAGCAGGGCTCAAGTGAGACCAACATACTGTAGAGACTCGTGTGACAGGAAAATGAAAAGAAAGAAAAACACCTGATGAGCTGTACTATACGGG

>287376

TGCAGACAACATGACTTCACATCTCTCACTGATTGTCATCAGCGATGGCTGCTGTCCTTCCACCAAACCACTACTGAGTCTGTACTGATCTCCCGT

>126648

TGCAGCTAAATAAAGCAAAAAGGCTCAACACCACATTCAGAGACATTCCTGACAATTTGCAAGGAACCTGGAATCACAAATATCTCAGTTCAAAGG

>6476

TGCAGCATATGAACACAGTCCGGCATTGGAGGACGCTCACCGCAGGTTCCGGGAAGCTCGTCTGTGCCGTCTCTACAGTCGTCTGTGCCATCACAC

>98871

TGCAGGATAATCTTATTTATGGTTTTCTAAACGGTAGCTGCTCTAAAAGAGTCACATGTCACTCTCATCAATATCCCTGGGTCCTTAGTGGCACCG

>67054

TGCAGGAAATGTTCTGACAGGGCTTCTGGTTAGTGTTTGGTCTCAGTAAAGGTCTACATCAGACCTTGTTGAGATAAGTACCCGACTCTCATAAAT

>323724

TGCAGAACAGAACTGACCTGTTCTCTAGAAGGTTCTAGCTGGTTCTGACTTTCCCAAGCCGTTCAGAACCAGAACCTGCTGCTTCTCAGGTAGATT

>32306

TGCAGCGTCTGACCCACGCTGGCGTGGAGAACGTCTCCGTGCGACGGCGTCCGGGACAGGAACAGGGGCAGAAGGTCACCGGCCACGCAGCTCGAT

>135677

TGCAGCCACAGTCAGCATGAGGGACACAGACCCCTCCACTCAGGAGGTAGCCTGAATTGCAGACACAGCTCTCCTGATTAGGCAGGGGGCAGTTGT

>241926

TGCAGGCGGGTGTCAGGAGTAAGTACAAATGTGATTCAGTGAAGCCATAAAACGAGTACGGGTCAGGGAGGGAGATGATGTGGAGGAACAGCATTA

>259815

TGCAGCAAATCTGTGGAGCAGTGTGGCTGGATAGGTCTGGCTTCAGTGCTGATCTCTCTTTTGCTCATGGTGTCGTACTTACAGCTGTAACTGCGC

>8168

TGCAGCTGGAAGCAGGAAATGAACAATACTTTATTTCCCTTTCTCTAAGCTTTTCATCGACGTCTTTCTTCATAGCCCTAATGTTACATGCTCCAA

>165488

TGCAGTGGGCCTGGACACTGGGAAATGTCTGGATGGTCCTGATGGCCAGTCCGCTCTGGTTGGAATGACAGAAACATTACAACCTGTAAATACAGG

>17573

TGCAGACAGCAGAAGTTCTTTGCTCTCGGTTCGGGACAAATCGTCCTGAAAACCTCCCTGCTGAACTCTGGAGATGATGCCTTCCTGCCTCGAGTG

>279863

TGCAGGAAACGACACACAGTTGATTTCAGCTTCCGCAAGCTTTGTAGACCTTGAACTGCTGCATTTATCCATTTATCAGAGAACGAGCCACTCTCT

>303051

TGCAGCTCGAATCTGTTTCCATTCAGAGAAATAAATCTGTCGACTGCTTTCCGTAGCTCCATTCATTCAGCTTTAGACTCTGGTTTGATGTTTGAA

>141735

TGCAGTTCTCTGCAATCACACAGCAGGTGCAATGTGACACATAACCCAAGAGTCAGAAACTCAGTGAGTGAATGAATATATCCTCCCTCACCACCA

>163236

TGCAGTGGACATAAAGCCATGCTGTGCTGCCAGTGATAGGCTTGGCCAGCCTTAGGTGGAGGCACCGGCATGAAATTACAGAACCTGCCCCGAAAT

>251550

TGCAGAGCGACACAAGACCACGAGACACAATTATCAAATTTGAAAAGAAAAAGGGAAAAGGAAGGATTAGATCAAAAACGTTGATGAGTCTAATCC

>337665

TGCAGCTGTGCTCGGTCAGGTAGCCCTGCTGTGTAGTCAGGGAAAAAATGGCCAGGCGGGAAATGTCATTATCATCCAGGTCAATCATTGTTGCAG

>255715

TGCAGCCTGAAATAAAAACCAGCAGCAGCAGGAGTGGAAACAGCCGCGTCGCTGCCAAGAACCACCCAAGTTACCGCAACACCTCGCCGTGACGGA

>141382

TGCAGACTGTTATTTGCAAACACCAATAAAGTGCCTCTCACTGTGTCTGCTCTGGAGGGAAATGGCATAAAGGTGGTTATGTCTACTACTCTCTGT

>174173

TGCAGGAAGGAACAGAACTCTTGACTGATGTCCAATCCAGGCGTCTCTCTGTCAACGGCAGCCGAACACAGAACTAGGGCTGTTCTAAACGATTAT

>165451

TGCAGCCTTTAGTAACAACATGCTGTCTGAAATCTGGTTTTCTGAGGCTGTCAGTAAACTACTAAGTCAACCTGTCTTTATGCAGTATGCCGTCTG

>74046

TGCAGCTATGAGGTGCCATCGGATCTGGAGGAAAGCCAGGAGGGCCATGATCAAGATGTCCGGTCCAAGAGAAGGGGACGTGGCGTCCAGTACCTG

>256765

TGCAGGAGGCCGAGCGCCGGCAGAGCGCGAGCGATGAGTGACGTCACAGATGGACACGCCCCCCCCAGCATCAGCAGGTACCTGGGCCCCGCCCAC

>159921

TGCAGGCTCCCCAGCAGCATTTTTGGCCTGTTCTATCAGGGCCTTCAGCTGCTGCACGTTGCTCATCAGTGTGTTTGCCAGTTCCTCCAGGTATAT

>225594

TGCAGCAGAGGCAGAAGTGGAATCACCGTTCCAGAAACTATCAGGTCGGAGATCTTGTACTGATCGTGCATGAATGCACGCCTCGCAGTCAGTGGC

>61959

TGCAGAAGCTCTAGAACTTCCAGAACCCTCCAGAACCCCAGGCGTGTGCAGCGCTGAGACTTGTGTTATGCATGCATGTAACTGAAAACAAACATC

>193863

TGCAGATGTCAAAGCAAGTTGATCTGGTTCGATCCCGGCCCGTTTACTGCATGTCTTCTTTCTCTGGCCTTCTTAGTGTCTGACTACTAATACATG

>140912

TGCAGCGCCGTCACCAGCGTAGGCTCTCTGACGTGGTTGGTGTGGCCCAGACCCAGCTGCGGGATCGGATCAACAGAGCGGGTCAGGACCGGACCT

>225959

TGCAGCGCACTGCGTGCAGGGCAATGCAGACGTCACCATCATCGTCACCGTCGTGATTTGACTTGGTAGCCAAAACACCAAGTCCAGTGTGGTAAT

>63241

TGCAGCGTAAACATCTTCAGGCAAATACTTGCTCTCAGTGAGACCCATTGTGTTCAGTCACTCAGCATTCATCCGTTGCATGGCTAATAAAGACAC

>182712

TGCAGGAACTCAGTCAACTCCCGTTCCTCCTGAAGGAGCTGGAGTAACTGCCCGACATCAGGACTTTACTGTCCTTCAGCCAGAAAGTCTTAAGAT

>9866

TGCAGGGGGGCAGCAACGAGAACAACCTGCGTTAGCGCAACGACGGCTTCCAGCTTGGAAGCAAACTGCGACTTTGTTGTAGTTGAGCAATCTGAG

>261519

TGCAGCCTCCTTCAGAAGACAATACAGCTACTAGAAGTCACTCTAGGTGTGTTCAAGATGACTGTTTGGTGTAAAATCTAACTTTTTTCAGTTTCT

>28137

TGCAGCAGGAGACGAAGCTCCGCCGCAGGCTGCCACCCAGCCGGCGAGCCCTTCGCACGGACCTGCGGTTCAGGTATGAACAGGAAAACTTTCATG

>181242

TGCAGAGAATGGCAGGACAAGATGAAGGGGAAGAGGCCTAATGGCTGGAGTTGAGCTGCCGGACCCCTCTCGCAATGAAAAATCACCTCAGGGTCA

>246307

TGCAGCGGCTCGGGTCAAACTGAGAATCTGTTTCAGGTCAGCCCACCGTGGTGAAGCTGGTGGACAAGGAGACGTTACTGAGGGAGAGAGAGGAGA

>159665

TGCAGCAGGTTACAGTGAAGTTATTGTTTCCTAGTGAACATCAGTACTTCCTGCTCTTTATCAAGCTGAATGAAAGAAACTCCCTATAAACTCAAA

>36916

TGCAGCAAGGTGACGTTTCGAACGTTGGCTCCTCATCAGACAGAAAGAGGTCTGACGAAGAGCCAAGTGTTCGAAGCGTCACCTTGCAATGTCATC

>54367

TGCAGTCCAGAAACCACACAAGAGTCCGGCATGGCATCATCAGTGGAGCAAATGCACACTGGCAAAAGCCACATACAGGCTGCCAGAGAAGCGCAG

>271449

TGCAGAGGTCAAAGGGCGGGAGGCTGGGAAAGGTCACCAGTCCTTCATGGGATAAACAAAATAAGTTAGCACTTTATAATTTATCCAGTAAAATCA

>23961

TGCAGGTCCTGAAACTGAAGCTGTGAAACGTTTCAGTCTAACTGGGGTCAGCTGGCTGCTGTCTGTCTGTCCTCCGCTGGACGCCTGTCCTCCGCT

>83656

TGCAGGAAGCAGAACAGGATGGGTGCCAGCATCCAGATGGAAGTCCTGCGCAGACCATGCGGGTTTGGGCTCACTGAAGTCATTTGTAGTCTATAT

>142427

TGCAGGATGGATGGATGGATGGATGGATGGATGGATGGATGGATGGATGGATGGATGGATGGATGGATGGATGGATGGATGGATGGATGGATGGAT

>53826

TGCAGTCCGTGAAAGTCCTCACAGAGATATCTGCTGCTTTGCTCTCCGGCGTTCTGAACGACTGAGTGTGTGTGTGGTTGTACGAGGAGACGTATG

>186193

TGCAGAACGCGGGGCGGAGCCTGGACCGGAGTCCAGTCGATCAGAGGACAACACCGAGGCTGTACTGTCTGGTTGTTCCGTCACATTTTCCTCTAA

>48568

TGCAGCCGCGTTGCCGTGGCAACCAGCGGAGCAGCGGGACAGCAGAGCGTCATGGTGGCTCTGGAGCACAGCGCCGTGTGCTGAGCAGCAGGGGGC

>194874

TGCAGCTCTCACACCTCACTGCAAAGAAACTGCTATTCCCGCCATTCTTTGCCTGACGATCAACCAACATGGTTCAGGCTATCGCCGGAATGCTAC

>177952

TGCAGCAGCGTTGAAGACTCCAGAGTCGACTCCTCCCCTGCGACTTGGGAACACAGGAAGTAGTGTCATTACATCATCACTGTGACATCACCCTTT

>7132

TGCAGGTGTCCTCCTGCGCTGGAGGCGTGAGGAGGAGGCAGGGAAATTCTGTGCAGTGAAAACCTTTATTTGGATTTTTACAGCCAAAGATGTGCA

>127864

TGCAGAGCCGGAGGAGGCCTGCCTCTGATCTAAAGACGCATCATGAGCTTCATCAGAGCCAAATCCAACAGCTGGAAGCGCGCAGGCGGAATCTAC

>279429

TGCAGGTACAAACATCCACTAGGCATACACAAAAGTGAGGCATGGTCAGAATTAGATGTTTTTTAGCAGACAGCACCATTTTGTATCGCAACACT

>283618

TGCAGTCACATGACCGGTTCAGGGTTGCTATGACGACCTTGGGGGGAGGCATTGAGGCCCTGTGAGGTGGCGGGACGCGATTGGCCGGCTGAGAGG

>222374

TGCAGCATCCATCCGTCCATCCATCCATCCATCCATCCATCCATCCATCCATCCATCCATCCATCCATCCATCCATCCATCCATCCATCCATCCAT

>100408

TGCAGTGCCTCTATTCAGTCTTGACAGTGTTCACTCCTCTGAAATGCCCTGTACAAATACAAGTGAAGCTTATCTTAGAAAACAGCCTCTCTGATT

>5257

TGCAGCACTAGAGGTGATCAAGACAGGAACTGTTAGGCCAGGTCGACCTTCATGCTCGGAGCCCAAACGGCTGCTCATGTCTGATCTCTTTGGCTG

>147491

TGCAGTCTGACCACCTGTAACCTGGACACACCTGGACTCCATGGTGACGTCTCTGAAACACCACCTAGTGGACGGAGTGCGGTAATGCAGGAGAAA

>150784

TGCAGAGTCATGCAGGATCAGAAAGCTCCACTCGAGGTTTACTCCGCTCCGCAAGAAGATGTTCAGCCACGACAAGGACGTCGCTGGGATGATGAA

>246545

TGCAGAACCGAGTCTAGACAGTCAGTCTGTGGAGCGACAGCAGAGAGGAGAAGCTCCTGCTGACACCTAAAACCAGATTGAAGATAATGAATAAAG

>43472

TGCAGCCTCACATCCTGTCGACGCTCGGATGCCTTCATCCTGAGACAATACTTTGACTAGGCCCTGCCCGTACCTCGTGGTCGATGTGCAGCTTGT

>338493

TGCAGAAACACAGGGGGTCGAAGGTCAGGAGCCTCATCAGATGGACAATGAGGCTCCTGACCTTCCATCTGGTAGAAAACATTCACATTATTTGTA

>175286

TGCAGAACACACACCACGCACAAGCACGCGCGCGCGACACACACACACACACACACACACACACACACACACACACACACACACACACACACACAC

>231616

TGCAGCTGTTCTCACCGGGTCACCATAGCAACCACTGGCTGTTACCTGCGTGTTCCCTGTGTGTCGCCGTAGTAACCAGAGGCATGGCGTCCATCC

>33933

TGCAGAGAGACGGGGACGGTCGGCTCAGAGGGAGAGCGGGACGGGTTCTGGACGCTCCGGCCCGGTTCTGACGGAACCAGCCGAGGAGGATGACGG

>4625

TGCAGCAGCTGACCGAGTGGCTGGACGTCACCGAGGCTCGCATCAAGAAGATCAGCGCTCAGCCGCTCGGCCCCGACCTGGAGGACATCAAGCACC

>96175

TGCAGCGGACACAAATCACCTGCCTGAAAACCCCGTCTGACTTGAGCCACACAGTTCAAAGTTTTCTAATGTACTTTTTATTCCCCTTTACAGTCA

>291628

TGCAGGGGGACAGACCTGTCGTCCTCGTCCAGAGAAGACGGTGGTCAGTCCGCTGCGCTTCTGGACGCGTCTCAGAGGATAAGTCTGTCTCCTAGT

>43969

TGCAGTGCATCAAGGGTGGGAAACACCTTCATTTGAGGTATTTGGCCAGAAGATGGAGCCATTTTCTTTATAATCAAGCAGATGCAAACTGGGCTG

>94455

TGCAGGCTCGGCGTGTCAGAGCAGCAGCACACTTTGACATCTCTGCGAAAAAGACAACACCTCATTCATCAACGCAACGAGCTAAAGAAGTGTGAG

>51375

TGCAGTGTTCCTGACATTCTAGCCTCTTCATTTGGATGGAGTCAGCTTTGGCAGCAGTCACTGGCAGAAATCAAATCAGAGGTTTCCAGACCCTGT

>66836

TGCAGAGAAAACCATCAACAAGTCAGGATGTCCTGCTGAAGAATAGCAGAAGATCATAACCATCATTTATAATCTGCTTTTTTATTCAGTTTATTC

>151056

TGCAGATCGTCACACCACCACCACCGTGTTTCACTGTAGGGACCACTGAAGATGGGAATTTGCGTCTCTGCGACCCTCCAAAGAAACAAGATTCAT

>277390

TGCAGAGGTCTTCAGACTGTGGGACGCATAGAGAAACAAACAAAAAGGGCAGAACTGGGCCGCAGCGTTCCCACCAAGTGACATGACCCAGTGTGG

>266814

TGCAGTGTGAGCTCAGTGTATGTTCACAGTCAATAAACATGAAAAACGCAACATCTGCACTCAAGCATGCATGTGAAATCAGAAGATTTATCTGTG

>235045

TGCAGGAGCATGCTCTCCTCTACAAACCCTTATTCTGTGTGAGACATGAGGTTCTTCGCTAATTCAGTTACTGCAAAATTTGCTCTCGTGATATTC

>99174

TGCAGCCCTAATGAACTCCTGGGCCTTCTCATCCCAGGACTGCAATAGATCAGCTCATGTTCGCCAGCGTGCTGAGACGCACACATGCATAGGGAT

>79735

TGCAGTCCCTGATCAAAGCGTTGCCGAAGTATCCTTCCTCGCACCGGGAACAGGACGGCCCGTCCGTGTGGTACAGGCACCTGAGGCACTGCCCAG

>169746

TGCAGGTGGAAATTCAGTGCATAAGCAGTTGTGTAGAGAGCATCAACAGGCTAAAACACTTTAGCAAATTCTAAGATTAGTTTCCTTTCTGACAGA

>272668

TGCAGGACGTTTCATTCGGATGCACAGACACAACCTGCTGGTTTTGATCAGCGAACATAAAAAGCTTGACTTTGAAGGACCTTTGTAAGCTTCTGA

>90455

TGCAGGCGAGGGAGGAGCAGCTGAGCGAGGGCGAGCAAGAGAGACGGCTGGAGAGGGAGGAGAGAGAGCGCAGCGTCGCCGAGCTGAGGGCCGACC

>90153

TGCAGCCGGTGGGCGCCGCCAATTATTCACCAACTTTTTCATCAGCGCGTCAGCTCAGAGCGCGGAGAGAGCGCTCCGCATATGACTGCGCAGGGG

>206343

TGCAGTTTGTGAGAGTTATCCGTTGTGTTTTATAGATTCTTGCCTTCACACCAGATTTGGATCAGTTCACTACGTCAAAAATTAGCAAGAAAATTG

>32238

TGCAGCAGGACTTATCAGCAAACATGATCCCTGCAACGCAACGCTCACTGCTAGGTAACCTCTGGCCAAGCTGAGACGCAGCGAGATGCGCTGATA

>42806

TGCAGCAGAGCTGACACGCTTCATCACCACTTTCTGTAAAAGATGTTTGTATTCCACAGCTCCACTGAAATACAGACATAATTATGTTGGGATTTG

>244031

TGCAGGGGTGTGCGTGTGATCTCACAGCCCGACAGGCTCATGTAATATTCAGCAGACCCCCGAGCCCCCTGGGTGCCACAGGTCTGTCCTGTGATG

>9355

TGCAGCAGGGCGTGCTGCCGGCTTTGTCCGCCGGGCAGGCTTTGGTTTCCGTGGCATCGCCATTGTAGGGGCCGGCATTGGCGGAAACGCTCTGGC

>195555

TGCAGCCCTTTGTCTTTGCTTCTTTGTACAAAACACACAGAGGGATGAGGACCATGTCAGTGTTTGCTTTCAATAAAACAGCAATTTGGTGGAATC

>177861

TGCAGCAGACAGACACATTCCGCCCCTGAAGCAACTGCATGAGGCGCGTTTCATCTGGTGCAAACCATCCACAAGAAAACATCCACAAGGAAACAG

>2866

TGCAGAGTGGGTCCGCCAGGCTTTGCGCGTCTCAGGATCACGACGTTTCTCTGTTCCTGGGCTCGGAAAATACAGTCCAGTCATGAGTAAAGCACA

>159151

TGCAGGGCTGGCAGGTCGGGGTTCTGGTTCTGAACCGTCTGTTGTTAGTCGGACCTTTTAGTCCAATCTATTGTGTTCTCCAAATTTCTGAGCTCC

>148366

TGCAGGCTAACGTTTCCCATTGATTCCAATGAGGAACGCTAGCAGGTTGAAACAGATCTTAGCAGACTCAGATCGTCATGTGACCTCCTGACCTGC

>225987

TGCAGGCTCCATGTCCGGCCGCTTCCTGCTTATCAGAGCGCATCGGAACCGTGTTGTGCTGGCTGGTGTTGCTCAGGTGGGCTCCTGCAACAGGAA

>116541

TGCAGGGAAGTGAGCGACACCGAGGTCTTCCTCTGAGCTCCGCCGGATCAGGACGGGAAACACCGCGGGATGCAAATCTCAACACACACACAGACA

>246502

TGCAGCAGGGGGCGCTGTCTCCGGCTCACTGGGCATCTTGTGTGGAGGTGCTCACCACCTTTCCGTCCACCAGCACTTCCTCTACAACAACGTACC

>143751

TGCAGTGCACGCCGCTCTGTGTAGCTCTGCCATAAGCTGTGATGTAATGGCGTTGAGACGAAGCCGCCGGGTGGTCGGGTGACCCGGACAAGCAAC

>208445

TGCAGGGGGCAAAGGGGGCAGTAAATCTGCGCCCGTGGAGTGGCTCTGAAGGAAAACACAGGATGTAAATCAGATAAAAACTTTTTACATCAGGCT

>37139

TGCAGGATCCGCTCCCTCACCACGGCGCTCAGCTCGTGCACCAGCAAGCGGAGGAGGAAAGGGAAGCAGGAAGGCCGAGACAGACGAGGACGCCGG

>17019

TGCAGGAGGAGCAAACAACTCCTGGTCCTCTGAAAGAGTCACATTACTTCTAAACGTCCAGAGCTGAGGCAGGCAAGCAGATGAAGGGGCTTCAGA

>51402

TGCAGGAGGATTGATGCGACTGGTATGATCGAGTGTAAAAATCTGATTGTTTTGACAGTGTGAGGCAATATGATCACTGCTCACTCTAAAACACGG

>58848

TGCAGCGAAGCTTTACGGTCGGATGAAAGATTGAACTGTTTGGCAACAGCACCAAGCAAGAGCAATGATGAAGTTGGACAACCTGGAAGTTCATTA

>255389

TGCAGTTTAGACAGTTCAGCTTTTACCTTTCCTGAATGTTTAGCAGCAGCTGAGCAGGAGTGAAATGTTGTTTCCTCTGCCTTCCAGATCCTCCTG

>339364

TGCAGACATCTCACAGTCCTATGTGAGCTCCACATAAAACAATCCTTTCATCCGTCACAGTGGGCTCACTCCATCTCCGAGGCTGCTTTGTGATCG

>281654

TGCAGGCTATGAGAAGGCCCGGCTGAAGAGGGAGAGGGAGGAGGCACAGGAGGCCAGAAGGAGGGAGCGAGAAGGCAAGGACAGGCTGAGGGAGGG
