# Supplementary material for: Identification of the male-specific region on the guppy Y Chromosome from a haplotype-resolved assembly
Source: Genome Res. 2025 Mar;35(3):489–98. doi: 10.1101/gr.279582.124 (PMC11960691; doi:10.1101/gr.279582.124)
Supplement: Supplement 12 [file Supplemental_dataset_S2.doc]

>ab.1

TGCAGGTCTGACTTGACTAAGTGGAGGTTTGTGTTCATCAAAAAATGGTCTCTGAGCAAACTCCAAGGTCCCAAACCATACTCAATAAAAGGAA

>ab.2

AGCAGAGAGGAGAAGAGACAATCAGAGCTCATCCAGGGCTCAAACGATTAAACATGCACTGCAAAAACACTGAATCCTACAGAGTAATCCTGCA

>ab.3

TGCAGGAAACCAAACCTCCACGTCTCGACTGGAGTCGCCGTGGCAACGCCTTTCAAACATTAGGATAGTGGGTTGAGACTGGATGAGAGTTGTT

>ab.4

AGATGGTTGAATGTGAGCTGCTTTGTTGTCATGAATCAGATGAAAAACACACTTACACTTCCTCAGACCTGGTGTCAAGAATCGGCCTCCTGCA

>ab.5

GAAGTGCTGGCAGACTGCTCCTCTTATACTTAAAATTGATGGGAGTAAAGAAGTTACAGGTGTGTAAAGGTGCAGGAGTAAGGCCTATCCTGCA

>ab.6

ACCTTTCAGAAAGTAGCTGTGAAGCTCTGTCCTCAGTTCTCAGCTCCCAGTCCTCCAGTCTCACAGGACTGGACCTGAGTAAGAACAACCTGCA

>ab.7

TGCAGGGCATCGCATGAAATCCATCTGGTGCTATAAACAGAAATGTGGTGCTATACCTAAAACTAATAATAACAAAGAAAACAAAACACTAAAC

>ab.8

CTTCAACTGTTTTCCAGATGCTACGGTGCTGCTAGTACGTTGCTAACGTCCGTGCGCGTCATGTTGGAGGGAGTATCTGCACAGTCCACCTGCA

>ab.9

TGCAGGCAGGACTTGAGTTTGTGGAAAGCCCTGAGCAAAAAAATCACAGAGATGTGAGCAGCGCACTACAGAGGAGCAGTGGGGAGACCTATGT

>ab.10

TGCAGGAGAAAAATGAAACGACAGATGAGCAACGAACTACAGTGTATCTAGTTTGGGAACTTTTTTTTGCAACTGTTACCTCCTTTGTAATGTA

>ab.11

TGCAGGTTTTAAAGTTTTGTTTTTTTTTATTGAAAGAAAACGATTTGGAAAAATTTTCTTTATCTTGATTTTTTGCTATTTATATGAATTCTTG

>ab.12

ATACATCAGTAGAAAACCCTTTAGAGAACATGCACAAGATATTTTCATCATATTTCTTTACTGAACTATTTGGTTTAAGACAGACTGACCTGCA

>ab.13

TGCAGGTAATTGTAATCTCCTGGCTCCATCTTGTGGACAACTATAGAAAGCAGCCCACAAAAATCCCAAAAACCCAAGTTCCTAACACATTTAT

>ab.14

TGCAGGTATGACAAGATAACTTGTTAGCCGCTGGCTGCTCACCACCCCGCTAACACAGAACCACCCTTCGTTCAGCTGTTGTGGAGAGCTCCAA

>ab.15

TGCAGGTCTAGTTTCTGGTGCAAACAGACTAAACTAACTTACTAATTTCCTCTTTCAGCAAGAAACATGAGTTCATTTTAAGTCAATATTTCCC

>ab.16

GCTTGTCATTTTATCACCTGGTCACAAATTCCAGCTGATTTAGAAGTGGTAAAACACCATTAGTGCTTGCCTTCTCTGAACTTACAAACCTGCA

>ab.17

TAACCAGGATTAAGAACATTGTACCTGAGAGTTTCCAGGTTGCAGTTTGGACTCTCTAGTCCAGCAGAGAGAAGCTTCACTCCTGAATCCTGCA

>ab.18

TAGTTTCTGCTGAAGCATCATTCACAGCATTCCGGCCTCTGCTGCGACCAGAGAACCTCCTGGCTGTAGCAGAACCAGGTCTGGTTTGCCTGCA

>ab.19

CTACCTTATCTAAAGCGCATTCACAGAATTAATAGACATTTATCTTTATTTTGTCTTCATGGCTGTTTTTGCTTAATCTGTGTTTATGCCTGCA

>ab.20

TGCAGGAAACGTGCAGTTTGAGCCCAGCGGCAGATTCCGTTTATTTAACCTCTATTTTCCTTAATTTGGAGTAAAAAAAAAAACACCCAATTCC

>ab.21

TGCAGGACAAACCGTCATCCACTGACACCATGTTTCATAGTTTGTCCAAGAACTACATGCTCAGCTCCCCGCTGGGCTCGGTCCAGAGCAGCGT

>ab.22

CATCAGATGAATAATTAAATCTCTAAATTAGTCAACGCCCTGGGCTCTGCTGACAGGTTCAGCTGCAGGTTTCCTGCCTCTGATCGTCCCTGCA

>ab.23

CCGCGGACGAGTCGTCTGGGTAGAGCTGCACACAGGGAGAGAGAGAGAGTCACATGACTCCACACCTGGCTCAACTCAGACAAGCCTACCTGCA

>ab.24

TGCAGGATGGATGGGGAGCTCCAGCGGACTGGAGTGGATCGGTTCTTAGTGTCCTCACTCCGGTCACTCATTGGCTTTGTTTGGAGAATCATGT

>a.1

TGCAGGGGTCAAAGGGTGACCCCCAACTGCAAAGTCTGGAAGGACAACCCAGACTGTGACAACTTGCTTCAGGAGTTTCAACATCACACATCGG

>a.2

CTGAGTAAGCAGTATGCAGCGTACATCGATGCGCACATTAACACAAGGTATTCATTCATGGGCAGGACAGATAGAACACCTGTTCACACCTGCA

>a.3

TGCAGGAGTCGACCTCCACCTGTCCTGGCCCCACCGAGGACGTACAGATATAAACAACACACAAGGACACAAACCAGACTTCTGACAGTTGTAG

>a.4

GCTGCTCTGAGCGCGCTCTGTGGGAGTGTCCCGGCAGCACGCTCTGCATCAAGCCCTCCATGATCTGTGACGGGTTCCCAGACTGCCCCCTGCA

>a.5

AGAGAAGCTTCATCAGCGTGGTGTGTTTCCTTAGCGCACCTGAAGGGGGCGCGGTCTGGGCCGGGCCCACATCCAACTCCTGCCACCACCTGCA

>a.6

TGCAGGGCCGGTCCAAGGCTGATGGGGCCTGGGGCAGAATCTGACTGAGGGGCCCCATCAGCACCTCTAAGCTTCTGTGTTGGATTTAATCTGG

>a.7

TGCAGGGTAGATGTTTGCAGAAAATGGCTTCAAGTTTTCAGAAGCCTTATGGACACAGTTTCTTACTTTTTTTTTTTTTTTTCCACTAAAATTC

>a.8

TGCAGGAGAGCCGAGAGGAGCTCAGGCGGGTCAGGTATGGTCTGGTTGGGACCCCTGACCTCTGTGACCTCAGGTCAACACACAGCAGGAACGT

>a.9

TGCAGGGCTGGGGGCGGAGCCTGAGCCACAGGGGTCACGACCCACACGGACCGGTTAGGGAGGAAATGGAGAGCAGTGACCTGGGAGGGGGGCG

>a.10

TGCAGGTCTGACAGGAGAAGAGCAGCACAGCGGGTCAGAACCAGAACCACAACCAGAACCACAACCTTTTCCTCCCGTTTTGCTCAAACAGACC

>a.11

CTGATTGGCTGCTGTGGCTGCTGTGGGCGGGACCAAGCCTCTATTATGATAATCTGACCTGATCAGAGCTGCAGGCGGAGCTCCGCCCCCTGCA

>a.12

AGGAACGCCACACACAACCATCAGTCCTACGGAAGGTCACGACCCTGAGGAACGCCACACACAACCATCAGCTCCCCTGCTGGGTCTCCCTGCA

>a.13

TGCAGGGAGCGCTGTGGTCGCTTCTGACGGCAGATATGAACACACTGCTTTTTGAAATTGGGCCCCTGGAGAGTAAAGAACTTCTCAAACATAA

>a.14

ACCTCTCAGAGAGAAGCTGTGAAGCTCTGTCCTCAGTTCTCATCTCCCAGTCCTCCAGTCTCAGAGAACTGGACCTTAGCAACAACAACCTGCA

>a.15

CGCCGCGGACGAGTCGTCTGGGTAGAGCTGCACACAGAGAGAGAGAGAGTCACATGACTCCACACCTGGCTCAACTCAGACAAGCCTACCTGCA

>a.16

CTCGTCGCGCTCGTCCTCCGTGCCGGGGTGTTCGATCATCCACATGGCGAGCACCGTGATGTTCTGGGCGTCCGCTTCTCCCCGAGCGCCTGCA

>a.17

TGCAGGACTCTTAATGATGCCTATATAATTATTCAGACGTCCTGAAACGCTCCAACAACAACAAAGGATTTAAGCTGGATGTGTCAACTTCACA

>a.18

TATATTATTGCTTGTTTGTCTGAGTCCATCTCCAGCCTCTTTAATGACTATAATTCTATTTTATCCTGTTAGTTCTTATTTCAGCCTCCCTGCA

>a.19

TGCAGGGACATGAGCTTCACCTCTTTAGACATCCCAGCCTCTTTAGTTCCTGGAGAACTTTCACTGCTTCACCATGAAGATGCTGCTGGACCCA

>a.20

TGCAGGAGAAGCAGAAGAGCTGTTGTTTACCAAACCAGCGCCTACTTCAGAGAGTTTTATTTTTCAGCAAGTCATCGACTGAGAAGCACAACCT

>a.21

TGCAGGTGGAGAGGAACTGGATGTCAGGAGGCGTCTGGAGAACCGGCTTCATGATAATGAATTTTAAACTCTGGAAGAAAGCCGGTCTGTGGGC

>a.22

TGCAGGCATCGTAATGATTTGATGACCAAGTCTGTACAATAAGATTTAAGTCTTTGTATTTTATTGAATATTACATGTTGTATTATGTCATATC

>a.23

TGCAGGACGGGAGGCGGCGCTGCAGGCGGAGGTGCTCTGGCTGCGGAGGGGCCTGGAGGAACACCTGAAGGTCTTCAAGAACGTCTTCAGCAAC

>a.24

TGCAGGATCATCTGGAGTTCAGACACAGCCAGTCTGTACCTGCTTCCTCACACACACACATACACACACACACACACAGCTACACACACACACA

>a.25

CTGCGCTGGTCCTCTTCCTCTTGGTTCCTCCTCTTCCTGTTGGCGAGGAGCTCTGAGACCCGTTTGGGCCCCCGGGTGGGGGGCGGGGCCTGCA

>a.26

AGTAATTAGTCTTCTCTAAATTAAAAATGAGCAGCTCAGTTCTTCTCAGACCTGGAGGAACATCAGCCTGGAATCATGATGAACTGCTCCTGCA

>a.27

ATCTACAAATTATGGTTCTGATCAACTGATCTCTCAATGCAGCTGATCAAACTTCAGACGAGACGTCTGGACACAGAGAGTCTCTTGTCCTGCA

>a.28

GATTTTGGGCTAATTTAGAAATATTTGTAAGGAATTTTGCAATATTTGCATTTATGAATGGCAGTAACAGTGTAATGAAACACTGCCACCTGCA

>a.29

GACTTTGACTGTTCCTGTGAACTAAGCTCTGCTTCCTCCATGCTGTGGCCTTTGCTCTTCTTTTGCTCCACAGCAGCTGAAAGGTTTTCCTGCA

>a.30

TGCAGGGTTTCACCTTTTCTTCATGGCAGGTCCTGCAGACTTGACTTTTTATTCTGCTTTAGTTTACCAACAAGATGTTTAACACCAATTACGC

>a.31

GTTTCAGGGTTTCTGTCATTTCATGCAAAAGCAAATCAAACGTTTCCGTGAATCCGTCCACAATCACAACCTGCCGAACCTTCTGCGTCCTGCA

>a.32

CTGGTGAGACCCTGATCCTGTTTGGACCTTCAGAGTCCTGATCCAGTTGTTTAACGTTTGAACTGGTGTGTGTTTCCCCAGCCGGACGCCTGCA

>a.33

TGCAGGAGAGGACACGTTTGAGTTTGGACATCCGTCCGTCCGTCCGTCCAATTTCTCTGAGCAGTTTCATGAGTCTCAGCTCTGATGTTTTCCG

>a.34

TGCAGGGGAGACAGAGAGGTCACATGACCCCACGGACCAATCAGAACGCAGCTCTATGCAAACTGTCCAATCAGAACGTAGGTCCACCCCACAC

>a.35

TGCAGGCTGCTGCACGGCCTGATGGGAGTTTTTGACCAATCAGGGATGTGCTTCTGTGTCCAGGTGGTGACAGGTGAGCGCTGGCCCCGCCCCC

>a.36

TGCAGGTAATTATTTCTAAAGTACAAGTCACAAAACTTTACAGCATAGTACTCAGCTGGAAAGCGATTAAAATCGTTTCTTGTGAGGAATTATG

>a.37

TAGGCAAGGAGAGAATTAAACAGGCAGGCAGCGAAGAGTCAAATGGTAATTTTGGAGACTGCAGAGATTAACAGCTCAAGTGGGAGAACCTGCA

>a.38

TGCAGGCCTCCTCACCACATCCATGCAGGCGGCTGCCCTGACGCCCGGCGCTGGCAGGCTGCAGGATGCGGTCTGCTGAGGGTTGAAAGGCTGA

>a.39

GGAGTTAGAAGTTCTCACCAGCCTCTGTGTGCGTGTGTGTGTGTGTGTGTGTGTGTGTTCATGTTTTCTGCTCCCACTCAGTTTGAATCCTGCA

>a.40

TGCAGGCGGAAAACAGAAAAAAGATGAAACAGTCAGCGGATAGTTTCCACAGGATGTAAGGAAATGCAAAACAAAAGCATATCAGAGTTTCCAT

>a.41

CTGAGTAAGCAGTATGCGACATACATCGATGCGCACATTAACACAAGGTATTCATTCATGGGCAGGACAGATAGAACACCTGTTCACACCTGCA

>a.42

TGCAGGAGTCGACCTCCACCTGCCCTGGCCCCACAGAGGACGTACAGATATAAACAACACACAAGGACACAAGCCAGACTTCTGGCAGTTGTAG

>a.43

GCTCTGCTTCCAGACTCTGAGGCGAGTCAAACATCCTCAGCATGTTCCCAGAATCCACCAGGCAGCTGAGTTTAATTTAATCAGGAAACCTGCA

>a.44

AGTACTTTTGCATTTTTTACTTCATAACTCTGCTGCTGTTGTGTTGTTGTGAATTTGGAGTGTGAGCTGCATCTGATGGTCTGGATGTCCTGCA

>a.45

TGCAGGTGGGAGCGCATCTTGAAGCAGCGCCGACATTTGGGGCAGGCGTGGGTACGATACACGTCCTCCGACCCCTCAGAAAGGAACGCTGGAG

>a.46

TGCAGGACAGACGGCATGCTCCATGCACGCCTCCTCACACCGGCCCACCACCCGGATCCAGGTCCAGATCCAGGTCCGGCACGCTGGACCAGAA

>a.47

ACACAGAACACAAAGGGTGAACCCGACCGAGCCCGGACGGTCAGAGGCCAGCCGGGGTCGGAGGTTACCTCGATGAAAGCTGCCACTTCCTGCA

>a.48

TGCAGGTCAGACTGGACTCACTGGTCATCTGGTCGGTTCTGATCCGATAAAGGCTGCTGAGCTTCAGCTGGATCTACGGGAGGCGAATTTGGCT

>a.49

AAATTCAGATTTTTTTTTTTTTGCGCTGATGCCCAATTTTCCACAAAAATTGTGAAAAGCTTTGGGTTCGAGTCAGTGCCAACTCCCACCTGCA

>a.50

TGCAGGTAAATGAGCTCTGCTGCTGCAATTCCTTAAAATCAGGCCAAAGTACCATATAATATCCAACATGGGAATTCATTATGTGATGGTCTCA

>a.51

TGATCTCCAAGGAGTCTTTCCTAAAGGATGCGCCGAATCTTTATTGGTTTCGTTCACTGCCCTGGTTTGGGAACAGATGAAATAAAACCCTGCA

>a.52

TGCTCTGTGATGTTCCATACAAGCGTAAACACAAAACATTCCTCCAGTTGGCTCGCTGTGCCTGCCTGCCTGCCTGCCTGCCTGCCTGCCTGCA

>a.53

TGCAGGATGCAGCATTCAGGTTTTTTTTTTTTTTTTTCCAGTATTCATATTTTTGTTGTGTCATATCACTGATGAGTTTTCCACATAGCCTCAG

>a.54

AAAATAATAGTGTGGGTAAATATGAGTGAGAGAATCCCAGAGAGAGAGAGAGAGAGAGACTGTACTCGGCCTCACTGGCTCCTGCAGACCTGCA

>a.55

ACATACATGGAAGTATGAGCTCTGGACACAAGAGACAAATTTTTAACTTTTTTTTCCCCGTTTTTCTCATAATATTTCATCTTCTCACCCTGCA

>a.56

TGCAGGCCGTACAACATGAGGTGGATTAAGAAAAGAAAAAACTAATTTACTGAAATCAACTCAGAAAGAACAGAGTTCATCTGAAACCTGCAGG

>a.57

TTCATTTTCAACCCGGTCTGTTGGCTTATTTTCCATGTCTGCAGAGCTTCTCAGTATTTATATTTATTACCGTCACGTAGATGTTTTCCCTGCA

>a.58

TGCAGGAACATCTCCAGCTGAGGAGGGCACTCCAGAACCGTATCCGGAACGTTGTGGCTTCATTAAAGTAAGGTGGTGGCAGCATCATGCCTGA

>a.59

CGGCCGGTTTAAACCGCCTGTCAGCTAGCCGCTGTGTTTACACGAAGCAGGGTCAGTGTTTATGAAACCCTGAATGTCCTGGGGGACACCTGCA

>a.60

TGCAGGACCTCAGCTTCCTGCTGTTGATTTATTACAGGAGAGAAAACATGGAGGCGAGGCTGACCGACTCCTGGCTGGAGGTTCCCGTGTCCAG

>a.61

CACTTGGACCGGAGCGCCGGGAAAGTTCATCCTCTTCTTTTACCGTCTGAAGACGGACAGCAACCAACTTACAGGGACTTTACTGCCACCTGCA

>a.62

TGCAGGTGAAGGACATTATGTCCGGTTCCCCGTTTCACTTCTCAGAAATTCAATGAAACCCAGTCACATCATTTTTTGTTTTACAACTTTAATT

>a.63

TGCAGGCCTCAGTCTGTCTGCATGGGTGTGTGTGCGTGTGTGTGTGTGTGTGTGTGACATAGGAGAAATAGAAGTGCTGTGGAACATCTCTCAG

>a.64

TGCAGGATTTGTTGGCTGTTCCTGATGCCTGCCTCGGCCCACAGCGCCGCCACGCGCTCTGGGCTGGTGCCGCCCGACCCGTCGTCCAGCCGGG

>a.65

AGTTTTCTGTTTTTTGGCTTCCTTCATCTGCTGACCATGTGGTTCTGGTTCTGAATGGTTCTGGATAGTTTTAAAGGTTCTATACGGTCCTGCA

>a.66

TGCAGGACGTTCACTGCTGAGCGGTCAGGGACATTAGCAGCACATGTCTTAGGGATTAACTCAGGCTACATATAGATGAAGCAGAGTTGTTCCC

>a.67

GAGACTCATGCAGGAACACAAGTAAGCTGCAGCTGCGTCTCATCCTGGCTGCTGGCGGAGCTAGCGGCGCTAACATGGATCTGCTTCCCCTGCA

>a.68

TGCAGGCTGAAGGCGGCGCGGCTGGACAACATCTTCCTGACCCGACTGAGCTGGGAGAATGTGGGAGGCCTGTCAGGTCAGAGGTCAACTCTCA

>a.69

TGCAGGCCTAACATGGGACTTATTTCATTATTTATGTGTGGACAGACTAGAAATGACGTCCTGAACTGAGATTAAGGAGCTTTAAATCTGTTGG

>a.70

TGCAGGGACAAACCGGGTTAACTGGGCCCTGGGTTCTGACTGGCAGGCTGCAGACAAGAAACAAACCTCTCTGCTGCCTCCTTGTGTGAGAAAC

>a.71

TGCAGGGGGCAGTGCTTCCAGCGCTGCCTCAGCCTGGCGTGGTTTCAGGTCCGAGTCATCAATGTCACAAAGATCCACCTGTTTTATTTTATTC

>a.72

TGCAGGAAGCAGAAACAATATCAGAACATTTCTGGAGTTATTTAAACGGGGCCTGCCAAAGTTTGCCCATCAGATGGAACCCAACGTTCTGATG

>a.73

CGCGCCGCTACGATGACATCCCTGCACTTCTTGCTGGCAAAGTGACAGTTGACATCTCTGGCGTATTTGAGCAGATCTGTGGAATCTACCTGCA

>a.74

TTAAAGGCCAGGCTGTGTGATTTTAAGGGTAACCCGTGCTTCCCTGAGGCTTTGCTCACATTAATGAGTGAAATCATCACAAAGTCATCCTGCA

>a.75

GCAAGATGATCGACAAGACAGGCAAATGCAGAAGCATTTCCAGGATGTAATACTCCACAAAATGAGCGTCTCTCCCCTGAGCAGAAACCCTGCA

>a.76

CAGGATGTCGGCCCTCGAGGACCCACTTTGGACCCCCCTGCTTTAGGCCATCCTTCGGCTTTGCTGCGCGTTAACCTTTGACCTTTGACCTGCA

>a.77

CTTAAGATTTAGCAAAAAAAAAAAGAGCTACATTGATGAGTTTATTTTGCTTGGCTGGACAAACTGTTAAATGTTGTTCCTTTCCTGTCCTGCA

>a.78

CAAGAAATTATTTTTGTTAAATCCAATATGGCCGACAGACAAAAAAGCATGCCAACAGACCCTGTTCCTCCTTCTACTGTAGACAACTCCTGCA

>a.79

GGACAACGTGGAGTCAAAAGTCCATATTTTAGCTTTTTATCTCTCTCCTGCAGCTGCACTGATGATGAATGATCAACACATTTATGGTCCTGCA

>a.80

TGCAGGGGTCTGTGCTTAGATTAATGTTCAACACACAAGGTTCTTGGGTTTCAAGTAAAATAAACCTTCTCGGTTTTACAATGACATATCCCAC

>a.81

GGTCTGTTTCCTGGTTTCAGGGAGCAGAAACCGGCAGGCGGGGATGGATGGAGAGTTTCTCCCGTAACTCTGGGCAGAGTAAAATAAACCTGCA

>a.82

TGCAGGGCACCATTAGATAAAAATAGAAAAGTAGATAAAATGTATAATTGAGAAGTGTATTTTATGCTCAAACTTAGTTTTTTATTGTCTTTAA

>a.83

TGCAGGGGGATAAGACAGGTTAAACAAACCAATCTTTACACCAGAAACCCGTTTGGACACTGACACTACAAATAGCTACAGATGTGTGTTTACC

>a.84

TGCAGGTTAAATTCCCTGGACTTTCTCTCGGTGCTAATTGGCTTCATGAACGAGGGCAGACGTGTCGACAGCCAGAAGGAAAAGCTCCTGCATC

>a.85

TGCAGGCCTTCGCAGGAGAGCCAGGGGTCAGGGGTCAGCACCACAGGGTGTGTGTGTGTGTGTGTGTGTGTGTGTGTGTGTGTGTGTGTGTGTG

>a.86

TGCAGGACATGTTGGAGTTTCTTTTCTTGTGACCTTTAGCTTAGCGGATTTTTAATTTCTCCATGAAGGTTTTTTTTATTATTATTATTATTTT

>a.87

AATCAGAACCGTTCTGATTCACATGGACCTTCCTGTTACATTTGTCTCTCCACTCCACCCTAACGTCTGCAGGGAGACAGGCTGTGGACCTGCA

>a.88

TCATCAGGATTGTTCACATTGTACCCGAGAGTTTCCAGGTTGCAGTTTGGACTCTTCAGTCCAGCAGAGAGAAGCTTCACTCCTGAATCCTGCA

>a.89

TCACCAGGATTGGTCACATTGTACTTGAGAGTTTCCAGGTTGCAGTTCAGACTCTTCAGTCCAGCAGAGAGAAGCTTCACTCCTGAATCCTGCA

>a.90

GTGACGTCACAGTGGGAGGATGGATGGATGGGGGTTGTATGTTTGTCAGATGGCTAGTGATGATGTCACAGCTCACCTGTCCTGGTTTCCTGCA

>a.91

CTGATTTACGCTTATGGATGTGGCCTCAGTAAACTGGTTCCACTGGAGAGCAGAGCTACCTCCCATCCTGCTGCTAGAACCTCCAGAACCTGCA

>a.92

GTTTTTTTTGTGCATTTTTTCATCTTGTTCTTTTCTATTTACCTTTTTTTTTTACATTTGTGAAAATATTTAGCTCCACTTCCTTCTCCCTGCA

>a.93

TGCAGGACACCAGCTGGTCTCCCCATCTCCAGGACAGCTTAACAGACTCCACCCCTGGTTCCACCTCCACCCTGTGGACTGAGAGACAACAGAG

>a.94

CTTCAGCCAACTGGCTCCTCAAAACGTATACTTCCTCTTAATCCATGTTCTTTTCCTCAGGGCGTATTGTTTTCTGGTGACCTGTTGACCTGCA

>a.95

TGCAGGCTGACCGCAGGCTCTGCCTGGTATCTTTAAGGGACTCAGTCTGACAAAGCAGGCGGCCATGTTTCTTATCTCCGAGGCATCGCCAGCC

>a.96

GCTGCTGTCTGTCCAACTCATCTTGTCCTCTGTTGTCTCTCAGACCCAAAGGTGGAGGTGGAATCAGGGAAGGAGTCTGTTAAGCTGCCCTGCA

>a.97

CCTCTGACGGCTTCTTGCTTTTTGACCCAGTCGGGCCTCCGTACGTTTTCTACTTTCGAGTGAAGTCGTATGTAGAAGATCCAAGGAACCTGCA

>a.98

TGCAGGCTGCATGGTGGTACAGTTGGTAGCAATGTTGCCCTGTAGGAGGAAAGTTTTTACAAACAGGATGCAACCCATTGTTTCCTTTAAATTT

>a.99

GCTCATTAAAAGATTCACACAGGAAGCAGGAGGGAGATTGATAGCAGAAAGTTTAGTGGAAGGATGACTGAGCTGAAACCAGATTCAACCTGCA

>a.100

TGCAGGCTGATCATTAATATCATCATATTAATGATGATGAGGACCCAAACGTTCCTCATCATCATTAATATAGAGATGATTTCTGTTTTCCAGA

>a.101

GTTACATCATCATCATCATCCCGTTACATCATCATCATCATCCCGTTACATCATCATCAGAAACGGAGGCTGTGAGGCTGTCTGTGTCCCTGCA

>a.102

TGCAGGTACTGGAACCAGCTGGTTCTGACCCGGTTCATGGCTGGTACCAGAACCAGCAGGGGCCCTACTGAGCATGCTCAGAGGGAATCTGACC

>a.103

TGGTTGTCAGGGAAACCTTCCTCTTCCTCCTCCTCCTCTAGATGAAGATGACGTGTAGTTATTCATGTTCAGGTAAAGTGTATAGTTTCCTGCA

>a.104

ATCTCAGGACAACGAGCAGAACCGTCCTTCTGGACTGCCTGCTCCAAAAACACTTCTATCGCTTAAGATAAGATATGTGGTCAAATTTCCTGCA

>a.105

TGCAGGAATCATCCTAAGATTCTGTTTACGGCGACGAAAAGTGTGTTACTGCTCTGCACTCTGCTAAGGAGCATGCTGCCATCTTGTAAACAAG

>a.106

ACCACATGCTATTTATTCAGCAATGTGTTTAATTCGTTCCACCTGCATGGGGGCCACGAAACGCCACCGACAGAATGGAGACCTCCCACCTGCA

>a.107

TGCAGGACAGACATTTCAACACATTTTAAACCACTGAAAGATTGTTTTTAATGAGACAGAAATATACATTTAAAGAATGTGCTTATGTGCAAGA

>a.108

TGCAGGCAAATTTAAAAATGTAGATAGAAGGGCTGCTGCATTTCAAACAAAAATACATTTCTTACAATTTTGCATGGCTGAGTGTCATGATGTA

>a.109

ACATCGAGGTAGTGTGCTTGGAAACAAAGGCACAACAATAAAGCCACTAGCCAGGCTCCACTAGTTTAGACTTGTCTCTTGAATAACACCTGCA

>a.110

TGCAGGCACCTGTACCACTCTGAGCAGAGCTGCTACAGCCAGAGGGGCCACGGTAATTTGTGCTAATCTCTTTTGTGCTGTTTATTAAAAAAGG

>a.111

TGCAGGCAACACAACCAGGGCAGCAACCTGGAGACCGCAGAGCAGCGTTGCTTCACAACTAGAAGCCAAATAACTGCTGGGCCTCCAGTTGTTT

>a.112

TGCAGGATGGACTGATTTCCTCACTACTCTACATGAACTAAATATTCCAGTATCTTGTGTGAATAACCCACAAGCCCGTGAGCAGTATCTGCAT

>a.113

TGGGTAGCGCAGTTAGCCTTTTTTTGATTTTATTGTGAAATATCAGGCAGGAAAGGAAAACGTTAATGCTGATGCCTTGTCCAGATTTCCTGCA

>a.114

TGCAGGCTGCAGCTGCTGACCGACTTTTGCCAGCAAAGCAGTTTGCATGTTTCATTTACAGACAAACTCACCTTTTAACTCGGTTAGAGTCCAA

>a.115

GCTTGTCATTTTTTCGCTTTGTCACAAATCCCAGCTGATTTCGAAGTGGTAAAACACCATTAGTGCTTGCCTTCTCTGAACTTACAAACCTGCA

>a.116

TGCAGGAAGGTGTCAAAGCTTAACAGAACGCCAGACCACAAGAACATTGGTTTAAGTTTCTGTGAAAACTATTACCGATTACTTTCATAGTTTC

>a.117

CTTCCTTCACTGAGTCCTCTGCTCCTCTGCTGACGCCAAACCGTCAGGAAGCTCCGCCTTCTCCTCCTCCGCCGCCGCCGCCGATGATCCTGCA

>a.118

TCACCAGGATTGGTGATATTGTACCTGAGAGTTTCAAGGTTGCAGTTTGGACTCTTCAGTCCAGCAGCAAGAAGCTTCACTCCTGAATCCTGCA

>a.119

TGCAGGGGTCAAAGGATGACTCCACACTGCAAAGTCTGGAAGGACAACCTAGTCTGTGACAACTTGCTTCTGGAGAGGTGTTTCAACATCATGC

>a.120

CGCGCGAGCAGGCCTGTCCTTCCCACCAGTGAATACCTCGTATTCCCCAGTAATTTTCCGAGTGCTGCGGTCTCAGACAGGCCCCAATCCTGCA

>a.121

TGGATGGGTGGGGCAGCAACGCAGCAAGACCCAAGGAGAAGTCGACCCTGTTCCGAGTAACAGCAGAGTACAACTTCCGTGACGGGATCCTGCA

>a.122

TGCAGGAGACACGGATGCTGTGTCTCCACAGTCAAGACTGGAACTGAGATTCCGGAGACCTCTTCCCACCACAGTCACATTGATAATCTATTCT

>a.123

TGCAGGTTGCCTTTTTTTGGCAGCAGTGTGATGACTGCCCTGCGGCAGGACAAAGGGAGCGAACCGGTGGACAGGCTTTCGTTGTAAACCTCGA

>a.124

AGAGATAACAACGCAGGAACACAGCGGAAAACACGCGCGACAATCTGACAAGATGACACCAAAAAAGCAGACACCGAAAAACACGTCCCCTGCA

>a.125

TGCAGGTTTCGAACGGTGAGTGTGTCTCCTTTTAAGGGAGTCATCGTCGTCGTTGTGACAGGAATTGTCTGTGTTGTGACGATGTTTCTCATTA

>a.126

TAAATCAAATGTCACCAAGGGATCCCATTTGACTGATTTACTCAAAAACTTTCTGGTTCCATTTAAAAGAAAAGCTCAAAGCCTCAAGCCTGCA

>a.127

TGCAGGATGGACTGATTTCCTCACTACTCTACATGAACTAAATATTCCAGTATCTTGTGTGAATAACCCGCAAGCCCGTGAACAGTATTTGCGT

>a.128

TGCAGGAGCTCTTACCAGGATCTGAGAGGAAGCCCAGAGGGGGCAGGGAATTCTTTACTTCTGCTCATCAATTGTGGGATAATCGTTTTGCTTT

>a.129

TTTGGCCCTCACTCCCAGGAGGCACAGGGTGTTCTGAGTCCTCAACAGCCTGCTCGGCCTGGGCTGAGGATTTACTCTTGTATCCTCACCTGCA

>a.130

TCTTTTTATACTGACTTGTACCAAACAAAAAACAGCTTAATTGCAAAAAATAAATAAATAAAATAAAATAAAATAAATAAATAGCTTACCTGCA

>a.131

GAGACCAGGTGATGATGATGCAGAGCGGCGAGGCGGTGAGGCGTGTTGCCAGGTGACACTGAGGTCTGGAGGTTTGTTTTTTCTTCCGCCTGCA

>a.132

ACTAACATGTTCCAACAAACTGGGATCATCTGGAGTAAAACCAGCAGGGAGACGAGAAGCAACAACAACAACATGTGACCAACTTCAGCCTGCA

>a.133

CCTAACCTGTTACAGGAAACAGAGTCGGTCGCCTTTGAGGTGCAGAGGCAGAAAGACGTCGAGAAAATGTCGGTTTGTGTTTGTACTTCCTGCA

>a.134

TGCAGGCCTGGAGCTGCCCACCCCTGATCTAGAGGATGAAAGTCTGGTTGCACCTGTTGATCCTCCAGTTTCCTCCTCCTGATCTGACCAGAAA

>a.135

TGCAGGGGAGCCAGAAAGGTGAGGCTACTGCAGACGGTGACGACTCAGTGGTTCCTCCATGCACCTGACAGCACCTGCATGACGGTCACGGCTC

>a.136

AGAAAACCAGTTTATATTAAATCCCTCCAACACTCACACCCCTCCTGAAGAAATGCTGCTTTCTGACTGCGGCCCGGCAGCGGCGCCCCCTGCA

>a.137

TGCAGGGCCTCCTGTAACTTTAGATGCGTCTCTACTTCAACACACCCGAGTCAAATAATGAGGTCGTTAGCAGGATTCTGGAGAACCTGGAGGA

>a.138

TGCAGGACAGGAAGAGAACCGGCAGCTTTAGACCCGGCGAGGCGGCGGGCGGGCGGGCGCCCGAAGCCACGGCGACACCTACGTTCATGAACTT

>a.139

TCCTTCCCGACAGCGGCGCGGCCTGCGGGAGGAGGAGGAGGTAAGCGGGAAGGTAACAGCTGGTGGTTCCGCCGCTGGTTCCGCGGGTCCTGCA

>a.140

TGCAGGAGTTATTACTATGCTTTGAGCCATAAAAACAAAACTAAACTATTCCATTGAGCAGAAATCTGTCATAGCTCTCTTATTTCTTACACAA

>a.141

CAACCTCTGCTGCTCCACCTCAATACCGACCTACACCAGGAGGAGATGACGTTTCATTCAGGTGTTTTCACCTGTGGCTCATCTAATACCTGCA

>a.142

GAACTTGGCCCGTTATATCGATGAAATGTAAAGATGGTTTGGCATGGAGACAGGAAGCGTCATTAGTTCCCAGTTTTCCCTCTGTGTCCCTGCA

>a.143

TGCAGGGCGGCGACAAAGAGAGACACGCTGAACGGCTGCAGGAAATAAATCTGTTTAATGCAGAGCTTCCATAAGATCCATGCCGCCTGAGGCT

>a.144

TGCAGGTCTGAGCGCTGGCATCAGGTGAAGAAGGCAGTGGACTGGGTGCGCTGGGCCGACCGGCAGTATCCGTACGCCTGCAGCCGGCTGGAGC

>a.145

GTTCTAGCTGATCCGGACAGCCAAAGATCCGGTTCTGGTTCTGGTTCCCGGTCCAAGTTTTGGTTCTGGTTCTGCAGATGGTCCAGCCCCTGCA

>a.146

TGCAGGGCTGGTGTCCTGTAACTCTTAATGTCTCCCTGGTCCAACACACTTGAATCCAGCAGCTGAATCACCTCCTCAGTGCAGGCAGGTTCTC

>a.147

TGCAGGACGCTTTACAACTCGACTCACGTCTCCAGGTTACCTCCAAAACATTCACTTAGAAACACAAGATTTGAGATTAGTATGAACTTAAAAA

>a.148

TGCAGGAACAACCAGGTGAGGCTGAGCTGCTTCTCATCCAGGTGAGCTGCTCAGGTGAGGCTGTACCTGCAGATGATGCTCTTCATCTGGCCGT

>a.149

TGCAGGTTAGAGAAGTTGTTCATGTTAAAATTTCAAAAACCACAACTCTATATCAAACAAACAAACAAACAAACAAACAAAAAAAAAACAGAAA

>a.150

TCATTGGTTCACATTTAGTTTCCTTTAACATTGTGGCCAACTAGTAGGACTCAGACGATGAATGTTTCAGACCAACTTAGAGAAGAACCCTGCA

>a.151

CGTTGCTACTAAAGCTCAAGCGTGTGTGTAGTTTAATTACAAATGCTGTCAAACACCCCTATTGGTTCAACATTTGGTATTGTGGCGGCCTGCA

>a.152

TGCAGGTGGTGAAGTACTGGCCAGAGATCGGAAAGTGTGGCTATTTGGTGTGGCGGTATCTGCTGAGACGAGACGACCTGGAGCCGGCTCCGTG

>a.153

GACTGCAGGAACAGCAAACCGGAAACAACCAGCAGATTCCTGCTGATCCCAGCGTTCCCAGTGTTCCCAGAGGAACCAGCTGCTTTAACCTGCA

>a.154

TGCAGGAAGTGACATCATGAGGAGGGTCGGACAACGCTGGTCTGGAACAAAGAGGAGGAAAGGTCAAAGGTCACCTGATGCAGGCTGCAGGCTA

>a.155

TTGGCCCACATGGACGCCTCCAGCTGTCCGCCTCATTAATGATCCACAGATAATCAAACCAAGCAGAGCTCAGAACCAGAACAGAGACCCTGCA

>a.156

TGCAGGTTTAAATAAAGCAACTGGACCTTCTGGTCACTGAAAAGAGCCGAATCTCAACTGTATGAACTCATATTTAACTCAGACGTTCATAATG

>a.157

TGCAGGCTGAGCAGGCCGACGGAGTGGTCGCTGGCCACGGAGCAAACGCAGTGCTGCACGCGGGTCTGCAGGGAGGAAACGGCAGGGACTCAAA

>a.158

TGCAGGATTCAGGAGTGAAGCTTCTCTCTGCTGGACTGAAGAGTCCAAACTGCAACCTGGAAACTCTCAGGTAAAATGTGATCACTCCTGGTGG

>a.159

CTTCGTATTGCTGCTTGTAGGAAGTCACTGAATTAATCTTGAGTTCATGTTGGACCCAGATTCTGTTTCTGCTGAAGCTGAAGCTCCACCTGCA

>a.160

TGCAGGAGAGAGCTGCGCTACAGTGACAACATGCCATCGTTTCCTTCTCTAATACAGACACGTCAGCGTTACAGTGCCTCCAGGATTTCATTAA

>a.161

TGCAGGCCCCCGCAGGCCCCCACAGGCCCCCGCAGCTCAGTCAGCCGGTTCAGCTCCGACTCACGCAGCTGCTGCTCAGAAAACATATAAAACC

>a.162

TGCAGGAACGCAGCACAAATATTATTCACACAGGACAGAGACGGAGCAACGACAATACAGGGATTACACTCACAACGACTTTTGGAAGATCTTT

>a.163

ATTTATAAATTAATTTCTTCCCAGCAAGGGATTGTGCAGCAGAGACTATAAAACAACTTTTAAAAACTCCTGGATTTGGGATTTCAGGCCTGCA

>a.164

ACTTCCATTTGTTATTGTGTCTGTGTTTGTGATTGCTCTTTTCATTTTGATTATTATTATTATTATTATTGGTTGTTTTCTCAGGAAACCTGCA

>a.165

CAGCCTGGTGGGGGCAGCAGTCGTGGCCGCCGCCCCCCCACCGCTGAGCCCGACAGCCAATGGGATGGCAGCTGCAGCAGACTCCGCCCCTGCA

>a.166

TGCAGGTTACCGACTTTGGACCAATCTGAGAAACGTCCGAACAAACAGCAGTCCTACAGGAACTGGACCATTTGGAGTTTCTTAAGGGGTCAAA

>a.167

TGCAGGAGGAGGAAGATGAAGGAGGAAGAGGAGCAGTAGAAGAAGAAGTGAACATACAGGCTCTAATGTTTACACACTCCACTTCCTGTCTTCA

>a.168

TGCAGGACGGAGGAAAGAAAACAAACAGCTTGTGGTTTTTGTTTTGTGAGCTCCTTCAGCCTCCCTCACATCTCGACTTGTTGTTTTACTAATT

>a.169

TGCAGGAACAGAACAGCTGCTCCAGCATTTCTTCCTCCAGCCTTCCCAAAATAAATGGCAGGGAAAGGTCACAGTTCTGTCCGCTCTGACCAAC

>a.170

TGCAGGATGAGTCTGTTAAAGATGAAAGTTACATGAGGAGTCCAGGCTGGATGTAATGAATCACTGATGAGGATTTTAAATTAATTTATGACTC

>a.171

TGCAGGATGGAGGAGCAGCCCTGATCTGAGTATTACCTGGTTACTATCTGGTTACTATCTGGTTACCATCTGGTTATTATCTGGTTGTTATCTG

>a.172

TGCAGGTGTGAGGTTCTGCTCTCAGGTCAAATCCTCCAATTTCATTAGGAAATGAGCGACTTCATTCCCGACTGAGCGGCGCCAGCGCTTCAGA

>a.173

TGCAGGCCAGCGAGACGAGAGCAGAGATGAGACCCAGACATGACCAGCCGATGACGGAGCGCCTGCTGCCCTCCTACCCCTCCATCTTGGCCCA

>a.174

TAATGGCCTAAAACAGGAGAAGTTTGGTCTGGTTTAGCTTCATTAATAATTAATAATGTCACTAGAACTTTGATAACTTTTAATTTGACCTGCA

>a.175

AAGGAGGAATTTATCTTCTTCAGAGTGTGTGTGTGTGTGTGTGTGTGTGTGGGGGGCAGGCAGCAGGTCGCACACAGATGAAACCAGACCTGCA

>a.176

TCTGGATGCTTTTGGTACCAACAACACACTCTTCATCATCATCCTCCTCCTCTTCCTCCTGACATCTGCTCTCTGTCTCCAGTCCAGGCCTGCA

>a.177

TGAAGTTTTGACACATCCAGCTTAAATCCTTTATTGTTGTTGGAGCTTTTCATGGTTTCTGAATAATTCTATAGGCATCATTAATAATCCTGCA

>a.178

TGCAGGACTGCAAAGCAAACACAATGTCCTGATGCCTCTGGCCGGCCGGCGTTACCTGCTGAGGGACCTGAGACAGATTCTGGAGGACAGAGAC

>a.179

ACACACACACACAGAGACACACACACAGAGACACACACACACACACACACAGAGACACACACACGCAGCCTGAAAAGCACCTGATCGTCCTGCA

>a.180

TGCAGGCAGCGACTCCTCCATCTCTGTCAGCCGCTCCGTCACCGCCGAGGCTGACACACATGAAAGCCGGATGCAGCTGCGGAAGCTCCGGATG

>a.181

TGCAGGGACACGCAGGTGCAGTGAGGGGCGGGCCCCACCTGAAGCTCCTCCTCCCGGCTGCAGCTTACCTGCATGCACCTGAGCAGGTCCAGGA

>a.182

ATCTCTGCTAACAGCTAGTGGATAGAAATCACCAGGATTGGTCACATTTTACCTGAGAGTCTCCAGGTTGCAGTTTGGACTCTTCAGTCCTGCA

>a.183

TGCAGGCAGGACTGTGAGCTGCTGTGAATCTGACCTCATGACCCAGAGCAAAAGGCCCCAGAGCCTGAGCCTCGTCCGAGCTCTCTGATTGGCT

>a.184

TGCAGGAGGGCGAGAACACCGTCACCTCCAACAGCTCCATCATCCACCGCCCCACGGTGAGCCCAGGGTCGGGACCGGACCGGACCGGACCAGA

>a.185

AGAGTCATGCTTTCATGCTGTATCCCCCCTTCATATATCATGACTTGTCTTTCTCACACTGGACGCATTTTGACATGTCGCTCCTCTCCCTGCA

>a.186

TGCAGGACTTTTATTTTGAAGTTCACGTTTGGATCAGATCGCTGGAGTCACGTCTCTAAAGATTTAACTCCCCCGATTCTGCCGTCATGTTTCC

>a.187

TTTGGTCTCAATAAGGTTCCAGAACGGAGAGGAGAGTTTTTCCATCTTTAGAGAATAATCCCAGGATGATGTTTGGCTAATTTCAGATCCTGCA

>a.188

CTCAAACATGATGAAGGCTCACCGAGCCGCTGCTGGCGGCCGCCCAGCAGGAGACCGAGGCCAGCAGAACCGCTTCACCTCCGCACGGCCTGCA

>a.189

TGCAGGAGCGCGGCTAGAGTCTTGAGCCGACCCGACAGTGAGAGCCCGTTGGCTGGATGATTGTGCTTGCACAGTTGACAGATGGTGGGTGATG

>a.190

CCCTGCAGCAACCTGGGAACCTCCTGGTGGACGCTGGCGGTGAGGGATACCGTCAGGCTGAAGAAGGAACCTTATCAGGCCTTTTTGGCCTGCA

>a.191

TGCAGGACCTCAGAAGCAGCTGATGGGTACCAGCAGTCCTTGTGGTATCCAGCTTGGGTGGTTGCTGAGGCAGAAATTCTGGCATGGGATGTTG

>a.192

TGCAGGATGAATGAGTGAATGACAGGTGAATGAGTGAATGCTGTTAGCTGTGTGGTAAATTATCAGGATATCGTTTAAAGTTTCTAAGTGCTGC

>a.193

TGCAGGAACACACACAGAGGGCATGGGGTTAGGAAAAGAGGAGCACTGGTCATCATAATATACACACACACACACACACTCTCATGCCATCTTT

>a.194

AAAGAAGAAAATTCACCTTTAACCTCAGGAATCAATAAAACATTTAGTTTTTGTTTATATTTTCTGATTTTGCTCCAAACTACGGTGGCCTGCA

>a.195

TGCAGGTGCGACACGCTGAACAAACAGAACATGATGCATCAGAAACAGAAAAACTTAAAAAACGTTCGTCCTCCGCGCCACCAGGGGGCGTCGA

>a.196

TGCAGGGACACACCCAGCGGGATTCACCCTGGATTTATTCATGATAACAGGATTTCTGCTGTTTGGAGCTTATTGATTCCTGGATTATCGACAA

>a.197

AATGTCGGCGCGTTCTGCAGCATTCCTGCCTTCAGCAGAAACCTGCAGACTGATGTTCAAATGGGAAACACTTTATGTTTAGAAACTACCTGCA

>a.198

CATTATTCAGTTTTATCCTTCAGTCTGAGTCTCTGCTGCTTCTGAACATGGAAACATTCAGACGTTTGTTTATGCTGACTTTAAAACTCCTGCA

>a.199

AAATACAGCAAACATGTCTTTCCTGCTACACCAGCAGGCTGCAACGCATTCCCCGTTAACGTGCCTCACCTGCGGCCGTGCAGGCGGCCCTGCA

>a.200

AACGGCAGCCCGTAATAAGTTATTCAGGACATTTAACTACCTAGTGCTTTGGCTGCTCCCAAACAGAGTGGCGCCCTGGGCAGTGAGCCCTGCA

>a.201

TTTTAACGAGGTGAAAACTTTTTCAAAAATGTTGATTTTTACGTGCTACTGGCCCTTTAAGCACCACTTTCAGTTGACCTGTAGAAAACCTGCA

>a.202

TGCAGGAAGGCTAGCAGAACCAGAACCAGAACCAGAACCAGAACCAGAACCGCCGGTCTTACTTGAGGCTCTCCTGCGAGGATGAGGAGGAGCG

>a.203

AACACATGGTTAAACAGAAACACAGGAGTCAGTGGATGATCTCTAGGAGAACATGGAGTTATTTGCAGTAGTAGTTCAAATGGCGCCCCCTGCA

>a.204

TGAAGGCTGACGTCTCGGGTTCAGCTGCAGGTGTTTGTCAGCCAGAGCCTGAAAACAAACTTTAGCTTCTGTTTGTTCTGCAGTTTTACCTGCA

>a.205

TGCAGGTAGAGCCAGGCTGGTTCAAACCACGCAGGCTCCCAGCGCAGCTACAACATGACCGCAGCAGAAACTTCCTCCAATATCTTCACTGATC

>a.206

TGCAGGCGGAGCAGGGCGCTGCAGACAGCCTGCGAGACCTGGAGAGGAAGGTGGAGCAGCAGGAGCAGAGGCTGGTCCGGGCGGAGGAGCGGAG

>a.207

GATCAATCTCAAAGTCCCGCTGGAGGTAAAGCTTCCCATGCAGCTGTCACGCCTCTAGTGGACGTCCTCTCAGCGTGTCCTTCGTCCTCCTGCA

>a.208

TGCAGGTCACACACACCTGGAGGTGATTTCTGTAAAAGTGCCCGTCTCTCTCAGCAGAGCGGTGGCGCCGCCCTGCTGGCCGTCTCTCTGCAGC

>a.209

GTTCGTTTTTGTTGTTGTTTTTTTTTTTGGTGGCTCTGATGGTGGATTAACATCGAAACTAAACTGGCAACATCCACTGCAAGCAATCCCTGCA

>a.210

GGCCAATGGGGCGGTTCCGGTGGGCGGGGCCAACAGGGGCGGTTCCGGTGGGCGGGGCCAATGCCAGGCTGTGCTGCGCAGCAGCAGGCCTGCA

>a.211

TTCTGAAATTAATCTAAAAATGTGATAGTTTACTGGTGGACATTTACTCCTTTTATTTTCTATCTACGATGGTCCTCATTTCATTTTACCTGCA

>a.212

CAGAATTCCTCCGTTTTCTTTCTTCTGTTTTTATTTGATTTGAATCAAATCATTTCAGTGGAATCTGAAGCTGCTAGGACTCCCTCATCCTGCA

>a.213

TGCAGGACAGACGGCATGCTCCATGCACGCCTCCTCACACTGGCCCACCACCCGGATCCAGGTCCAGATCCAGGTCCGGAACACTGGACCAGAA

>a.214

TGCAGGGGGCAGCGATGGGTCATTTATGAGTTAAGCACAGAGAGCTACCGGTAGCCCTGCAGCAGCTAGCTGCTGCAGGGCTACTGGCTGTAGG

>a.215

TGCAGGTCTCGGCCTCTTTGCCTCGCCTAACAAAGTTCAATATAAGCTTGCAGGCCCGGCTGCCATGTGCCTAACTTGGCTTTAACAGCCTGCA

>a.216

TGCAGGAGCACAAACAGCCTCAGAGTGGTGGGCGGGGCTATGACATCACCACCAGGTGAGGCGACCGTACCTGTCCACCAGCTGCTGCAGAGGG

>a.217

TGCAGGTGTCCAAGCAGTACCATCAGAAATTACCTATGTGGGAGAGAAAATTGTTGCTCCTTCAGTCCATGAGCAAATAGATGCATTGGATGTG

>a.218

TGCAGGCGGAACCGCAGCGCGGCAGAACCAGCAGGAACCAGGAGGAGTCAGCGAGTTTCAGCACAGGGAGGCATTTTCAAATTTAAAAATGATA

>a.219

TGCAGGATTTAACATTTCCAACTGTCAGGACGAATTAACCGGCACTAATTCCGCTTTTCATCTGCGCGGCGCGGCGTGACGCGGCGCGGCGGCG

>a.220

TGCAGGATCCTTCACCGTGTCCTTATCAAAGAAGACAAATTTTCTCCACTGTAGAAATGCTGCCATTTTCATAAGGTTAGCCAGCTAATGACCG

>a.221

TGCAGGTTCAACCACAGAATTCATTTCTAATAAAGCATCAAACGGTACGCTTTAGAAATCCAGAACATCCAGGAGTTTATTTCACTTTGTTTGT

>a.222

TGCAGGATAAACACAGAGATCCAAATGTTTCCCCTCAGACTGACAGGAAGAAACATATGGAAACACTGATGAGTAGAAACTCATGCAGAGATTT

>a.223

TGTATCATTTCAGTATTTAAAATGATAAACAAGACGGACAATCTGACAAAATGGGCTTTTCTTTCTTTTTTTTTTTTTTTTGCGCCAACCTGCA

>a.224

CTGCAAATAATAATACAGGTTCTGTCCTTGGATCATTGATCCGATCGGTACTGATGGGGTCTGATCAGTTCTCCTGATGGGTTCTGCCCCTGCA

>a.225

TGCAGGAGGCAGCAGGGGGCGCCATGAGGCTGGCAGACATCACTGGATCCCGAGCCTATGAGGTTCACCTGTCTGTCCACCTGTCTGTCCACCT

>a.226

TGCAGGATTTTGGGTTTTCTGCACAAAATGGTCAGAAAATCCCACCGAAACATTTTGAAGTGAAGCTTCAGACATCAGCAGCTTGAAGCTCACA

>a.227

GTTTGTCTTTCTGCCTCGCCCCCCCCAACCCAAGTCTCTCTTCATTAATGGTTACATGTGTCACACCCGCTCTCACTTCAGTCTTTTCCCTGCA

>a.228

TAAATAAAAAGATTCCAGTTCCAATAACAGCAGATAATACAGACTGGAGAGTAACAGGAGAAGAAAGTGAGAGACGTGGCCCAATTGTCCTGCA

>a.229

TGCAGGTGCCTGTAGCATCATGACTACAGACACAGCTCAGGATGACCCCATGCTCAGCCCAGGCTGCGGGCGAAAGCGGCTCTCTGCGGCGCTG

>a.230

TATATTTATAATTGGTTTGCTGAAAACGTTACAGTTTGATGCTGAAGCAAAAATGTTTAGCTGGACCTCATTGCTGCTGGTCTGTGTCCCTGCA

>a.231

TGCAGGTGAGAAATAGTTTTACAAAATGATAATAAATATAAATGTAAAAAAAAAAAAAAAATTAGACAATGAAGTTTAGCTTTGTTGAGAATCC

>a.232

CAATTTCCCCACTGCTCTGTGATGTTCCATACAAGCGTAAACACAAAACATTCCTCCAGTTGGCTCGCTGTGCCTGCCTGCCTGCCTGCCTGCA

>a.233

TGCAGGATGCAGCATTCAGGTTTTTTTTTTTTTTTTCCAGTATTCATATTTTTGTTGTGTCATATCACTGATGAGTTTTCCACATAGCCTCAGT

>a.234

TGCAGGTTCTTCAGGACATGAGTATTCTGGTCCAGACATCAGAACCTGCAAAGACTGCAGCACATGAATCTGGACCAGGTGTGTTAGGAGAGGT

>a.235

TGTACTCTGAGCTCCACGCCAGGCAGTAACCGTGATTTTAAATATTCAACTGGCTCTGGTCAGGATTTTCCCAGAATTAAAAGGTTTTCCTGCA

>a.236

TGCAGGACAGCAGAGGACTGGAGTTTAACACCTGTGCTGCACAGCAATCCAAGCATGAGAGGAAAAACTAGAGGATTCGTCTAGAACTGATGAC

>a.237

TGCAGGACGGCTCACACAAAGTATGGGTTTTACCAACTAAACACTTTTTTACCTCTTATATTCTACTGTAAGAAAATGTGGTAATTTGACTCCA

>a.238

TGCAGGACCAGGAACCTCCTGAAACCCTACAGGACCAGGAACCTCCTGAAACCCTGCTGGACCAGGAACCTCCTGAAACCCTGCTGGACCAGGA

>a.239

TGCAGGTCTCGGCTTCTTTGCCTTGCCTAACAAAGTTCGACATGAGCTTGCAGGCTGGCTGCCGTGAACCTAACAAGGTCCAAACAAGCCTGCA

>a.240

TGCAGGGAGGGGAGGGGGGGAGGGGGTCTGCTGTAAAACATGAACGGATTCACTGTGAGCCGTGACCGCTTAGATTCTCAAGAACAATCTAAAA

>a.241

TGCAGGGGGCGCTACAGAGCATCAGCTCACCTGTCCCACCAGCAGGTGAGTGCTGCTGAGCGGTCCGTGTCCCGTCTCCTCCTGATCCTCCTGG

>a.242

TGCAGGCAACAGCACCGCCGGCTTTAACACCAAACTGCTTCTGTGGCTCCACGTGGCGCCGACCCGCAGGCCGGGTTCTGCTACCTGGTGGCTG

>a.243

CGATTTTTTTGCCCCTATTTTCCCCTTATGACAATCTTATAATGTAGGCCAATCTAAGATTGTCGCTGGTGATTTTGTAACCAATCATCCTGCA

>a.244

TGCAGGTTGGAAAATGCGTGGTTTTAATTCCATGTCACAATTATTGACCACTTTGTGTTTCTCTAGCACATATAATTCCAGTACATTGAAGCTC

>a.245

AGAACTTTGTTTTTAATTATGAGAATAAAGTCATTATTTACCAGAGTAAATGGTTATTTTAATGGGACAGAAGCGAAGGTGTCGCCGCCCTGCA

>a.246

TCTGGACACTTGGGAAACAAAAAAGACCCAAGCAGCTGAATGTTTCAGAGTAGCCTAGTCAAAGTCTGGACTTAATTCCAATTGAAATCCTGCA

>a.247

TGCAGGATTCAGGAGTGAAGCTTCTCTCTGCTGGCCTAAGGAGTCCAAACTGCAACCTGGAAACCCTCAGGTAAGTCTGGGCCCTTGAGAAATT

>a.248

CTGTAGGGTCTGATGGAGCCATTCTCTCTGAACCATGCAACTGTTGGCCTCGTGATCCTCTCACTCTGCTGTGGTTGATGTGGAGAGGCCTGCA

>a.249

GTAAAAAAAAAAGTAAATTTTTAAGTAATTGTTTTCATAAAAGTTAAAGTAAATCTAATTGGTTAAATGTAAGTAGTTACAACACAACCCTGCA

>a.250

TGCAGGCCACCGCAGTCATTGTCTCGCTTAACAAAGCTTCGAACAGCTTGCAGGCCGGCAGCCGCAGAGCCTAACAAGGCCTGATCAGCCTGCA

>a.251

TTTTCTCTCAGTGACAGCTGCTGTCTCAAAATAATTCACAGAACTGGACGTGATGCAGTAACTTTGTGTGAAACAAGTTCAGGATTTACCTGCA

>a.252

CTGATAATCCCTCTATAAAGTCTGAGGTGTAAAGCTAGCAATGAATGGTTGTCATGACAGCATATAGACATGACACATTAGCTGTTTACCTGCA

>a.253

TGCAGGTGGTATGAAAATTCTGTATATATACTTTTTTTTTTTTTTTACCTTGCATCACTGCCATTCCTAGTGTCTTAAAATAAGTCTCTGTGTC

>a.254

TCGATTAATGACTAAGACATACTTGTTTTTCATCTCTCCATGTAAGAACTGAATAAGAAACAGCCAGAATAAATAAACAGAGGAAATACCTGCA

>a.255

TGCAGGAAAATGTCCTTCAGACAGCAGAGTTTAGCTGAAGGTTGATCAGATTCGCTCTAACACAACACACAAGTTCTCAAGAAGACATTTGTTC

>a.256

ACACACACTCACAGACACACACACAGAGACACACACAGAGACACACACACAGAGACACACACACACAGCCTGAAAAGCACCTGATCGTCCTGCA

>a.257

GGGGAAGGTGGGCGGAGCCAGGGCCGTGACCCCAGGGGTCGCCGTGGGGAAGAGGGCCATGGCATATCGCGCCAAGGTAGGACCACGCCCTGCA

>b.1

CAGGAGACCTTGGAAAACCAGCGTCATCAGCAGAGCCGCTGATGCATCCAGAAAACACACAAAACAAGAAGGGACTCGAAAACCCCGTCCTGCA

>b.2

TGCAGGACGGAGGAAGAACAGGCTGCGGCTACTGGTCTGCTCCCAGTTCCTGCTGGTCCCAGCAGGGAATCTGGAACTGGAGTTAACTGTAACA

>b.3

TGCAGGCGCCGTTACTACAAACTATGTAAACTGTTTTAATAATTTACTGACCTTATTATAAATGTAATGGCGACTGGAATGTAGGTGACTGAAG

>b.4

AAGTCAAATTATTATTCATGTTTCATGTGGAATGATGGAATAAATTGTTGGGAAAAAAAGCTTATCAAACCATTACTAGAAGGTCAGTCCTGCA

>b.5

TAATCTGCCTGGATGTAGTCCAAAGCAATGACTCAAGTTTAAATCACGTATGTGGAGACTGCAGCTTTTCTTTTTTTTCCCCCCTACGCCTGCA

>b.6

CTTTCTCCCCTGCCTGTAAAATGCTGTGCCTAACGCAGTCCGTCAAGCAGAACATGTTCTCTGTGAAGCACGGTGGCGGAAACGTCATCCTGCA

>b.7

TGCAGGACCAGAGAATGCATTTCGCTCTGTCACGTGCATACACCGGCGTTTTCCAAATAGATTTCATGAGGTGCTGCAGAGATGACGTTCATGT

>b.8

ATCTCTGACCGTCTGCAGAGAAAATTAAGATAATTTATTTCCATGGCAACGACCCAGTGAGGCTCAGAAACAGCAGCTGCTGCCTGGACCTGCA

>b.9

TGCAGGTGAAAAGGTTCCTGGAGGTGAAAAGGTTCCTGCTGGCAGGTGAGAACTTTGATCTTCATCAGGAAGAAAAAAACCTGGAAGCAGGAAA

>b.10

ACCGACAGGCAGCTAACAGAATGACTTCCTGTTAGCGTAGAAGCTGCCGGCGGCTAACGGCTACAACTCGAGTGTCTCTGTTTTCGTTCCTGCA

>b.11

GAACTTCAGGAATATCTCTGGTTTCGTGTCCCGACGTCCCTCCGAACCTCTGGGGAGTTCTGCATGTGGAGTTCCGAACCAACCGGACCCTGCA

>b.12

TGCAGGCGTCCCCAGCACATTCACGCCGAAGTCGCCACTGAGATCTCACCCTCCGTCTGAATGCGTGTGTTGATGTGCGTGTGCGTGTGCGTGC

>b.13

TGCAGGGAAAACCCATTCCTCCTCAGAAGCATCAGACTTTGACTAGGCCATCAGCAGAAACGTCACCAGATTTTTTCACCAAGATGTTTTTGGT

>b.14

GCACAAGTTTAATTAAGTGAGCACTCTTCACATTATCAACATCATGAATTGTATGAATTTTAGCAGAAGCAAAAGAAGAAAAAAAAAACCTGCA

>b.15

AGTAAAAAGGACCAACTTTAAATCAGTTTATTCCTGAATGAAATTATTTCTATAGAATGAGTCGGAATGTTTGGATCCATGGCAGATTCCTGCA

>b.16

TGCAGGCCTCAGTCTGTCTGCATGGGTGTGTGTGCGTGTGCGTGTGTGACATAGGAGAAATAGAAGTGCTGTGGAACATCTCTCAGTGAGTCTG

>b.17

TGCAGGGAGAGACGTCCAATCAGAGACGTAGTTCAGTAGTACTGCATTAGGTCTAACATAGTACTACTATAGTAGTACTATAGTAGTACTATAG

>b.18

CGAGCGGTGAGTTCTGGCCCGGTTGGACTTCAGAACCGCCCTTCTCCCTCGGCCGGGTGTGTGTGACTCCGGTGTGTGTGTTTGGGTTCCTGCA

>b.19

ATAAAACCTACTGGCAGATTTTTCTGTAAACATGTGGCTCTTGTAAGTTCCTTTGCTCTCACTCTCCTGTCTATTCCATCCCAAACTACCTGCA

>b.20

TGCAGGTTTAAGTAGTAAGTAAGGTCAAAGGTAGTTCTGGCACAGCTTGGCTTGTAGGTTCTGGTAGAATCACCTTTGACGGCTTAATTAACCT

>b.21

AAAAAACTAAAAGAATTGGCCATATAATTATTGTACTGTCATCTGATAATCTAGCTTTGCTAAATCAGGAGTAAGCAAAAAAAAGAGGCCTGCA

>b.22

TGCAGGACAAACTGAGACACTGCAGCAGGAAGCCAGGAGTCAGGGCTGCCGGGTGGTACCGGCTGGTACCGGTCGGGCTCAGGTCATCTGGACC

>b.23

TGCAGGCTTTTGTATTCCATTTATATTGACAGAACAGCATGCAATCACTGTTTTTTTTTTTACAGAAAACAACTCATAAAGAGATAACTCTAGA

>b.24

TGCAGGGCGAGGACGGGCTGCAGGGGACCAAGGGAGAAGCGGGTCGCAAGGGGAACCGAGGCCGAGCGGTGAGTTCTGGCCCGGTTGGACTTCA

>b.25

TCACCAGGGAGGAGAACATTGTACCTGAGAGTTTCCAGGTTGCAGTTTGGACTCTTCAGTCCAGCAGAGAGAAGCTTCACTCCTGAATCCTGCA

>b.26

TGCAGGTGAGAAATAGTTTTACAAAATGATAATAAATATAAATGTAAAAAAAAAAAAAAAAATTAGACAATGAAGTTTAGCTTTGTTGAGAATC

>b.27

TGCAGGTTCTGTCTAGTTTCCTTATCTCTATTGGGCAGGTCACAATTTGTTTCTGCAGAGCTGAGCTGCAGGTTCATTATCACAAAGGCACCAG

>b.28

CAGGTCAAAGTTCTGCTGAGGTTCTGCTAACGAGTCATCAGTTGGTCCAGTTGAGTTGAACCAGAACCAAAGCATGCAGGATCCGGGTCCTGCA

>b.29

TGCAGGCTTTGGGGGAAACGTTGTTCTCTGCAAACACTGAACCTGGTTCTGAGTTCTGATGACTCAGTTCAACACATCAGCTCCCAATCTGCCA

>b.30

GTTTCTAATTTTTTAAAGACTCGTCAGATGAAAATCTTTTTAAGCCTGGCTGAGTTATTTTTAACATCTTTCATGTCGCGCCTTCAAACCTGCA

>b.31

CAAGGTTCTCTTAATCTTTTTTTTTTTCCACTGGAAAGGATGAGAAGTTGTGCTTTTCTGCCTTTGTCTGTGGGGGATATGTGTTTACCCTGCA

>b.32

GGCAATATTATGGACATTGTGAAGGAAAGAAATGACTTTATGATTAAGTCCAAATGAAGATCGGCATTGAACGCACGTAAAACCAAAGCCTGCA

>b.33

TGCAGGAACAACAGTAACCAGTAGAATACAATATGGGACGACATAATTACCTTCACTCCCGAGTTTCTCTTCACTTGTCCTACACTCATGACCA

>b.34

TGCAGGGTGACTTCCTAATTAAAATGAAGCGTATGGTTCTGGTTCCATTGTTCTGATGATTTCACTCATACAAGCAGCAGCAGCAGCAGCAGCA

>b.35

TGCAGGATAAAAGCAGCTTTTTGAAGCGTCAGTGAGTCGGTGATCTTTTCCATTTGGCATAAATCTCTTACTGTCTGAACCCCTGAGTCGTAGA

>b.36

TGCAGGGGGCCAGGAGCCGGGCGCCGCGCCCTCCAGGGTCATCGTGGACATGCGGGAGTTCCGCAGCGAGCTGCCGGCGCTGCTGCACCGCCGT

>b.37

GAATGTCTTTGTTTTTTGGTTTTTCAGAAACTCTTTTCTATAACTAGTCTGAACTCCGTCAGGTTGTTGGTGTGAAATATTGGCCTCTCCTGCA

>b.38

TAAGGAAATCCAGCATATTGATGCTAACATGTTCAGCTAGCGGTGGGCTCCCTAAAGCTCACAGCAGCTGAGAACTACTCATGATGAACCTGCA

>b.39

TGCAGGCAACACACACTCCCTCCGCCACACACACACACACACATCGTGGTTAGACCTCCAATTAAATCAAAAGTGGTTTGTTTTTATTGGTCTT

>b.40

TGCAGGAGACACGGATGCTCTGTCTCCACAGTCAAGCGGCTCCCTGAGACTGGAACTGAGATTTTGGAGACCTCTTCCCACCACAGTCACATTG

>b.41

AGTCTGGCATAGGCATTGAGTTTGTAAAAGGAGTTTGCAATAAGTCATTCATTTGAATCCGGTGTGTTGGAGCAGGATGCATCTAAAACCTGCA

>b.42

TGCAGGATAAAATGAGTGTGCAAACCAAAATATTTTTATAGGTTCTGAATTATTCAGAAATTGAGCTGAAGAGCATTGGCTCATTTTCATAAAA

>b.43

AGAATAACTTGTAAAGGTCCAGACTGCTTAGAGACAAGCGTTCAAAATCATCACATAAAGTGAATCTGGAGCACTTTTGTTCTCTGAACCTGCA

>b.44

GGCTTGTCATTTTTTGCCTTGTTACAAATCCCAGCTGATTTAAAAGTGGTAAAACACCATTAGTGCTTGCCTTCTCTGAACTTACAAACCTGCA

>b.45

TGCAGGTAACATTCAAATAGACCTTATTGTGAGAAATAATTTTTGAAGAGCTCATTTTGTTATCTTGGTTTACGCTTGTACATTTAGTTAATCT

>b.46

TGCAGGCTCACTACCAGAGATCCAAGCCGCCCATGTAACCAATGTAACGGCTTCTCCAAATCCTCAGGAGCCTGGACTACAGATACAGGATTGG
