# Supplementary material for: Identification of the male-specific region on the guppy Y Chromosome from a haplotype-resolved assembly
Source: Genome Res. 2025 Mar;35(3):489–98. doi: 10.1101/gr.279582.124 (PMC11960691; doi:10.1101/gr.279582.124)
Supplement: Supplement 14 [file Supplemental_Material.docx]

# **Supplemental Material**

**Identification of the male specific region on the guppy Y chromosome from a haplotype resolved assembly**

Kang Du^1^, Oliver Deusch^2^, Ilja Bezrukov^2^, Christa Lanz^2^, Yann Guiguen^3^, Margarete Hoffmann^2^, Anette Habring^2^, Detlef Weigel^2^, Manfred Schartl^1,4,5^*, Christine Dreyer^2^*

^1^Xiphophorus Genetic Stock Center, Texas State University, TX78666, USA

^2^ Max Planck Institute for Biology Tübingen, Department of Molecular Biology, 72076 Tübingen, Germany

^3^INRAE, LPGP, 35073 Rennes, France

^4^Theodor Boveri Institute, Developmental Biochemistry, Biocenter, University of Würzburg, 97074 Würzburg, Germany

^5^Research Department for Limnology, University of Innsbruck, 5130 Mondsee, Austria

*Co-corresponding authors
^4^[phch1@biozentrum.uni-wuerzburg.de](mailto:phch1@biozentrum.uni-wuerzburg.de)
^2^christine.dreyer@tuebingen.mpg.de

## **Reordering of Guanapo X/Y chromosome 12**

As guide for assembly of Y-specific contigs the published chromosome 12 contigs [(Fraser et al. 2020)](https://paperpile.com/c/d5FAn1/9rMq) were rearranged according to the Hi-C-data, and few unpublished scaffolds were added (Supplemental Table S1). Contig IV was omitted because no contact information for Contig IV was available in the Hi-C data. Contact information for Contig XIII remained inconclusive with respect to its direction, probably because no Hi-C information on unplaced scaffolds was reported. Unplaced scaffolds 250F_0, 181F_0 and 149F_0 are lacking near the end of Chromosome12 LR880656.1, as confirmed by additional genetic markers [(Whiting et al. 2022)](https://paperpile.com/c/d5FAn1/rwxoB). Further the XY contigs IV and XII appeared to be partially redundant with respect to gene content, especially of *cyclin I* and *cyclin G2*, and after trimming only a single copy of cyclin I and of *cyclin G2* was supported on Y_mac_ and Y_gua_ haplotype contigs. Chromosome 12 LR880656.1 was assembled from an XY male and two phases were collapsed in the assembly, but these could not be assigned as being X or Y, respectively. Therefore, we cannot exclude that some apparent duplications are due to allelic variation.

In addition, we tentatively inserted three unplaced scaffolds, 200F_0, 250F_0 and 149F_0. These scaffolds are partially (200F_0) or predominantly syntenic with *X. maculatus* chromosome 8 and should therefore be part of chromosome 12 at the respective positions.

## **Assembly of the Guanapo and Maculatus Y chromosomes**

Guanapo and Maculatus haplotype contigs were compared to each other by dotplots using D-Genies [(Cabanettes and Klopp 2018)](https://paperpile.com/c/d5FAn1/BFTjS). They were also aligned to a version of the published X/Y chromosome 12 that was rearranged according to Hi-C data (Supplemental Table S1). D-Genies also provides associations that list all contigs that could be part of a given chromosome, irrespective of their length. Summing up the lengths of all contigs associated with chromosome 12 would result in a total length of about 37 Mb for Y_gua_ and 39 Mb for Y_mac_. These unrealistically high values are due to overlapping sequences near contig ends and additional shorter contigs with partial overlaps. In addition, some long contigs that clearly belong to another chromosome contain parts that were seemingly associated to chromosome 12, mostly due to the content of highly similar repeats (data not shown). Although for simplicity we omitted short contigs from the primary assembly, further refinement is required. After closer analysis of gene contents such corrections became obvious (see below).

After trimming the Guanapo and Maculatus haplotype contigs were assembled using the rearranged version of LR880656 as template (Supplemental Table S1). We mapped the parental sequences from which genetic markers were derived on the resulting Y_gua_ and Y_mac_ chromosomes. This revealed only a few minor differences between the genetic and the physical map. The reconstructed Y_gua_ and Y_mac_ haplotypes were aligned to the published male X/Y chromosome 12 (LR880656.1) and female LG12 (Figure 2) as well as to two different rearranged chromosome versions of LR880656.1 (not shown). This suggested that the published contigs IV and XII are partially redundant or allelic in the XY guppy. Since contig IV (placed to an anterior position of chromosome 12 by [Fraser et al. 2020)](https://paperpile.com/c/d5FAn1/9rMq) contains the male-specific SNPs of *cyclin I* and *shroom 3*, this contig is more likely Y-specific than contig XII. All contigs from LR880656.1 and the unplaced contigs may consist of mixed X and Y reads. Unplaced scaffolds 200F_0 (rc) and 149F_0 are required for optimal fit with Y haplotypes, but scaffold 250F is partially redundant. Telomere repeats are found at the start of Gua_tig00000738 (1 kb) and of Gua_tig000001309 (3.2 kb), but not on the corresponding Maculatus contigs nor on the published X/Y chromosome LR880656.1. Telomere repeats are also present on the unplaced XY scaffold_149F_0, which aligns to Gua_tig000001309 (Supplementary Fig S3).

## **Structure comparison of distal X and Y haplotype regions**

Supplemental Fig S3 shows the distal regions of the alignments in more detail. Two unplaced scaffolds of the published female genome, namely NW_007615031.1 at about 23.2 to 23.3 Mb and NW_007615023.1 at about 26.2 are now integrated into the Y-guided X_gua_ assembly. It was noted previously, that by synteny with the *Xiphophorus maculatus* chromosome 8 these scaffolds should be placed to that specific region [(Charlesworth et al. 2020)](https://paperpile.com/c/d5FAn1/tnjEs). The previously unplaced XY male scaffold 149F_0 is a longer counterpart of the female NW_007615031.1 and it aligns well to contig Gua_1309 (Supplemental Fig S3).

Differences between haplotypes and between X and Y chromosomes near the chromosome ends could indicate sequence divergence or might be due to additional short contigs missing. A detailed analysis of alignment gaps in the distal region is presented in Supplemental Fig S3.

## **Comparison of Y_gua_ and Y_mac_ gene content**

All genes annotated on Y_gua_ and Y_mac_ are listed in Supplemental Table S5.

Some discrepancies between both haplotypes are seen where genes of one haplotype are on short contigs. On the other hand, binning errors may have caused seeming gene duplications on Y_gua_.
E.g., at about 11.9 Mb, *sphingosine 1-phosphate receptor 3-like* (g416 and g417), *semaphorin-4D* (g419 on Y_gua_), and the genetic marker_90 are on Y_gua_ but not found on Y_mac_. These genes are found on short Maculatus contigs Mac_tig00000312, Mac_tig00000379 and Mac_tig00000994 that have to be included adjacent to Mac_tig00000993 to fully cover the “missing” genes. Among the shorter Guanapo contigs, Gua_tig00000158 also contains *semaphorin-4D* but is fully covered by Gua_tig00001219 and therefore be redundant.

## **Candidate sex-specific genes in the putative MSY**

Genes seemingly missing or impaired on the X chromosome are listed in Supplemental Table S6.

For bile-salt-activated lipase two copies are annotated on Y_mac_ (g773, g774) and Y_gua_ (g775, g776) but only g776 is complete on X_gua_.

The *pumilio3-like* gene (Y_mac_ g855, Y_gua_ g858) has 17 exons, only 12 of which were annotated on X_gua_ or LG12. The incomplete female version would be defective in its functional domains as an RNA binding protein. *Pum3* has been implicated in germ cell specification in *Drosophila* [(Forbes and Lehmann 1998)](https://paperpile.com/c/d5FAn1/j9vYK), self-renewal of germ cells in *C. elegans* [(Ferdous et al. 2023)](https://paperpile.com/c/d5FAn1/uFuRi), and preRNA processing in mammals (reviewed by [(Goldstrohm, Hall, and McKenney 2018)](https://paperpile.com/c/d5FAn1/ApCZy).

The heat shock protein gene *dnaj* c25 (Ymac g864; Ygua g870) consists of five exons on both Y haplotypes but the first one of five exons was not found on LG12 or on the reassembled X chromosomes.

*Cyclin I* (Y_gua_ g876) and *shroom 3* (Y_gua_ g879) contain male-specific SNPs that can be used as reliable markers for sex in many guppy populations [(Sharma et al. 2014;](https://paperpile.com/c/d5FAn1/ZxHCm) and unpublished observations of MH and CD). These SNPs are distinct between contigs IV and XII of LR880656.1. Based on SNPs, contigs IV might contain the Y copy of *cyclin I* and contig XII the X allele. The *cyclin I* gene on Y_gua_ has the same SNPs as the ones seen on contig IV but *cyclin I* on Y_mac_ shares the SNPs of the putative X_gua_ allele. *Cyclin I* and *shroom 3* are on contigs Gua_tig00000808 and Mac_tig00000982. While Gua_tig00000808 has the characteristic Guanapo male SNPs for these two markers, this is not the case on Mac_tig00000982. For *shroom 3* all 30 exons annotated on the Y-chromosomes are also present on the reassembled X_gua_, although only 26 of these exons were found on LG12.

The *spin-1-like* gene (Y_mac_ g879, Y_gua_ g886) contains a Ssty domain and has been implicated in spermatogenesis [(Staub, Mennerich, and Rosenthal 2002)](https://paperpile.com/c/d5FAn1/4wcIJ). The first three of its five exons could neither be located on LG12 nor on the X_gua_. For the *zer1* gene that was discussed to be partially sex-linked [(Charlesworth, Bergero, et al. 2021)](https://paperpile.com/c/d5FAn1/Bcfb) one of its 15 exons was not detected on the female LG12.

Almost all exons that were seemingly missing on the X-chromosome could be reconstructed from the female raw reads or retrieved from female pool-sex reads (Supplemental Table S6). For *bsa lipase* (Ygua g775), *gadd45g* (g836), *spin1* (g886), and *zer1* (g980) presence of those exons that were not retrieved from the published female LG12 was however unequivocally shown by genomic PCR (Supplemental Table S7).

## **Assembly of autosomes**

All autosomes were reconstructed from Guanapo and Maculatus trimmed contigs using the published male autosomes [(Fraser et al. 2020)](https://paperpile.com/c/d5FAn1/9rMq) as guide (Supplemental Fig S1). In general, only a few haplotype contigs were sufficient to reconstruct a Guanapo or Maculatus autosome. Even a single long haplotype contig was sufficient to cover a whole chromosome (chr 17) or most of it (chromosome 5, 8, 9, 11, 19, 21, 23).

The observation that most of the Guanapo chromosomes are longer than their Maculatus counterparts suggests a potential bias of the binning procedure. Length differences are apparent for guppy chromosomes 3, 4, 9, 10,11, 14, 15,16, 18, 19, 20 and 22. Unfortunately, mis-binned contigs cannot easily be sorted out because they might consist of mixed reads from both parents. Some gaps in the alignments between both haplotypes are, however, likely caused by binning errors: asterisks on chromosome 4, 9 and 11 alignment gaps mark regions where the D-genies dot plots reveal many short Guanapo contigs that are partially congruent with longer contigs. Closer analysis showed that the shorter contigs are prone to misbinning, as indicated by more mismatches with published *P. reticulata* coding sequences (data not shown).

Three long and additional short gaps between the Guanapo and Maculatus haplotypes add up to a difference of ~10 Mb in chromosome 4 length. Binning errors might be a plausible reason, since coding genes at 6.5 to 7.2 Mb are each found on two Guanapo haplotype contigs but not on any Maculatus contig (data not shown).

We aligned the guppy chromosomes also to their *Xiphophorus* orthologues because these were of outstanding quality among published Poeciliid genome assemblies. An alignment gap of more than 3Mb length in the proximal region of chromosome 9 resembles in length and position an alignment gap between the orthologous chromosomes 12 of *X. hellerii* and *X. maculatus*. The distal region of *X.hellerii* chromosome 12 may be subject to interchromosomal translocation between *Xiphophorus* species [(Lu et al. 2023)](https://paperpile.com/c/d5FAn1/BhML). *X. hellerii* chromosome 11 aligns to both guppy chromosome 14 haplotypes over their entire lengths, yet with two substantial portions being reversed and translocated. Alignments of Guanapo chromosome 11 to *X. hellerii* chromosome 13 confirms one guppy-specific and two *X.maculatus*-specific inversions.

**Supplemental Tables**

Supplemental Table S1: Rearrangement of XY Chromosome 12 (LR880656.1)

Supplemental Table S2: Primary construction of Y-haplotypes from long contigs

Supplemental Table S3: Repeat content in pseudoautosomal region (PAR) and MSY of Ygua and Ymac

Supplemental Table S4: Ygua genes expressed with male bias

Supplemental Table S5: Genes annotated on Ymac and Ygua

Supplemental Table S6: Y-chromosomal genes annoted as incomplete on female LG12

Supplemental Table S7: Genomic PCR test for presence of genes on female genome

# **
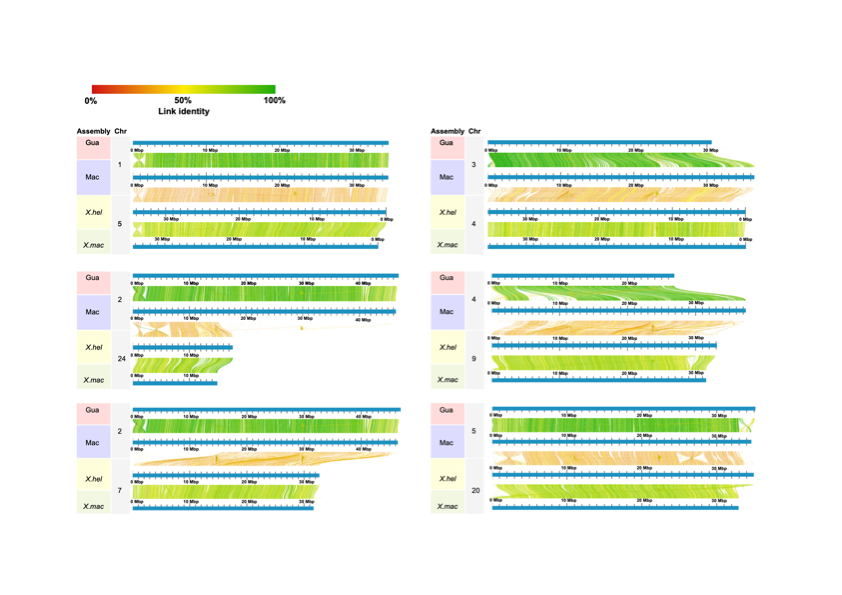
Supplemental Figures**

## **Supplemental Fig S1: Assembly of all autosomal haplotypes**

All autosomes were reconstructed from Guanapo and Maculatus haplotype contigs using the published male autosomes [(Fraser et al. 2020)](https://paperpile.com/c/d5FAn1/9rMq) as guide. They were then aligned to the published male and female guppy chromosomes (left) and to their *X. hellerii* and *X. maculatus* orthologs [(Lu et al. 2023)](https://paperpile.com/c/d5FAn1/BhML). Differences in length between Guanapo and Maculatus haplotypes (e.g. of chromosome 4) are considered to be mostly due to binning errors (data not shown).

## **Supplemental Fig S2: D-Genies dot plot analysis of trimmed Guanapo (Gua) and Maculatus (Mac) chromosome 12 contigs**

All Maculatus and Guanapo contigs were plotted against each other. Only contigs that are longer than 200 kb and matching chromosome 12 are shown. Numbers of Mac_tigs from left to right (last 4 digits): 0304,0950, 0082, 0993, 0951, 0028, 0315, 0982, 0193; Gua_tigs from bottom to top: 0738, 1218, 1219, 1220, 0294, 0770, 1198,1197, 0808, 1309, 1310. The arrow points at Mac_tig00000403 and Gua_ tig00001195 that may consist of mixed sequence, parts belonging to chromosome11. Further analysis showed that Mac_tig00000193 and Gua_tig0001309 plus Gua_tig00001310 must be reverse complement (see also Supplemental Fig S3 and Supplemental Table S2).

## **Supplemental Fig S3: Comparison of Y_gua_ and X_gua_ chromosome ends including female scaffolds**

Chromosome ends are to the left. NW_007615031.1 (scaffold 241) and NW_007615023.1 (scaffold 219) are unplaced scaffolds belonging to the Guanapo X-chromosome NC_024342.1 [(Charlesworth et al. 2020)](https://paperpile.com/c/d5FAn1/tnjEs). XY scaffold 000149F_0 is an unplaced scaffold not included in the published X/Y Chr_12 LR880656.1. Scaffold_ 241 and scaffold_ 219 were integrated in the X_gua_ chromosome by the reassembly process. The distal alignment gaps between Y_gua_ and X_gua_ are characteristic for the chromosome ends. Total length difference between Y_gua_ and X_gua_ is about 4.2 Mb.

## **Supplemental Fig S4: Analysis of alignment gaps between distal X_gua_, Y_gua_, and Y_mac_**

Alignment of the distal regions of the reconstructed X_gua_ ,Y_gua_ and Y_mac_. Some of the alignment gaps could be analyzed in detail: a; X_gua_, and Y_gua_ match; on Y_mac_ three short contigs are required to fill this gap: Mac_tig00000293, Mac_tig00000376, Mac_tig00000600. b; On Y_gua_, *wdr7*, *TATA box bp* (g870, g871, on Ymac) and the last exon of *cyclin G2* (g875 on Ygua) missing, already before trimming. c; two protein coding genes missing on Y_mac_ (g883 and 884 on Y_gua_). Mac g878 and Gua g885 are paralogs of *rabepK* (Mac g448 and Gua g455). d; Y_gua_ genes g919, g921 to g927 and g934 to g979 annotated as uncharacterized gene are without definite orthologues on Y_mac_. e; 12 Gua gff g1000 to g10011 around *septin_1* not found on Y_mac_, and also not on other shorter Mac_contigs. Corresponds to part of female scaf_219 (NW_007615023.1). Additional alignment gaps between 24.3 and 25.5 Mb represent gene-poor regions rich in repeats.

## **Supplemental Fig S5: Sex specific SNP density**

PoolSex data from a population of *P. obscura* were mapped on the Y_gua_. Note that less of the male than female SNPs from *P.obscura* map to the distal end of Y_gua_ between 26 and 28 Mb.

## **Supplemental Fig S6: Repeats on Y_gua_ and Y_mac_**

Repeat content (a), Kimura values (b), and frequency of Helitron-4 (c) along chromosome Y_gua_ and Y_mac_ in 100 kb sliding windows. As an age index the TEs Kimura values were calculated using repeatMasker. Kimura values are significantly smaller in the region from 22 Mb (red dotted line) to the chromosome ends (Mann-Whitney U test, p<2.2e-16) for both haplotypes, indicating recent accumulation of TEs in these regions. This accumulation of young repeats extends proximal of the sharp border of X/Y sequence divergence at 23.5 Mb. Helitron-4 elements accumulated at different locations of the MSY of Ygua and Ymac.
